# The Complexity and Dynamics of the Tissue Glycoproteome Associated With Prostate Cancer Progression

**DOI:** 10.1074/mcp.RA120.002320

**Published:** 2021-01-05

**Authors:** Rebeca Kawahara, Saulo Recuero, Miguel Srougi, Katia R.M. Leite, Morten Thaysen-Andersen, Giuseppe Palmisano

**Affiliations:** 1 Departamento de Parasitologia, Instituto de Ciências Biomédicas, Universidade de São Paulo, USP, São Paulo, Brazil; 2 Department of Molecular Sciences, Macquarie University, Sydney, NSW, Australia; 3 Biomolecular Discovery Research Centre, Macquarie University, Sydney, NSW, Australia; 4 Laboratório de Investigação Médica da Disciplina de Urologia da Faculdade de Medicina da USP, São Paulo, Brazil

**Keywords:** glycosylation, glycomics, glycoproteomics, MS, prostate cancer, ACN, acetonitrile, AGC, automatic gain contro, BPH, benign prostatic hyperplasia, C2GNT, core 2 β1,6-N-acetylglucosaminyltransferase, ECM, extracellular matrix, FA, formic acid, FWHM, full width at half maximum, GnT, N-acetylglucosaminyltransferase, GS, Gleason score, HCD, higher-energy collision-induced dissociation, LAMA5, laminin subunit α-5, LTF, lactotransferrin, Neu5Gc, N-glycolylneuraminic acid, OST, oligosaccharyltransferase, PAP, prostatic acid phosphatase, PCa, prostate cancer, PGC, porous graphitized carbon, PSA, prostate-specific antigen, SPE, solid phase extraction, TFA, trifluoroacetic acid

## Abstract

The complexity and dynamics of the immensely heterogeneous glycoproteome of the prostate cancer (PCa) tumor microenvironment remain incompletely mapped, a knowledge gap that impedes our molecular-level understanding of the disease. To this end, we have used sensitive glycomics and glycoproteomics to map the protein-, cell-, and tumor grade–specific *N*- and *O*-glycosylation in surgically removed PCa tissues spanning five histological grades (*n* = 10/grade) and tissues from patients with benign prostatic hyperplasia (*n* = 5). Quantitative glycomics revealed PCa grade–specific alterations of the oligomannosidic-, paucimannosidic-, and branched sialylated complex-type *N*-glycans, and dynamic remodeling of the sialylated core 1- and core 2-type *O*-glycome. Deep quantitative glycoproteomics identified ∼7400 unique *N*-glycopeptides from 500 *N*-glycoproteins and ∼500 unique *O*-glycopeptides from nearly 200 *O*-glycoproteins. With reference to a recent Tissue and Blood Atlas, our data indicate that paucimannosidic glycans of the PCa tissues arise mainly from immune cell–derived glycoproteins. Furthermore, the grade-specific PCa glycosylation arises primarily from dynamics in the cellular makeup of the PCa tumor microenvironment across grades involving increased oligomannosylation of prostate-derived glycoproteins and decreased bisecting GlcNAcylation of *N*-glycans carried by the extracellular matrix proteins. Furthermore, elevated expression of several oligosaccharyltransferase subunits and enhanced *N*-glycoprotein site occupancy were observed associated with PCa progression. Finally, correlations between the protein-specific glycosylation and PCa progression were observed including increased site-specific core 2-type *O*-glycosylation of collagen VI. In conclusion, integrated glycomics and glycoproteomics have enabled new insight into the complexity and dynamics of the tissue glycoproteome associated with PCa progression generating an important resource to explore the underpinning disease mechanisms.

## Highlights

• Glycomics and glycoproteomics of PCa tissues during disease progression.
• Cell-specific dynamics of pauci- and oligomannosylation during PCa progression.
• Increased *N*-glycan branching and core 2-type *O*-glycosylation in ECM glycoproteins.
• Increased *N*-site occupancy and oligosaccharyltransferase expression in PCa.

## Graphical Abstract

**Figure:**
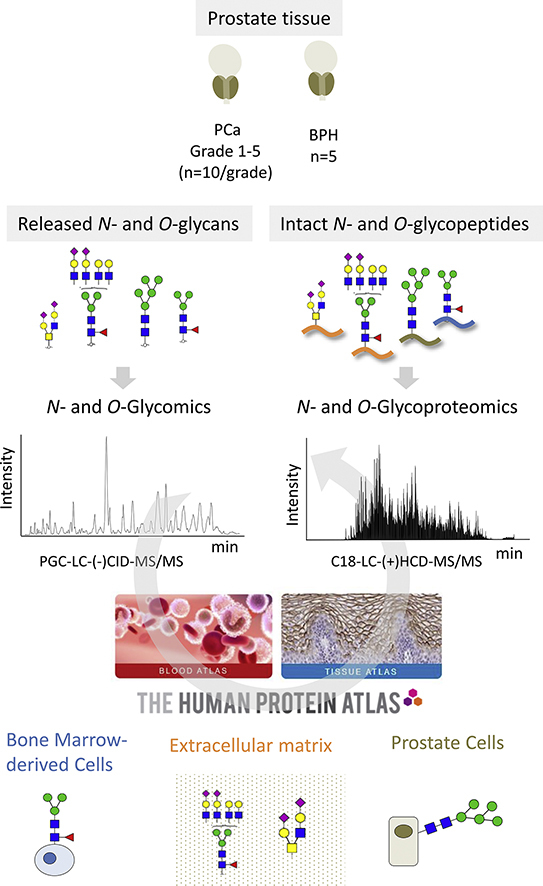

## In Brief

Integrated glycomics and glycoproteomics with reference to the established Tissue Atlas were used to uncover the cell-, protein-, site-, and structure-specific *N*- and *O*-glycosylation in prostate cancer tissues spanning five disease grades relatively to benign prostatic hyperplasia control tissues. Dynamic cell-specific changes in the paucimannosylation and oligomannosylation of known bone marrow– and prostate-derived glycoproteins, respectively, and increased *N*-glycan branching and core 2-type *O*-glycosylation of known extracellular matrix glycoproteins were found to be key changes associated with prostate cancer progression.

Prostate cancer (PCa) is the second leading cause of cancer death in men worldwide (1). PCa is a highly heterogeneous disease leading to a variety of clinical outcomes spanning latent to highly aggressive disease forms (2). The PCa cells are surrounded by a heterogenous stroma comprising a complex extracellular matrix (ECM) and different types of cells, including immune cells, fibroblast cells, and endothelial cells, which dynamically modulate the tumor phenotype (3).

The gold standard for PCa diagnosis and prognosis is still based on a subjective histological examination of prostate tissue. PCa tissues are by convention classified into five grades, *i.e.*, grade 1 to 5 (G1–G5) based on the Gleason score (GS) of the architectural patterns of the prostate adenocarcinoma tissue (4, 5). The inherent variability of tumor sampling associated with the intratumoral heterogeneity is a severe limitation for accurate PCa prognostication (6). As recently reviewed (7), PCa biospecimens, including serum, urine, and tissues, have repeatedly been subjected to a variety of omics-type analyses with the aim to find novel quantitative molecular signatures to more accurately detect and stratify PCa. We have recently performed grade-specific proteome profiling of PCa tissues spanning G1 to G5 (8). In short, we identified key biological processes being altered during PCa progression, including cell adhesion, osteoblast differentiation, and blood coagulation. We reported in that study a panel of 11 proteins able to stratify patients with low and high PCa grades. Despite the growing list of reported candidate biomarkers, none has replaced the blood prostate-specific antigen (PSA) currently used as a biomarker for PCa screening despite its relatively low specificity and limited prognostic value (9, 10).

Building evidence indicates that not only the protein expression but also the glycans decorating proteins are altered in PCa (11, 12, 13). Such molecular aberrations generate distinct signatures associated with PCa that may be used for more accurate PCa diagnosis and prognosis by being more quantitative features that are less prone to inconsistent sampling (14). PSA glycoform profiling has been shown to provide better accuracy in detecting PCa compared with the conventional protein-level analysis of PSA in blood (15), which demonstrates the potential of using glycoprofiling to discover new and improve existing PCa biomarkers.

Glycans cover all cell surfaces including the surface of PCa tumor cells. In fact, the glycocalyx forms the interface between the tumor cells and the microenvironment, thus enabling communication between cells within the tumor and the ECM (16, 17). It has been demonstrated that glycans mediate cellular events associated with hallmarks in cancer, such as invasion and metastasis, immune evasion, activation of endothelial cells, and induction of angiogenesis (18). Aberrant glycosylation occurring in both the cancer cells and the surrounding cells facilitates a dynamic and complex glycan-mediated crosstalk within the tumor microenvironment of importance to PCa onset and progression (19).

Characterization of protein glycosylation remains analytically challenging because of the nontemplate-driven biosynthesis dictated by competing glycosyltransferases and glycoside hydrolases, which produce extensive structural diversity of structurally related glycoforms. It is widely recognized that comprehensive glycoprofiling requires multiple orthogonal analytical approaches (20, 21). MS-based glycomics and glycoproteomics have emerged as powerful analytical tools to quantitatively map the glycan fine structures and the site-specific microheterogeneity and macroheterogeneity of glycoproteins at scale in complex biological samples (22, 23, 24, 25). Indeed, both glycomics and glycoproteomics have already been utilized to profile different features of protein glycosylation in PCa. Glycomics profiling revealed that core fucosylation and α2,3-sialylation are elevated features in PCa sera relative to benign prostatic hyperplasia (BPH) sera (26). Elevation of triantennary and tetraantennary *N*-glycans on serum proteins was also found to be predictors of castration-resistant PCa (27). In addition, *N*-glycomics revealed that highly branched sialylated *N*-glycans of urinary proteins are elevated features associated with high-grade PCa (GS = 9) relative to low-grade PCa (GS = 6) (28).

In separate efforts aiming to identify diagnostic and prognostic glycoprotein biomarkers for PCa, peptides were profiled using conventional proteomics after de-*N*-glycosylation of proteins extracted from tissue, urine, and serum obtained from PCa patients and control cohorts (29, 30, 31). A panel of serum and urinary glycoproteins with the potential to detect PCa, predict the PCa grade, and discriminate between aggressive and nonaggressive PCa tissues was reported. Furthermore, intact glycopeptide profiling of two PCa cell lines, LNCaP and PC3, representing cell line models for androgen-dependent and androgen-independent PCa, respectively, demonstrated a higher site-specific fucosylation and altered site occupancy of glycoproteins in PC3 cells (32). Finally, we recently used glycoproteomics to identify more than 900 *N*- and *O*-glycopeptides from urine glycoproteins, of which a panel of 56 *N*-glycopeptides was able to discriminate PCa and BPH (33). Concertedly, these studies have considerably advanced our knowledge of protein glycosylation in PCa, but the glycan fine structures, their site-specific microheterogeneity and macroheterogeneity, and their dynamics and cellular origins within the complex tumor environment during PCa progression remain unexplored.

To this end, we here apply integrated glycomics and glycoproteomics to investigate a clinically annotated collection of surgically removed fresh PCa tissues spanning five grades (G1–G5) and BPH tissues as a relevant clinical reference. This is the first study to report both on the glycan fine structures and the site- and cell-specific expression and dynamics of *N*- and *O*-glycosylation during PCa progression.

## Experimental Procedures

### Ethics

The study was approved by the ethics review board of the Faculdade de Medicina da Universidade de São Paulo under the protocol no. 2.695.126. Written informed consents were obtained from all participants. All experiments were performed in accordance with the approved guidelines and regulations.

### Patient Cohort, Tissue Collection, Storage, Isolation, and Protein Extraction

All prostate surgical specimens were examined by a trained uropathologist. Fresh tissues were collected after radical prostatectomy from a total of 55 patients, which included 10 patients from each PCa grade (G1–G5) and five individuals treated surgically for BPH. The PCa grades were determined using the modified Gleason grading system proposed by the International Society of Urological Pathology (34). The samples included in this study were only used for discovery phase research. Details of the sample cohort are available in our previous study, where label-free quantitative proteomics profiling was carried out on the same samples (8) and can be also found in supplemental Table SA. Fresh prostate tissues (∼60 mg) stored in RNAlater for improved stability were washed in 80% (v/v) cold acetonitrile (ACN) and resuspended in an extraction buffer containing 8 M urea, 10 mM DTT, 1 mM sodium fluoride, 1 mM sodium orthovanadate, and a protease inhibitor cocktail (1:10). Prostate tissues were rapidly lysed (2 min, 30 Hz with a 5 mm stainless steel bead) using a TissueLyser (Qiagen). Protein concentrations were determined by the Qubit fluorimetric detection method (Thermo Fisher) and immediately used for glycomics and glycoproteomics.

### Glycan Release

Extracted proteins (20 μg) were reduced using 10 mM DTT, 30 min, 30 °C, and alkylated using 40 mM iodoacetamide 30 min in the dark. The proteins were blotted on a primed 0.45 μm polyvinylidene fluoride membrane (Merck Millipore). The *N*- and *O*-glycans were sequentially released as described (35). Briefly, protein spots were stained using Direct Blue (Sigma–Aldrich), excised, and transferred to a flat bottom polypropylene 96-well plate (Corning Life Sciences), blocked with 1% (w/v) polyvinylpyrrolidone in 50% (v/v) methanol and washed with MilliQ water. The *N*-glycans were then released using 10 U recombinant *Elizabethkingia miricola N*-glycosidase F (Promega; V4831, 10 U/μl), 16 h, and 37 °C. The detached glycans were reduced with 1 M sodium borohydride in 50 mM aqueous potassium hydroxide for 3 h at 50 °C. The reaction was stopped using glacial acetic acid, and the glycans were desalted using strong cation exchange/C18 and porous graphitized carbon (PGC) solid phase extraction (SPE). The same protein spots were then incubated with 0.5 M sodium borohydride in 50 mM potassium hydroxide at 50 °C for 16 h to release and reduce the *O*-linked glycans by reductive β-elimination. The *O*-glycans were desalted as described previously.

### Glycan Analysis

PGC–LC–MS/MS–based glycan analysis was performed on an UltiMate 3000 HPLC system (Dionex, Sunnyvale, CA, USA) interfaced with a linear trap quadrupole Velos Pro ion trap (Thermo Scientific, San Jose, CA, USA). The glycans were loaded on a PGC HPLC capillary column (Hypercarb KAPPA; 5 μm particle size, 200 Å pore size, 180 μm inner diameter × 100 mm length; Thermo Scientific) operated at 50 °C with a constant 4 μl/min flow rate. About 10 mM aqueous ammonium bicarbonate solution, pH 8 (solvent A), and 10 mM aqueous ammonium bicarbonate in 70% ACN (solvent B) were used as mobile phases using the following gradient for *N*-glycans: 8 min at 2.6% B, 2 min from 2.6 to 13.5% B, 45 min from 13.5 to 55% B, 15 min from 55 to 70% B, 1 min from 70 to 98% B, 5 min at 98% B, 1 min from 98 to 2.6% B and 6 min at 2.6% B as well as *O*-glycans: 5 min at 2.8% B, 33 min from 2.8 to 30% B, 5 min from 30 to 98% B, 5 min at 98% B, 2 min from 98 to 2.8% B, and 5 min at 2.8% B.

The electrospray ionization source was operated in negative-ion mode with source potential of +3.2 kV. Full MS scans were acquired using a mass range *m*/*z* 570 to 2000 for *N*-glycans and *m*/*z* 380 to 1800 for *O*-glycans, 3 microscans, *m*/*z* 0.25 full width at half maximum (FWHM) resolution, 5 × 10^4^ automatic gain control (AGC), and 50 ms accumulation. For the MS/MS settings, *m*/*z* 0.35 FWHM resolution; 2 × 10^4^ AGC, 300 ms accumulation, and 2 *m*/*z* precursor ion window. The five most abundant precursors in each full scan spectrum were selected for collision-induced dissociation-based MS/MS using normalized collision energy of 33% with an activation Q of 0.250 and 10 ms activation.

### Glycan Data Analysis

Glycan precursor ions were extracted using RawMeat, version 2.1 (Vast Scientifi; www.vastscientific.com) (36). Common contaminants and redundant precursors were manually removed. The *m*/*z* of the extracted monoisotopic precursor ions was converted to mass [M–H]– and searched against GlycoMod (http://www.expasy.ch/tools/glycomod) to identify putative monosaccharide compositions. The mass tolerance was set to 0.5 Da. Only compositions containing Hex, HexNAc, dHex, NeuAc, NeuGc, and glycans reported in the UniCarbKB database were considered. The glycan fine structures were manually elucidated using monoisotopic mass and the PGC LC elution and MS/MS fragment patterns. Extracted ion chromatogram–based glycan quantification was performed using Skyline, version 19.1, as described (37). GlycoWorkBench, version 2.1 (https://code.google.com/archive/p/glycoworkbench/) was used to aid the manual annotation of glycan fragment spectra and generate the glycan cartoons. Xcalibur, version 2.2 (Thermo Scientific) was used to browse and annotate raw LC–MS/MS data.

### Protein Extraction, Digestion, and Desalting

Proteins (500 μg) were reduced using 10 mM DTT, 30 min, 30 °C, and alkylated using 40 mM iodoacetamide (both final concentrations), 30 min in the dark at room temperature. The reaction was quenched using DTT. Proteins were digested using sequencing grade porcine trypsin (1:50, w/w; Promega), 12 h, 37 °C, and stopped using 1% trifluoroacetic acid (TFA). Peptides were desalted using hydrophilic–lipophilic balance SPE cartridges (Waters) and dried.

### TMT Labeling of Peptides

A pooled peptide reference sample containing peptides from all samples was generated for quantitative comparison across multiple tandem mass tag (TMT)-10plex experiments. The peptide samples were randomly combined in six TMT-10plex sets. Each set included at least one sample from each PCa grade and BPH. The reference sample was consistently labeled with the 126 Da reporter ion channel. In total, 54 peptide samples were labeled with TMT for quantitative proteomics, including five BPH samples, 10 PCa samples each from G1 to G4, and nine PCa samples from G5 (supplemental Table SB).

Peptides originating from 25 μg protein extracted from each sample were dissolved in 100 μl 100 mM triethylammonium bicarbonate buffer and labeled individually with 0.23 mg TMT-10plex mass tags (Thermo). In brief, the TMT10plex tags (0.23 mg) in 41 μl neat anhydrous ACN were added to the peptide samples and incubated for 1 h at room temperature. Reactions were quenched using 8 μl 5% (v/v) hydroxylamine, 15 min at room temperature. After TMT labeling, all peptide samples were mixed 1:1 (w:w), desalted using hydrophilic–lipophilic balance SPE cartridges (Waters), and dried. Aliquots of the TMT-labeled peptide mixtures from the six TMT-10plex experiments were analyzed without any prefractionation by LC–MS/MS (supplemental Table SB).

### Glycopeptide Enrichment

TMT-labeled peptides (∼250 μg) were reconstituted in 50 μl loading and washing solvent containing 80% ACN in 1% aqueous TFA. Peptides were loaded onto primed custom-made hydrophilic interaction liquid chromatography (HILIC) SPE microcolumns packed with zwitterionic HILIC resin (10 μm particle size, 200 Å pore size, kindly provided by Sequant/Merck, Umea, Sweden) onto supporting C8 disks (Empore) in p10 pipette tips (38). Flow-through fractions were collected. The HILIC microcolumns were then washed with 50 μl loading solvent, and the wash fraction was collected and combined with the flow-through fraction for separate downstream analysis. This fraction contained the nonmodified peptides. Glycopeptides were eluted with 50 μl 0.1% (v/v) TFA, 50 μl 25 mM aqueous ammonium bicarbonate, and then 50 μl 50% (v/v) ACN. The three fractions were combined to form mixtures containing enriched glycopeptides, which were dried, desalted on a primed Oligo R3 reversed phase SPE microcolumn, aliquoted, and dried (supplemental Table SB).

### Peptide Deglycosylation

Aliquots of the enriched glycopeptides were resuspended in 50 mM triethylammonium bicarbonate, pH 8.0. De-*N*-glycosylation was performed using 10 U recombinant *E. miricola* peptide:*N*-glycosidase F (Promega, V4831, 10 U/μl), 12 h, 37 °C. The de-*N*-glycosylated peptides were desalted on a primed Oligo R3 reversed phase SPE microcolumn and dried (supplemental Table SB).

### Glycopeptide Prefractionation

Enriched glycopeptides, de-*N*-glycosylated peptides, and nonmodified peptides were resuspended in 50 μl 25 mM ammonium bicarbonate for high pH prefractionation using Oligo R2 reversed phase SPE microcolumns packed on supporting C18 discs (Empore) in standard p10 pipette tips. The columns were washed three times with 50 μl of 100% ACN and 50 μl of 25 mM aqueous ammonium bicarbonate. Samples were loaded on the columns followed by two washing steps with 50 μl of 25 mM aqueous ammonium bicarbonate. The peptides were eluted in three fractions, *i.e.*, fraction 1: 10% ACN in 25 mM aqueous ammonium bicarbonate; fraction 2: 20% ACN in 25 mM aqueous ammonium bicarbonate, and fraction 3: 60% ACN in 25 mM aqueous ammonium bicarbonate, and dried. The peptides from the three fractions were resuspended in 0.1% (v/v) formic acid (FA) for separate LC–MS/MS analysis (supplemental Table SB).

### Peptide Profiling

TMT-tagged peptides were loaded on a trap column (2 cm × 100 μm inner diameter) custom packed with ReproSil-Pur C18 AQ 5 μm resin (Dr Maisch, Ammerbuch-Entringen, Germany). Approximately 1 μg of total peptide was injected per LC–MS/MS run. The peptides were separated at 250 nl/min on an analytical column (Reprosil-Pur C18-Aq; 25 cm × 75 μm, 3 μm ID; Dr Maisch, Ammerbuch-Entringen, Germany) using an UltiMate 3000 RSLCnano System. The mobile phases were 99.9% ACN in 0.1% (both v/v) aqueous FA (solvent B) and aqueous 0.1% (v/v) FA (solvent A). The gradient was 2 to 30% B over 100 min, 30 to 50% B over 18 min, 50 to 95% B over 1 min, and 9 min at 95% B. The nanoLC was connected to a Q-Exactive HF-X Hybrid Quadrupole-Orbitrap mass spectrometer (Thermo Fisher Scientific) operating in positive-ion mode. The Orbitrap was used to acquire the full MS scan with an AGC of 3 × 10^6^ ions and 50 ms accumulation. Full MS scans were acquired at high-resolution 60,000 FWHM at *m*/*z* 200 with an *m*/*z* range of 350 to 1800. The 20 most abundant precursor ions were selected from each MS full scan using data-dependent acquisition and fragmented utilizing higher-energy collision-induced dissociation (HCD) fragmentation with a normalized collision energy of 35%. Only multicharged precursors (*Z* ≥ 2) were selected for fragmentation. Fragment spectra were acquired at 45,000 resolution with an AGC of 1 × 10^5^ and 90 ms accumulation using a precursor isolation window of *m*/*z* 1.0 and a dynamic exclusion of 30 s after a single isolation and fragmentation of a given precursor ion.

### Analysis of Intact *N*-Glycopeptides

The HCD–MS/MS spectra of intact glycopeptides were searched with Byonic, version 2.6.46 (Protein Metrics, CA, USA) (39) using 10/20 ppm as the precursor and product mass tolerance, respectively. Cys carbamidomethylation (+57.021 Da) and TMT (+229.163 Da) at *N* terminus and lysine (K) were considered fixed modifications. Trypsin-specific cleavages were considered with a maximum of two missed cleavages per peptide. The following variable modifications were included: Met oxidation (+15.994 Da) and *N*-glycosylation of sequon-localized Asn with a predefined glycan database of 309 mammalian *N*-glycans without sodium adducts to which the paucimannose M3F (Man3GlcNAc2Fuc1) was manually added or *O*-glycosylation of Thr/Ser with a predefined *O*-glycan database containing 78 common mammalian *O*-glycans without sodium adducts available within Byonic. The MS/MS spectra were searched against a protein database composed of all reviewed UniProtKB human proteins (supplemental Table SB). All searches were filtered to <1% false discovery rate at the protein level and 0% at the peptide level by using a decoy database (40). Only glycopeptides identified with high score ID were considered (PEP 2D scores <0.001) (33). Glycopeptides identified with low confidence, in the reverse database and contaminant database, were excluded. Intact glycopeptides were quantified using the Report Ion Quantifier as a node in Proteome Discoverer, version 2.2 (Thermo Scientific). The abundances of the reporter ion intensities from the MS/MS scans were extracted from the QuantSpectra table. Glycopeptides were manually grouped by summing the reporter ion intensities from peptide spectral matches with the same UniProtKB identifier, same glycosylation site within the protein, and same glycan composition. The abundances of the unique glycopeptides from each channel were first normalized by dividing each unique glycopeptide reporter ion intensity by the reference reporter intensity within that specific experiment and further normalized by the total ratio intensity of each channel to correct for any variation in the total yield during the labeling reactions.

### Protein Identification and Glycosylation Site Analysis

For protein identification and quantification as well as glycosylation site analysis, the LC–MS/MS raw files were imported into MaxQuant, version 1.5.2.8 (41). The database search engine Andromeda (42) was used to search the HCD–MS/MS spectra against a database composed of all reviewed UniProtKB human proteins (downloaded July, 2017; 20,201 entries) with a precursor ion tolerance of 4.5 ppm and product ion tolerance of 20 ppm. Report fragment ions 10plex TMT were included in the quantification settings, and enzyme specificity was set to trypsin with a maximum of two missed cleavages. Cys carbamidomethylation (+57.021 Da) was considered a fixed modification, and Met oxidation (+15.994 Da), deamidation of Asn (N) and Gln (Q) (+0.984 Da), and protein N-terminal acetylation (+42.010 Da) were selected as variable modifications. Five variable modifications per peptide were allowed. All identifications were filtered to achieve 1% peptide spectral match and 1% protein false discovery rate. The quantitation of the identified proteins was determined by reporter ion intensities using at least one razor/unique peptide (supplemental Table SB). The individual protein abundances were normalized across experimental sets by dividing each protein reporter ion intensity by the reference reporter intensity within that specific experiment. The data were further normalized by the total ratio intensity of each channel to correct for any variation in total yield during the labeling reactions.

Peptides identified as potential contaminants or appearing in the reverse database were excluded for the glycosylation site analysis. Only peptides containing deamidated Asn within conserved *N*-glycosylation motifs, *i.e.*, NxS/T/C, where *x* ≠ P, were considered. The de-*N*-glycopeptides were normalized by the reference channel and further normalized by the total ratio intensity of each channel.

### Bioinformatics

Bioinformatic analyses were performed using Perseus, version 1.5.4.1, available within MaxQuant.

The Human Tissue and Blood Atlas, both part of the Human Protein Atlas (43, 44, 45), were used to annotate the cellular origin of the identified proteins. Sets of elevated proteins within relevant tissues (which included tissue enriched, group enriched, and tissue enhanced) including in the bone marrow (total of 534 proteins) and the prostatic tissue (total of 120 proteins) were extracted from the Tissue Atlas. The list of ECM proteins was obtained from the Human Blood Atlas, under the section the human secretome and category secreted to ECM (total of 234 proteins).

### Experimental Design and Statistical Rationale

In total, 55 biological replicates from BPH (*n* = 5) and PCa patients (G1–G5, *n* = 10/grade) were investigated. No technical replicates were acquired. The BPH served as a clinically relevant reference. For glycome profiling and glycosylation site analysis, statistical significance was assessed using unpaired two-tailed Student's *t* tests with *p* < 0.05 as the confidence threshold. For glycoproteome profiling, the statistical significance of the summed intensities of the glycopeptides grouped based on glycan classes or tissue origin was assessed using unpaired two-tailed Student's *t* tests with *p* < 0.05 as the confidence threshold. Correlation analysis was carried out using Pearson correlation coefficient and tested for significance using *t* distribution tests with *p* < 0.05 as the confidence threshold.

## Results and Discussion

### Integrated Glycomics and Glycoproteomics Profiling of PCa and BPH Tissues

**Fig. 1:**
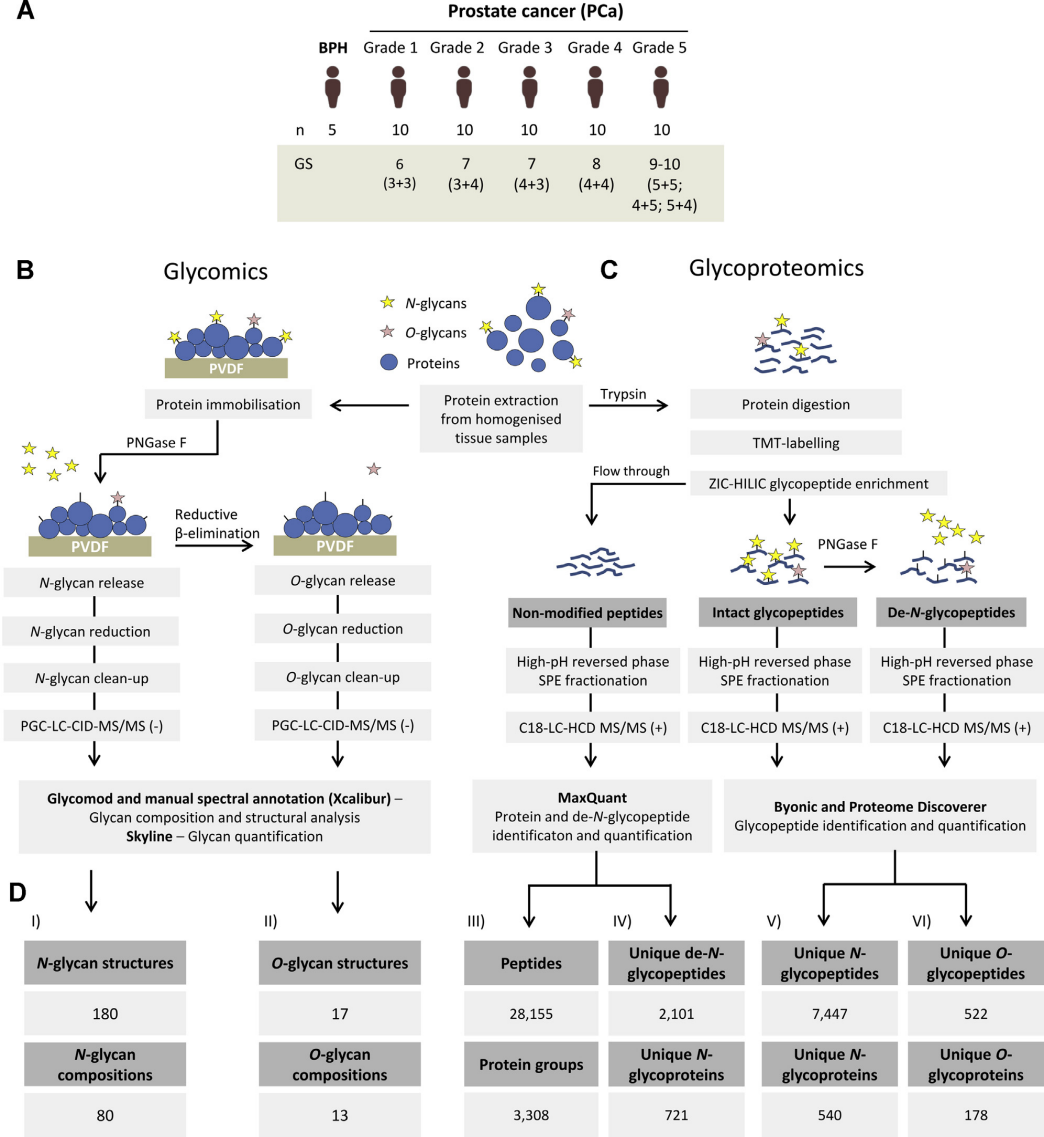
**Overview of patient cohort and experimental workflows used to investigate the tissue glycoproteome during prostate cancer (PCa) progression.***A*, overview of the patient cohort included in this study including BPH patients (*n* = 5) and PCa patients suffering from five different grades of PCa (G1–G5) as classified by Gleason score annotation (Gleason score 6–10). The applied (*B*) glycomics and (*C*) glycoproteomics experimental approaches. *D*, the PCa tissue glycoproteome coverage. Identifications made across all samples have been stated for (*I*) *N*-glycans and (*II*) *O*-glycan structures (isomers) and compositions, (*III*) unique peptides and corresponding proteins identified in the nonmodified fraction, (*IV*) unique glycosylation sites (as measured by de-*N*-glycopeptides) and corresponding *N*-glycoproteins, (*V*) unique intact *N*-glycopeptides and *N*-glycoproteins identified after ZIC-HILIC enrichment, and (*VI*) unique intact *O*-glycopeptides and source *O*-glycoproteins identified after ZIC-HILIC enrichment and de-*N*-glycosylation. BPH, benign prostatic hyperplasia; HCD, higher-energy collision-induced dissociation; PGC, porous graphitized carbon; PNGase, peptide:*N*-glycosidase F; PVDF, polyvinylidene fluoride; TMT, tandem mass tag; ZIC-HILIC, zwitterionic hydrophilic interaction liquid chromatography.

**Fig. 2:**
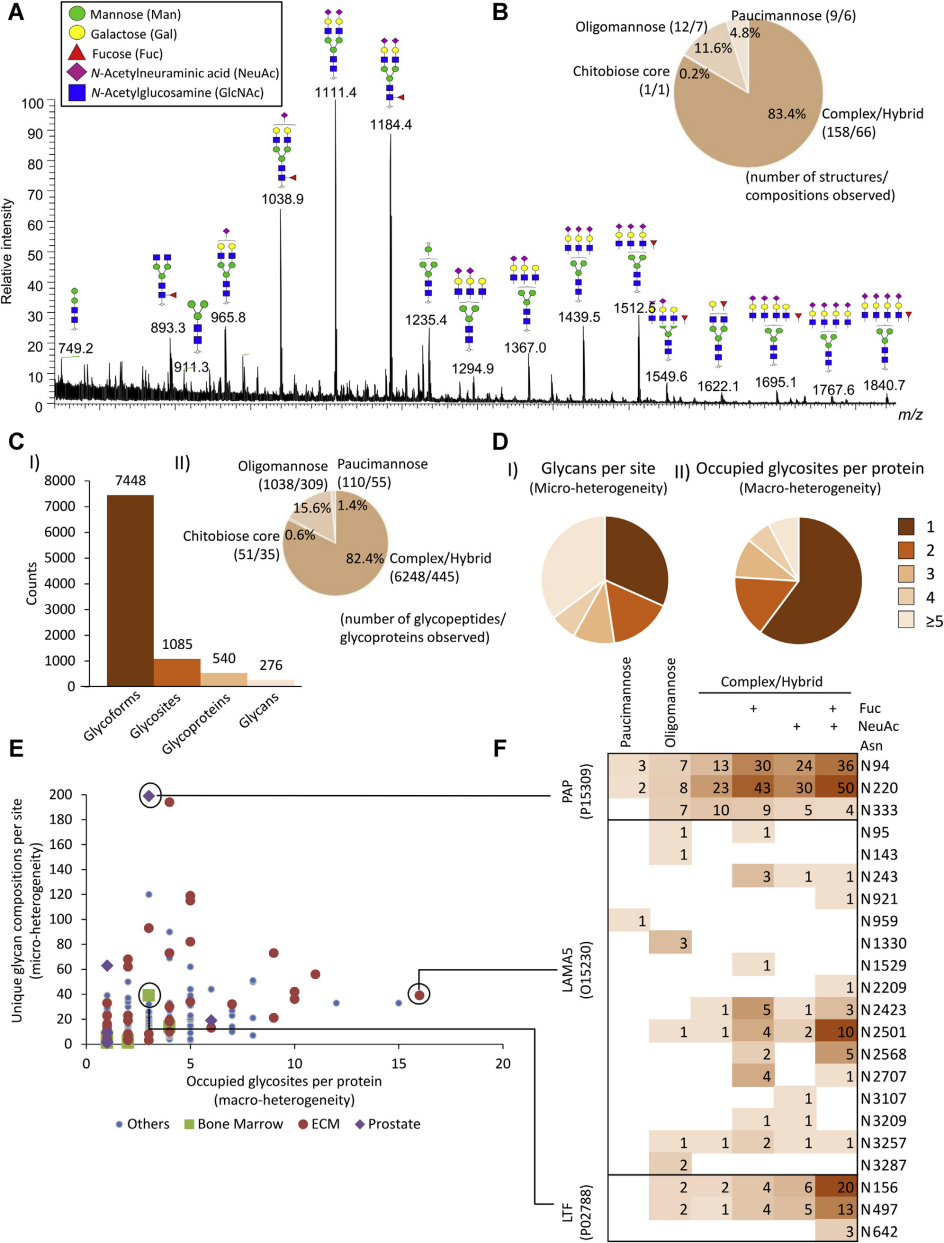
**The prostate cancer (PCa) tissue *N*-glycome and *N*-glycoproteome.***A*, summed MS spectrum (10–68 min) from a representative LC–MS/MS *N*-glycan profile of PCa tissue (G1). *B*, *N*-glycan class distribution based on the identified glycans across the investigated PCa grades and BPH (average of the relative abundance from all samples). *C*, (*I*) overview of the *N*-glycoproteome of PCa tissue including the identified unique *N*-glycoforms (unique protein + unique site + unique glycan), *N*-glycosites, *N*-glycoproteins, and *N*-glycans identified in ZIC-HILIC-enriched fractions. (*II*) *N*-glycan class distribution based on identified intact glycopeptides. *D*, microheterogeneity (*I*) and macroheterogeneity (*II*) of protein *N*-glycosylation based on the glycans (unique) observed at each site and the occupied glycosites per protein. *E*, glycan heterogeneity by protein. The cell source of the identified proteins has been annotated according to the Protein Atlas, see *key* for color coding. *F*, extensive microheterogeneity and macroheterogeneity of selected glycoproteins from different cell origins including the prostate tissue–derived prostatic acid phosphatase (PAP), ECM-derived laminin subunit alpha-5 (LAMA5), and bone marrow–derived lactotransferrin (LTF) in the PCa tumor microenvironment. ECM, extracellular matrix.

In this study, the protein *N*- and *O*-glycosylation were investigated from fresh prostate tissue collected after radical prostatectomy from age-matched cohorts of 50 PCa patients spanning five grades of the disease (G1–G5, *n* = 10/grade) and a group of BPH patients (*n* = 5) included as a clinical reference (Fig. 1*A*). Multiple analytical approaches were used to quantitatively profile: (1) the *N*- and *O*-glycan fine structures, including their composition, branching, and linkage isomers using PGC–LC–MS/MS–based glycomics of released *N*- and *O*-glycans (Fig. 1*B*); (2) the protein carriers, *N*- and *O*-glycosylation sites, and their microheterogeneity obtained using label-assisted glycoproteomics of intact *N*- and *O*-glycopeptides; and (3) the *N*-glycan occupancy at each site (macroheterogeneity) using proteomics profiling of formerly *N*-glycosylated peptides (hereafter referred to as de-*N*-glycopeptides) adjusted by the protein level (Fig. 1*C*).

In short, glycomics identified 180 *N*-glycans spanning 80 unique compositions and 17 *O*-glycans spanning 13 *O*-glycan compositions (supplemental Tables S1 and S2 and supplemental Datas S1 and S2), glycoproteomics identified 7447 unique intact *N*-glycopeptides (herein defined as a unique glycan composition at a unique site on a unique glycoprotein) and 522 unique intact *O*-glycopeptides covering 540 *N*-glycoproteins and 178 *O*-glycoproteins, respectively (supplemental Tables S3 and S4). Furthermore, site occupancy analysis established the occupancy of 2101 *N*-glycosylation sites covering 721 *N*-glycoproteins and the relative protein level of 3308 proteins (supplemental Tables S5 and S6) across all samples (Fig. 1*D*).

### Heterogeneous Cell- and Site-Specific *N*-Glycosylation in PCa and BPH Tissues

The *N*-glycome of the investigated tissue samples was found to comprise mainly complex-type biantennary and triantennary sialoglycans with and without fucosylation (83.4%) and a lower amount of oligomannosidic-type *N*-glycans (11.6%) and paucimannosidic-type *N*-glycans (4.8%, average relative abundance of all replicates) (Fig. 2, *A* and *B*). LC separation of the α2,6- and α2,3-linked sialoglycans was achieved, and MS/MS signatures confirmed the presence of core fucosylation, bisecting GlcNAc, LacdiNAc, and NeuGc and other important glycan fine structural features (supplemental Fig. S1, supplemental Table S1, and supplemental Datas S1 and S2).

The glycoproteomics data enabled deep glycoproteome coverage comparable to recent glycoproteomics studies (23, 24, 25). In total, 1223 unique glycopeptides, corresponding to 16.4% of the data set, were observed across all 54 samples (supplemental Fig. S2A), and 6887 unique glycopeptides (92.5% of the data set) were detected across all patient groups (supplemental Fig. S2B). Importantly, quantitative glycoproteome profiling achieved by TMT labeling and detection of intact glycopeptides is getting more common in glycoproteomics as elegantly demonstrated in a recently published study tracking the changes in protein *N*-glycosylation during mouse myogenesis and muscle development (46). Key advantages of TMT-based quantitative glycoproteomics include enhanced reproducibility, quantitative accuracy, sensitivity, and sample throughput enabled by the ability to multiplex the glycopeptide analyses relevant when dealing with larger sample cohorts (47). Complex/hybrid, oligomannose, paucimannose, and chitobiose core glycopeptides covered 82.4%, 15.6%, 1.4%, and 0.6% of the glycoproteome, respectively (Fig. 2*C*). The glycoproteomics and glycomics data showed excellent agreement as demonstrated by the high correlation (*r* = 0.827) of the glycans detected by the two data sets (data not shown). Moreover, 70% of all identified intact glycopeptides carried glycans observed in the *N*-glycome profile. Nearly 70% of the glycosites carried more than one glycan (average of 6.8 glycan/site; Fig. 2*D*, *panel I*), whereas 60% of the proteins were observed with only one occupied glycosite (Fig. 2*D*, *panel II* and supplemental Table S3).

As expected, the identified tissue glycoproteome was found to comprise proteins expressed by both the PCa cells and the tumor stroma including fibroblasts, immune, and endothelial cells and proteins present in the surrounding ECM as inferred based on well-annotated tissue expression in the Protein Atlas (43, 44, 45). To explore the tissue-specific expression of the PCa glycoproteome, the identified glycoproteins were grouped into three crude cellular origins including bone marrow (immune cells), ECM, and prostate aiming to cover the main tissue sources contributing to the complex PCa tumor microenvironment. The bone marrow glycoproteins comprised granulocytic markers of neutrophil and macrophage origins, including myeloperoxidase (P05164), lactotransferrin (LTF; P02788), cathepsin G (P08311), and neutrophil elastase (P08246) (48, 49, 50), whereas prominent prostate-specific glycoproteins were PSA (P07288), prostatic acid phosphatase (PAP; P15309), and glutamate carboxypeptidase 2 (Q04609) (51). Finally, many ECM glycoproteins were identified including various laminin isoforms, *e.g.*, laminin subunit α-5 (LAMA5; O15230), collagen as well as agrin (O00468), tenascin (P24821), and basement membrane–specific heparan sulfate proteoglycan core protein (P98160) that reportedly play important roles in PCa development and metastasis (52, 53) (supplemental Table S3).

Extensive microheterogeneity and macroheterogeneity of the identified glycoproteins arising from the various cellular origins were observed (Fig. 2*E*). The ECM glycoproteins were particularly heterogenous displaying more than two glycans per site and often more than five occupied glycosites per protein. The proteins from prostate and bone marrow origin carried fewer glycosites per protein but displayed high microheterogeneity. The data allowed for a detailed assessment of the site-specific microheterogeneity and macroheterogeneity of the identified glycoproteins with tissue information as demonstrated by analysis of key glycoproteins from the three tissue origins (Fig. 2*F*). The prostate-specific PAP carried few occupied glycosites (N94, N220, and N333) that displayed high microheterogeneity of all three sites. Conversely, the ECM glycoprotein LAMA5 carried 16 occupied glycosites displaying limited heterogeneity; *e.g.*, N143, N959, N1330, N1529, N2209, and N3107 carried only a single glycan type. Finally, the bone marrow–derived LTF contained three occupied sites, two of them with high glycan heterogeneity (N156 and N497) and one with low heterogeneity (N642) (supplemental Table S3).

We then compared our site-specific glycoprofiling data of PAP, LAMA5, and LTF to the literature. In agreement with our data, N94, N220, and N333 were previously found to be utilized glycosylation sites of seminal fluid PAP (54). We identified 199 *N*-glycans of PAP adding significantly to the 21 *N*-glycans previously reported from that protein. Similarly, N156, N497, and N642 were previously reported glycosylation sites of milk LTF (55). In line with our data, complex-type *N*-glycans were found to decorate LTF; Yu *et al.* (56) and Parc *et al.* (57) showed 32 and 18 unique glycan compositions, respectively, from human LTF. Of these, 13 overlapped with the 39 glycan compositions identified of LTF in this study. Finally, UniProtKB lists 23 potential *N*-glycosylation sites for LAMA5, of which in our glycoproteome data confirmed 16 sites. Several site mapping studies have previously evidenced glycosylation at N95, N2209, N2303, N2423, N2501, N2568, N2707, and N3107 in LAMA5 by LC–MS/MS (58, 59, 60), but no details of the *N*-glycans carried by this protein are available in the literature.

### Dynamics of the *N*-Glycoproteome During PCa Progression

**Fig. 3:**
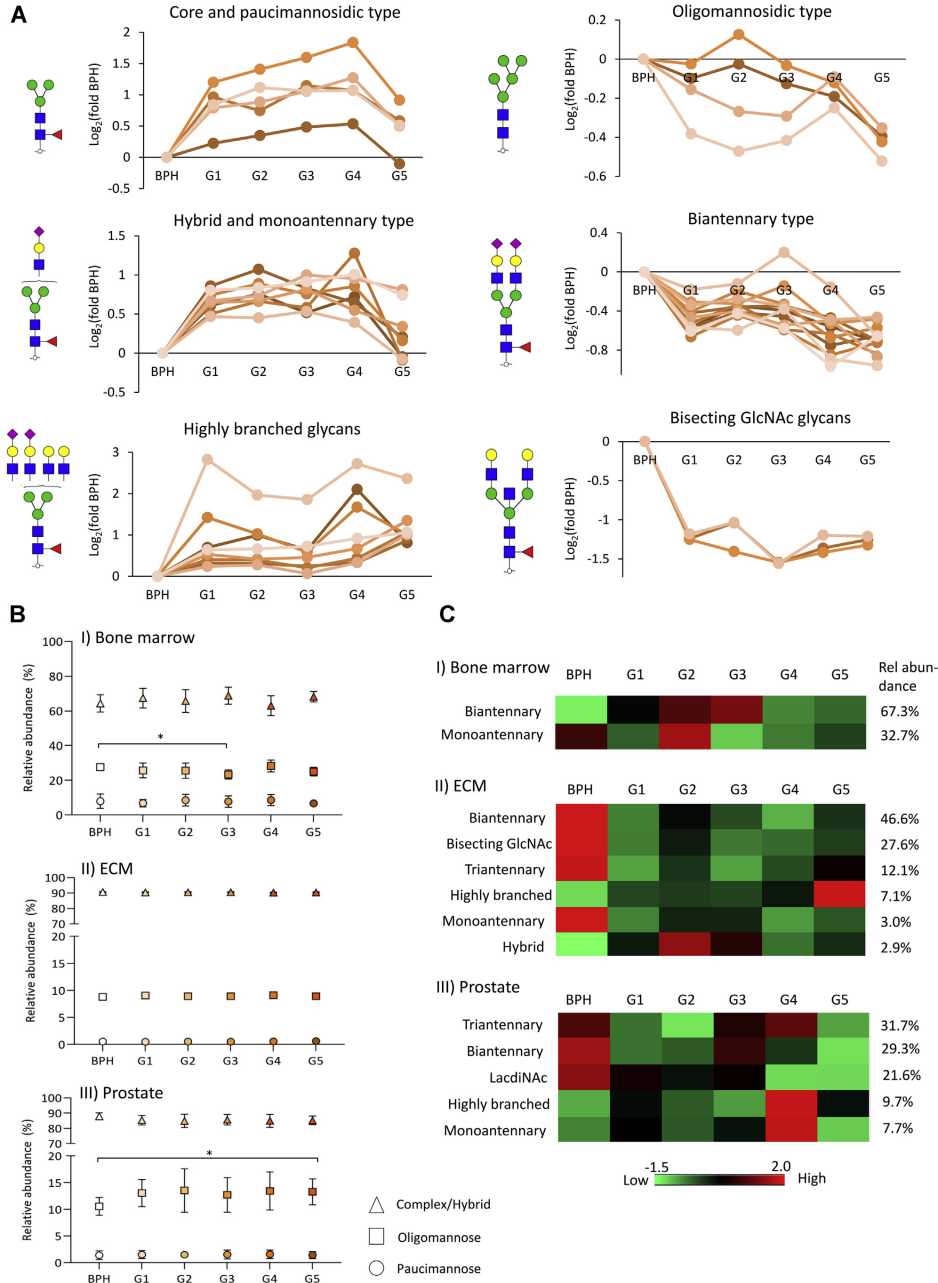
***N*-glycosylation remodeling associated with prostate cancer (PCa) progression**. *A*, depiction of the *N*-glycome alteration associated with PCa progression (PCa G1–G5 *versus* BPH, Student's *t* test, *p* < 0.05, supplemental Table S1). Graphs are based on the Log2-fold change of the relative abundance between each PCa grade and BPH. The individual glycans were grouped according to their glycan type and other structural features. The most abundant glycan structure from each class is depicted next to each graph. *B*, weakly altered tissue-specific glycosylation across PCa progression as determined by intact glycopeptide profiling. Decreased oligomannosylation was observed in the bone marrow–derived glycoproteins from PCa G3 compared with BPH, whereas increased oligomannosylation was observed in prostate-derived glycoproteins from PCa G5 compared with BPH (Student's *t* test, *p* < 0.05). *C*, distribution of intact glycopeptides in PCa and BPH grouped by cellular origin and glycan feature that showed significant correlation with the glycome data set (Pearson, *p* < 0.05) (supplemental Table S7). The heat map shows the average of the summed relative abundance of intact glycopeptides from all replicates (*n* = 5 BPH; *n* = 10/G1–G4, and *n* = 9/G5). BPH, benign prostatic hyperplasia, ECM, extracellular matrix.

The protein *N*-glycosylation dynamics during PCa progression was investigated using three levels of analysis. First, quantitative *N*-glycan distribution analysis revealed that many *N*-glycans are differentially expressed across the PCa grades relative to BPH (*t* test, *p* < 0.05) (Fig. 3*A*). Low-grade PCa tissues (G1–G4) were dominated by a high expression of paucimannosidic- and monoantennary complex-type *N*-glycans relative to BPH tissues, whereas high-grade PCa tissues (G5) were dominated by highly branched complex-type *N*-glycans. Oligomannosidic-, hybrid-, and biantennary complex-type *N*-glycans, some of which carried LacdiNAc features, showed a lower expression in G5 relative to BPH tissues. Bisecting GlcNAc-containing glycans were relatively lowly expressed in PCa tissues regardless of grade, whereas the α2,6- or α2,3-sialylation and core fucosylation were uniformly expressed glycosignatures across the examined tissue samples (supplemental Table S1).

Previous studies have reported that the *N*-acetylglucosaminyltransferase (GnT)-III, which catalyses bisecting GlcNAcylation, is associated with tumor suppression by inhibiting growth factor signaling, tumor development, and migration (61, 62, 63). The antennary branching of *N*-glycans is catalyzed by GnT-IV and GnT-V (64). Lange *et al.* (65) reported that the GnT-V–mediated glycan branching is elevated in metastatic PCa and is a potential marker for poor PCa prognosis. Gao *et al.* (66) reported higher mRNA levels and activity of GnT-V in PCa cell lines compared with nontumorigenic prostatic cells. Elevation of triantennary and tetra-antennary *N*-glycan in serum was also shown to be a signature associated with castration-resistant PCa patients (67).

*N*-glycolylneuraminic acid (Neu5Gc)-containing *N*-glycans were also identified in our analysis of prostate tissue as evidenced by LC–MS/MS (supplemental Data S1). Most notably, a significant lower abundance of the α2,6-linked monovalent and divalent Neu5Gc-containing biantennary core–fucosylated glycans were identified in PCa G4 and G5 compared with BPH (supplemental Table S1). Humans are deficient in the hydroxylase that converts CMP–Neu5Ac to CMP-Neu5Gc, but Neu5Gc can be metabolically incorporated into human tissues *via* dietary intake (*e.g.*, red meat) (68). Neu5Gc has previously been associated with inflammation and risk of cancer (69, 70), but little is known about its role in PCa.

Next, we profiled the distribution of intact glycopeptides by their glycan class and inferred tissue origins across the PCa grades and BPH (Fig. 3*B*). The bone marrow glycoproteins showed significant expression of oligomannosidic-type *N*-glycans (average of 26%) and paucimannosidic-type *N*-glycans (7.8%) compared with the ECM/prostate glycoproteins (9.0%/12.8% and 0.6%/1.5% for oligomannose and paucimannose, respectively). In line with these findings, paucimannosidic- and oligomannosidic-type *N*-glycans are known features of neutrophils and macrophages (49, 50). Examples of paucimannosidic- and oligomannosidic-rich neutrophil and macrophage glycoproteins, such as myeloperoxidase, neutrophil elastase, and cathepsin G (71, 72, 73), were identified in the glycoproteome data set. Modest cellular origin–specific alterations in the glycan class distribution during PCa progression were observed. The bone marrow–derived glycoproteins showed a slight but significant reduction in oligomannosylation at PCa (G3) (BPH: 27.6% *versus* G3: 23.4%, *t* test *p* < 0.05), whereas the prostate-derived glycoproteins carried elevated oligomannosylation in advanced PCa tissues (G5) (BPH: 10.6% *versus* G5: 13.3%, *t* test *p* < 0.05) (supplemental Table S3).

Supporting the prostate-specific elevation of oligomannose observed in this study, spatial distribution analysis of detached glycans in cancer tissues using MALDI–MS imaging previously demonstrated that oligomannosidic-type glycans are primarily expressed in the tumor region of late stage of ovarian cancer (74) and PCa (75, 76). Furthermore, enhanced oligomannosylation of serum glycoproteins in advanced PCa detected by antioligomannose antibodies was reported (77). Finally, downregulation of the Golgi α-mannosidase I responsible for processing of oligomannose glycans was associated with metastatic cholangiocarcinoma and increased migratory and invasive capabilities (78).

Correlation analysis between the *N*-glycome and *N*-glycoproteome data sets was then carried out to infer the glycan fine structural features decorating each glycosylation site. The intact glycopeptides carrying complex-type glycan compositions showed significant correlation with the matched glycan structures from the glycome data set (supplemental Table S7). Correlations were found for several structural features including glycan types, number of antennas, and the presence of bisecting GlcNAcylation and LacdiNAc motifs. We observed that bisecting GlcNAc is an enriched signature of glycans carried by the ECM glycoproteins, whereas prostate-derived glycoproteins are enriched in LacdiNAc glycans (Fig. 3*C*). Supporting these findings, previous studies reported that LacdiNAc glycans decorate prostate-specific glycoproteins such as PSA (79, 80) and that bisecting GlcNAc is a glycan feature of ECM glycoproteins (81, 82, 83).

Recapitulating the downregulation of bisecting GlcNAc and LacdiNAc *N*-glycans in PCa relative to BPH, bisecting GlcNAc- and LacdiNAc-containing glycopeptides of ECM and prostate-derived glycoproteins, respectively, showed decreased abundances in PCa. Furthermore, elevation of highly branched glycans decorating ECM-derived glycoproteins was observed in advanced PCa (G5), recapitulating the grade-specific changes observed in the glycome data set.

### Altered Expression of Oligosaccharyltransferase and *N*-Glycan Site Occupancy During PCa Progression

**Fig. 4:**
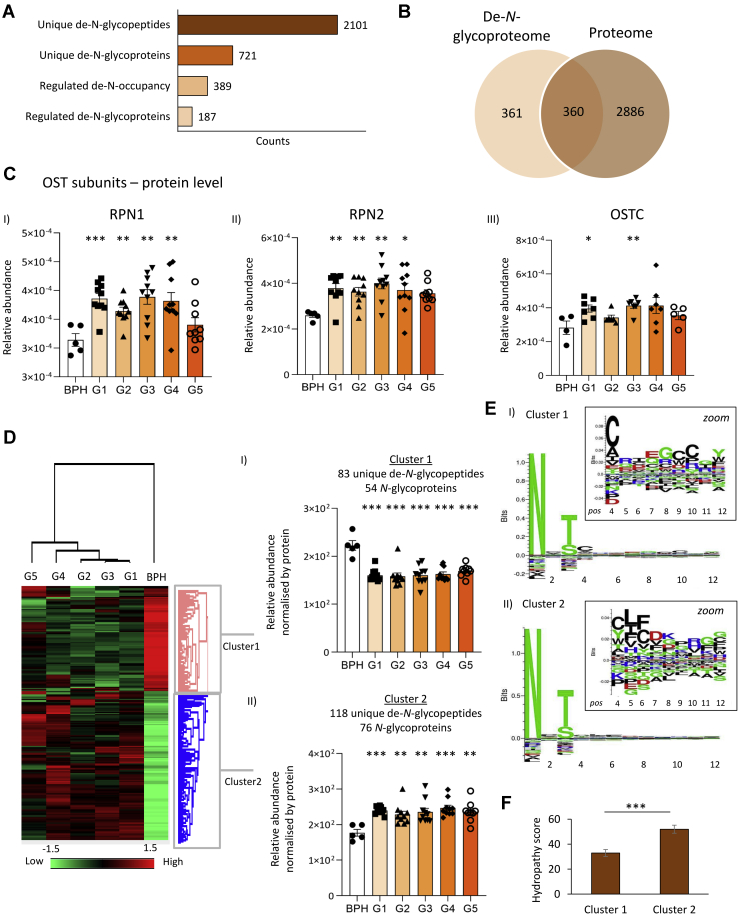
**Site occupancy analysis of the prostate cancer (PCa) tissue *N*-glycoproteome**. *A*, identified de-*N*-glycopeptides and the corresponding *N*-glycoproteins (both unique) and sites showing altered occupancy across the five PCa grades (G1–G5) and regulated glycoproteins (Student's *t* test, BHP *versus* G1–G5, *p* < 0.05). *B*, overlap between proteins identified in the de-*N*-glycoproteome and the entire proteome from all studied samples. *C*, enhanced expression of RPN1 (*i*), RPN2 (*ii*), and OSTC (iii) forming subunits of the OST complex in PCa compared with BPH (Student's *t* test, ∗*p* < 0.05, ∗∗*p* < 0.01, ∗∗∗*p* < 0.001). Other known OST subunits (*e.g.*, STT3A/B and DAD) were identified below the limit of quantitation. *D*, Euclidean clustering analysis of sites consistently identified with altered *N*-glycan occupancy in PCa. The heat map plots the average site occupancy (adjusted for protein level) that were identified without missing values (*n* = 5 BPH; *n* = 10/G1–G4 and *n* = 9/G5). Two major clusters were identified: (*i*) cluster 1 sites showed a reduced occupancy, whereas (*ii*) cluster 2 sites showed a higher site occupancy in G1–G5 relative to BPH (Student's *t* test, ∗*p* < 0.05, ∗∗*p* < 0.01, ∗∗∗*p* < 0.001). *E*, sequence motif analysis of cluster 1 and cluster 2 sites. Residues immediately C terminal to the *N*-glycosylation site were explored for enriched motifs. Sequence logos were probability weighted (Kullback–Leiblerhe divergence). The height of the amino acid residue corresponds to their probability times their log-odd scores. *F*, hydropathy scores of cluster 1 and cluster 2 sites. BPH, benign prostatic hyperplasia; OST, oligosaccharyltransferase; OSTC, oligosaccharyltransferase complex.

Aiming to also map the dynamics of the glycosylation sites and their occupancy levels in PCa progression, we identified 2101 de-*N*-glycopeptides from 721 *N*-glycoproteins following peptide:*N*-glycosidase F digestion (Fig. 4*A*). In total, 360 of the formerly *N*-glycosylated proteins were also identified in the proteome data set (Fig. 4*B*), which was used to normalize the de-*N*-glycopeptide level and determine the site occupancy. The *N*-glycan site occupancy was compared across PCa grades and BPH, and a total of 389 de-*N*-glycopeptides belonging to 187 *N*-glycoproteins showed altered occupancy (supplemental Table S5).

The utilization of glycosylation sites relates to the first step of the biosynthesis of *N*-glycoproteins involving the transfer of a lipid-linked *N*-glycan precursor (Glc3Man9GlnNAc2) to nascent polypeptides by the large oligosaccharyltransferase (OST) complex (84). The proteome data revealed that two OST subunits, RPN1 (P04843) and RPN2 (P04844), were significantly elevated in PCa grades (G1–G4) providing a molecular basis for the increased site occupancy in PCa (Fig. 4*C*). In support, another key OST subunit, OSTC (Q9NRP0), was also found to be overexpressed in PCa grades G1 and G3. The catalytic OST subunits, STT3A (P46977), STT3B (Q8TCJ2), and DAD1 (P61803), were detected below the level of quantitation (supplemental Table S6).

Clustering analysis was performed using the set of de-*N*-glycopeptides that showed altered site occupancy and was consistently identified in all replicates (Fig. 4*D*). Two main clusters were observed: cluster 1 grouped peptides containing less occupied glycosylation sites in PCa relative to BPH, whereas cluster 2 comprised peptides with elevated site occupancy. Motif sequence analysis of cluster 1 and cluster 2 de-*N*-glycopeptides was performed to explore the OST substrate specificity in light of the occupancy data using the peptide region immediately C-terminal to the N-glycosylation site. Cluster 1 de-*N*-glycopeptides showed an enrichment of hydrophilic amino acids, such as cysteine (C), arginine (R), and glutamic acid (E), whereas hydrophobic amino acids, such as leucine (L) and phenylalanine (F), were over-represented in cluster 2 de-*N*-glycopeptides. Accordingly, the hydropathy of the OST recognition region of the de-*N*-glycopeptides upregulated in PCa (cluster 2) was found to be significantly higher than the sites experiencing less occupancy in PCa (cluster 1) (Fig. 4*F*). Our data point to that the increased expression of relatively hydrophobic sequons and the elevation of OST subunits concertedly increase the site occupancy during PCa progression, which are findings that require further investigations.

Other mechanisms besides expression of OST subunits have been demonstrated to impact the *N*-glycosylation site occupancy. The OST substrate recognition and glycosylation transfer efficiency are modulated by the oxidoreductase activity of the yeast OST subunits Ost3p and Ost6p (85). Furthermore, the STT3B-OST-specific subunit TUC3, found to be downregulated in PCa tissues, is known to reduce the OST-mediated *N*-glycan transfer efficiency (86). In the same study, knockdown of TUC3 in PCa cell lines led to increased proliferation, invasion, and *in vivo* tumor growth, indicating that this subunit may act as a tumor suppressor in PCa. Finally, the expression of RPN2 was previously linked to other types of cancer, such as breast cancer, colorectal cancer, and lung cancer, but hitherto not to PCa (11).

### Altered Protein *O*-Glycosylation During PCa Progression

**Fig. 5:**
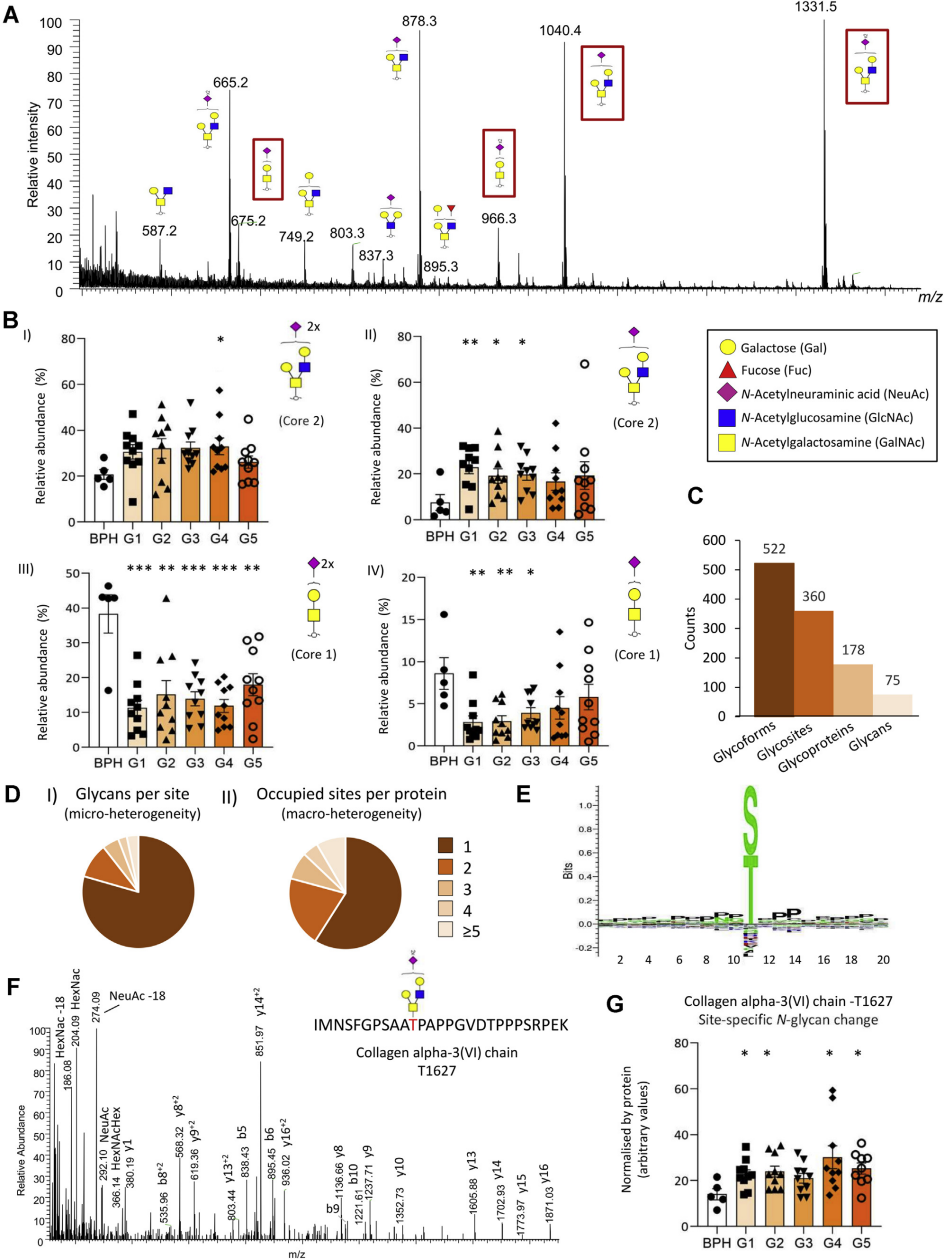
**Overview of the prostate cancer (PCa) tissue *O*-glycome and *O*-glycoproteome**. *A*, representative summed MS spectrum of released *O*-glycans from PCa tissue (G1) (*m*/*z* 500–1450, 5–30 min). The abundant core 1- and core 2-type *O*-glycans investigated for their relative distribution across the PCa grades and BPH in *B* are highlighted. *B*, the sialylated core 1- and core 2-type *O*-glycans showed differential expression between BPH (*n* = 5) and the five PCa grades, G1 to G5 (*n* = 10/grade) (Student's *t* test, *p* < 0.05, all data plotted as mean ± SD). *C*, overview of the *O*-glycoproteome of all investigated samples. *D*, microheterogeneity (*I*) and macroheterogeneity (*II*) of protein *O*-glycosylation based on the unique glycans observed at each site and occupied glycosites per protein. *E*, sequence motif analysis of 160 *O*-glycosites. *F*, annotated higher-energy collision-induced dissociation–MS/MS spectrum of an intact *O*-glycopeptide from collagen alpha-3(VI) chain (P12111) carrying the HexNAc(2)Hex(2)NeuAc(2) *O*-glycan at T1627. *G*, altered site-specific core 2-type *O*-glycosylation of collagen alpha-3(VI) chain (P12111) during PCa progression (see *F* for spectral evidence). BPH, benign prostatic hyperplasia.

The *O*-glycome profile of the PCa tissue revealed a total of 17 structures covering 13 compositions (supplemental Table S2). Core 1- and 2-type structures were prominent *O*-glycans across all investigated samples (Fig. 5*A*). Quantitative analysis showed that sialylated core 1 glycans were significantly reduced, whereas sialylated core 2 structures were elevated during PCa progression (Fig. 5*B*).

We identified a total of 522 unique *O*-glycopeptides from 360 *O*-glycosites covering 178 *O*-glycoproteins and 75 unique *O*-glycan compositions (Fig. 5*C*).

Relatively low microheterogeneity and macroheterogeneity of the *O*-glycoproteome data was observed (Fig. 5*D*). Most *O*-glycosites (∼80%) carried only a single glycan, and most *O*-glycoproteins (60%) were identified with a single utilized glycosylation site. Sequence motif analysis showed a position-unspecific enrichment of proline around the glycosite (Fig. 5*E*) in agreement with a previous report demonstrating enhanced *O*-glycosylation at Pro-rich sites (87).

Most *O*-glycosites were unambiguously identified (308 of 360; 85%), but our HCD–MS/MS data left, as expected, some *O*-glycosylation sites ambiguously determined (88, 89).

Correlation analysis between the *O*-glycome and *O*-glycoproteome showed that the glycans of 13 *O*-glycopeptides quantitatively correlated with 5 *O*-glycan structures (supplemental Table S8). For example, the *O*-glycopeptide from the high abundant glycoprotein collagen VI (P12111; supplemental Table S6) correlated with the abundant sialylated core 2 *O*-glycan. Moreover, this glycoform was confidently identified at T1627 in all 54 replicates (Fig. 5*F* and supplemental Tables S4 and S8). This glycoform recapitulated the changes in the *O*-glycome by displaying a PCa grade–specific elevation relative to BPH (Fig. 5*G*).

Core 2-branched *O*-glycans are synthesized by the core 2 β1,6-*N*-acetylglucosaminyltransferase (C2GNT; Q02742), which catalyses the transfer of *N*-acetylglucosamines onto mucin-type core 1 *O*-glycans. This protein was not identified in the proteomics data set. C2GNT expression reportedly is high in PCa cells as shown by immunohistochemistry of formalin-fixed paraffin-embedded tissues and correlates with PCa progression (90). It was also reported that high expression of C2GNT is associated with poor prognosis and higher risk of recurrence after radical prostatectomy (91). Finally, core 2 *O*-glycans were found to play important roles in the evasion of natural killer cell immunity, thus favoring PCa metastasis (92). Our data add to these observations by showing a site-specific elevation of core 2 *O*-glycosylation of collagen VI during PCa progression. The role of core 2 *O*-glycosylation of collagen VI in PCa progression remains to be studied.

## Conclusion

**Fig. 6:**
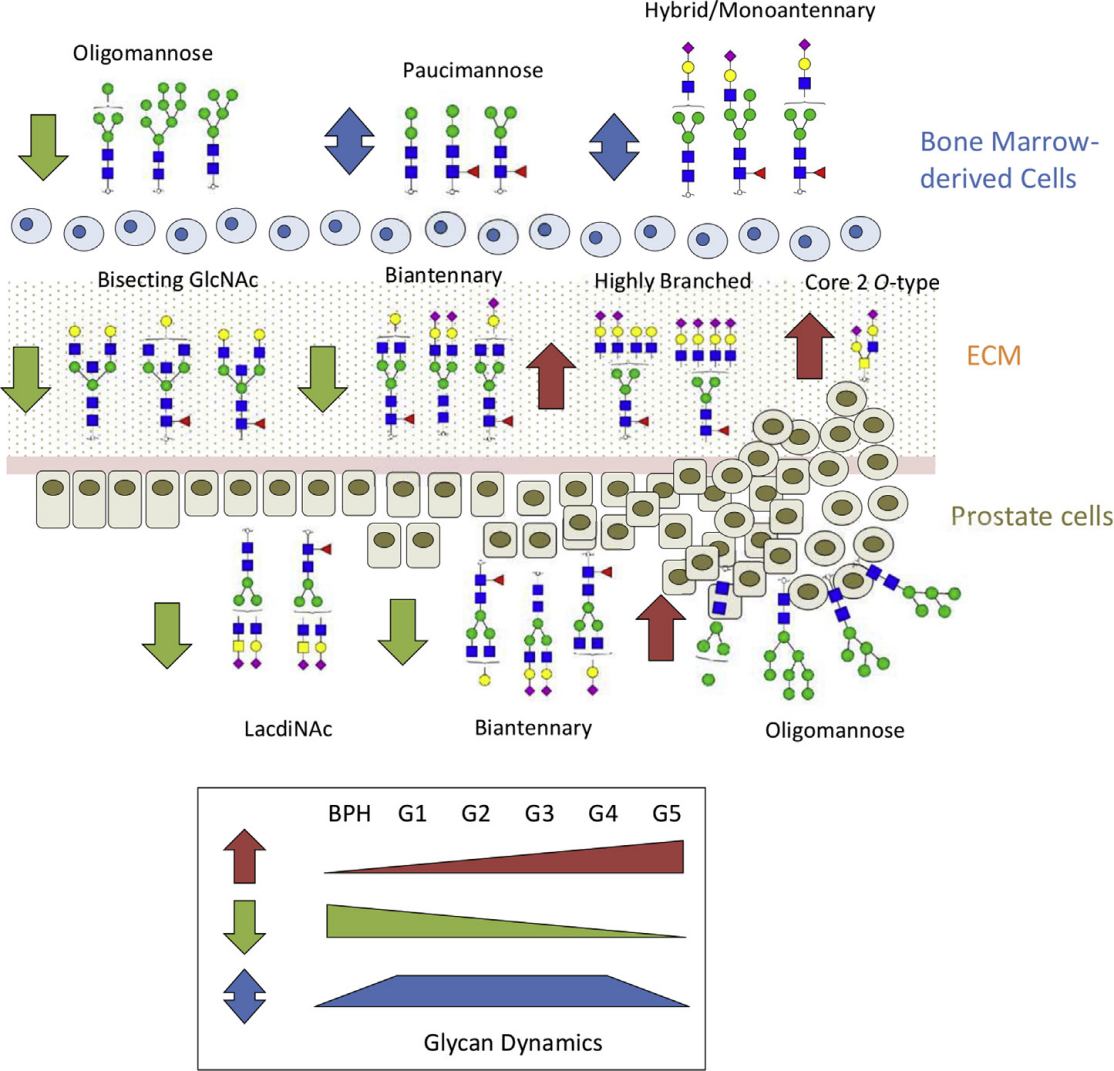
**Summary of the integrated glycomics and glycoproteomics findings showing the complexity and dynamics of the *N*- and *O*-glycosylation changes in the tumor microenvironment associated with prostate cancer progression.** BPH, benign prostatic hyperplasia; ECM, extracellular matrix.

We are the first to report on the complexity and dynamics of the tissue glycoproteome during PCa progression. Integrated use of MS-driven glycomics and glycoproteomics with valuable tissue annotation reference libraries enabled sensitive and quantitative insight into the cell-, protein-, and site-specific glycosylation across the PCa grades (Fig. 6). This study forms a valuable resource to explore how the dynamic remodeling of the glycoproteome functionally impacts PCa progression, thus, ultimately contributing to an improved molecular level understanding the disease mechanisms and fueling ongoing efforts to develop effective diagnostic and prognostic markers for improved patient outcome.

## Data Availability

All proteomics LC–MS/MS data files supporting the conclusions presented herein have been deposited to the ProteomeXchange Consortium *via* the PRIDE (93) partner repository with the data set identifiers PXD014271 and PXD019443. The PGC–LC–MS/MS *N*-glycome raw data files are available *via* the MassIVE Consortium (identifier: MSV000083727) under the project published by Chatterjee *et al.* (94).

## Conflict of interest

The authors declare no competing interests.

## Supporting Information

MCPRO_MCP/2020/002320_6.pdf

MCPRO_MCP/2020/002320_7.xlsx

MCPRO_MCP/2020/002320_8.pdf

## References

[1] Rawla P. (2019). Epidemiology of prostate cancer. World J. Oncol..

[2] Yadav S.S., Stockert J.A., Hackert V., Yadav K.K., Tewari A.K. (2018). Intratumor heterogeneity in prostate cancer. Urol. Oncol..

[3] Runa F., Hamalian S., Meade K., Shisgal P., Gray P.C., Kelber J.A. (2017). Tumor microenvironment heterogeneity: Challenges and opportunities. Curr. Mol. Biol. Rep..

[4] Offermann A., Hohensteiner S., Kuempers C., Ribbat-Idel J., Schneider F., Becker F., Hupe M.C., Duensing S., Merseburger A.S., Kirfel J., Reischl M., Lubczyk V., Kuefer R., Perner S. (2017). Prognostic value of the new prostate cancer International Society of Urological Pathology grade groups. Front. Med. (Lausanne).

[5] Descotes J.L. (2019). Diagnosis of prostate cancer. Asian J. Urol..

[6] Corcoran N.M., Hovens C.M., Hong M.K., Pedersen J., Casey R.G., Connolly S., Peters J., Harewood L., Gleave M.E., Goldenberg S.L., Costello A.J. (2012). Underestimation of Gleason score at prostate biopsy reflects sampling error in lower volume tumours. BJU Int..

[7] Tanase C.P., Codrici E., Popescu I.D., Mihai S., Enciu A.M., Necula L.G., Preda A., Ismail G., Albulescu R. (2017). Prostate cancer proteomics: Current trends and future perspectives for biomarker discovery. Oncotarget.

[8] Kawahara R., Recuero S., Nogueira F.C.S., Domont G.B., Leite K.R.M., Srougi M., Thaysen-Andersen M., Palmisano G. (2019). Tissue proteome signatures associated with five grades of prostate cancer and benign prostatic hyperplasia. Proteomics.

[9] Adhyam M., Gupta A.K. (2012). A review on the clinical utility of PSA in cancer prostate. Indian J. Surg. Oncol..

[10] Saini S. (2016). PSA and beyond: Alternative prostate cancer biomarkers. Cell Oncol. (Dordr.).

[11] Munkley J. (2017). Glycosylation is a global target for androgen control in prostate cancer cells. Endocr. Relat. Cancer.

[12] Munkley J., Mills I.G., Elliott D.J. (2016). The role of glycans in the development and progression of prostate cancer. Nat. Rev. Urol..

[13] Drake R.R., Jones E.E., Powers T.W., Nyalwidhe J.O. (2015). Altered glycosylation in prostate cancer. Adv. Cancer Res..

[14] Scott E., Munkley J. (2019). Glycans as biomarkers in prostate cancer. Int. J. Mol. Sci..

[15] Yoneyama T., Ohyama C., Hatakeyama S., Narita S., Habuchi T., Koie T., Mori K., Hidari K.I., Yamaguchi M., Suzuki T., Tobisawa Y. (2014). Measurement of aberrant glycosylation of prostate specific antigen can improve specificity in early detection of prostate cancer. Biochem. Biophys. Res. Commun..

[16] Mockl L. (2020). The emerging role of the mammalian glycocalyx in functional membrane organization and immune system regulation. Front. Cell Dev. Biol..

[17] Hudak J.E., Canham S.M., Bertozzi C.R. (2014). Glycocalyx engineering reveals a Siglec-based mechanism for NK cell immunoevasion. Nat. Chem. Biol..

[18] Pinho S.S., Reis C.A. (2015). Glycosylation in cancer: Mechanisms and clinical implications. Nat. Rev. Cancer.

[19] Chandler K.B., Costello C.E., Rahimi N. (2019). Glycosylation in the tumor microenvironment: Implications for tumor angiogenesis and metastasis. Cells.

[20] Thaysen-Andersen M., Packer N.H. (2014). Advances in LC-MS/MS-based glycoproteomics: Getting closer to system-wide site-specific mapping of the N- and O-glycoproteome. Biochim. Biophys. Acta.

[21] Thaysen-Andersen M., Packer N.H., Schulz B.L. (2016). Maturing glycoproteomics technologies provide unique structural insights into the N-glycoproteome and its regulation in health and disease. Mol. Cell. Proteomics.

[22] Parker B.L., Thaysen-Andersen M., Solis N., Scott N.E., Larsen M.R., Graham M.E., Packer N.H., Cordwell S.J. (2013). Site-specific glycan-peptide analysis for determination of N-glycoproteome heterogeneity. J. Proteome Res..

[23] Riley N.M., Hebert A.S., Westphall M.S., Coon J.J. (2019). Capturing site-specific heterogeneity with large-scale N-glycoproteome analysis. Nat. Commun..

[24] Leung K.K., Wilson G.M., Kirkemo L.L., Riley N.M., Coon J.J., Wells J.A. (2020). Broad and thematic remodeling of the surfaceome and glycoproteome on isogenic cells transformed with driving proliferative oncogenes. Proc. Natl. Acad. Sci. U. S. A..

[25] Fang P., Xie J., Sang S., Zhang L., Liu M., Yang L., Xu Y., Yan G., Yao J., Gao X., Qian W., Wang Z., Zhang Y., Yang P., Shen H. (2020). Multilayered N-glycoproteome profiling reveals highly heterogeneous and dysregulated protein N-glycosylation related to Alzheimer's disease. Anal. Chem..

[26] Saldova R., Fan Y., Fitzpatrick J.M., Watson R.W., Rudd P.M. (2011). Core fucosylation and alpha2-3 sialylation in serum N-glycome is significantly increased in prostate cancer comparing to benign prostate hyperplasia. Glycobiology.

[27] Ishibashi Y., Tobisawa Y., Hatakeyama S., Ohashi T., Tanaka M., Narita S., Koie T., Habuchi T., Nishimura S., Ohyama C., Yoneyama T. (2014). Serum tri- and tetra-antennary N-glycan is a potential predictive biomarker for castration-resistant prostate cancer. Prostate.

[28] Yang S., Clark D., Liu Y., Li S., Zhang H. (2017). High-throughput analysis of N-glycans using AutoTip via glycoprotein immobilization. Sci. Rep..

[29] Liu Y., Chen J., Sethi A., Li Q.K., Chen L., Collins B., Gillet L.C., Wollscheid B., Zhang H., Aebersold R. (2014). Glycoproteomic analysis of prostate cancer tissues by SWATH mass spectrometry discovers N-acylethanolamine acid amidase and protein tyrosine kinase 7 as signatures for tumor aggressiveness. Mol. Cell. Proteomics.

[30] Cima I., Schiess R., Wild P., Kaelin M., Schuffler P., Lange V., Picotti P., Ossola R., Templeton A., Schubert O., Fuchs T., Leippold T., Wyler S., Zehetner J., Jochum W. (2011). Cancer genetics-guided discovery of serum biomarker signatures for diagnosis and prognosis of prostate cancer. Proc. Natl. Acad. Sci. U. S. A..

[31] Jia X., Chen J., Sun S., Yang W., Yang S., Shah P., Hoti N., Veltri B., Zhang H. (2016). Detection of aggressive prostate cancer associated glycoproteins in urine using glycoproteomics and mass spectrometry. Proteomics.

[32] Shah P., Wang X., Yang W., Toghi Eshghi S., Sun S., Hoti N., Chen L., Yang S., Pasay J., Rubin A., Zhang H. (2015). Integrated proteomic and glycoproteomic analyses of prostate cancer cells reveal glycoprotein alteration in protein abundance and glycosylation. Mol. Cell. Proteomics.

[33] Kawahara R., Ortega F., Rosa-Fernandes L., Guimaraes V., Quina D., Nahas W., Schwammle V., Srougi M., Leite K.R.M., Thaysen-Andersen M., Larsen M.R., Palmisano G. (2018). Distinct urinary glycoprotein signatures in prostate cancer patients. Oncotarget.

[34] Epstein J.I., Egevad L., Amin M.B., Delahunt B., Srigley J.R., Humphrey P.A., Grading C. (2016). The 2014 International Society of Urological Pathology (ISUP) consensus conference on Gleason grading of prostatic carcinoma: Definition of grading patterns and proposal for a new grading system. Am. J. Surg. Pathol..

[35] Jensen P.H., Karlsson N.G., Kolarich D., Packer N.H. (2012). Structural analysis of N- and O-glycans released from glycoproteins. Nat. Protoc..

[36] Ashwood C., Lin C.H., Thaysen-Andersen M., Packer N.H. (2018). Discrimination of isomers of released N- and O-glycans using diagnostic product ions in negative ion PGC-LC-ESI-MS/MS. J. Am. Soc. Mass Spectrom..

[37] Adams K.J., Pratt B., Bose N., Dubois L.G., St John-Williams L., Perrott K.M., Ky K., Kapahi P., Sharma V., MacCoss M.J., Moseley M.A., Colton C.A., MacLean B.X., Schilling B., Thompson J.W. (2020). Skyline for small molecules: A unifying software package for quantitative metabolomics. J. Proteome Res..

[38] Mysling S., Palmisano G., Hojrup P., Thaysen-Andersen M. (2010). Utilizing ion-pairing hydrophilic interaction chromatography solid phase extraction for efficient glycopeptide enrichment in glycoproteomics. Anal. Chem..

[39] Bern M., Kil Y.J., Becker C. (2012). Byonic: Advanced peptide and protein identification software. Curr. Protoc. Bioinformatics.

[40] Bern M.W., Kil Y.J. (2011). Two-dimensional target decoy strategy for shotgun proteomics. J. Proteome Res..

[41] Cox J., Mann M. (2008). MaxQuant enables high peptide identification rates, individualized p.p.b.-range mass accuracies and proteome-wide protein quantification. Nat. Biotechnol..

[42] Cox J., Neuhauser N., Michalski A., Scheltema R.A., Olsen J.V., Mann M. (2011). Andromeda: A peptide search engine integrated into the MaxQuant environment. J. Proteome Res..

[43] Thul P.J., Akesson L., Wiking M., Mahdessian D., Geladaki A., Ait Blal H., Alm T., Asplund A., Bjork L., Breckels L.M., Backstrom A., Danielsson F., Fagerberg L., Fall J., Gatto L. (2017). A subcellular map of the human proteome. Science.

[44] Uhlen M., Fagerberg L., Hallstrom B.M., Lindskog C., Oksvold P., Mardinoglu A., Sivertsson A., Kampf C., Sjostedt E., Asplund A., Olsson I., Edlund K., Lundberg E., Navani S., Szigyarto C.A. (2015). Proteomics. Tissue-based map of the human proteome. Science.

[45] Uhlen M., Oksvold P., Fagerberg L., Lundberg E., Jonasson K., Forsberg M., Zwahlen M., Kampf C., Wester K., Hober S., Wernerus H., Bjorling L., Ponten F. (2010). Towards a knowledge-based human protein atlas. Nat. Biotechnol..

[46] Blazev R., Ashwood C., Abrahams J.L., Chung L.H., Francis D., Yang P., Watt K.I., Qian H., Quaife-Ryan G.A., Hudson J.E., Gregorevic P., Thaysen-Andersen M., Parker B.L. (2020). Integrated glycoproteomics identifies a role of N-glycosylation and galectin-1 on myogenesis and muscle development. Mol. Cell. Proteomics.

[47] Delafield D.G., Li L. (2020). Recent advances in analytical approaches for glycan and glycopeptide quantitation. Mol. Cell. Proteomics.

[48] Rorvig S., Ostergaard O., Heegaard N.H., Borregaard N. (2013). Proteome profiling of human neutrophil granule subsets, secretory vesicles, and cell membrane: Correlation with transcriptome profiling of neutrophil precursors. J. Leukoc. Biol..

[49] Thaysen-Andersen M., Venkatakrishnan V., Loke I., Laurini C., Diestel S., Parker B.L., Packer N.H. (2015). Human neutrophils secrete bioactive paucimannosidic proteins from azurophilic granules into pathogen-infected sputum. J. Biol. Chem..

[50] Hinneburg H., Pedersen J.L., Bokil N.J., Pralow A., Schirmeister F., Kawahara R., Rapp E., Saunders B.M., Thaysen-Andersen M. (2020). High-resolution longitudinal N- and O-glycoprofiling of human monocyte-to- macrophage transition. Glycobiology.

[51] Iglesias-Gato D., Wikstrom P., Tyanova S., Lavallee C., Thysell E., Carlsson J., Hagglof C., Cox J., Andren O., Stattin P., Egevad L., Widmark A., Bjartell A., Collins C.C., Bergh A. (2016). The proteome of primary prostate cancer. Eur. Urol..

[52] Elgundi Z., Papanicolaou M., Major G., Cox T.R., Melrose J., Whitelock J.M., Farrugia B.L. (2019). Cancer metastasis: The role of the extracellular matrix and the heparan sulfate proteoglycan perlecan. Front. Oncol..

[53] Stewart D.A., Cooper C.R., Sikes R.A. (2004). Changes in extracellular matrix (ECM) and ECM-associated proteins in the metastatic progression of prostate cancer. Reprod. Biol. Endocrinol..

[54] White K.Y., Rodemich L., Nyalwidhe J.O., Comunale M.A., Clements M.A., Lance R.S., Schellhammer P.F., Mehta A.S., Semmes O.J., Drake R.R. (2009). Glycomic characterization of prostate-specific antigen and prostatic acid phosphatase in prostate cancer and benign disease seminal plasma fluids. J. Proteome Res..

[55] Zlatina K., Galuska S.P. (2020). The N-glycans of lactoferrin: More than just a sweet decoration. Biochem. Cell Biol..

[56] Yu T., Guo C., Wang J., Hao P., Sui S., Chen X., Zhang R., Wang P., Yu G., Zhang L., Dai Y., Li N. (2011). Comprehensive characterization of the site-specific N-glycosylation of wild-type and recombinant human lactoferrin expressed in the milk of transgenic cloned cattle. Glycobiology.

[57] Parc A.L., Karav S., Rouquie C., Maga E.A., Bunyatratchata A., Barile D. (2017). Characterization of recombinant human lactoferrin N-glycans expressed in the milk of transgenic cows. PLoS One.

[58] Chen R., Jiang X., Sun D., Han G., Wang F., Ye M., Wang L., Zou H. (2009). Glycoproteomics analysis of human liver tissue by combination of multiple enzyme digestion and hydrazide chemistry. J. Proteome Res..

[59] Deb B., Patel K., Sathe G., Kumar P. (2019). N-glycoproteomic profiling reveals alteration in extracellular matrix organization in non-type bladder carcinoma. J. Clin. Med..

[60] Melo-Braga M.N., Schulz M., Liu Q., Swistowski A., Palmisano G., Engholm-Keller K., Jakobsen L., Zeng X., Larsen M.R. (2014). Comprehensive quantitative comparison of the membrane proteome, phosphoproteome, and sialiome of human embryonic and neural stem cells. Mol. Cell. Proteomics.

[61] Song Y., Aglipay J.A., Bernstein J.D., Goswami S., Stanley P. (2010). The bisecting GlcNAc on N-glycans inhibits growth factor signaling and retards mammary tumor progression. Cancer Res..

[62] Yoshimura M., Nishikawa A., Ihara Y., Taniguchi S., Taniguchi N. (1995). Suppression of lung metastasis of B16 mouse melanoma by N-acetylglucosaminyltransferase III gene transfection. Proc. Natl. Acad. Sci. U. S. A..

[63] Lu J., Isaji T., Im S., Fukuda T., Kameyama A., Gu J. (2016). Expression of N-acetylglucosaminyltransferase III suppresses alpha2,3-sialylation, and its distinctive functions in cell migration are attributed to alpha2,6-sialylation levels. J. Biol. Chem..

[64] Kizuka Y., Taniguchi N. (2016). Enzymes for N-glycan branching and their genetic and nongenetic regulation in cancer. Biomolecules.

[65] Lange T., Ullrich S., Muller I., Nentwich M.F., Stubke K., Feldhaus S., Knies C., Hellwinkel O.J., Vessella R.L., Abramjuk C., Anders M., Schroder-Schwarz J., Schlomm T., Huland H., Sauter G. (2012). Human prostate cancer in a clinically relevant xenograft mouse model: Identification of beta(1,6)-branched oligosaccharides as a marker of tumor progression. Clin. Cancer Res..

[66] Gao Y., Chachadi V.B., Cheng P.W., Brockhausen I. (2012). Glycosylation potential of human prostate cancer cell lines. Glycoconj. J..

[67] Matsumoto T., Hatakeyama S., Yoneyama T., Tobisawa Y., Ishibashi Y., Yamamoto H., Yoneyama T., Hashimoto Y., Ito H., Nishimura S.I., Ohyama C. (2019). Serum N-glycan profiling is a potential biomarker for castration-resistant prostate cancer. Sci. Rep..

[68] Banda K., Gregg C.J., Chow R., Varki N.M., Varki A. (2012). Metabolism of vertebrate amino sugars with N-glycolyl groups: Mechanisms underlying gastrointestinal incorporation of the non-human sialic acid xeno-autoantigen N-glycolylneuraminic acid. J. Biol. Chem..

[69] Samraj A.N., Pearce O.M., Laubli H., Crittenden A.N., Bergfeld A.K., Banda K., Gregg C.J., Bingman A.E., Secrest P., Diaz S.L., Varki N.M., Varki A. (2015). A red meat-derived glycan promotes inflammation and cancer progression. Proc. Natl. Acad. Sci. U. S. A..

[70] Samraj A.N., Laubli H., Varki N., Varki A. (2014). Involvement of a non-human sialic Acid in human cancer. Front. Oncol..

[71] Loke I., Packer N.H., Thaysen-Andersen M. (2015). Complementary LC-MS/MS-based N-glycan, N-glycopeptide, and intact N-glycoprotein profiling reveals unconventional Asn71-glycosylation of human neutrophil cathepsin G. Biomolecules.

[72] Loke I., Ostergaard O., Heegaard N.H.H., Packer N.H., Thaysen-Andersen M. (2017). Paucimannose-rich N-glycosylation of spatiotemporally regulated human neutrophil elastase modulates its immune functions. Mol. Cell. Proteomics.

[73] Reiding K.R., Franc V., Huitema M.G., Brouwer E., Heeringa P., Heck A.J.R. (2019). Neutrophil myeloperoxidase harbors distinct site-specific peculiarities in its glycosylation. J. Biol. Chem..

[74] Briggs M.T., Condina M.R., Ho Y.Y., Everest-Dass A.V., Mittal P., Kaur G., Oehler M.K., Packer N.H., Hoffmann P. (2019). MALDI mass spectrometry imaging of early- and late-stage serous ovarian cancer tissue reveals stage-specific N-glycans. Proteomics.

[75] Drake R.R., Powers T.W., Jones E.E., Bruner E., Mehta A.S., Angel P.M. (2017). MALDI mass spectrometry imaging of N-linked glycans in cancer tissues. Adv. Cancer Res..

[76] Powers T.W., Neely B.A., Shao Y., Tang H., Troyer D.A., Mehta A.S., Haab B.B., Drake R.R. (2014). MALDI imaging mass spectrometry profiling of N-glycans in formalin-fixed paraffin embedded clinical tissue blocks and tissue microarrays. PLoS One.

[77] Wang D., Dafik L., Nolley R., Huang W., Wolfinger R.D., Wang L.X., Peehl D.M. (2013). Anti-oligomannose antibodies as potential serum biomarkers of aggressive prostate cancer. Drug Dev. Res..

[78] Park D.D., Phoomak C., Xu G., Olney L.P., Tran K.A., Park S.S., Haigh N.E., Luxardi G., Lert-Itthiporn W., Shimoda M., Li Q., Matoba N., Fierro F., Wongkham S., Maverakis E. (2020). Metastasis of cholangiocarcinoma is promoted by extended high-mannose glycans. Proc. Natl. Acad. Sci. U. S. A..

[79] Hagiwara K., Tobisawa Y., Kaya T., Kaneko T., Hatakeyama S., Mori K., Hashimoto Y., Koie T., Suda Y., Ohyama C., Yoneyama T. (2017). Wisteria floribunda agglutinin and its reactive-glycan-carrying prostate-specific antigen as a novel diagnostic and prognostic marker of prostate cancer. Int. J. Mol. Sci..

[80] Haga Y., Uemura M., Baba S., Inamura K., Takeuchi K., Nonomura N., Ueda K. (2019). Identification of multisialylated LacdiNAc structures as highly prostate cancer specific glycan signatures on PSA. Anal. Chem..

[81] Kariya Y., Kato R., Itoh S., Fukuda T., Shibukawa Y., Sanzen N., Sekiguchi K., Wada Y., Kawasaki N., Gu J. (2008). N-Glycosylation of laminin-332 regulates its biological functions. A novel function of the bisecting GlcNAc. J. Biol. Chem..

[82] Dang L., Shen J., Zhao T., Zhao F., Jia L., Zhu B., Ma C., Chen D., Zhao Y., Sun S. (2019). Recognition of bisecting N-glycans on intact glycopeptides by two characteristic ions in tandem mass spectra. Anal. Chem..

[83] Allam H., Aoki K., Benigno B.B., McDonald J.F., Mackintosh S.G., Tiemeyer M., Abbott K.L. (2015). Glycomic analysis of membrane glycoproteins with bisecting glycosylation from ovarian cancer tissues reveals novel structures and functions. J. Proteome Res..

[84] Jones J., Krag S.S., Betenbaugh M.J. (2005). Controlling N-linked glycan site occupancy. Biochim. Biophys. Acta.

[85] Schulz B.L., Stirnimann C.U., Grimshaw J.P., Brozzo M.S., Fritsch F., Mohorko E., Capitani G., Glockshuber R., Grutter M.G., Aebi M. (2009). Oxidoreductase activity of oligosaccharyltransferase subunits Ost3p and Ost6p defines site-specific glycosylation efficiency. Proc. Natl. Acad. Sci. U. S. A..

[86] Horak P., Tomasich E., Vanhara P., Kratochvilova K., Anees M., Marhold M., Lemberger C.E., Gerschpacher M., Horvat R., Sibilia M., Pils D., Krainer M. (2014). TUSC3 loss alters the ER stress response and accelerates prostate cancer growth *in vivo*. Sci. Rep..

[87] Thanka Christlet T.H., Veluraja K. (2001). Database analysis of O-glycosylation sites in proteins. Biophys. J..

[88] Darula Z., Medzihradszky K.F. (2018). Analysis of mammalian O-glycopeptides-we have made a good start, but there is a long way to go. Mol. Cell. Proteomics.

[89] Riley N.M., Malaker S.A., Driessen M.D., Bertozzi C.R. (2020). Optimal dissociation methods differ for N- and O-glycopeptides. J. Proteome Res..

[90] Hagisawa S., Ohyama C., Takahashi T., Endoh M., Moriya T., Nakayama J., Arai Y., Fukuda M. (2005). Expression of core 2 beta1,6-N-acetylglucosaminyltransferase facilitates prostate cancer progression. Glycobiology.

[91] Sato T., Yoneyama T., Tobisawa Y., Hatakeyama S., Yamamoto H., Kojima Y., Mikami J., Mori K., Hashimoto Y., Koie T., Ohyama C. (2016). Core 2 beta-1, 6-N-acetylglucosaminyltransferase-1 expression in prostate biopsy specimen is an indicator of prostate cancer aggressiveness. Biochem. Biophys. Res. Commun..

[92] Okamoto T., Yoneyama M.S., Hatakeyama S., Mori K., Yamamoto H., Koie T., Saitoh H., Yamaya K., Funyu T., Fukuda M., Ohyama C., Tsuboi S. (2013). Core2 O-glycan-expressing prostate cancer cells are resistant to NK cell immunity. Mol. Med. Rep..

[93] Perez-Riverol Y., Csordas A., Bai J., Bernal-Llinares M., Hewapathirana S., Kundu D.J., Inuganti A., Griss J., Mayer G., Eisenacher M., Perez E., Uszkoreit J., Pfeuffer J., Sachsenberg T., Yilmaz S. (2019). The PRIDE database and related tools and resources in 2019: Improving support for quantification data. Nucleic Acids Res..

[94] Chatterjee S., Lee L.Y., Kawahara R., Abrahams J.L., Adamczyk B., Anugraham M., Ashwood C., Sumer-Bayraktar Z., Briggs M.T., Chik J.H.L., Everest-Dass A., Forster S., Hinneburg H., Leite K.R.M., Loke I. (2019). Protein paucimannosylation is an enriched N-glycosylation signature of human cancers. Proteomics.

