# Supplementary material for: The Complexity and Dynamics of the Tissue Glycoproteome Associated With Prostate Cancer Progression
Source: Mol Cell Proteomics. 2021 Jan 5;20:100026. doi: 10.1074/mcp.RA120.002320 (PMC8010466; doi:10.1074/mcp.RA120.002320)

Supporting Information (SI) for

The complexity and dynamics of the tissue glycoproteome associated with prostate cancer progression

Rebeca Kawahara[1,2,3], Saulo Recuero[4], Miguel Srougi[4], Katia R.M. Leite[4], Morten Thaysen-Andersen[2,3#§], and Giuseppe Palmisano[1#§]

[1]Instituto de Ciências Biomédicas, Departamento de Parasitologia, Universidade de São Paulo, USP, São Paulo, Brazil.
[2]Department of Molecular Sciences, Macquarie University, Sydney, NSW, Australia
[3]Biomolecular Discovery Research Centre, Macquarie University, Sydney, NSW, Australia

[4]Laboratório de Investigação Médica da Disciplina de Urologia da Faculdade de Medicina da USP, LIM55, São Paulo, Brazil.

[§] These authors share authorship

* To whom correspondence should be addressed:

i. GlycoProteomics Laboratory, Department of Parasitology, Institute of Biomedical Sciences, University of São Paulo, Avenida Lineu Prestes 1374, CEP: 05508-000, São Paulo, Brazil. Email: palmisano.gp@usp.br

ii. Department of Molecular Sciences, Macquarie University, Sydney, NSW 2109, Australia. E-mail: morten.andersen@mq.edu.au

Keywords: glycosylation, glycomics, glycoproteomics, mass spectrometry, prostate cancer

Running title: The glycoproteome dynamics underpinning prostate cancer progression

Supplementary Figures Legends

Supplemental Figure 1 Example of N-glycan isomeric separation by PGC and MSMS fragment-specific substructure diagnostic ions. A) Distinct PGC-LC elution pattern for α2,6 and α2,3 sialic acid and fragment-specific substructure diagnostic ions for core fucosylation and 6 arm composition. B) Fragment-specific substructure diagnostic ions for LacdiNac containing N-glycans. C) Fragment-specific substructure diagnostic ions for bisecting containing N-glycans. D) Fragment-specific substructure diagnostic ions for NeuGc containing N-glycans.

Supplementary Figure 2 Overview of the reproducibility of unique glycoform identified in the prostate tissue. A) Distribution of (number) and % of unique glycoforms identified across 10-100% of the 54 files. The numbers in parenthesis show the number of glycoforms. B) Distribution of (number) and % of unique glycoforms identified across 1-6 conditions (five Pca grades and BPH) is shown in the bar graph.

Supplementary Tables Legends

Supplemental Table SA: Clinical information of the patients included in the study

Supplementary Table SB: Overview of the raw files and search parameters.

Supplementary Table S1: N-glycome dataset of PCa and BPH tissues.

Supplementary Table S2: O-glycome dataset of PCa and BPH tissues.

Supplementary Table S3: Intact N-glycopeptide dataset of PCa and BPH tissues.

Supplementary Table S4: Intact O-glycopeptide dataset of PCa and BPH tissues.

Supplementary Table S5: De-N-glycopeptide dataset of PCa and BPH tissues.

Supplementary Table S6: Proteome dataset of PCa and BPH tissues.

Supplementary Table S7: Significant correlated intact N-glycopeptide with N-glycan structure.

Supplementary Table S8: Significant correlated intact O-glycopeptide with O-glycan structure.

Supplementary Data Legends

Supplementary Data S1: Spectra evidences for reduced N-glycans (alditols) released from PCa and BPH tissues

Supplementary Data S2: Spectra evidences for reduced O-glycans (alditols) released from PCa and BPH tissues

Supplementary Figures

Supplementary Figure S1

A

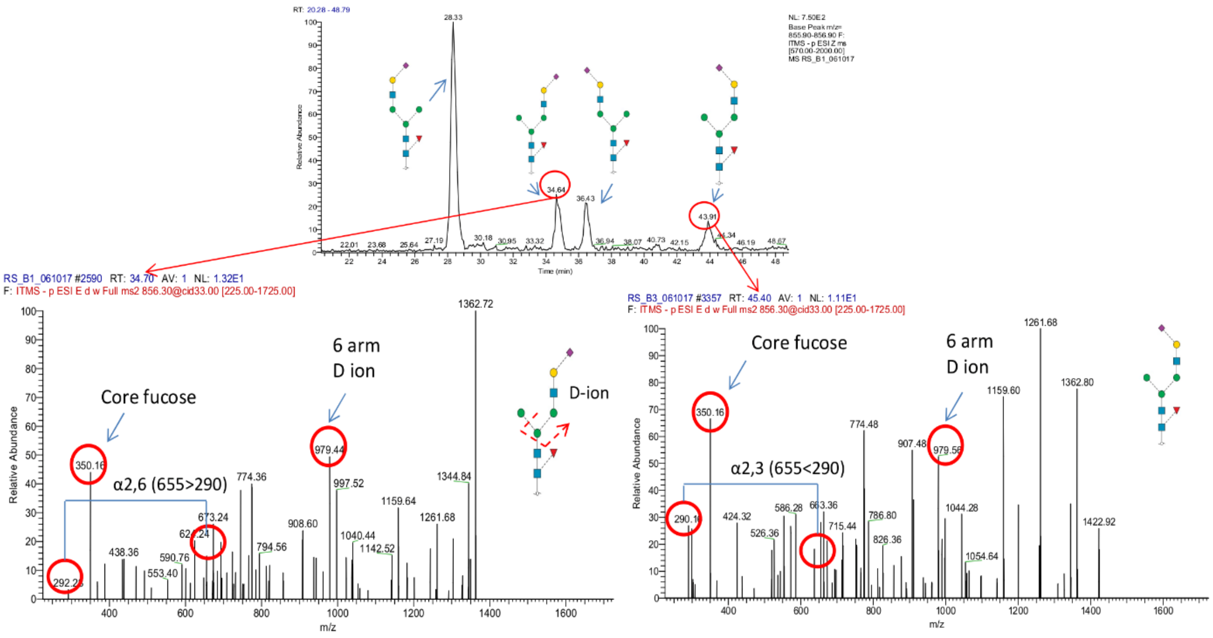

B

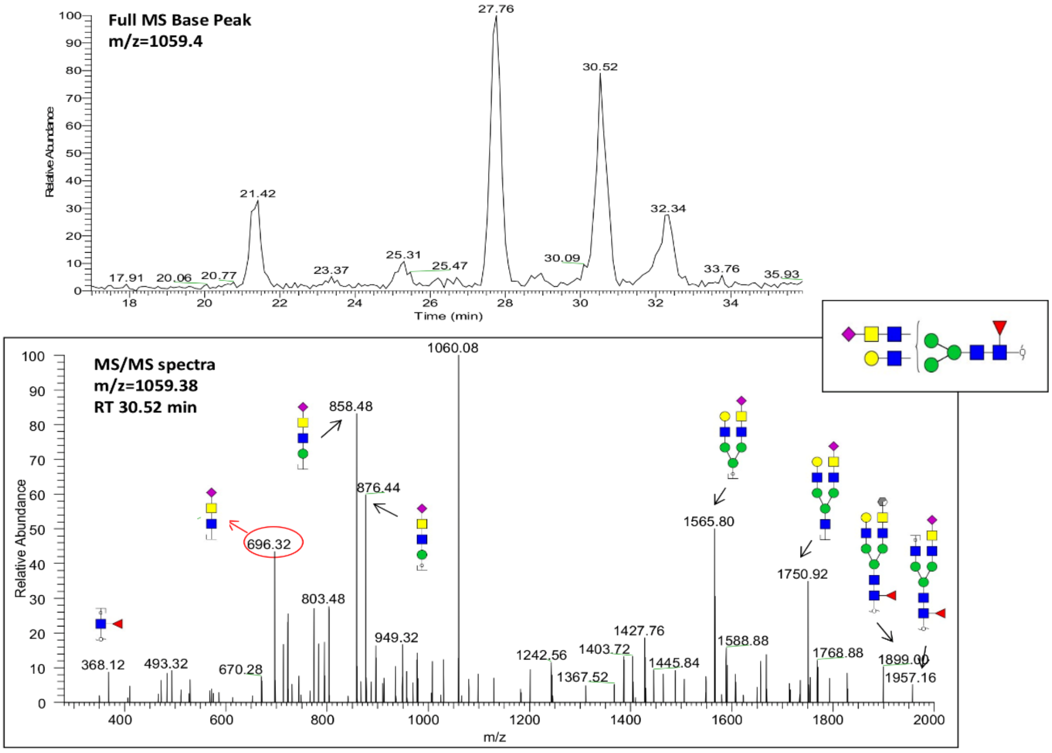

C

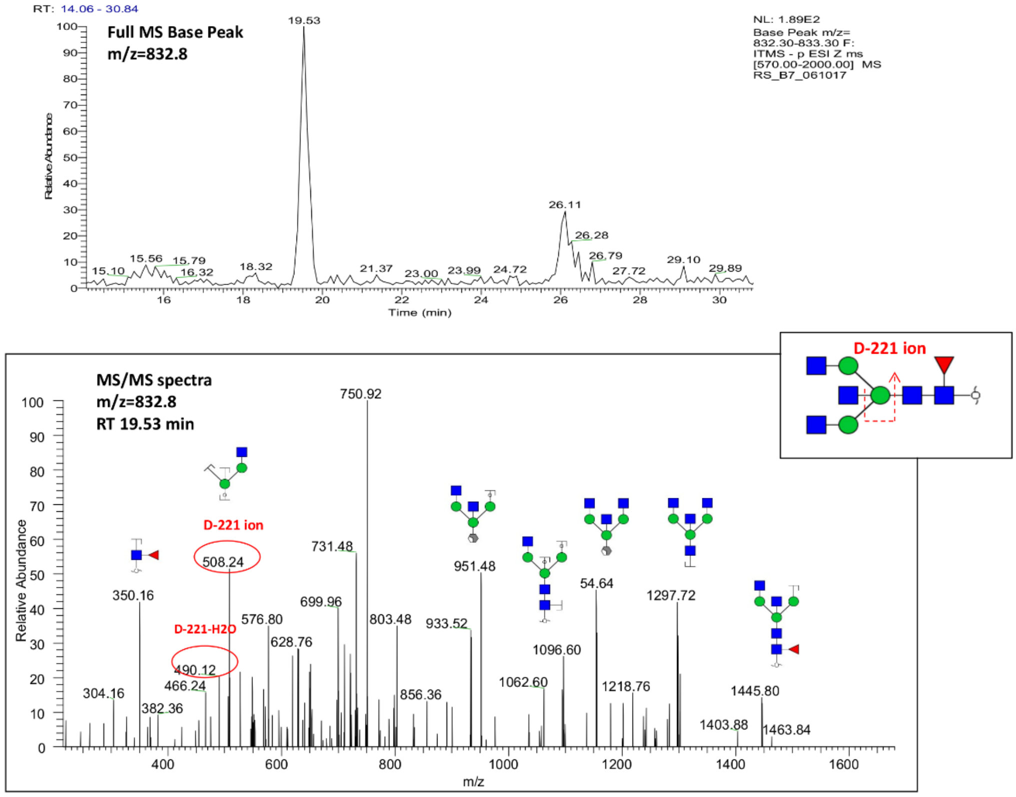
RT: 14.06 - 30.84
Full MS Base Peak
m/z=832.8
NL: 1.89E2
Base Peak m/z= 832.30-833.30 F: ITMS - p ESI Z ms [570.00-2000.00] MS RS_B7_061017
Relative Abundance
Time (min)
19.53
26.11
26.28
26.79
27.72
29.10
29.89
15.10
15.56
15.79
16.32
18.32
21.37
23.00
23.99
24.72
MS/MS spectra
m/z=832.8
RT 19.53 min
D-221 ion
D-221-H2O
750.92
731.48
699.96
508.24
576.80
628.76
803.48
933.52
951.48
350.16
490.12
466.24
304.16
382.36
856.36
1062.60
1096.60
54.64
1297.72
1218.76
1445.80
1403.88
1463.84
Relative Abundance
m/z

D

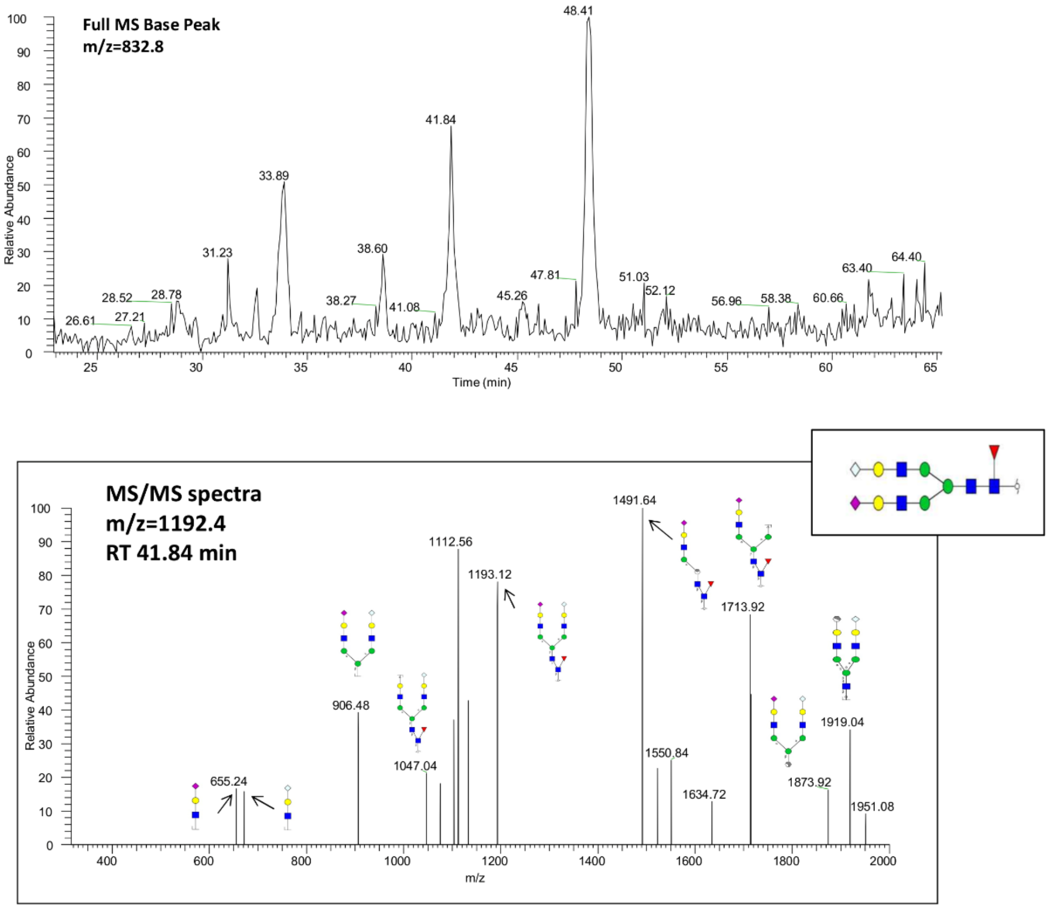
Full MS Base Peak
m/z=832.8
Relative Abundance
Time (min)
48.41
41.84
33.89
31.23
38.60
28.52
28.78
26.61
27.21
38.27
41.08
45.26
47.81
51.03
52.12
56.96
58.38
60.66
63.40
64.40
MS/MS spectra
m/z=1192.4
RT 41.84 min
1491.64
1112.56
1193.12
1713.92
906.48
1047.04
655.24
1550.84
1634.72
1873.92
1919.04
1951.08
Relative Abundance
m/z

Supplementary Figure S2

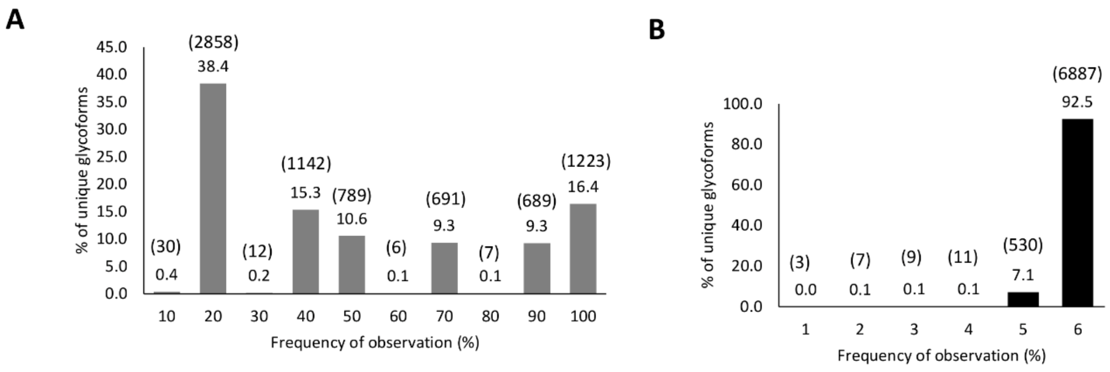

Supplement: MCPRO_MCP/2020/002320_6.pdf [file mmc1.pdf]
