# Supplementary material for: The Complexity and Dynamics of the Tissue Glycoproteome Associated With Prostate Cancer Progression
Source: Mol Cell Proteomics. 2021 Jan 5;20:100026. doi: 10.1074/mcp.RA120.002320 (PMC8010466; doi:10.1074/mcp.RA120.002320)
Supplement: MCPRO_MCP/2020/002320_8.pdf [file mmc3.pdf]

Supplementary Data S1 and S2

for

The complexity and dynamics of the tissue glycoproteome associated with prostate cancer progression

Rebeca Kawahara[1,2,3], Saulo Recuero[4], Miguel Srougi[4], Katia R.M. Leite[4], Morten Thaysen-Andersen[2,3#§], and Giuseppe Palmisano[1#§]

[1]Instituto de Ciências Biomédicas, Departamento de Parasitologia, Universidade de São Paulo, USP, São Paulo, Brazil.

[2]Department of Molecular Sciences, Macquarie University, Sydney, NSW, Australia

[3]Biomolecular Discovery Research Centre, Macquarie University, Sydney, NSW, Australia

Annotation and Fragmentation Key

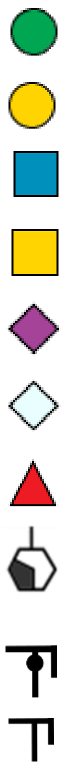

Mannose (162.0528 Da)

Galactose (162.0528 Da)

N-Acetylglucosamine (203.0794 Da)

N-Acetylgalactosamine (203.0794 Da)

N-Acetylneuraminic acid (291.0954 Da)

N-glycolyl neuraminic acid (307.0903 Da)

Fucose (146.0579 Da)

Cross-ring fragment (unspecified)

Indicates mostly Y ions (includes oxygen of glyosidic linkage)

Indicates mostly Z ions (excludes oxygen of glyosidic linkage)

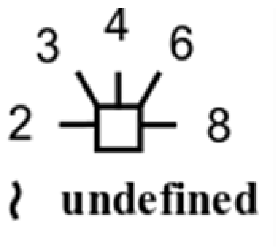

Comment 1: All glycans contain reducing end which is not depicted in the *N*-glycan structure cartoon

Comment 2: linkage represented only for α2,6 and α2,3 sialic acid residues

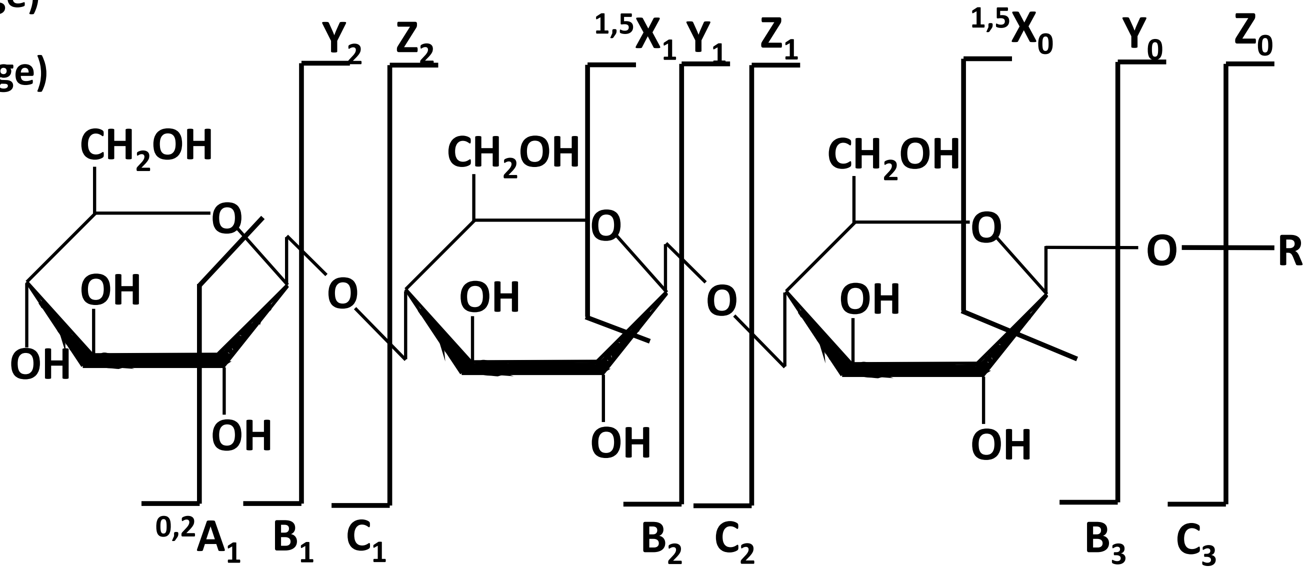

Han, L. & Costello, C.E. Mass spectrometry of glycans. *Biochemistry (Moscow)* 78, 710-720 (2013).

Supplementary Data S1

Spectra evidences for reduced *N*-glycans (alditols) released from PCa and BPH tissues

The *N*-glycans reported in this paper were characterised and quantified by:

a. EIC (MS) and monoisotopic integration area (blue highlights in insert) (Skyline) (top)
b. Retention time
c. CID-MS/MS (-) manual annotation (bottom)

Supporting literature:

1. Hinneburg, H., Pedersen, J. L., Bokil, N. J., Pralow, A., Schirmeister, F., Kawahara, R., Rapp, E., Saunders, B. M., and Thaysen-Andersen, M. (2020) High-resolution longitudinal N- and O-glycoprofiling of human monocyte-to- macrophage transition. *Glycobiology*
2. Abrahams, J. L., Campbell, M. P., and Packer, N. H. (2018) Building a PGC-LC-MS N-glycan retention library and elution mapping resource. *Glycoconj J* **35**, 15-29
3. Everest-Dass, A. V., Abrahams, J. L., Kolarich, D., Packer, N. H., and Campbell, M. P. (2013) Structural feature ions for distinguishing N- and O-linked glycan isomers by LC-ESI-IT MS/MS. *J Am Soc Mass Spectrom* **24**, 895-906

(HexNAc)2 (Deoxyhexose)1

GlytouCan ID: G99948OF

[M-H] 571.2

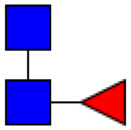

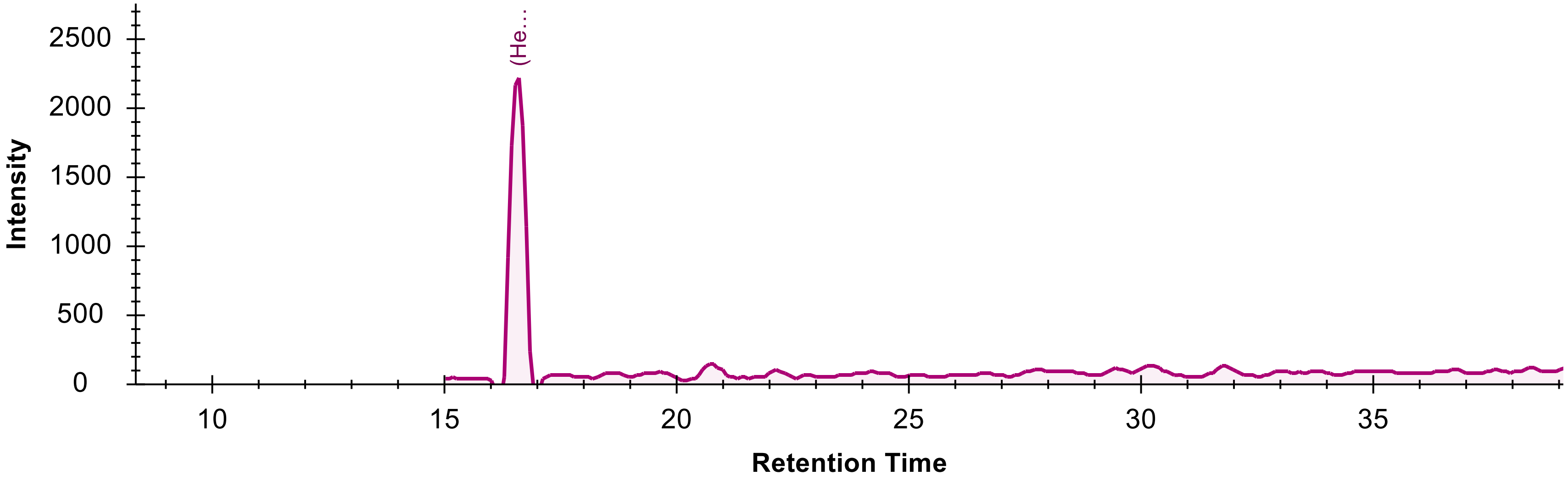

CID-MS/MS (*m/z* 571.20 $^{1-}$)

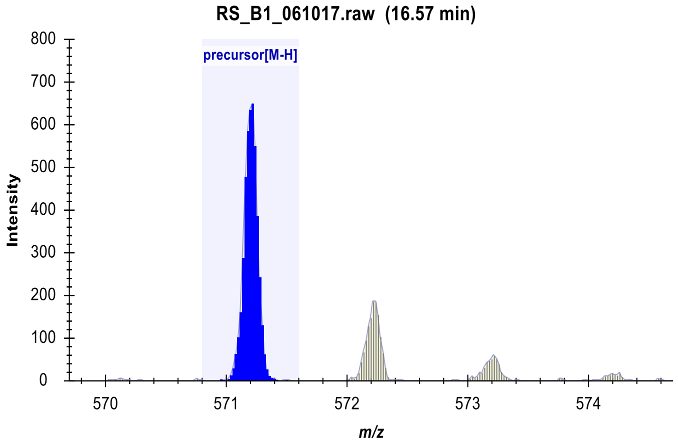

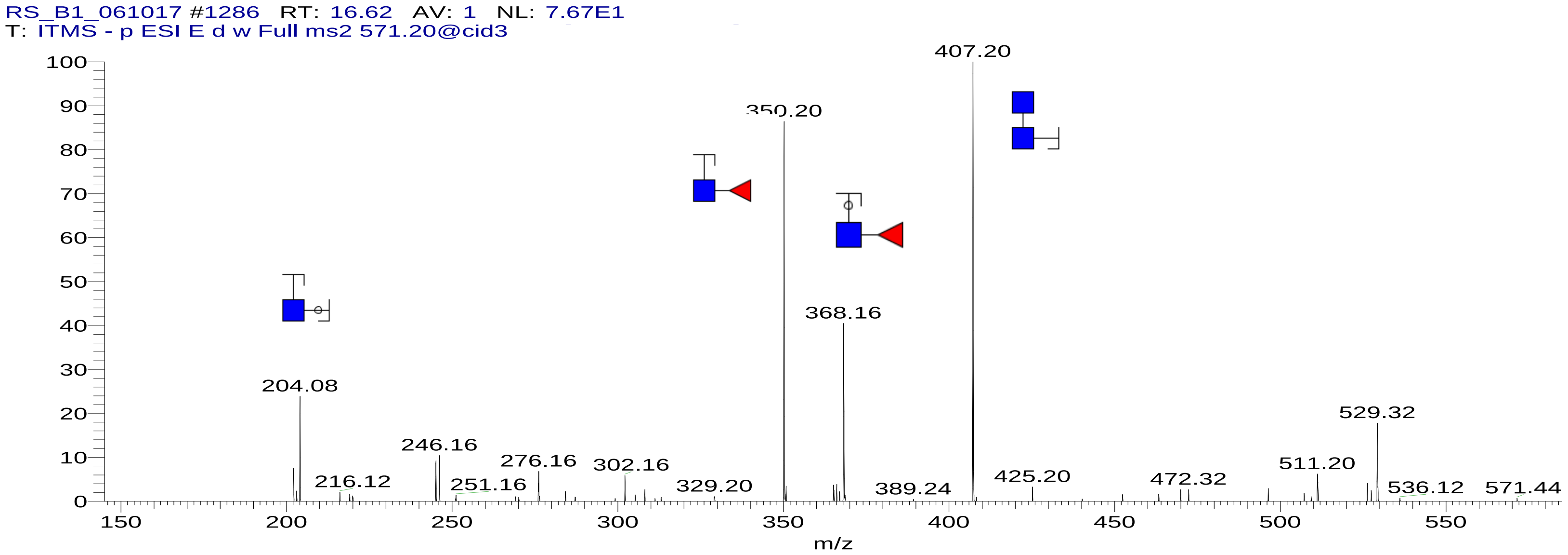

Glycan ID: 1

(Hex)1 (HexNAc)2

GlytouCan ID: G14469GR

[M-H] 587.28

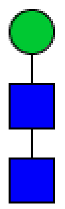

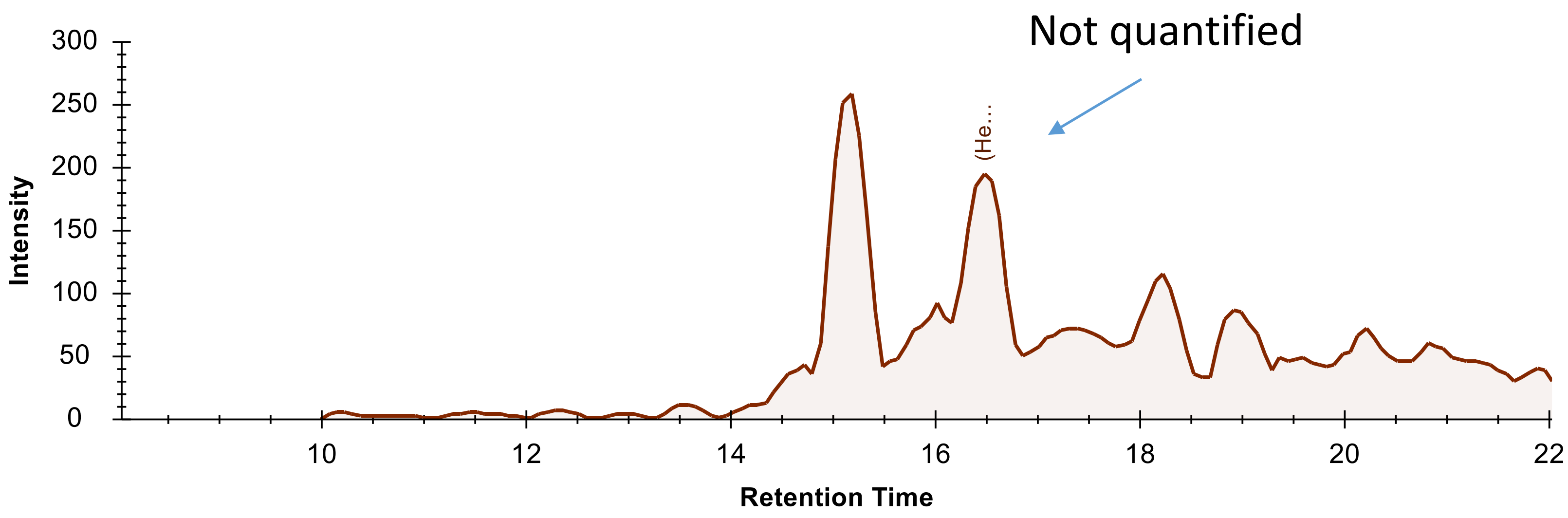

CID-MS/MS (*m/z* 587.28 $^{1-}$)

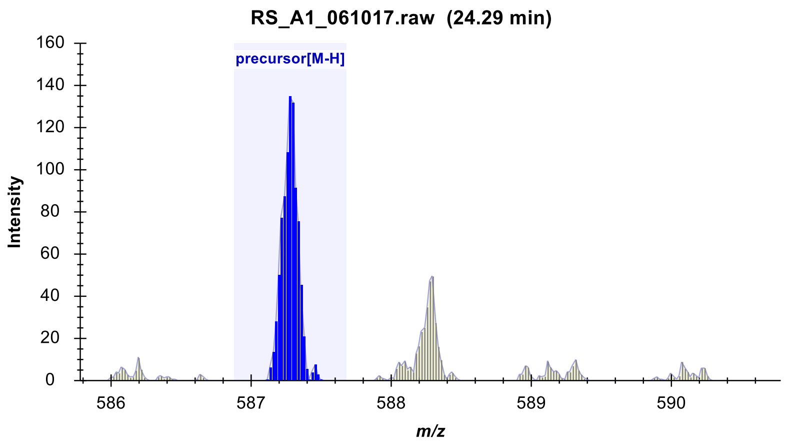

Glycan ID: 2

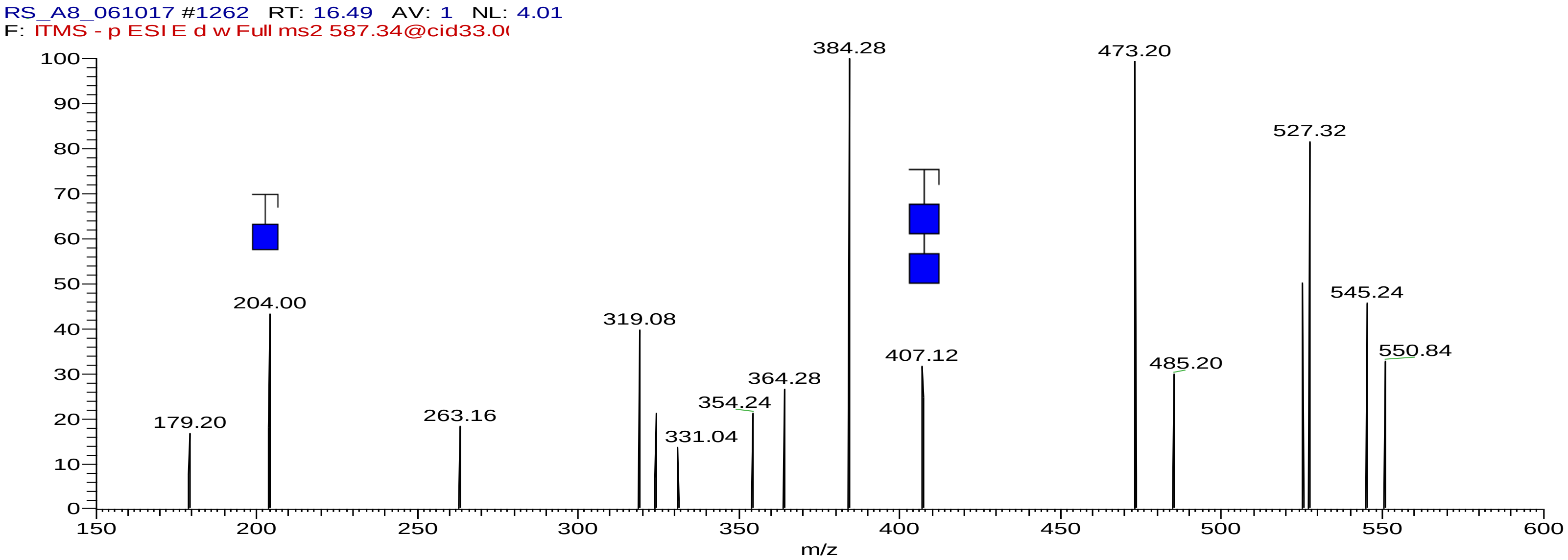

(Hex)1 (HexNAc)2 (Deoxyhexose)1
GlytouCan ID: G37641EM
[M-H] 733.27

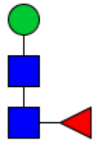

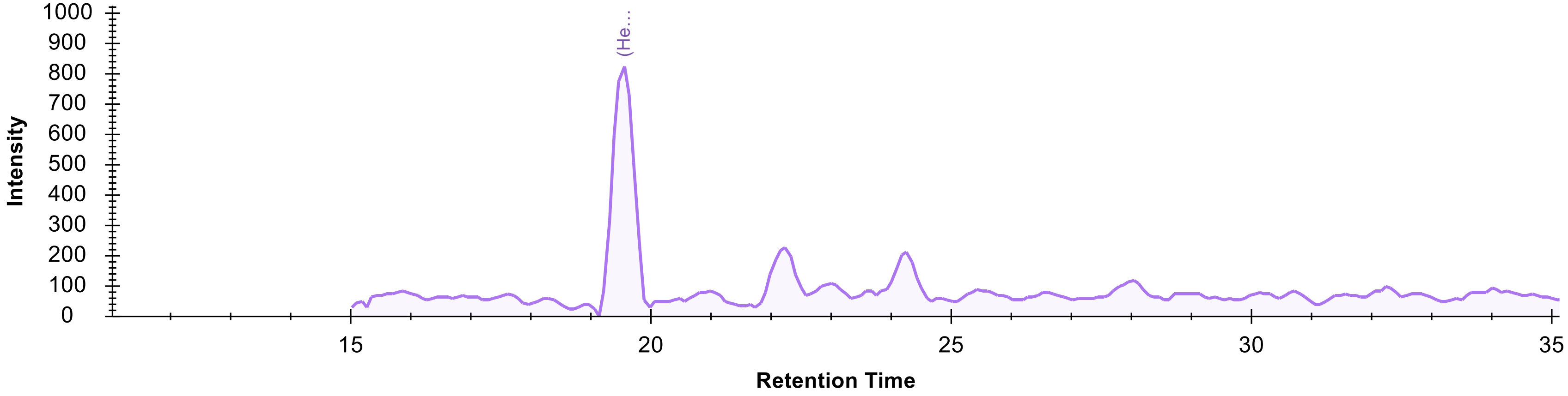

CID-MS/MS (m/z 733.27 $^{1-}$)

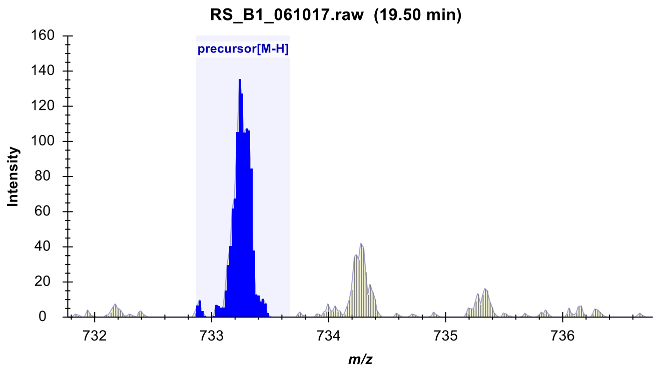

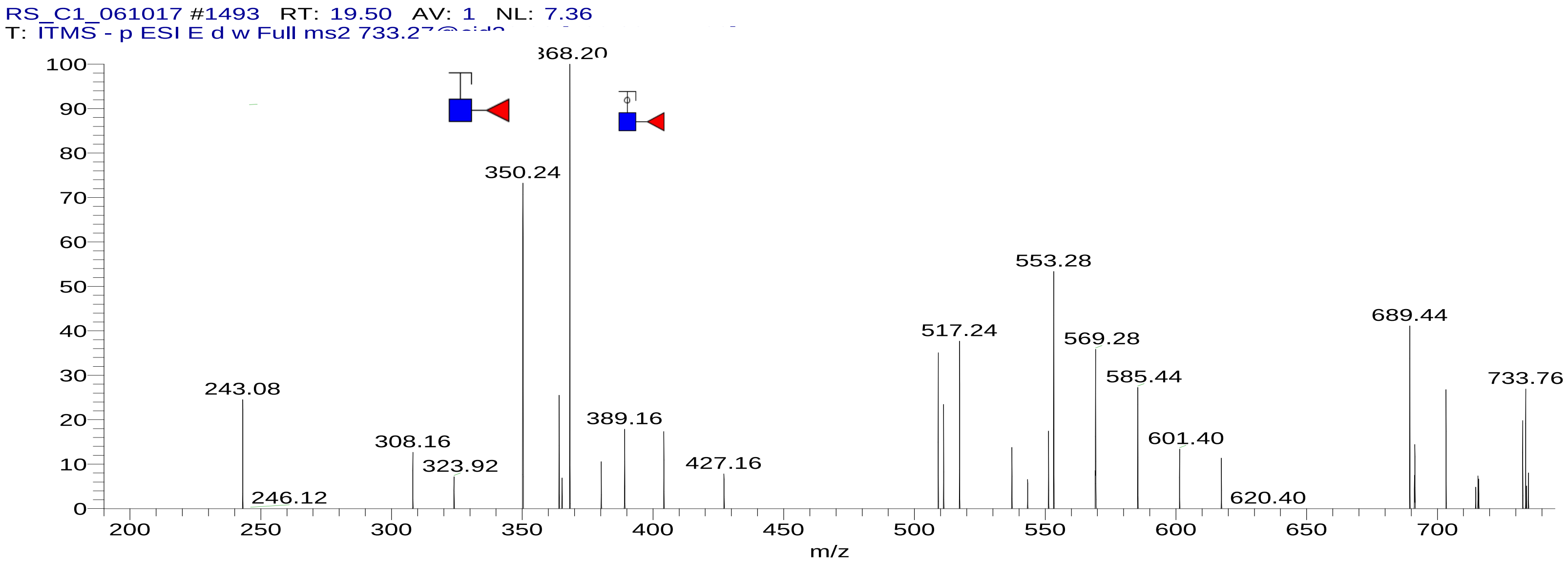

Glycan ID: 3

(Hex)2 (HexNAc)2

GlytouCan ID: G13947MP
[M-H] 749.26

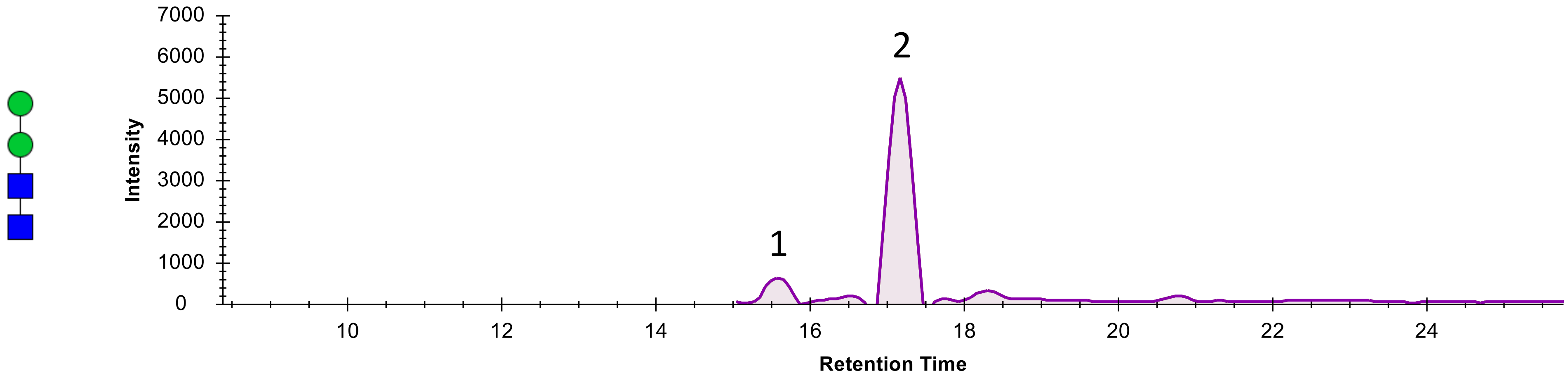

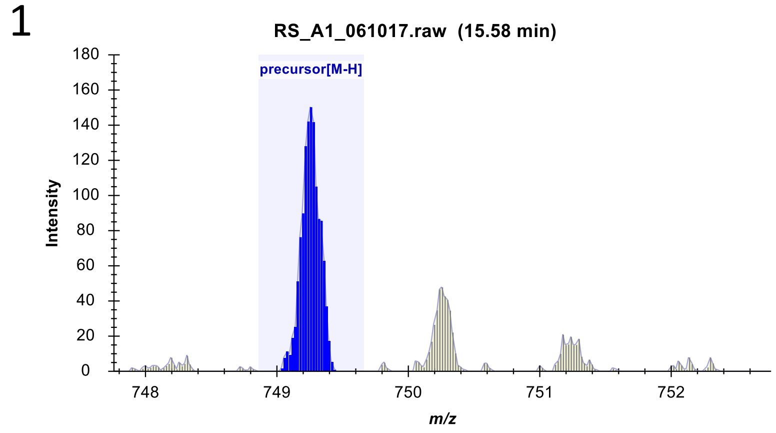

4a

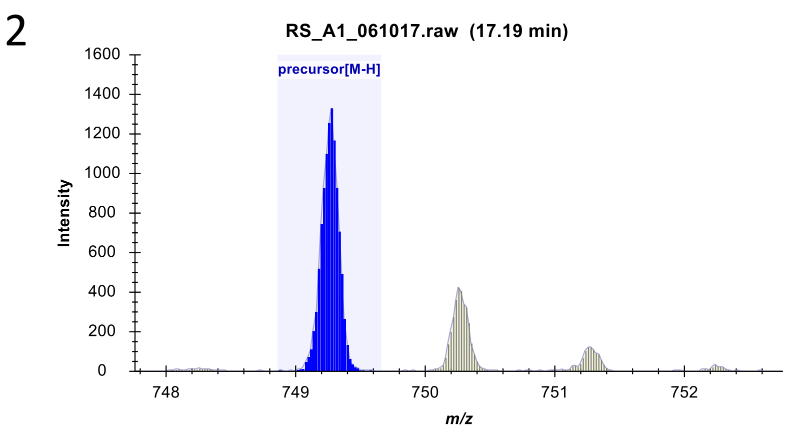

4b

GlytouCan ID: G13947MP

CID-MS/MS
(*m/z* 749.26 $^{1-}$)

4a

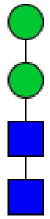

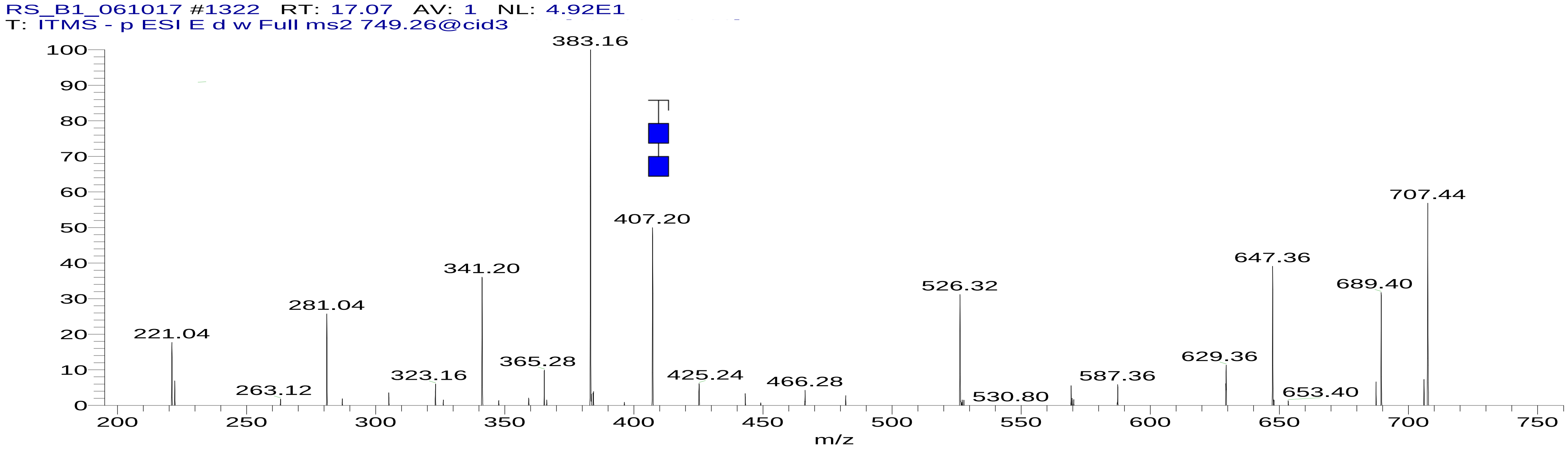

4b

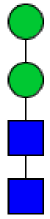

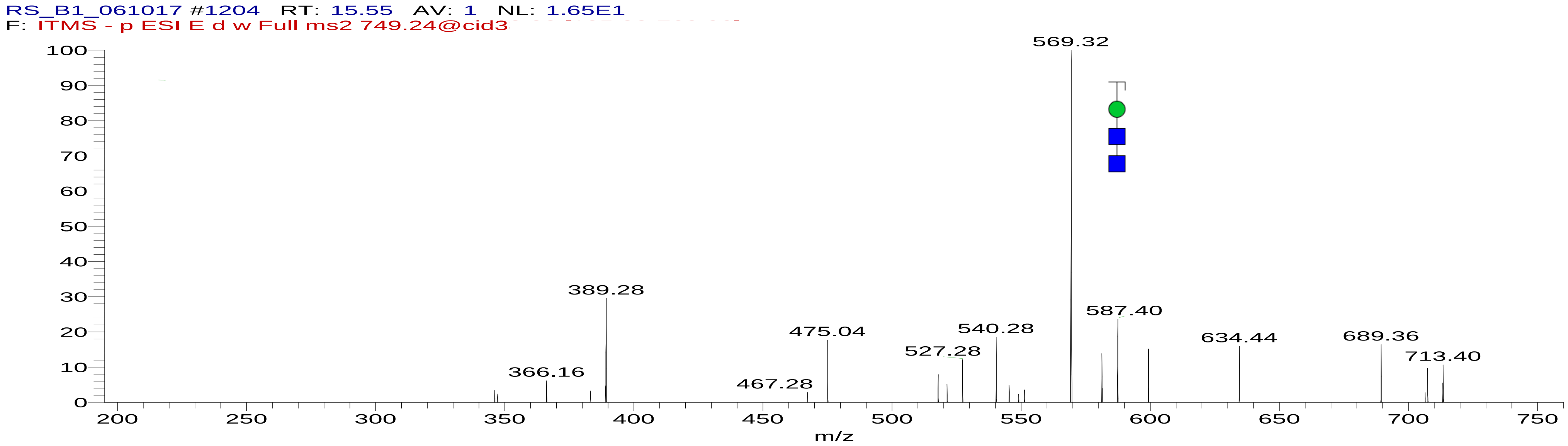

(Hex)2 (HexNAc)2 (Deoxyhexose)1
GlytouCan ID: G09088BU
[M-H] 895.33

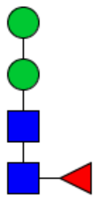

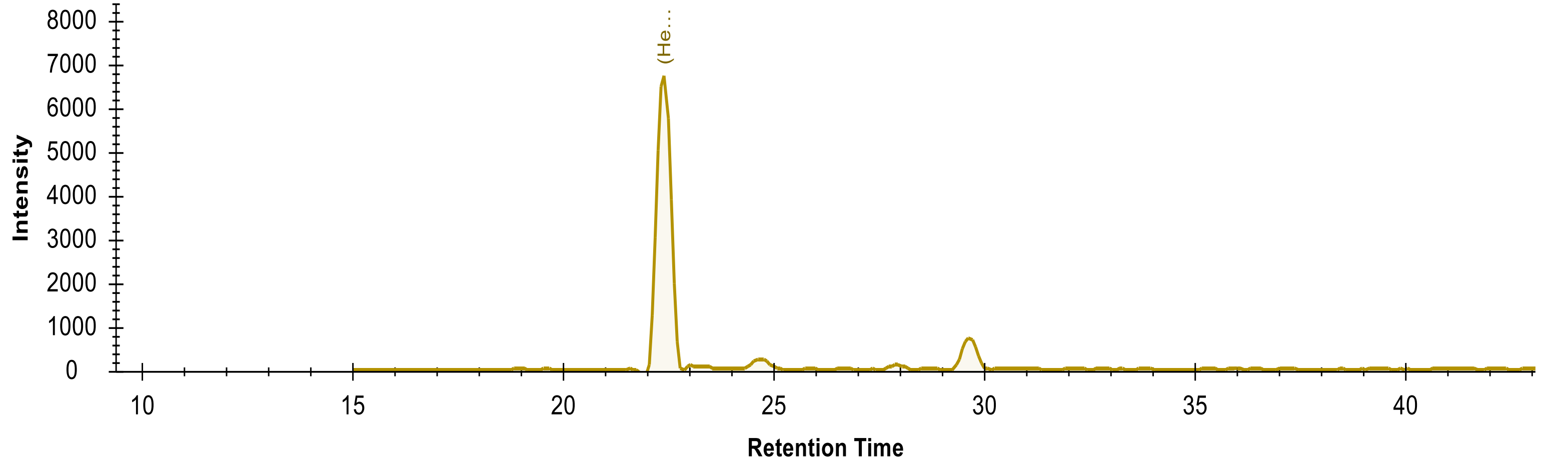

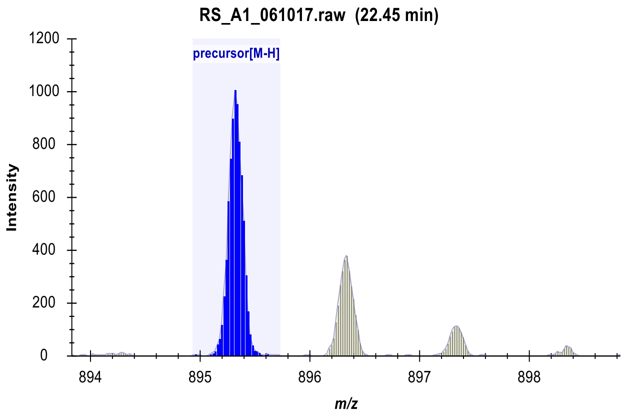

Glycan ID: 5

CID-MS/MS (*m/z* 895.53 [1-])

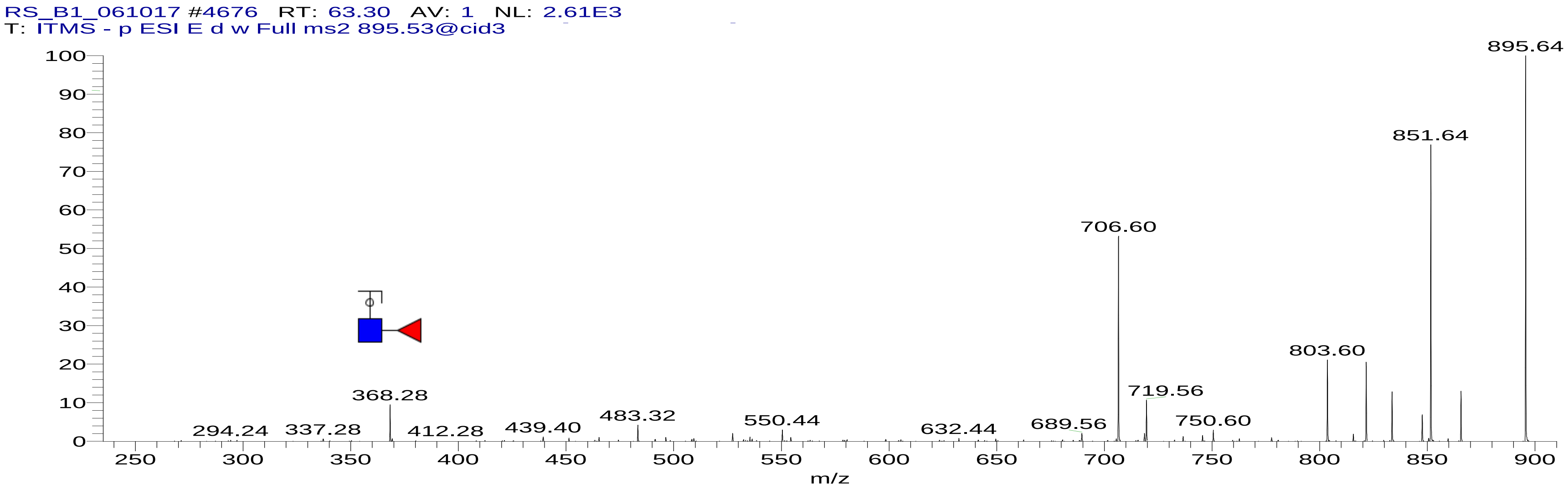

(Hex)3 (HexNAc)2

GlytouCan ID: G62167DK

[M-H] 911.32

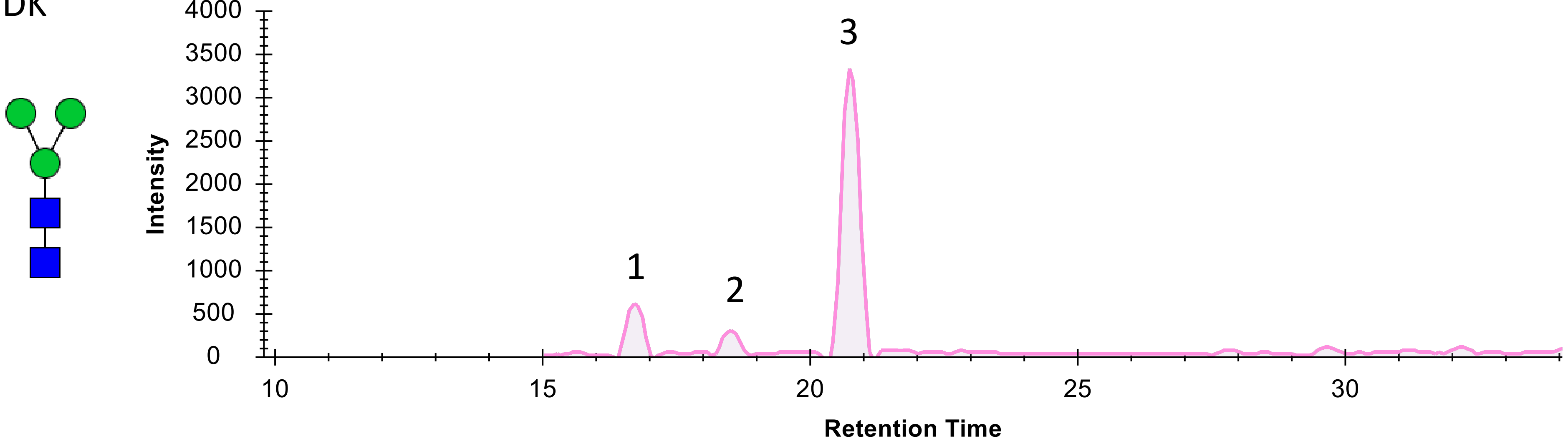

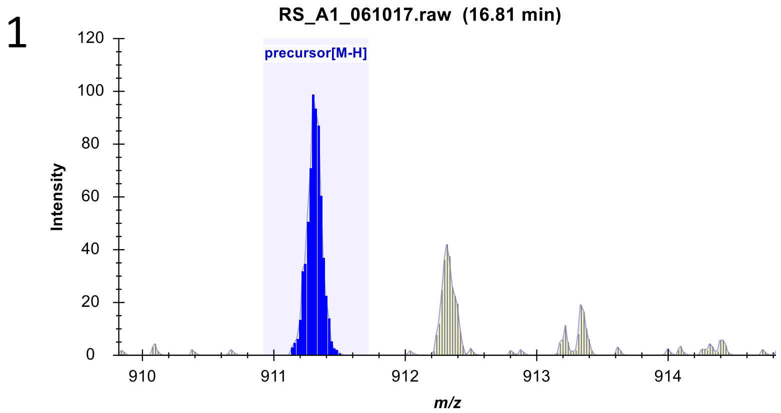

6a

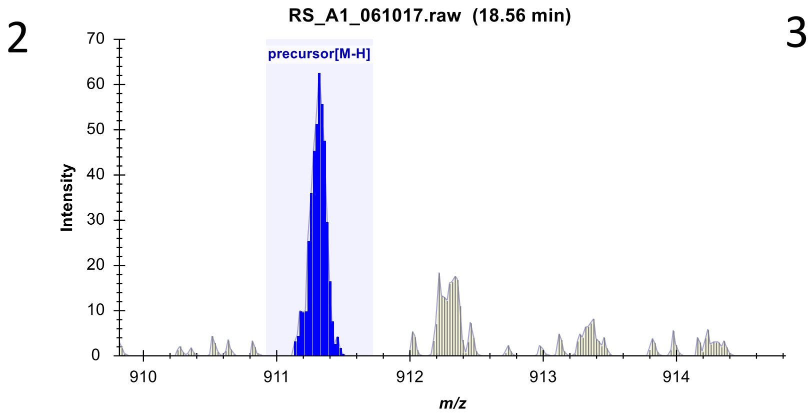

6b

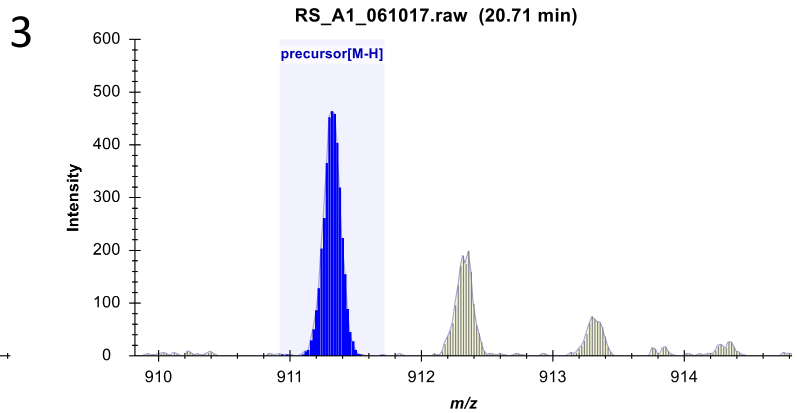

6c

GlytouCan ID: G62167DK

CID-MS/MS
(*m/z* 911.30 [1-])

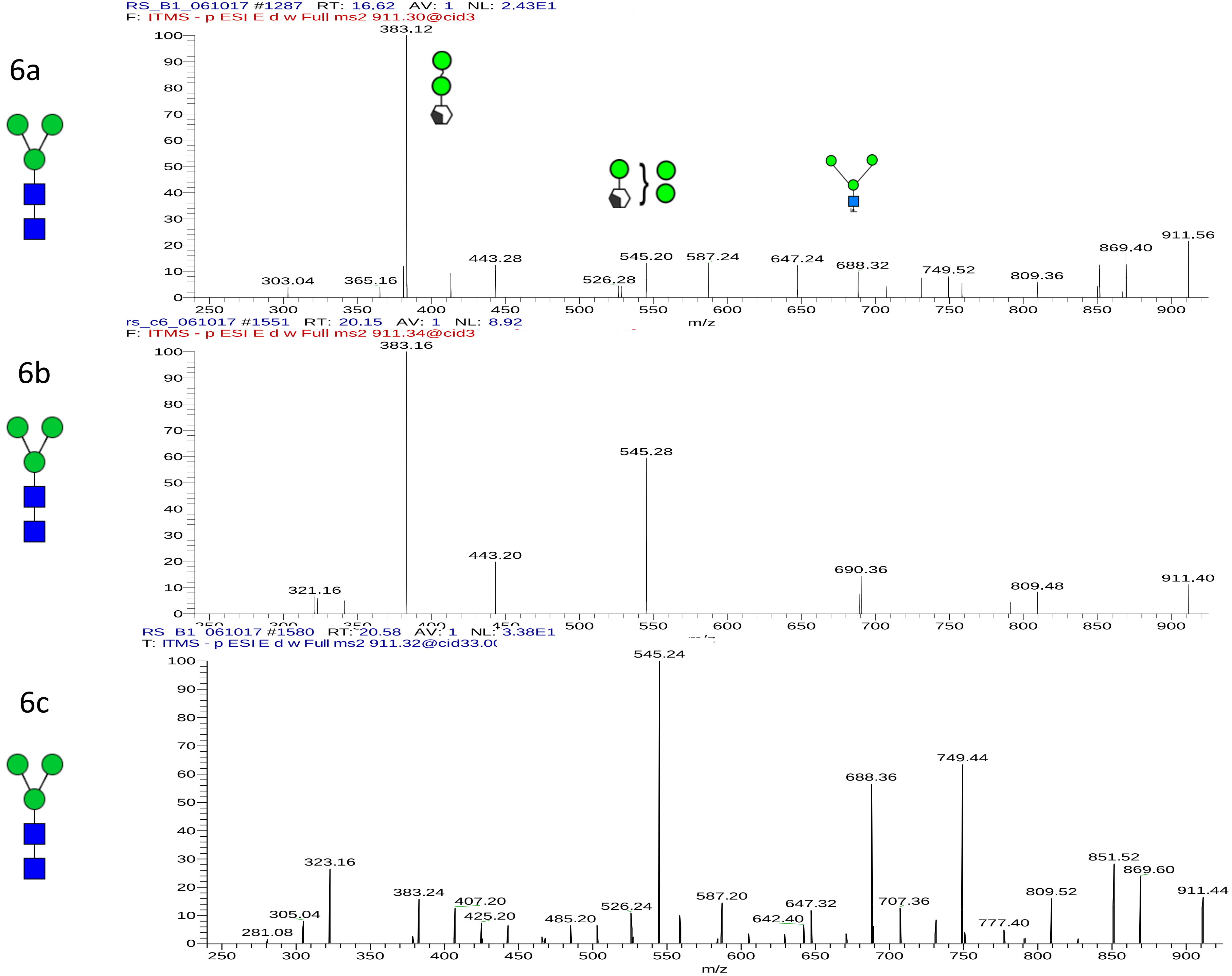

(Hex)3 (HexNAc)2

GlytouCan ID: G77712UR

[M-H] 1057.38

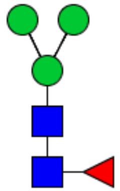

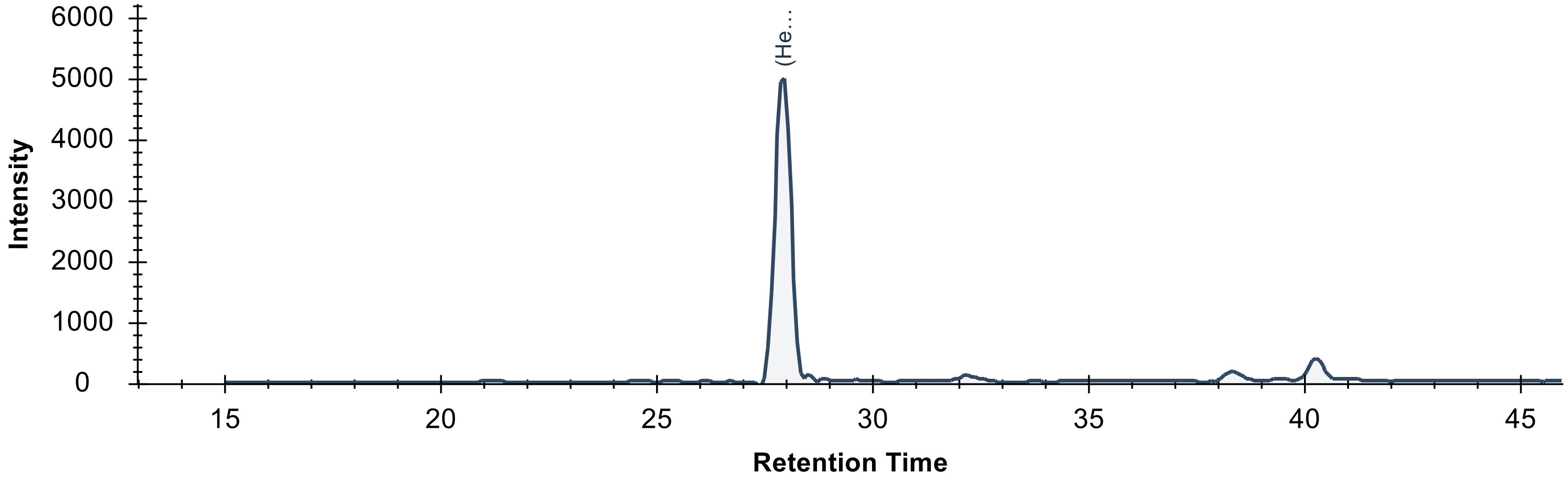

CID-MS/MS (m/z 1057 $^{1-}$)

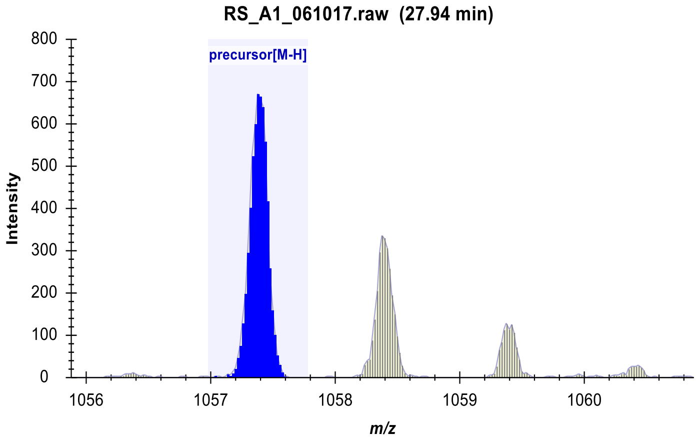

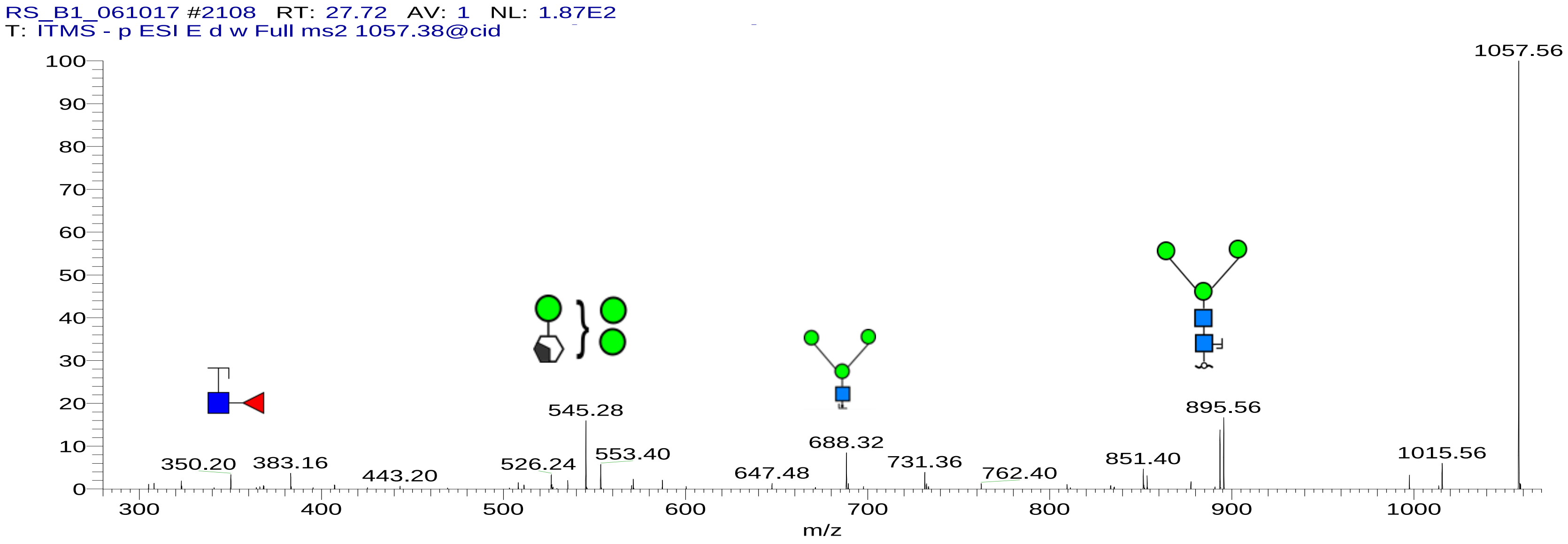

Glycan ID: 7

(Hex)2 + (Man)3(GlcNAc)2
GlytouCan ID: G13974DV

[M-2H]	617.22
[M-H]	1235.48

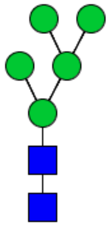

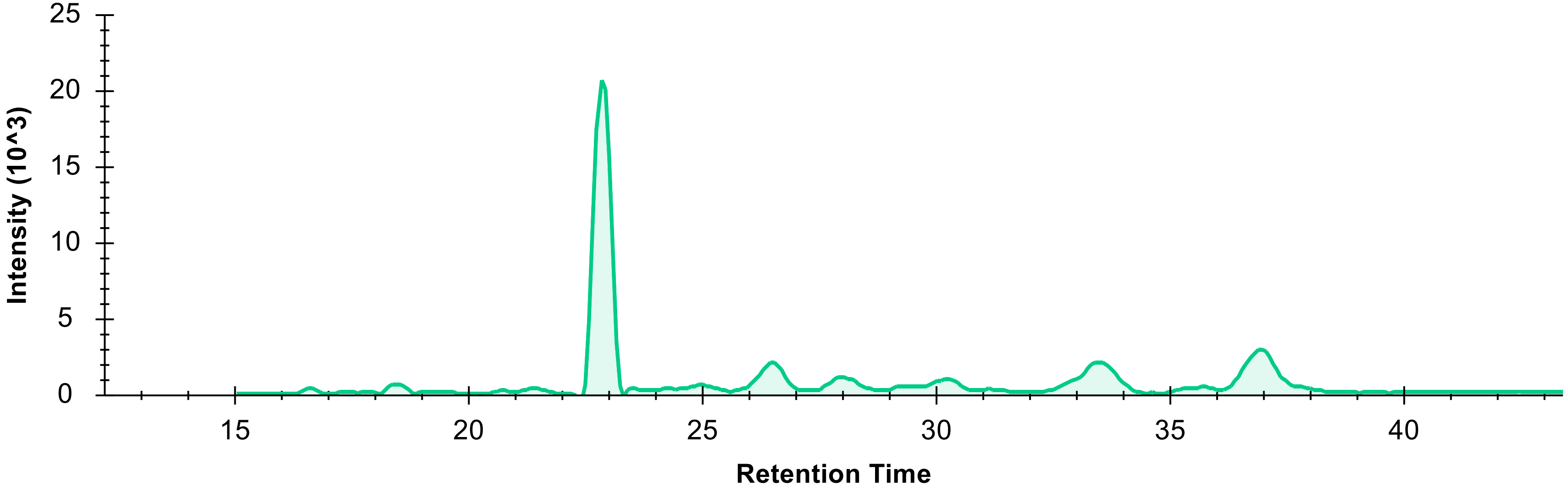

CID-MS/MS (*m/z* 617.22 $^{2-}$)

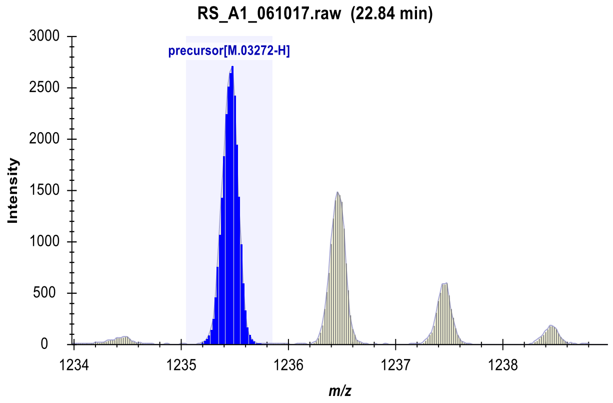

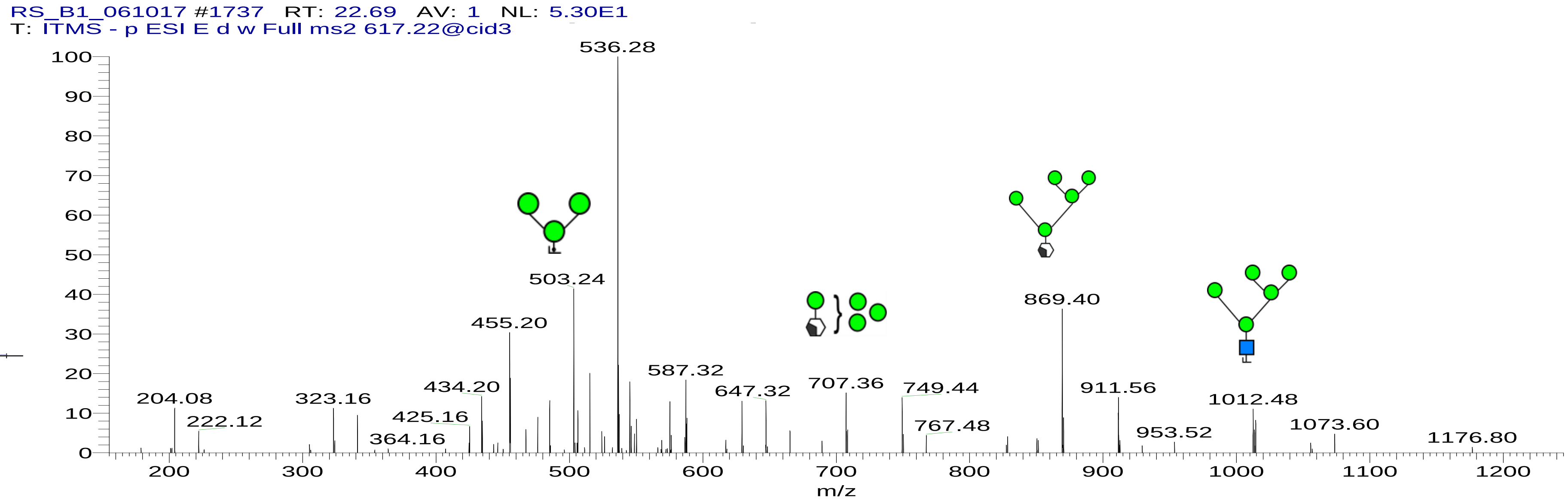

Glycan ID: 8

(Hex)3 + (Man)3(GlcNAc)2

GlytouCan ID: G19949UL

[M-2H]	698.24
[M-H]	1397.53

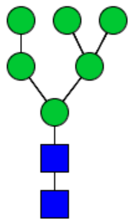

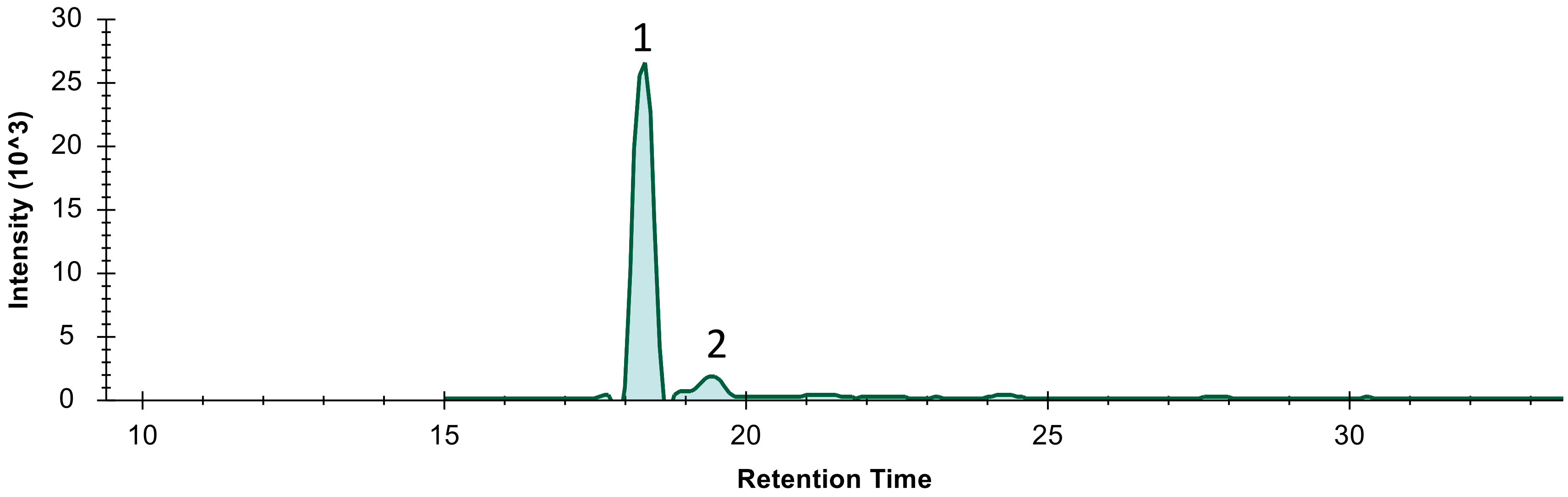

1

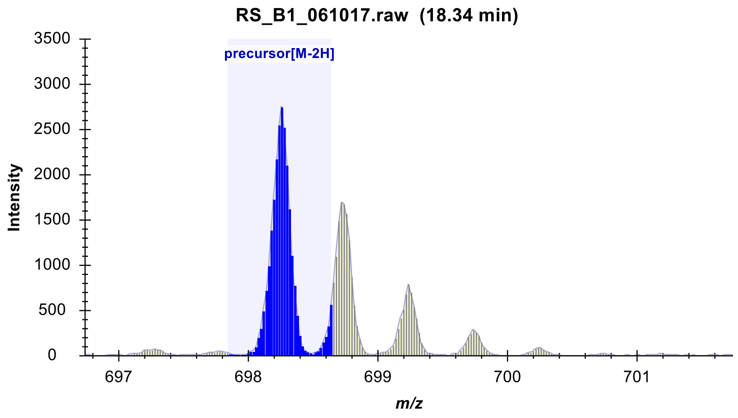

9a

2

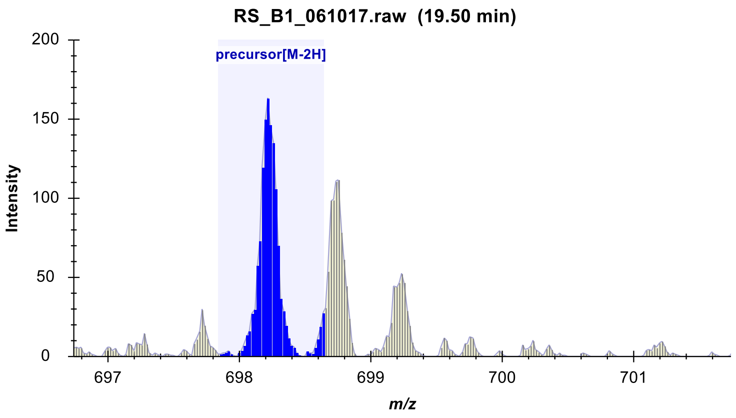

9b

GlytouCan ID: G19949UL

CID-MS/MS
(*m/z* 698.24 $^{2-}$)

9a

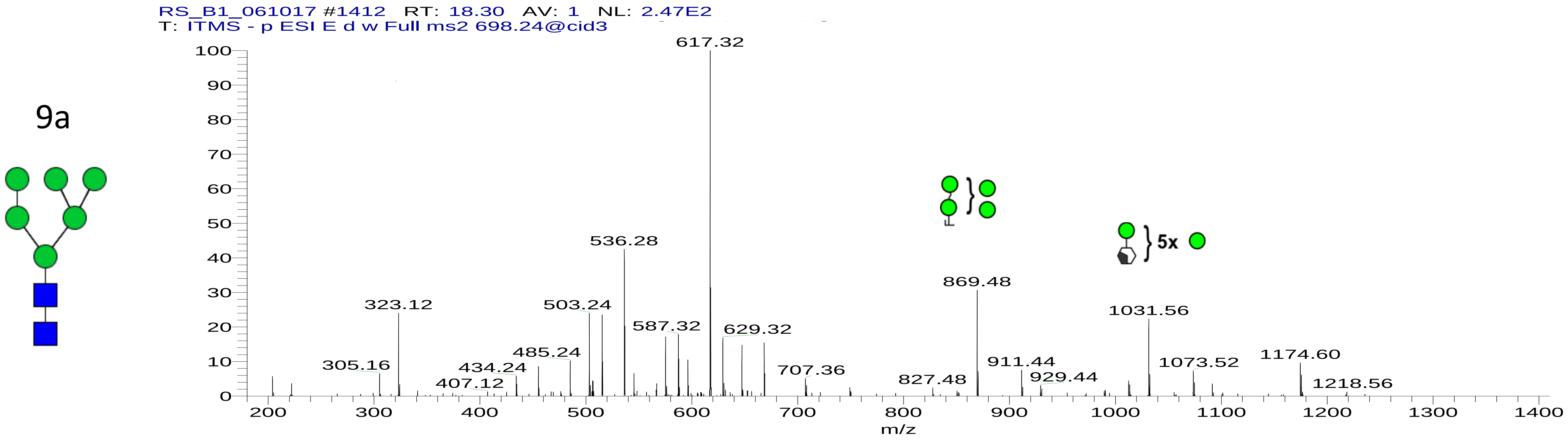

9b

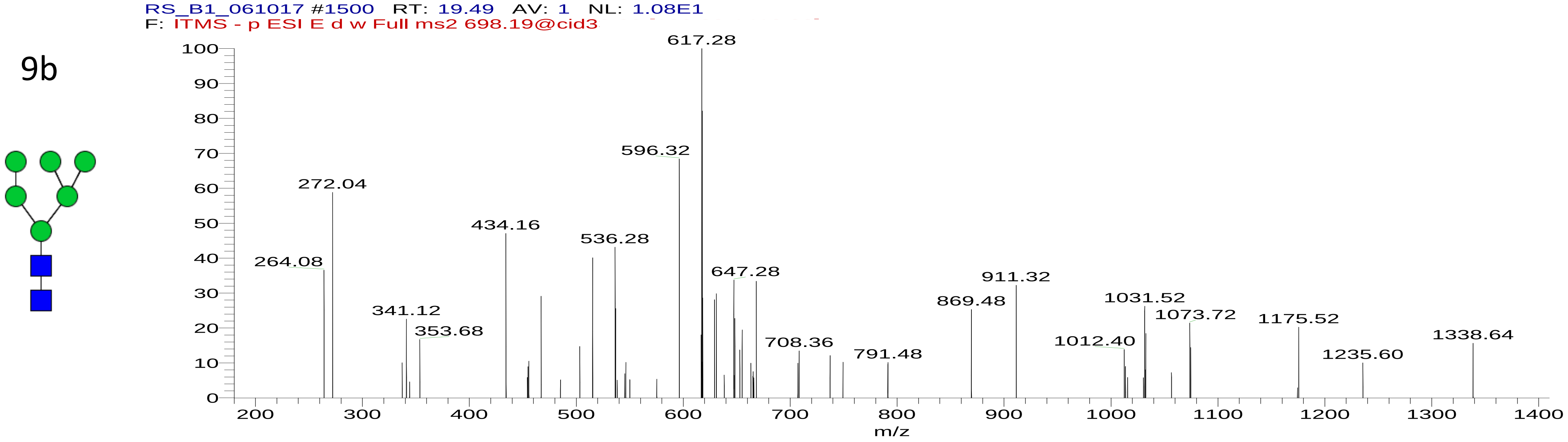

(Hex)4 (HexNAc)2

GlytouCan ID: G92509FK

[M-H] 1073.38

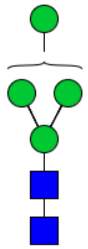

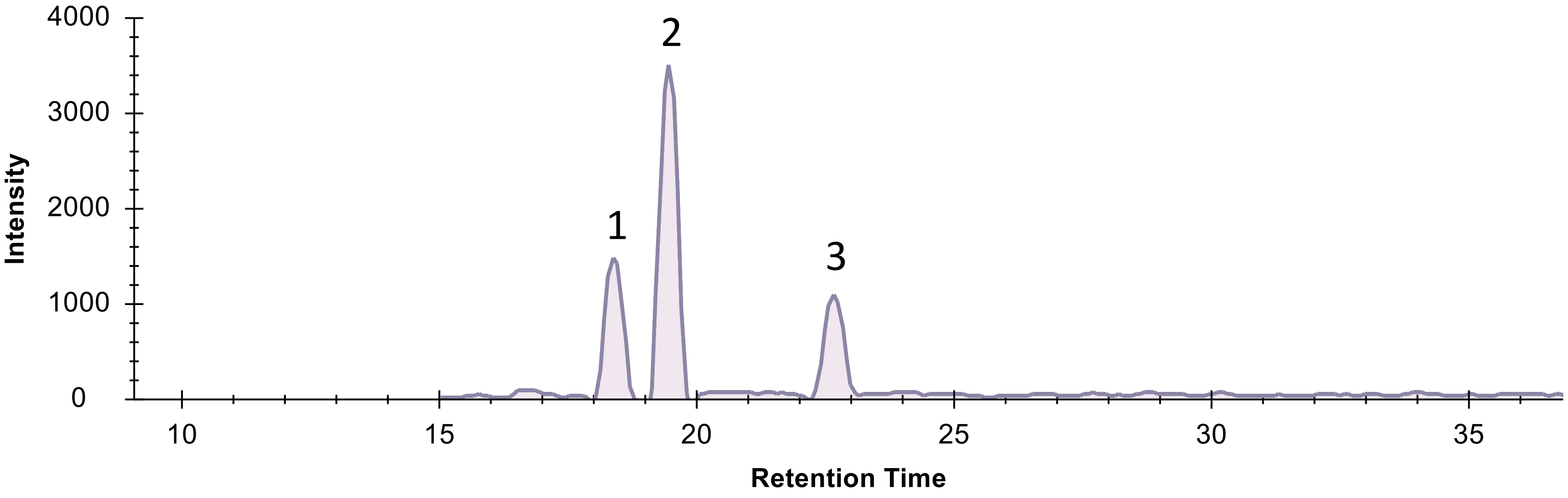

1

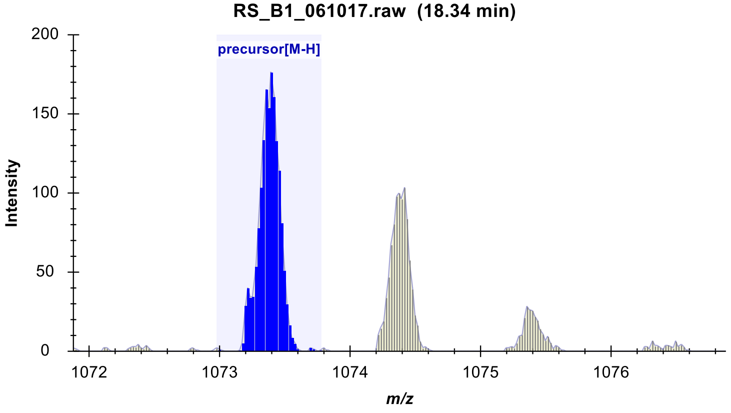

10a

2

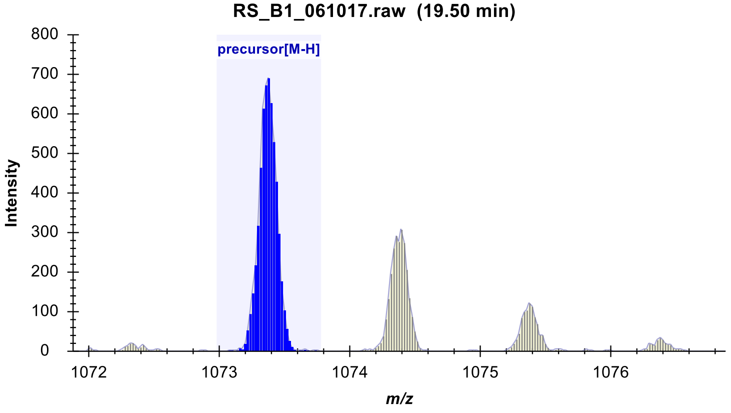

10b

3

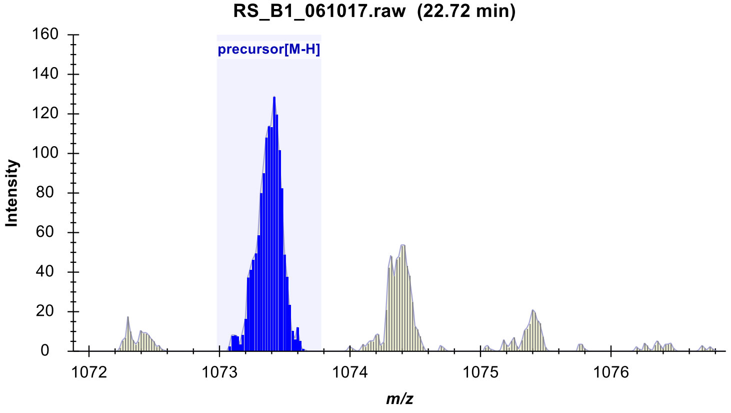

10c

GlytouCan ID: G92509FK

CID-MS/MS

(m/z 1073.37 $^{2-}$)

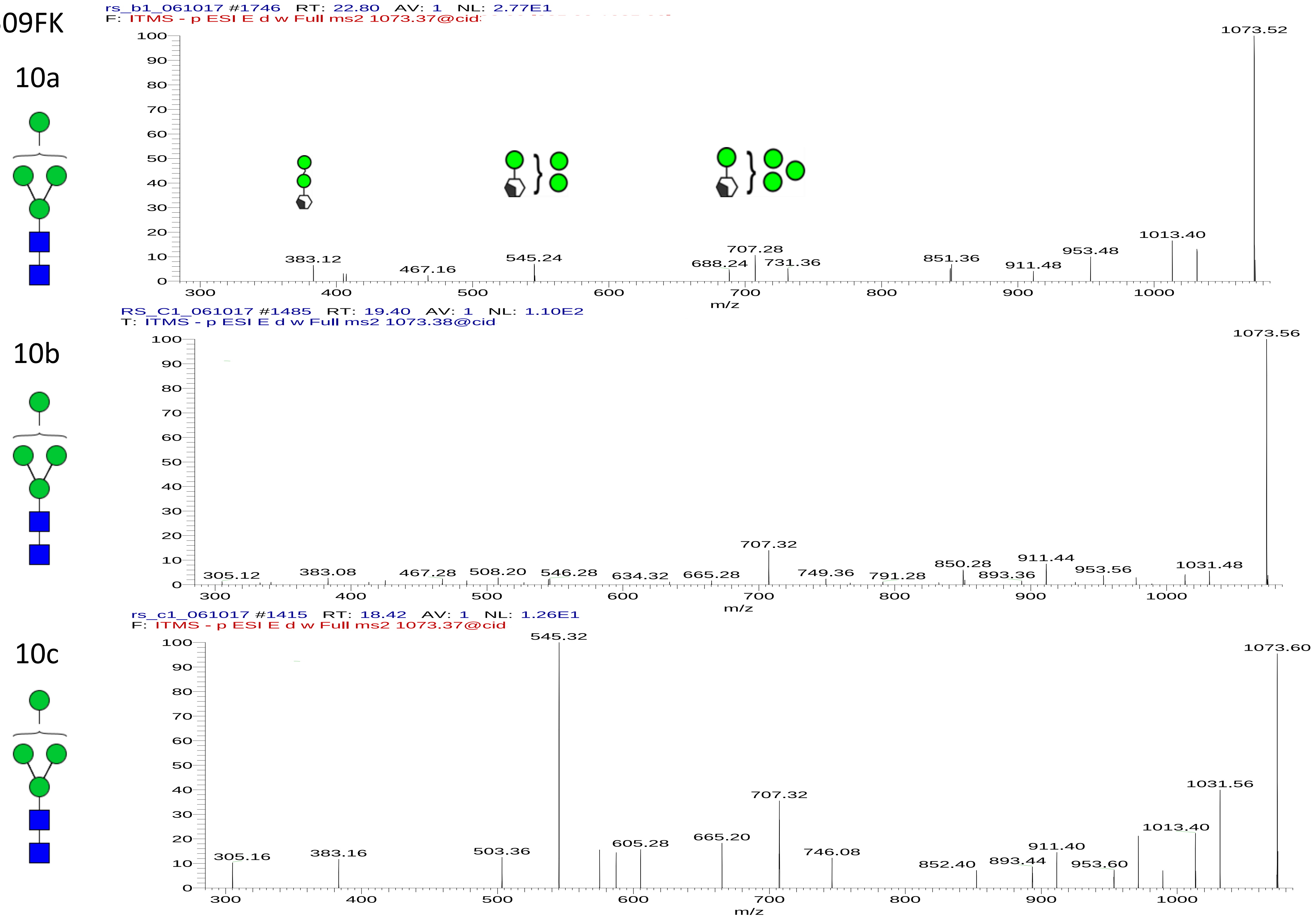

(Hex)4 + (Man)3(GlcNAc)2

GlytouCan ID: G13869TL

[M-2H]   779.27
[M-H]   1559.61

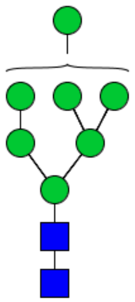

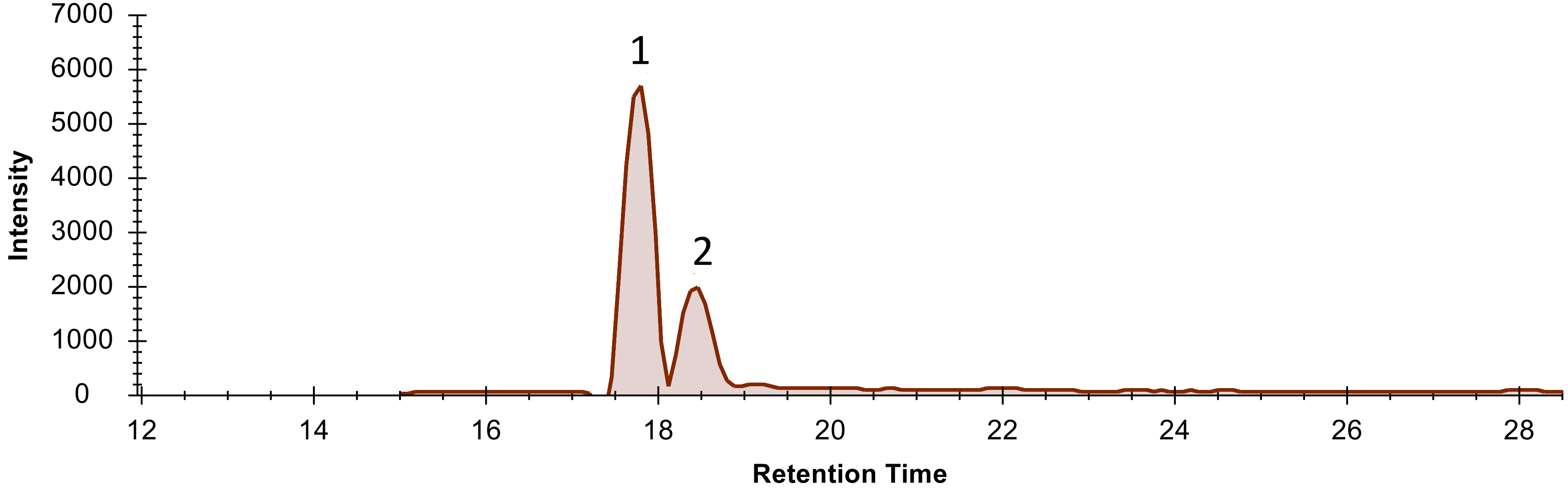

1

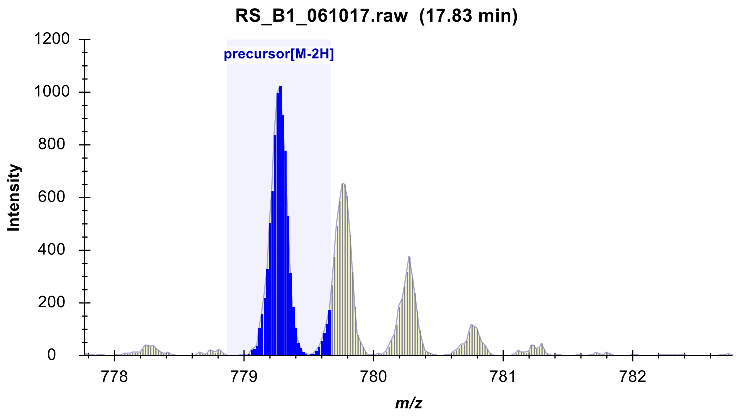

11a

2

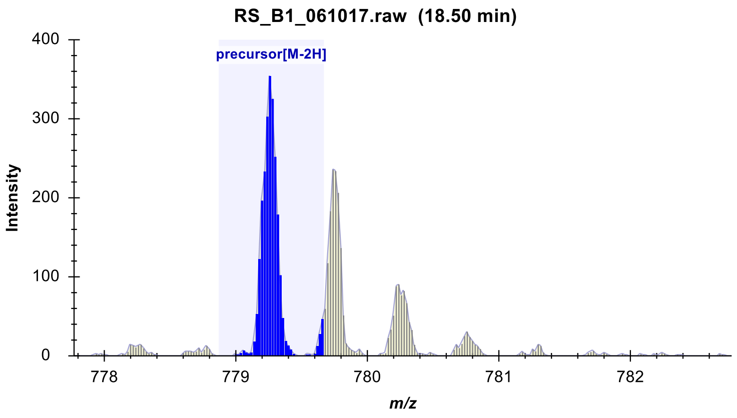

11b

GlytouCan ID: G13869TL

CID-MS/MS
(*m/z* 779.27 $^{2-}$)

11a

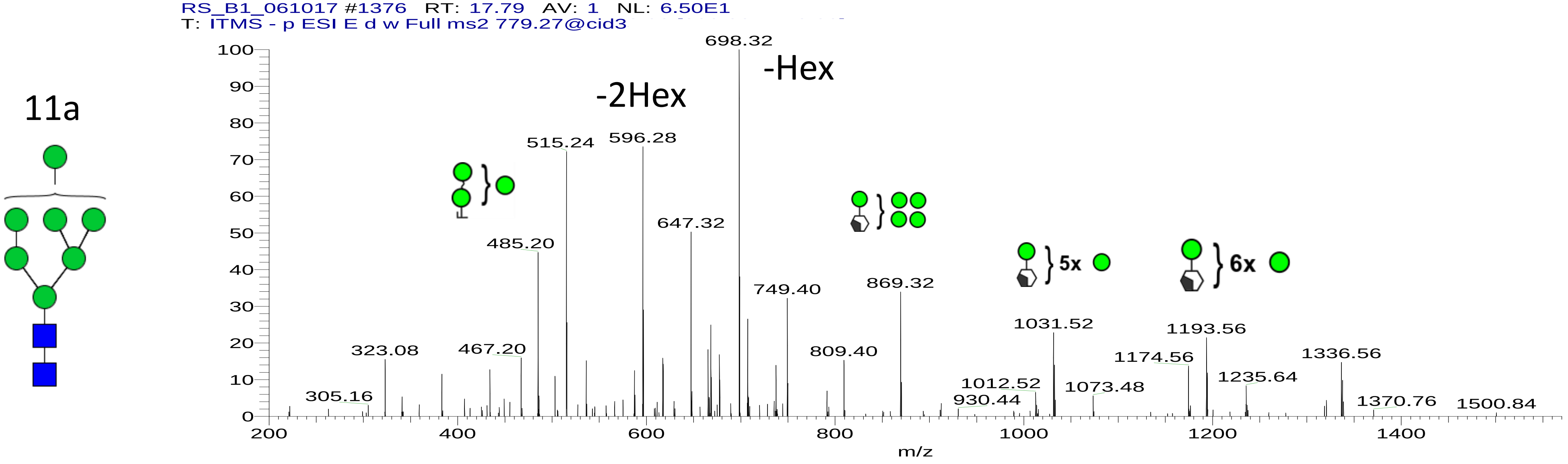

11b

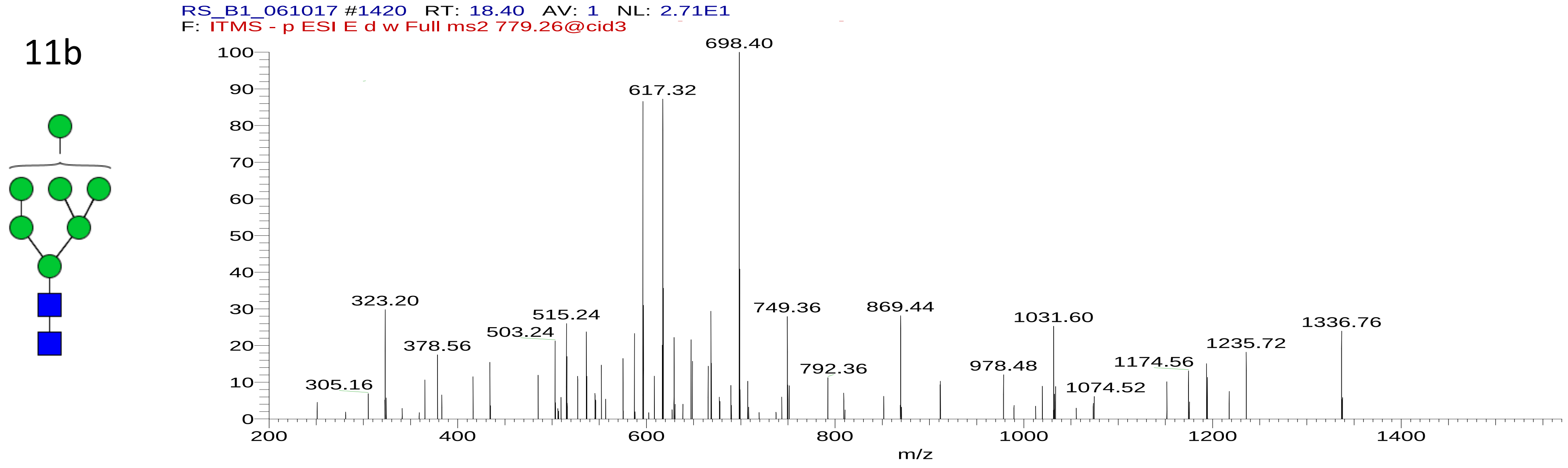

(Hex)5 + (Man)3(GlcNAc)2

GlytouCan ID: G70358QM

[M-2H]   860.29
[M-H]   1721.66

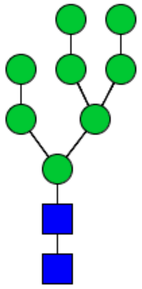

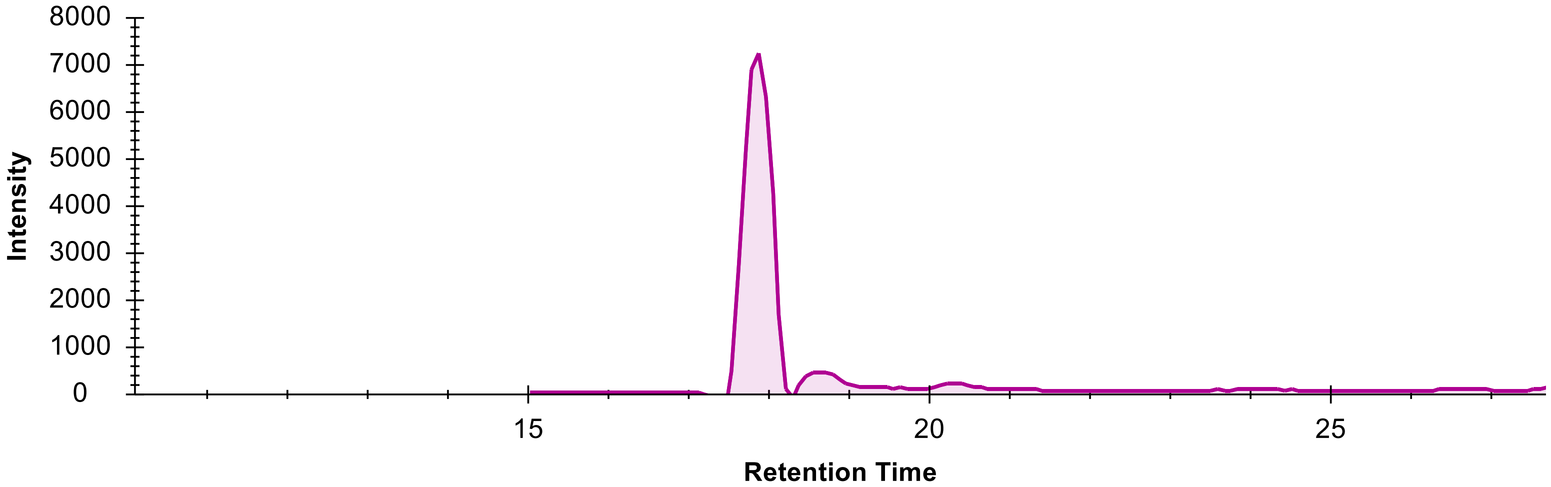

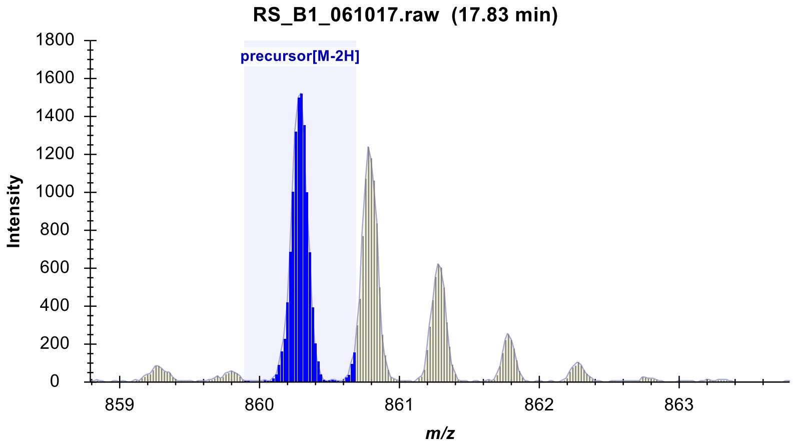

Glycan ID: 12

CID-MS/MS (m/z 860.29 $^{2-}$)

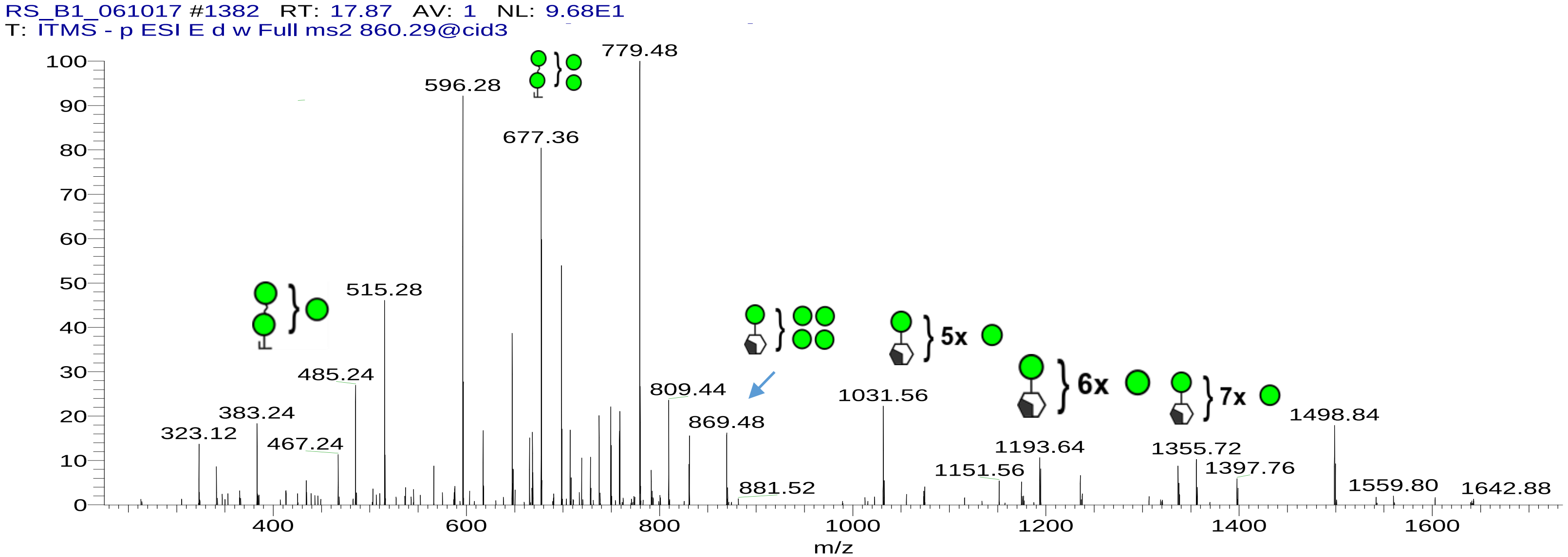

(Hex)6 + (Man)3(GlcNAc)2

GlytouCan ID: G66257KQ

[M-2H] 941.34

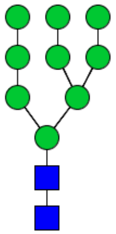

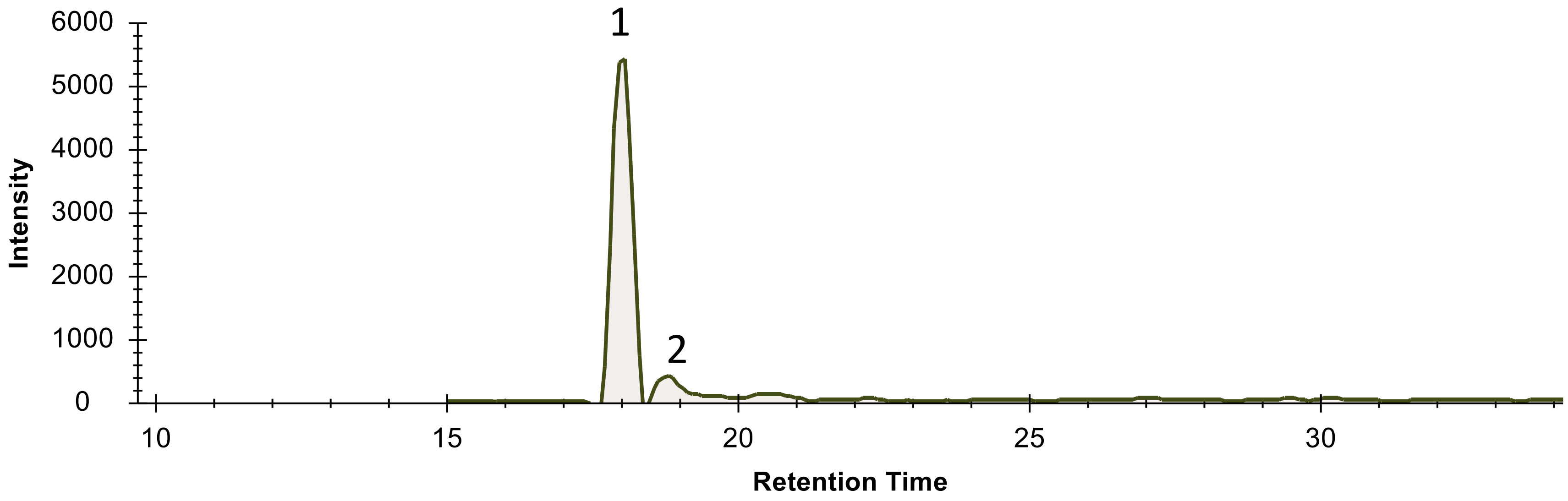

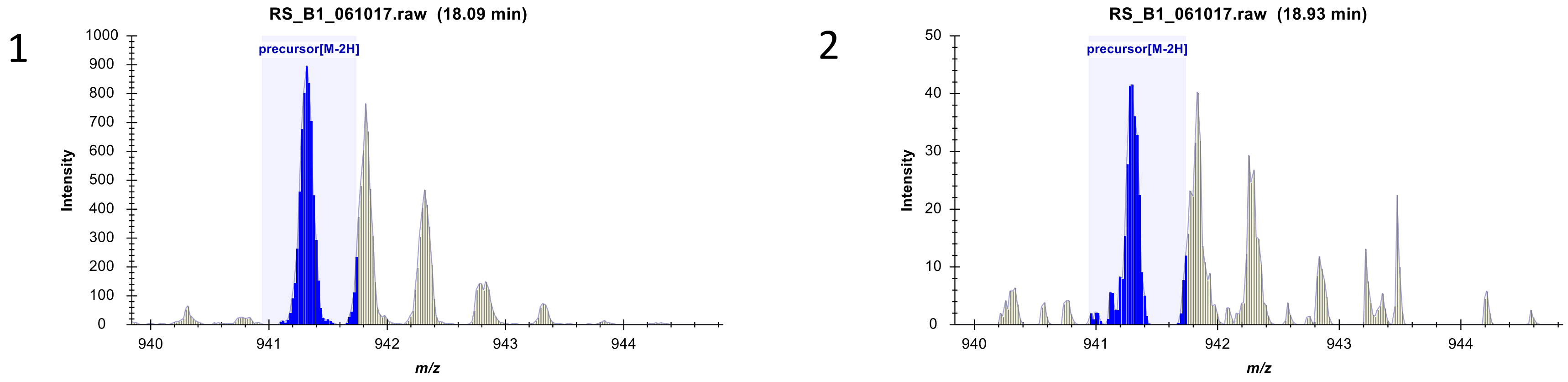

13a

13b

GlytouCan ID: G66257KQ

CID-MS/MS
(m/z 941.34 $^{2-}$)

13a

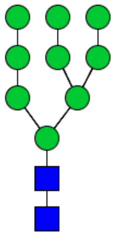

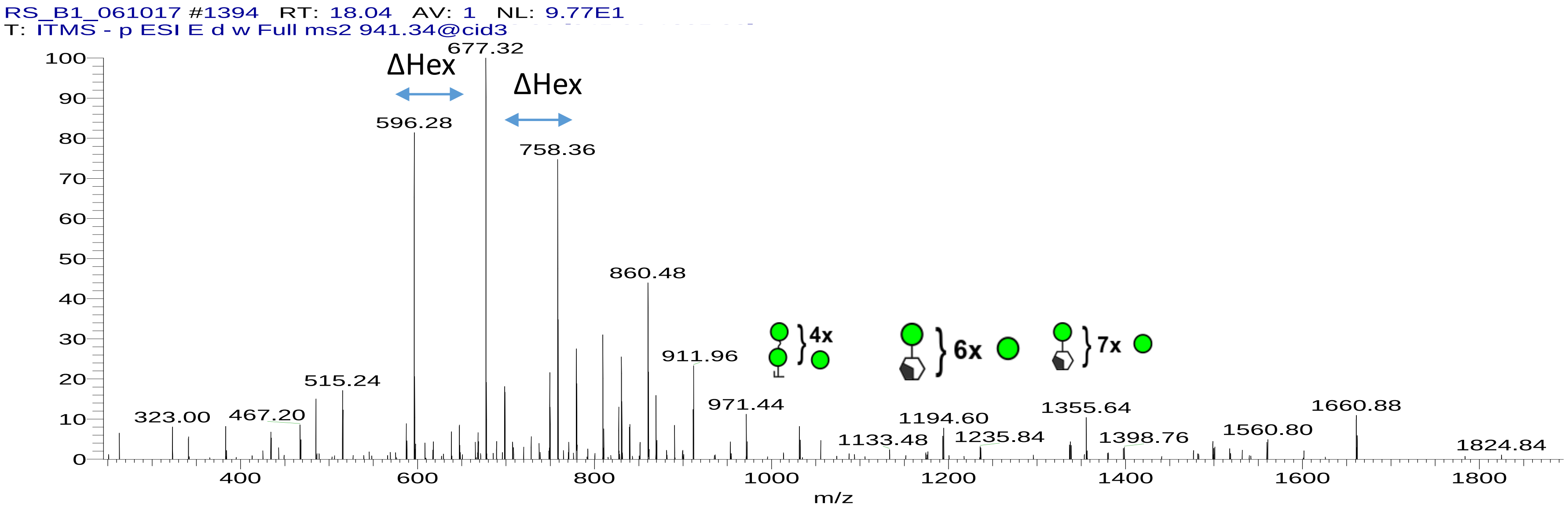

13b

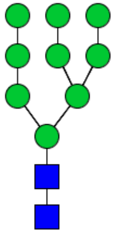

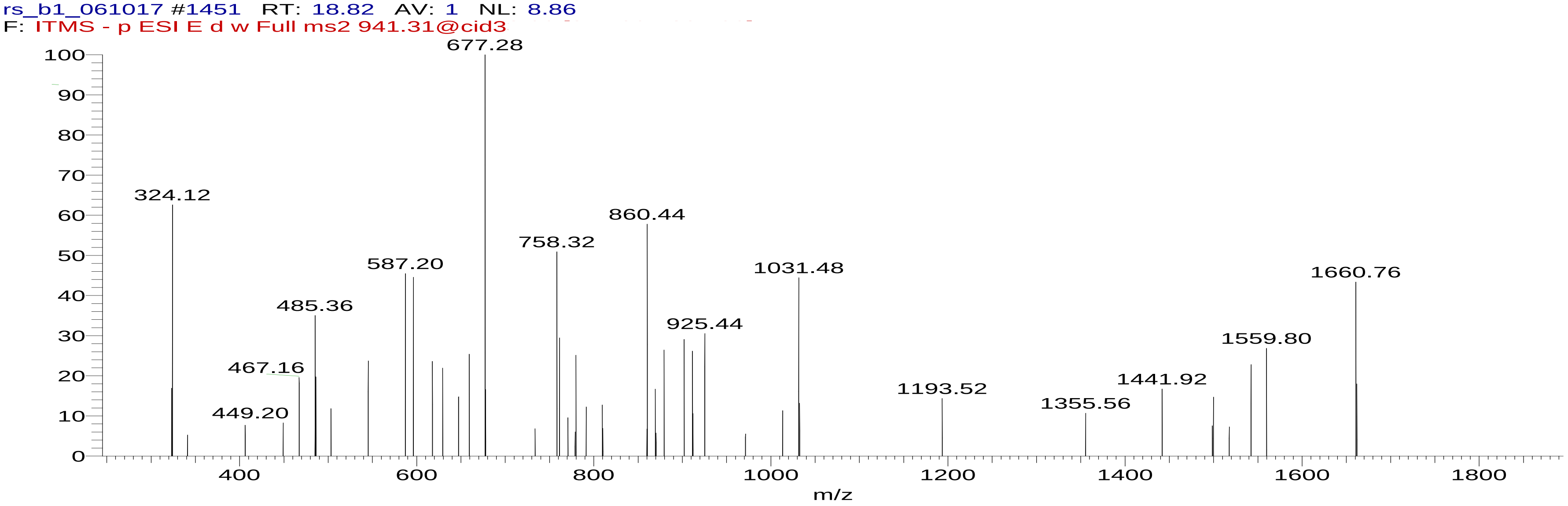

(Hex)7 + (Man)3(GlcNAc)2

GlytouCan ID: G19763QB

[M-2H] 1022.35

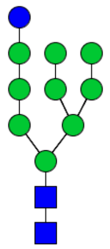

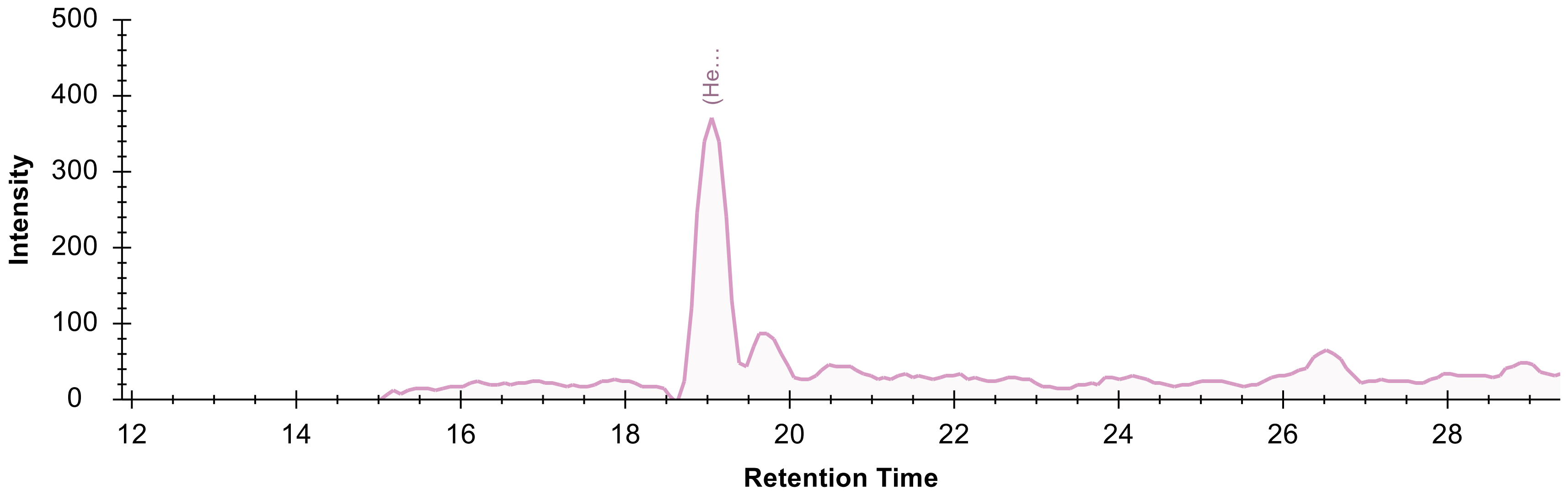

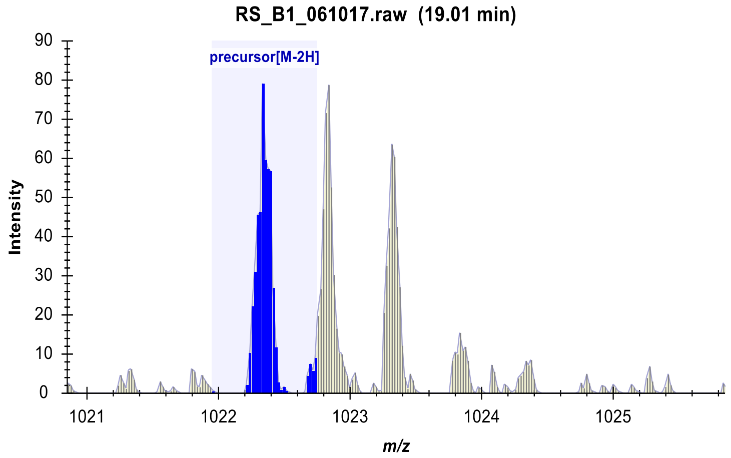

Glycan ID: 14

CID-MS/MS (m/z 1022.34 $^{2-}$)

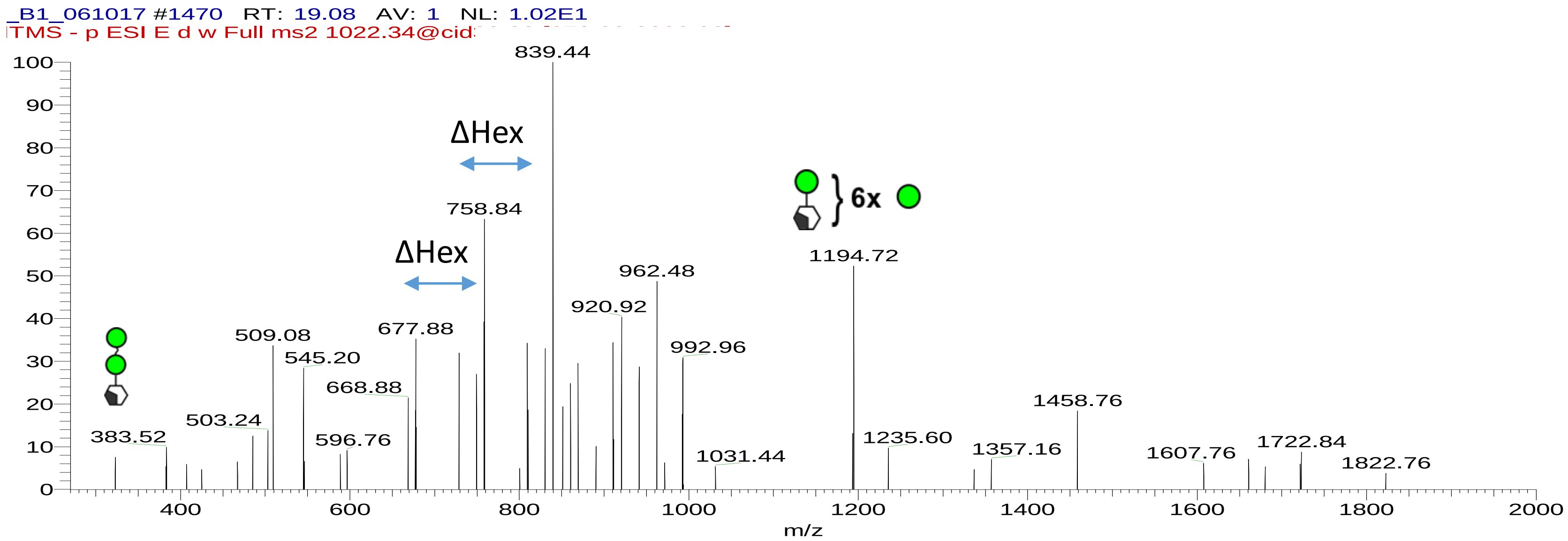

(HexNAc)1 + (Man)3(GlcNAc)2

No GlytouCan ID

[M-H] 1114.39

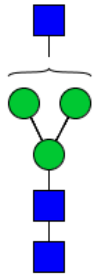

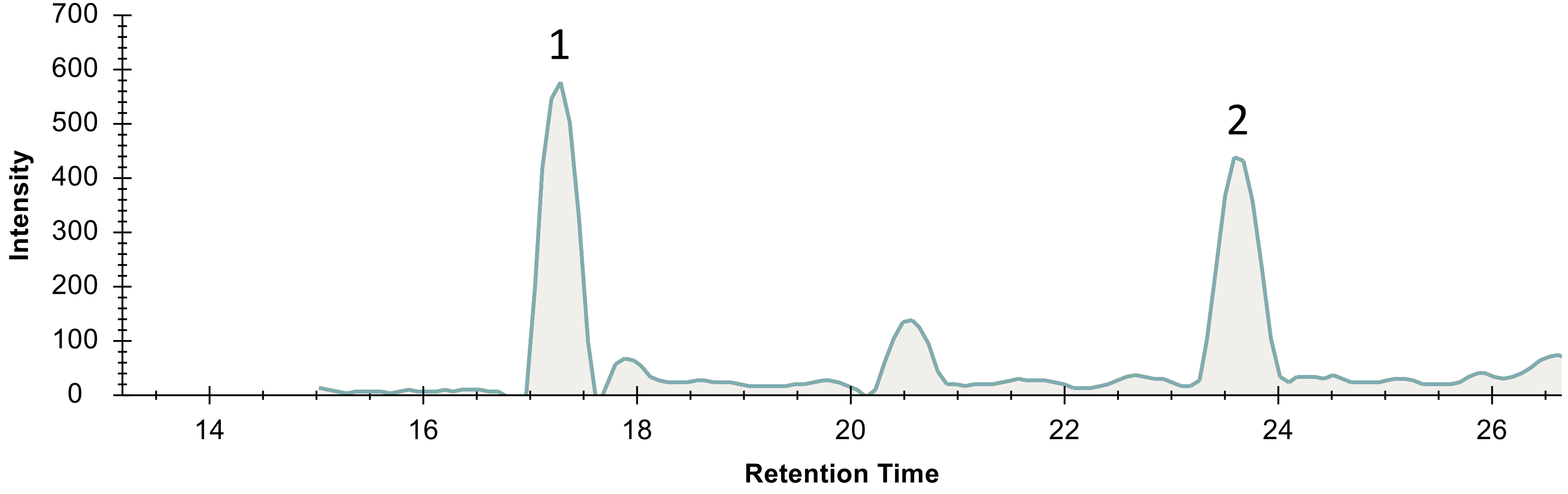

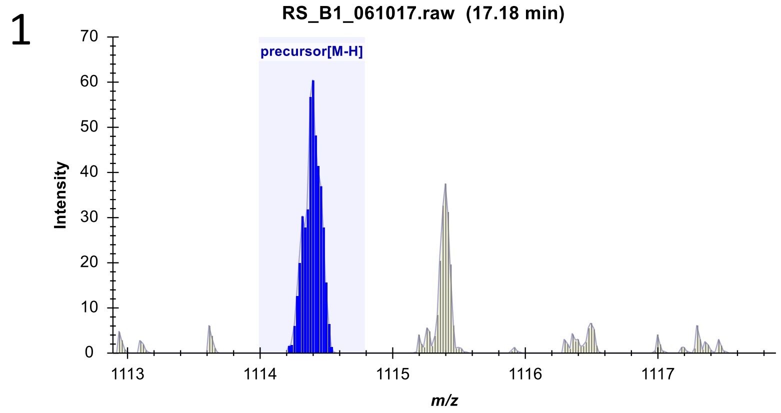

15a

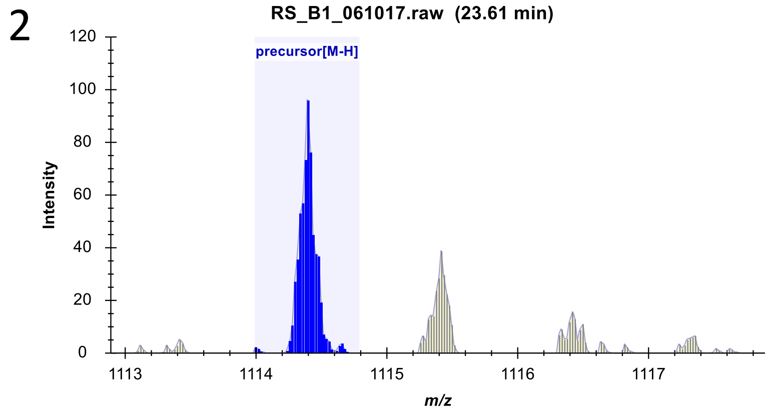

15b

CID-MS/MS
(*m/z* 1114.40 $^{1-}$)

15a

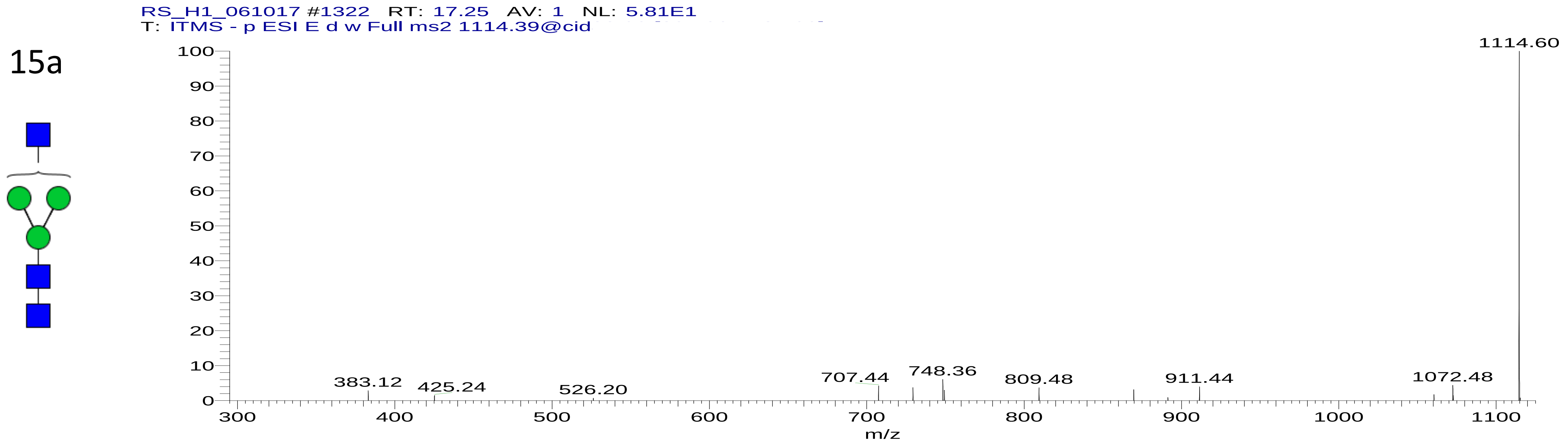

15b

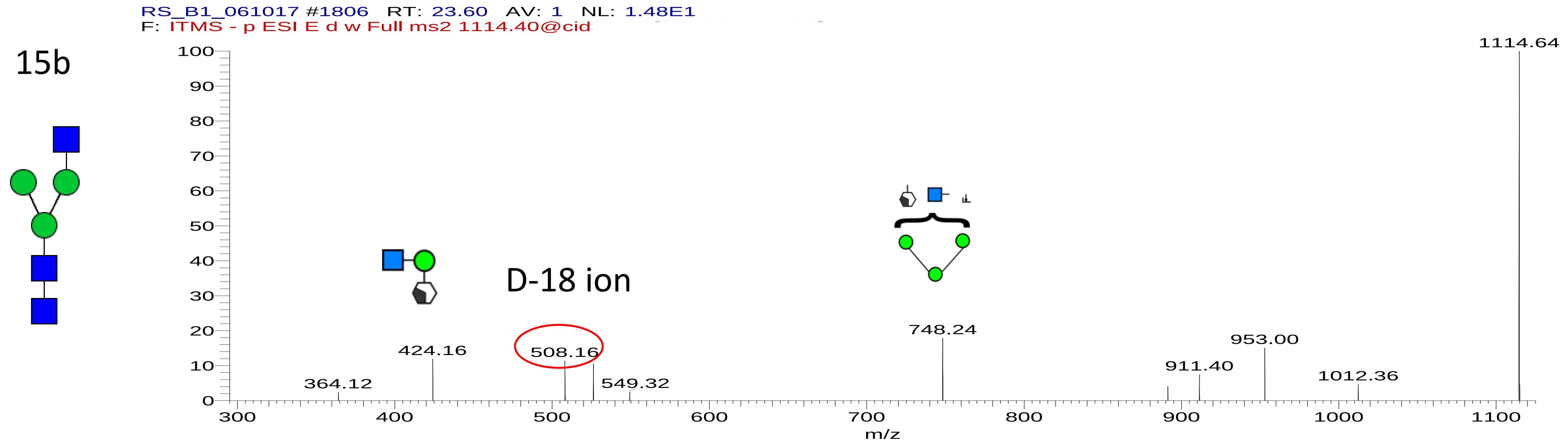

(HexNAc)1 (Deoxyhexose)1 + (Man)3(GlcNAc)2

GlytouCan ID: G27314YB

[M-H] 1260.48

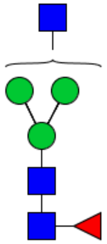

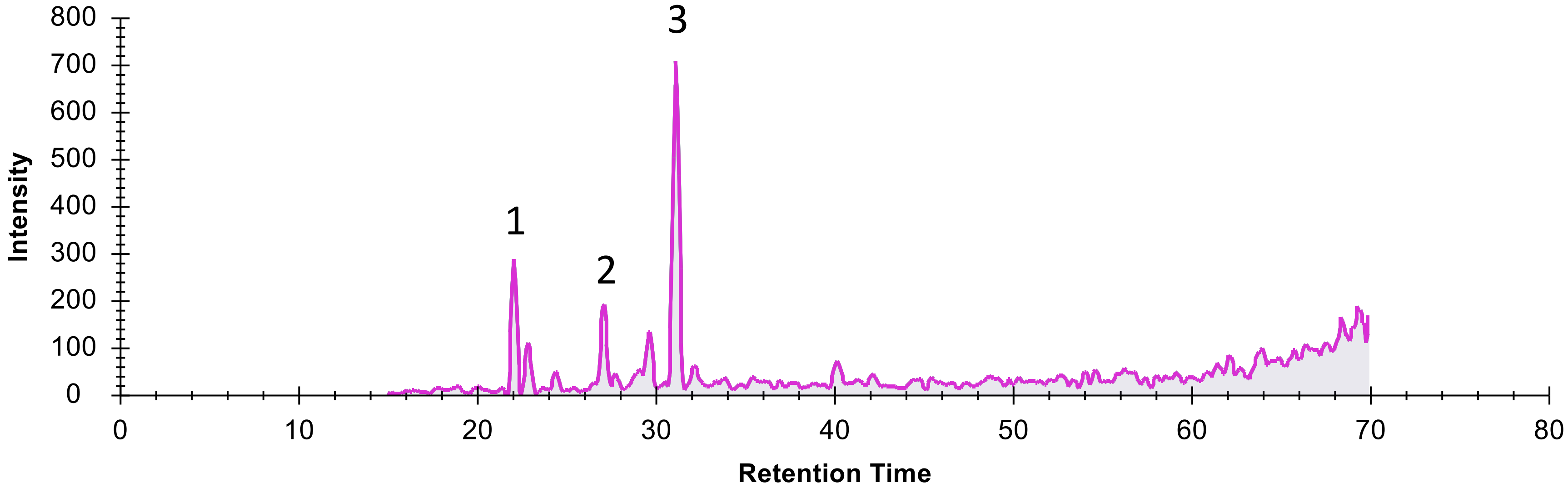

1

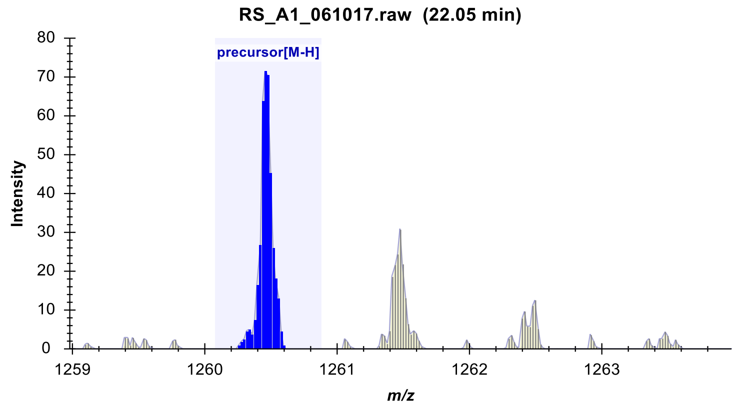

16a

2

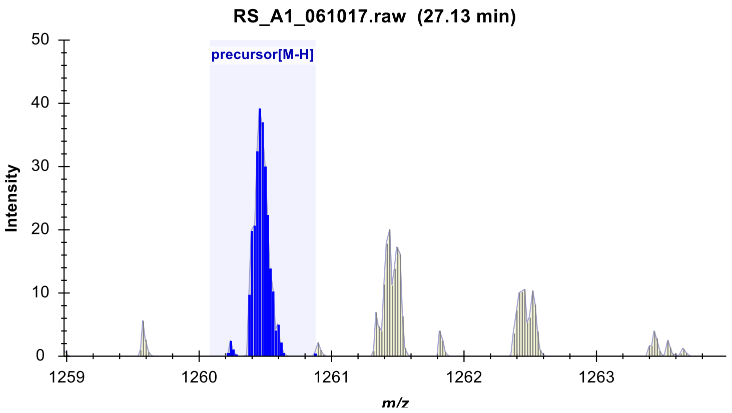

16b

3

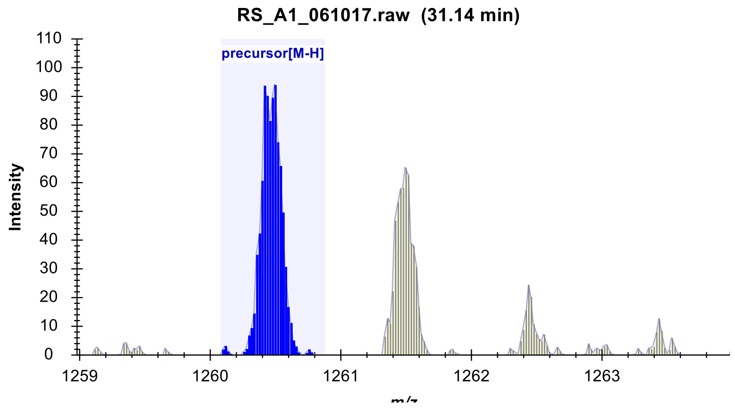

16c

CID-MS/MS
(m/z 1260.46 [1-])

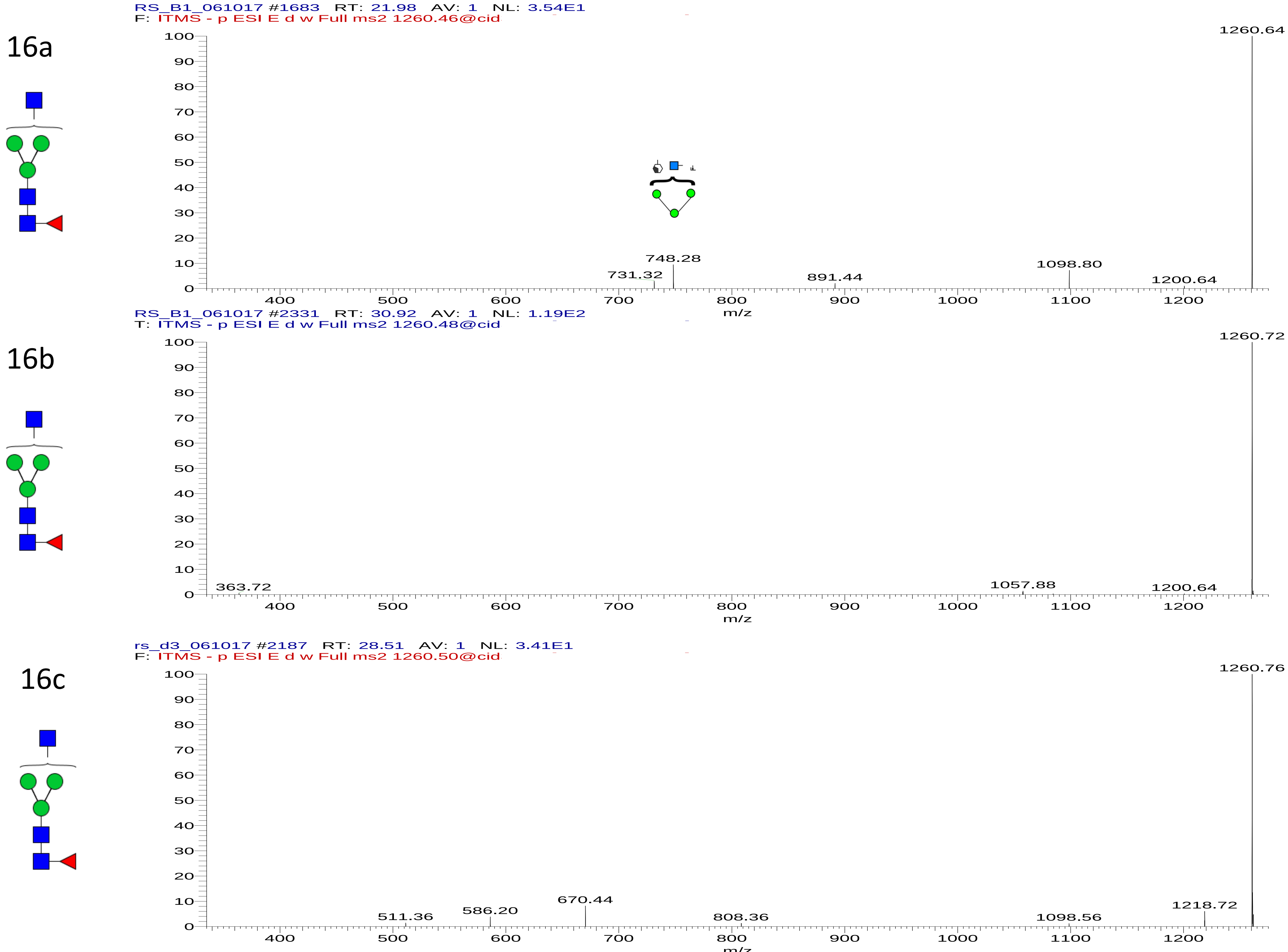

(Hex)1 (HexNAc)1 + (Man)3(GlcNAc)2
GlytouCan ID: G86309VR
[M-2H] 637.71
[M-H]1276.49

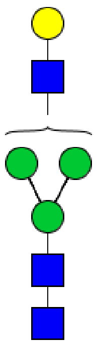

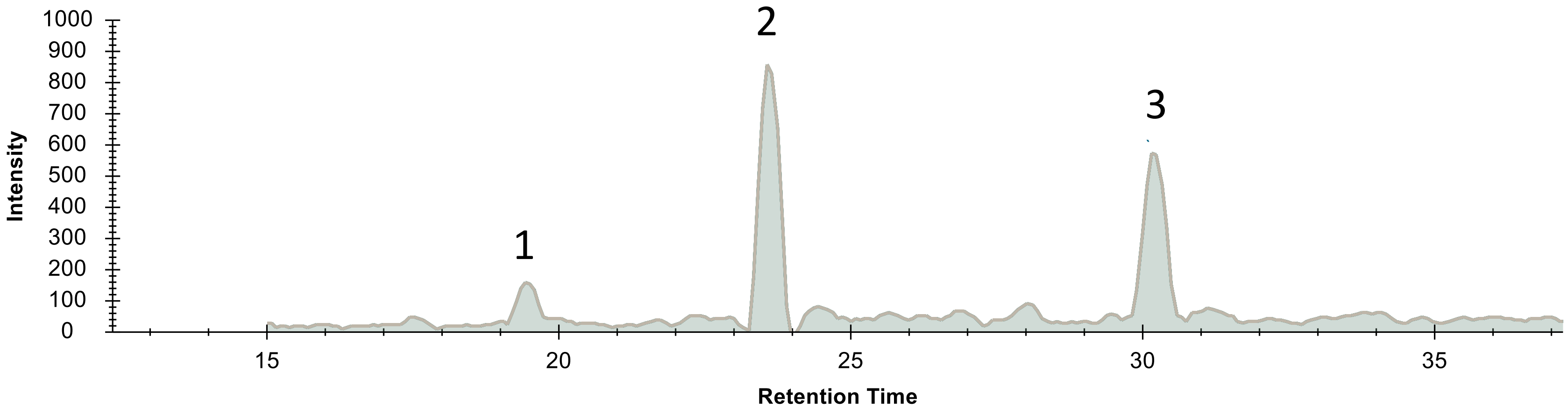

1

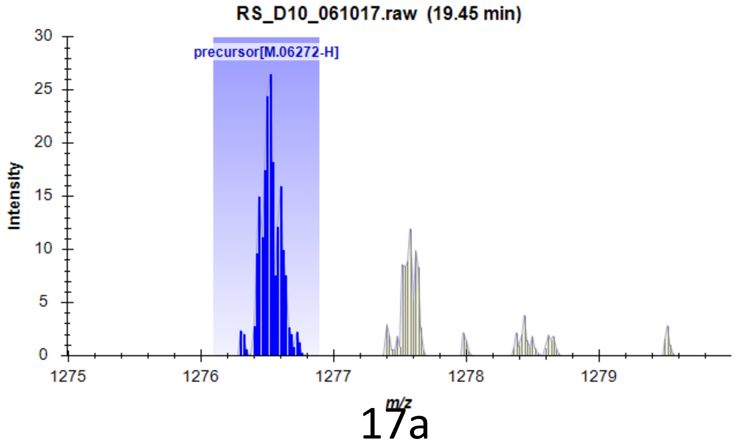

17a

2

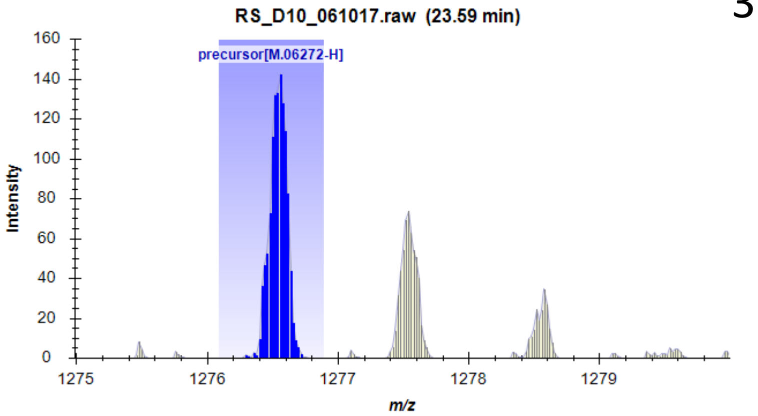

17b

3

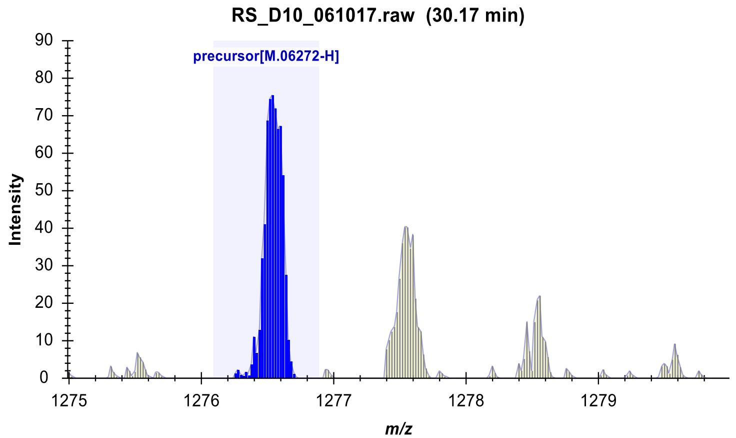

17c

GlytouCan ID: G86309VR

CID-MS/MS (m/z 1276.49 $^{1-}$)

17b

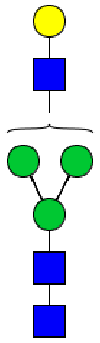

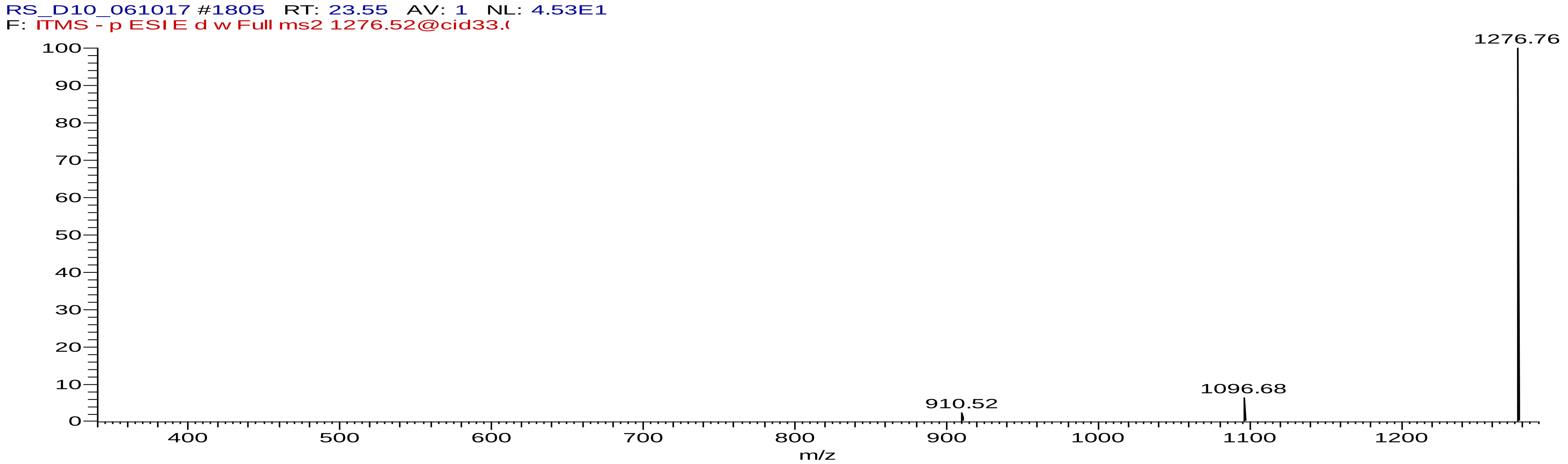

17c

3

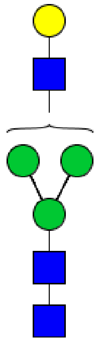

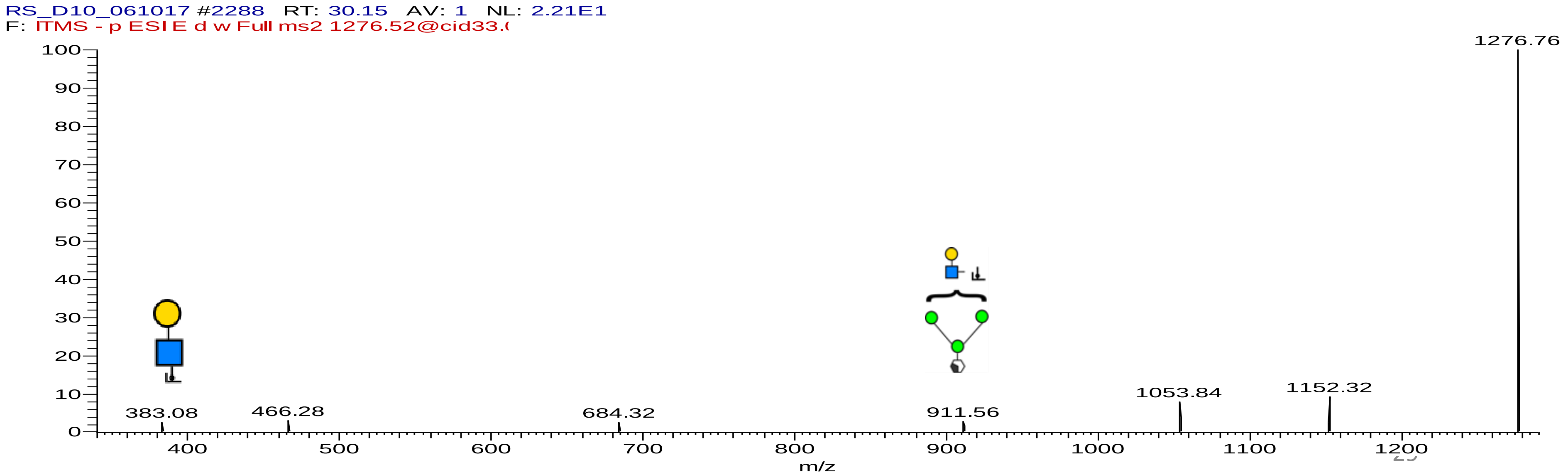

(HexNAc)2 + (Man)3(GlcNAc)2

GlytouCan ID: G38426SE

[M-2H] 658.25

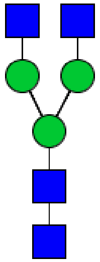

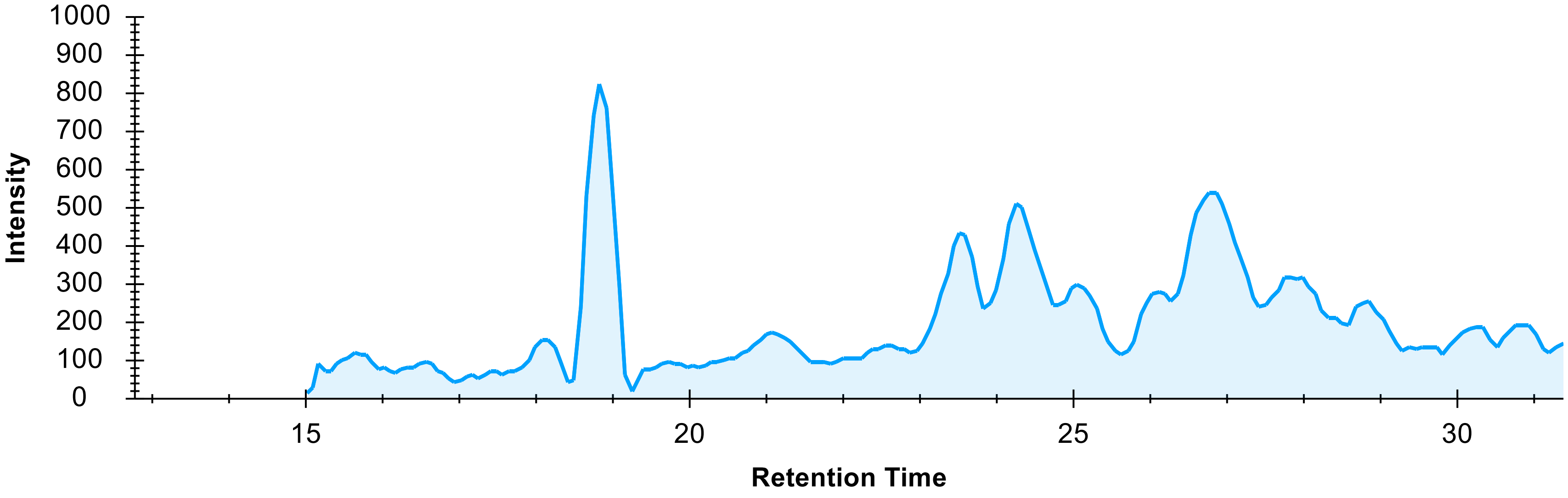

CID-MS/MS (m/z 658.25 $^{2-}$)

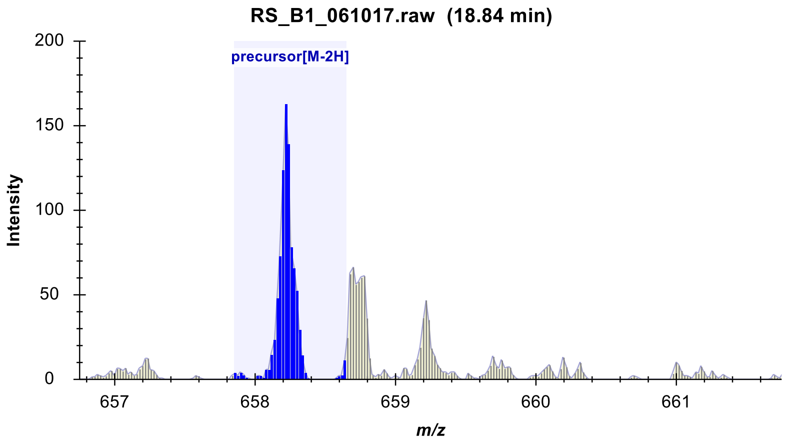

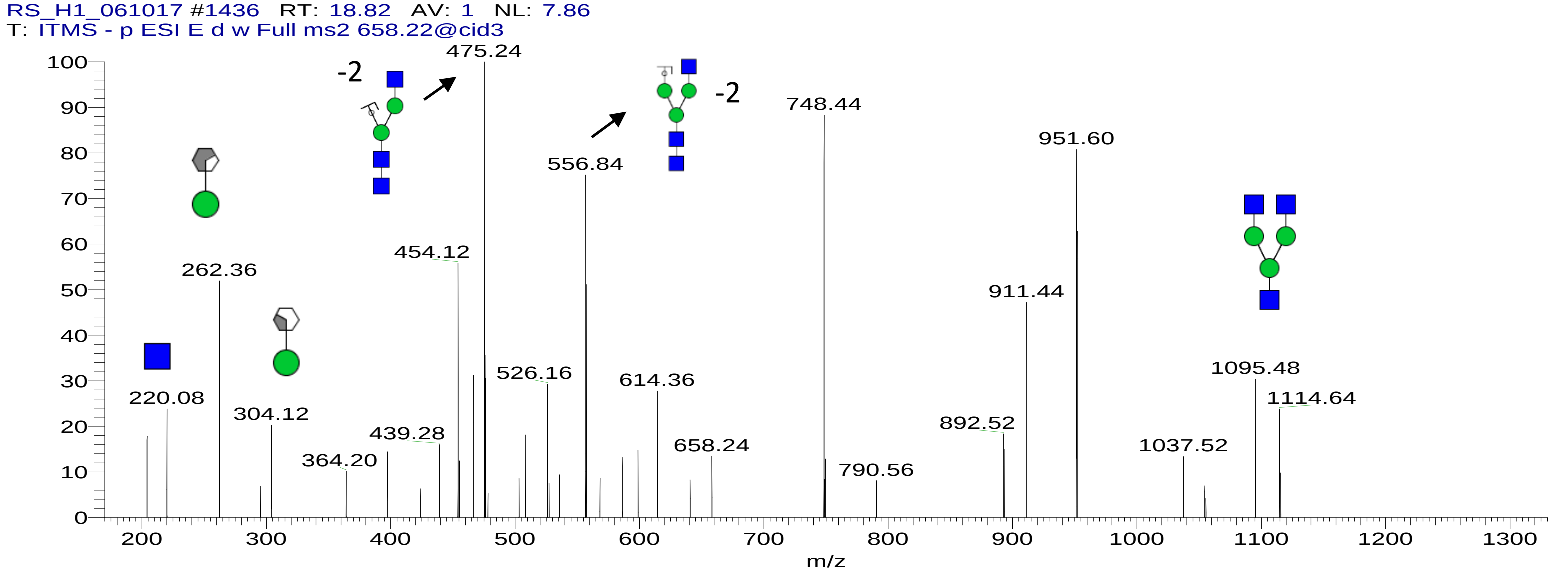

Glycan ID: 18

(Hex)1 (HexNAc)1 (Deoxyhexose)1 + (Man)3(GlcNAc)2_1

[M-2H] 710.74

[M-H] 1422.55

GlytouCan ID: G17800QD

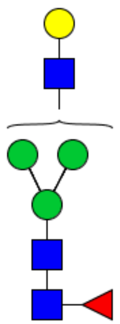

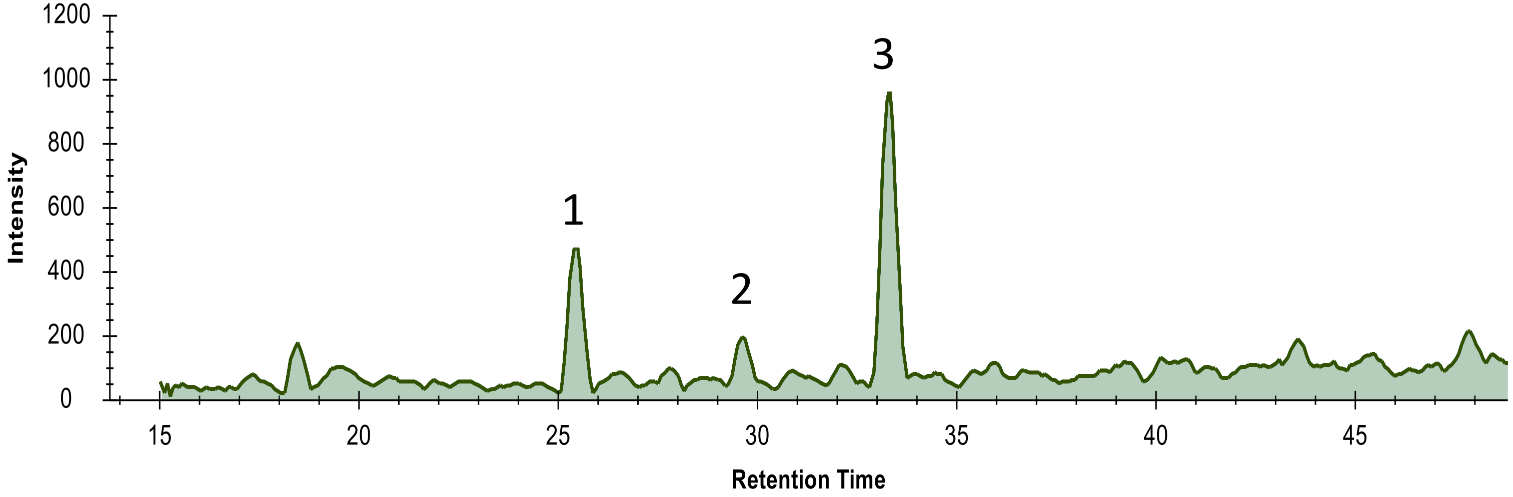

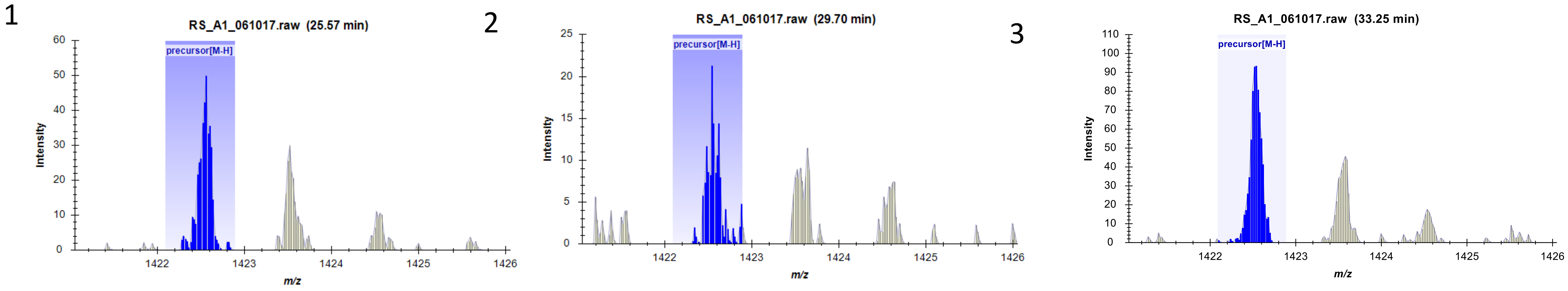

19a 19b 19c

GlytouCan ID: : G17800QD

CID-MS/MS
(*m/z* 710.74 $^{2-}$)

19a

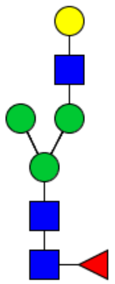

1

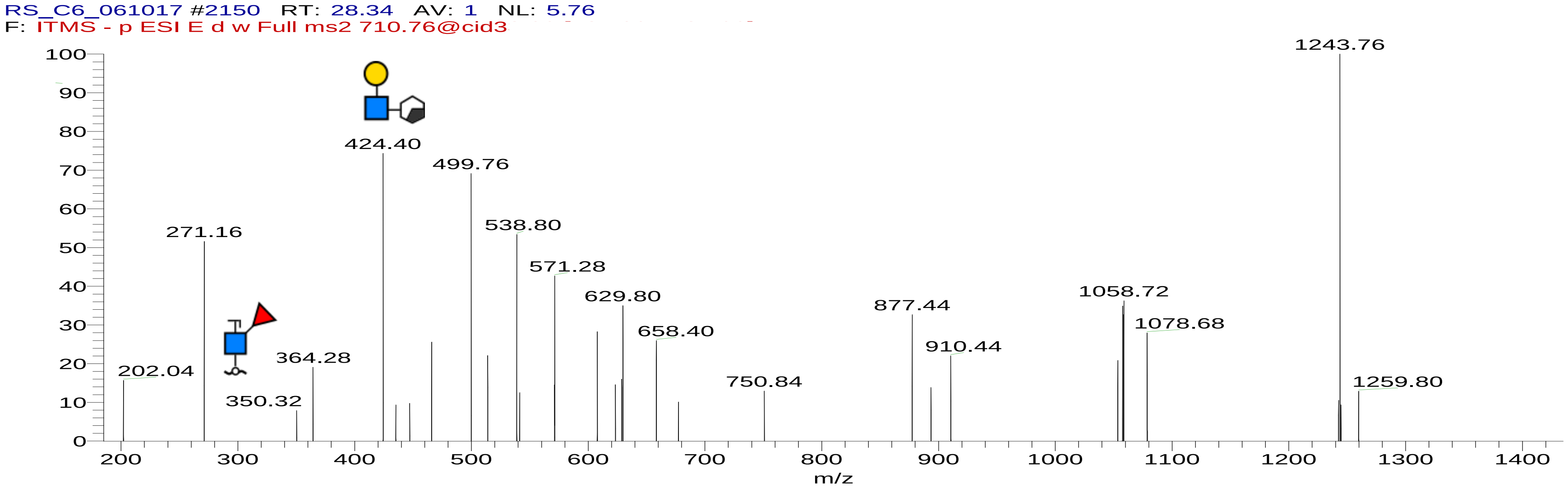

3

19c

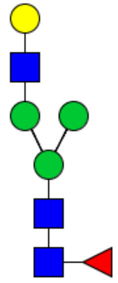

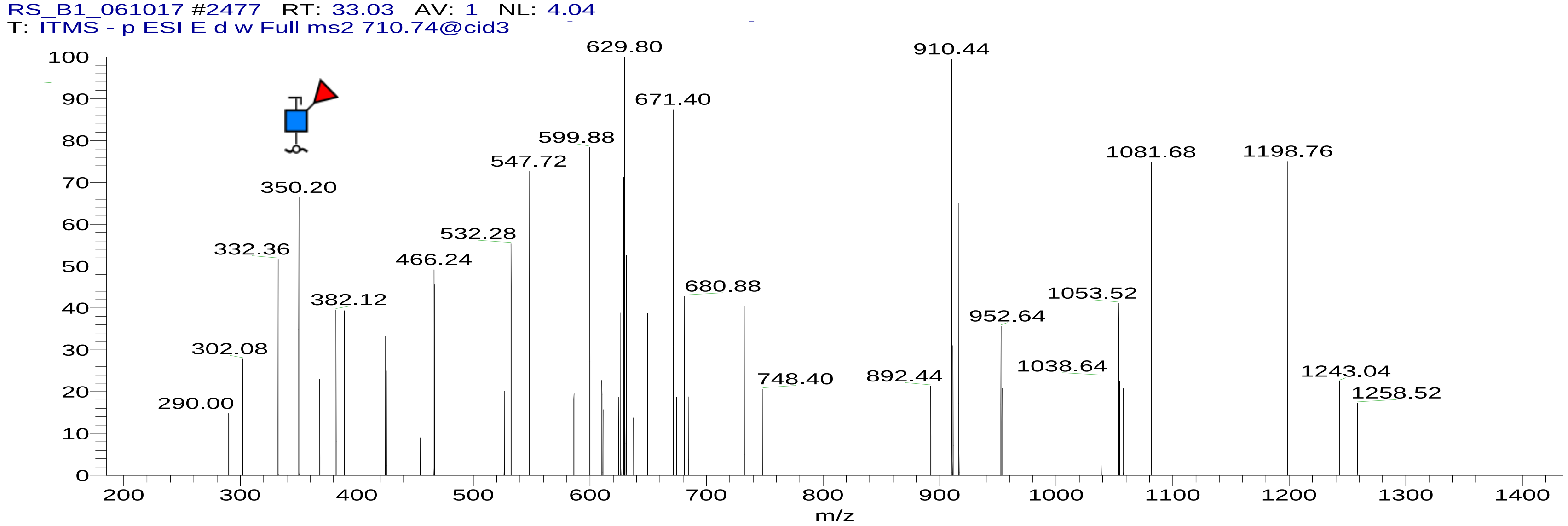

(Hex)2 (HexNAc)1 + (Man)3(GlcNAc)2
GlytouCan ID: G41072QC
[M-2H] 718.75

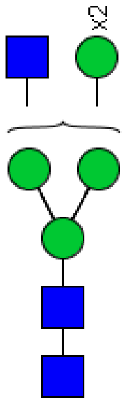

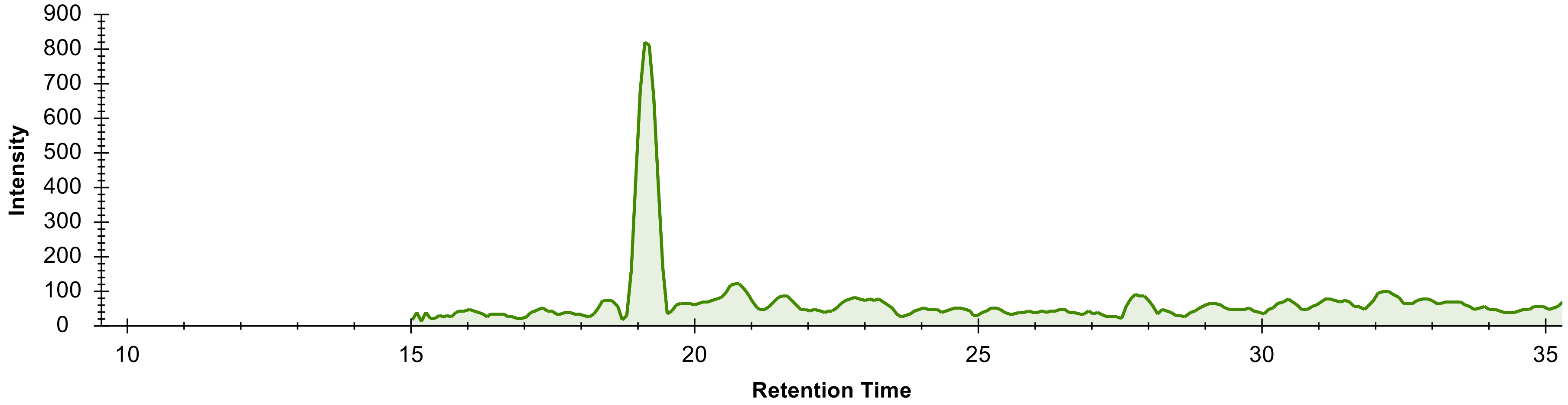

CID-MS/MS (m/z 718.75 $^{2-}$)

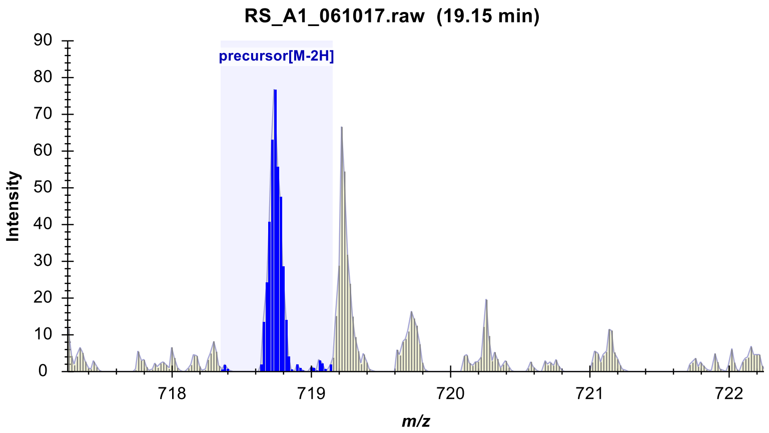

Glycan ID: 20

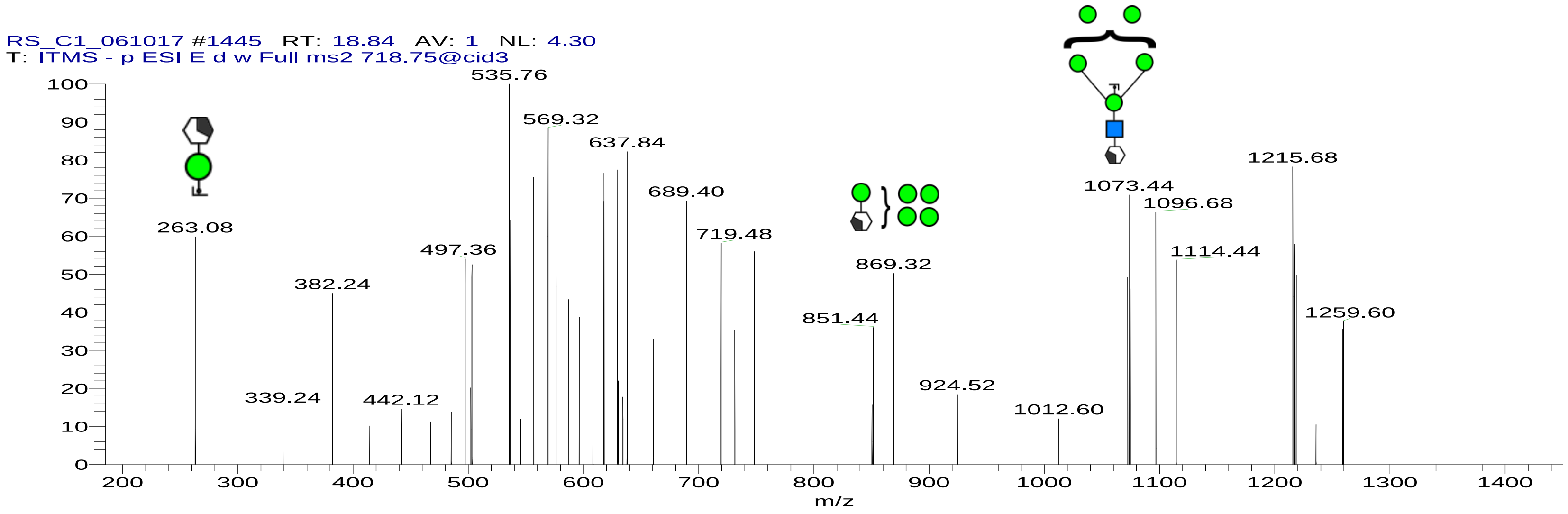

(HexNAc)2 (Deoxyhexose)1 + (Man)3(GlcNAc)2

GlytouCan ID: G84053BQ

[M-2H] 731.26

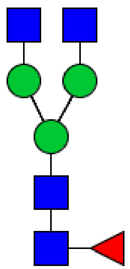

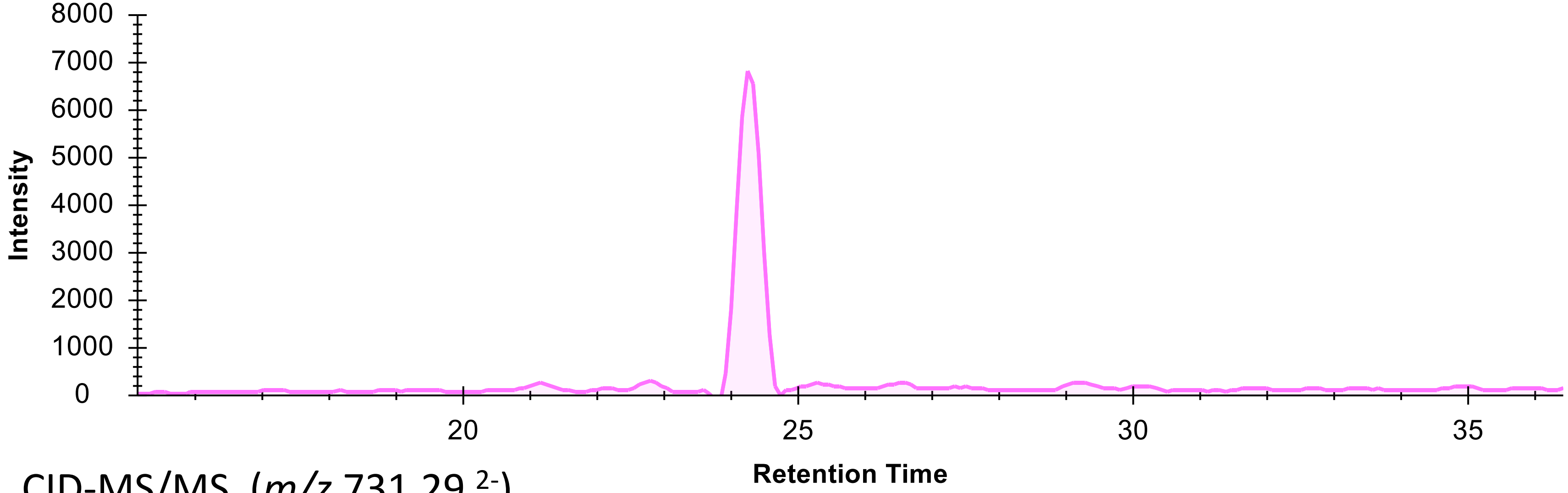

CID-MS/MS (m/z 731.29 $^{2-}$)

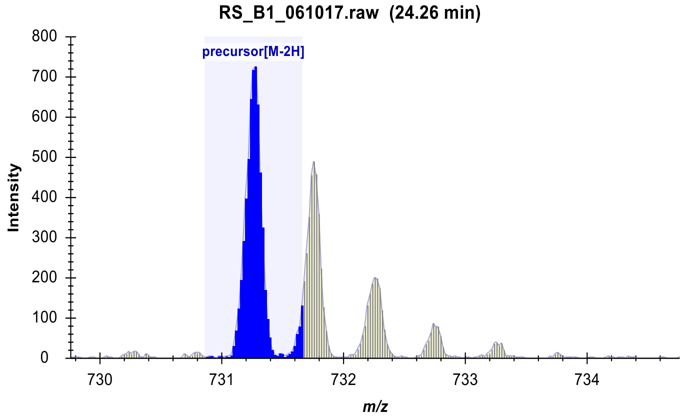

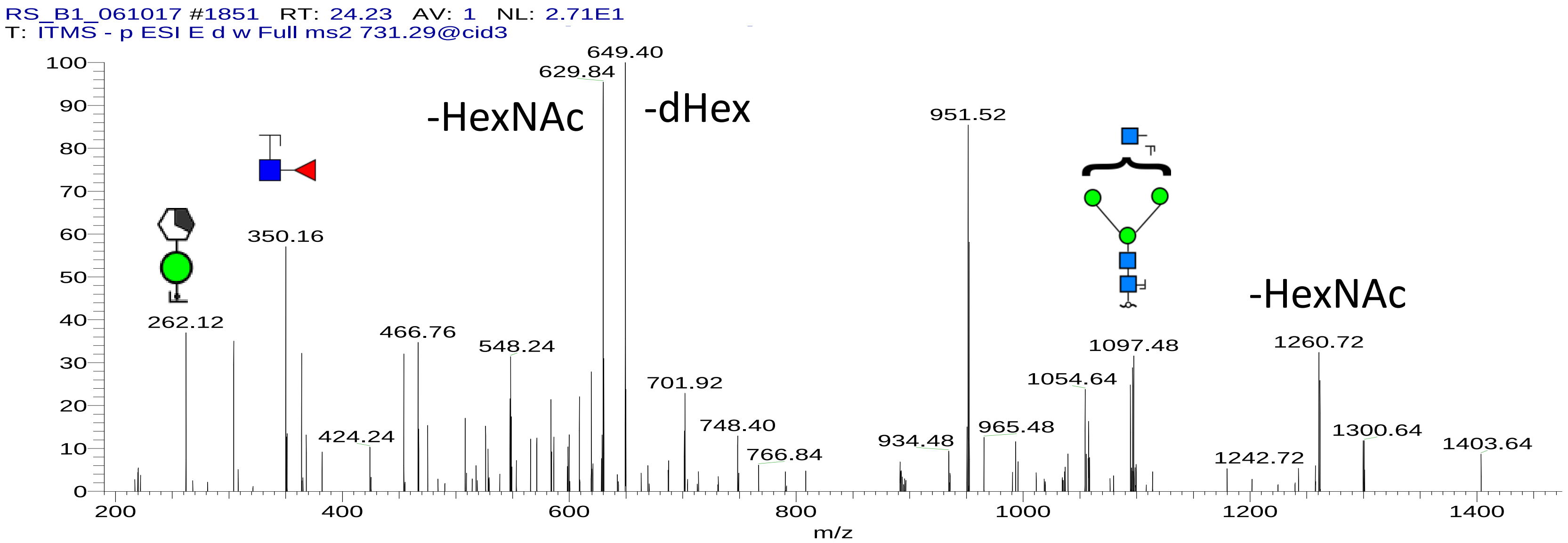

Glycan ID: 21

(Hex)1 (HexNAc)2 + (Man)3(GlcNAc)2
GlytouCan ID: G06550JR
[M-2H] 739.26

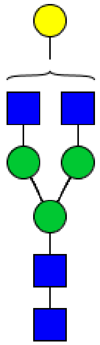

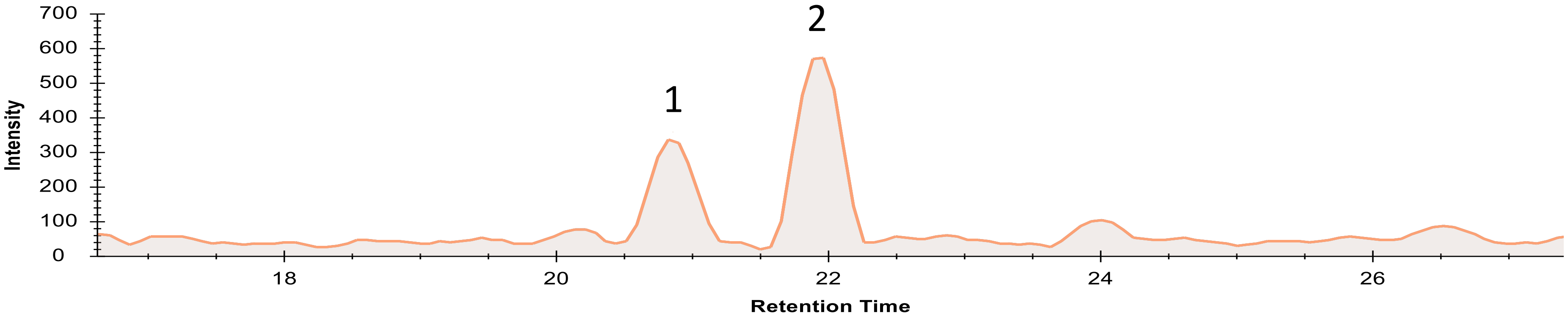

1

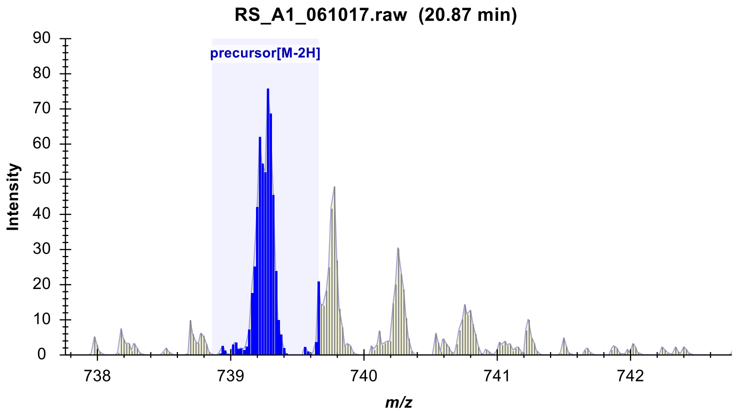

2

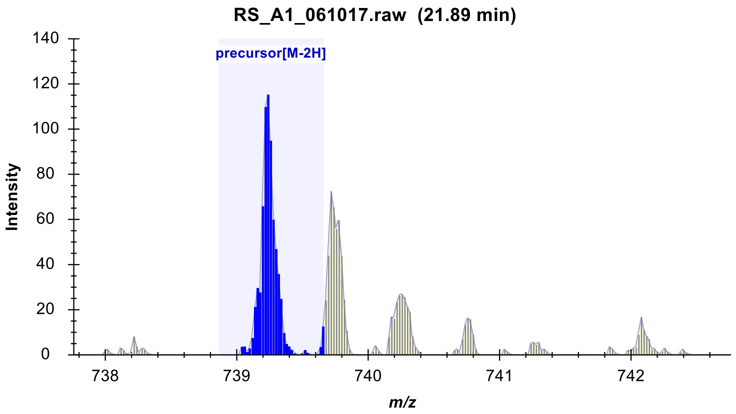

22a

22b

GlytouCan ID: G06550JR

CID-MS/MS
(*m/z* 739.22 $^{2-}$)

22a

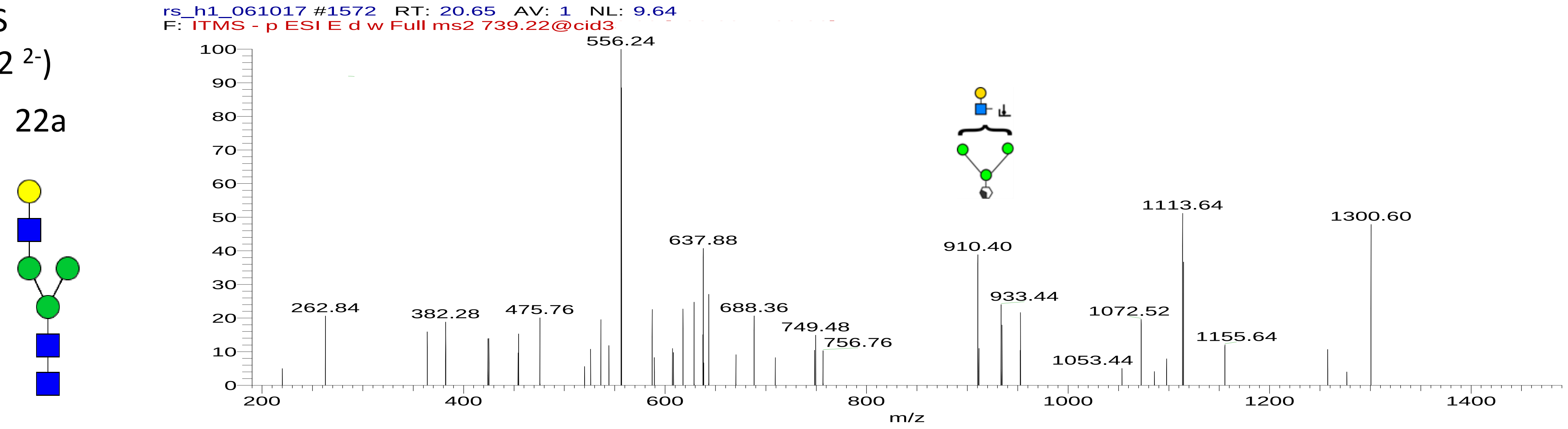

22b

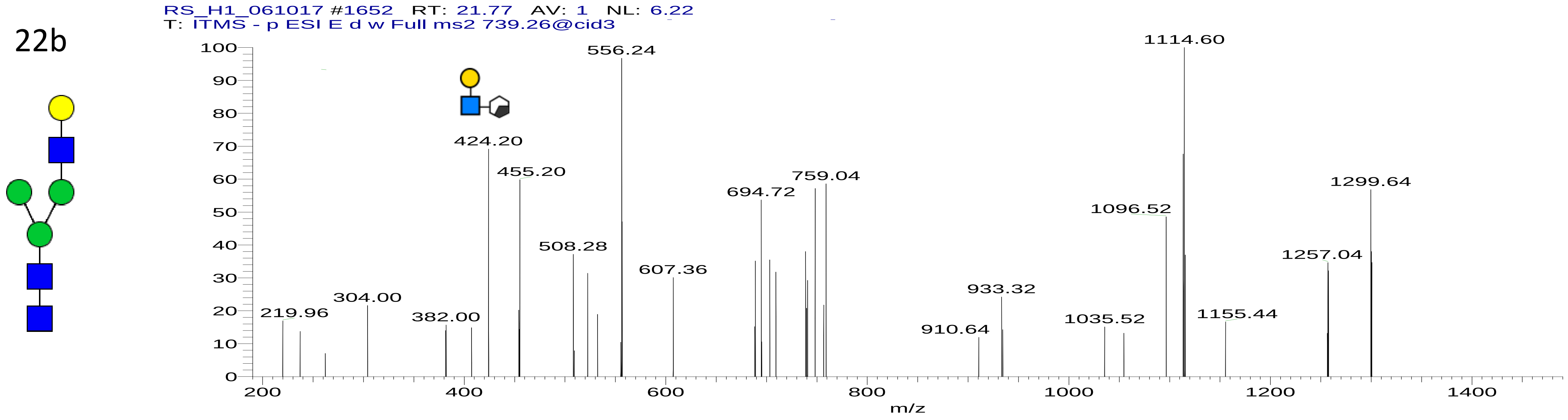

(Hex)1 (HexNAc)1 (NeuAc)1 + (Man)3(GlcNAc)2
[M-2H] 783.28
GlytouCan ID: G75448JJ

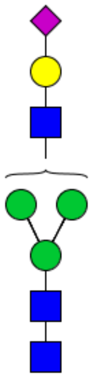

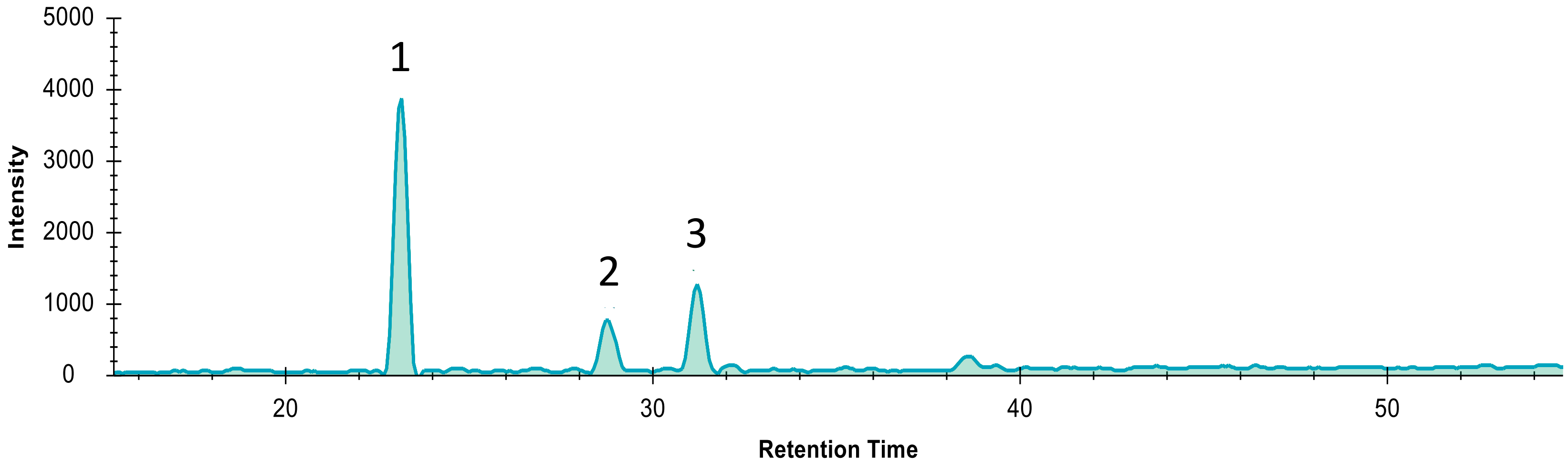

1

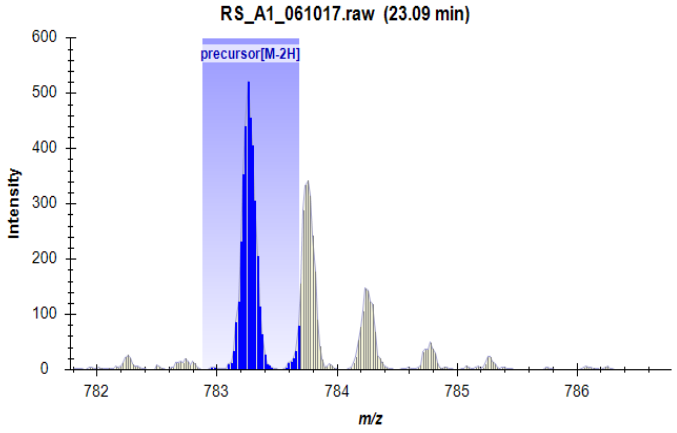

23a

2

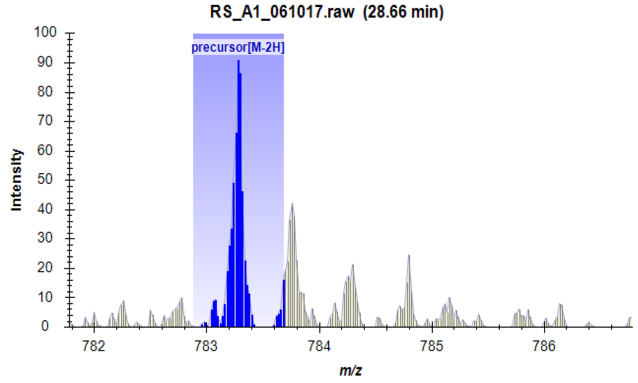

23b

3

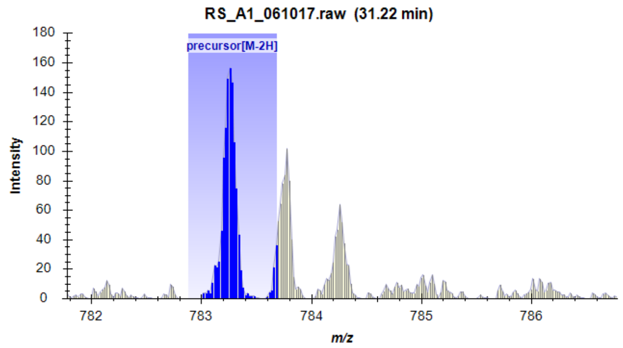

23c

GlytouCan ID: G75448JJ

CID-MS/MS

(m/z 783.28 $^{2-}$)

23a

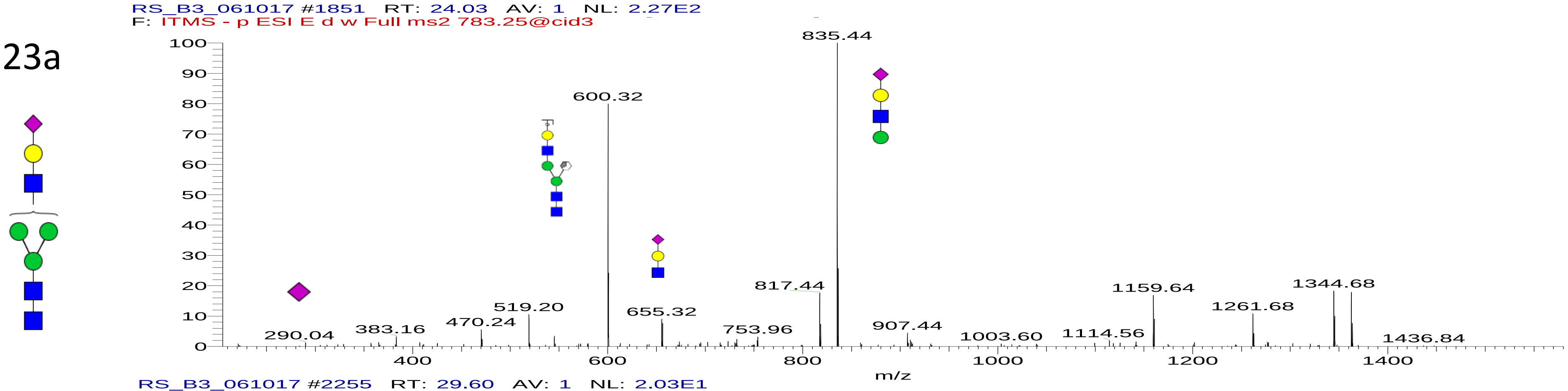

23b

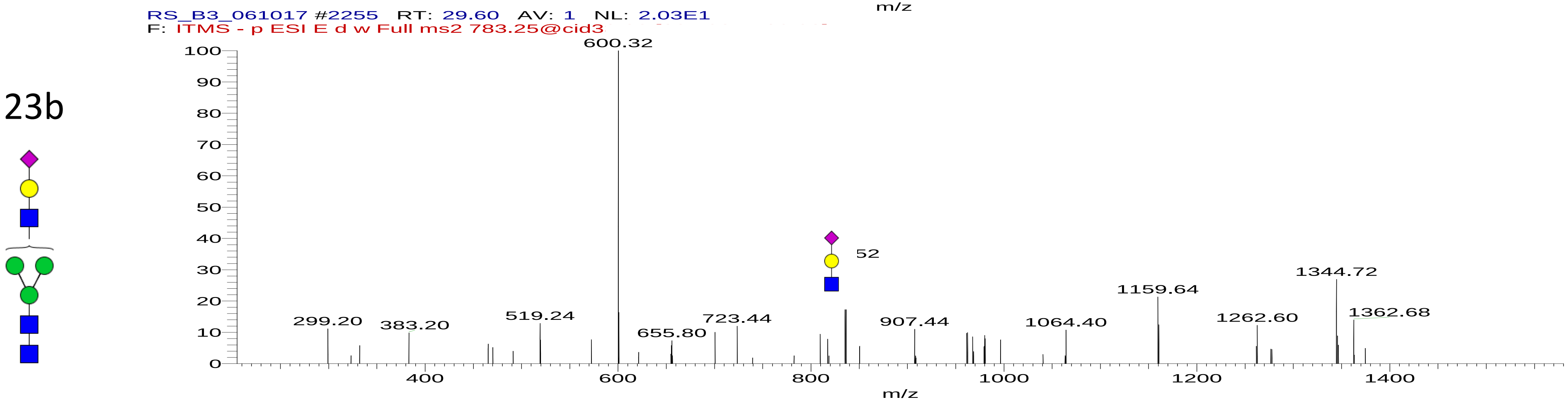

23c

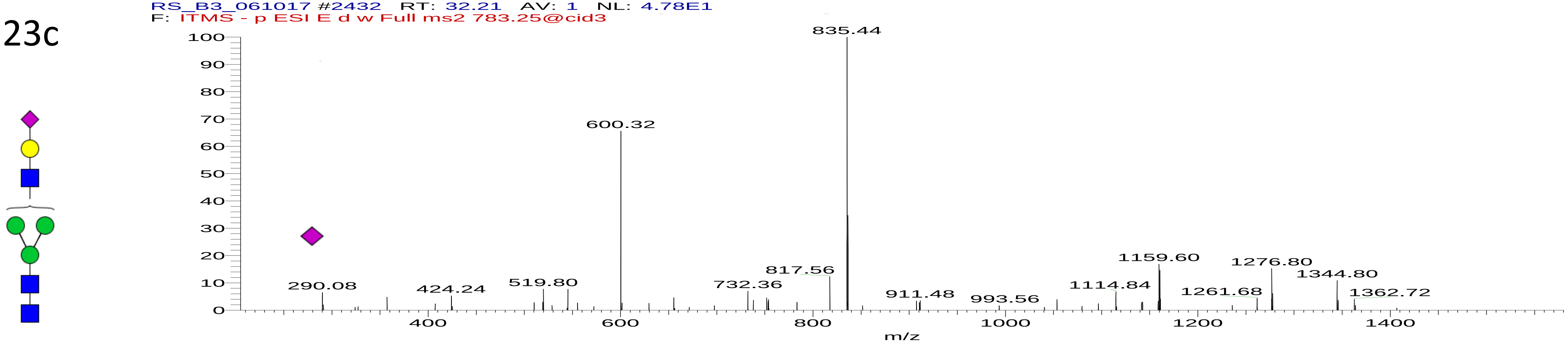

(Hex)3 (HexNAc)1 + (Man)3(GlcNAc)2

GlytouCan ID: G97385ZA

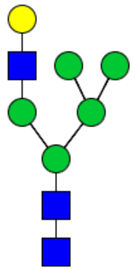

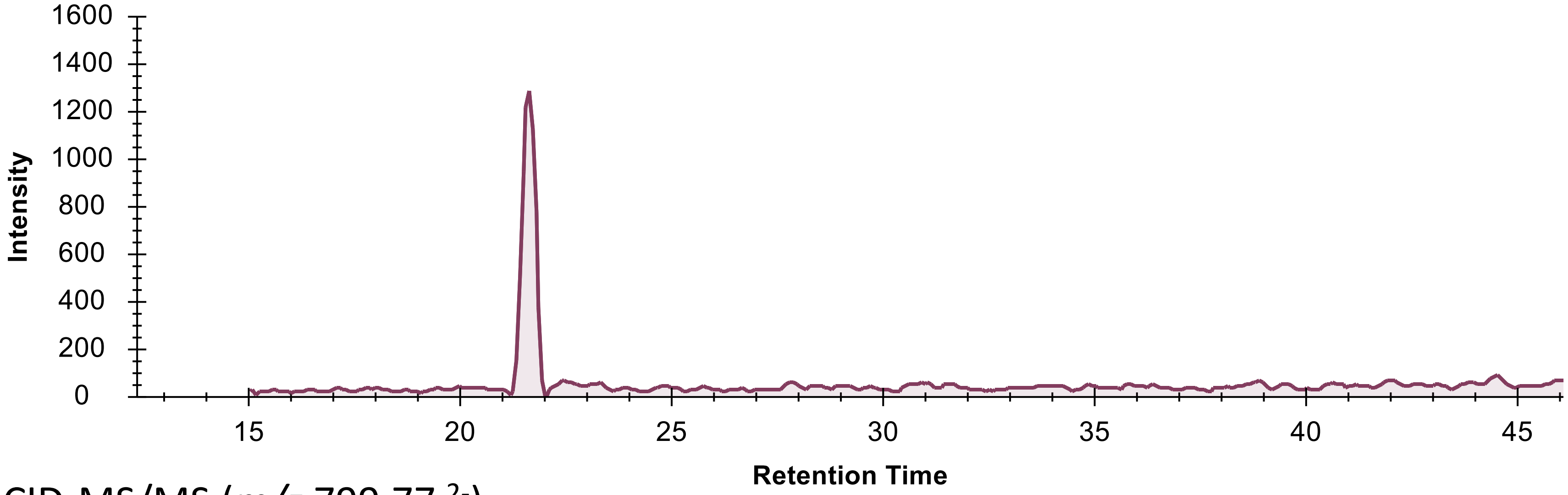

CID-MS/MS (*m/z* 799.77 $^{2-}$)

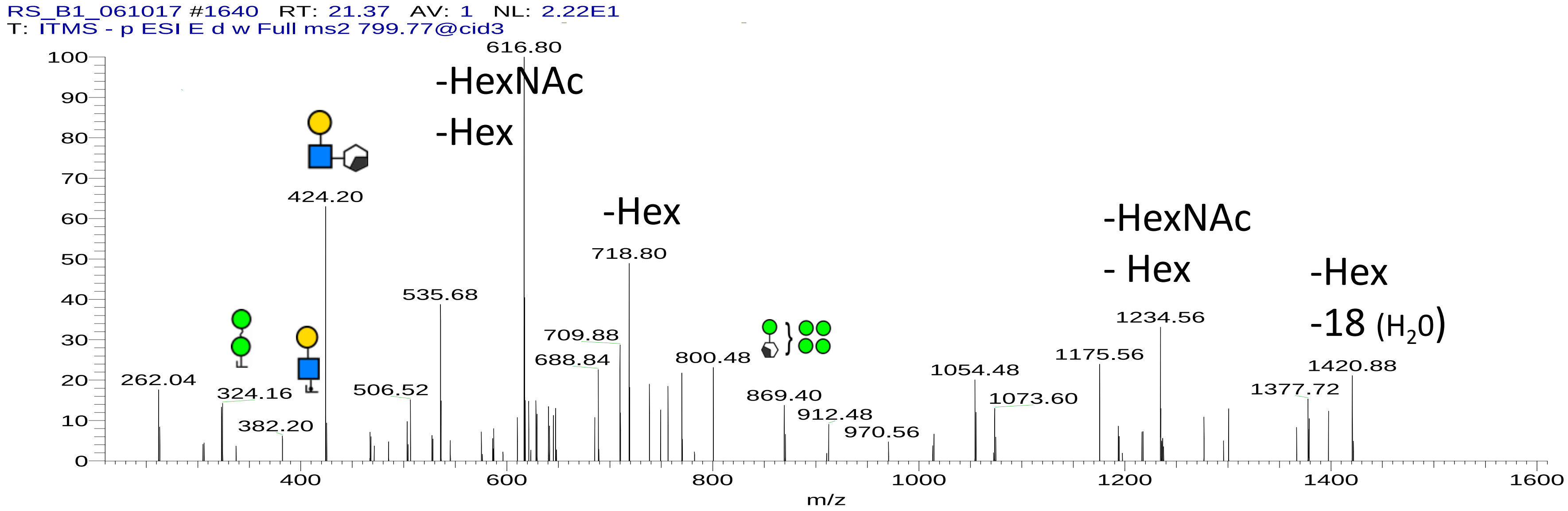

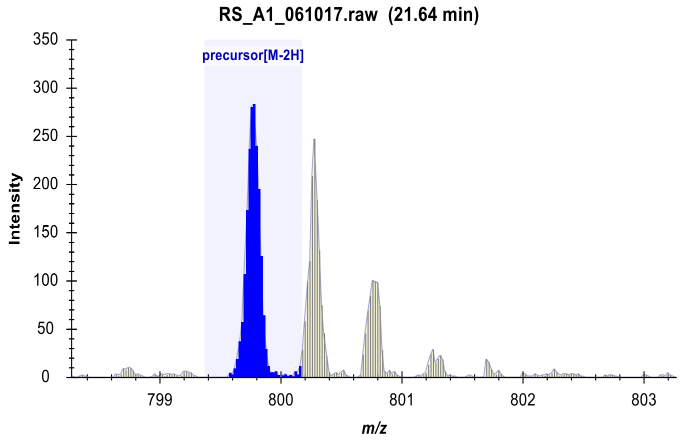

Glycan ID: 24

(Hex)1 (HexNAc)2 (Deoxyhexose)1 + (Man)3(GlcNAc)2

GlytouCan ID: G32915BP

[M-2H] 812.29

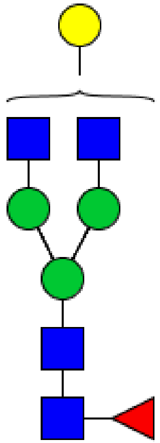

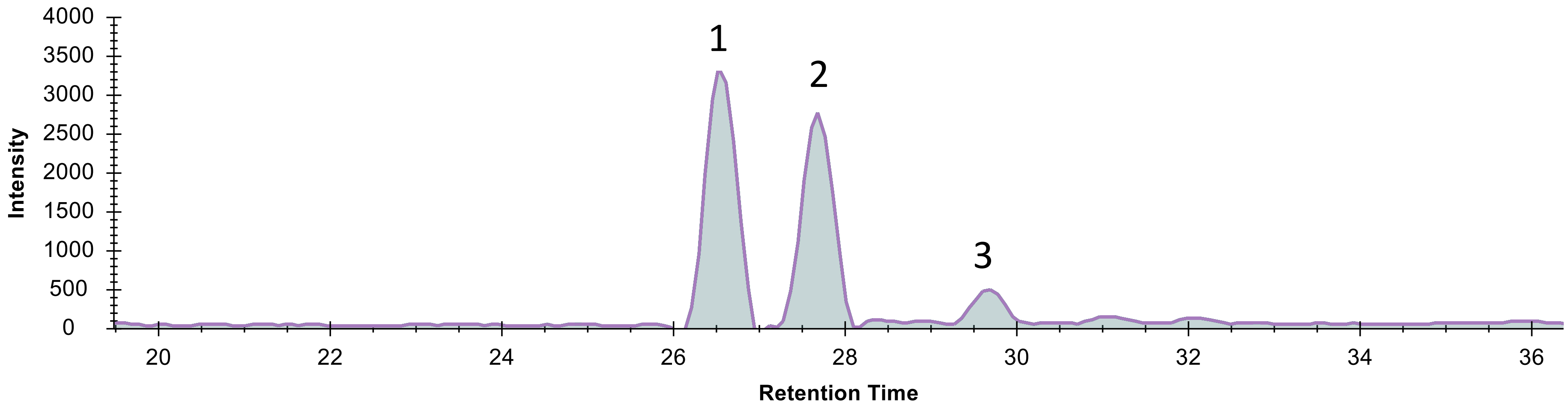

1

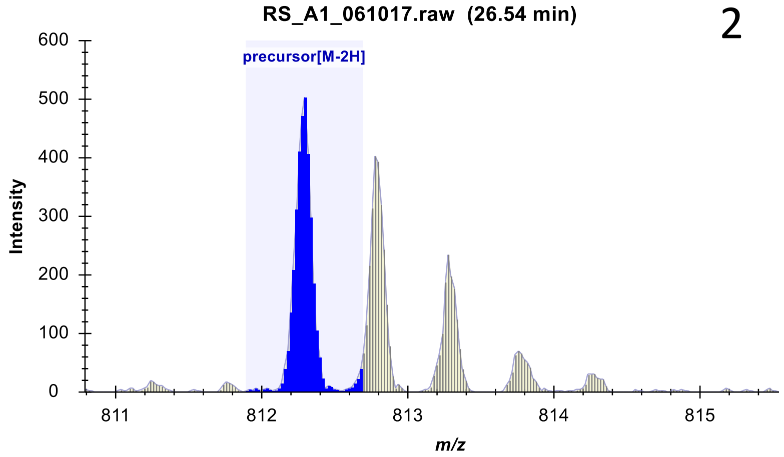

25a

2

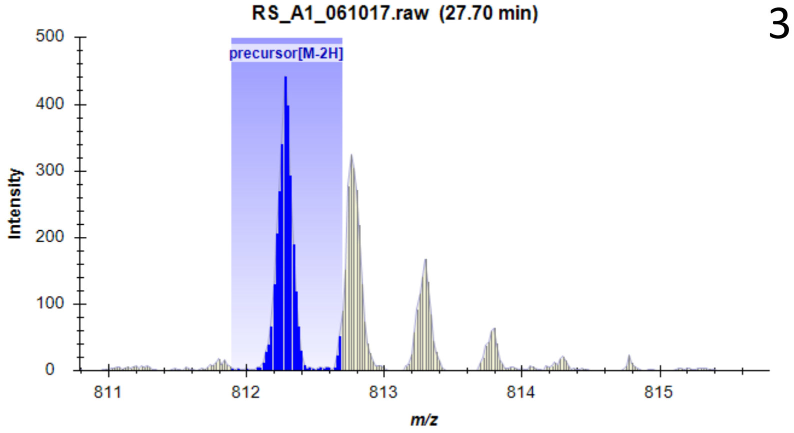

25b

3

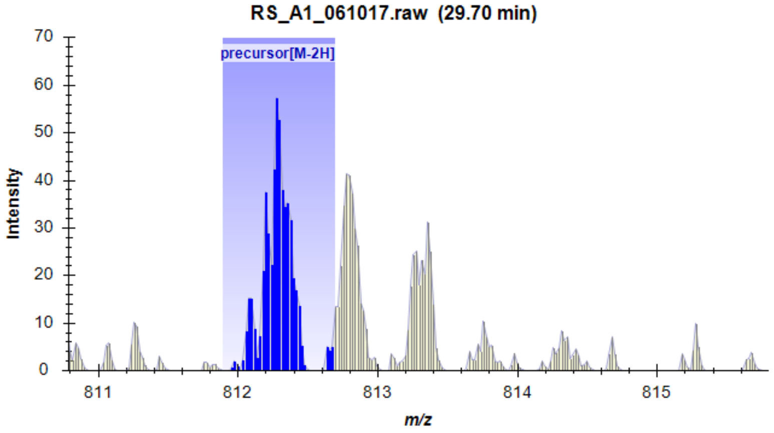

25c

GlytouCan ID: G32915BP

CID-MS/MS
(m/z 812.29 $^{2-}$)

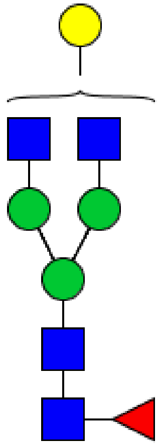

25a

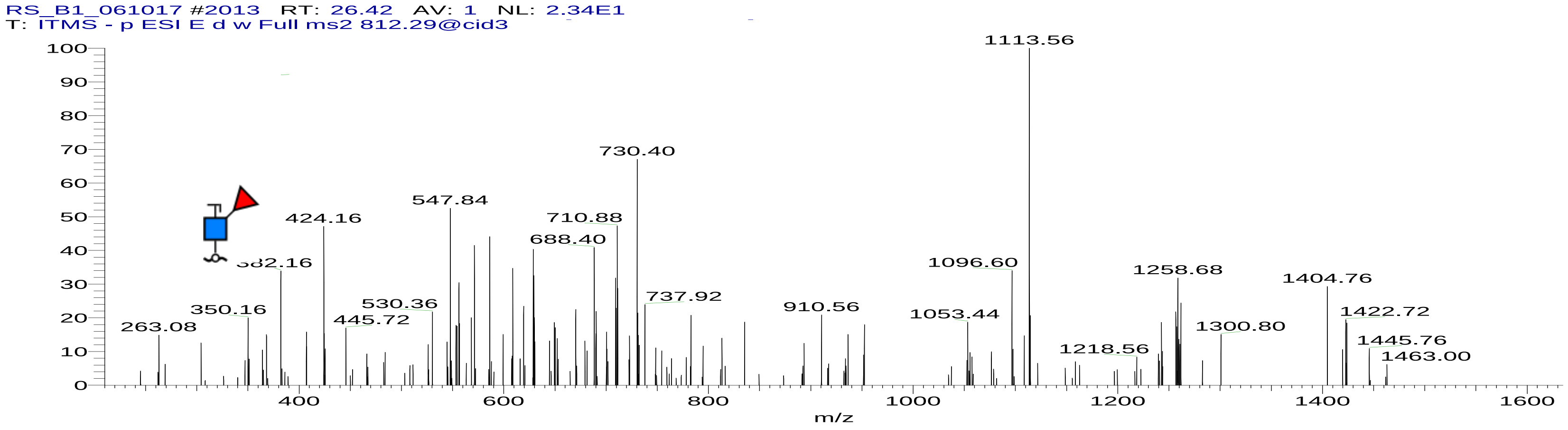

25b

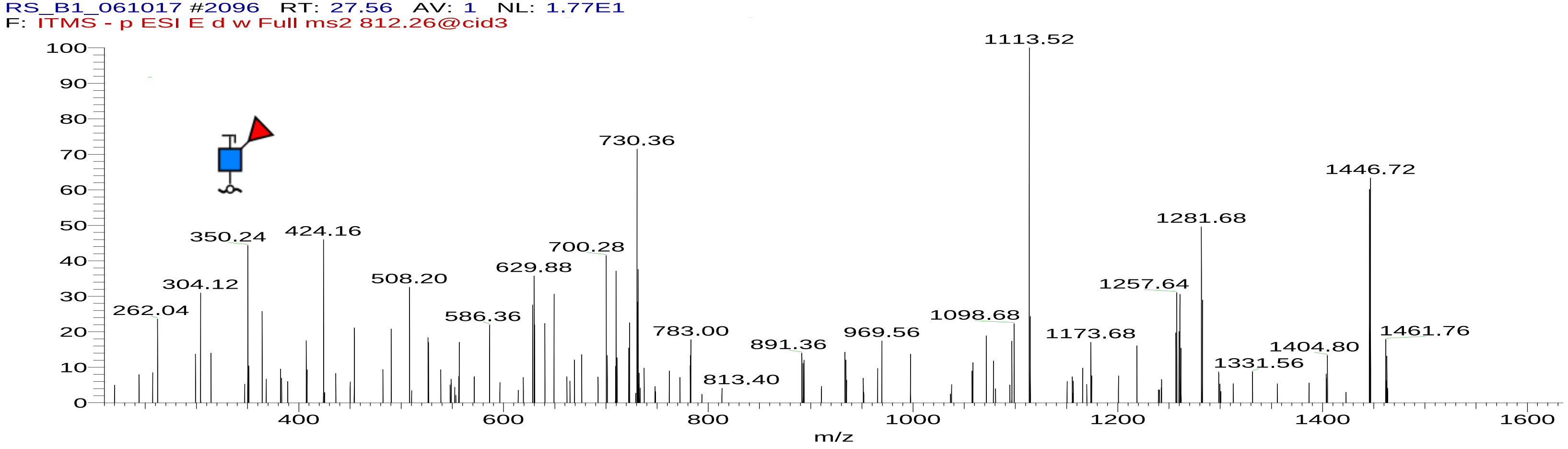

25c

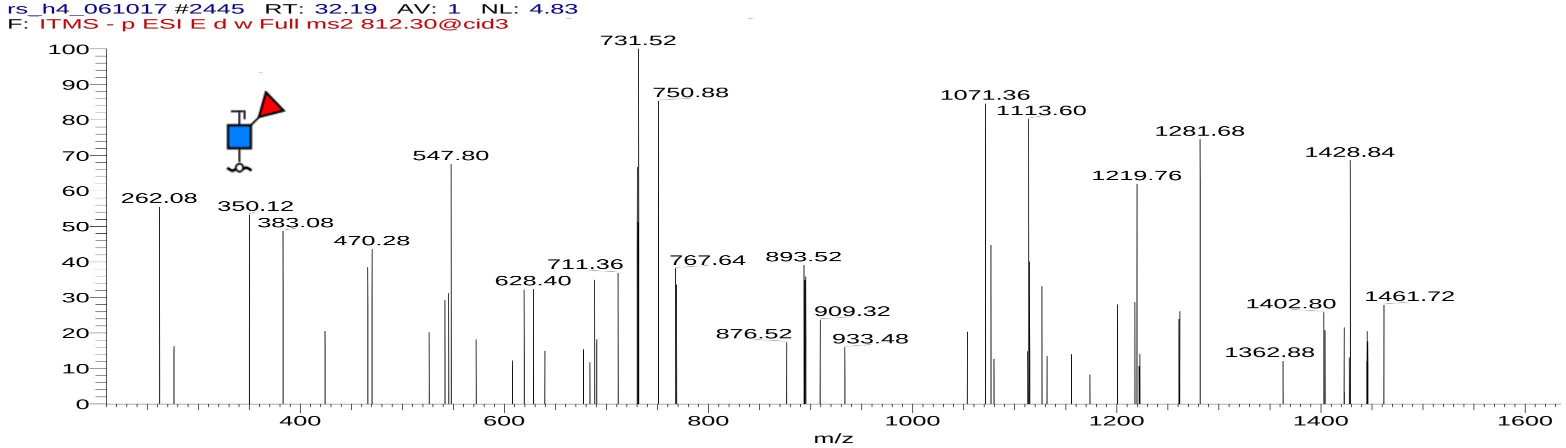

(Hex)2 (HexNAc)2 + (Man)3(GlcNAc)2

GlytouCan ID: G10179FS

[M-2H] 820.29

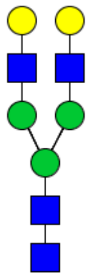

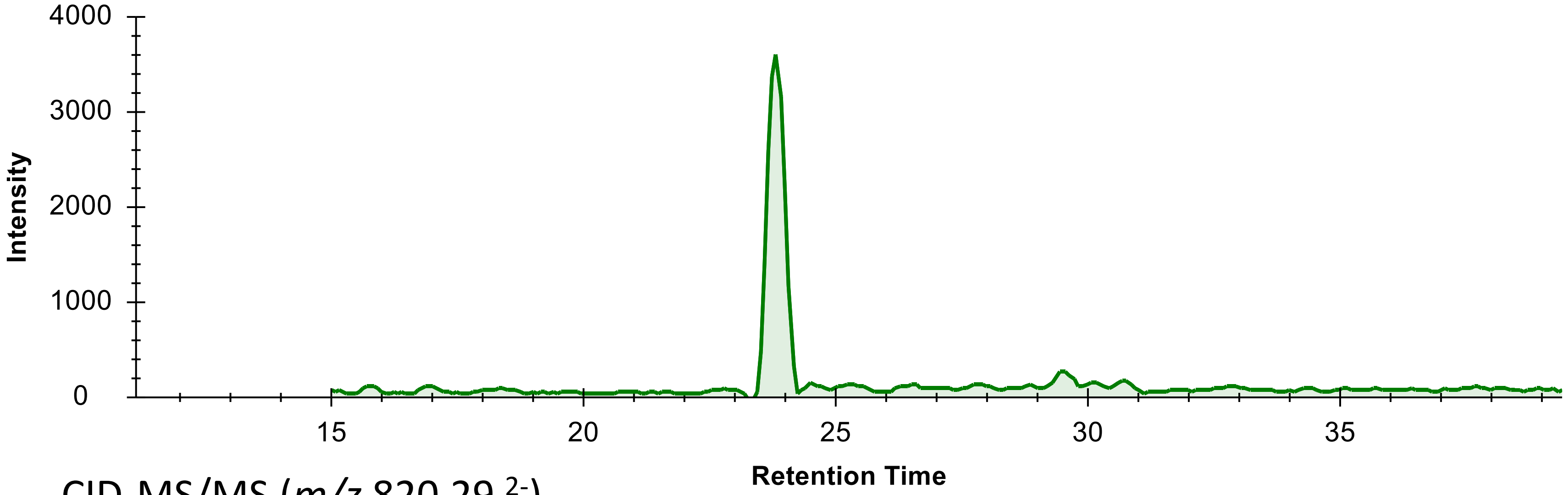

CID-MS/MS (m/z 820.29 $^{2-}$)

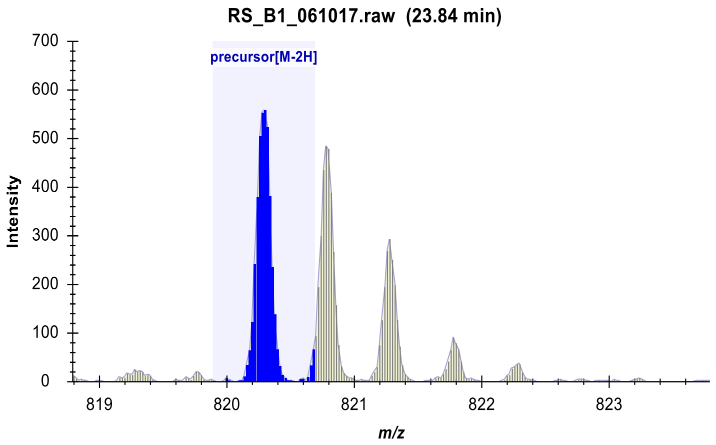

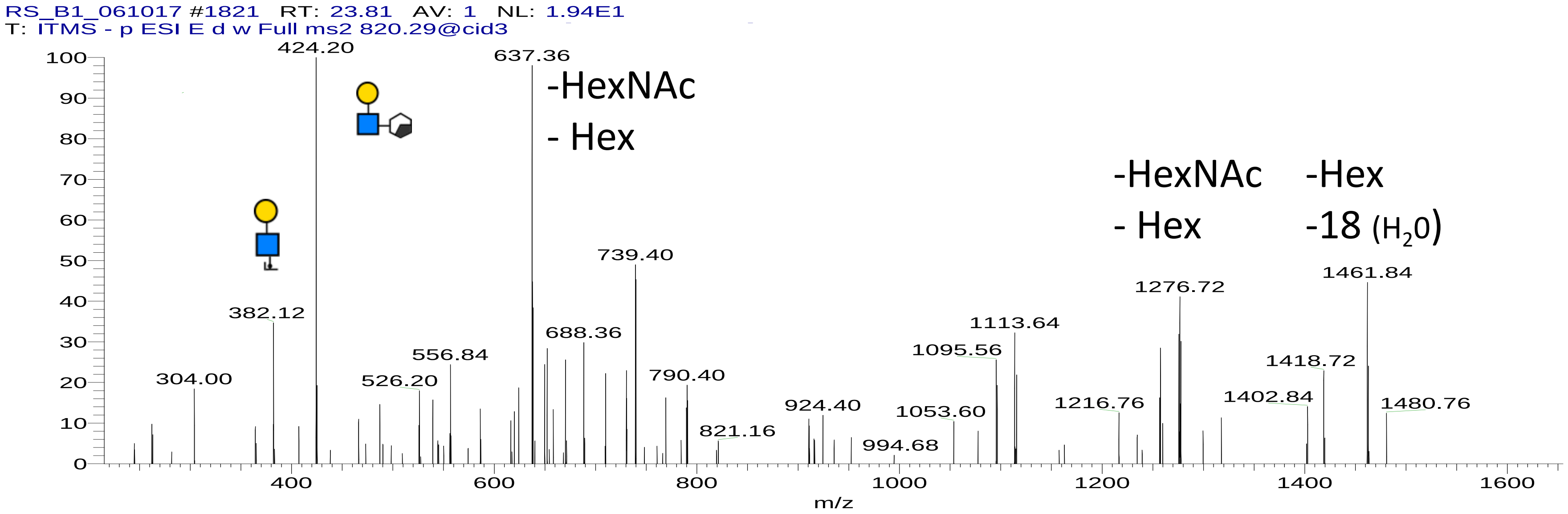

Gycan ID: 26

(Hex)1 (HexNAc)1 (Deoxyhexose)1 (NeuAc)1 +
(Man)3(GlcNAc)2
[M-2H] 856.31
GlytouCan ID: G58809AD

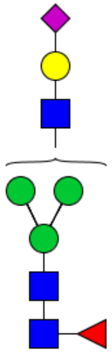

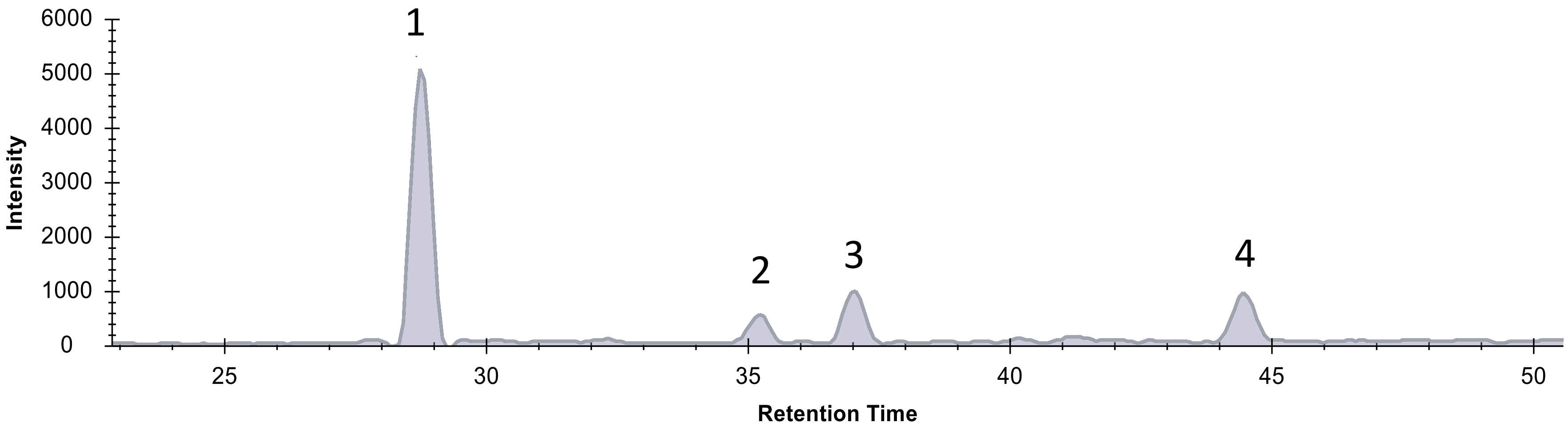

1

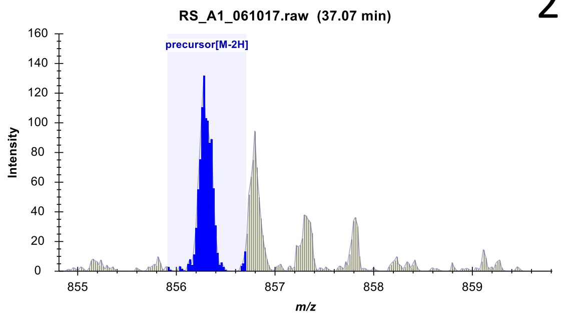

27a

2

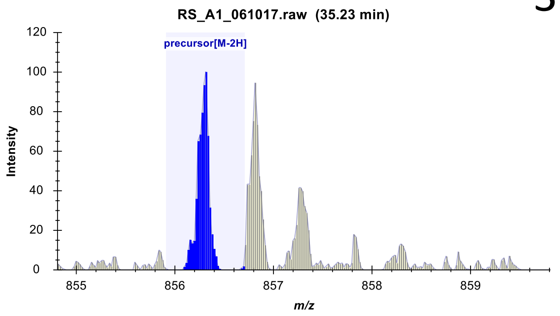

27b

3

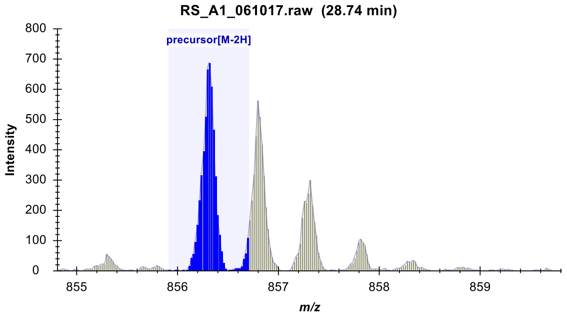

27c

4

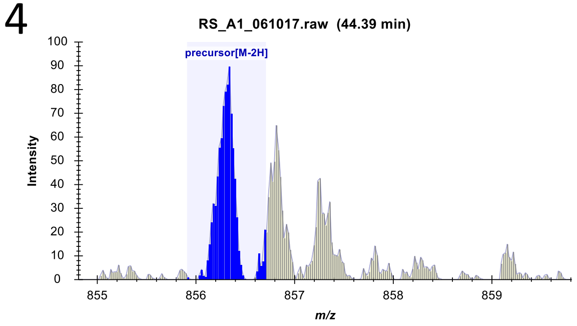

27d

GlytouCan ID: G58809AD

CID-MS/MS
(*m/z* 856.31^{2-})

27a

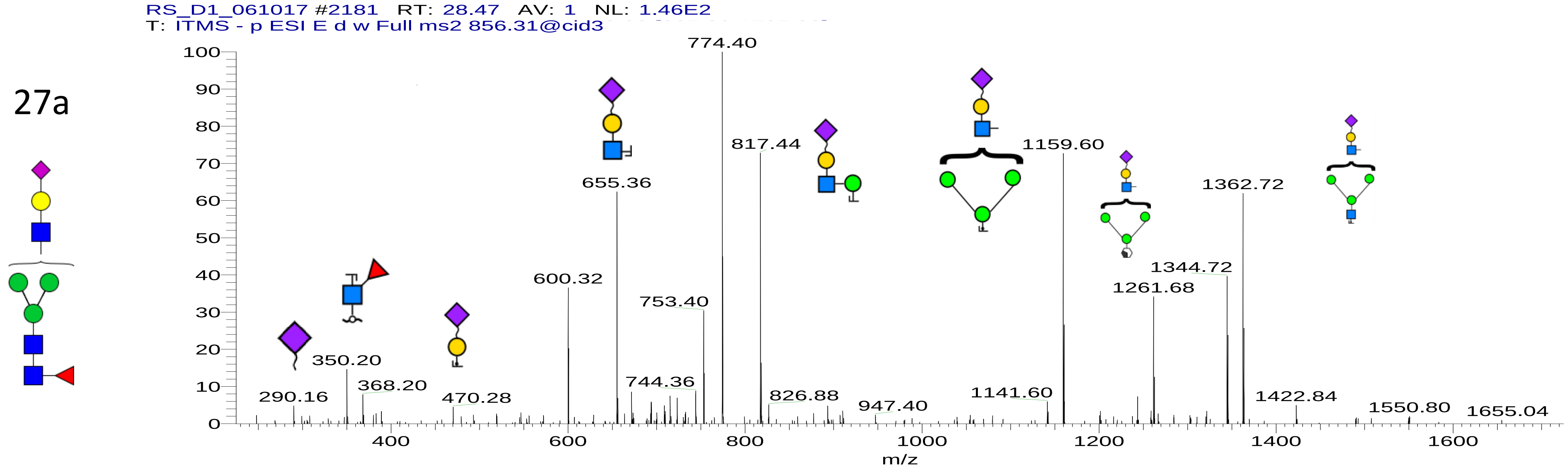

27b

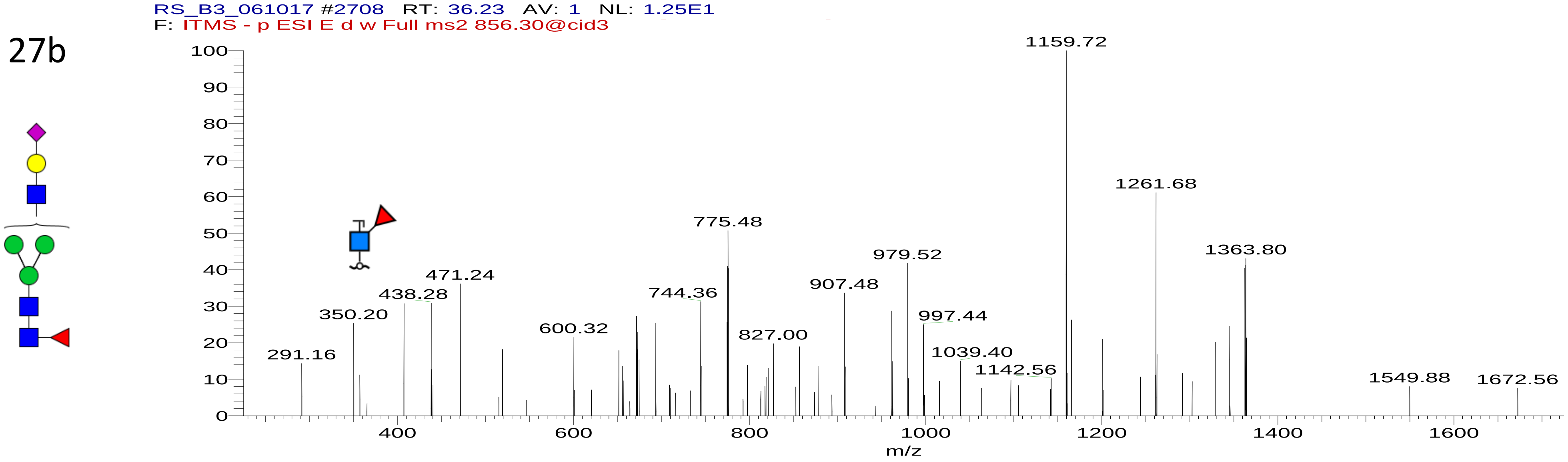

GlytouCan ID: G58809AD

CID-MS/MS
(m/z 856.31^{2-})

27c

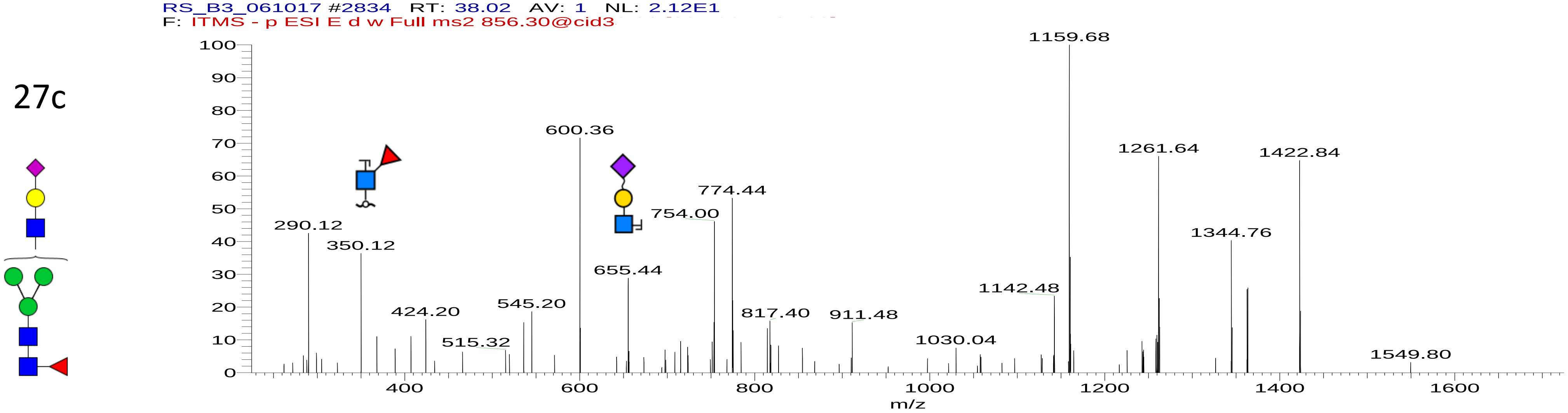

27d

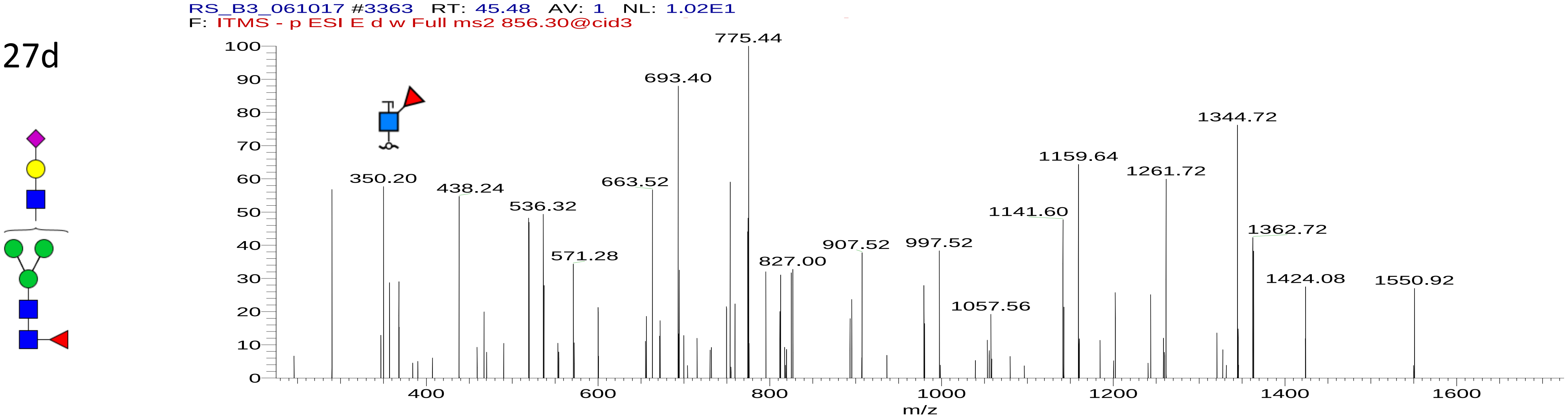

(Hex)2 (HexNAc)1 (NeuAc)1 + (Man)3(GlcNAc)2
GlytouCan ID: G29203KL
[M-2H] 864.29

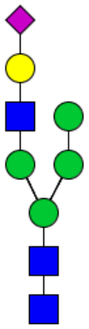

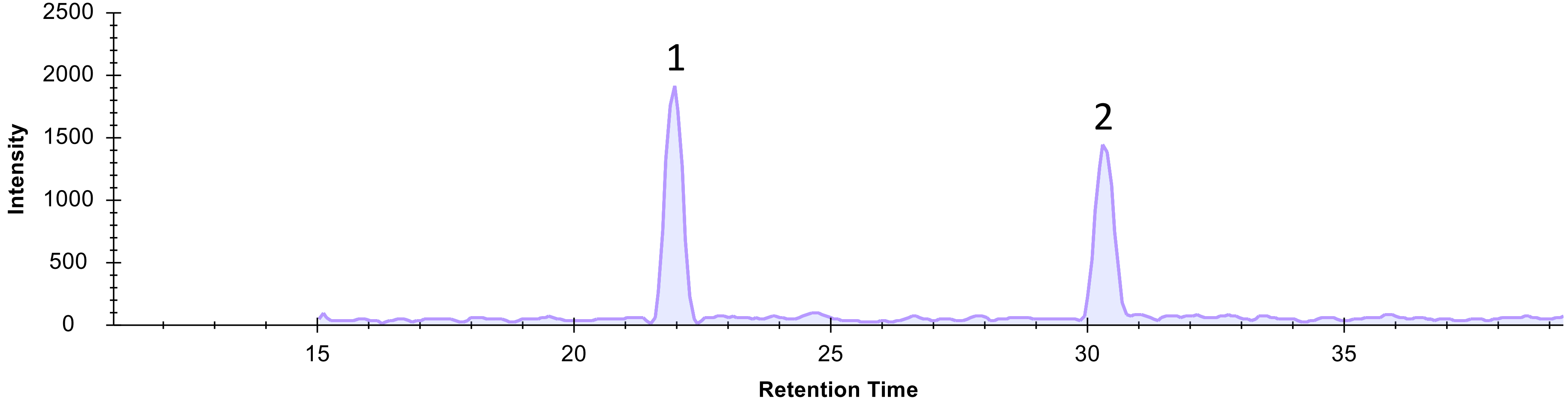

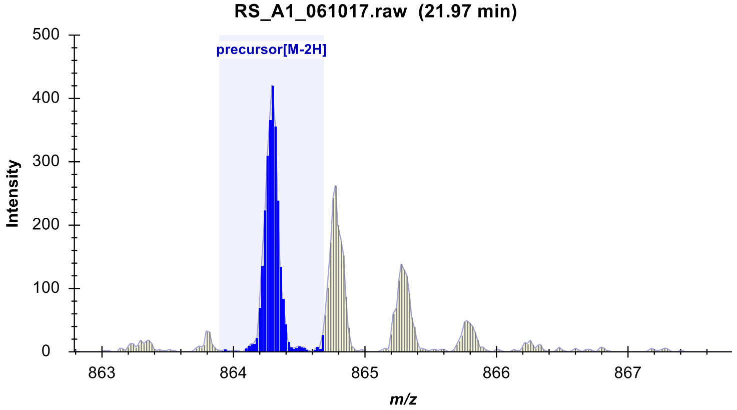

28a

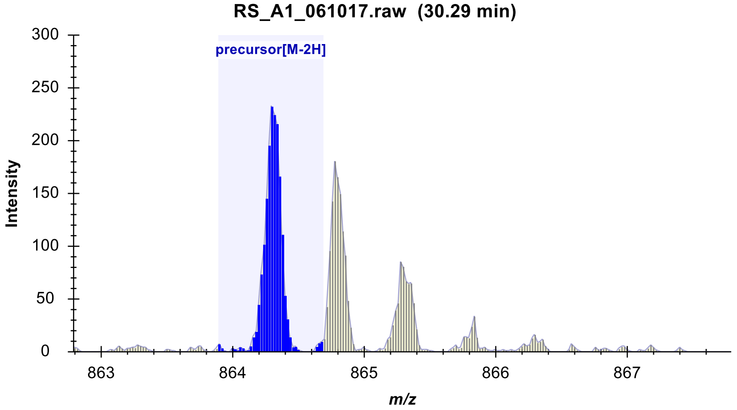

28b

GlytouCan ID: G29203KL

CID-MS/MS
(m/z 864.33 $^{2-}$)

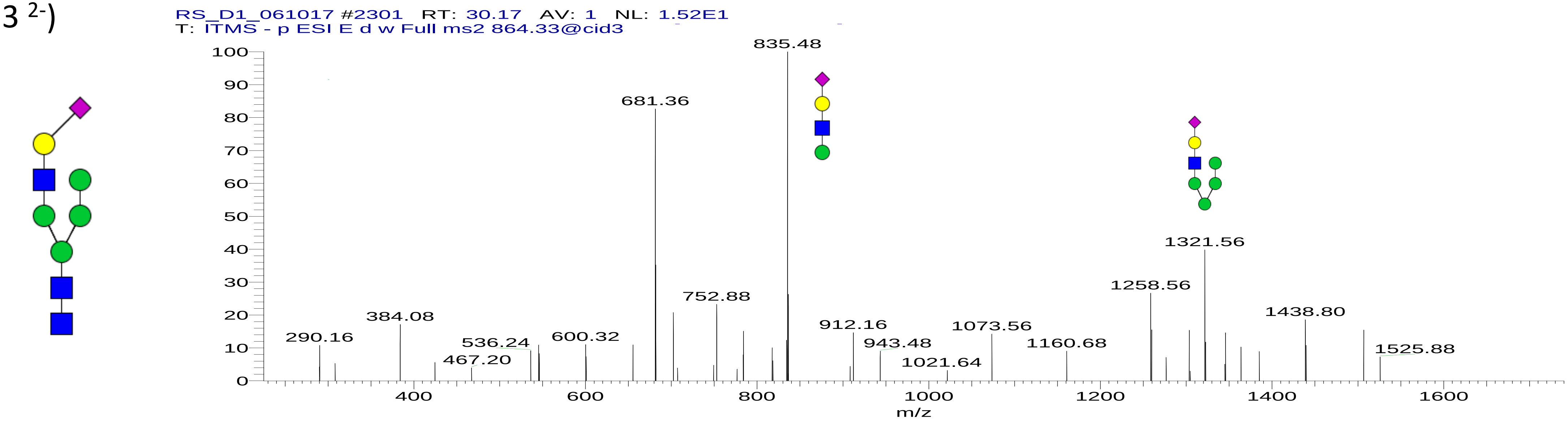

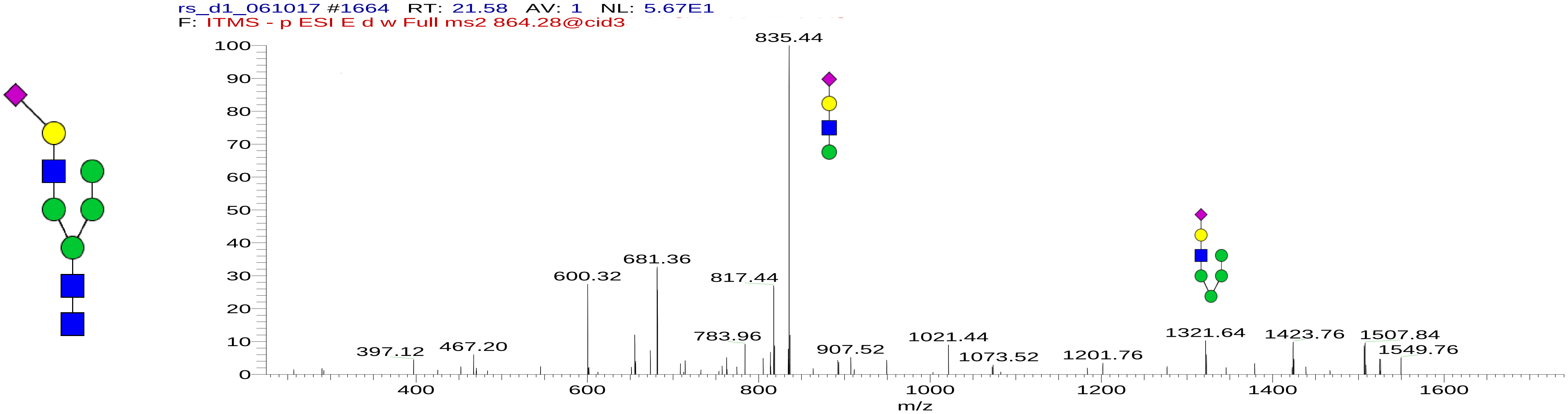

(Hex)2 (HexNAc)2 (Deoxyhexose)1 + (Man)3(GlcNAc)2
GlytouCan ID: G16768LX
[M-2H] 893.35

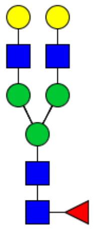

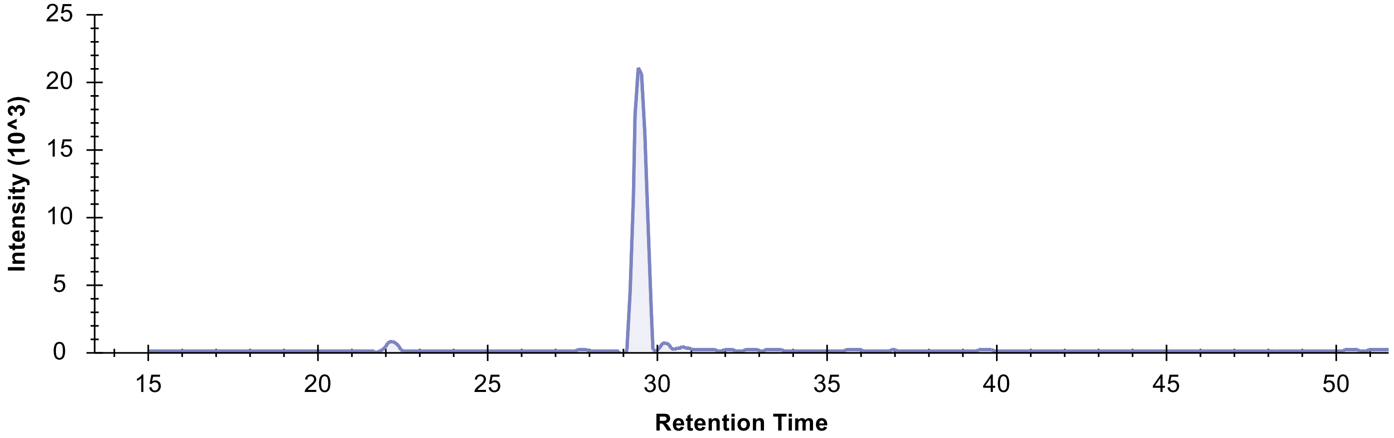

CID-MS/MS (*m/z* 893.35 $^{2-}$)

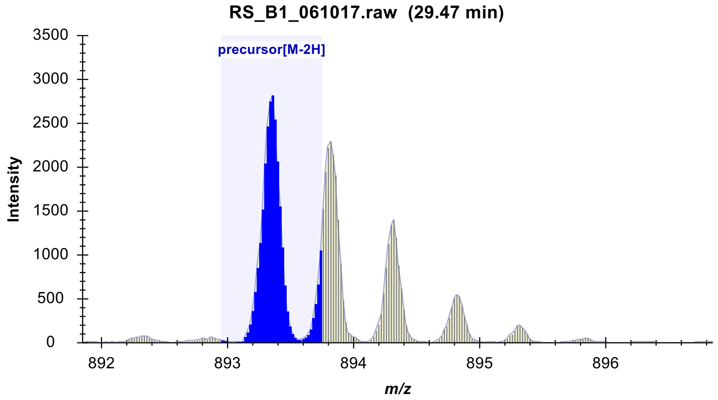

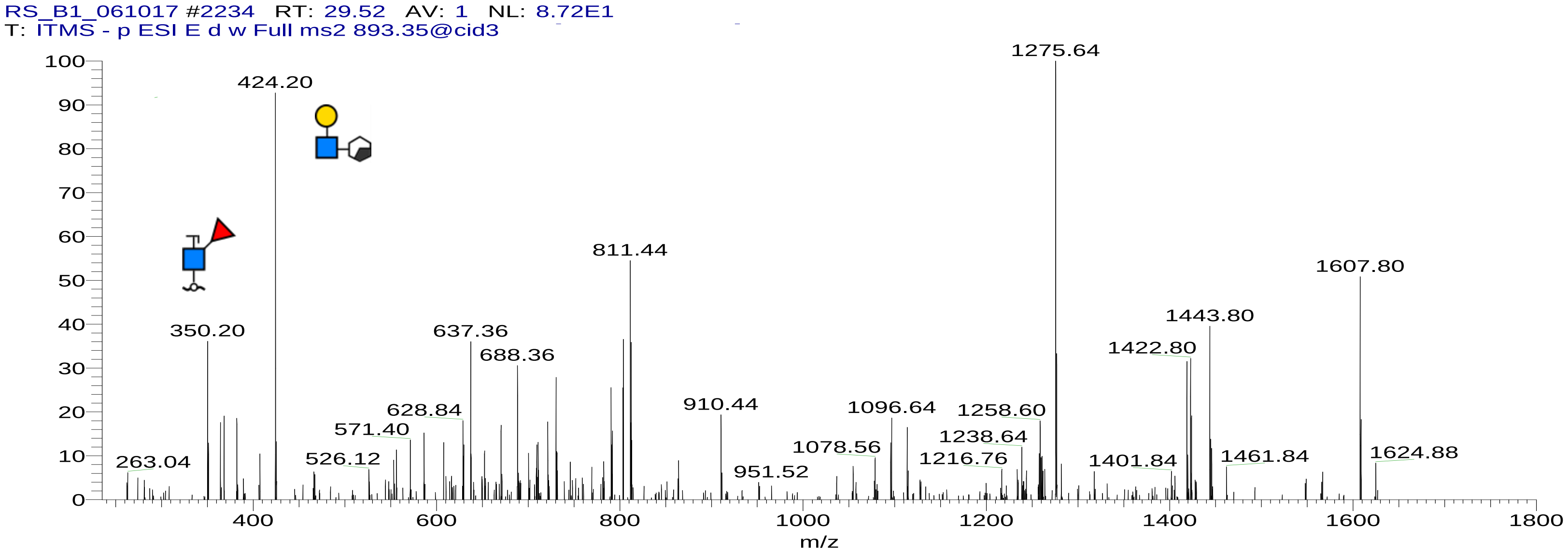

Glycan ID: 29

(HexNAc)1 (NeuAc)1 + (Man)3(GlcNAc)2

[M-2H] 702.23

O glycan contaminant?

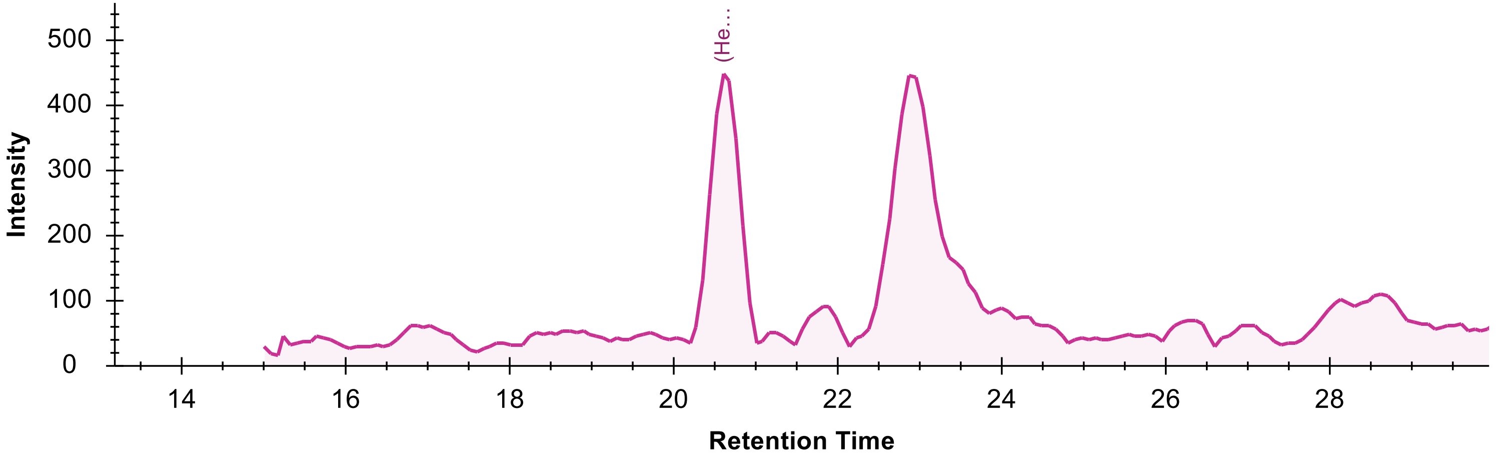

CID-MS/MS (*m/z* 702.23 $^{2-}$)

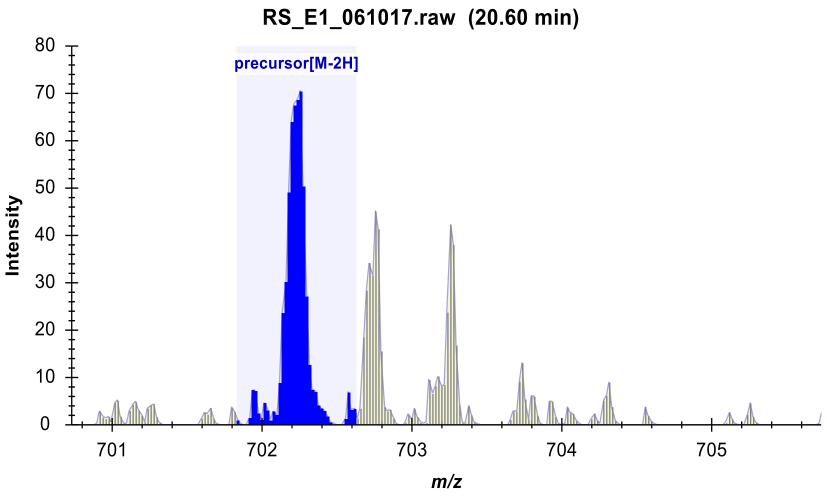

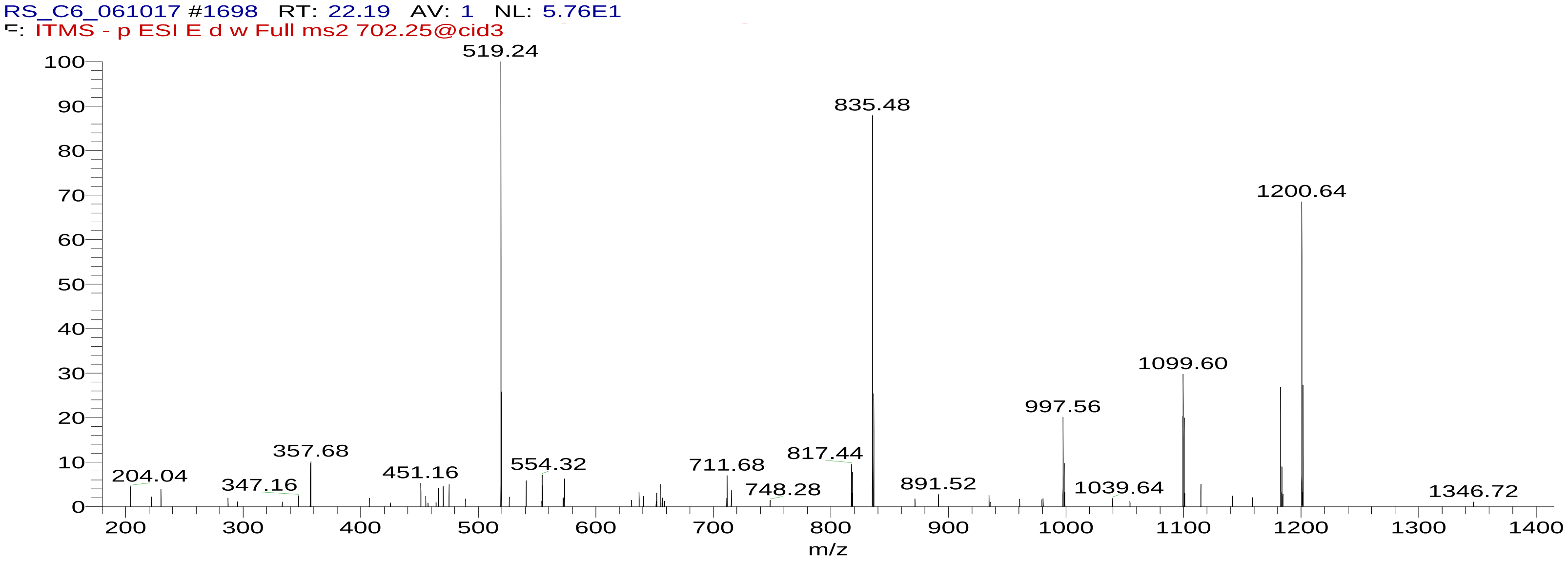

Glycan ID: 30

(HexNAc)3 + (Man)3(GlcNAc)2

GlytouCan ID: G21251LN

[M-2H] 759.77

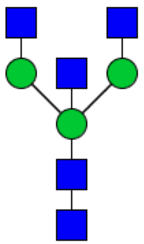

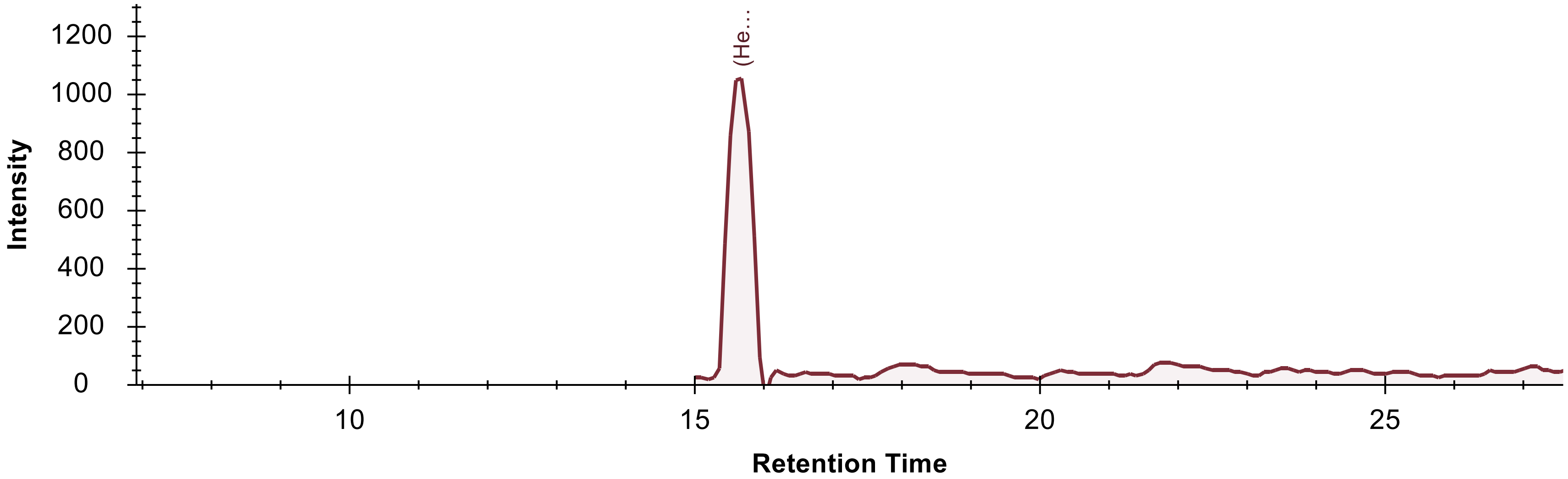

CID-MS/MS (*m/z* 759.77 $^{2-}$)

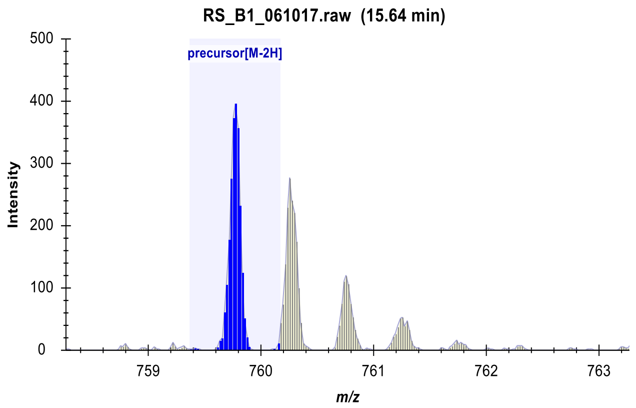

Glycan ID: 31

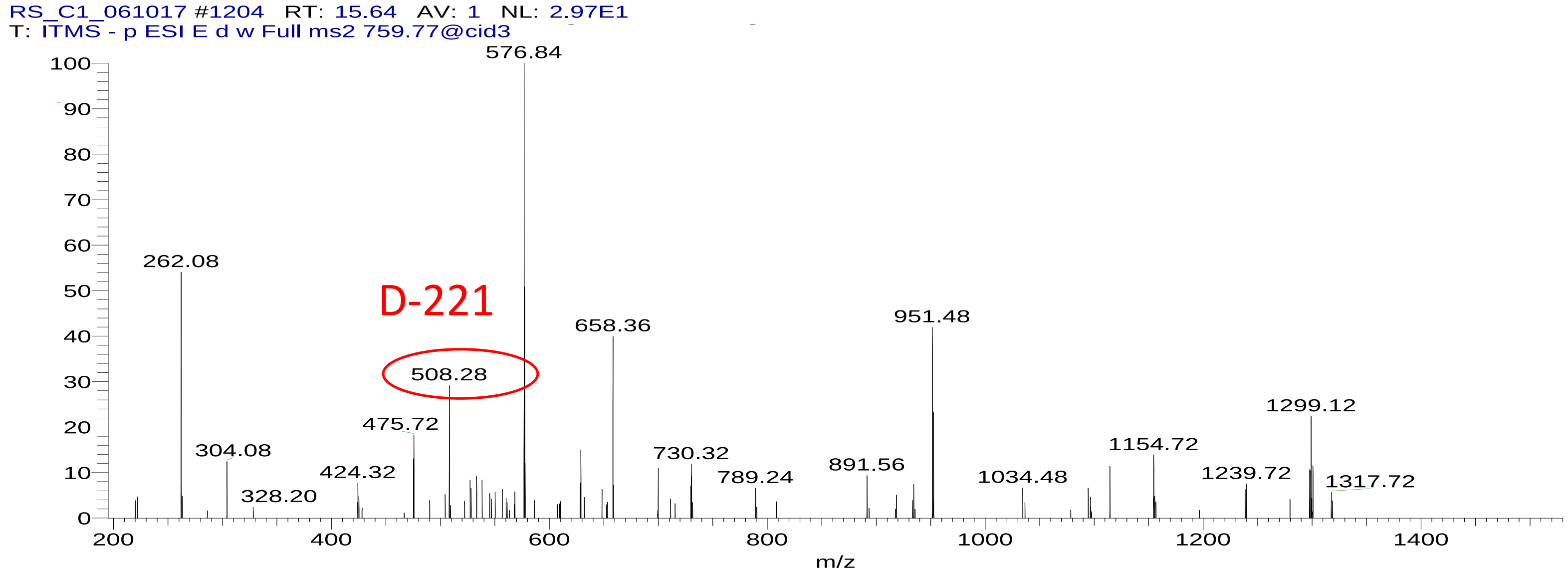

(HexNAc)2 (NeuAc)1 + (Man)3(GlcNAc)2

[M-2H] 803.77

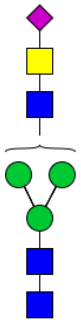

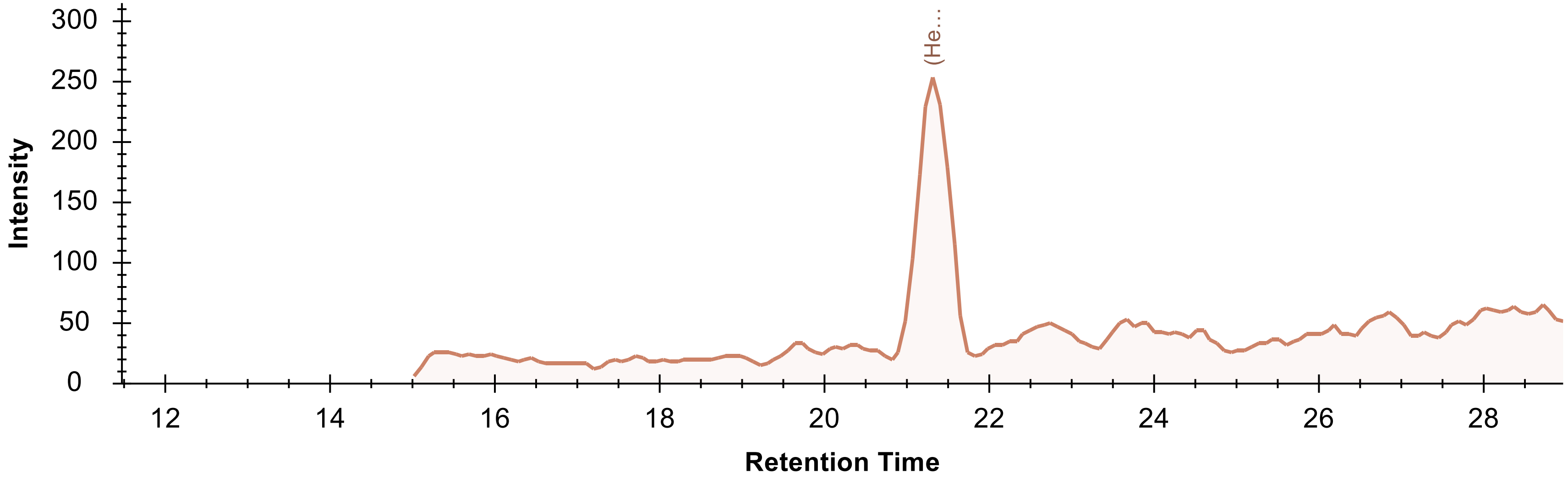

CID-MS/MS (*m/z* 803.77 $^{2-}$)

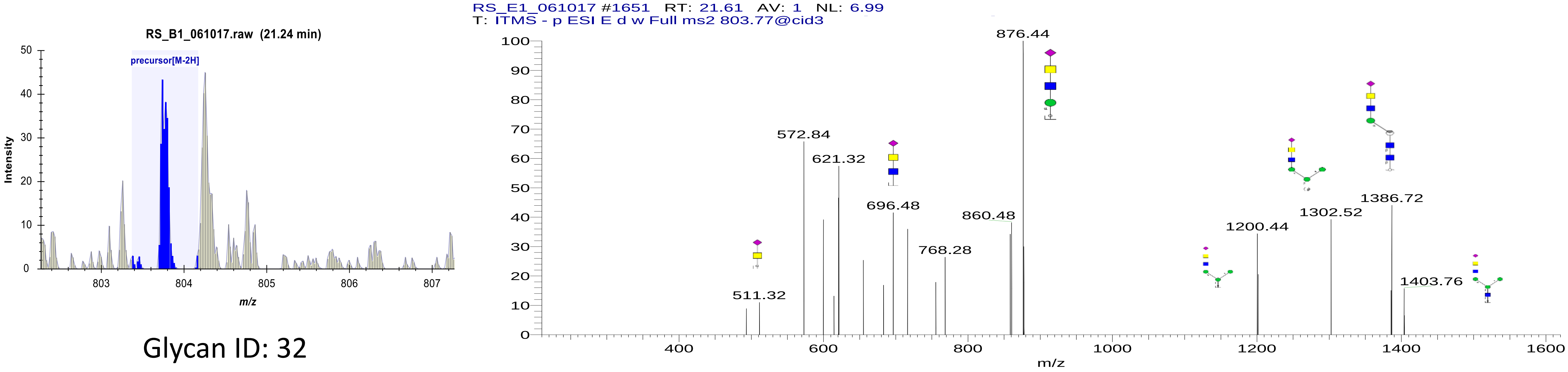

Glycan ID: 32

(HexNAc)3 (Deoxyhexose)1 + (Man)3(GlcNAc)2

[M-2H] 832.8

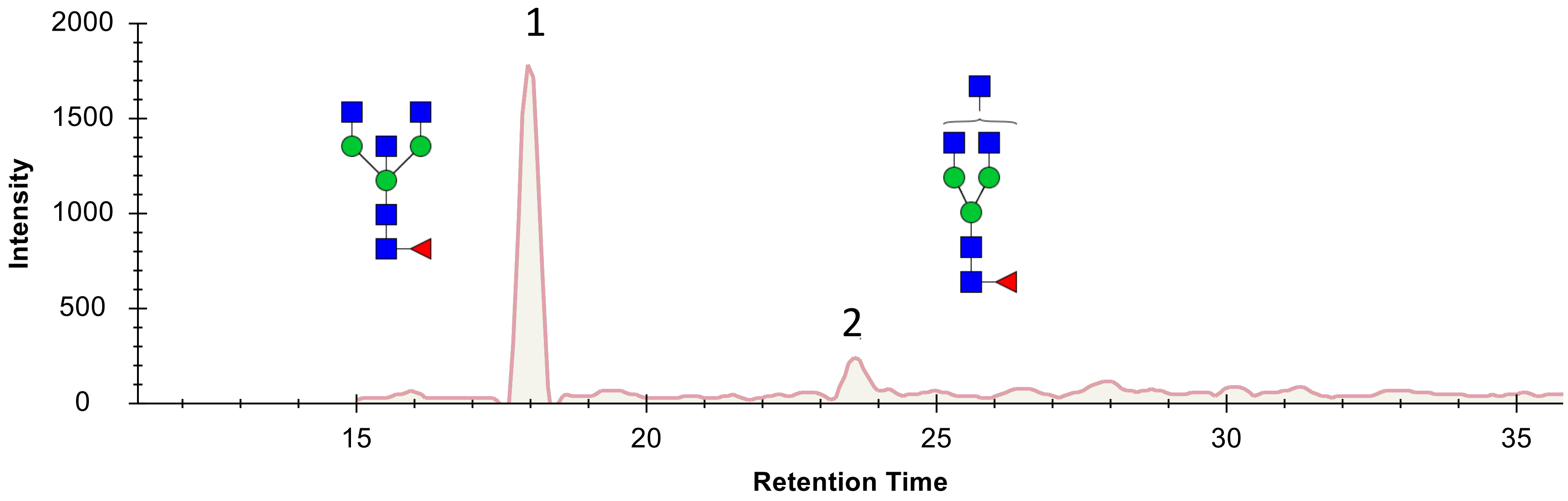

1

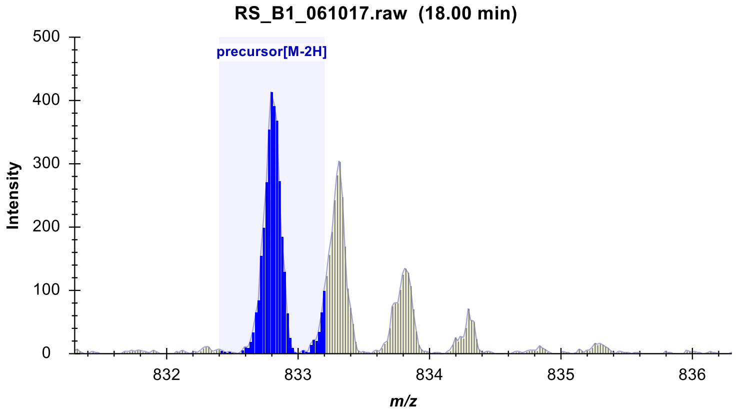

33a

2

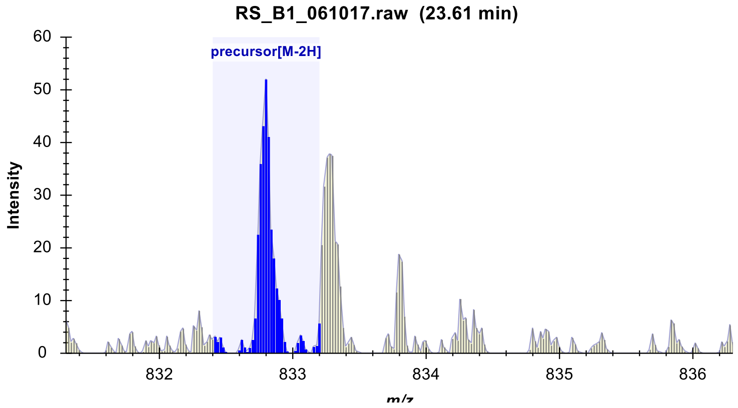

33b

CID-MS/MS
(*m/z* 832.82 $^{2-}$)

D-221

33a

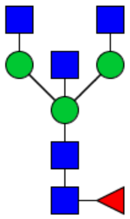

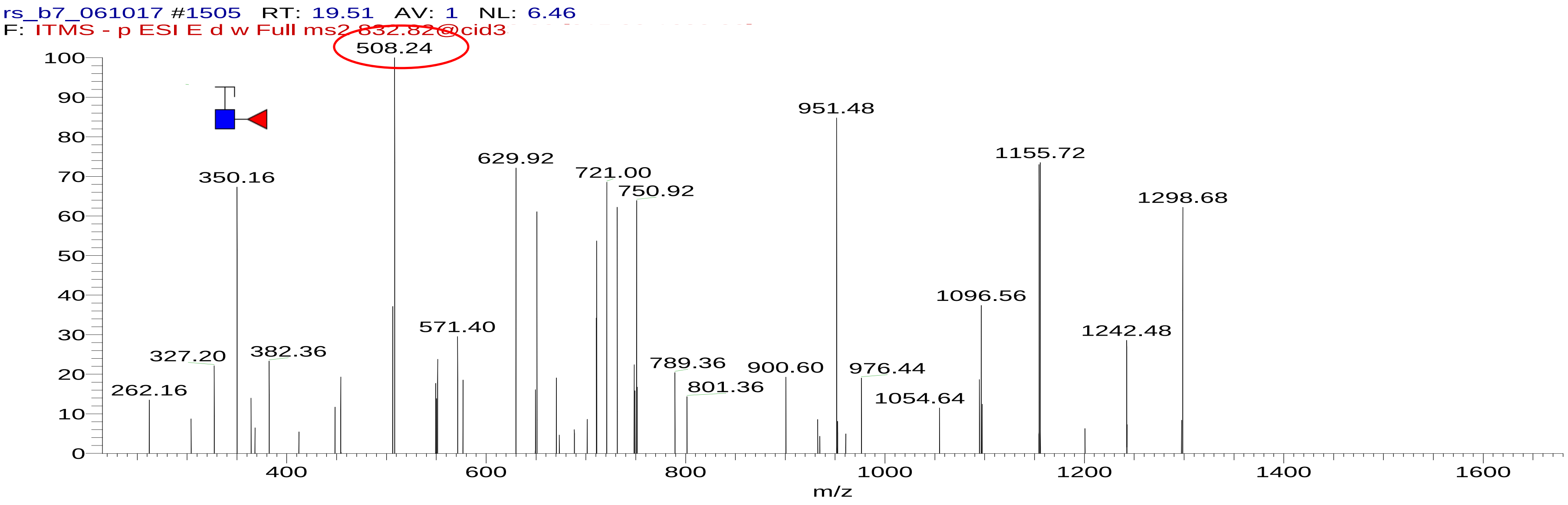

33b

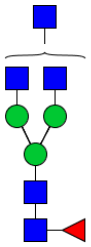

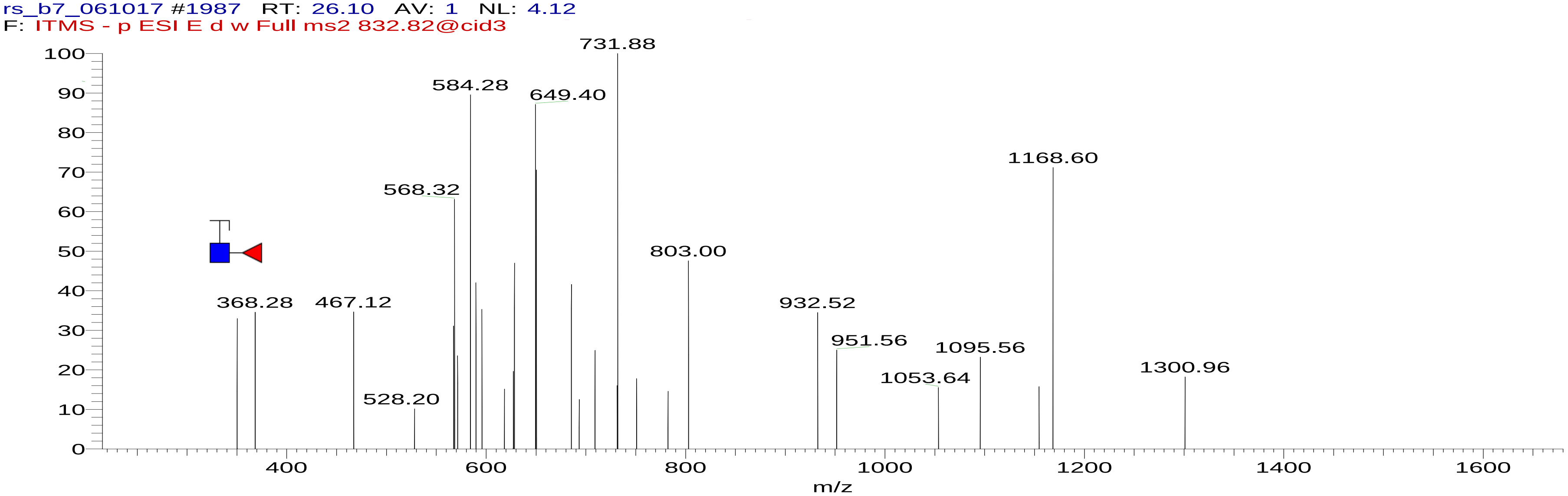

(Hex)1 (HexNAc)3 + (Man)3(GlcNAc)2
GlytouCan ID: G85287WC
[M-2H] 840.8

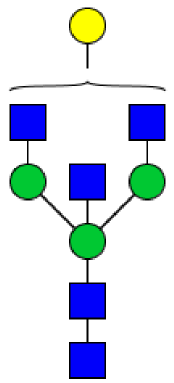

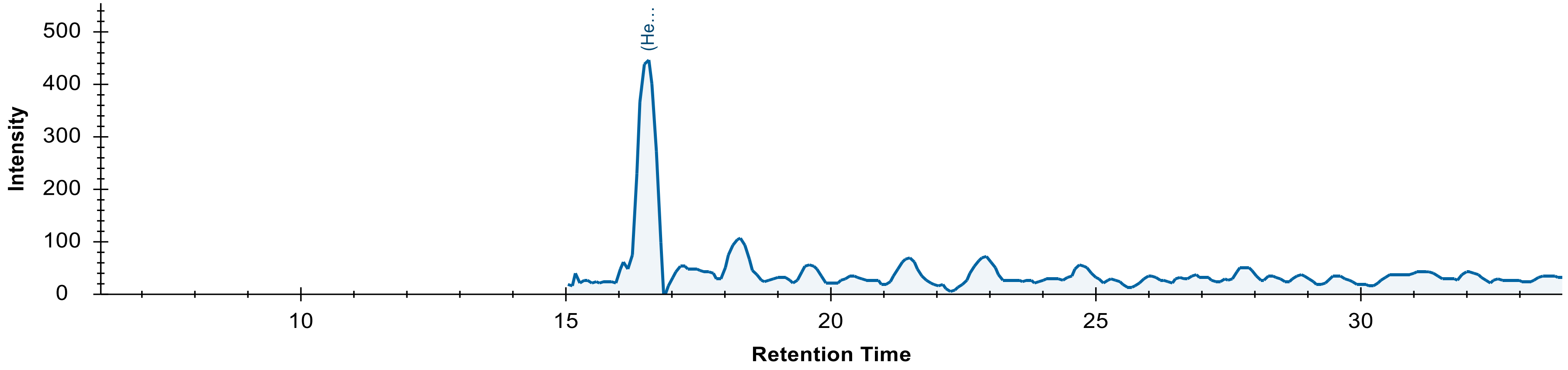

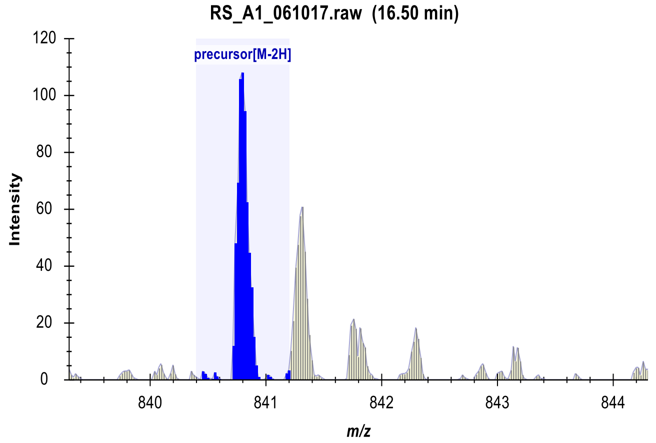

Glycan ID: 34

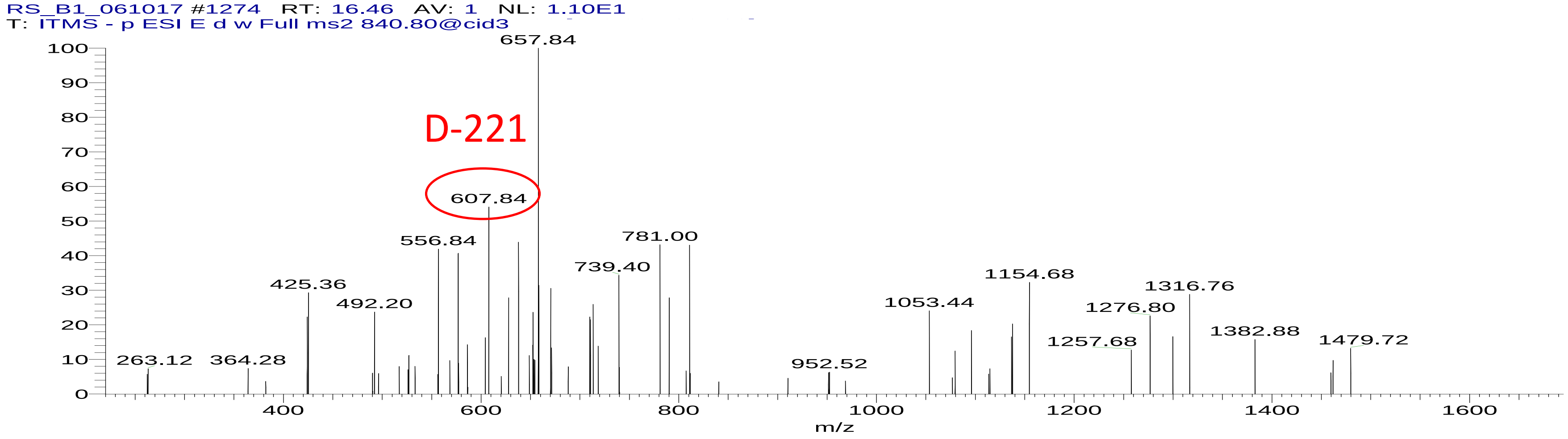

(HexNAc)2 (Deoxyhexose)1 (NeuAc)1 + (Man)3(GlcNAc)2

[M-2H] 876.81

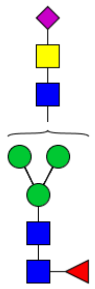

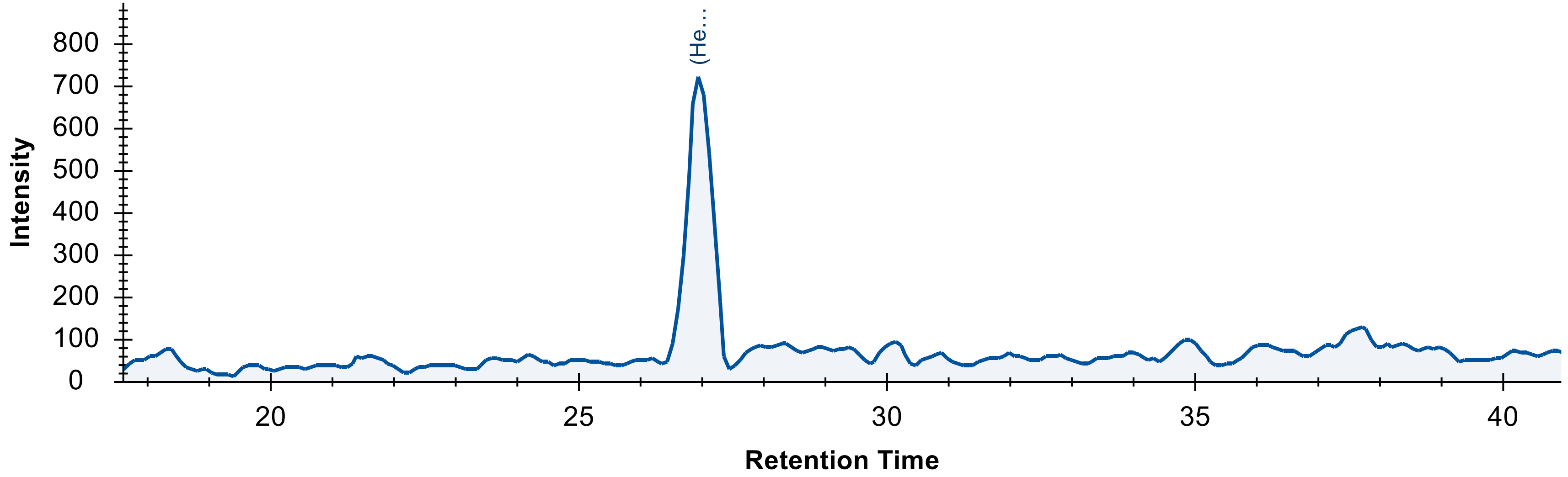

CID-MS/MS (*m/z* 876.81 $^{2-}$)

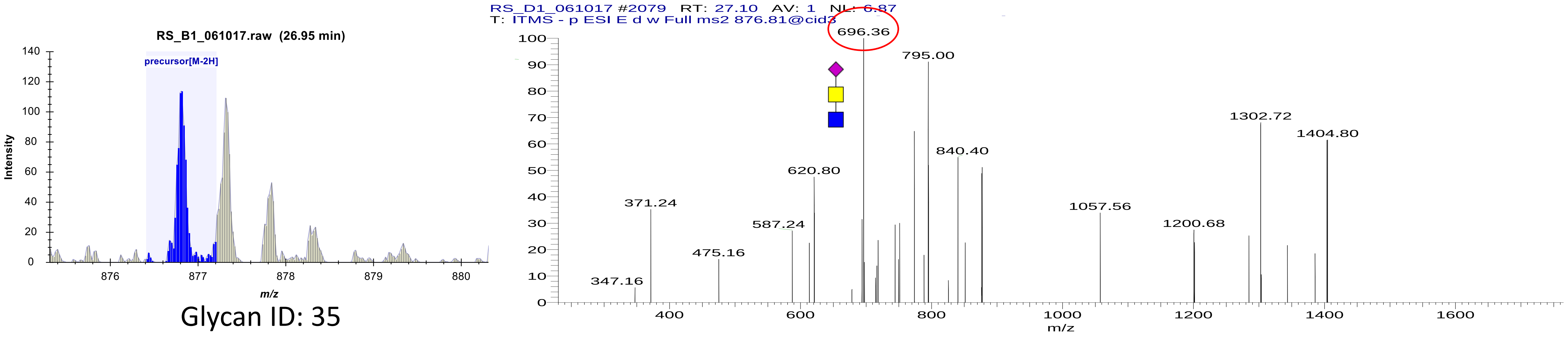

Glycan ID: 35

(Hex)1 (HexNAc)2 (NeuAc)1 + (Man)3(GlcNAc)2
GlytouCan ID: G62446JD

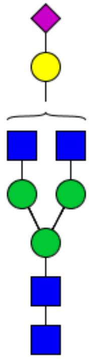

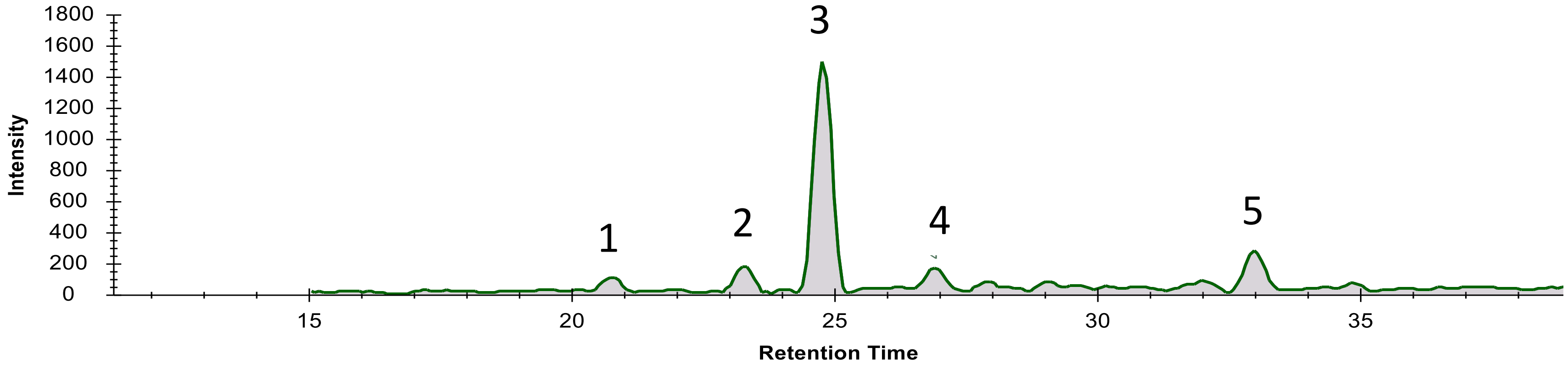

1

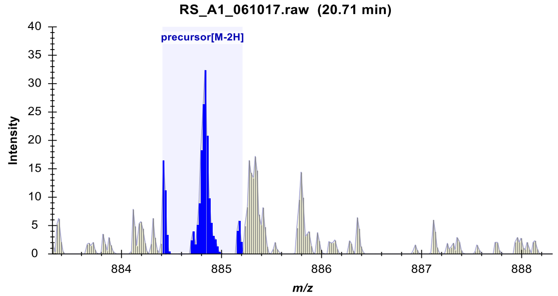

36a

2

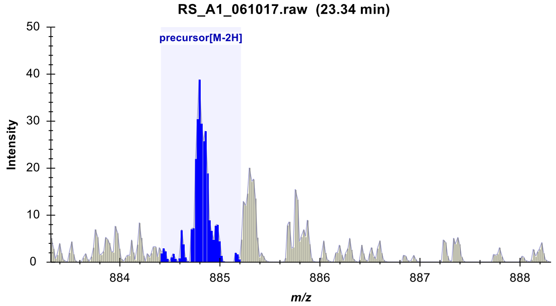

36b

3

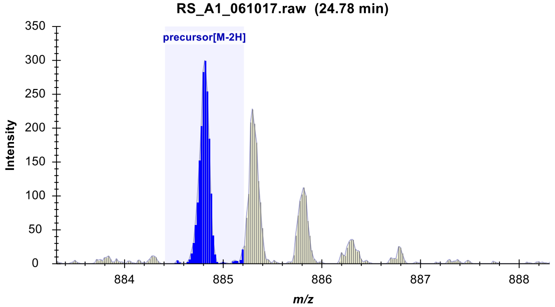

36c

4

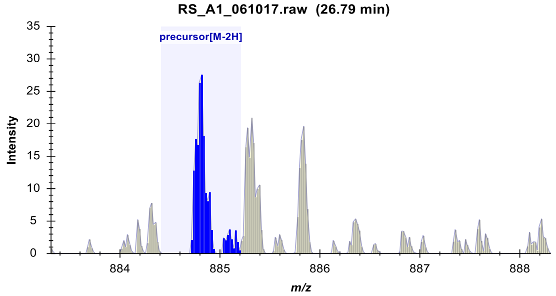

36d

5

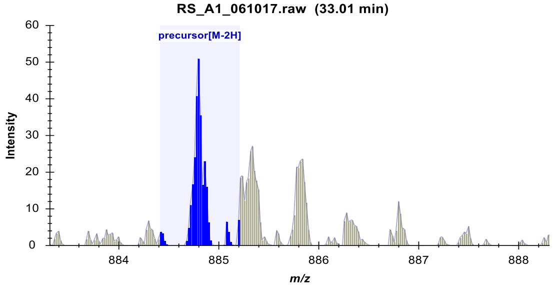

36e

GlytouCan ID: G62446JD

CID-MS/MS

(m/z 884.80 $^{2-}$)

36a

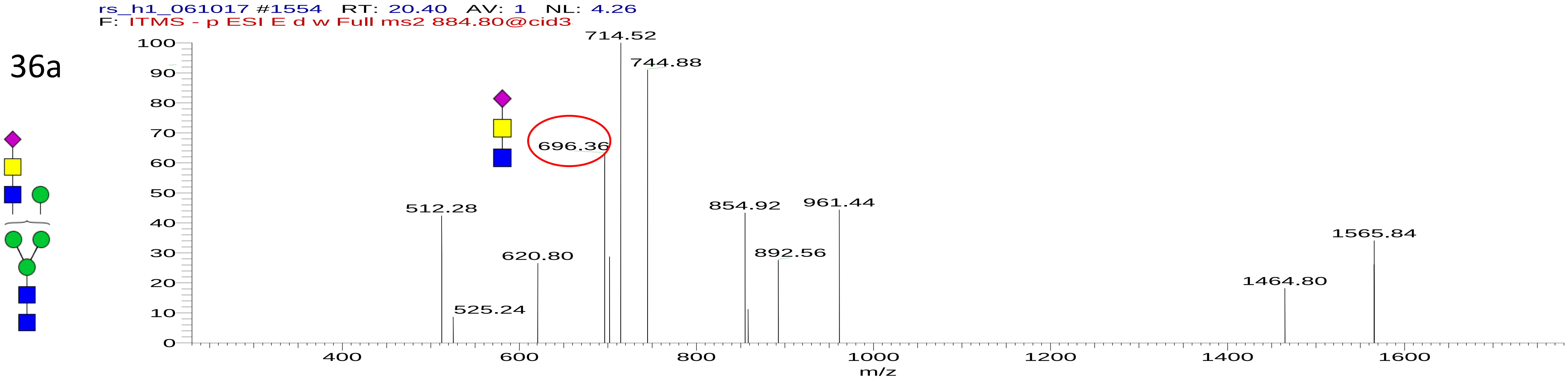

36b

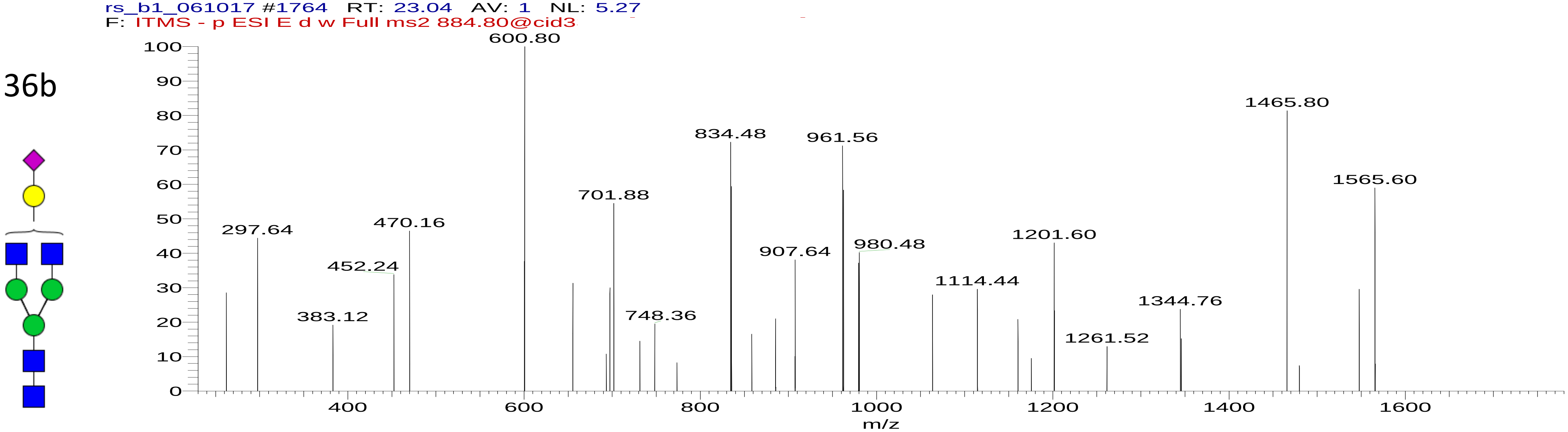

GlytouCan ID: G62446JD

CID-MS/MS
(m/z 884.80 $^{2-}$)

36c

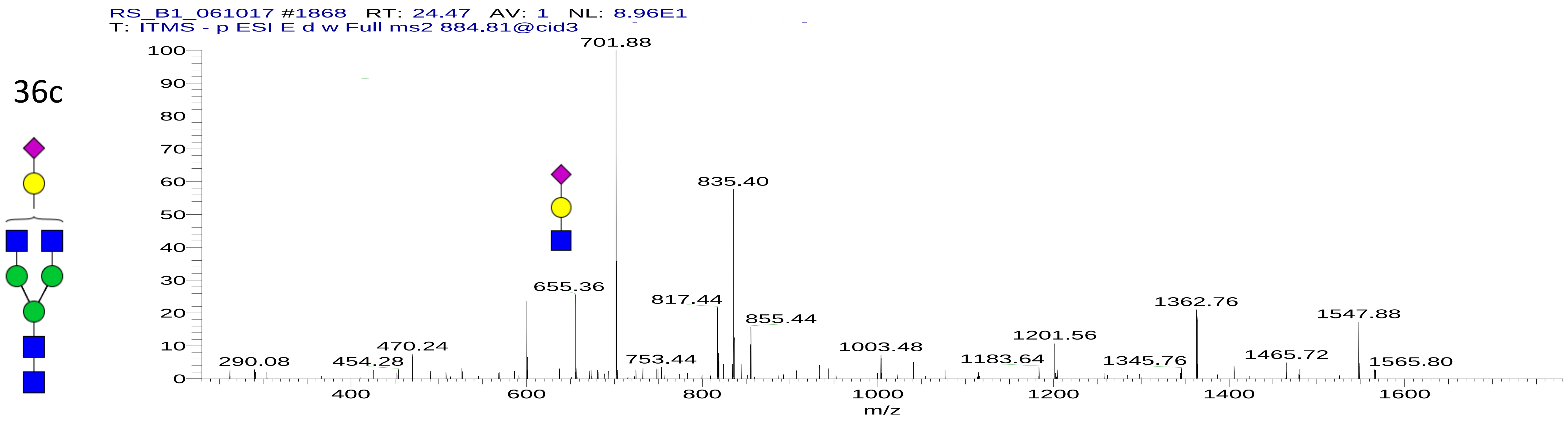

36d

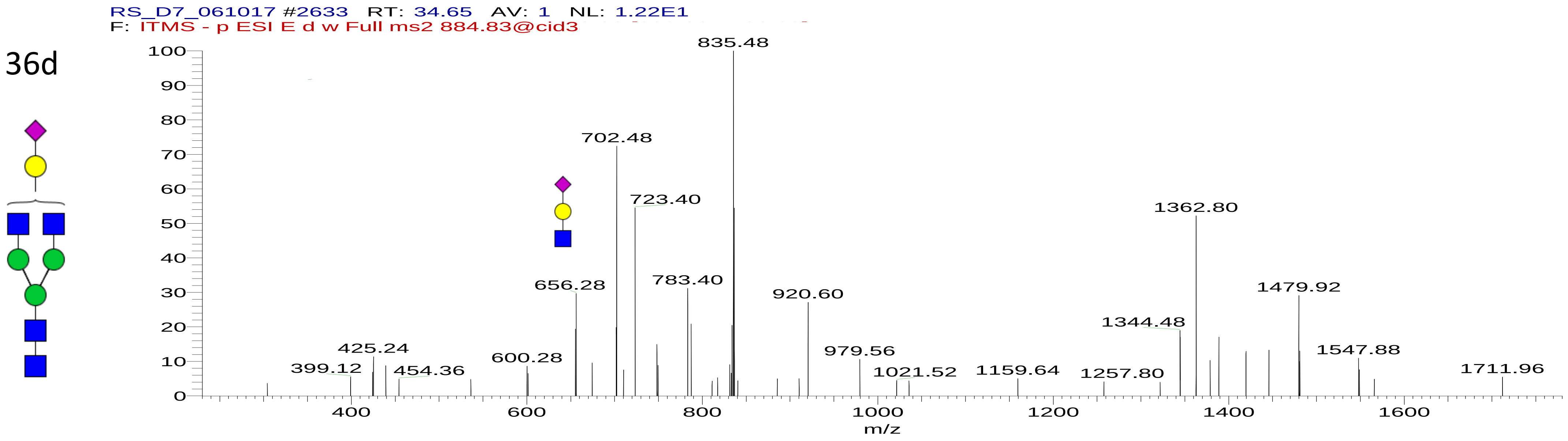

(Hex)1 (HexNAc)3 (Deoxyhexose)1 + (Man)3(GlcNAc)2
GlytouCan ID: G64661YG, G11652JU
[M-2H] 913.82

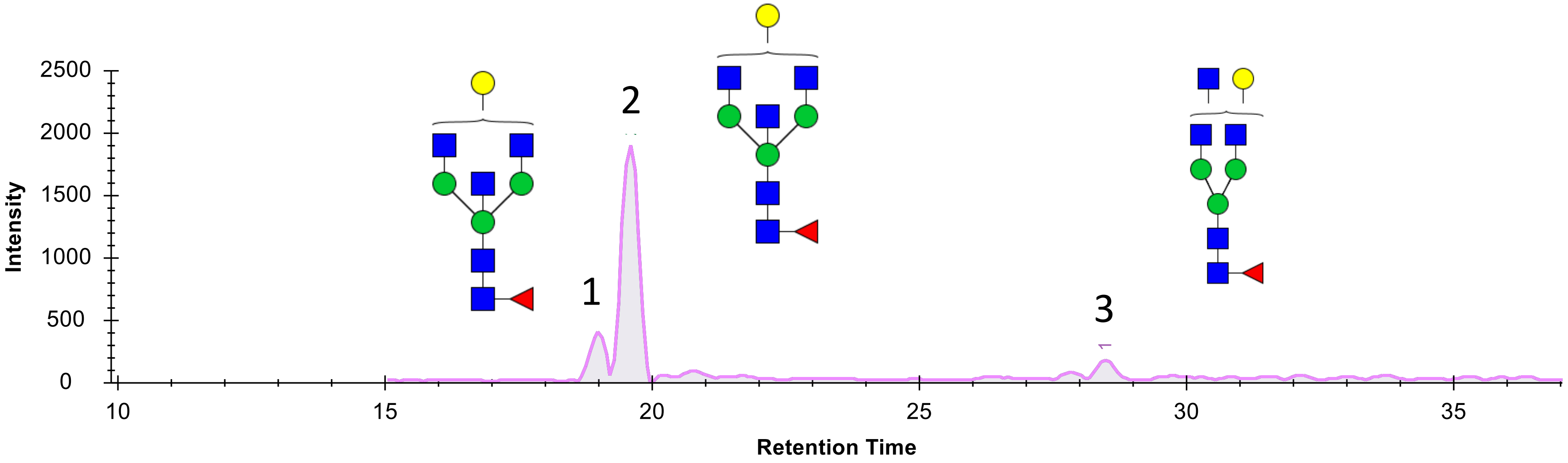

1
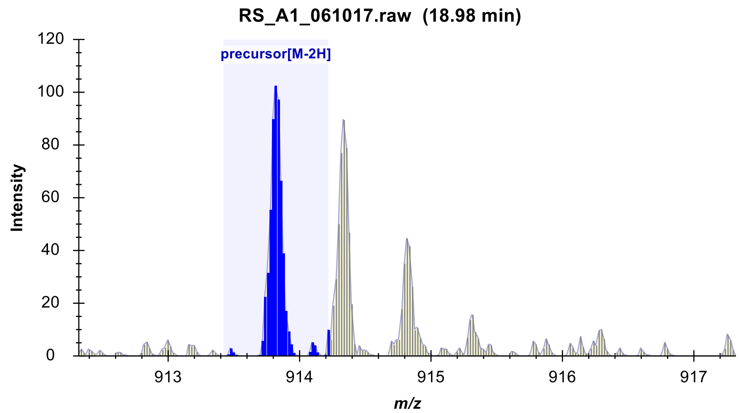

37a

2
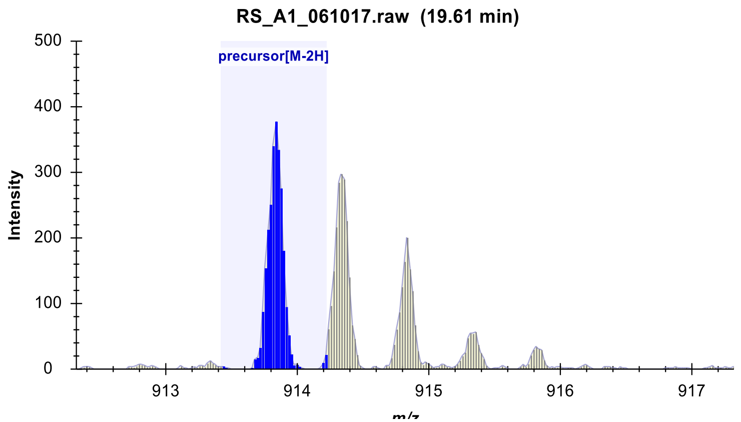

37b

3
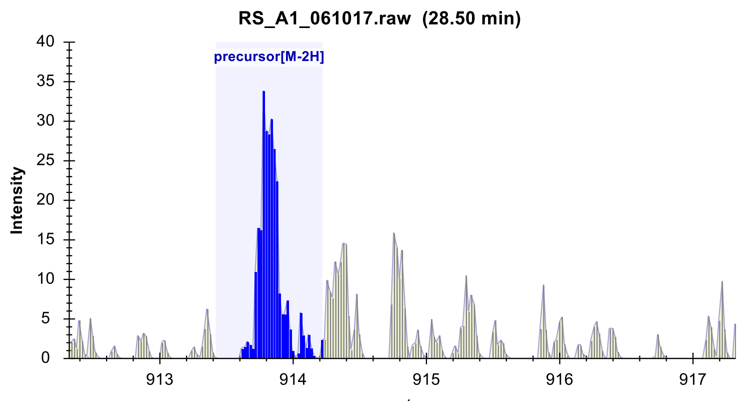

37c

GlytouCan ID:
G64661YG, G11652JU

CID-MS/MS
(*m/z* 913.86 $^{2-}$)

37a

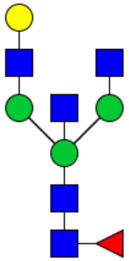

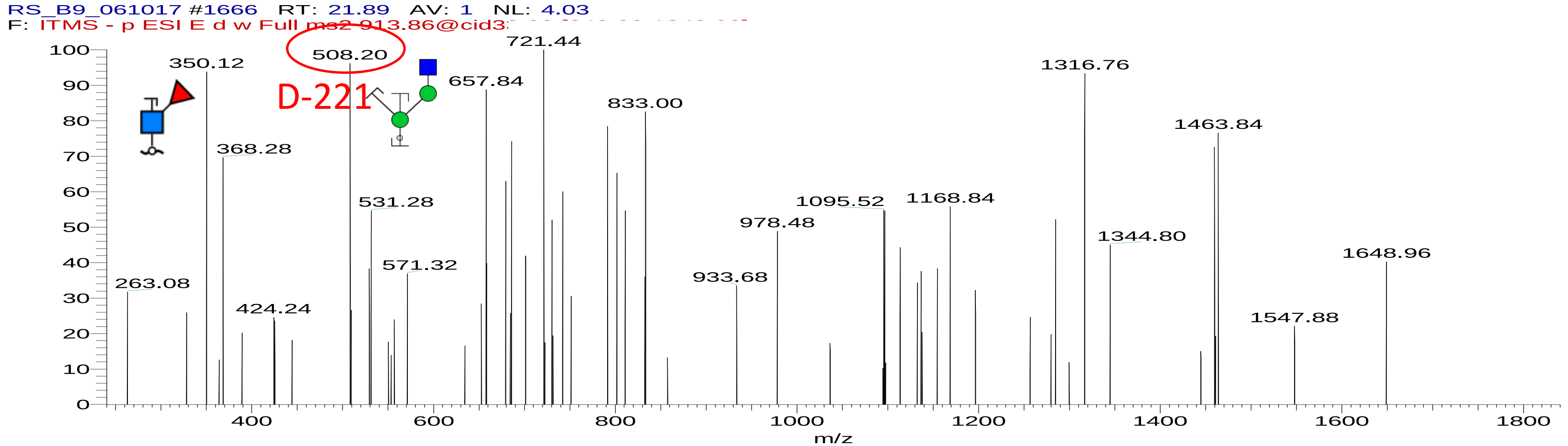

37b

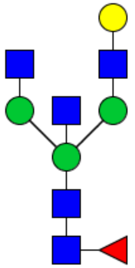

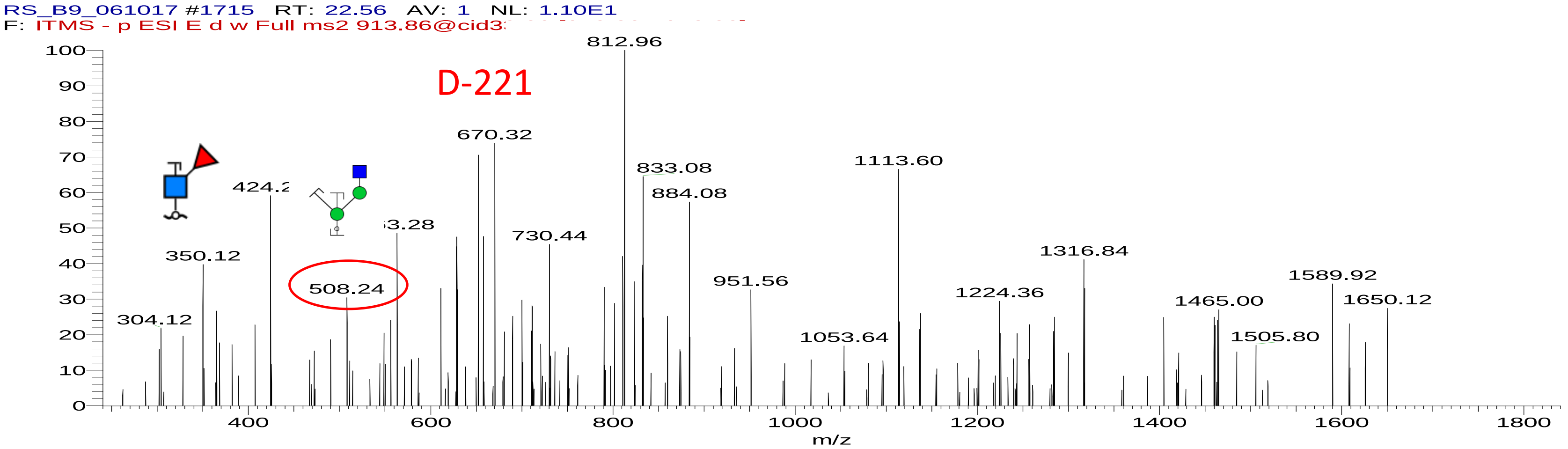

37c

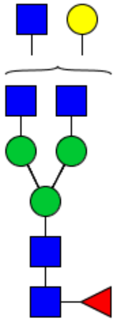

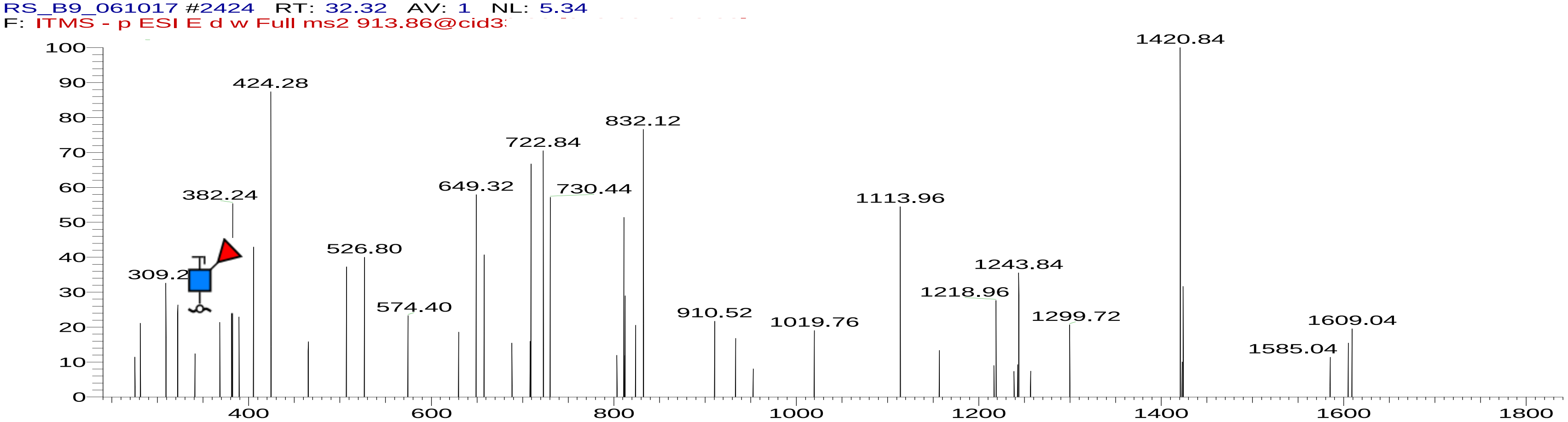

(Hex)2 (HexNAc)3 + (Man)3(GlcNAc)2
GlytouCan ID: G28183PJ
[M-2H] 921.80

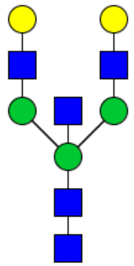

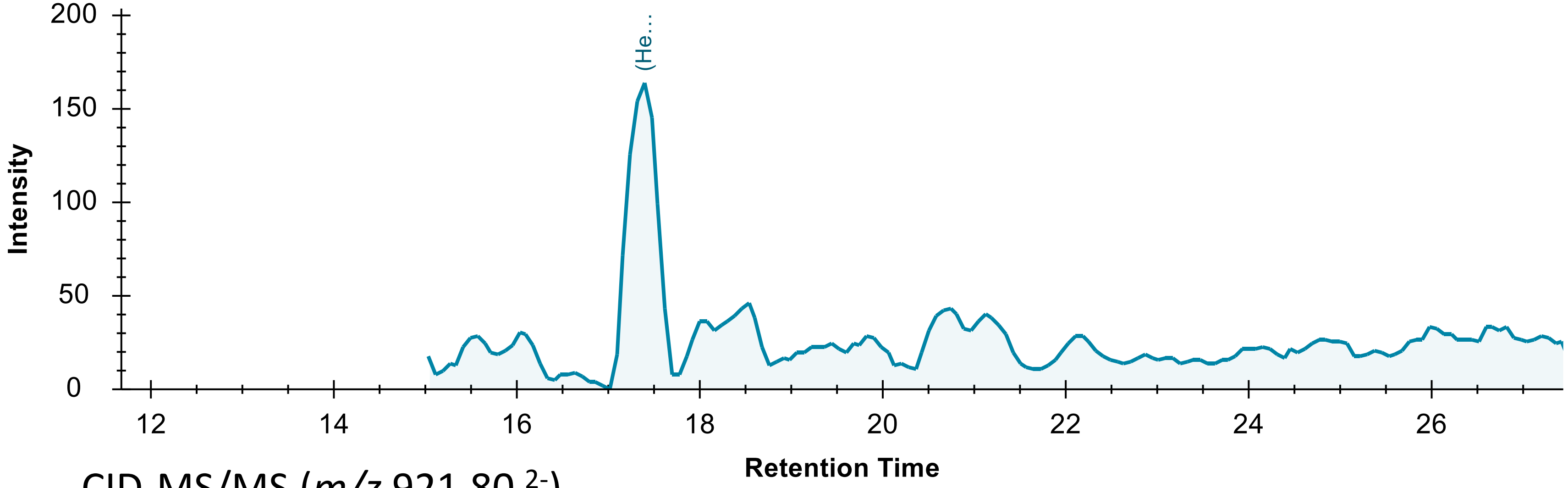

CID-MS/MS (m/z 921.80 $^{2-}$)

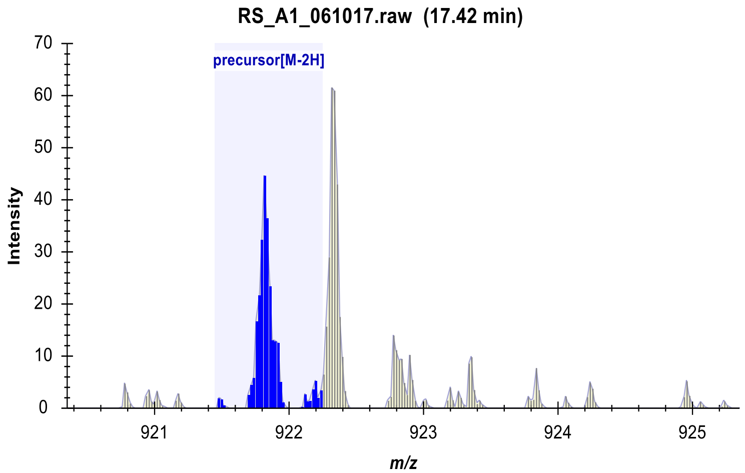

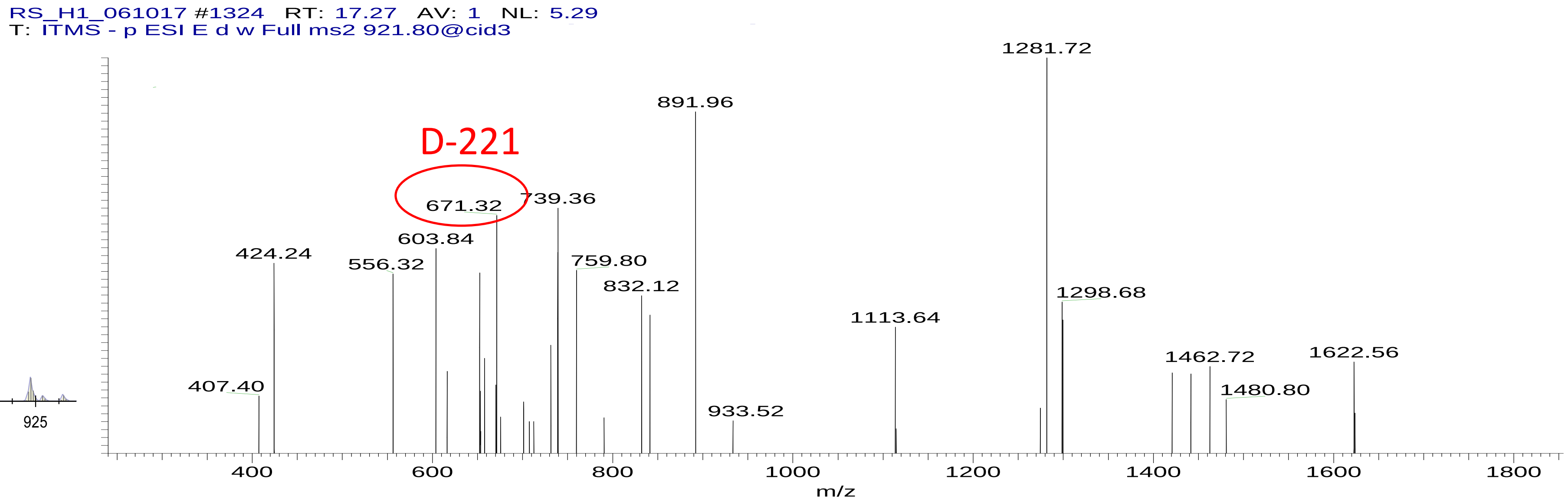

Glycan ID: 38

(Hex)2 (HexNAc)1 (Deoxyhexose)1 (NeuAc)1 +
(Man)3(GlcNAc)2
GlytouCan ID: G84576YN
[M-2H] 937.31

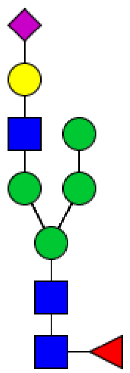

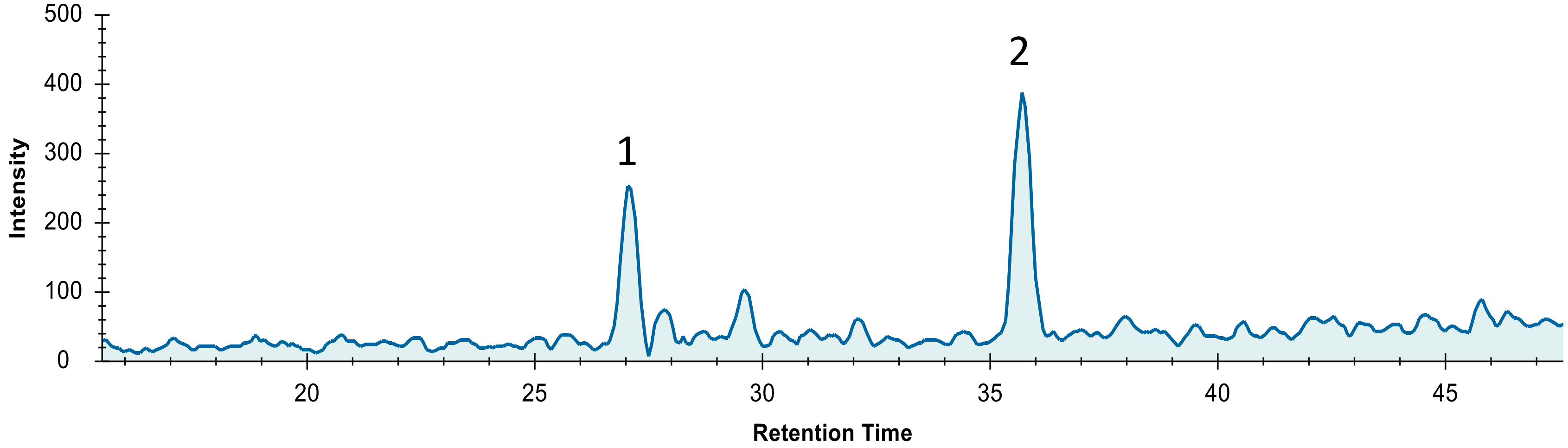

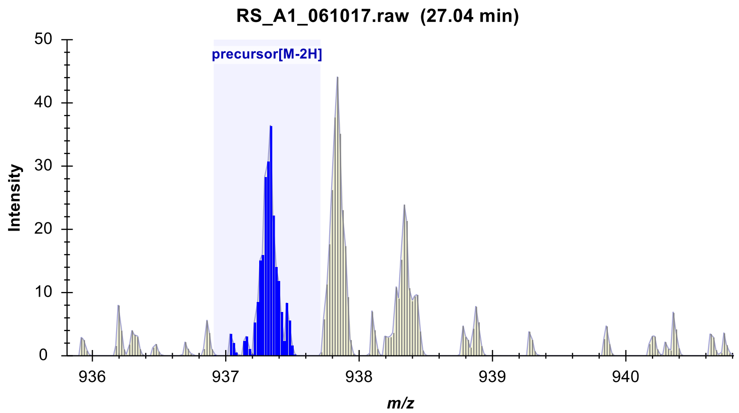

39a

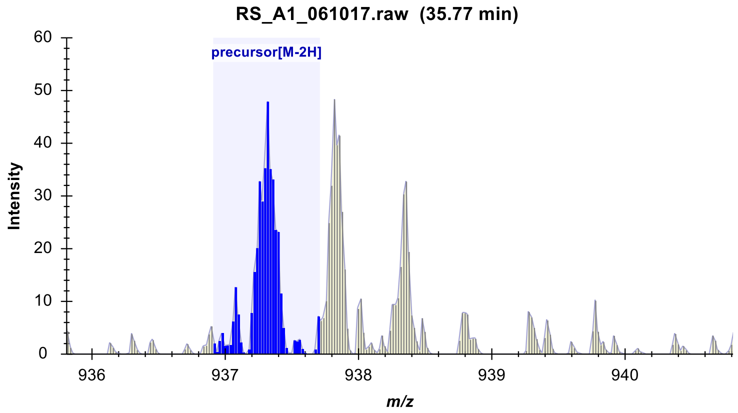

39b

GlytouCan ID: G84576YN

CID-MS/MS

(m/z 937.31 $^{2-}$)

39a

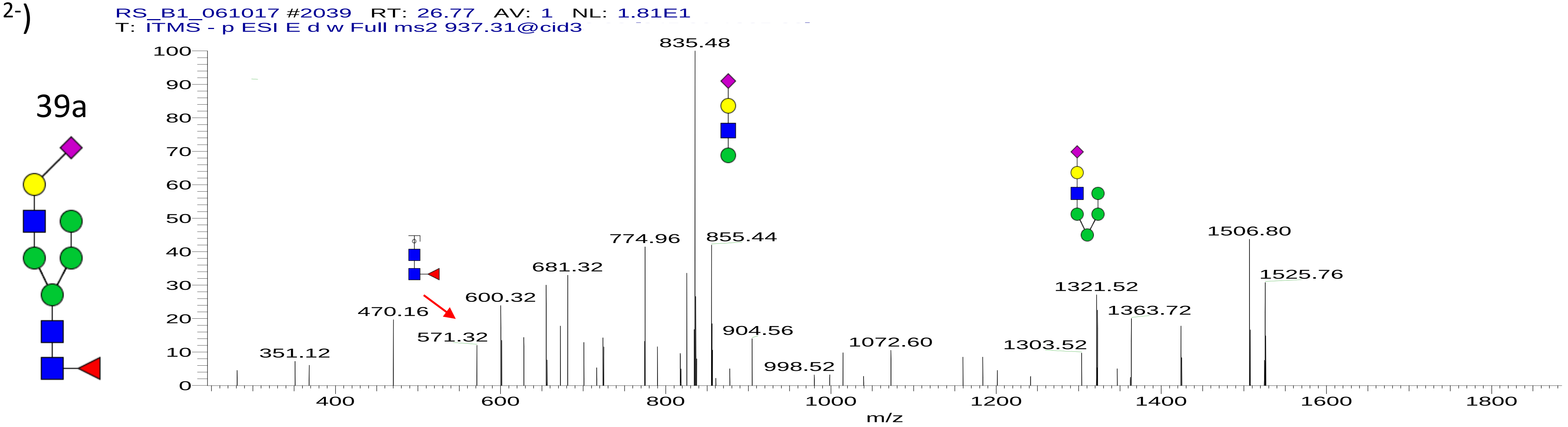

39b

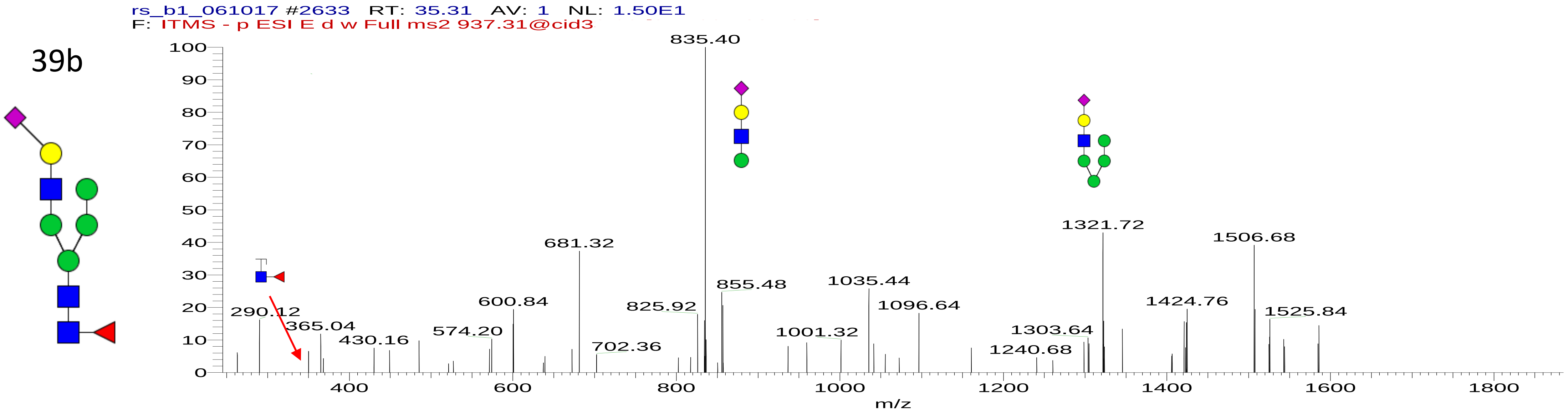

(Hex)3 (HexNAc)1 (NeuAc)1 + (Man)3(GlcNAc)2

GlytouCan ID: G74784VI
[M-2H] 945.33

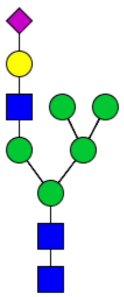

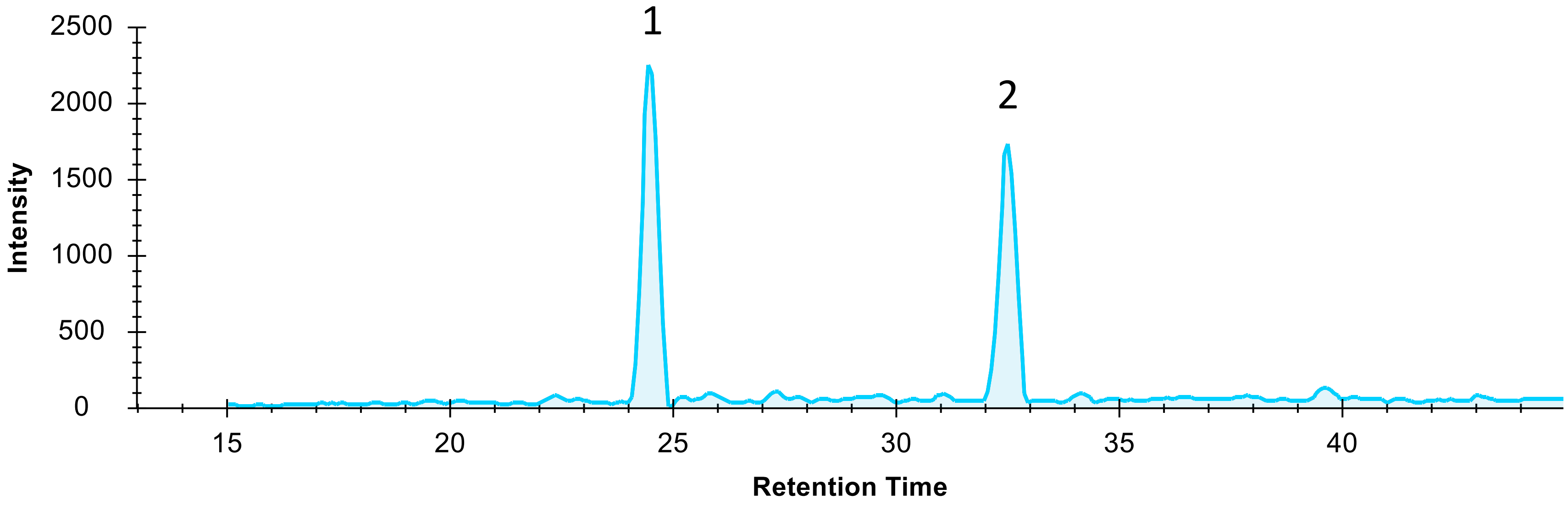

1

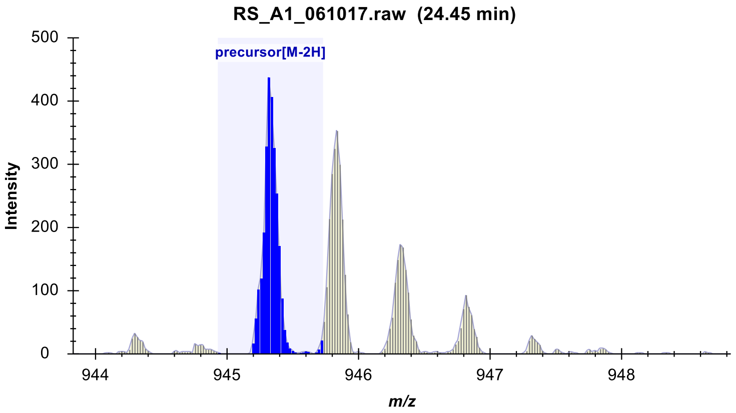

40a

2

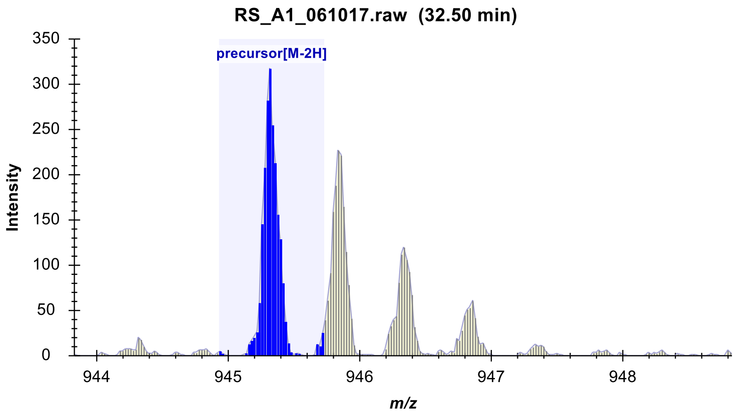

40b

GlytouCan ID: G74784VI

CID-MS/MS

(*m/z* 945.33 $^{2-}$)

40a

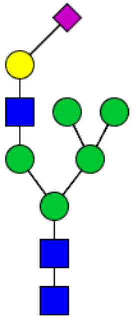

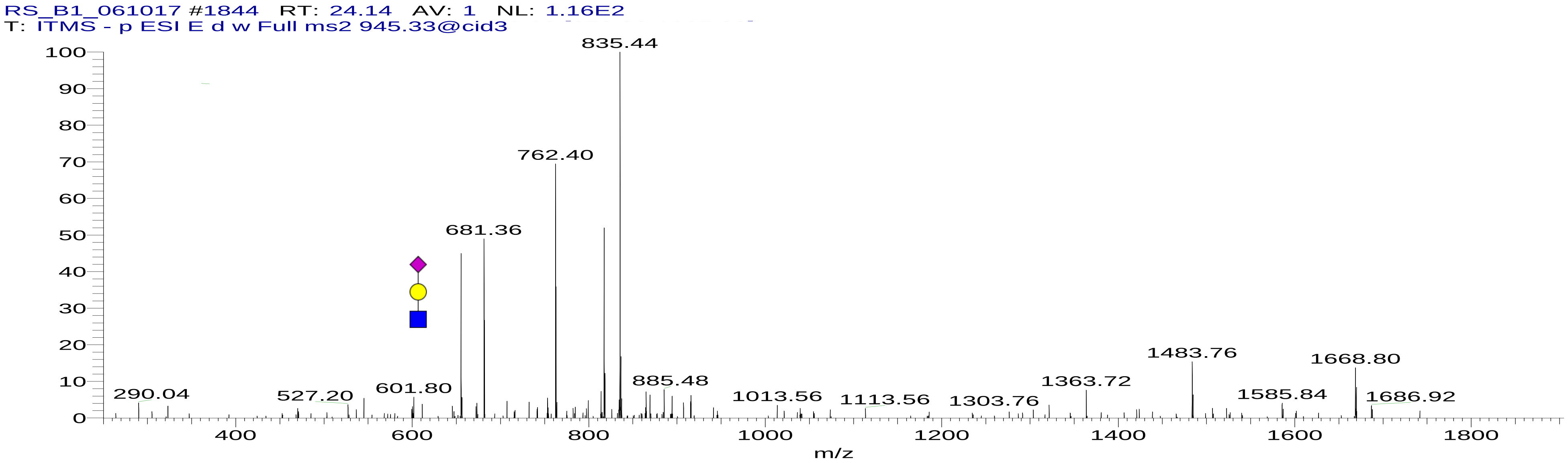

40b

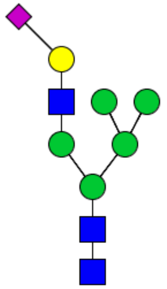

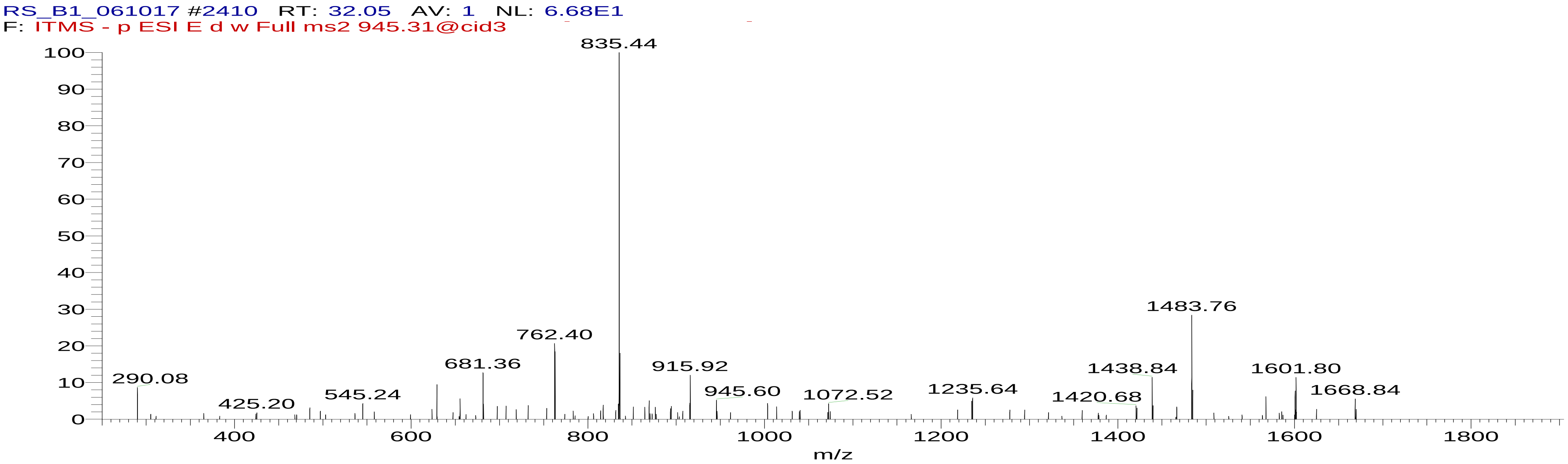

(Hex)4 (HexNAc)1 (Deoxyhexose)1 + (Man)3(GlcNAc)2

GlytouCan ID: G82052AF

[M-2H] 953.82

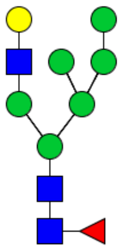

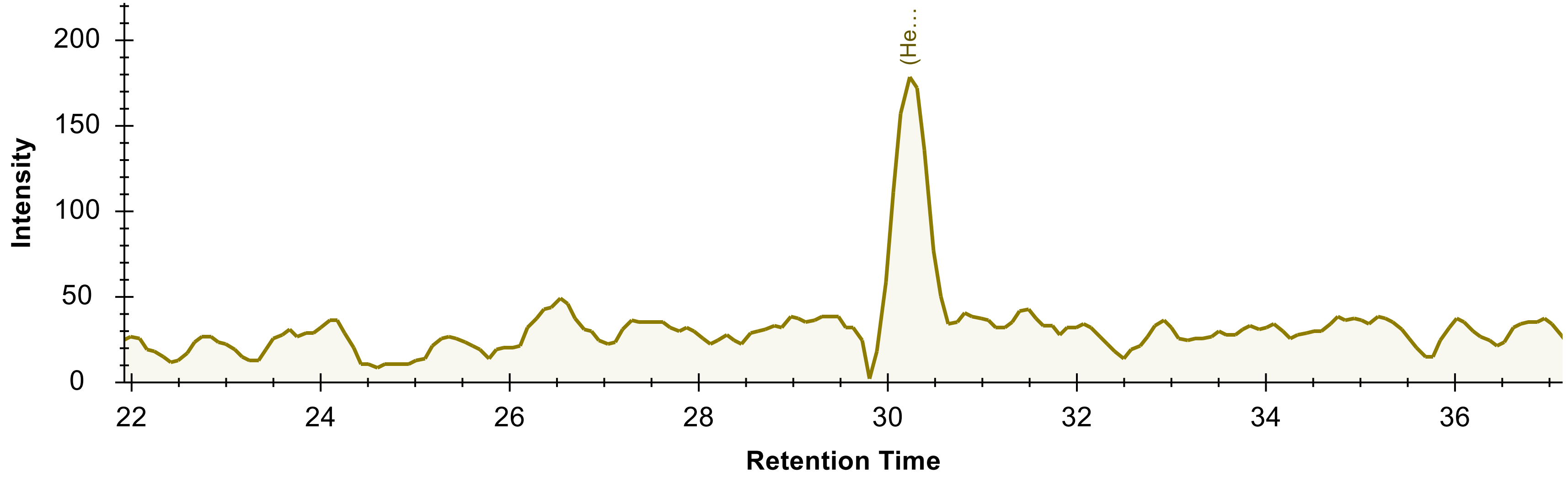

CID-MS/MS (m/z 953.82 $^{2-}$)

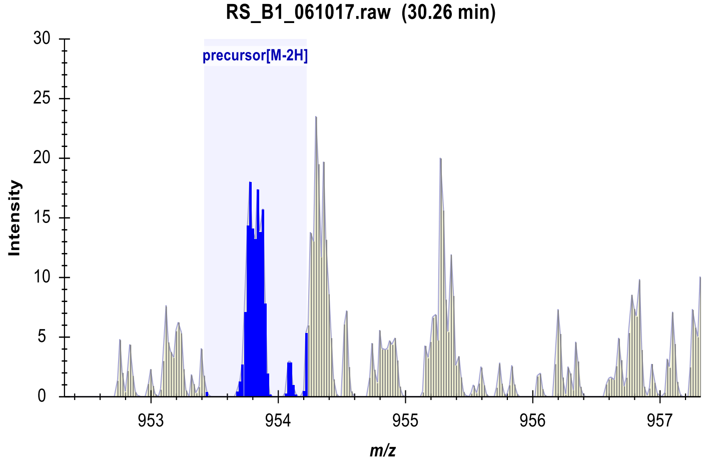

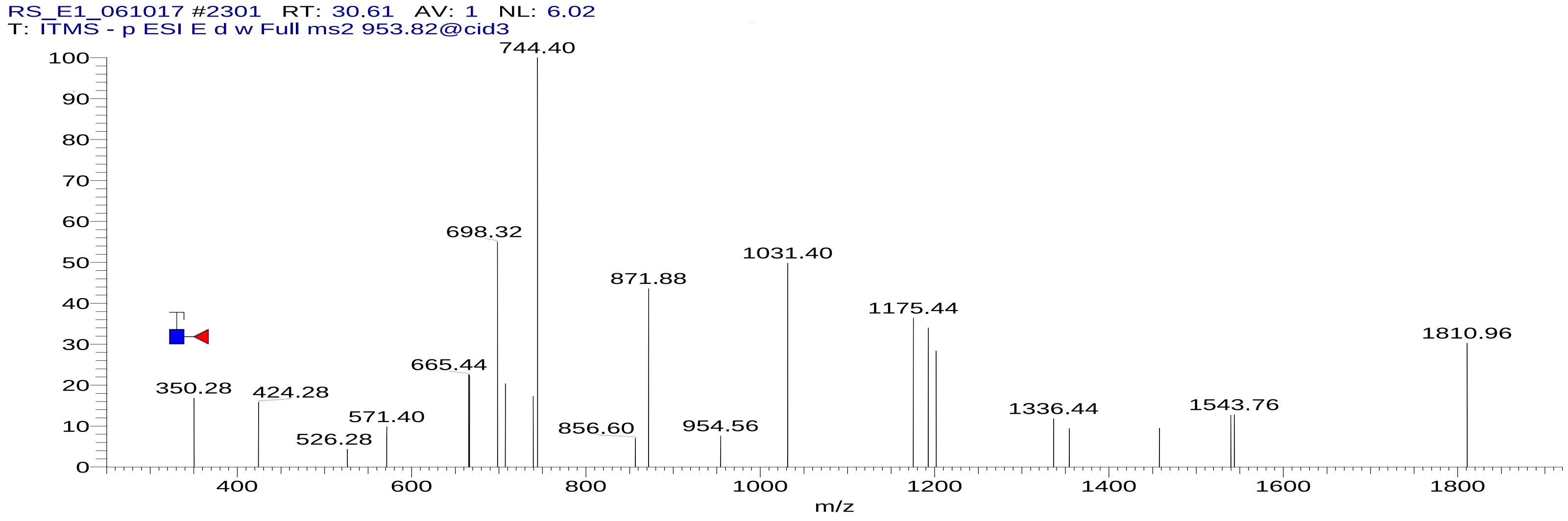

Glycan ID: 41

(Hex)1 (HexNAc)2 (Deoxyhexose)1 (NeuAc)1 + (Man)3(GlcNAc)2

GlytouCan ID: G86681UT

[M-2H] 957.85

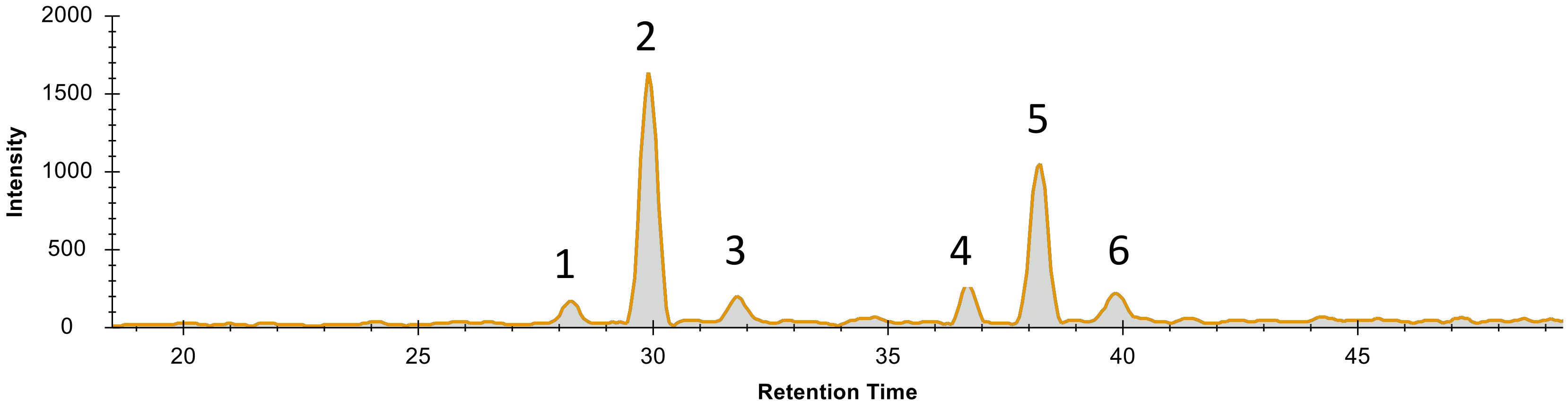

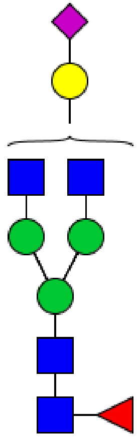

1

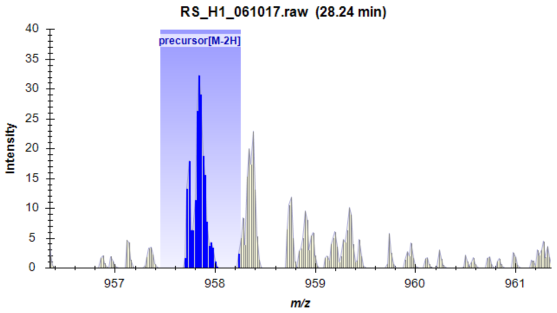

42a

2

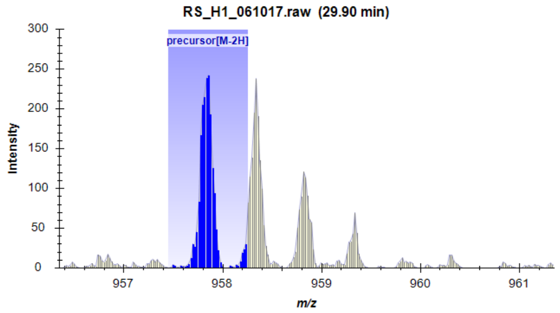

42b

3

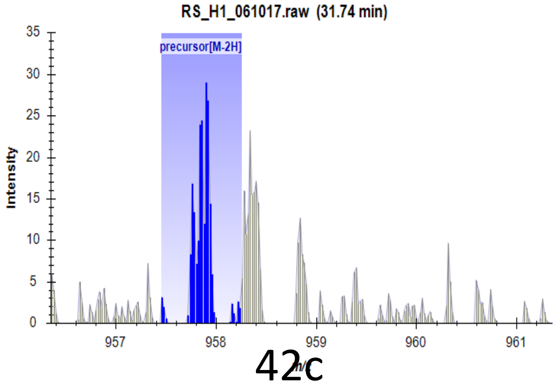

42c

4

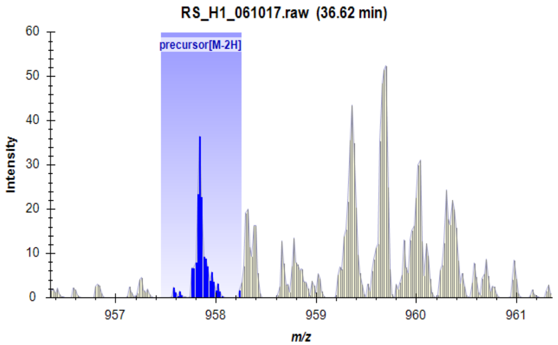

42d

5

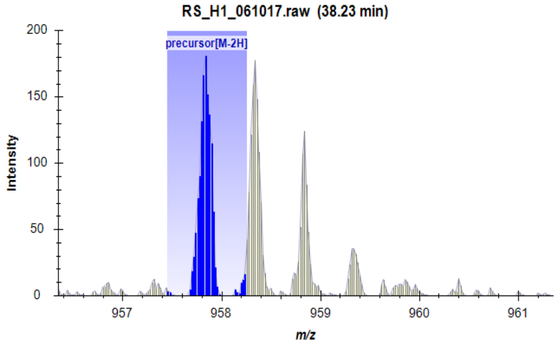

42e

6

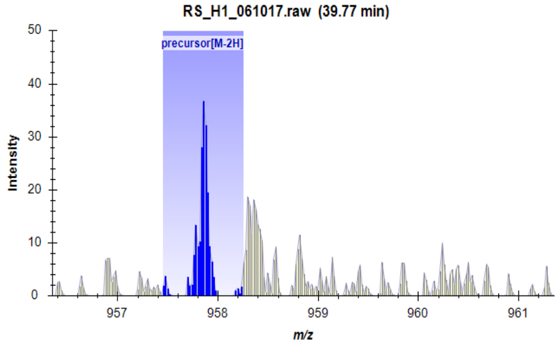

42f

(Hex)1 (HexNAc)2 (Deoxyhexose)1 (NeuAc)1 + (Man)3(GlcNAc)2

GlytouCan ID: G86681UT

[M-2H] 957.85

CID-MS/MS

(*m/z* 957.85 [2-])

42a

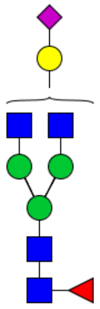

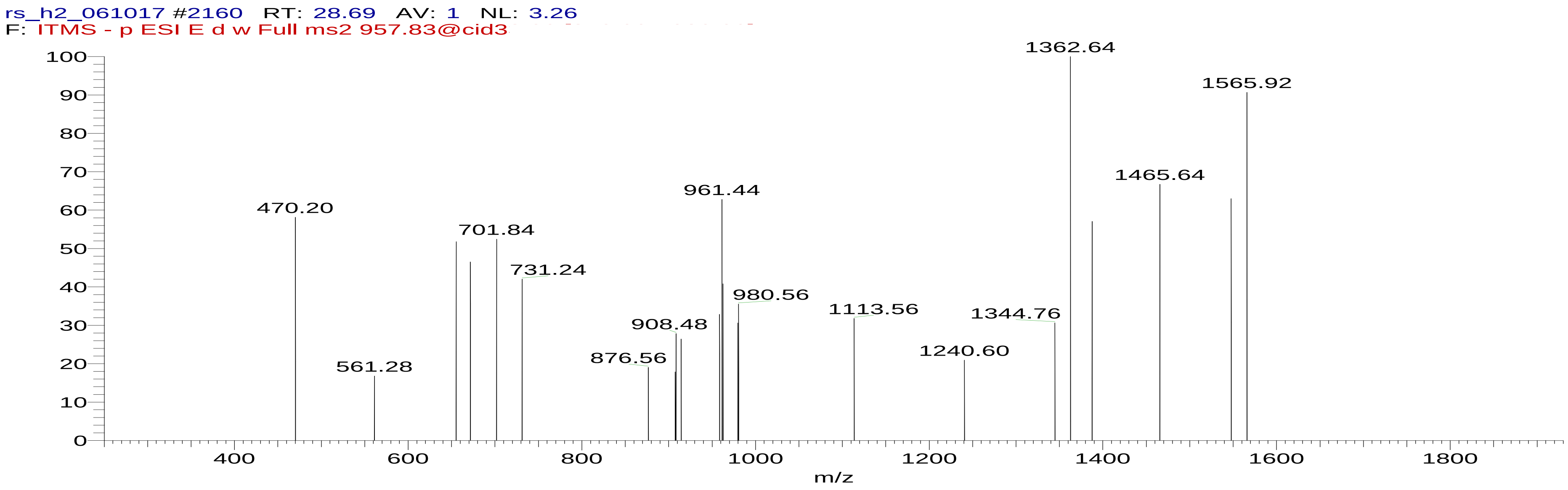

42b

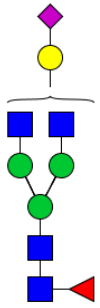

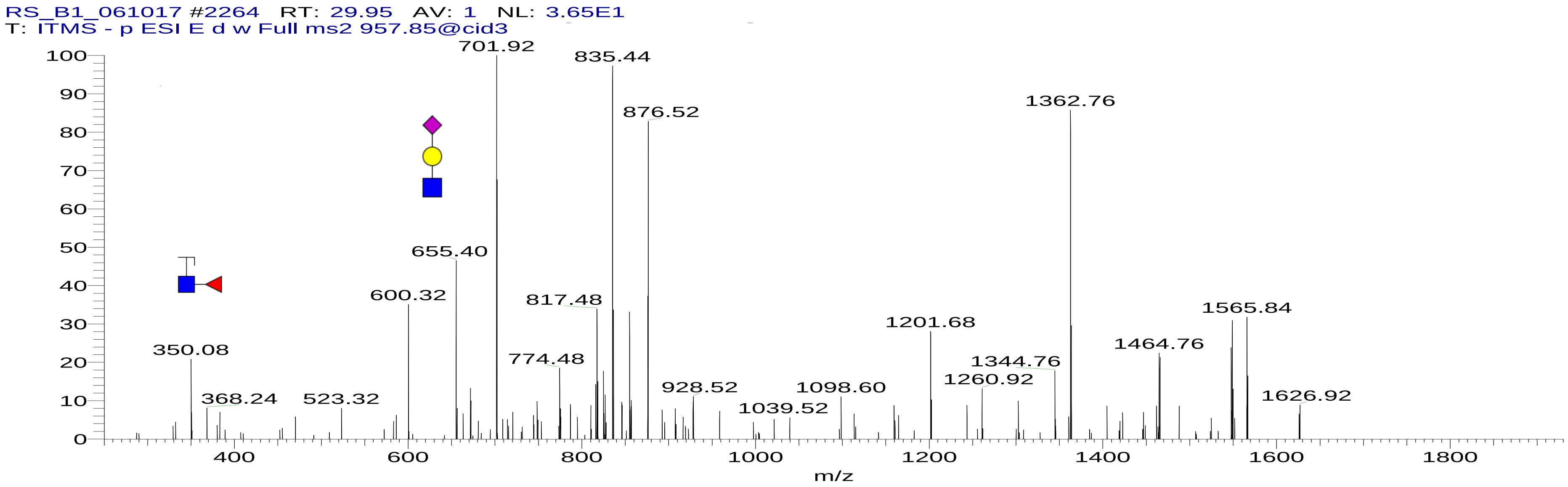

CID-MS/MS
(m/z 957.85 $^{2-}$)

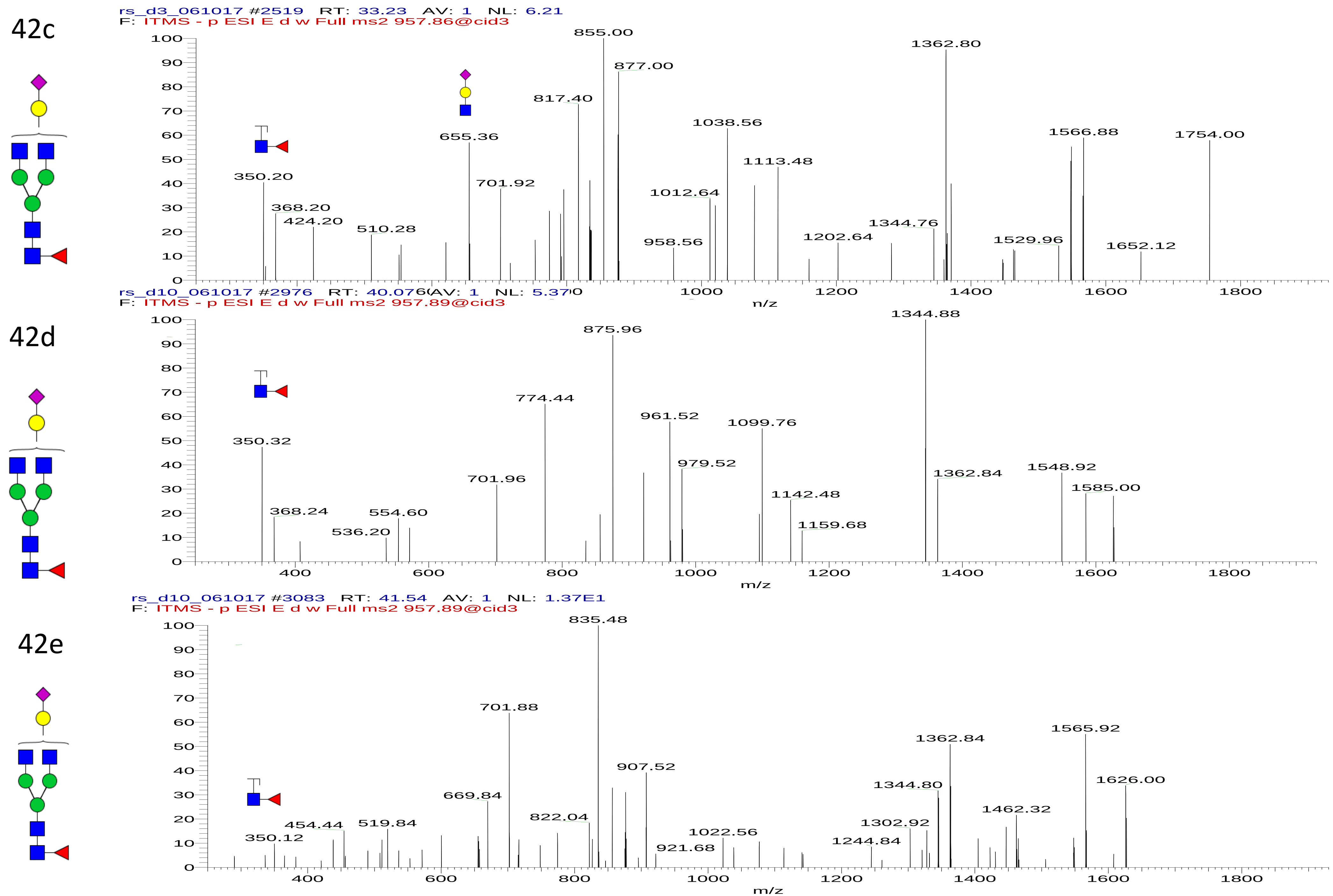

(Hex)2 (HexNAc)2 (NeuAc)1 + (Man)3(GlcNAc)2
GlytouCan ID: G07990UY
[M-2H] 965.86

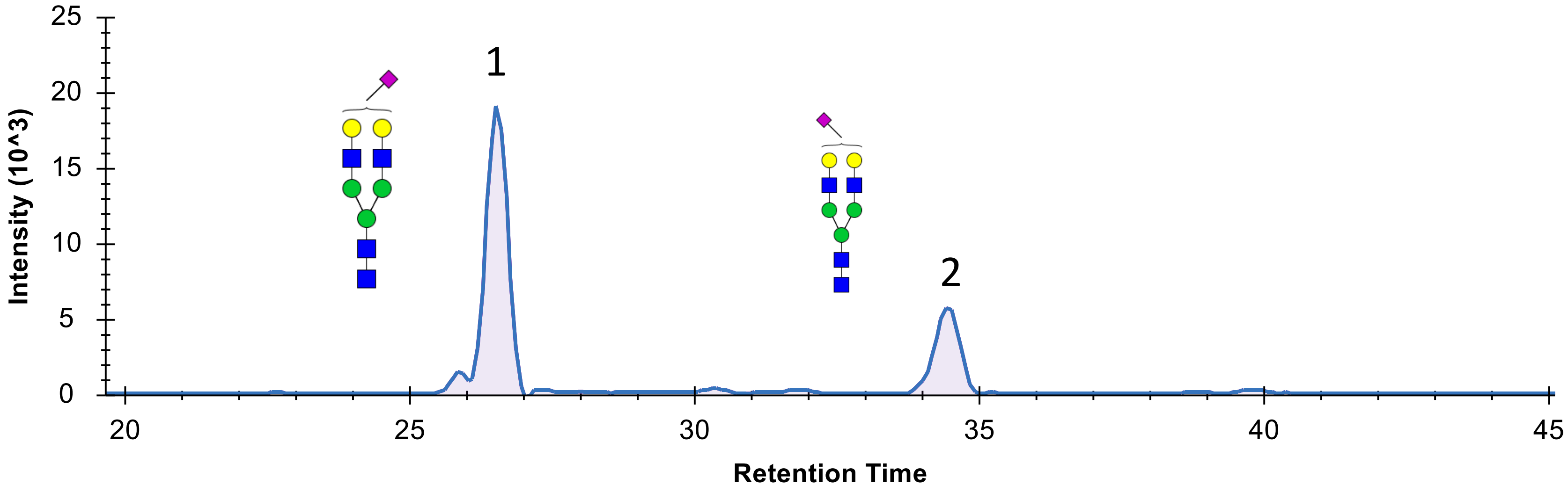

1

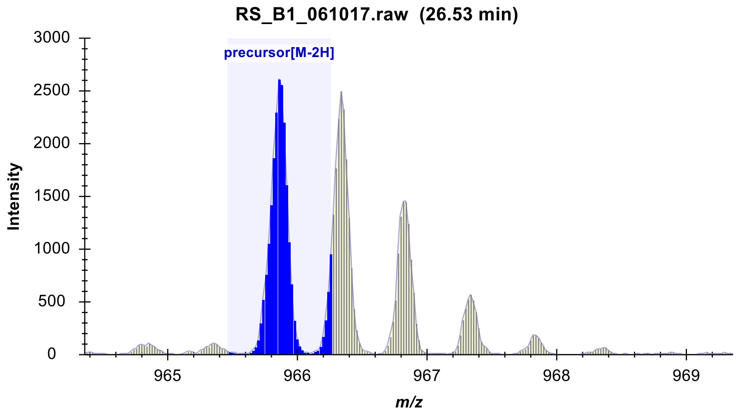

43a

2

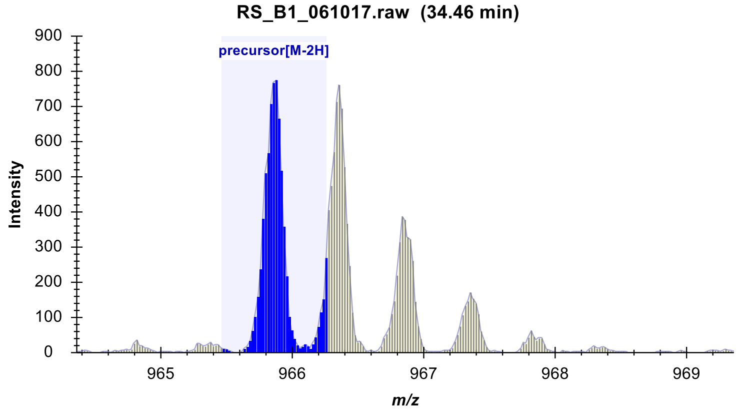

43b

GlytouCan ID: G07990UY

CID-MS/MS

(*m/z* 965.83 $^{2-}$)

43a

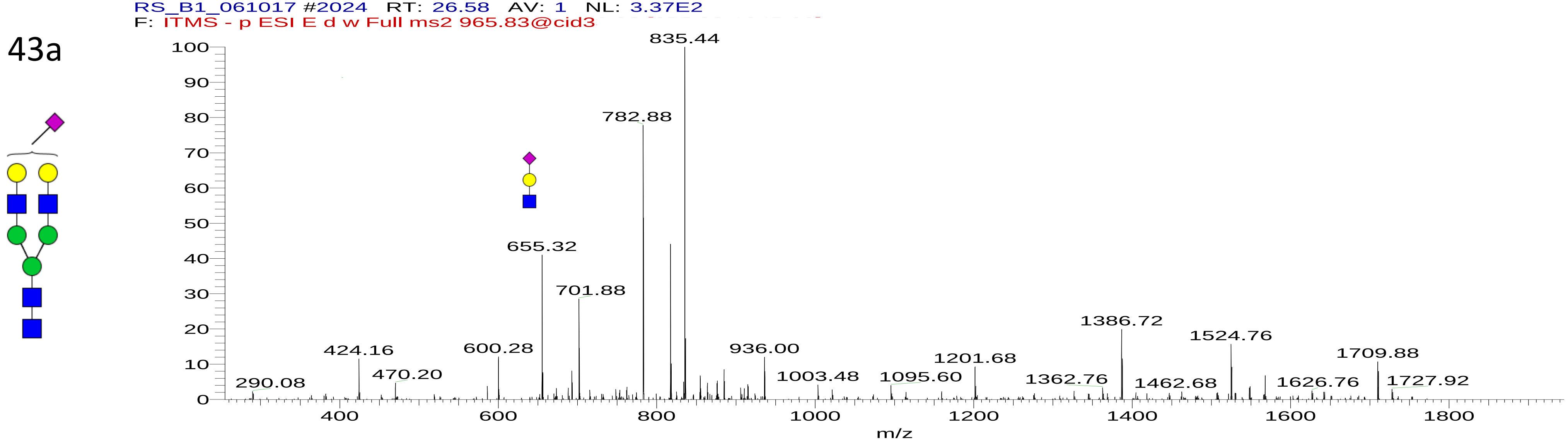

43b

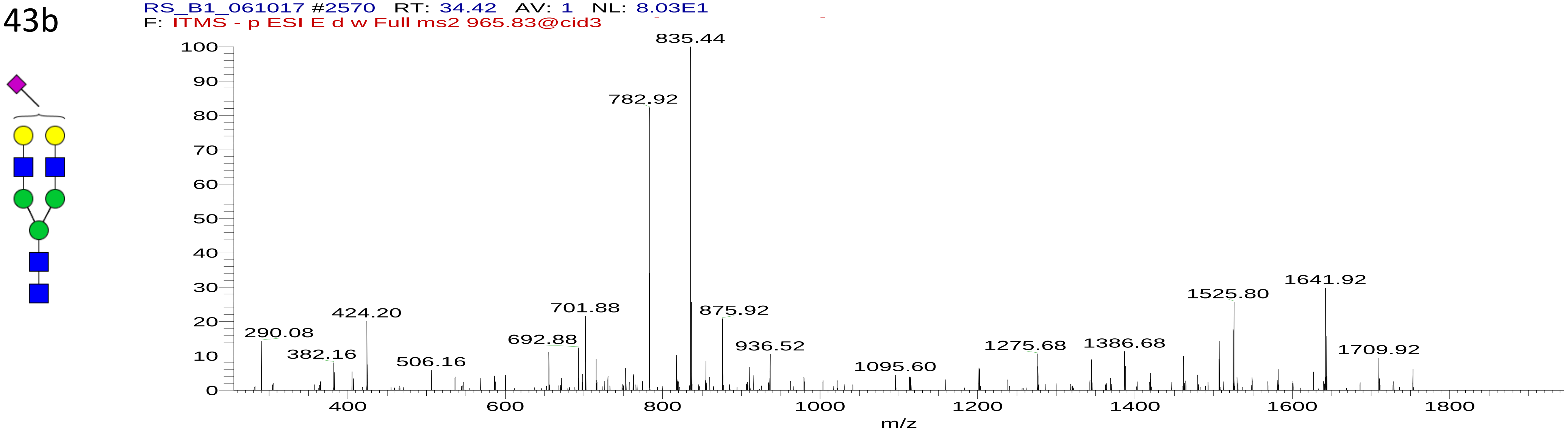

(Hex)1 (HexNAc)3 (NeuAc)1 + (Man)3(GlcNAc)2
GlytouCan ID: G17856XT
[M-2H] 986.35

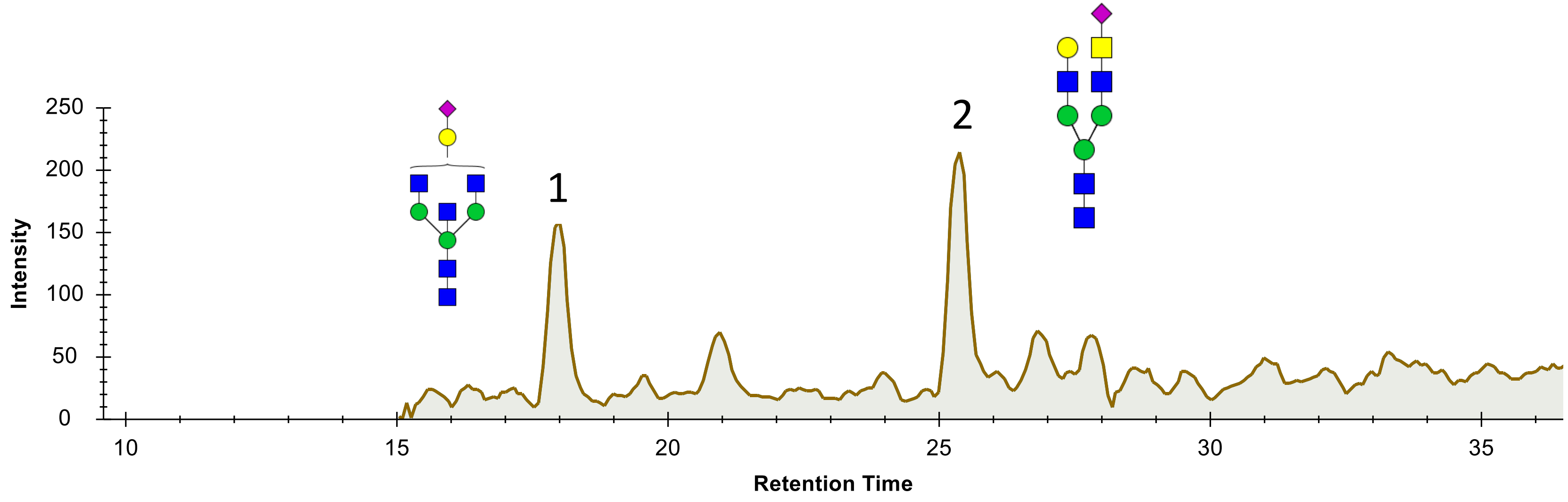

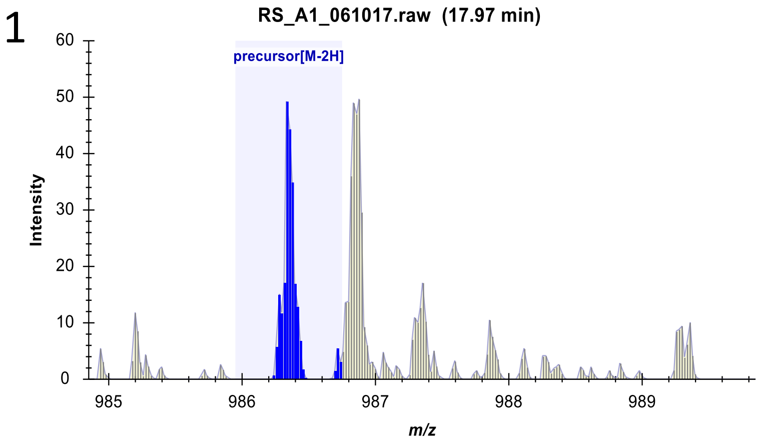

44a

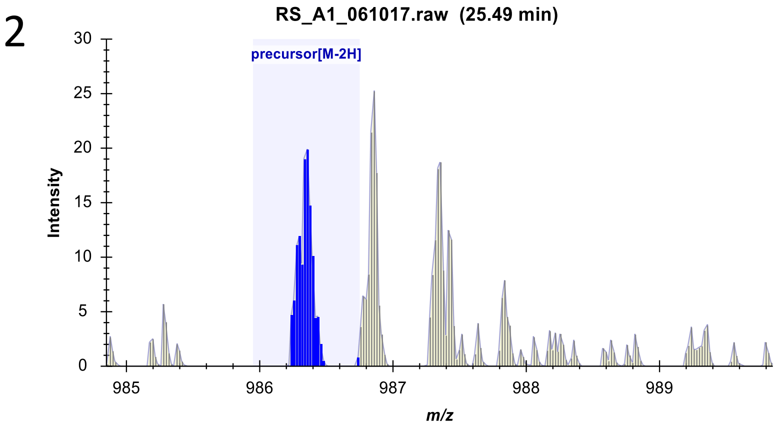

44b

GlytouCan ID: G17856XT

CID-MS/MS

(m/z 986.34 $^{2-}$)

44a

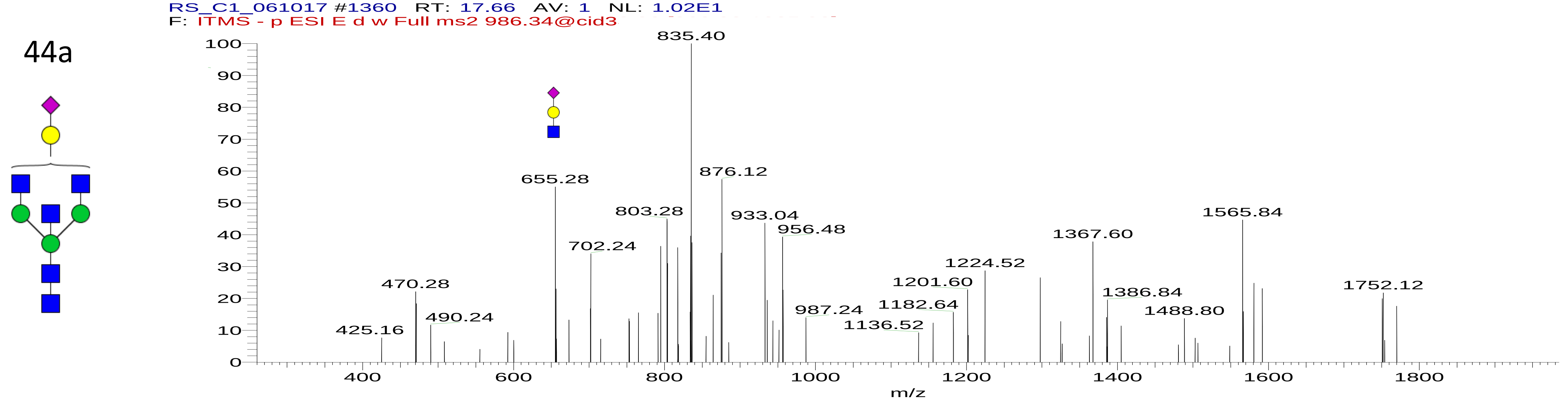

44b

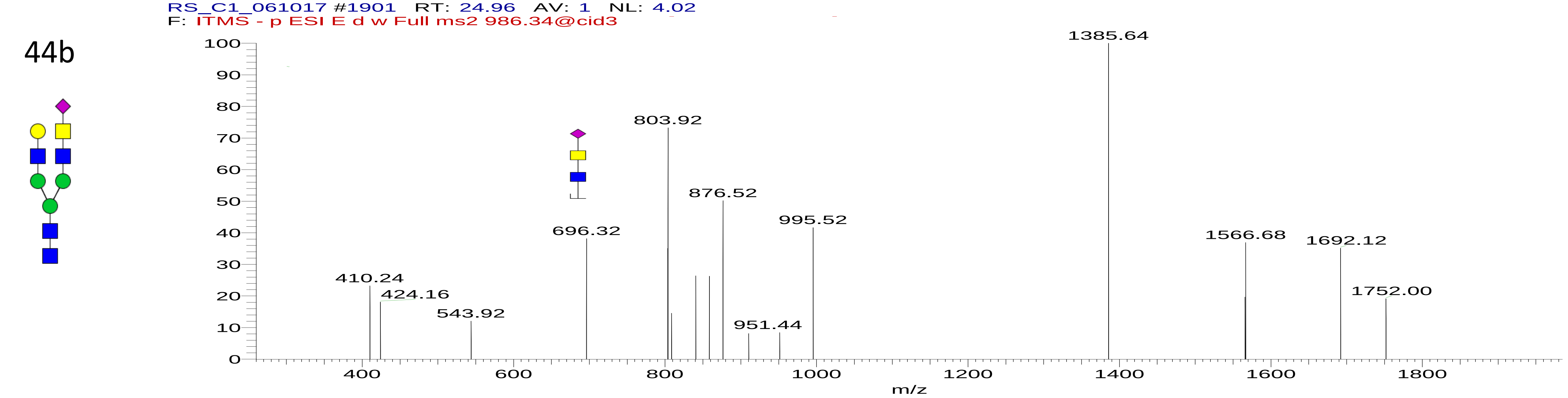

(Hex)2 (HexNAc)3 (Deoxyhexose)1 + (Man)3(GlcNAc)2

Glytoucan ID: G73161WI

[M-2H] 994.88

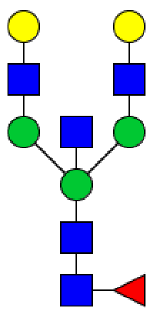

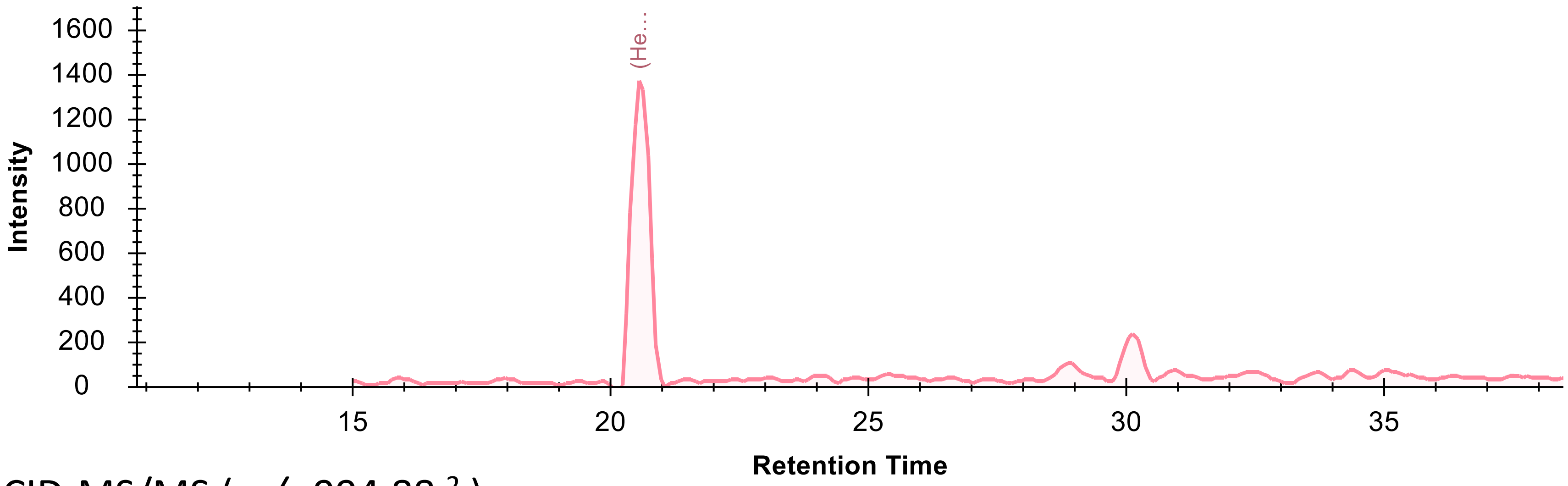

CID-MS/MS (m/z 994.88 $^{2-}$)

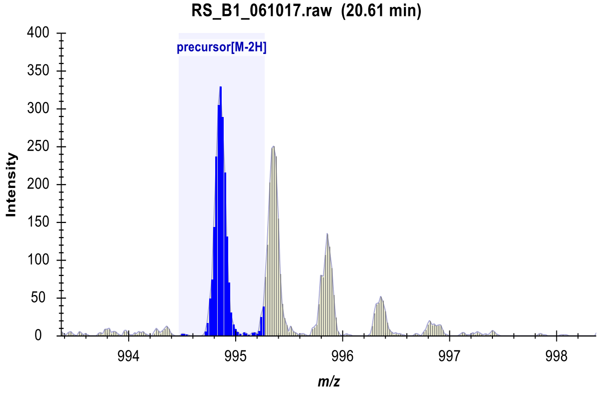

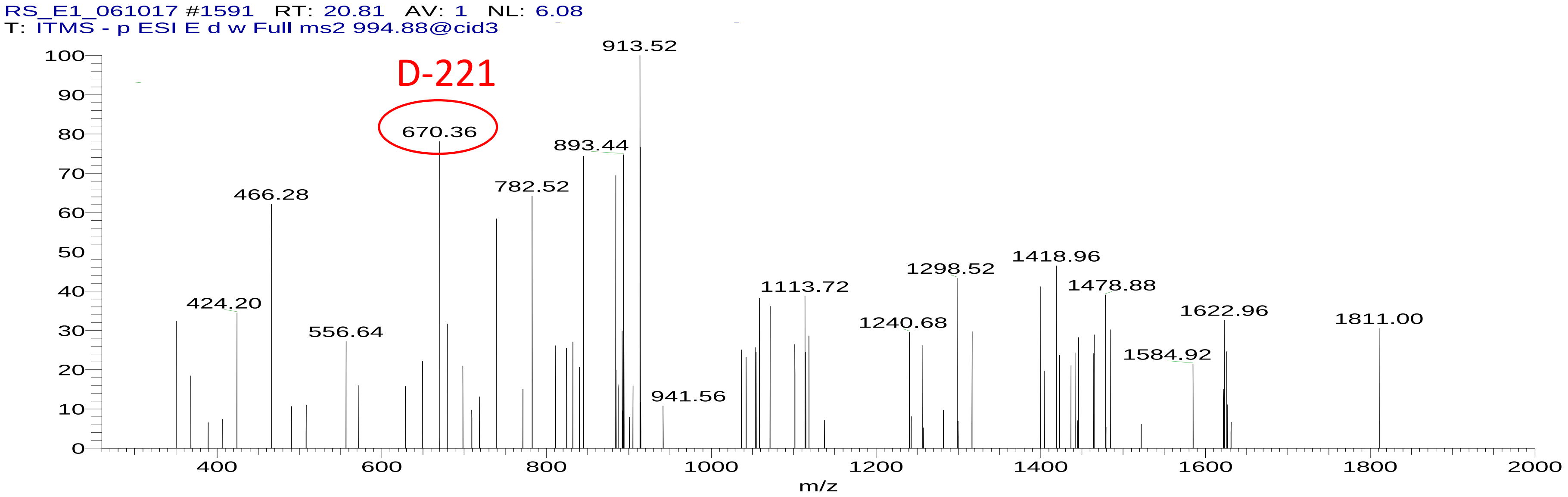

Glycan ID: 45

(Hex)3 (HexNAc)1 (Deoxyhexose)1 (NeuAc)1 + (Man)3(GlcNAc)2

GlytouCan ID: G46683SB

[M-2H] 1018.35

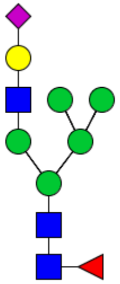

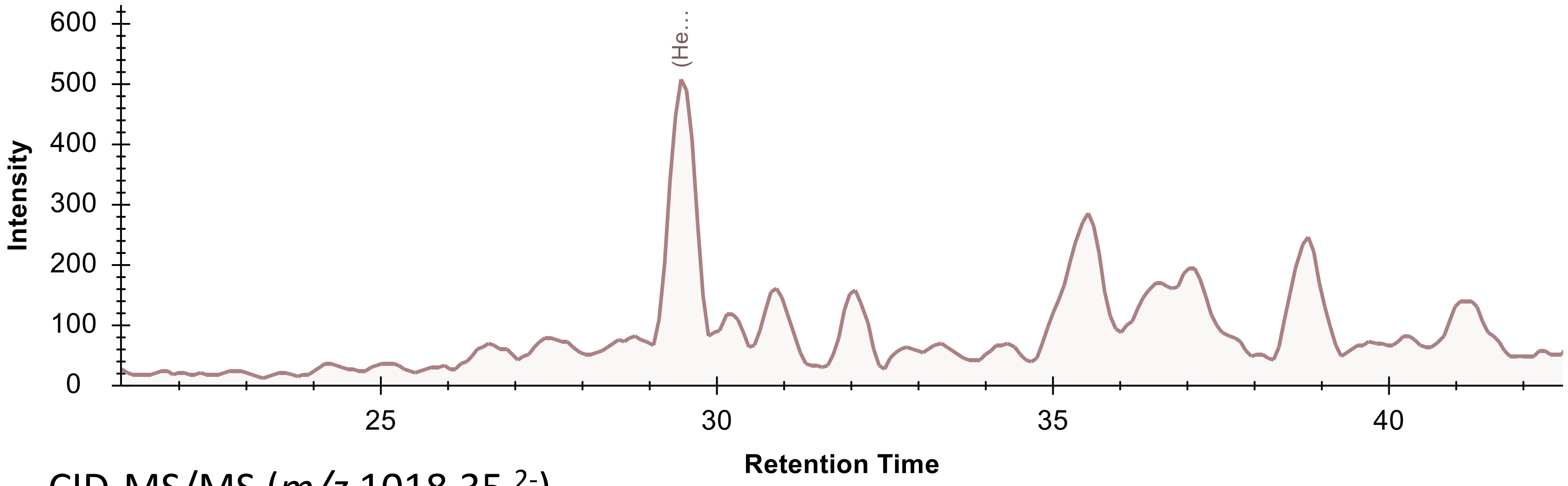

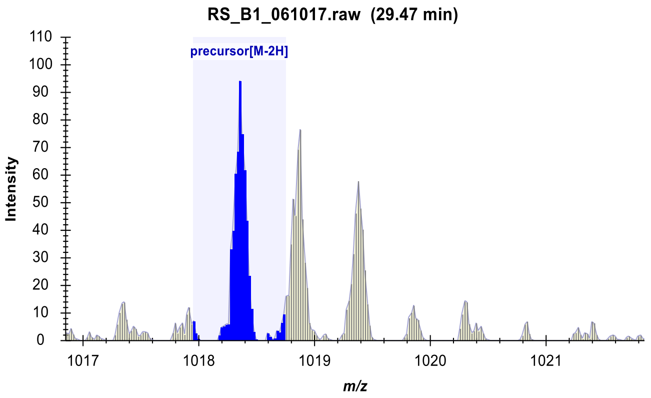

Glycan ID: 46

CID-MS/MS (*m/z* 1018.35 $^{2-}$)

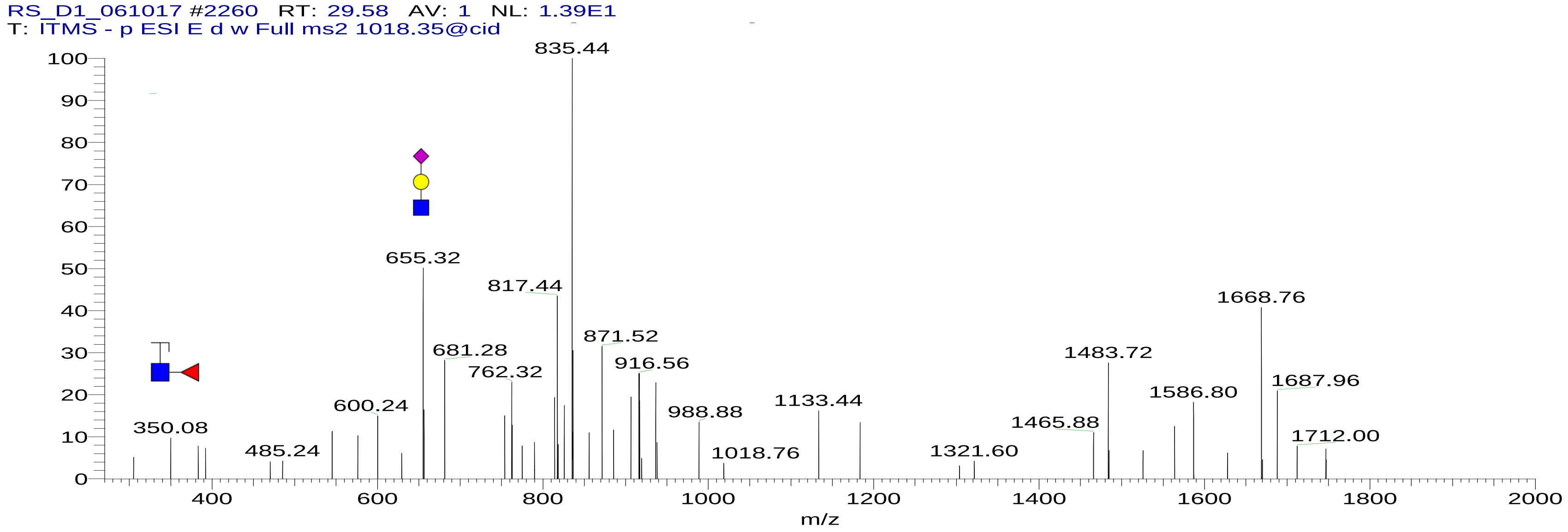

(Hex)2 (HexNAc)2 (Deoxyhexose)1 (NeuAc)1 + (Man)3(GlcNAc)2
GlytouCan ID: G02646YL

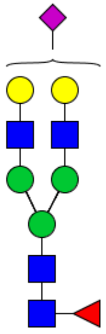

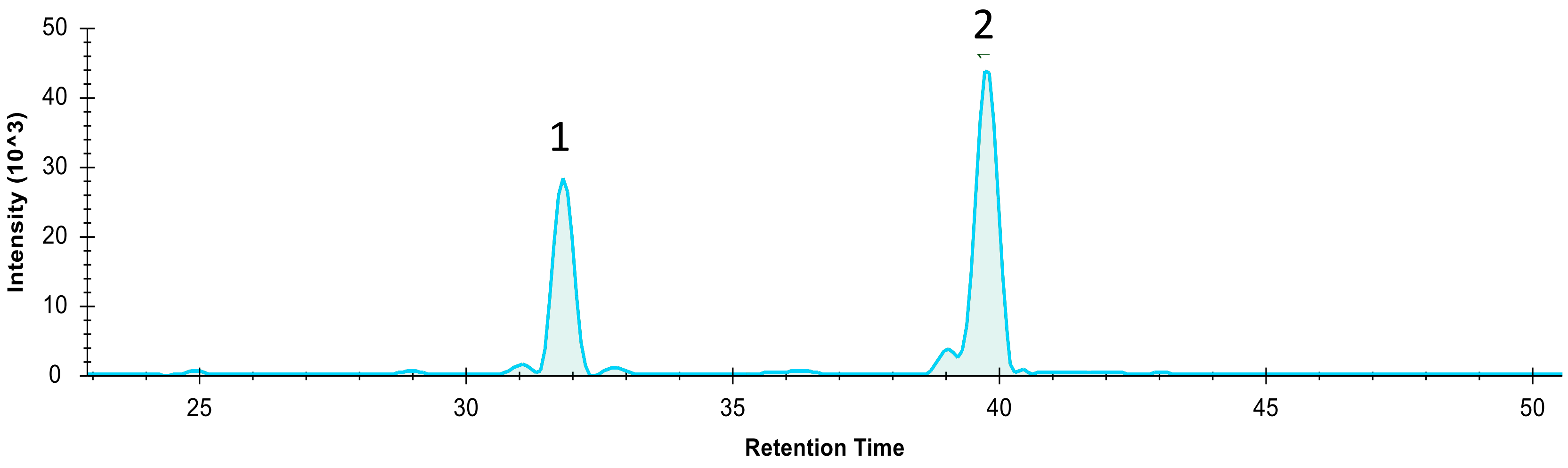

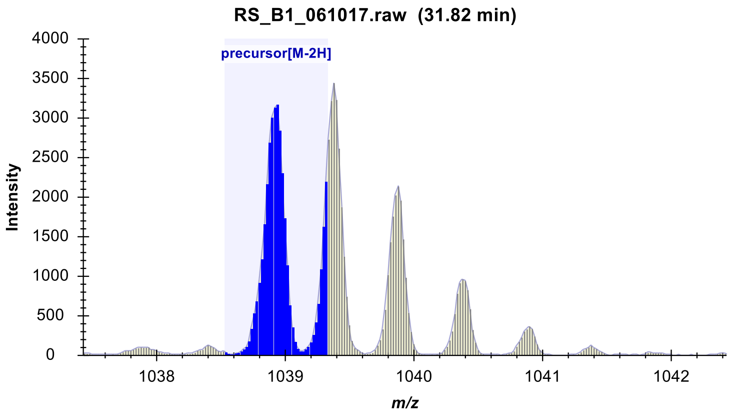

47a

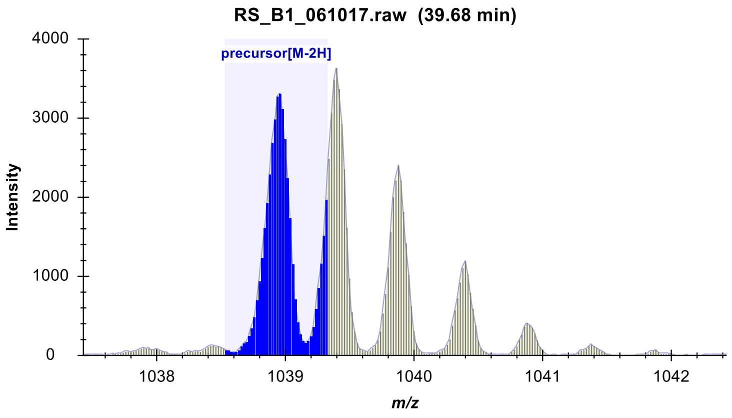

47b

GlytouCan ID: G02646YL

CID-MS/MS
(m/z 1038.78 $^{2-}$)

47a

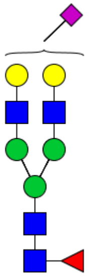

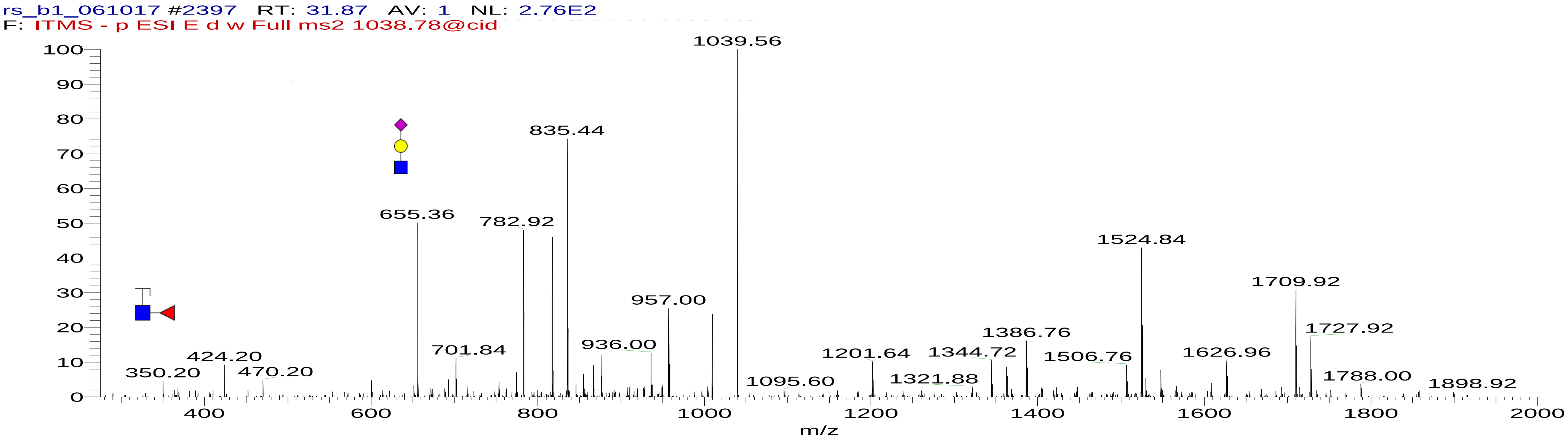

47b

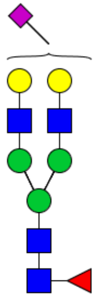

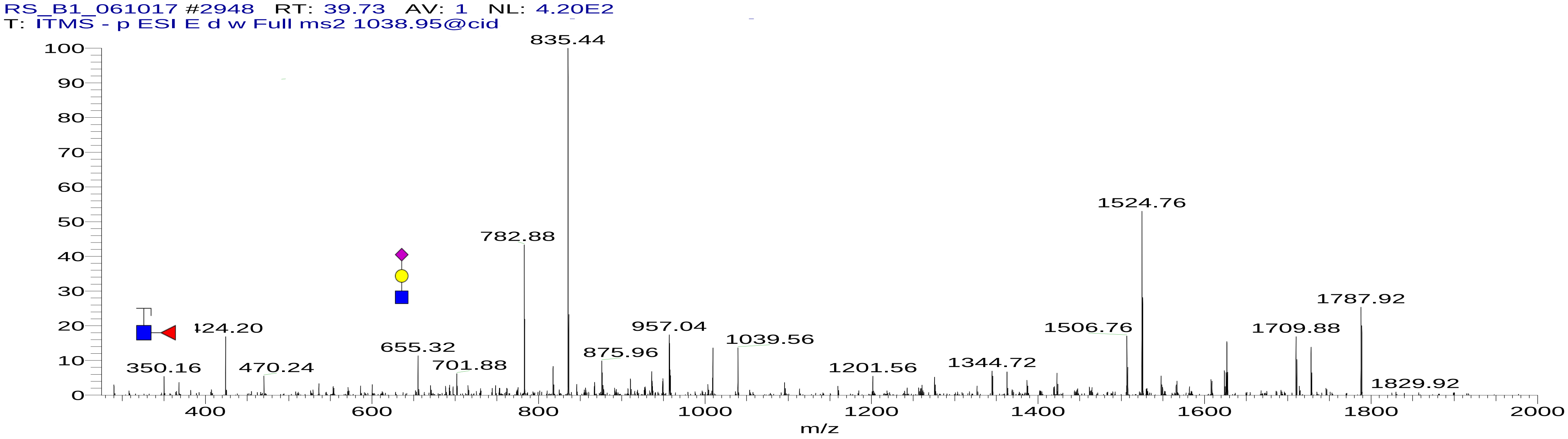

(Hex)1 (HexNAc)3 (Deoxyhexose)1 (NeuAc)1 + (Man)3(GlcNAc)2
GlytouCan ID: G03380EA, G46208AF
[M-2H] 1059.38

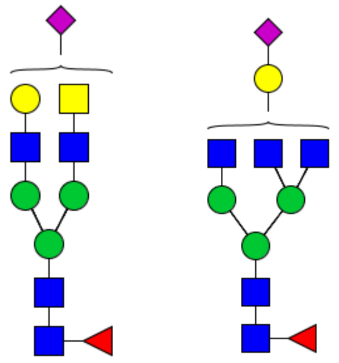

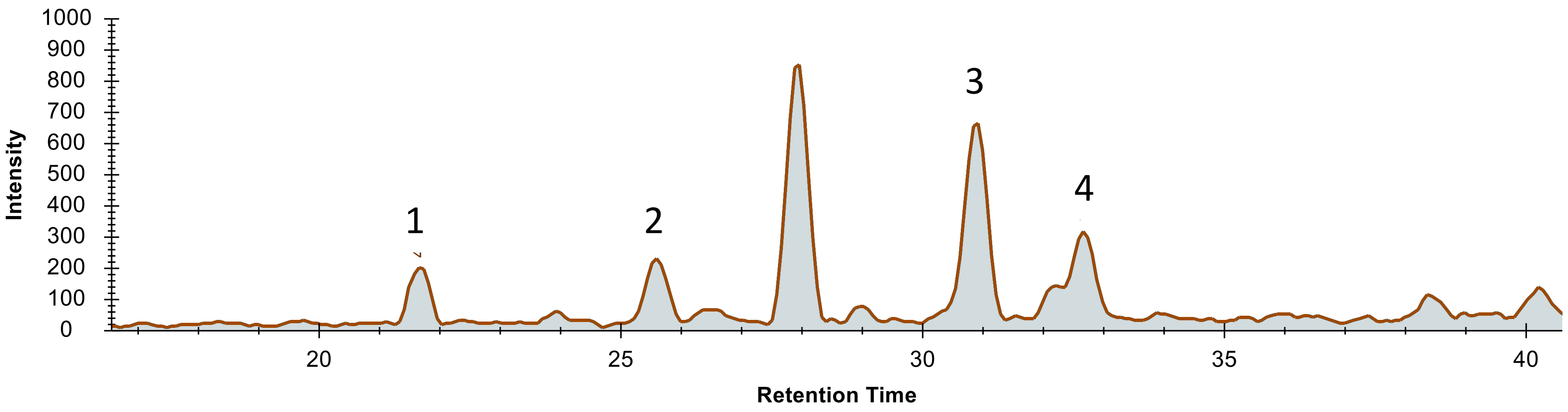

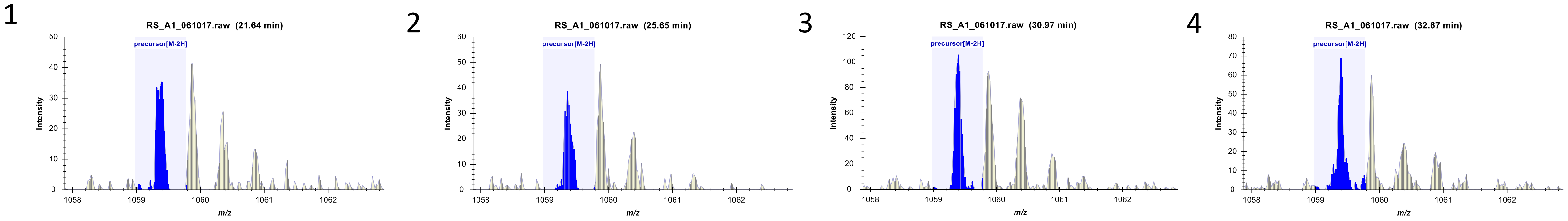

48a 48b 48c 48d

GlytouCan ID: G03380EA, G46208AF

CID-MS/MS
(m/z 1059.40 $^{2-}$)

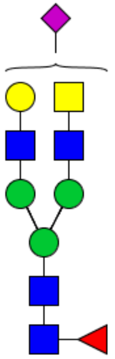

48a

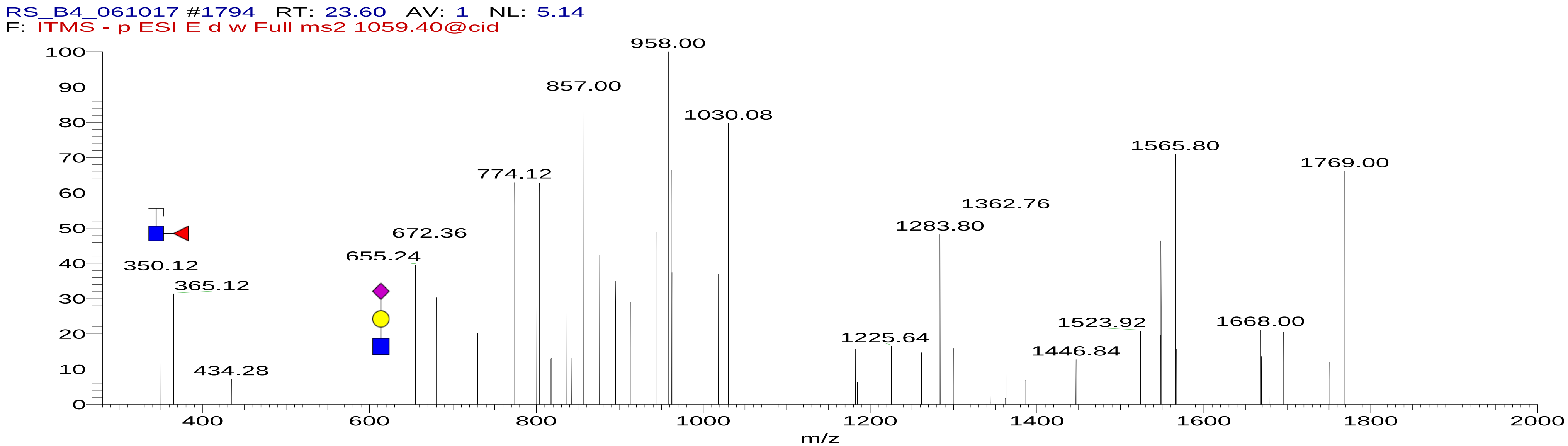

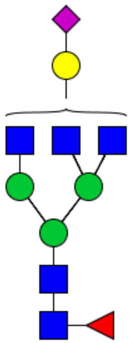

48b

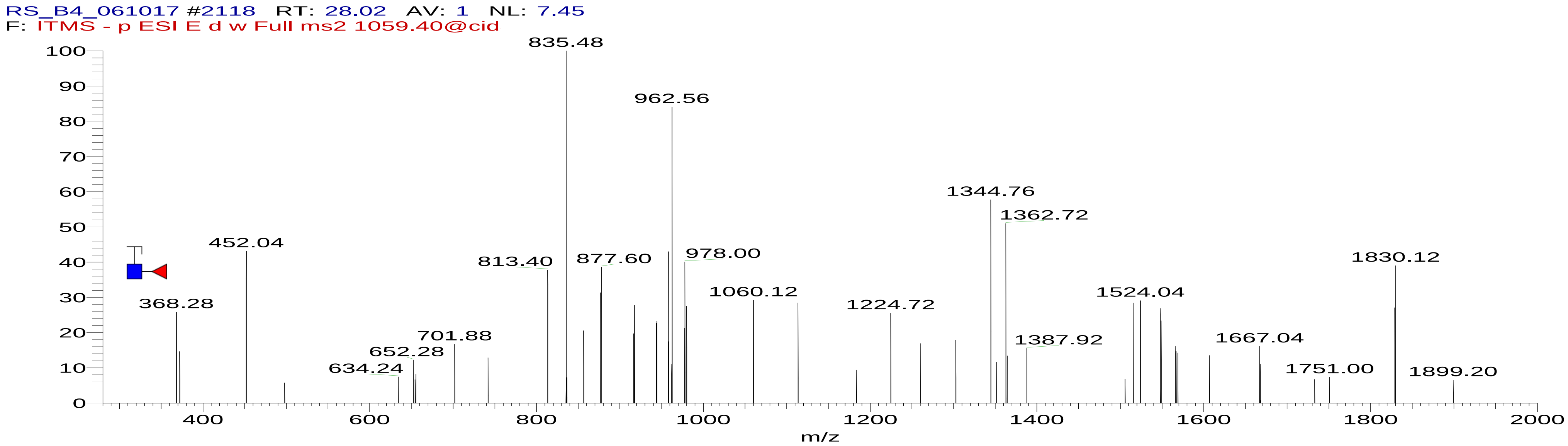

GlytouCan ID: G03380EA, G46208AF

CID-MS/MS
(*m/z* 1059.40 $^{2-}$)

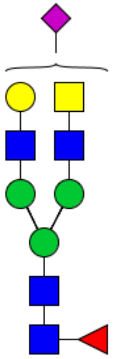

48c

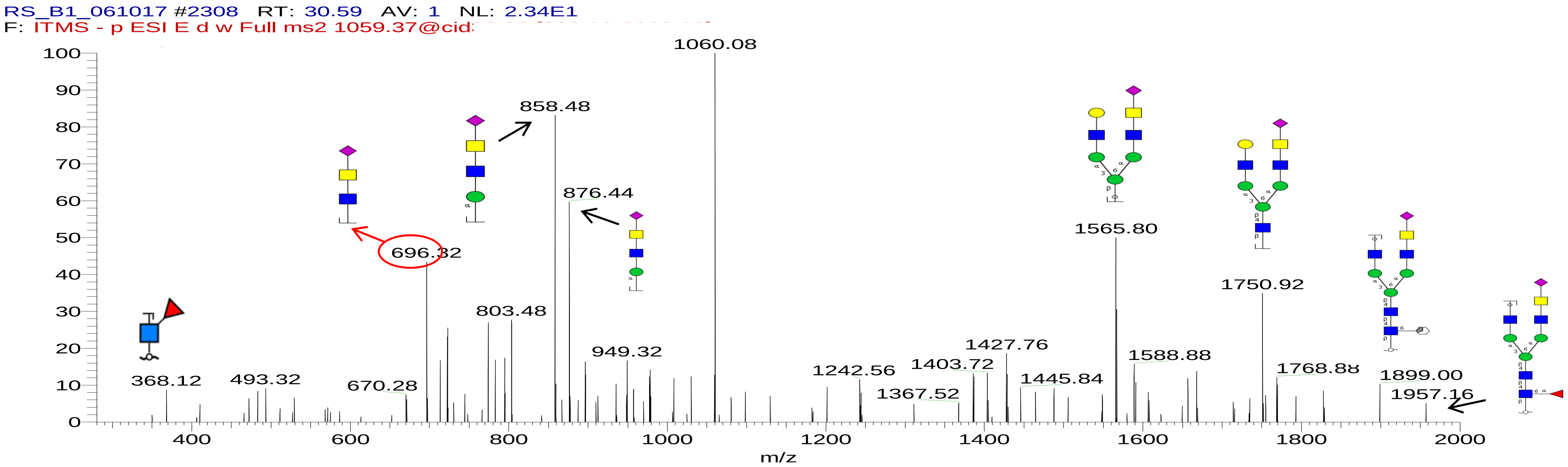

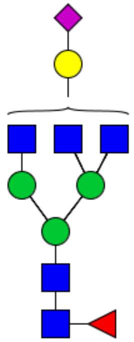

48d

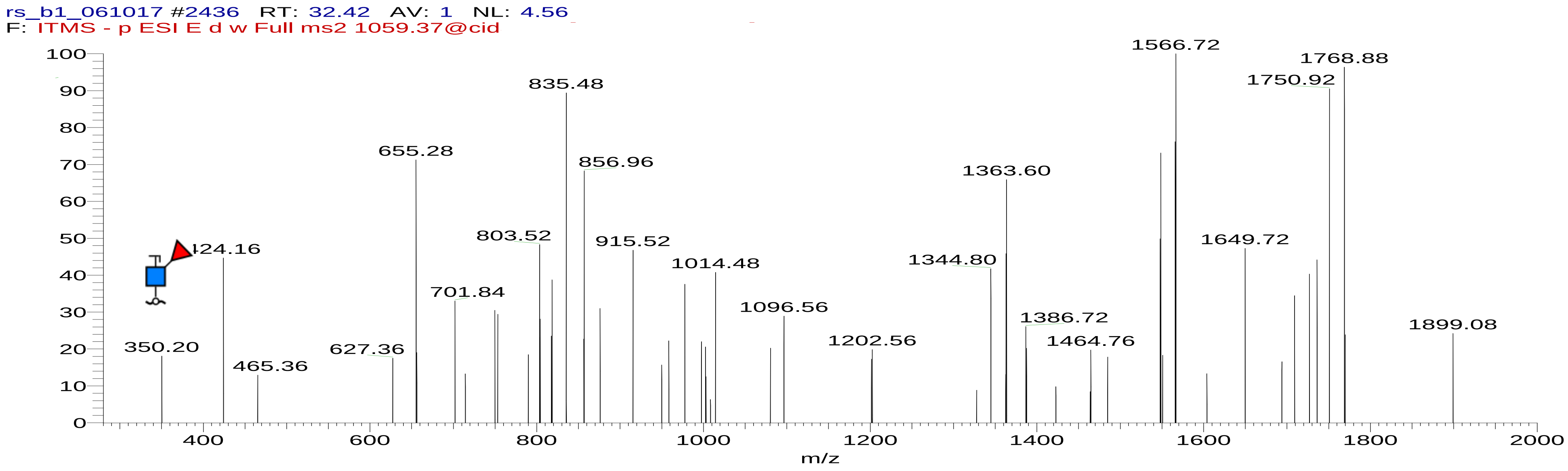

(Hex)2 (HexNAc)3 (NeuAc)1 + (Man)3(GlcNAc)2

GlytouCan ID: G53989JC

[M-2H] 1067.39

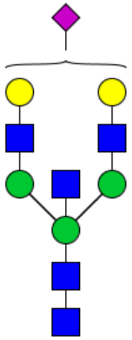

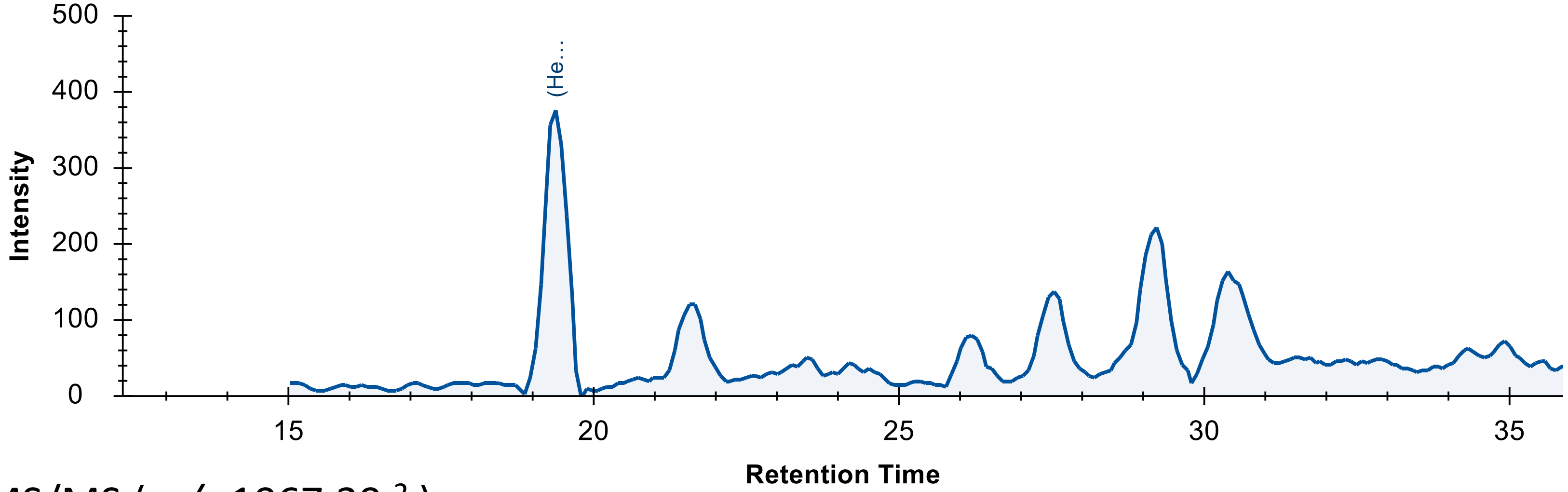

CID-MS/MS (m/z 1067.39 $^{2-}$)

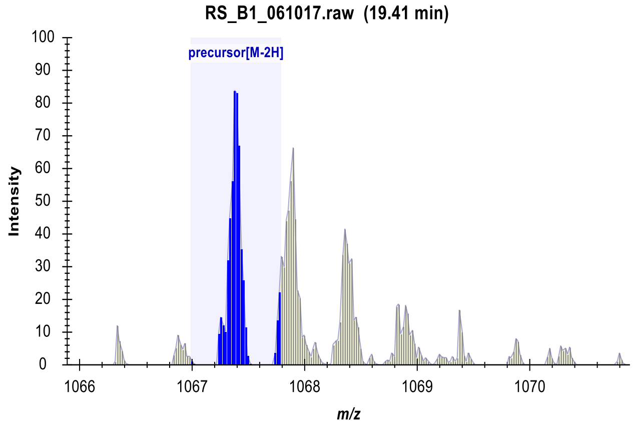

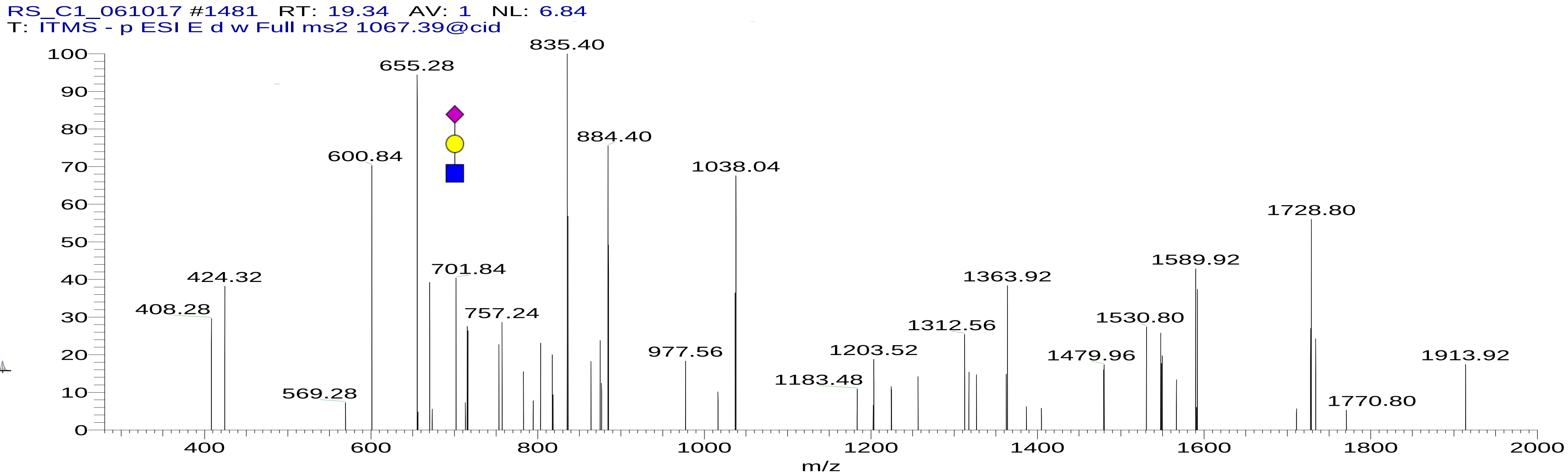

Glycan ID: 49

(Hex)3 (HexNAc)3 (Deoxyhexose)1 + (Man)3(GlcNAc)2

GlytouCan ID: G42397NB
[M-2H] 1075.88

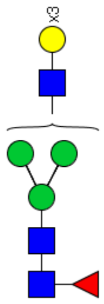

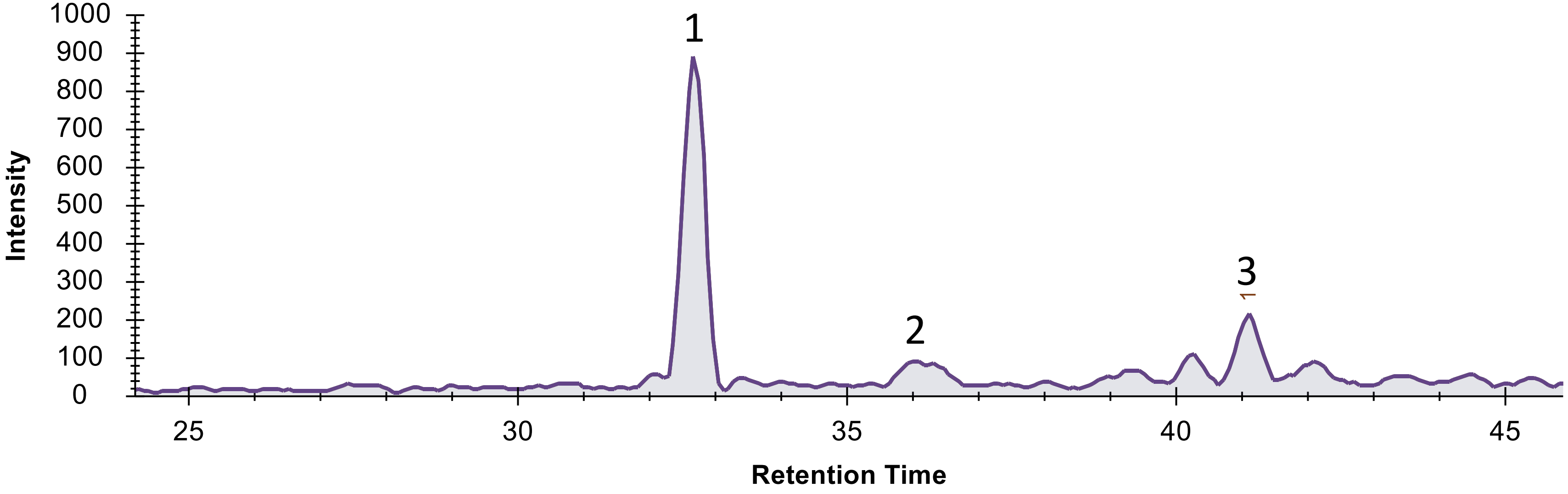

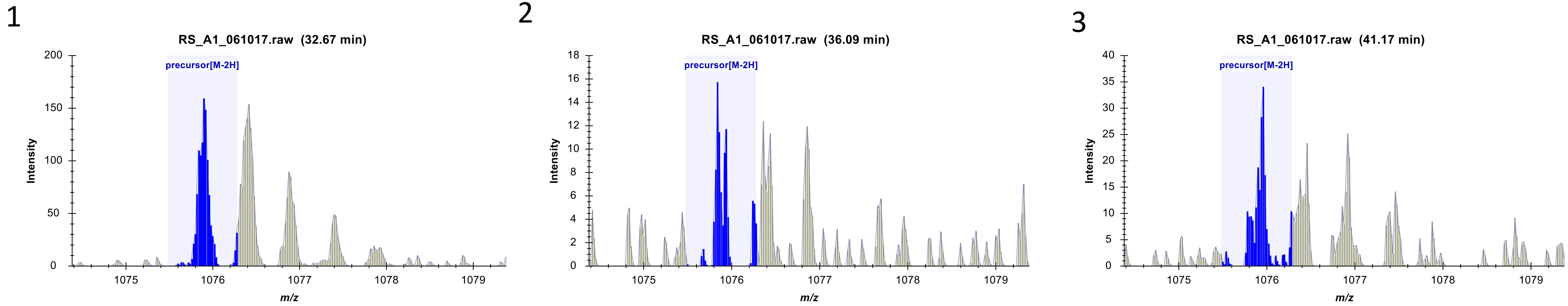

50a 50b 50c

GlytouCan ID: G42397NB

CID-MS/MS
(m/z 1075.88 $^{2-}$)

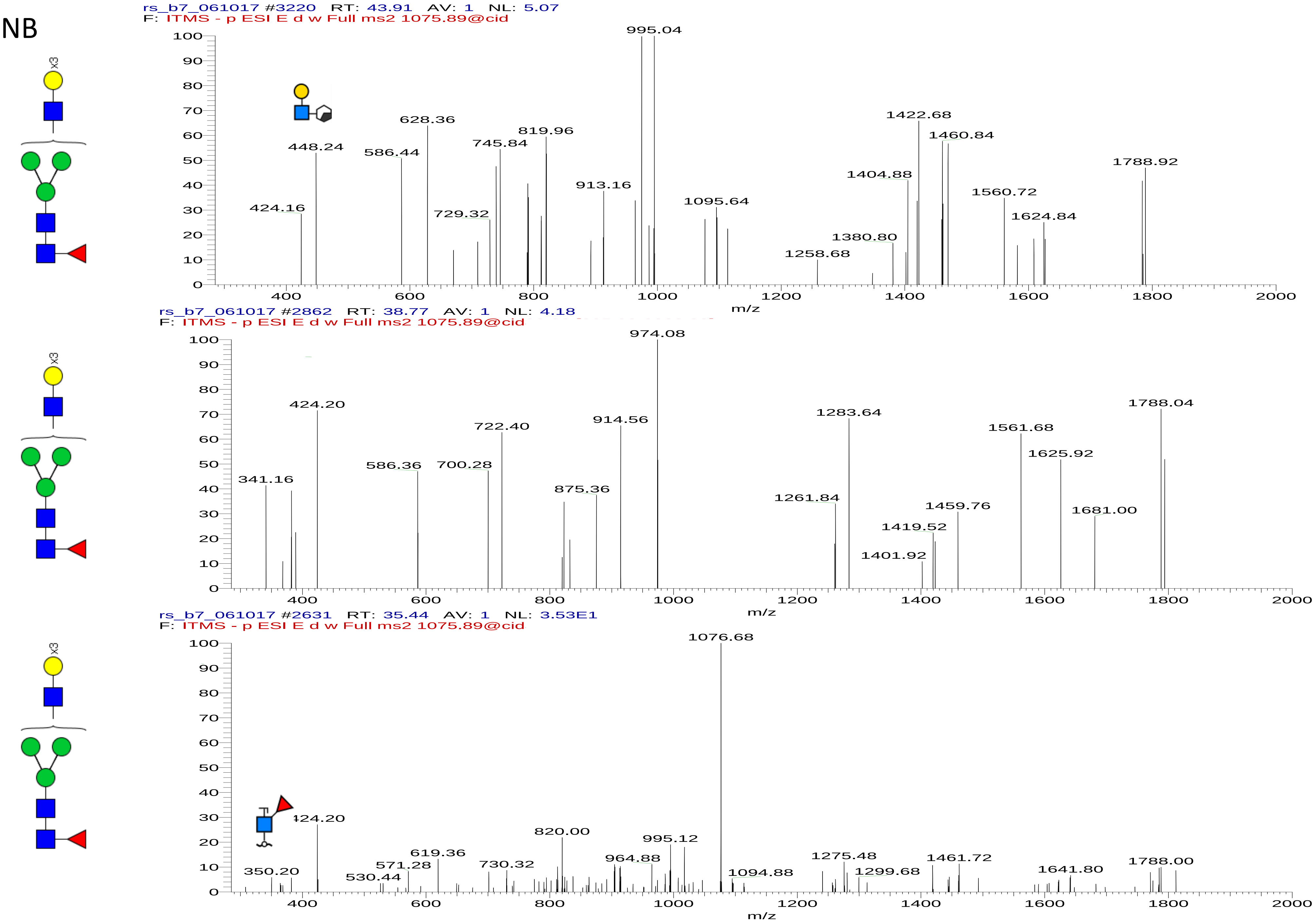

(HexNAc)4 (Deoxyhexose)1 (NeuAc)1 + (Man)3(GlcNAc)2

No GlytouCan ID

[M-2H] 1079.9

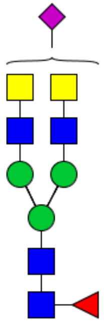

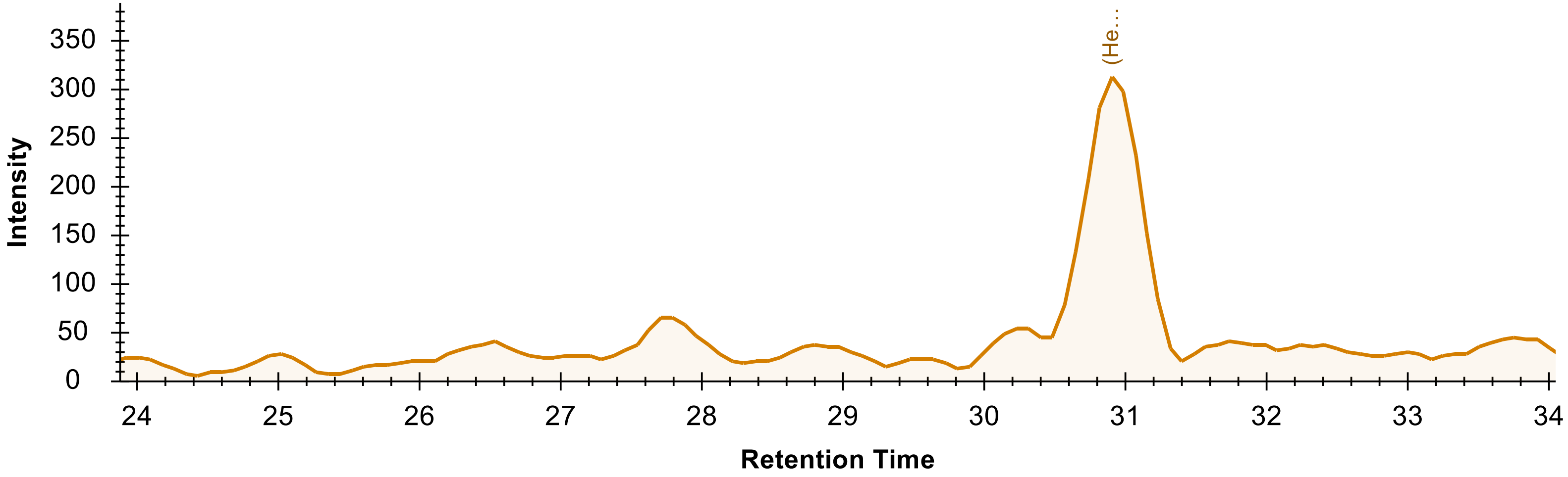

CID-MS/MS (*m/z* 1079.9 $^{2-}$)

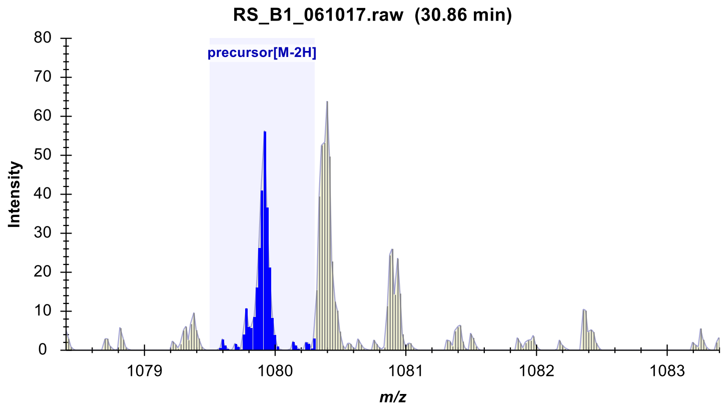

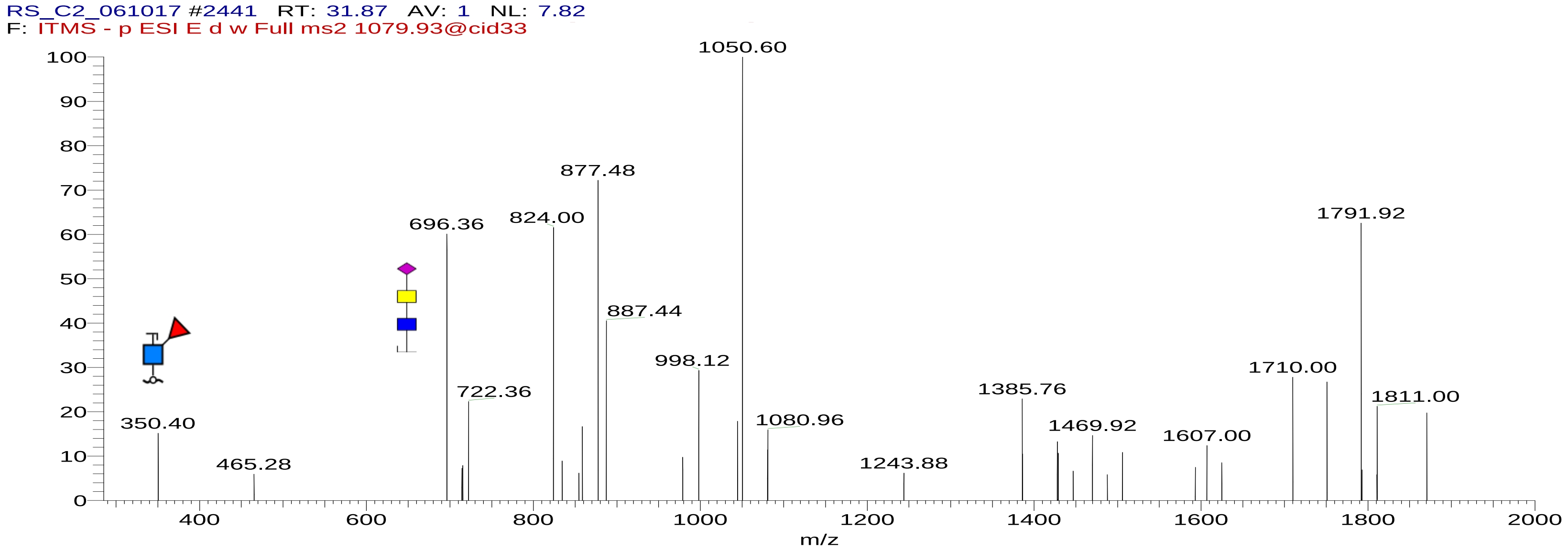

Glycan ID: 51

(Hex)2 (HexNAc)2 (NeuAc)2 + (Man)3(GlcNAc)2
GlytouCan ID: G09675DY
[M-2H] 1111.40

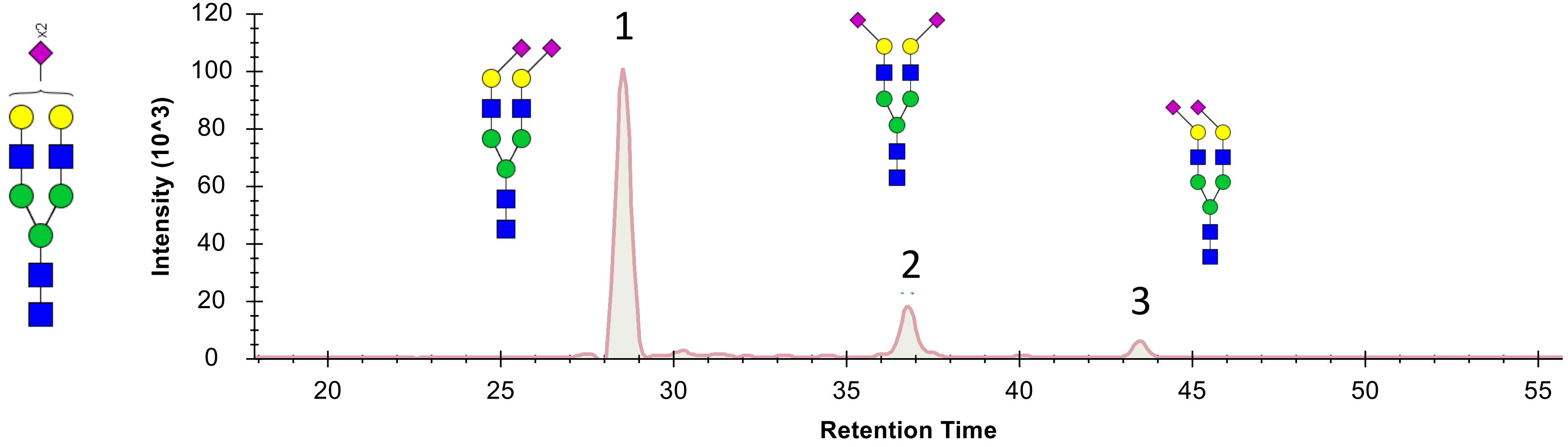

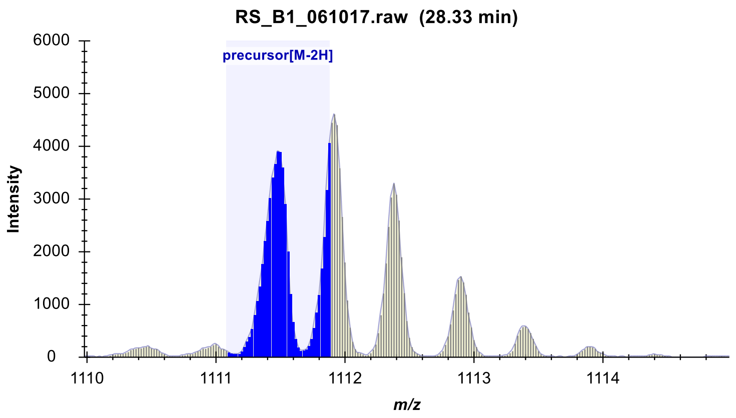

52a

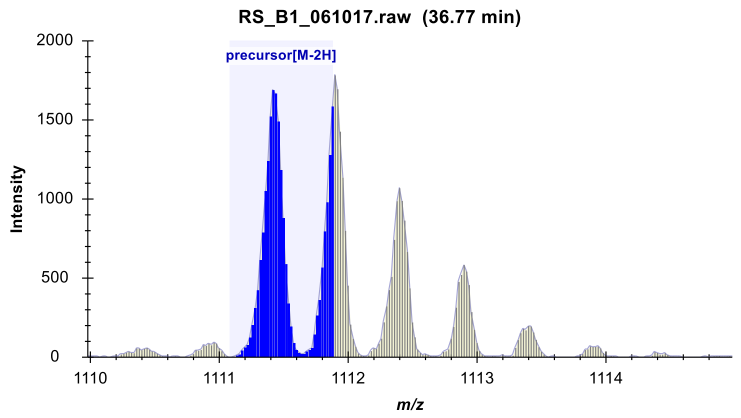

52b

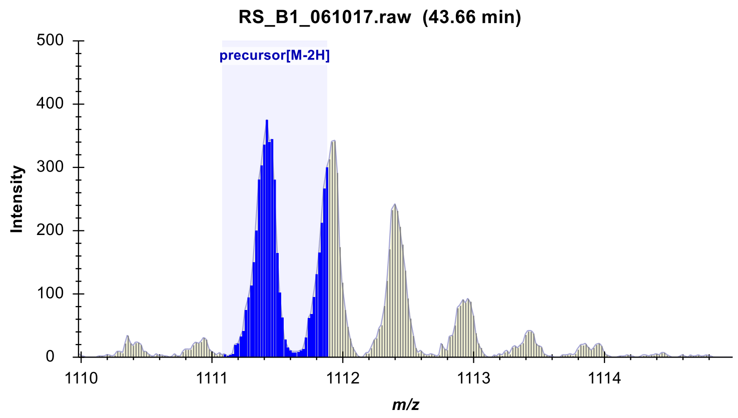

52c

GlytouCan ID: G09675DY

CID-MS/MS
(m/z 1111.40 $^{2-}$)

52a

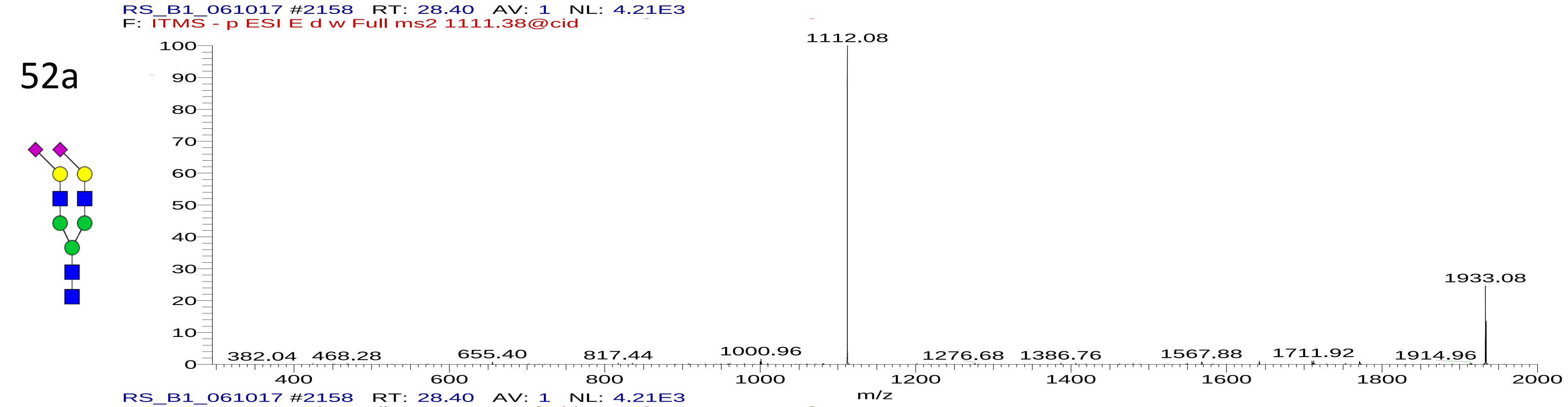

52b

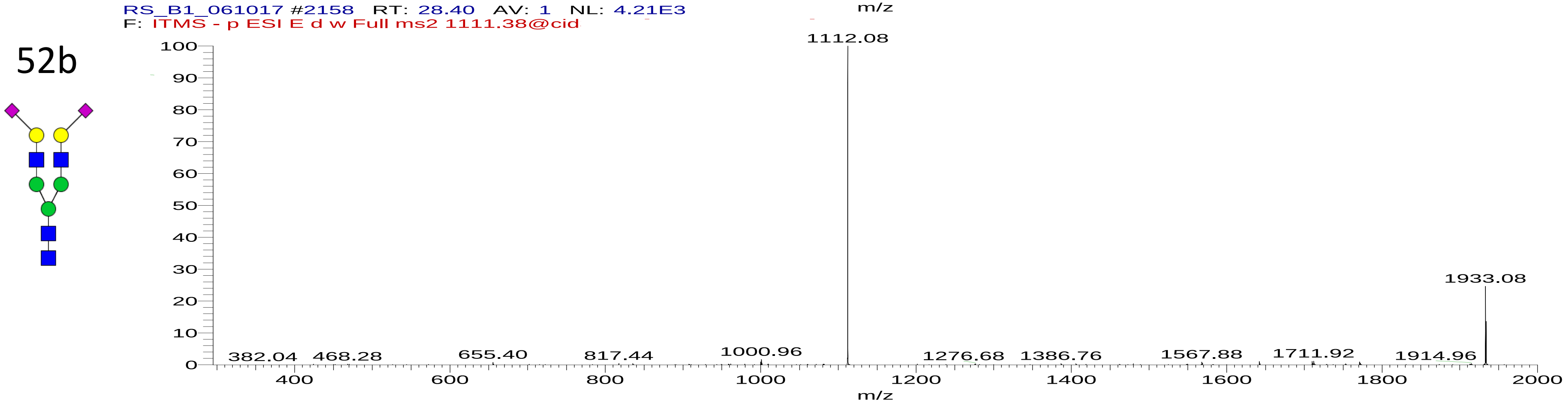

52c

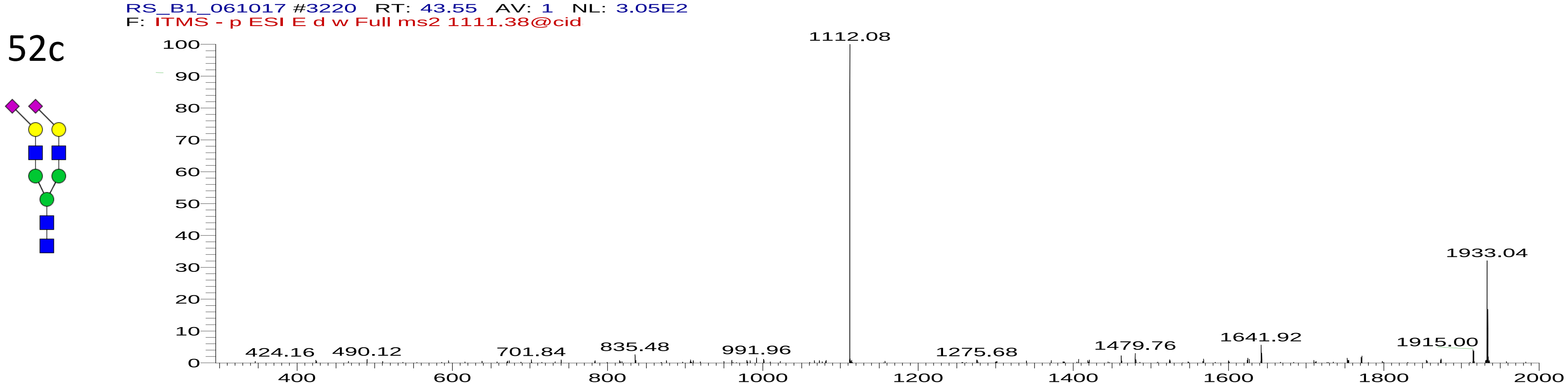

(Hex)2 (HexNAc)2 (NeuAc)1 (NeuGc)1 + (Man)3(GlcNAc)2

No GlytouCan ID

[M-2H] 1119.41

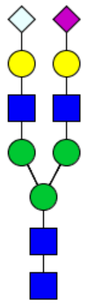

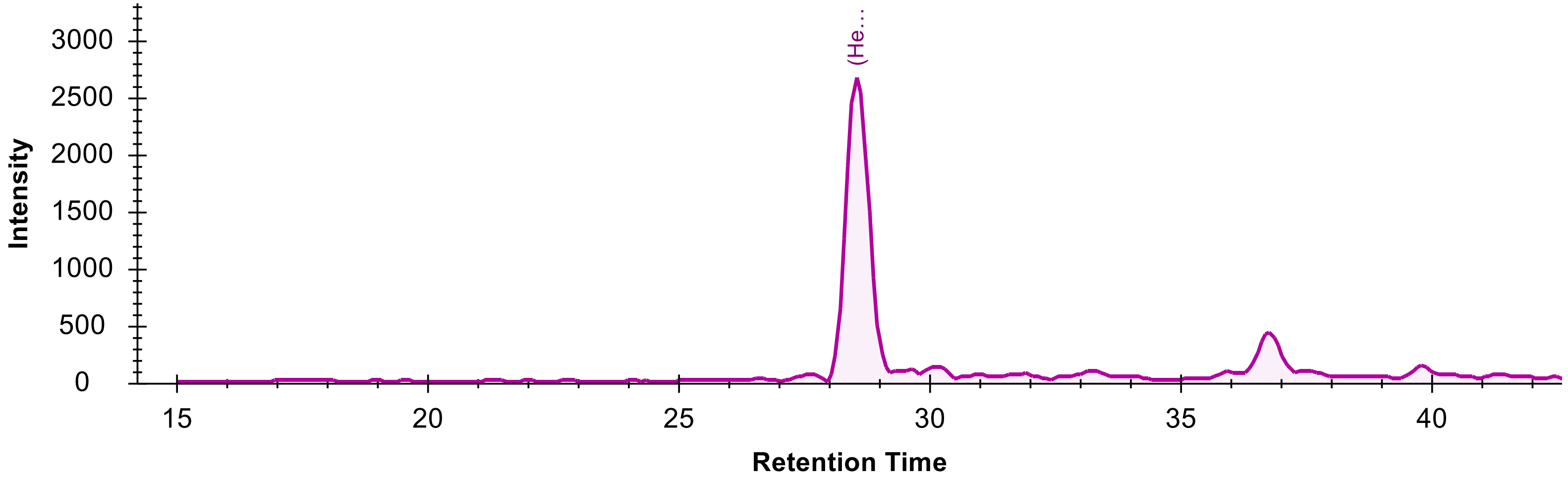

CID-MS/MS (m/z 1119.41 $^{2-}$)

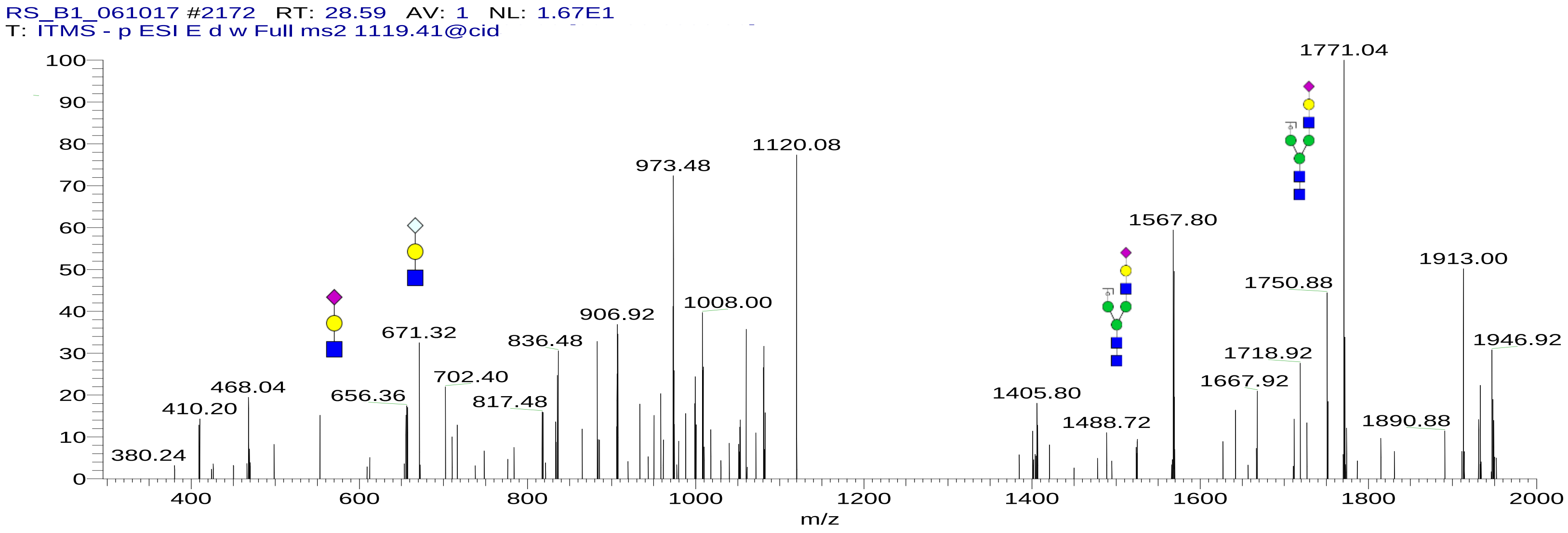

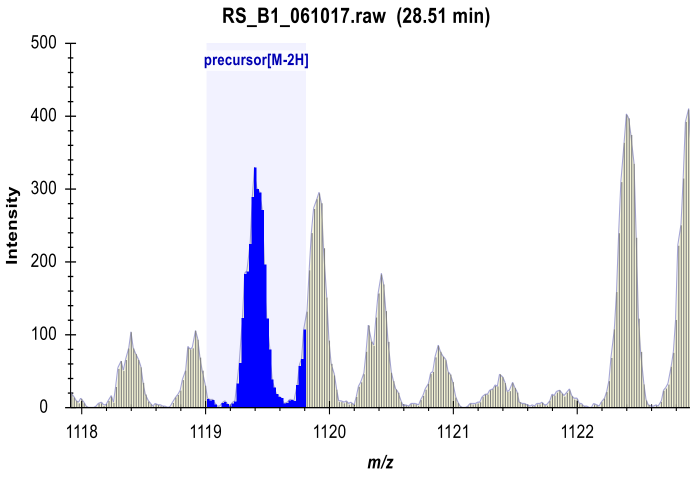

Gycan ID: 53

(Hex)2 (HexNAc)2 (NeuGc)2 + (Man)3(GlcNAc)2
No GlytouCan ID
[M-2H] 1127.39

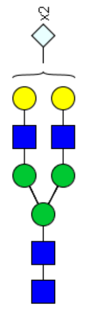

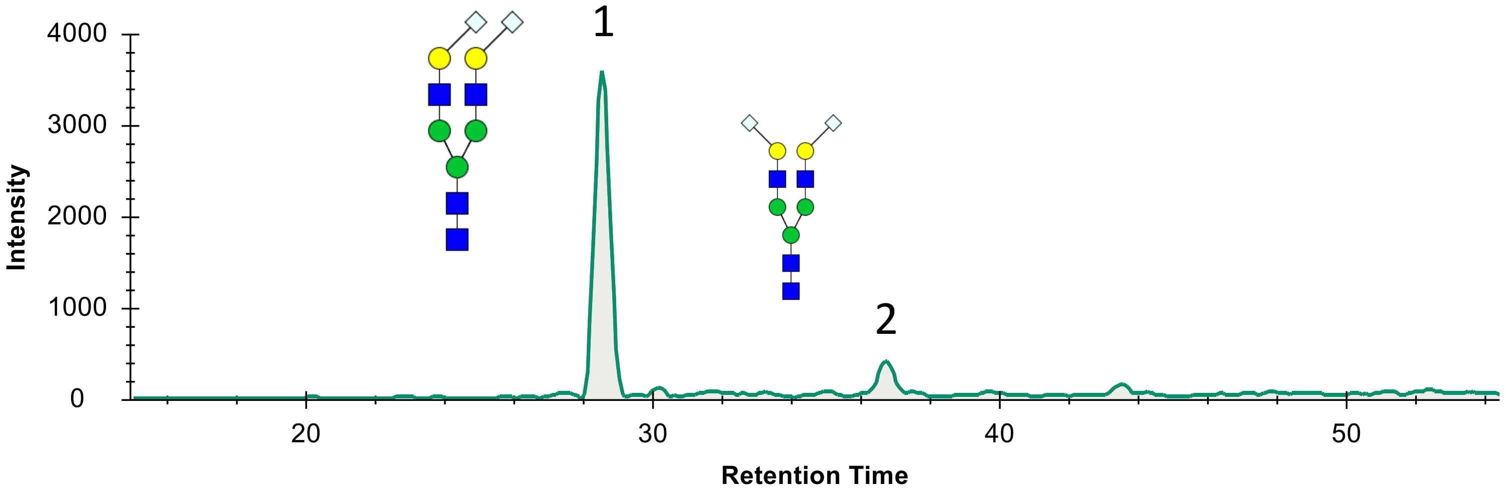

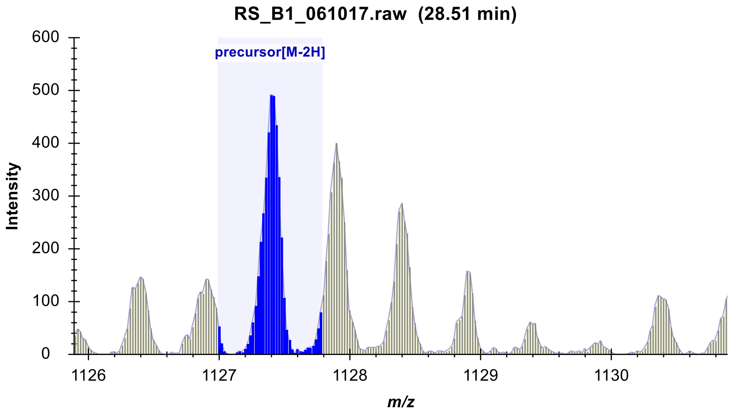

54a

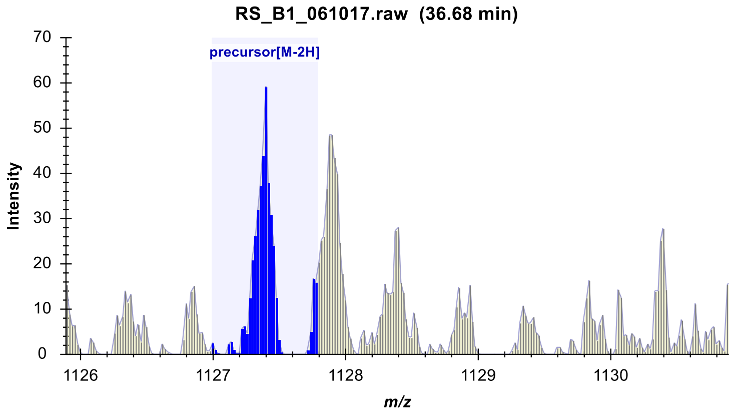

54b

CID-MS/MS
(m/z 1127.39 $^{2-}$)

54a

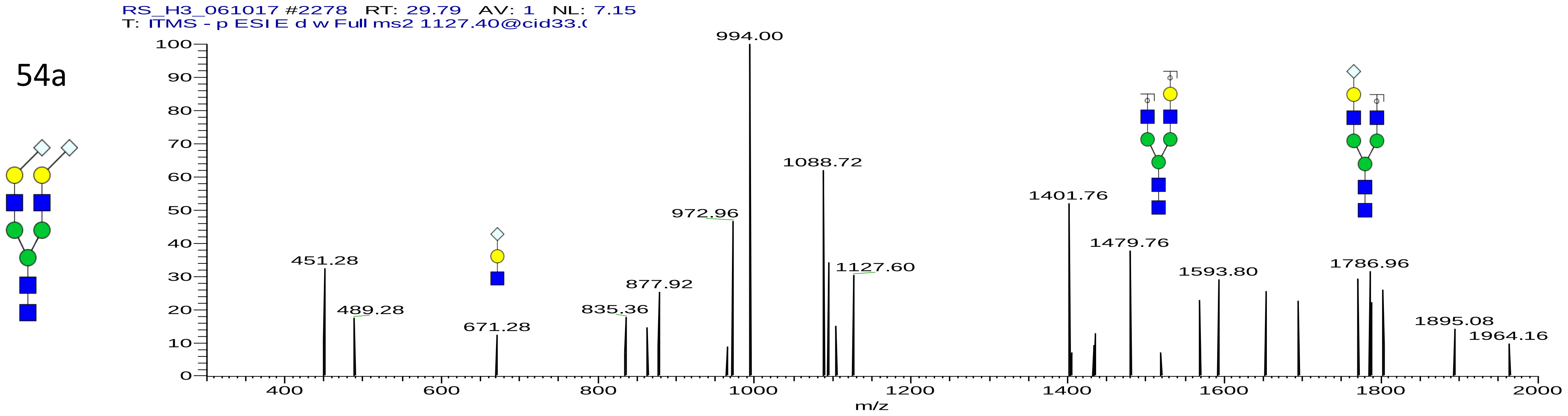

54b

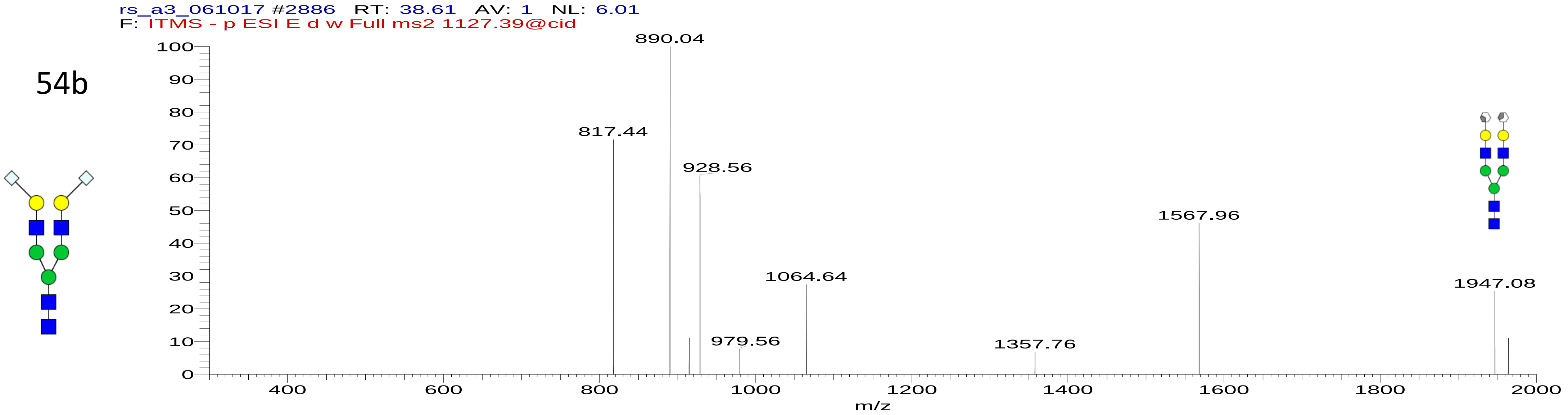

(Hex)1 (HexNAc)3 (NeuAc)2 + (Man)3(GlcNAc)2
No glytoucan
[M-2H] 1131.92

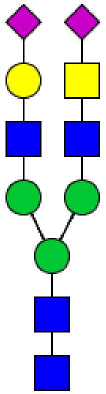

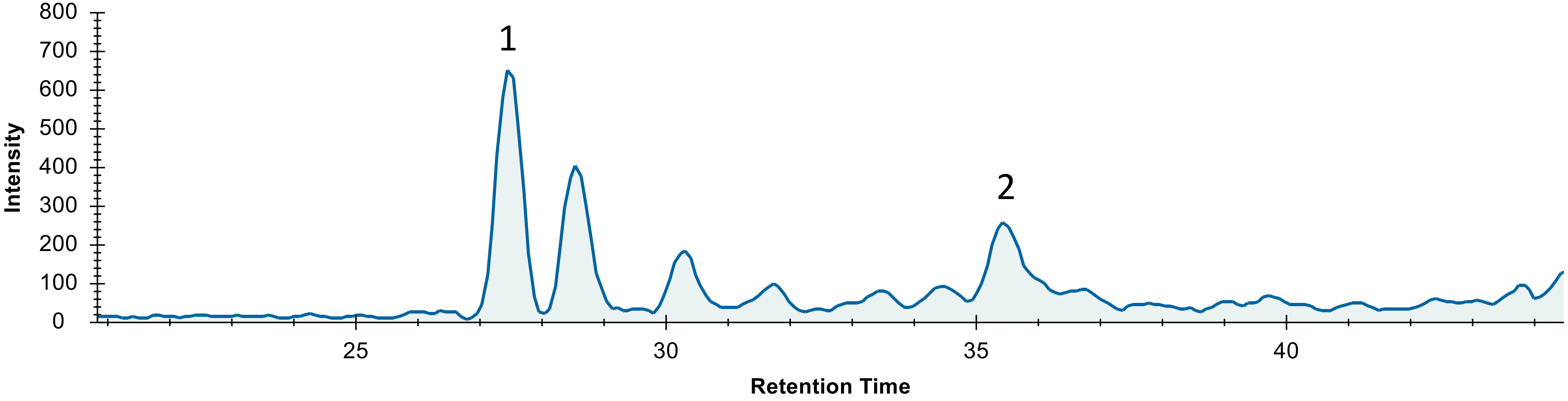

1

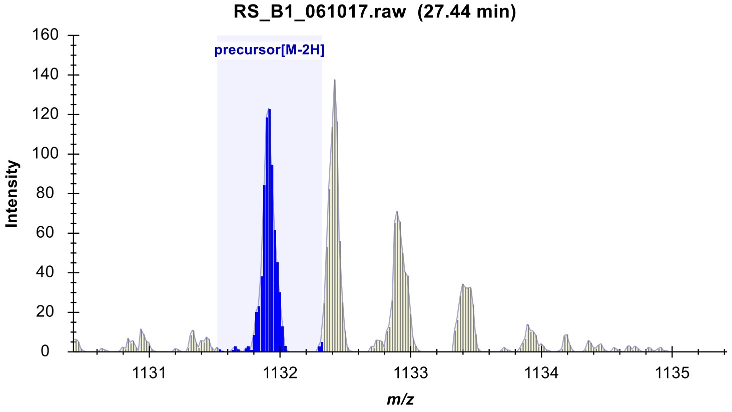

55a

2

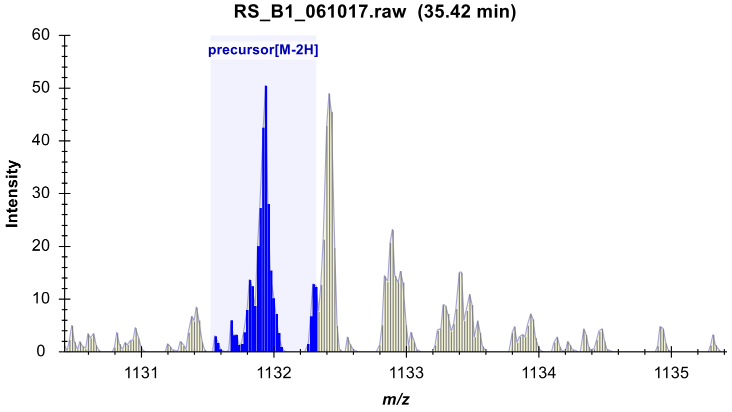

55b

(Hex)1 (HexNAc)3 (NeuAc)2 + (Man)3(GlcNAc)2

CID-MS/MS
(*m/z* 1131.94 $^{2-}$)

55a

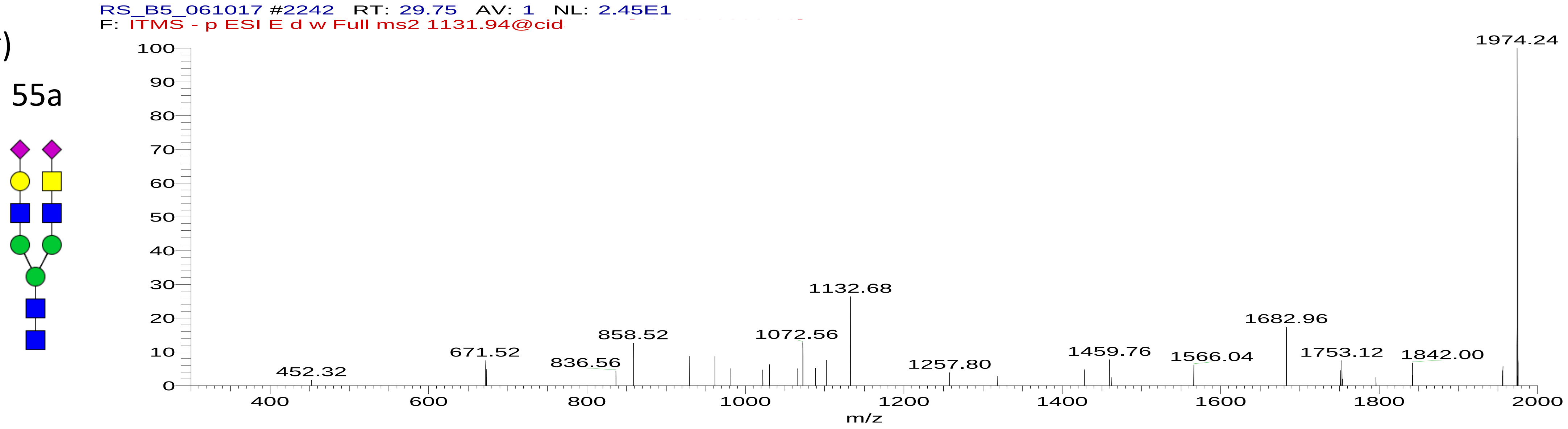

55b

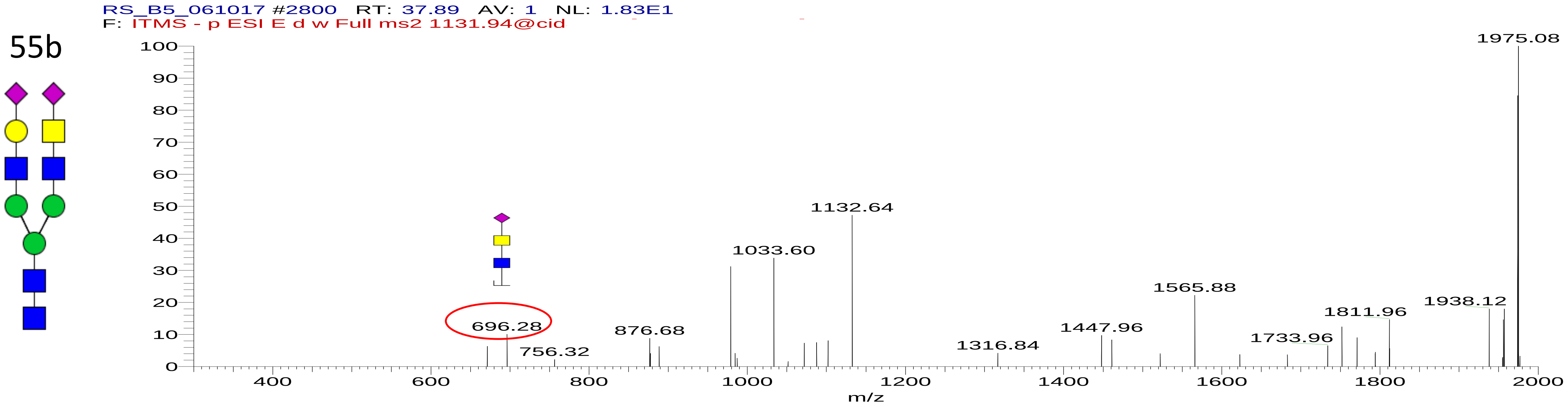

(Hex)2 (HexNAc)3 (Deoxyhexose)1 (NeuAc)1 + (Man)3(GlcNAc)2
No GlytouCan ID
[M-2H] 1140.42

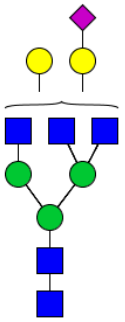

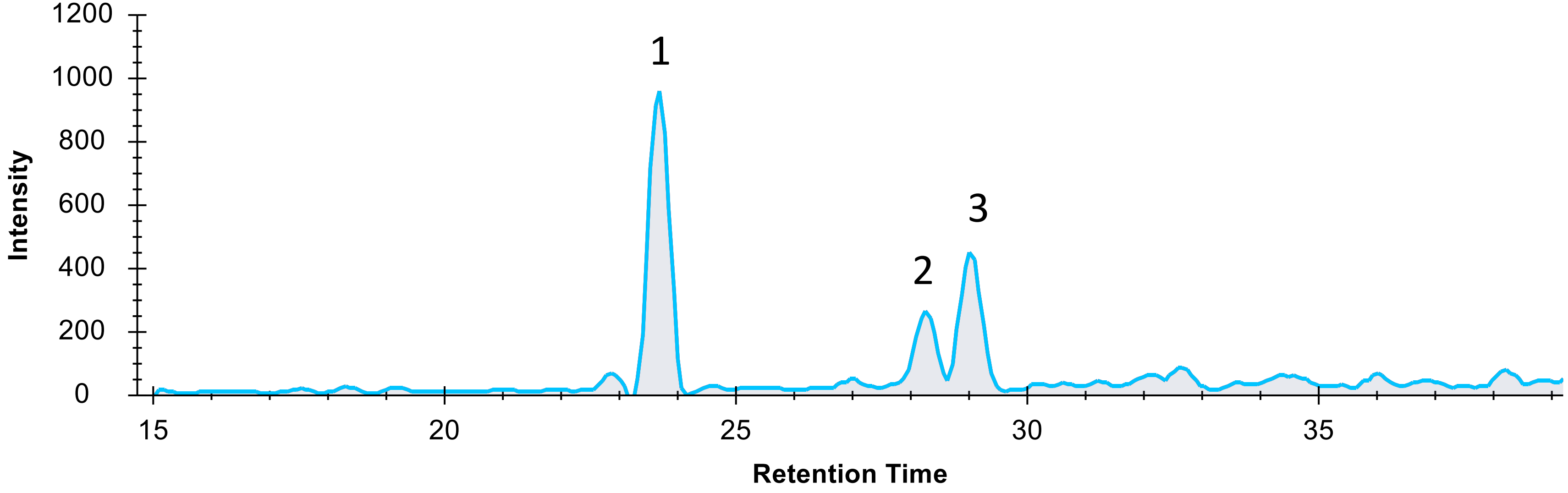

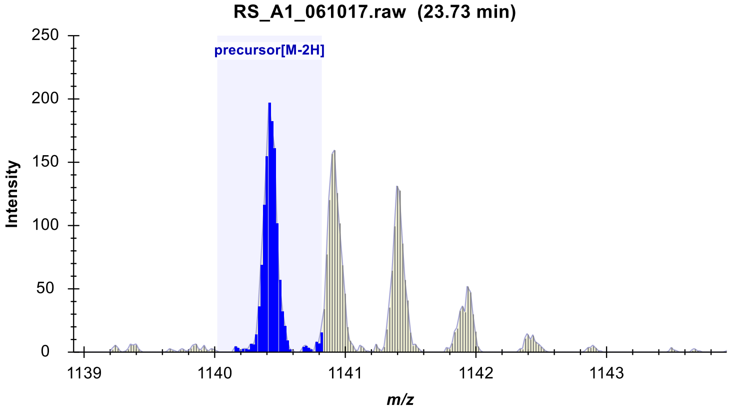

56a

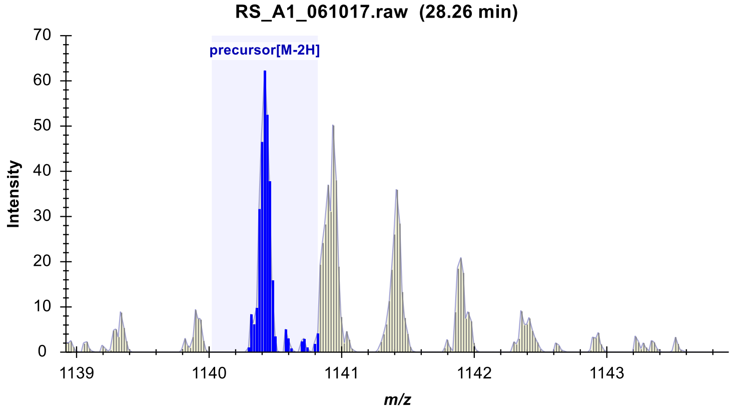

56b

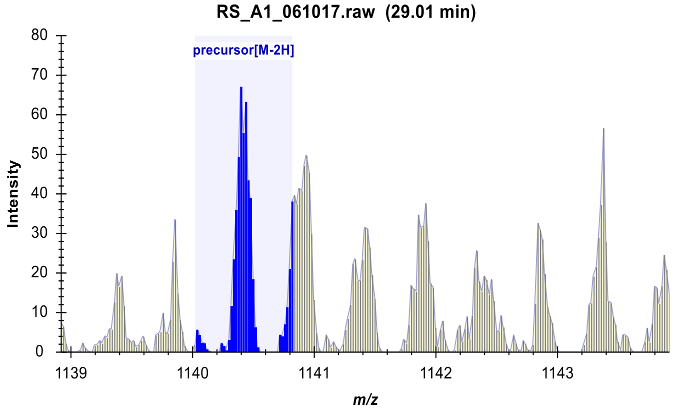

56c

CID-MS/MS

(m/z 1140.42 $^{2-}$)

56a

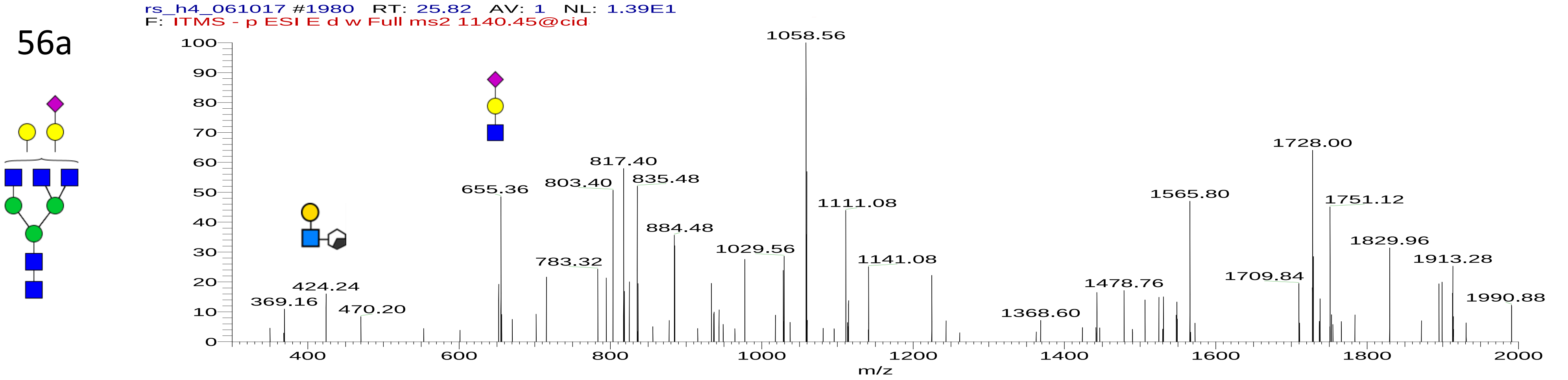

56b

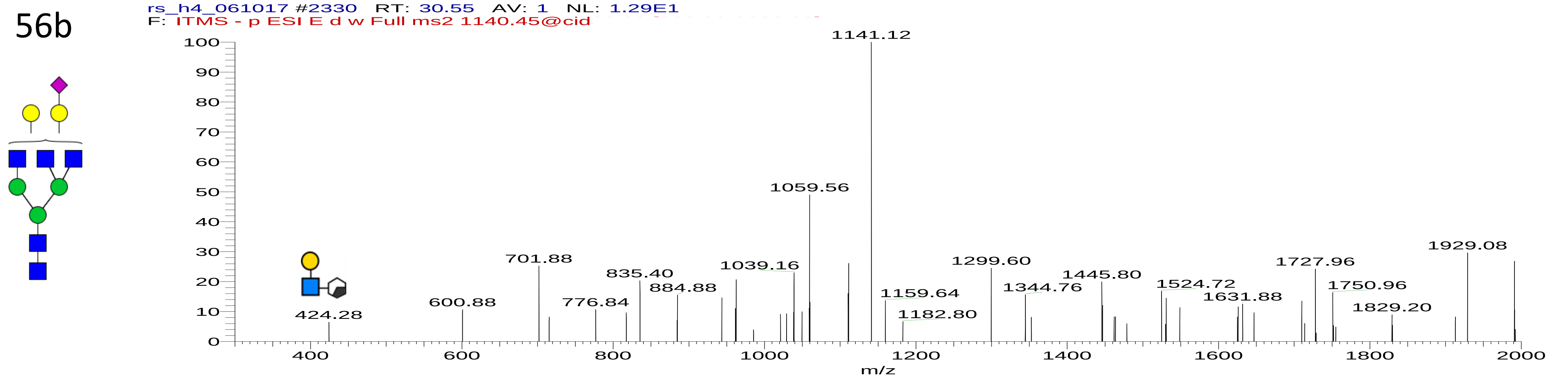

56c

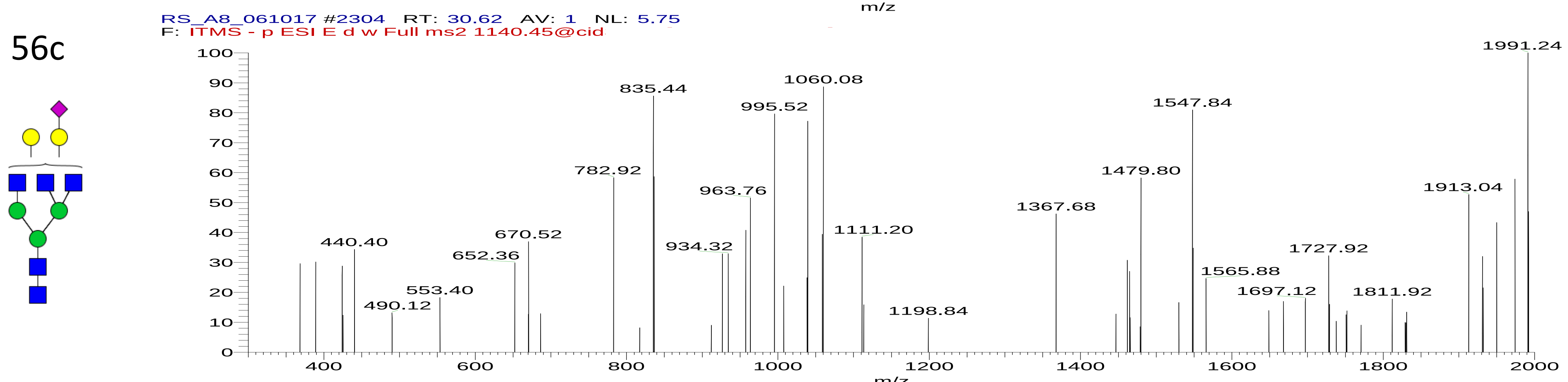

(Hex)3 (HexNAc)3 (NeuAc)1 + (Man)3(GlcNAc)2
GlytouCan ID: G02945OA

[M-2H] 1148.45

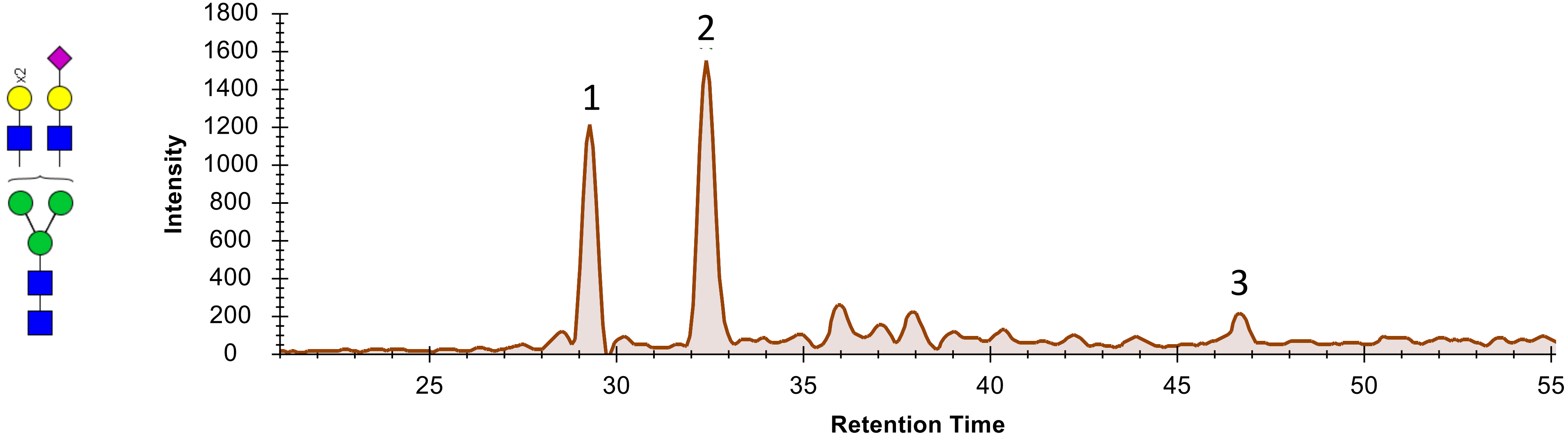

1

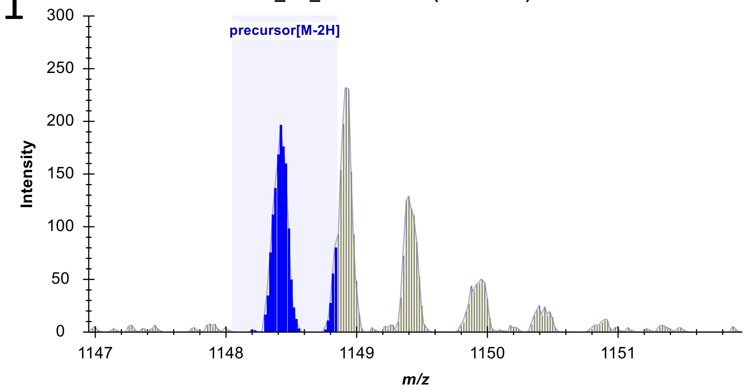

57a

2

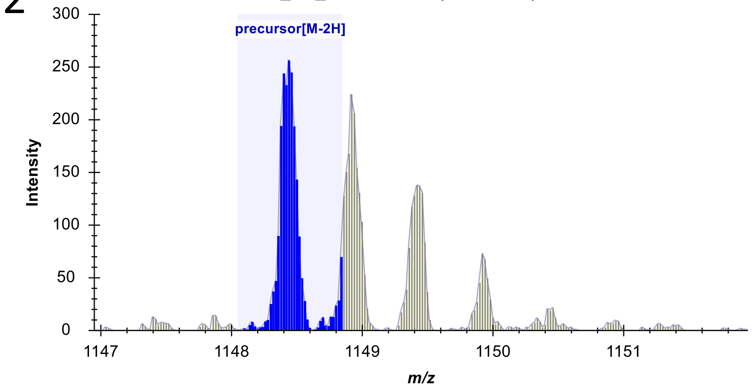

57b

3

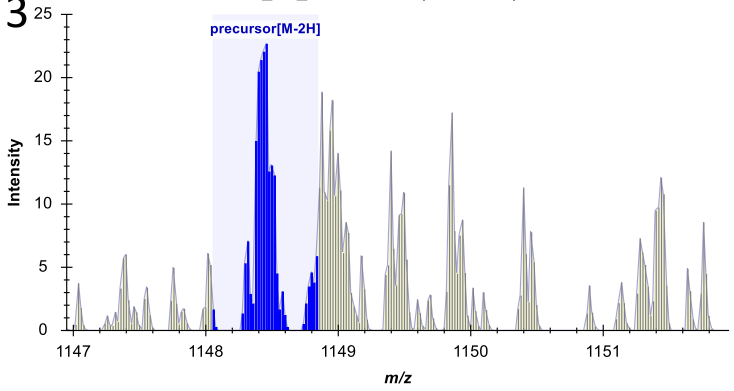

57c

GlytouCan ID: G02945OA

CID-MS/MS
(*m/z* 1148.43 $^{2-}$)

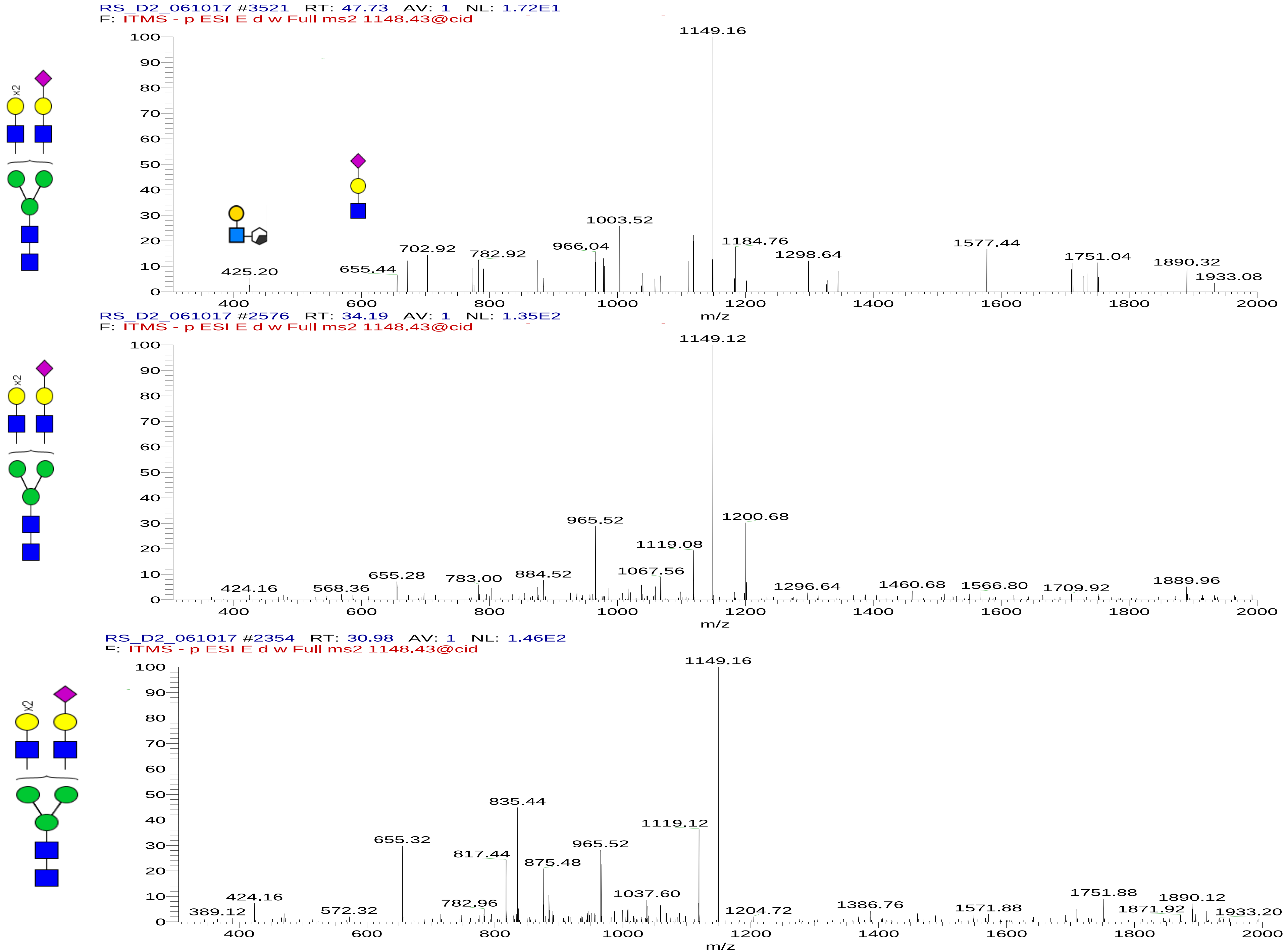

(Hex)2 (HexNAc)2 (Deoxyhexose)1 (NeuAc)2 + (Man)3(GlcNAc)
[M-2H] 1184.42
GlytouCan ID: G22754FQ

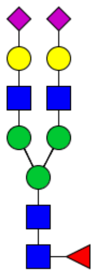

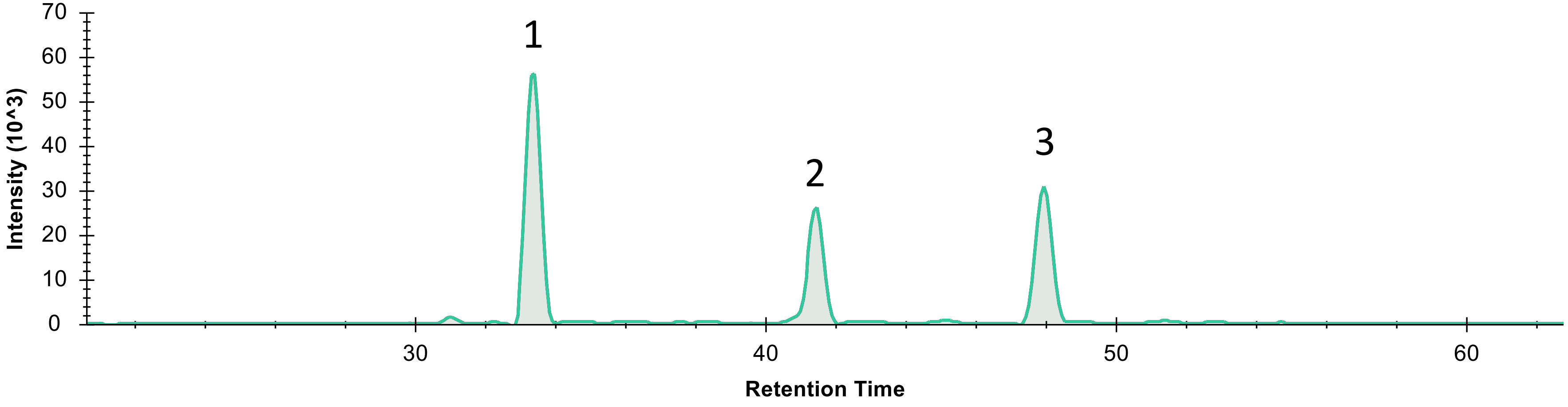

1
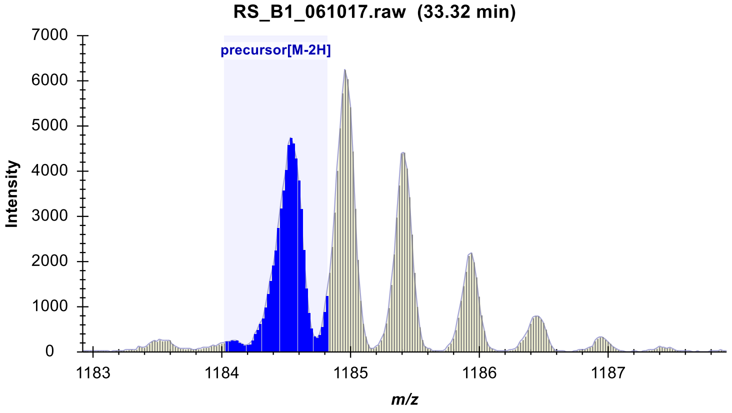

58a

2
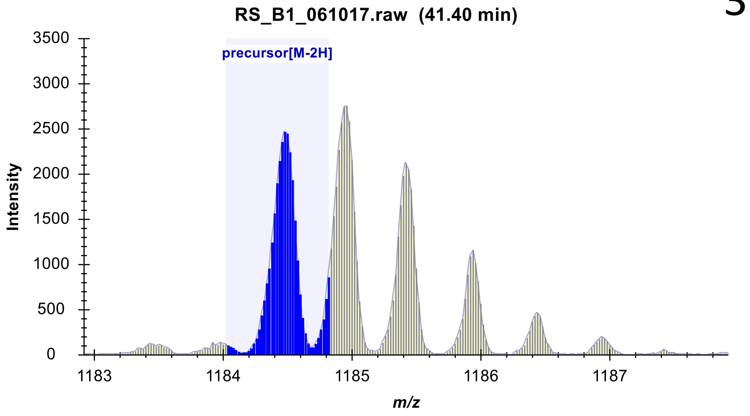

58b

3
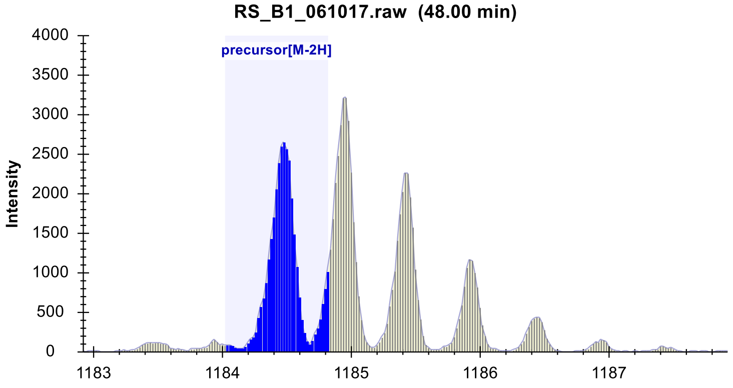

58c

GlytouCan ID: G22754FQ

CID-MS/MS

(m/z 1184.49 $^{2-}$)

58a

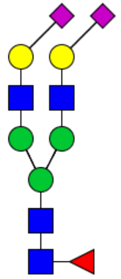

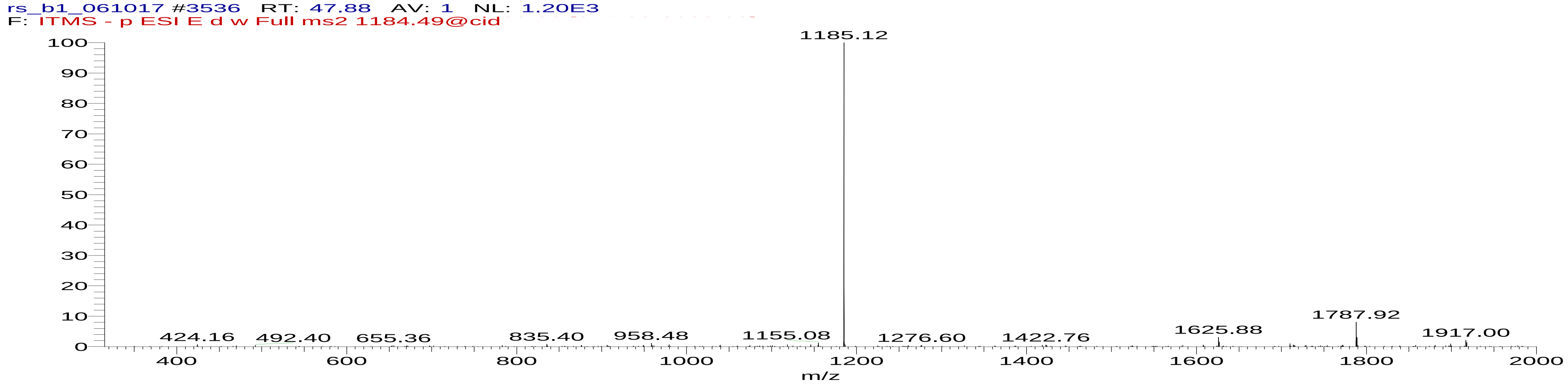

58b

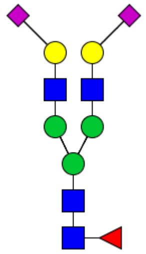

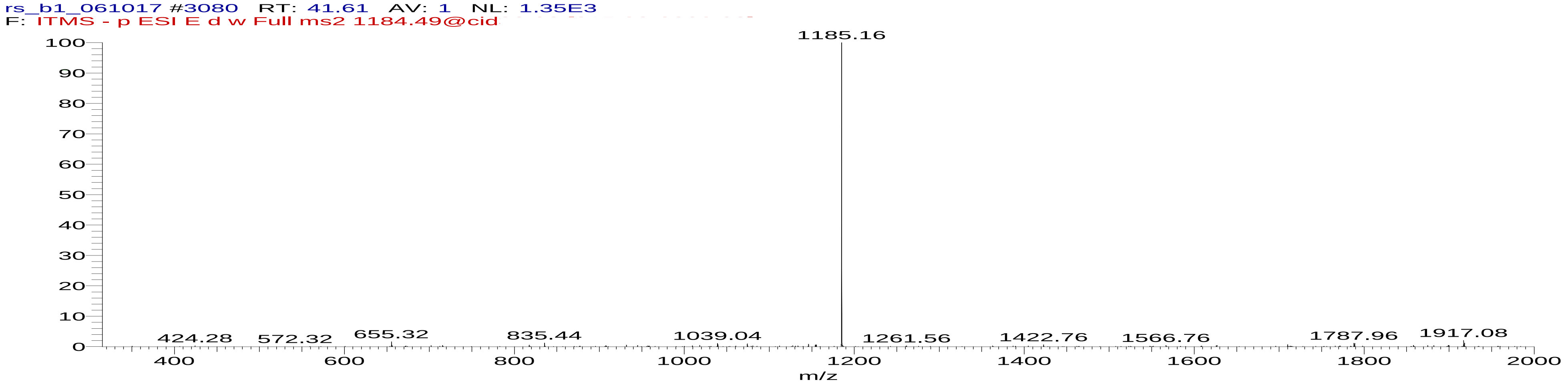

58c

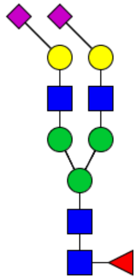

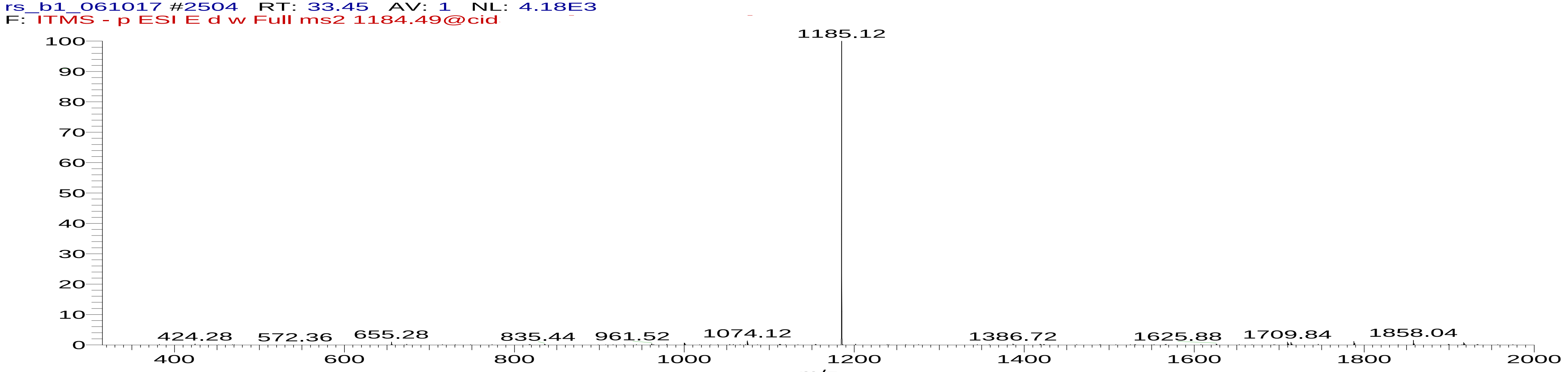

(Hex)2 (HexNAc)2 (Deoxyhexose)1 (NeuAc)1 (NeuGc)1 + (Man)3(GlcNAc)2
No Glytoucan ID
[M-2H] 1192.42

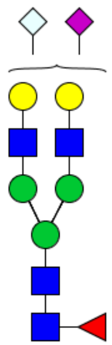

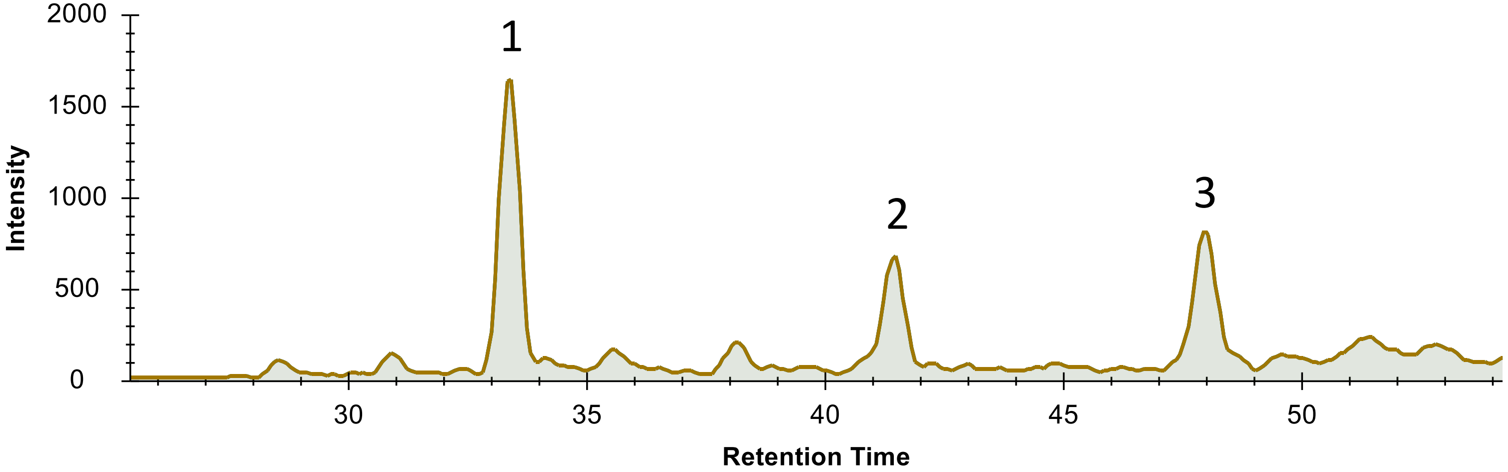

1
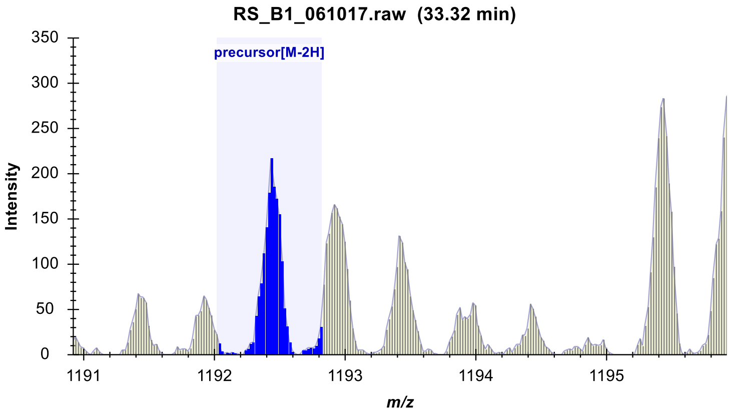

59a

2
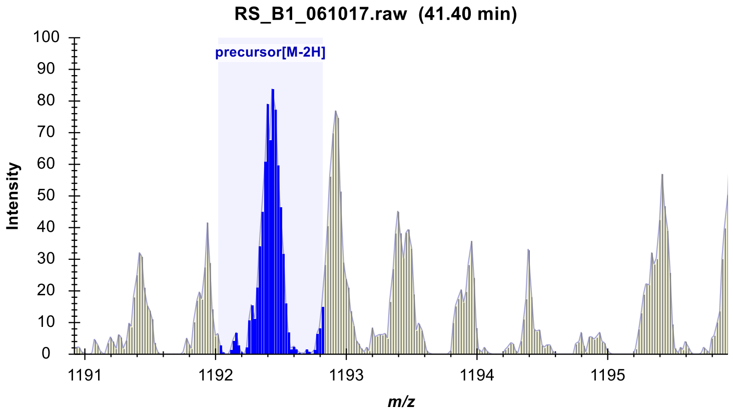

59b

3
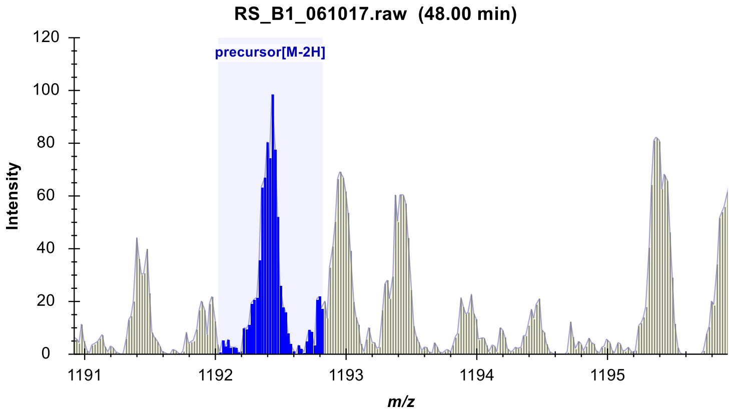

59c

CID-MS/MS

(m/z 1192.47 $^{2-}$)

59a

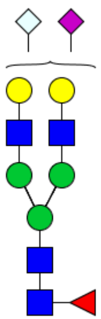

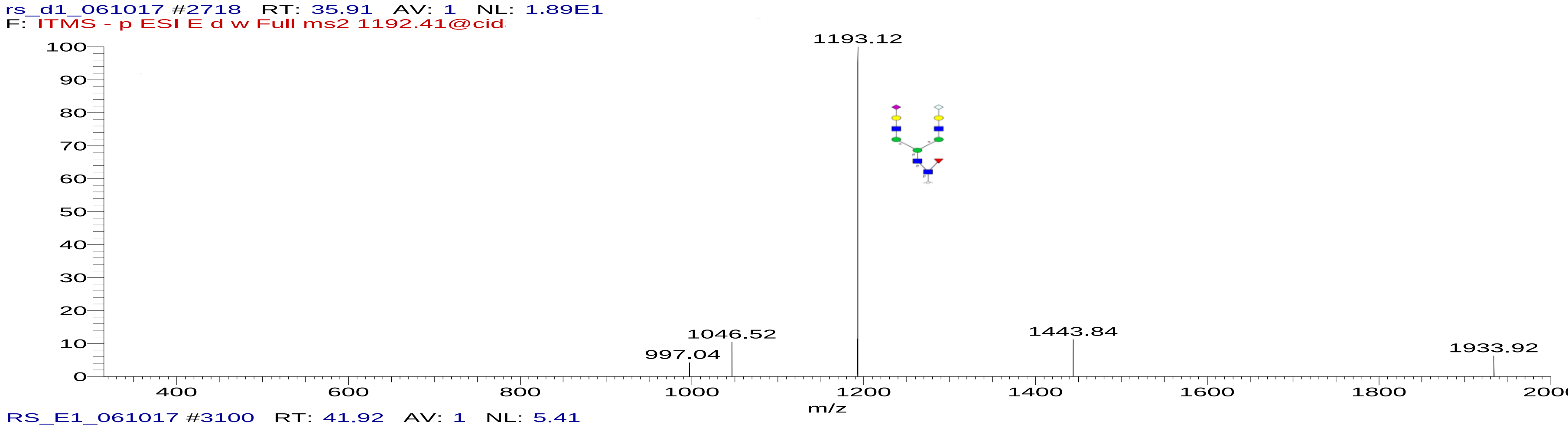

59b

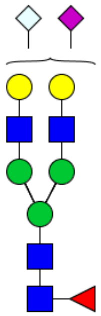

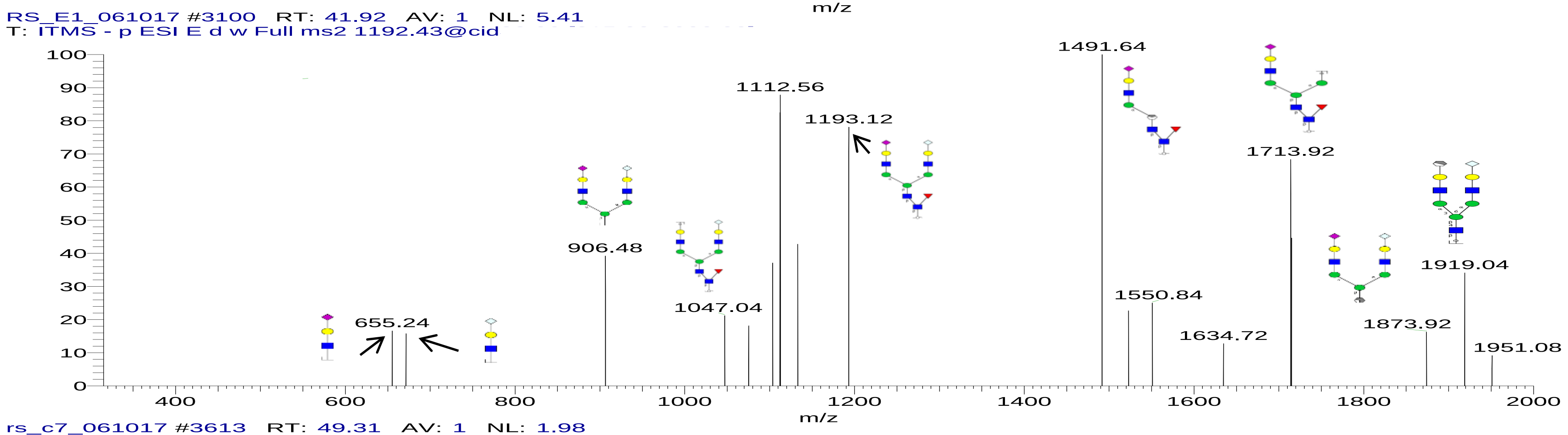

59c

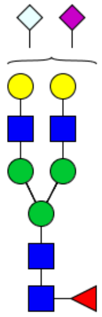

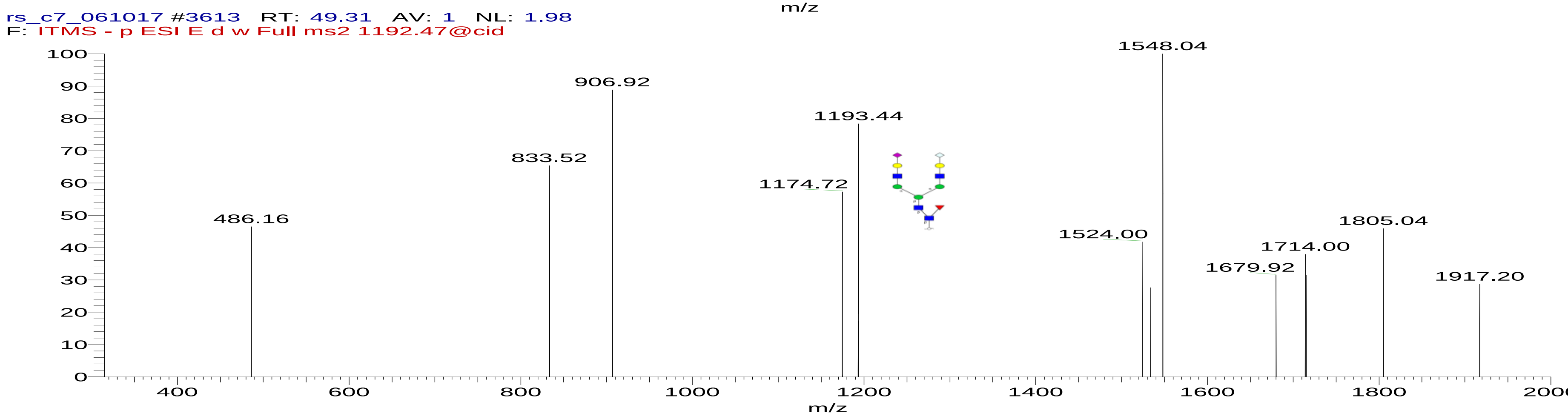

(Hex)2 (HexNAc)2 (Deoxyhexose)1 (NeuGc)2 + (Man)3(GlcNAc)2
No GlytouCan ID
[M-2H] 1200.43

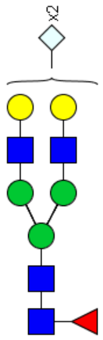

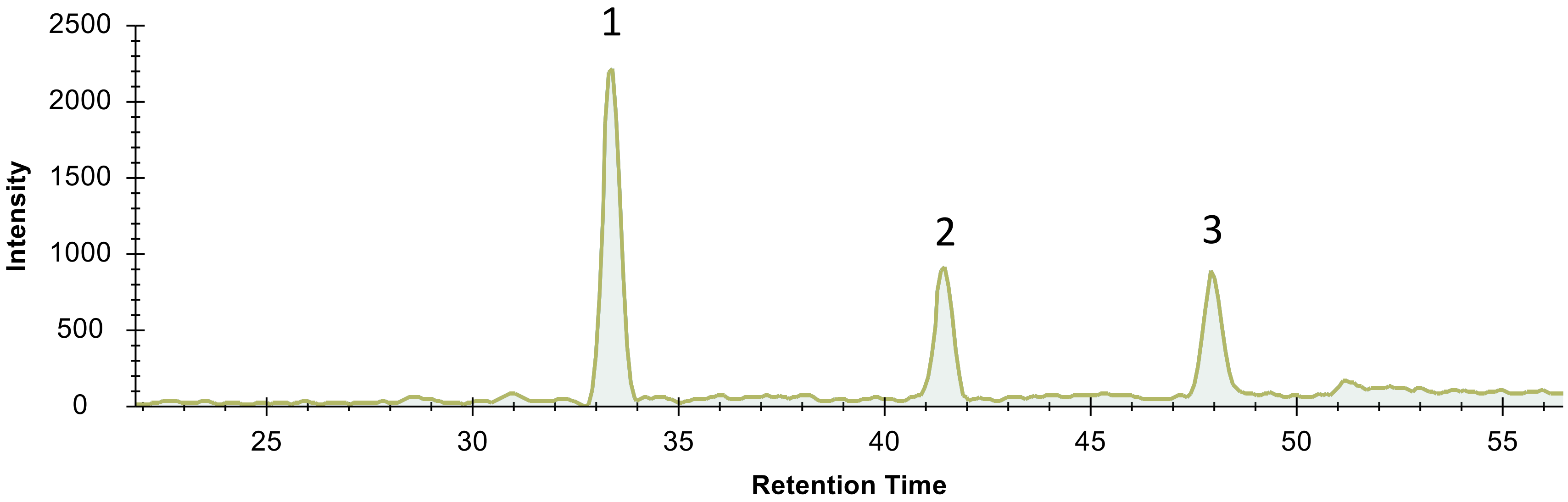

1

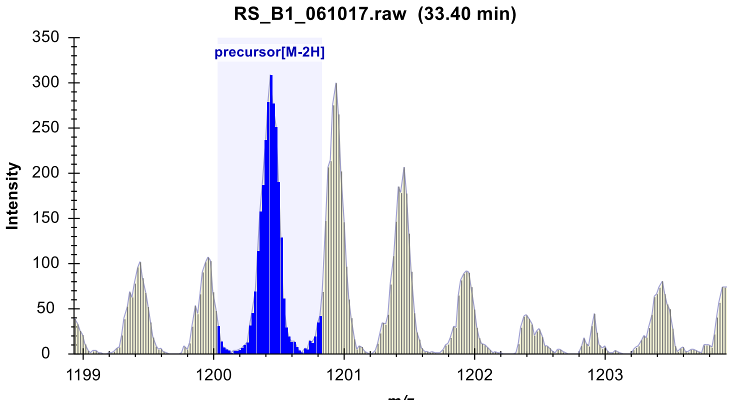

60a

2

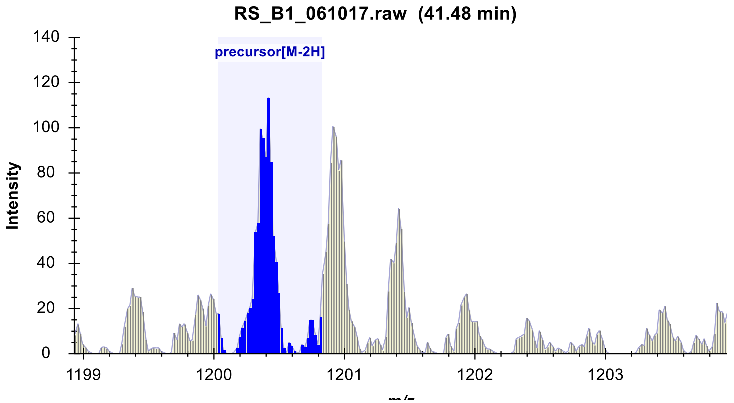

60b

3

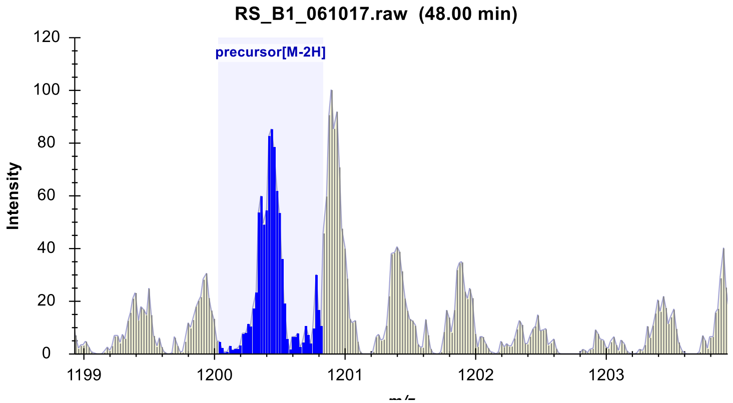

60c

CID-MS/MS
(m/z 1200.43 $^{2-}$)

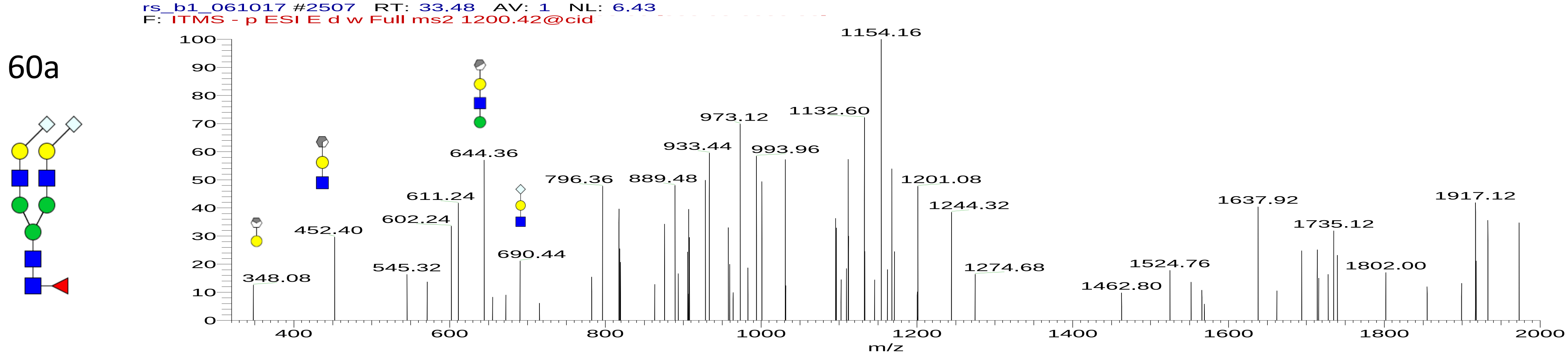

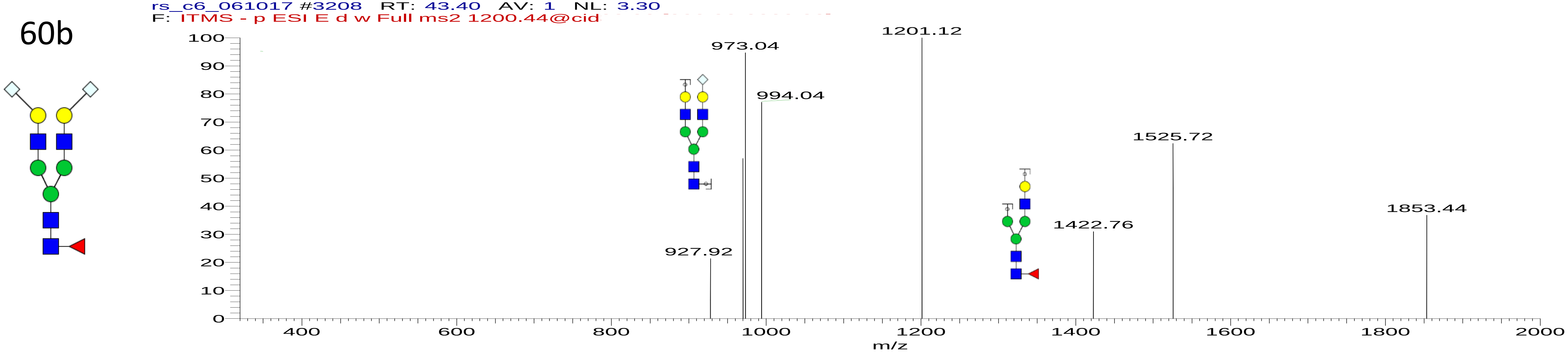

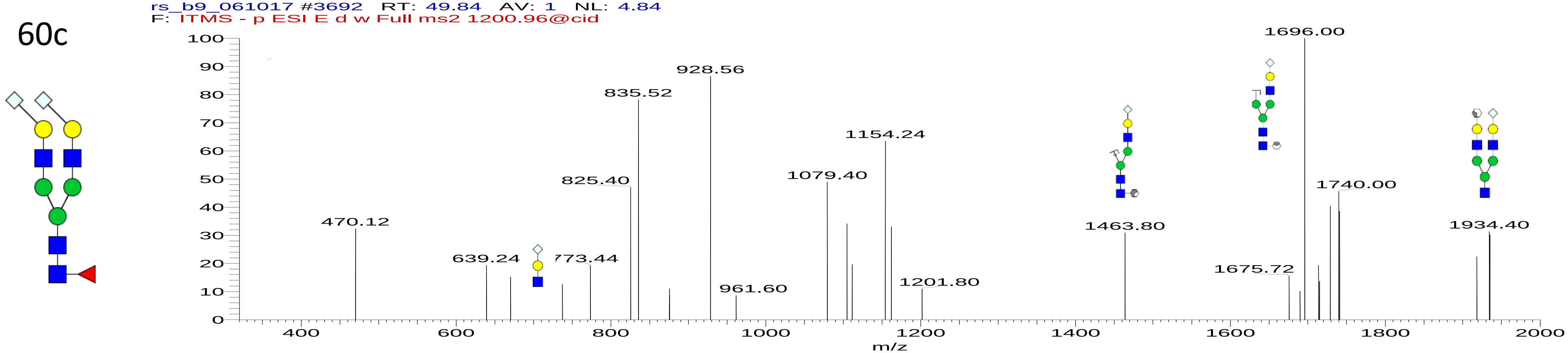

(Hex)1 (HexNAc)3 (Deoxyhexose)1 (NeuAc)2 + (Man)3(GlcNAc)2
GlytouCan ID: G56309UJ
[M-2H] 1204.94

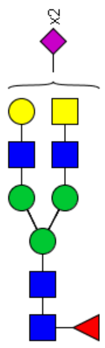

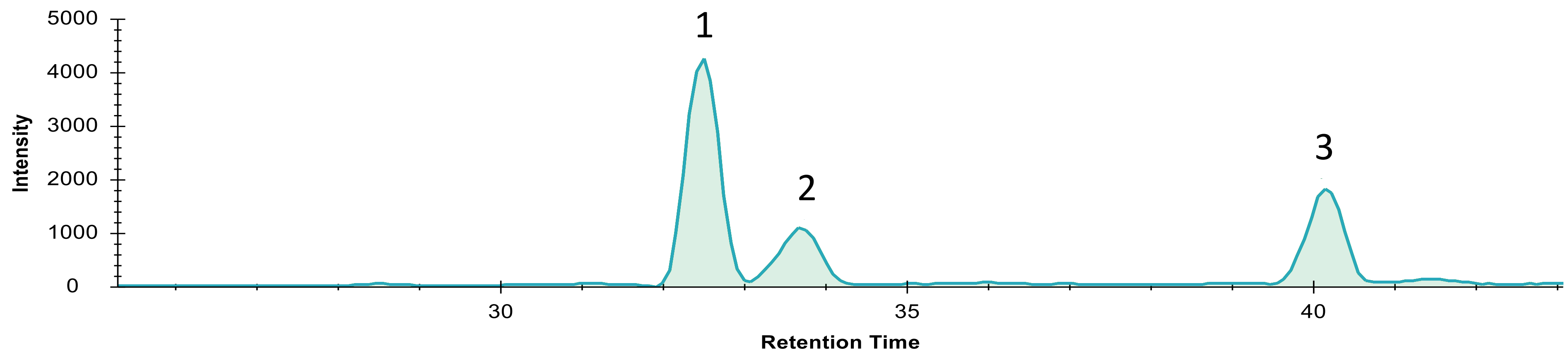

1

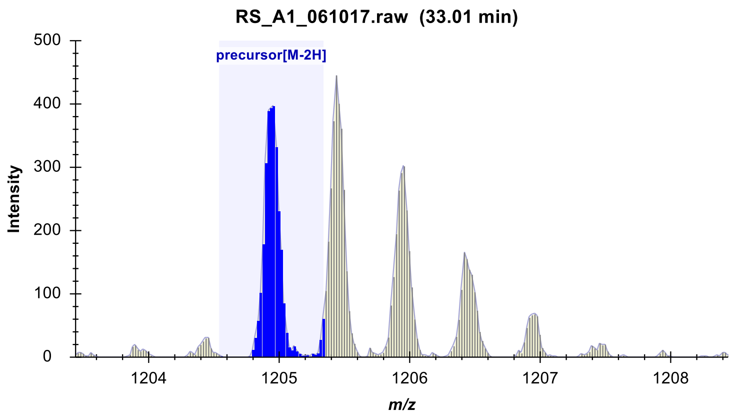

61a

2

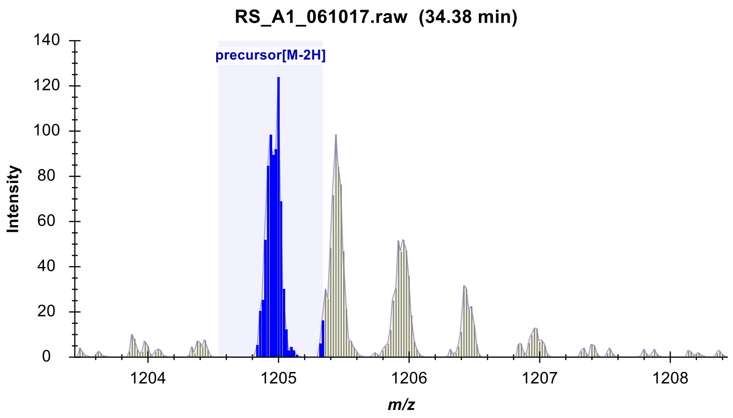

61b

3

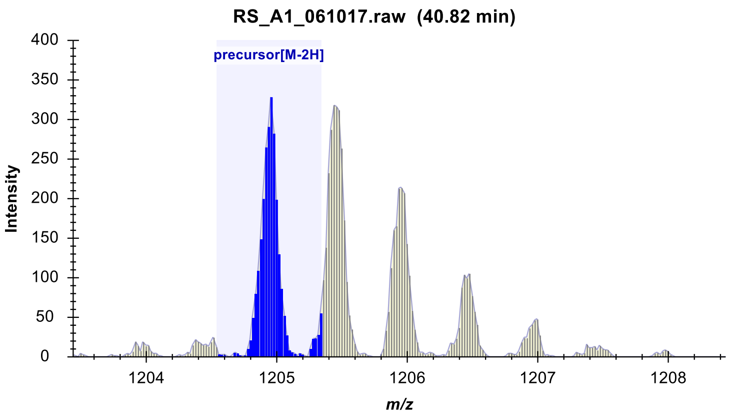

61c

GlytouCan ID: G56309UJ

CID-MS/MS
(m/z 1204.94 $^{2-}$)

61a

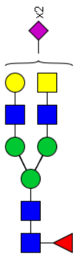

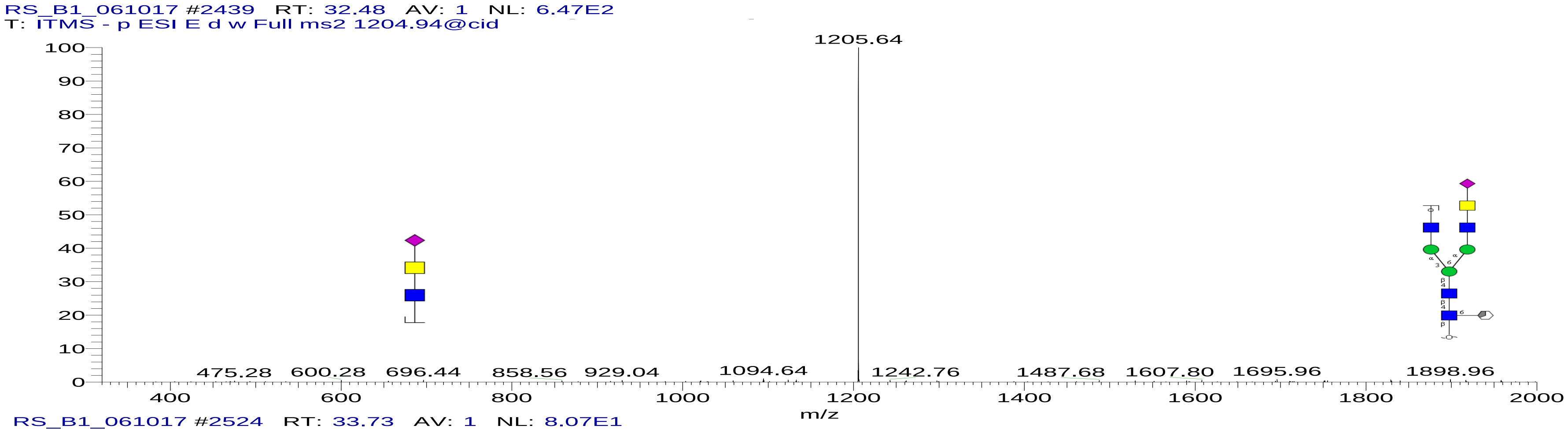

61b

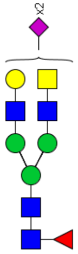

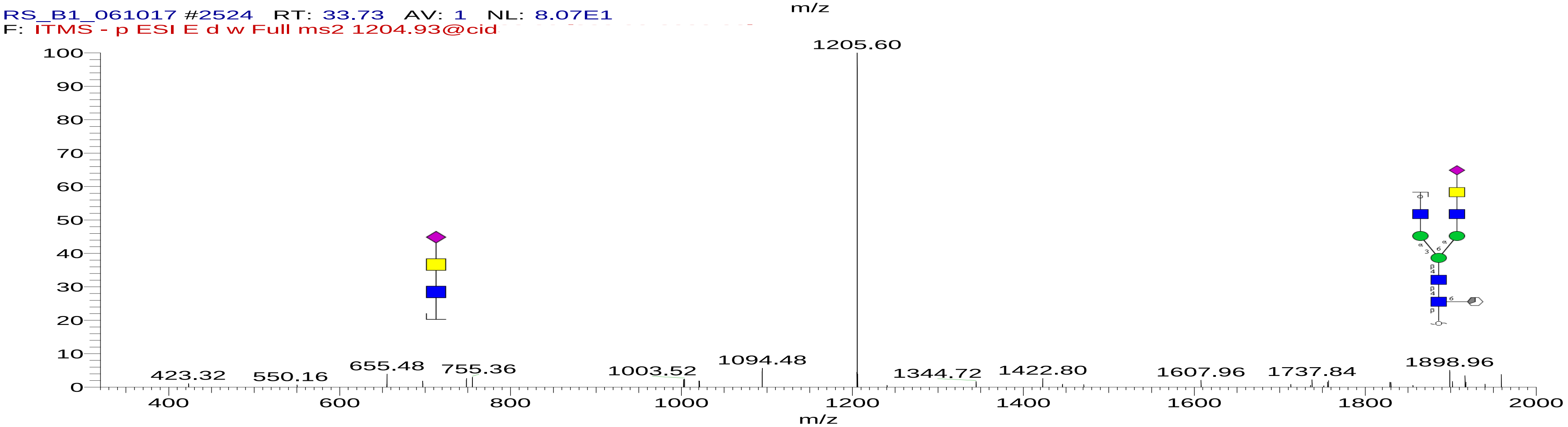

61c

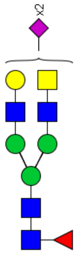

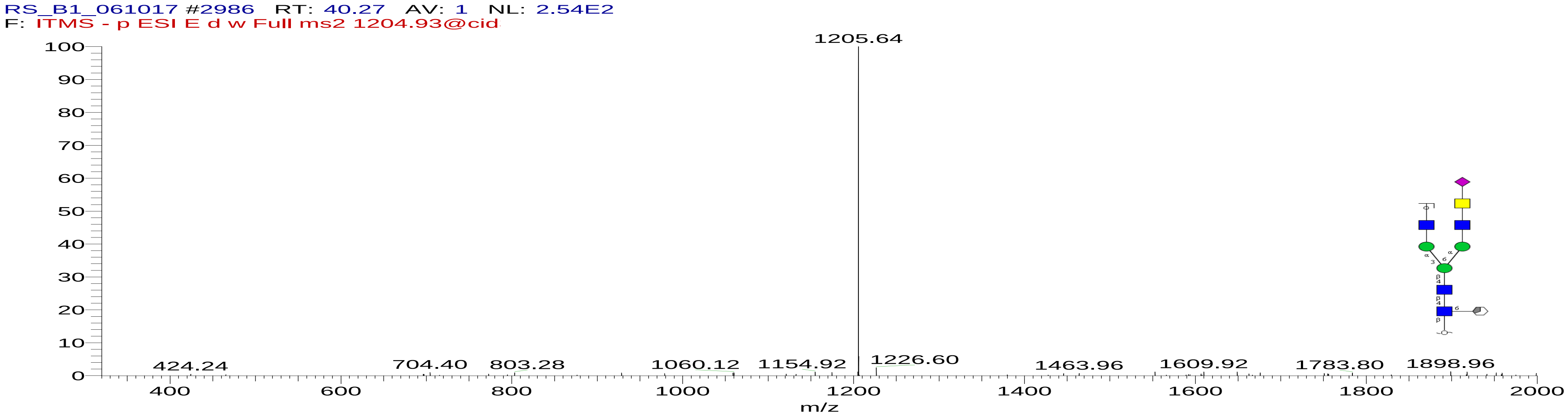

(Hex)2 (HexNAc)3 (NeuAc)2 + (Man)3(GlcNAc)2
No GlytouCan ID
[M-2H] 1212.93

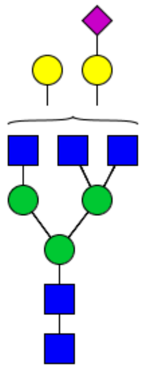

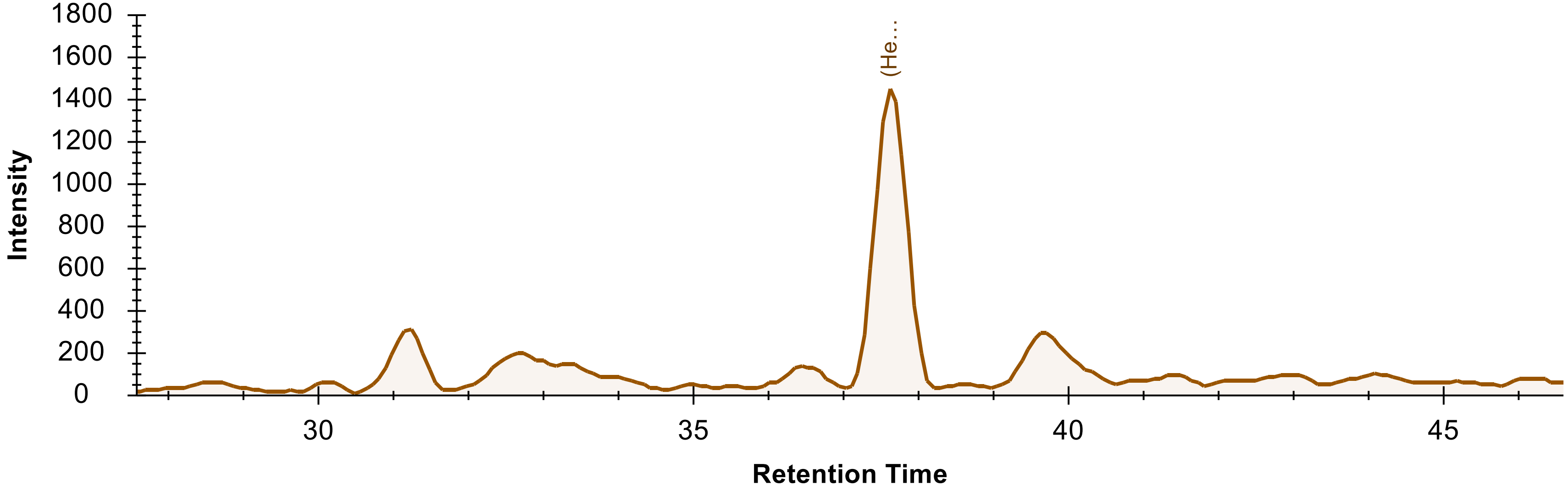

CID-MS/MS (*m/z* 1212.93 [2-])

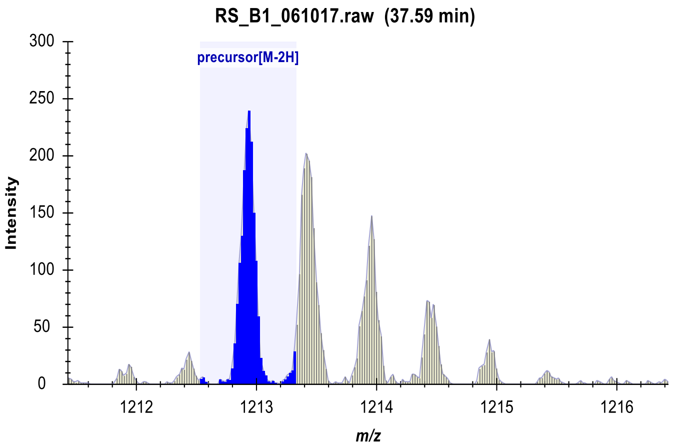

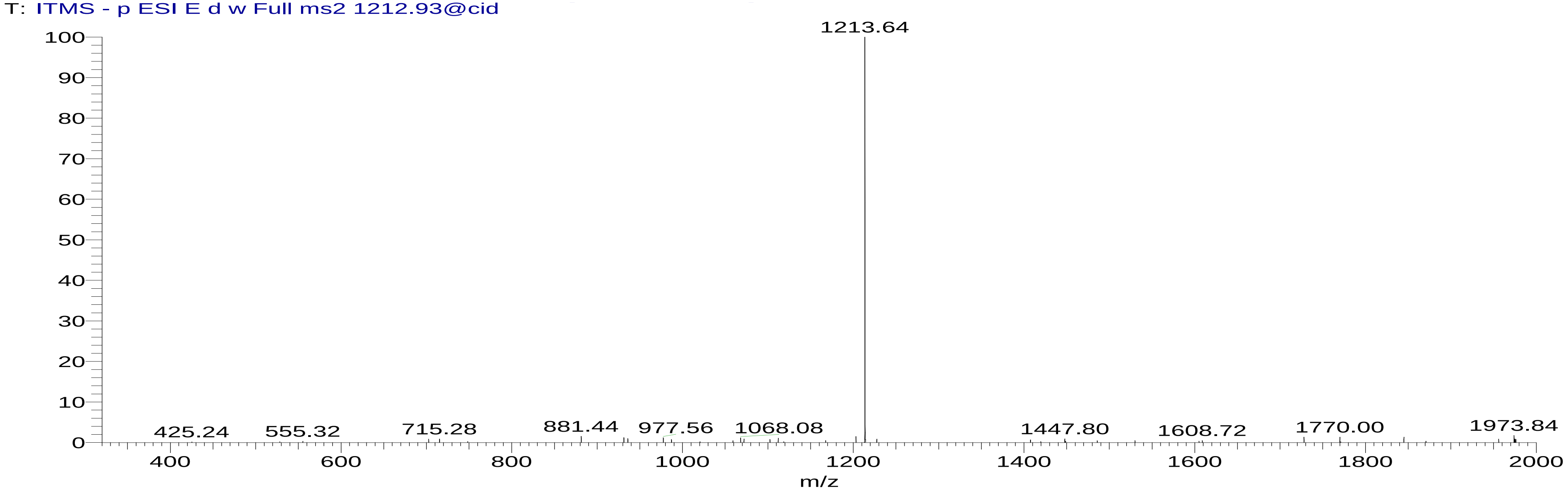

Glycan ID: 62

(Hex)3 (HexNAc)3 (Deoxyhexose)1 (NeuAc)1 + (Man)3(GlcNAc)2

GlytouCan ID: G13441RZ

[M-2H] 1221.44

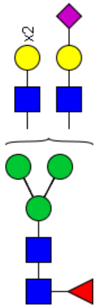

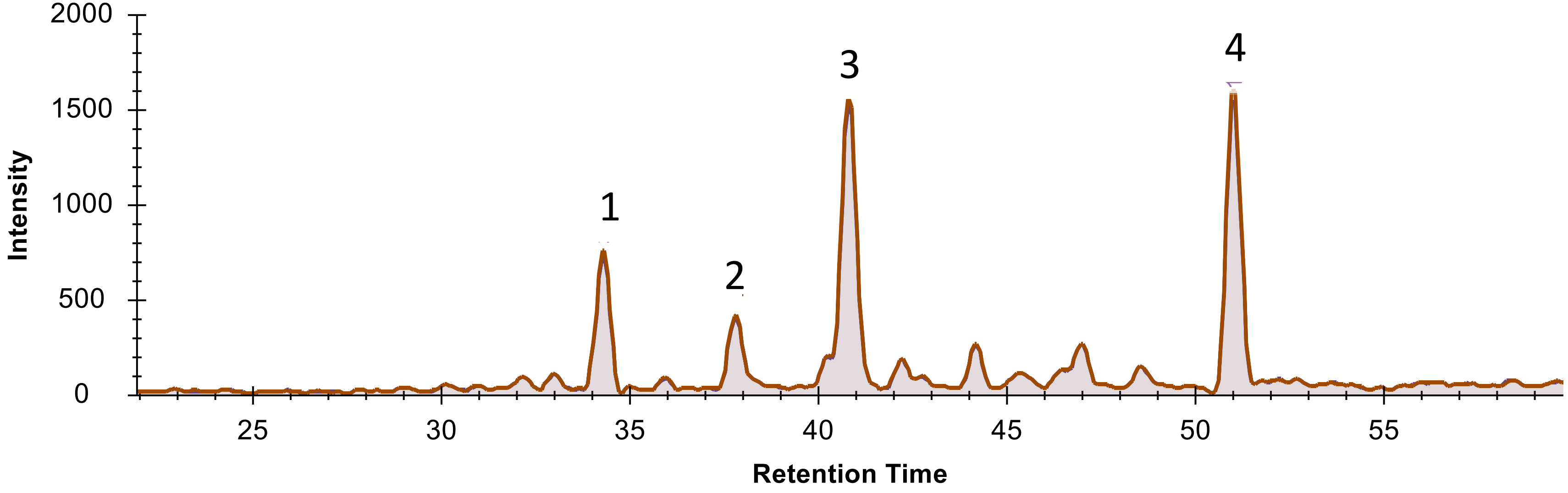

1

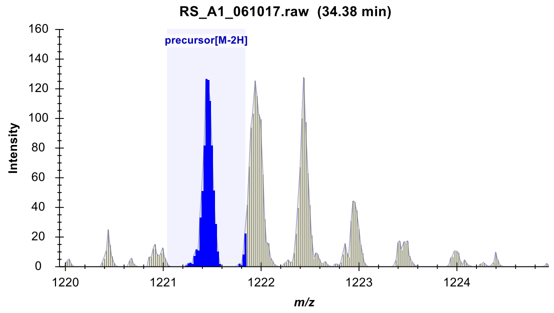

63a

2

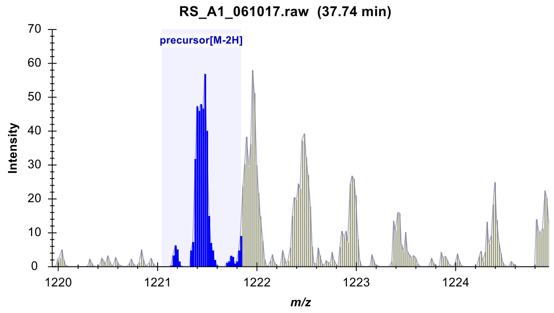

63b

3

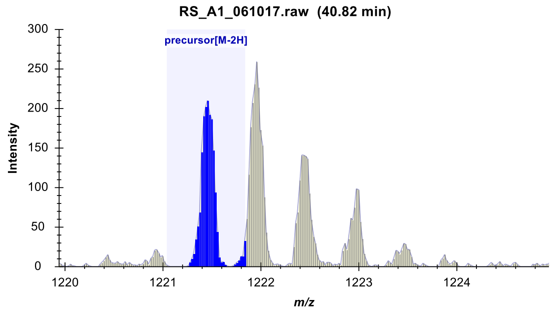

63c

4

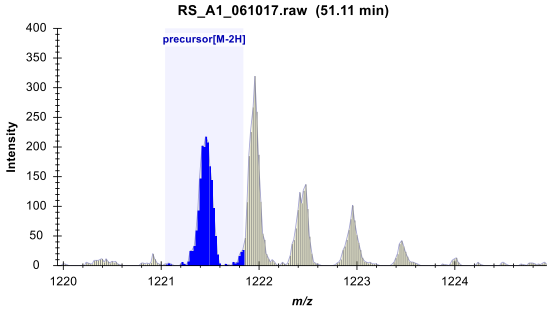

63d

GlytouCan ID: G13441RZ

CID-MS/MS
(m/z 1221.44 $^{1-}$)

63a

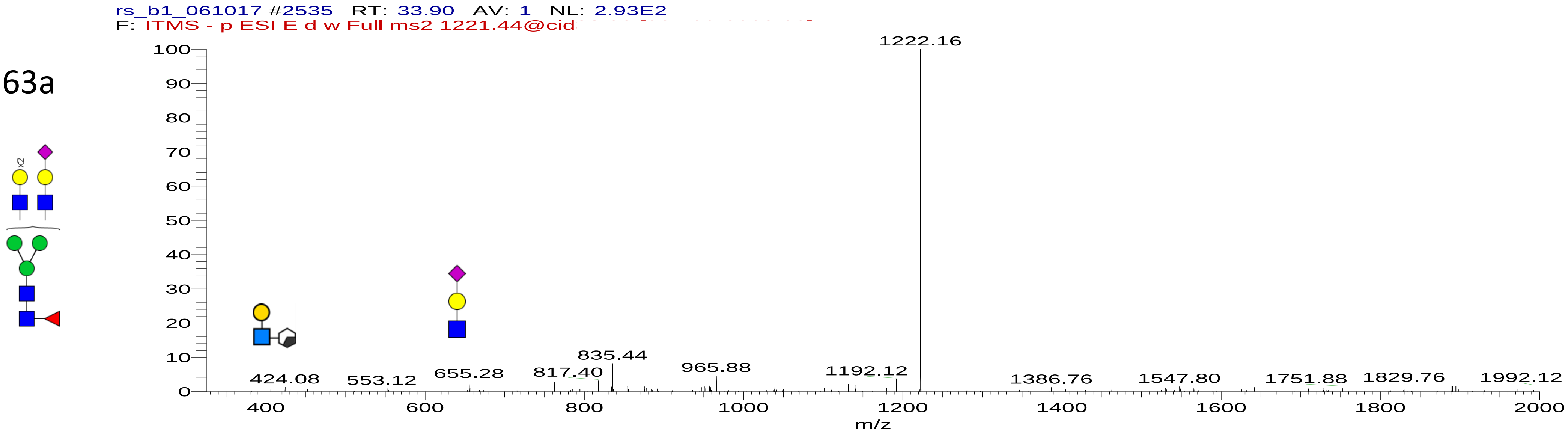

63b

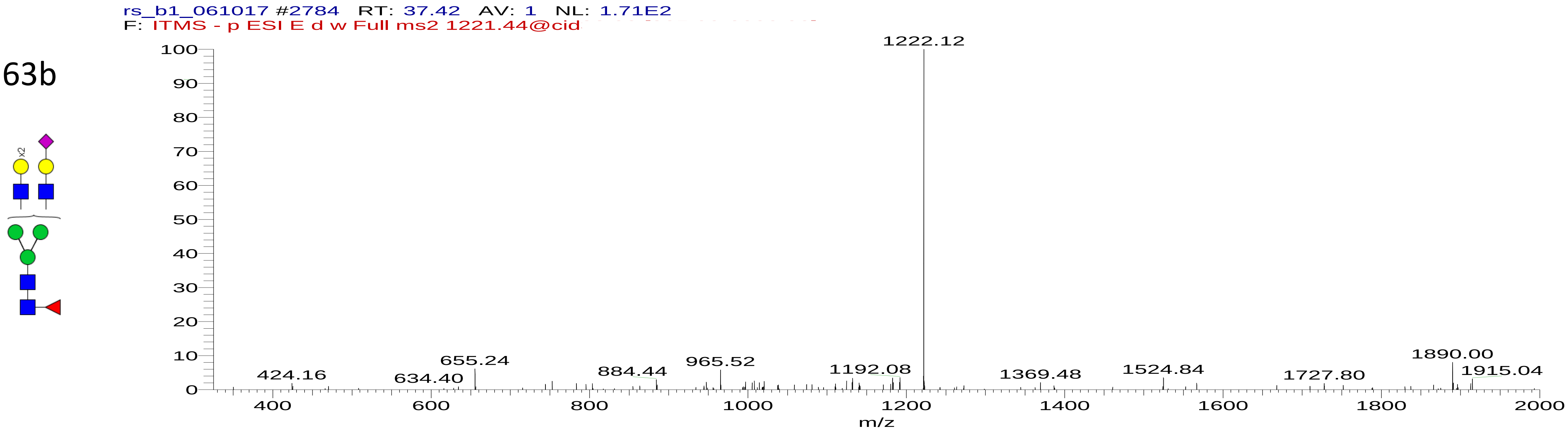

GlytouCan ID: G13441RZ

CID-MS/MS

(m/z 1221.44 $^{1-}$)

63c

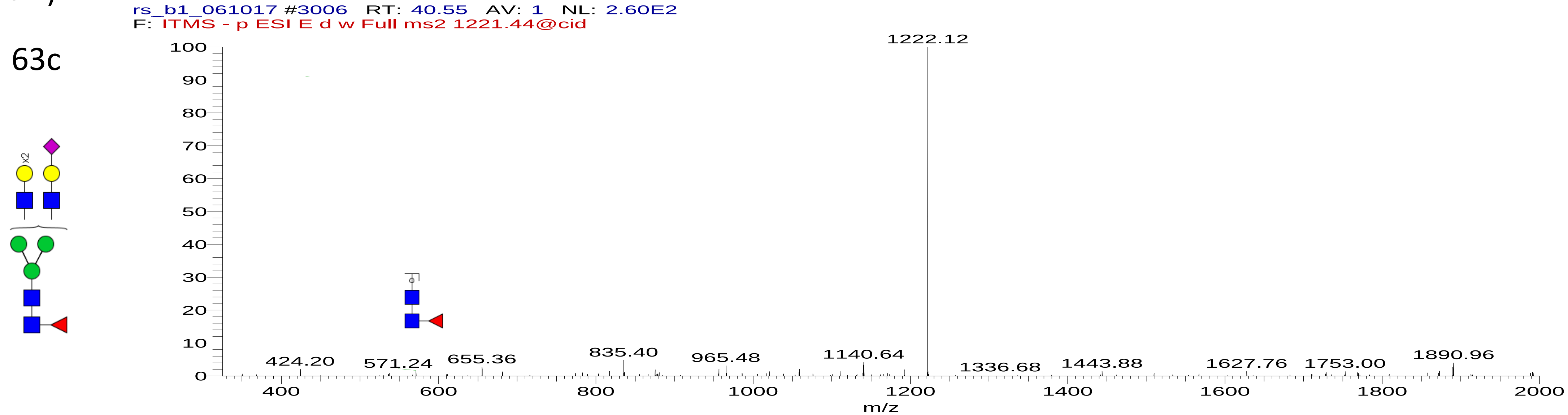

63d

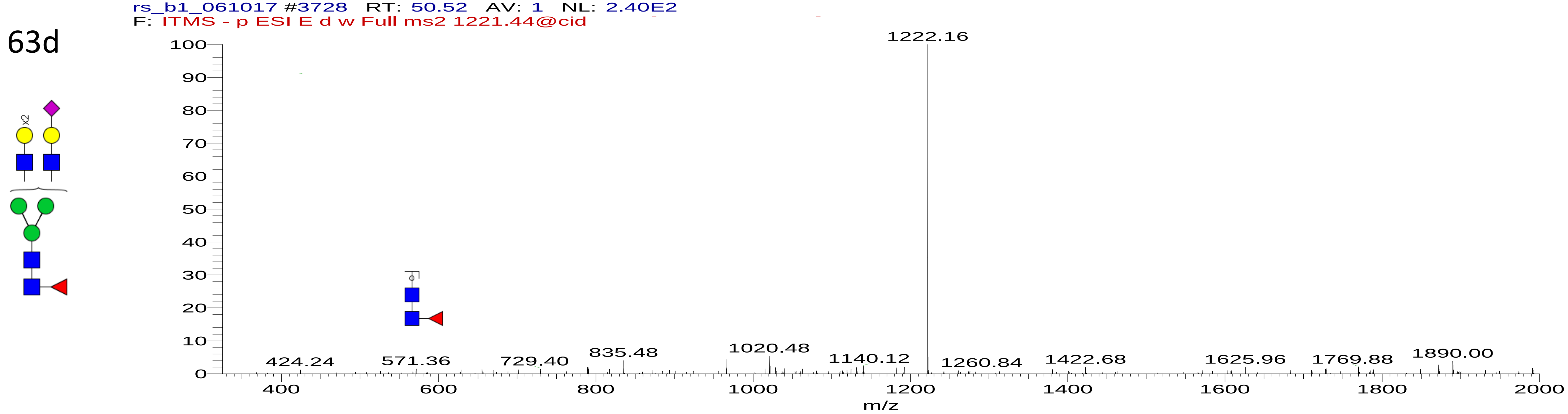

(HexNAc)4 (Deoxyhexose)1 (NeuAc)2 + (Man)3(GlcNAc)2

[M-2H] 1225.45

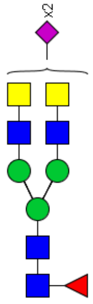

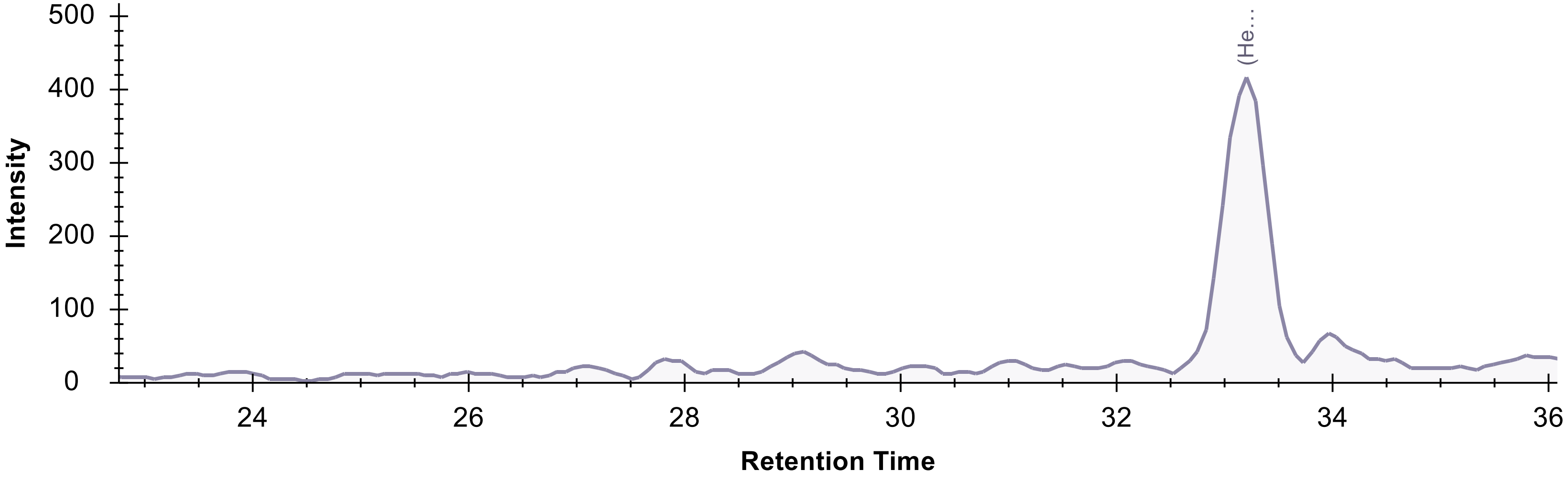

CID-MS/MS (*m/z* 1225.45 $^{2-}$)

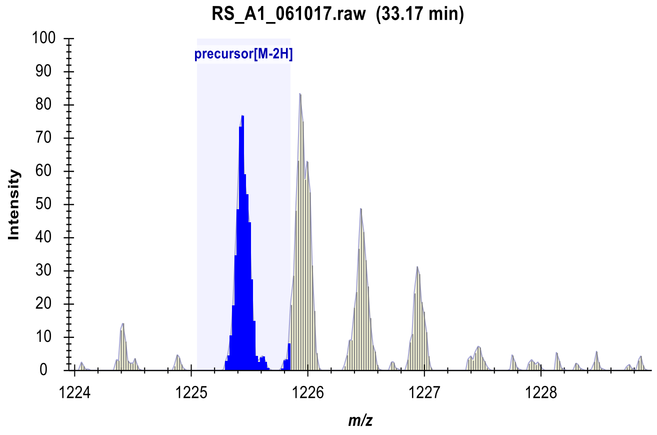

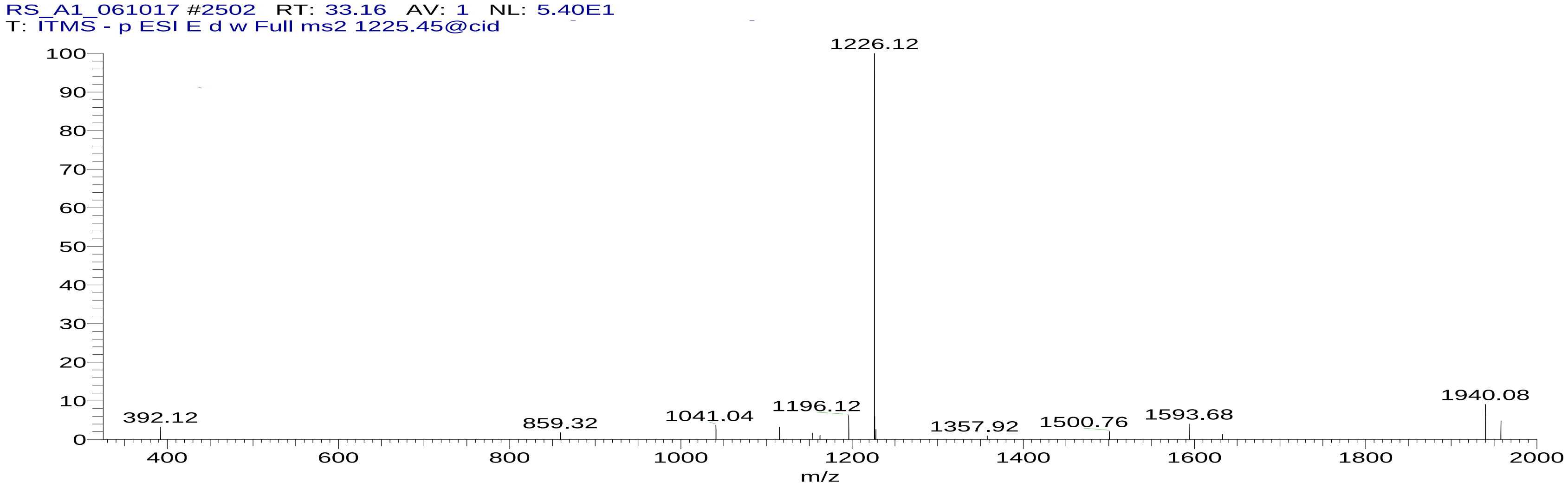

Glycan ID: 64

(Hex)2 (HexNAc)3 (Deoxyhexose)1 (NeuAc)2 + (Man)3(GlcNAc)2

GlytouCan ID: G79553XF

[M-2H] 1285.97

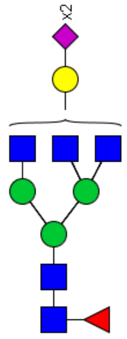

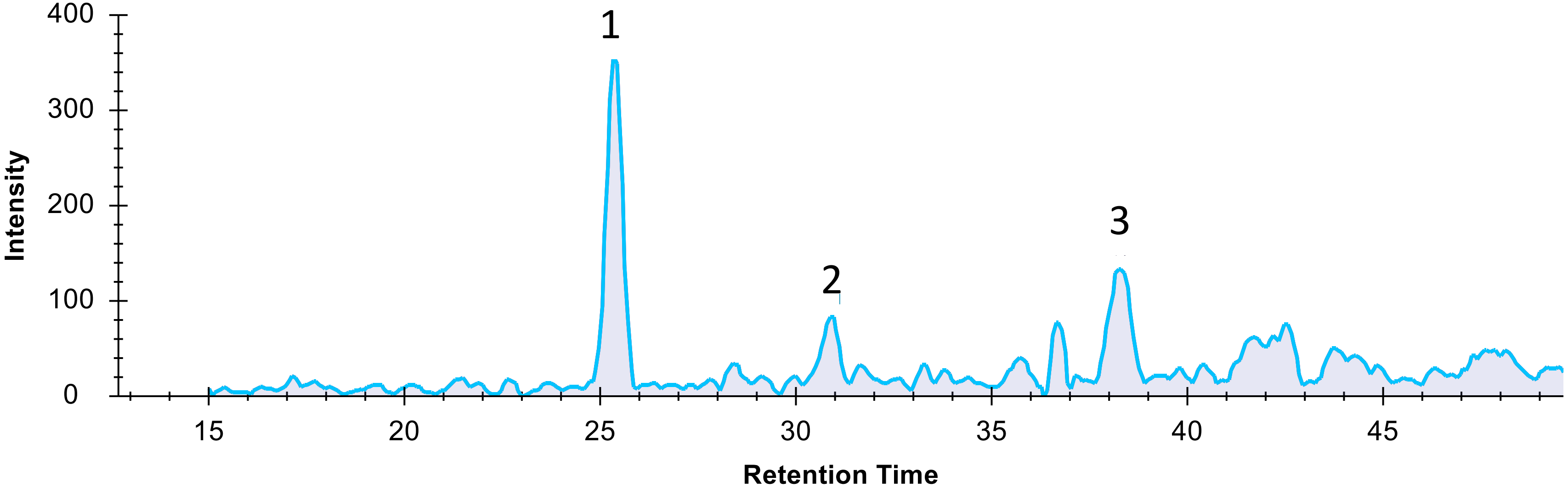

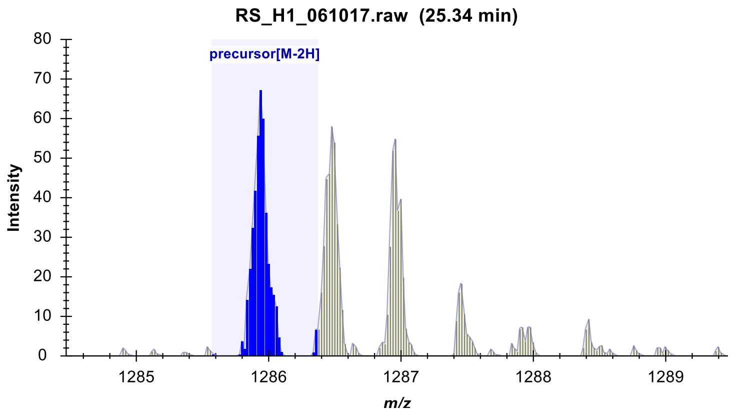

65a

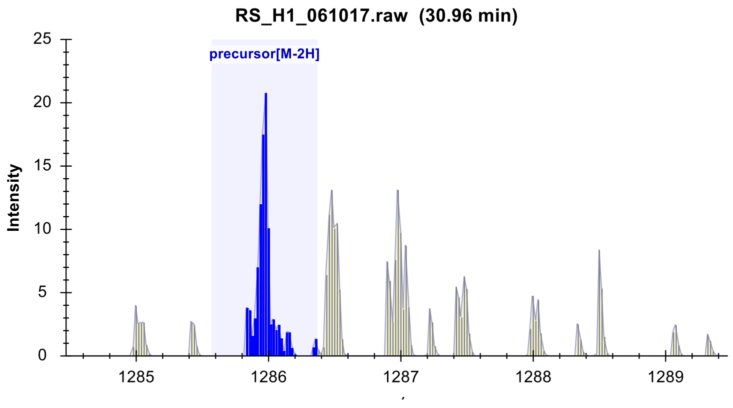

65b

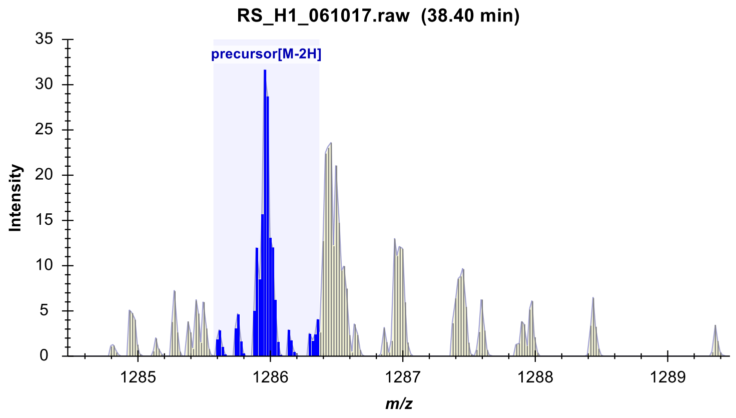

65c

GlytouCan ID: G79553XF

CID-MS/MS
(m/z 1285.97 $^{2-}$)

65a

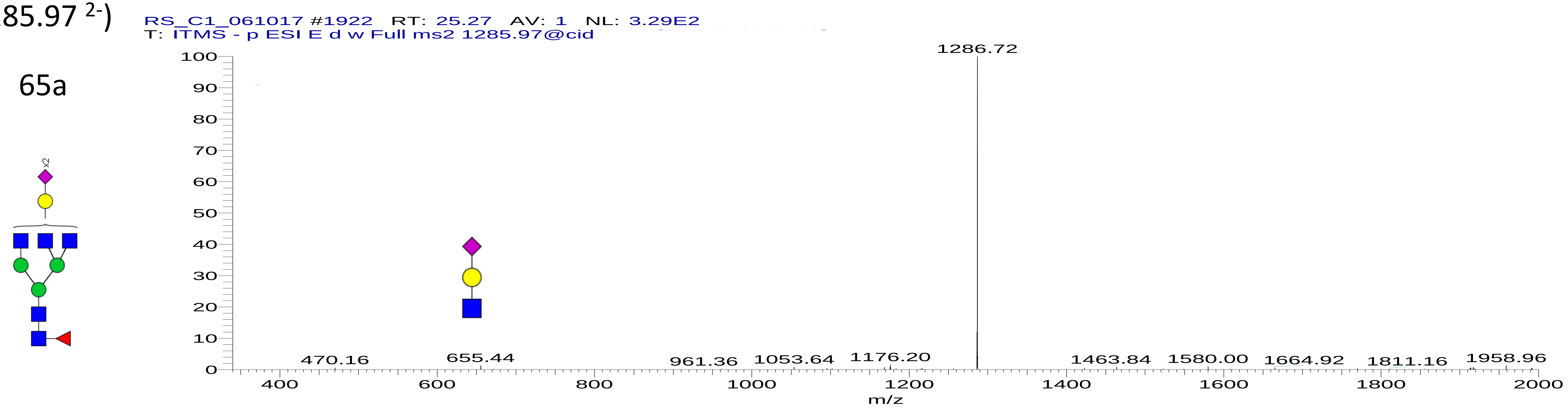

65c

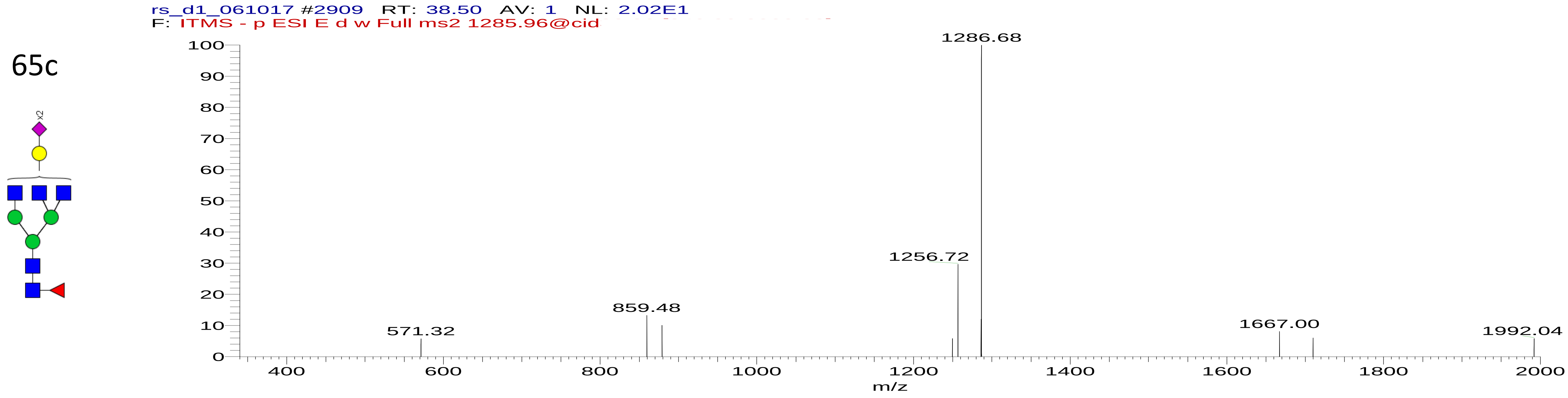

(Hex)3 (HexNAc)3 (NeuAc)2 + (Man)3(GlcNAc)2

GlytouCan: G57551SM

[M-2H] 1293.98

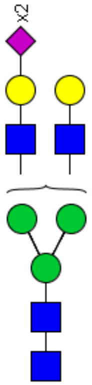

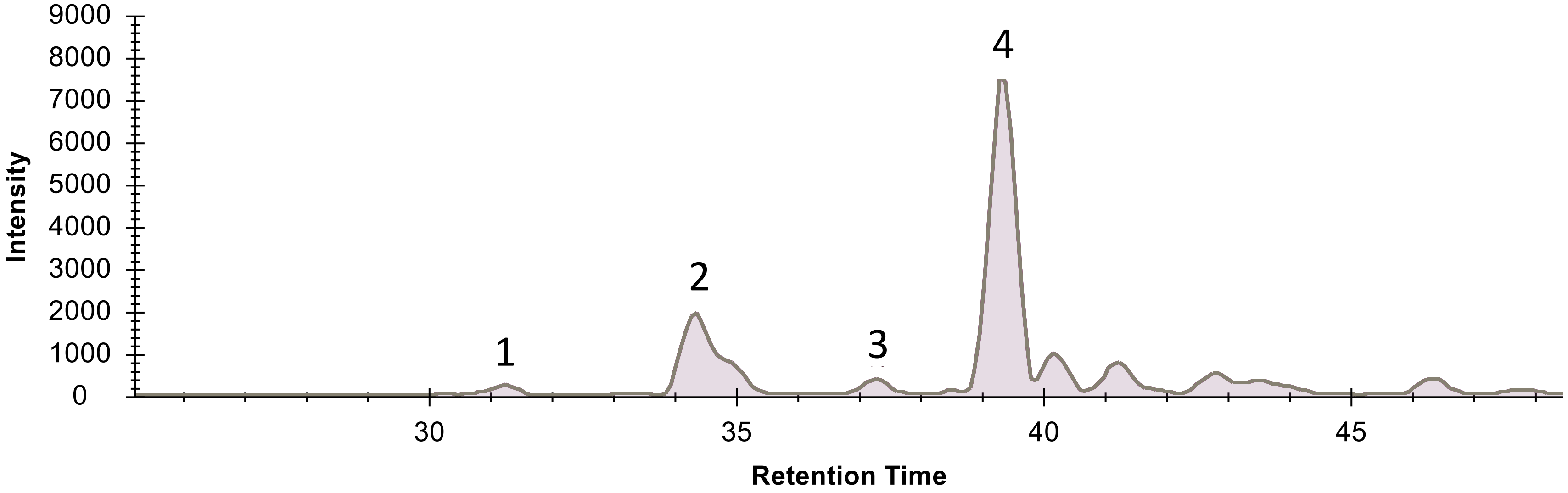

1

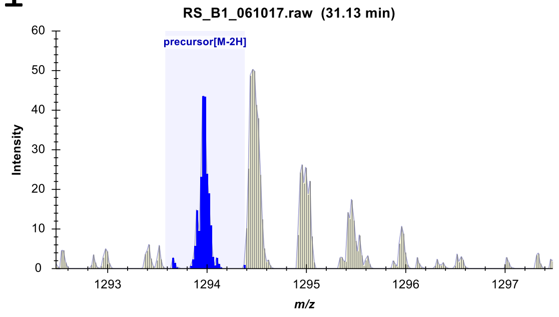

66a

2

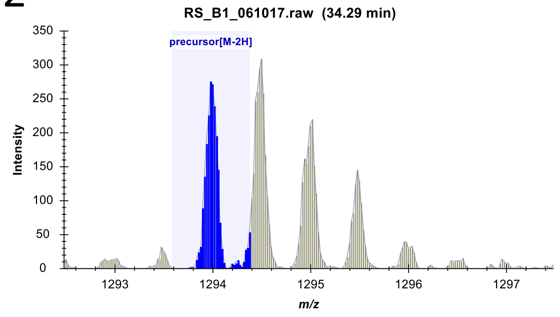

66b

3

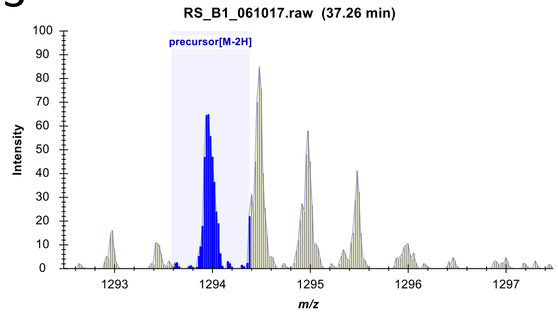

66c

4

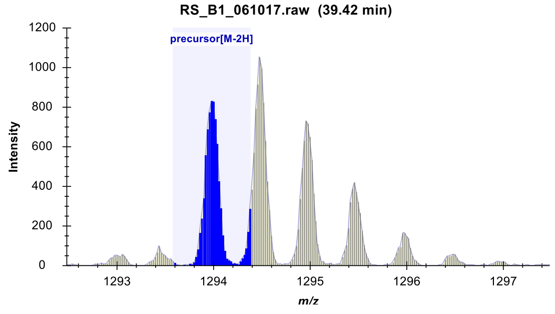

66d

GlytouCan: G57551SM

CID-MS/MS
(m/z 1294.00 $^{2-}$)

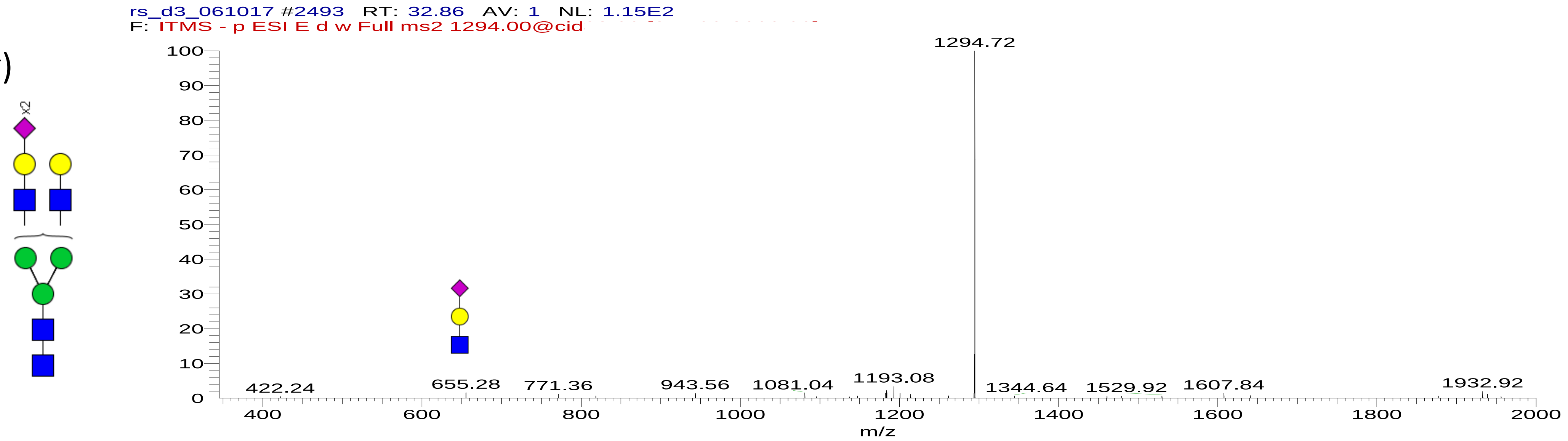

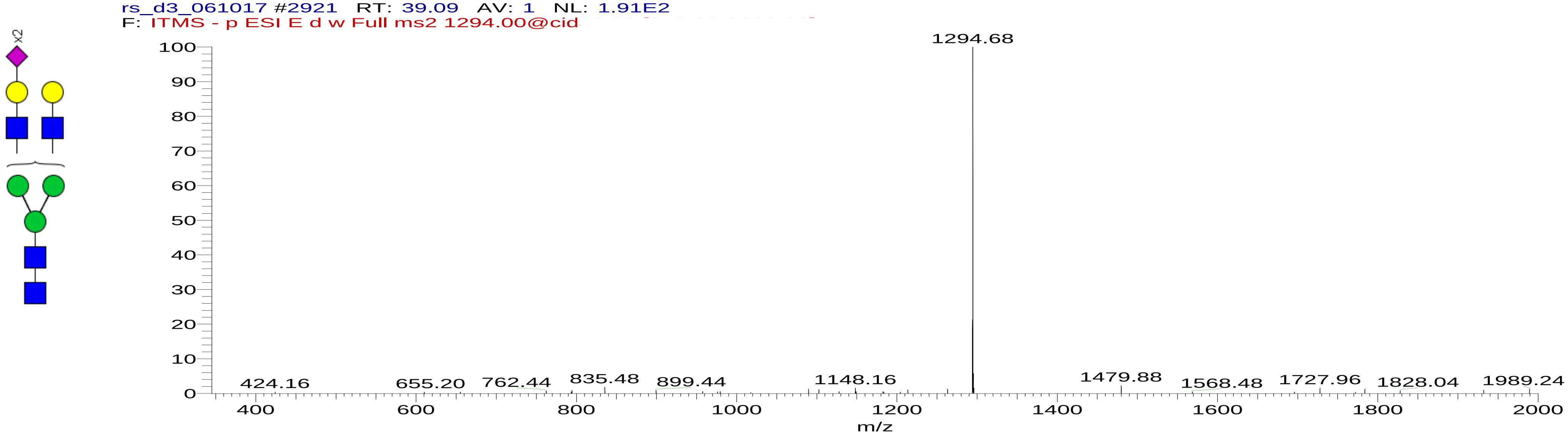

GlytouCan: G57551SM

CID-MS/MS
(m/z 1294.00 $^{2-}$)

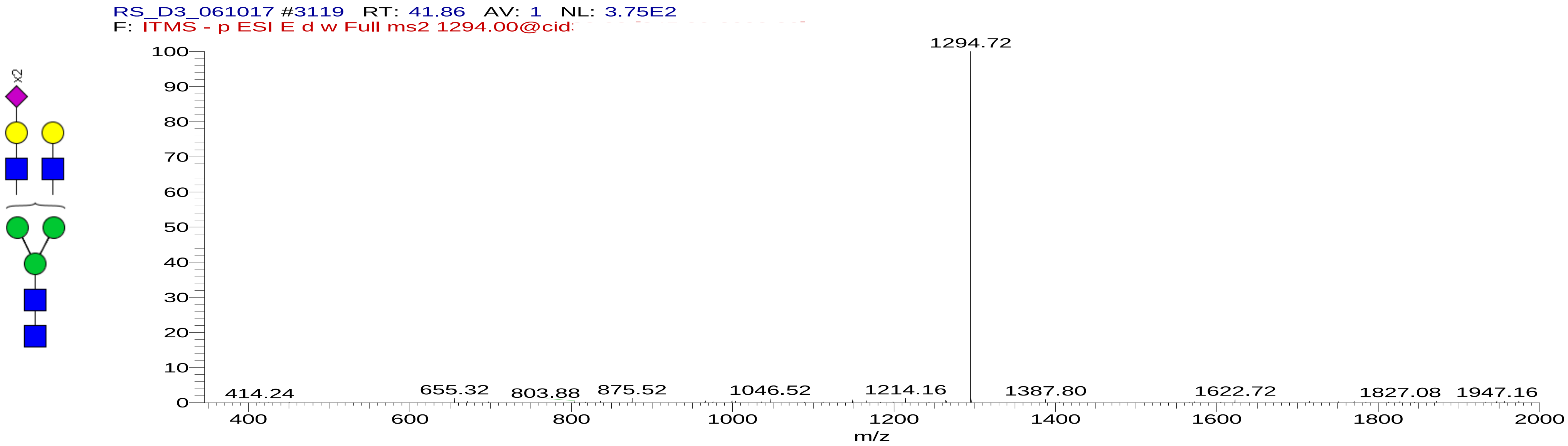

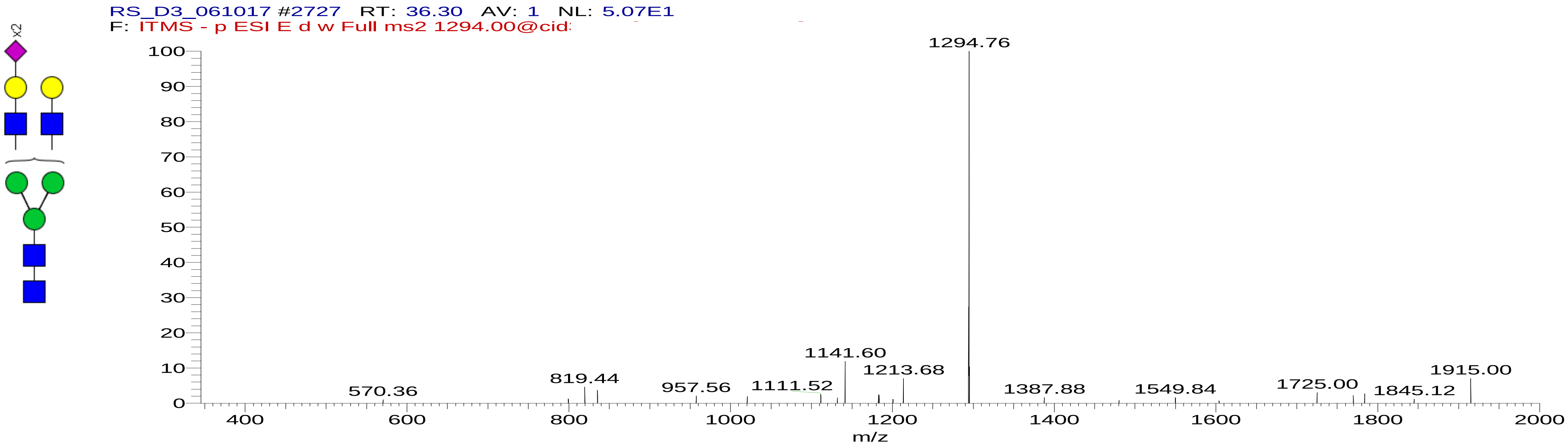

(Hex)2 (HexNAc)2 (Deoxyhexose)1 (NeuAc)3 + (Man)3(GlcNAc)2

No GlytouCan ID

[M-2H] 1330

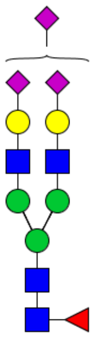

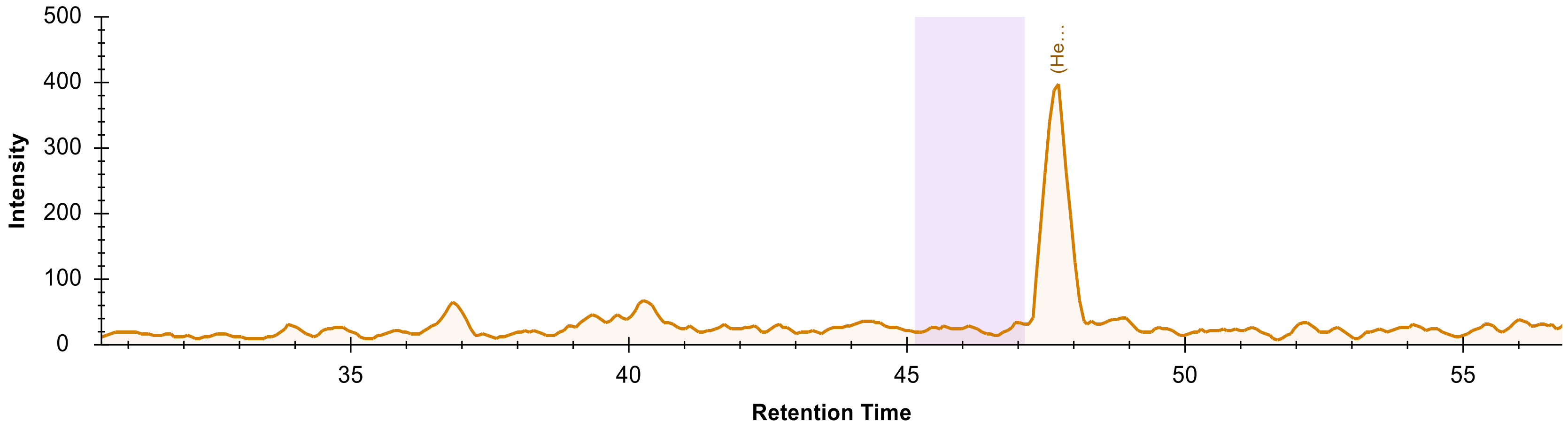

CID-MS/MS (m/z 1330 $^{2-}$)

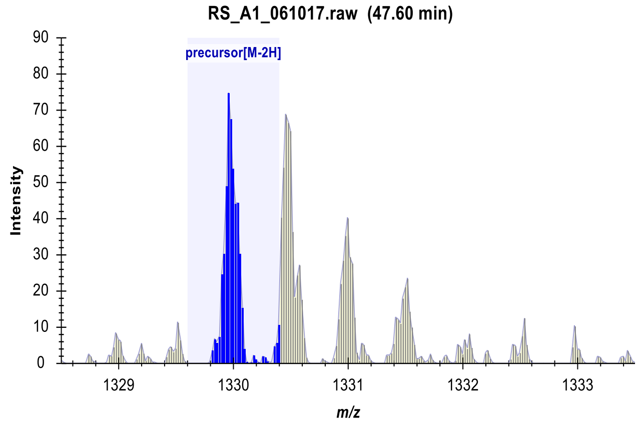

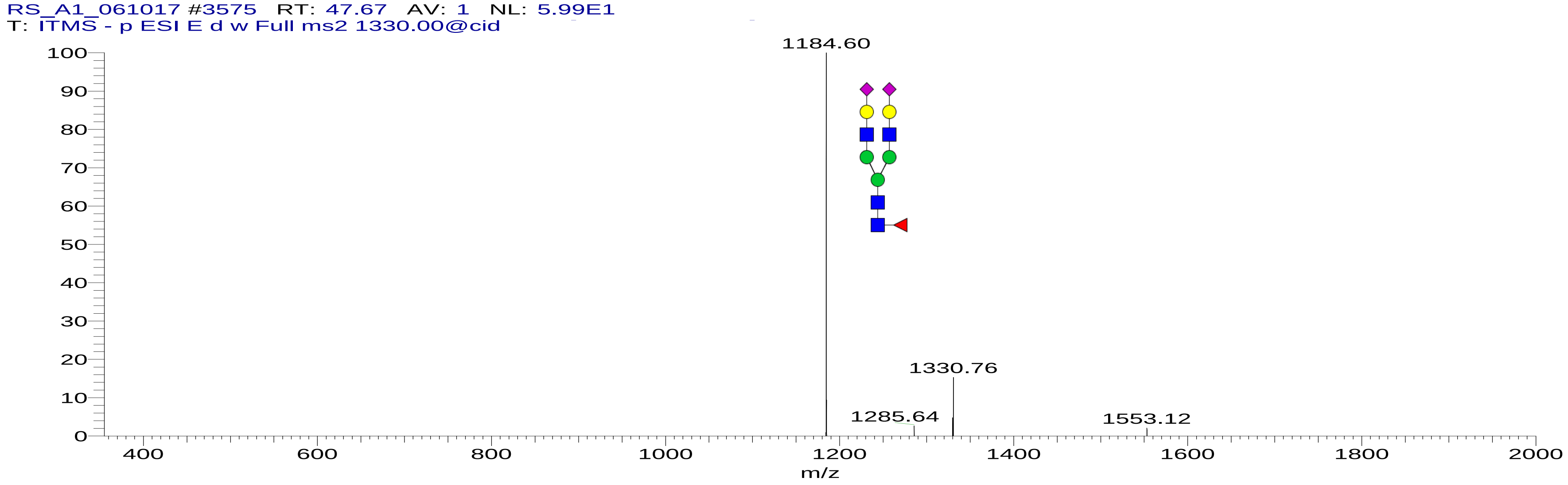

Glycan ID: 67

(Hex)4 (HexNAc)4 (NeuAc)1 + (Man)3(GlcNAc)2

GlytouCan ID: G93952RU

[M-2H] 1330.99

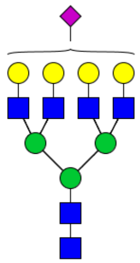

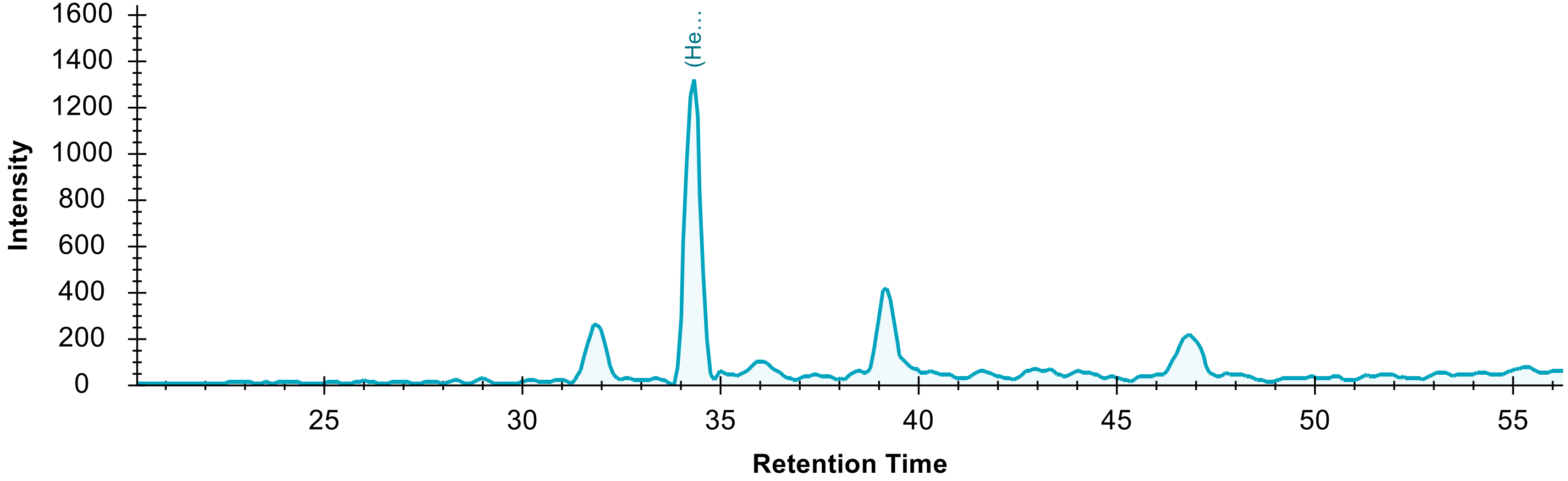

CID-MS/MS (*m/z* 1330.99 [3-])

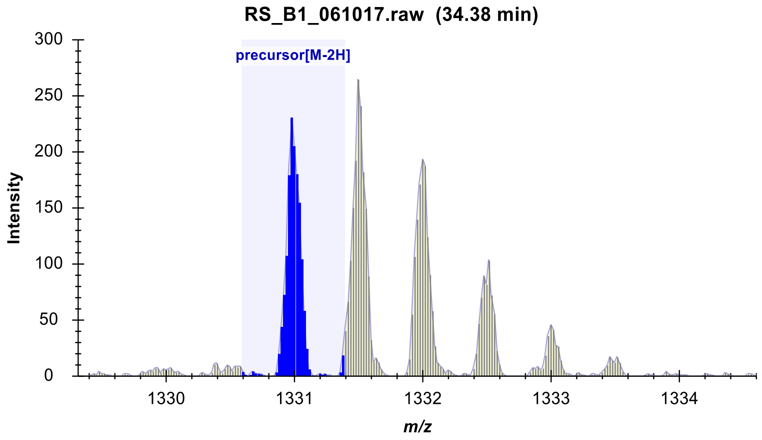

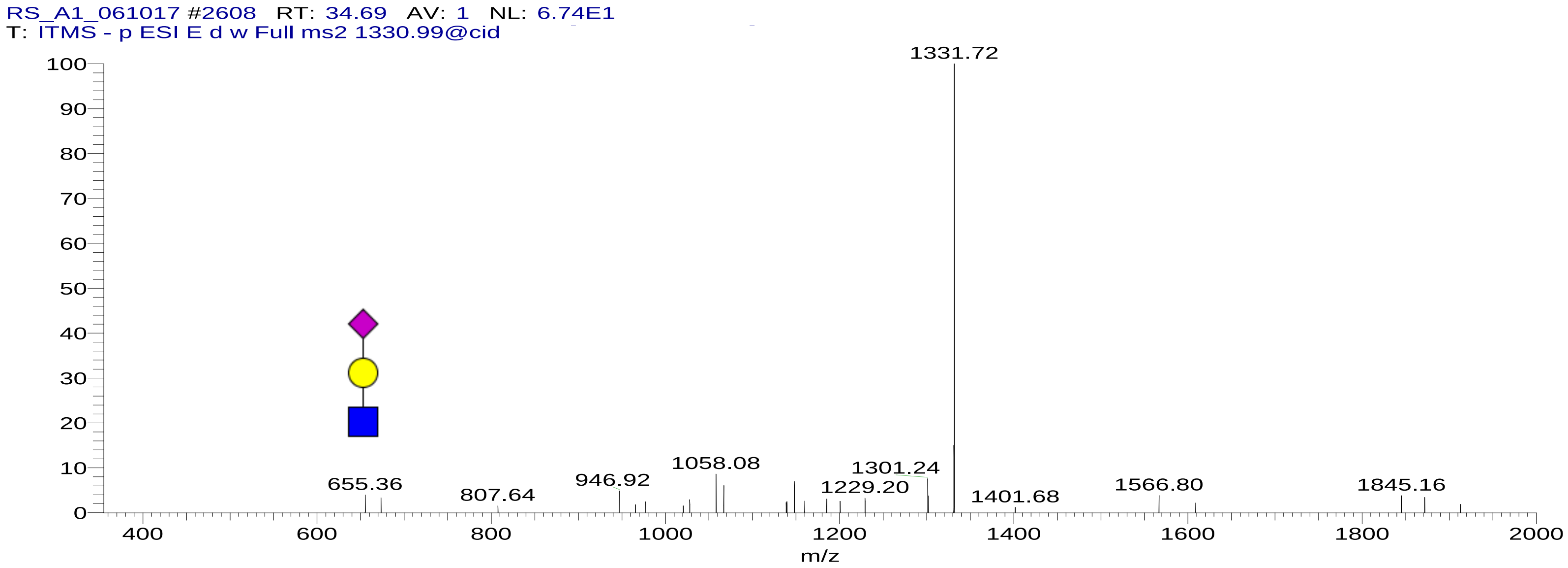

Glycan ID: 68

(Hex)3 (HexNAc)3 (Deoxyhexose)1 (NeuAc)2 + (Man)3(GlcNAc)2

GlytouCan ID: G04256WF

[M-2H] 1367.01

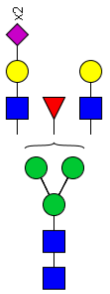

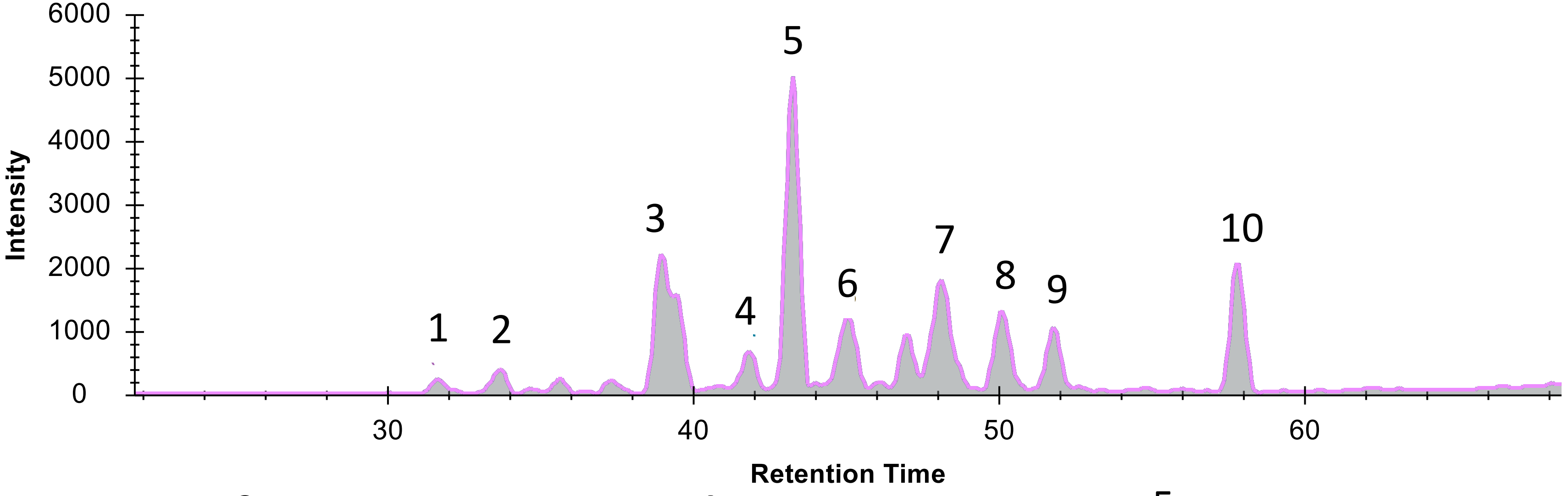

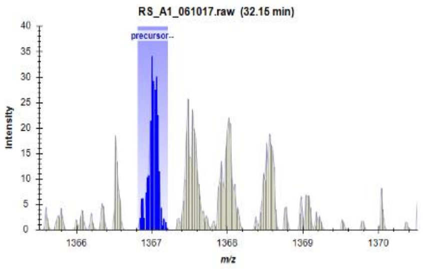

69a

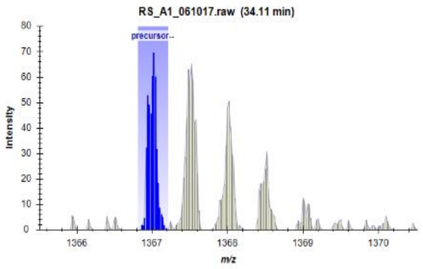

69b

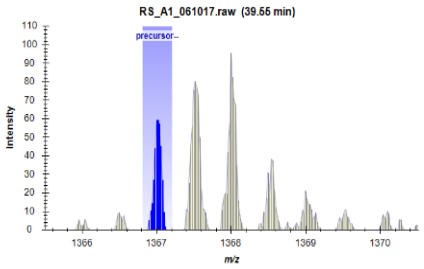

69c

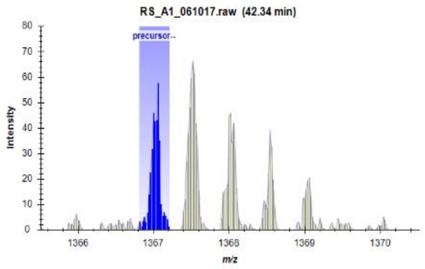

69d

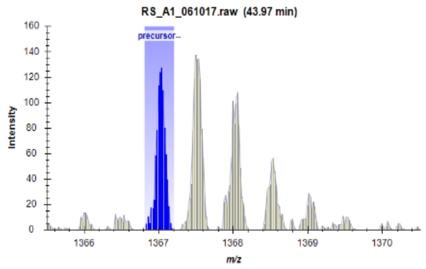

69e

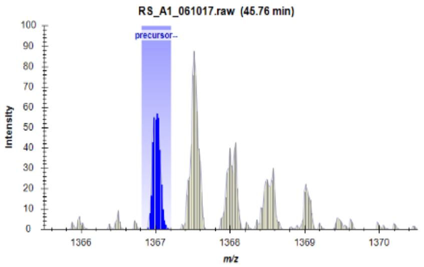

69f

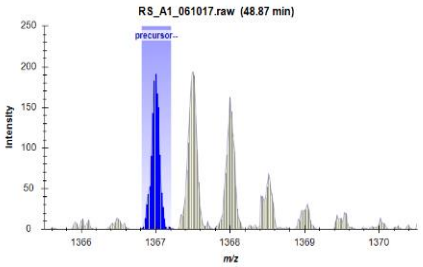

69g

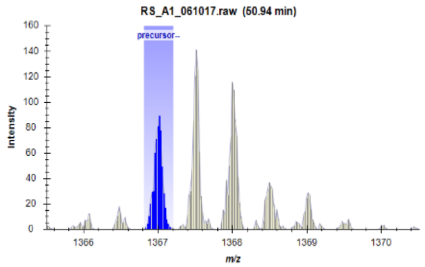

69h

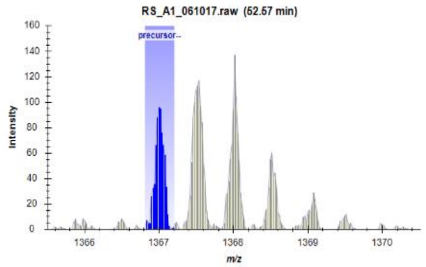

69i

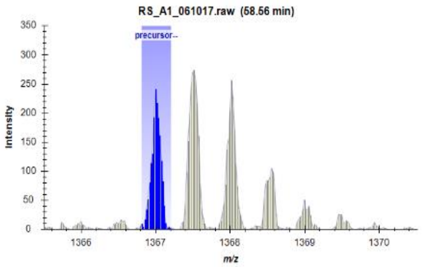

69j

GlytouCan ID: G04256WF

CID-MS/MS
(*m/z* 1367.52 $^{2-}$)

69a

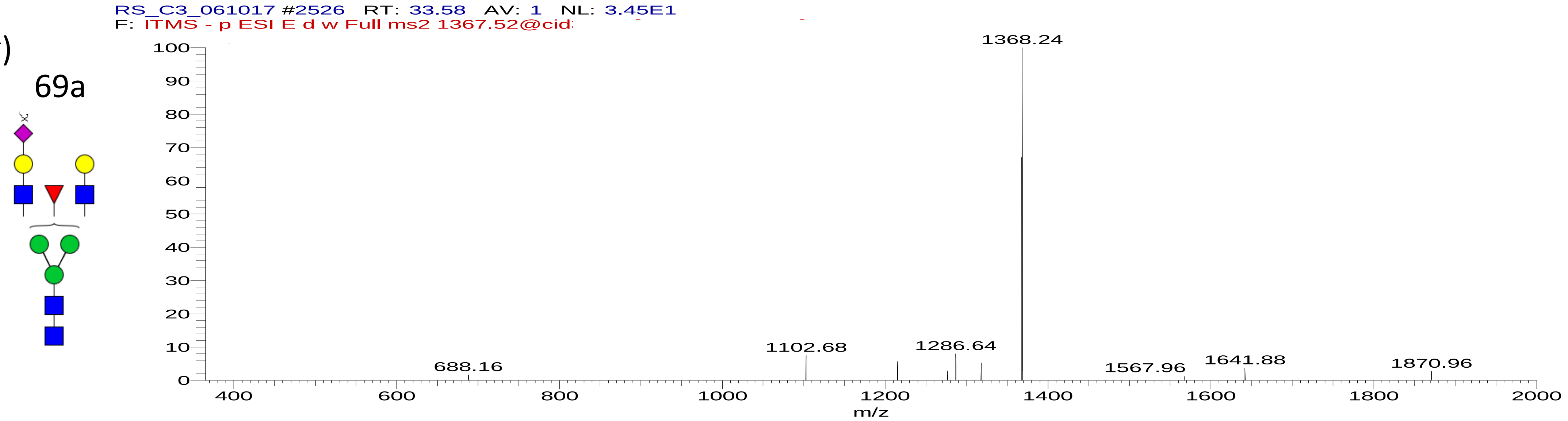

69b

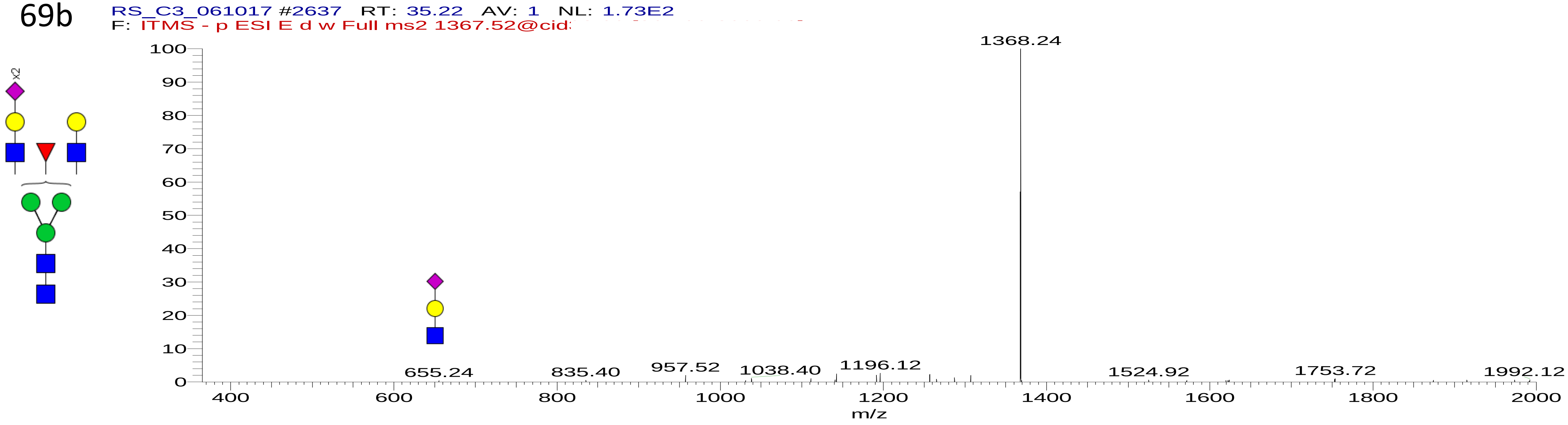

GlytouCan ID: G04256WF

CID-MS/MS

(*m/z* 1367.52 $^{2-}$)

69c

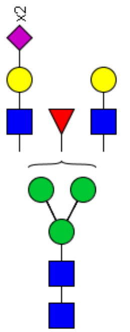

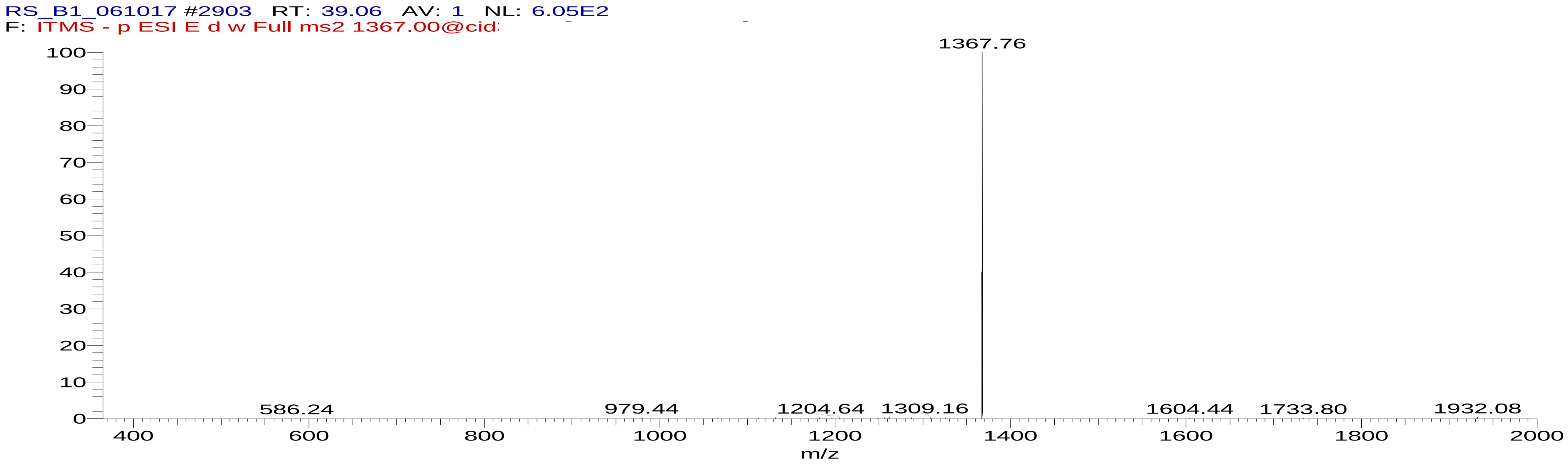

69d

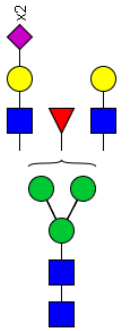

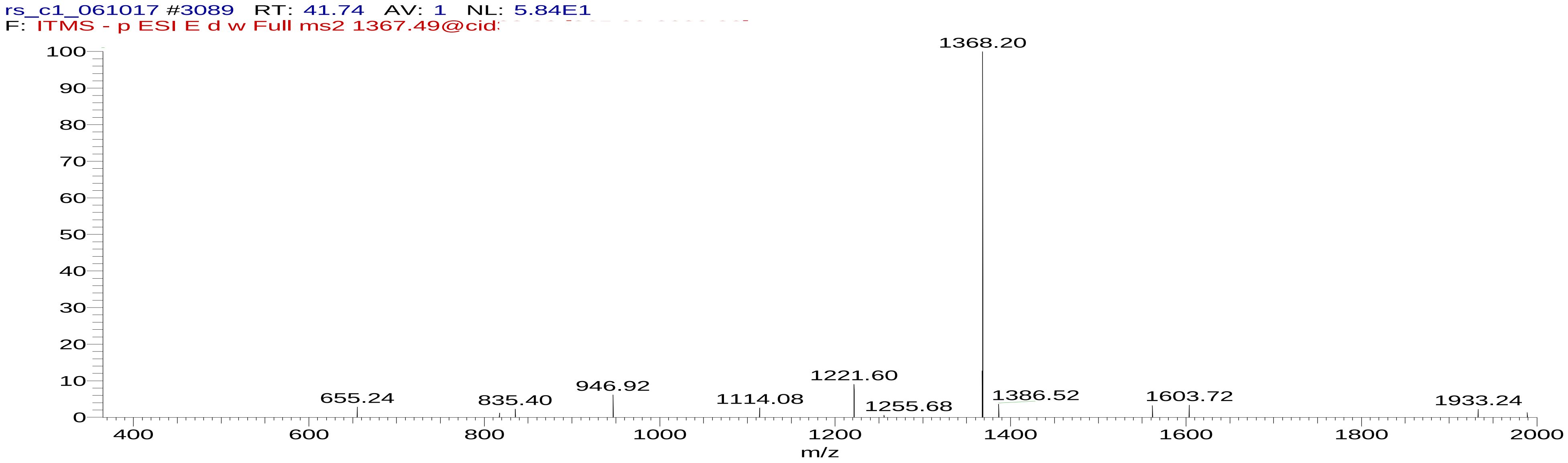

GlytouCan ID: G04256WF

CID-MS/MS
(m/z 1367.52 $^{2-}$)

69e

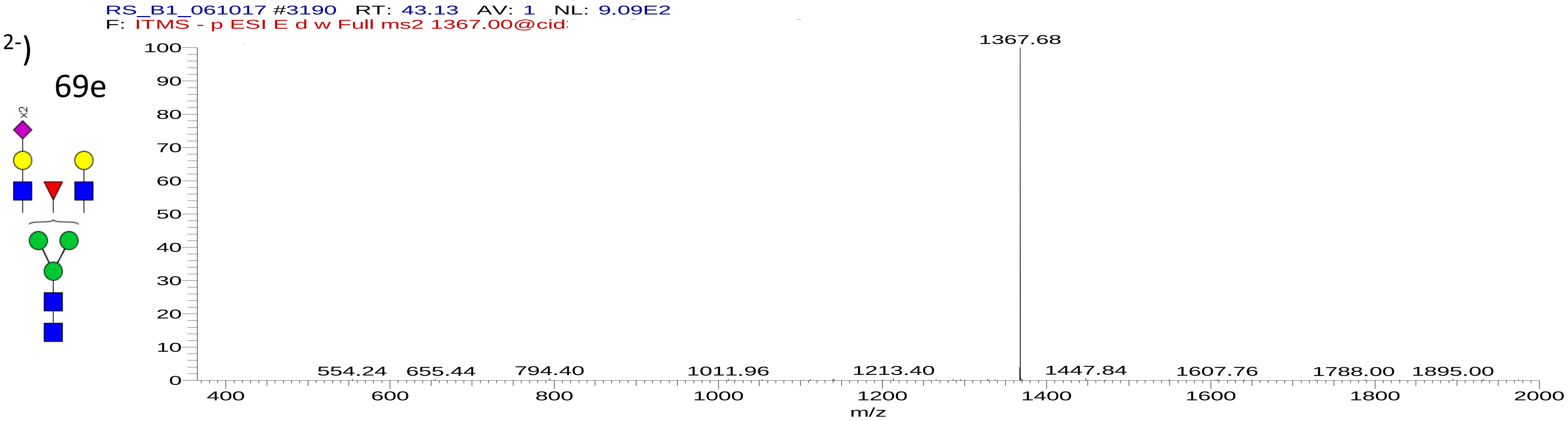

69f

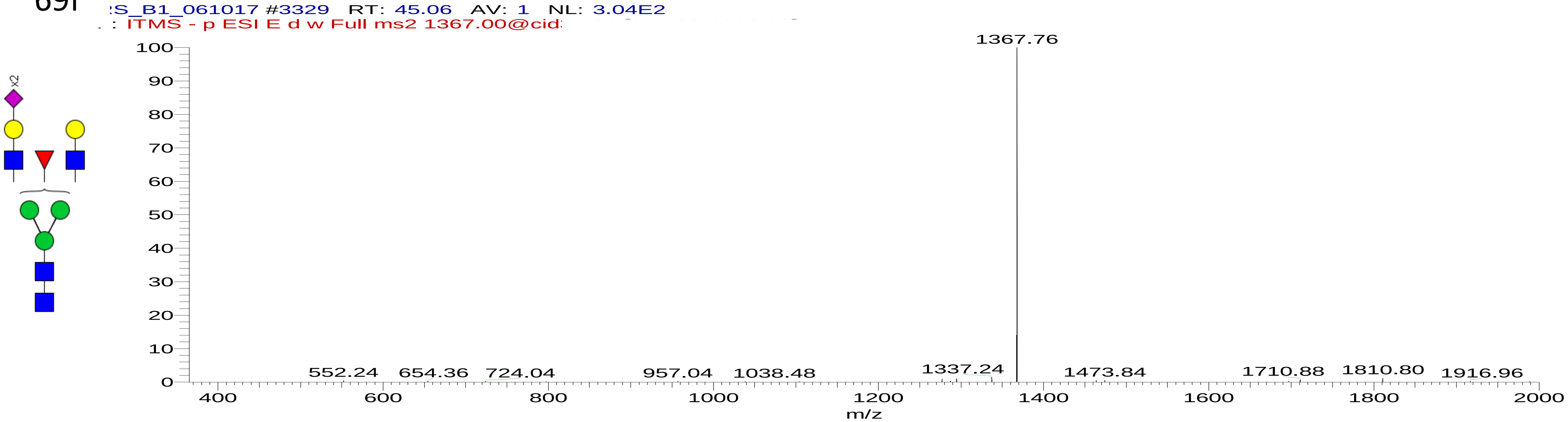

GlytouCan ID: G04256WF

CID-MS/MS
(m/z 1367.52 $^{2-}$)

69g

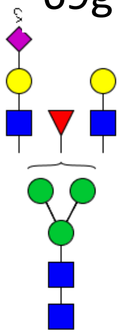

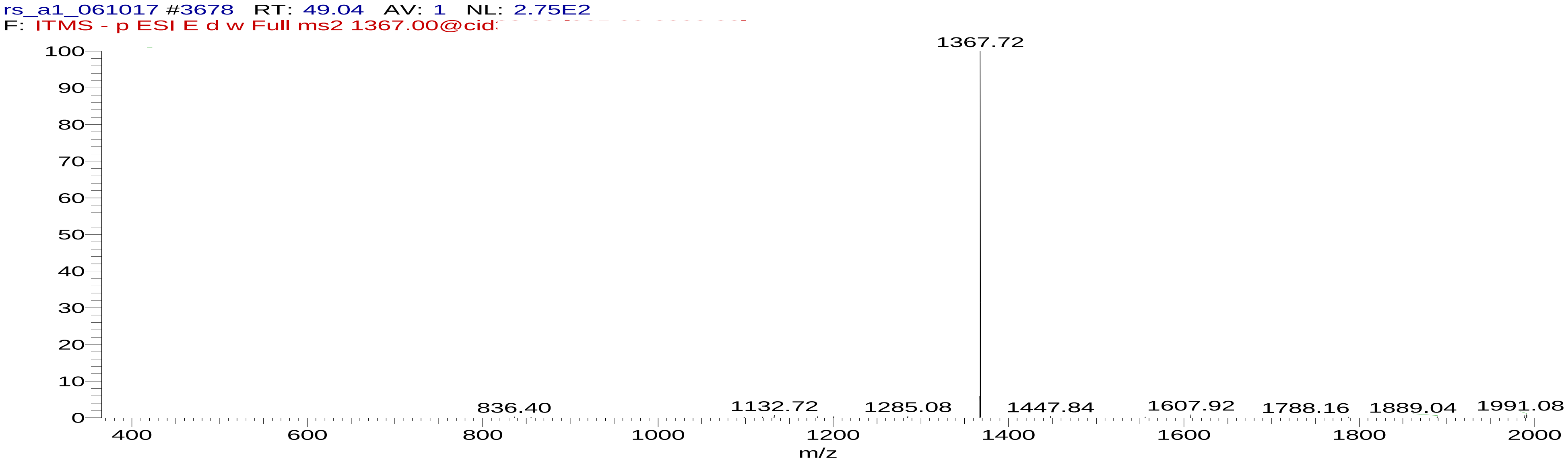

69h

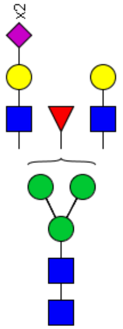

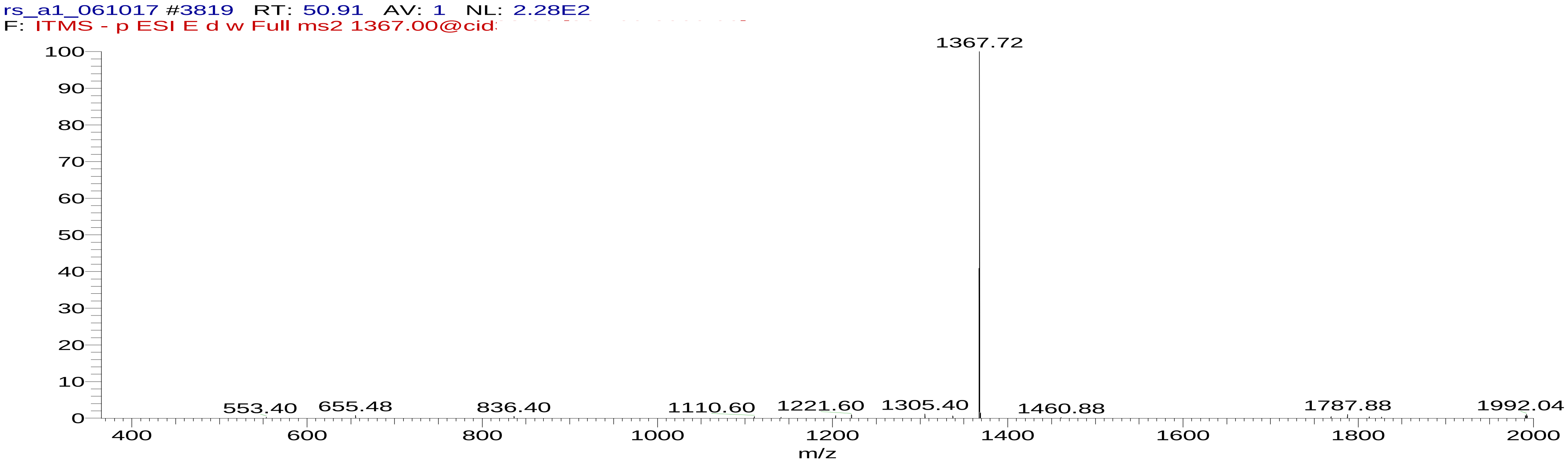

GlytouCan ID: G04256WF

CID-MS/MS
(m/z 1367.52 $^{2-}$)

69i

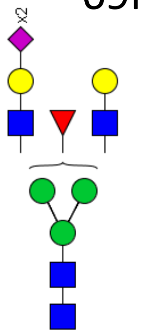

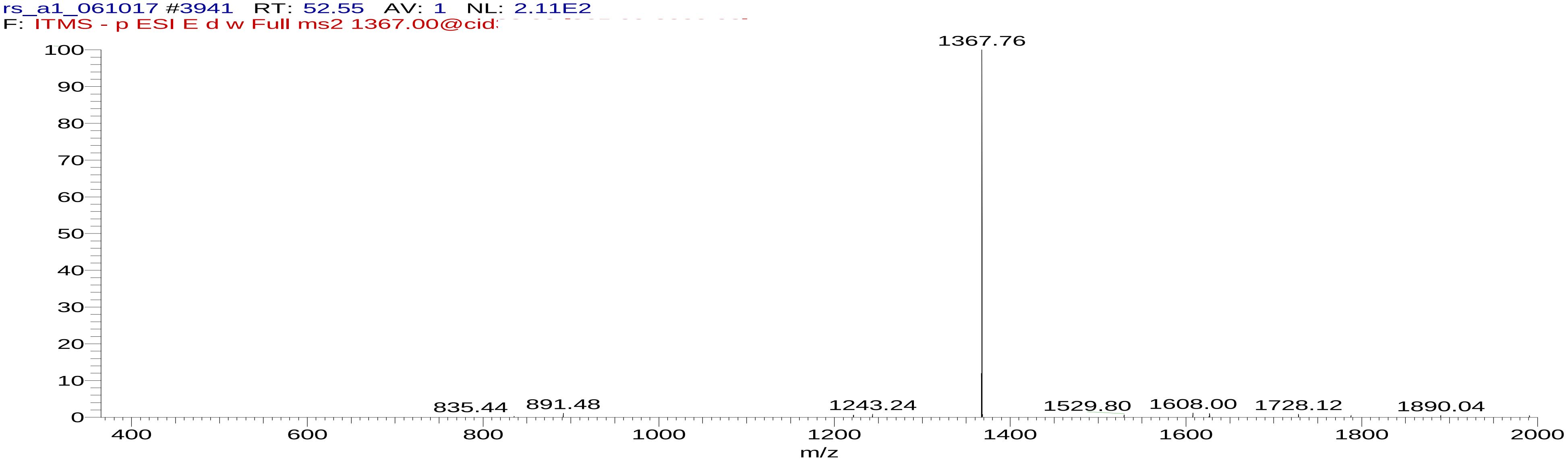

69j

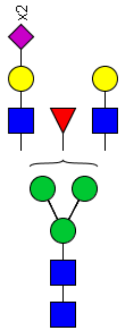

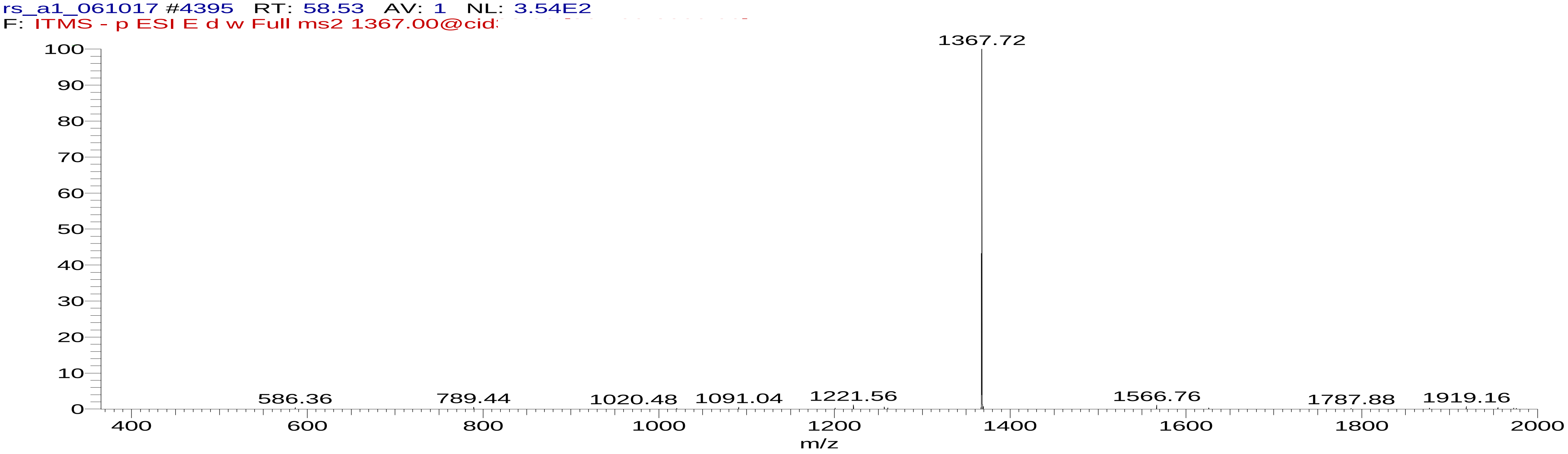

(Hex)4 (HexNAc)4 (Deoxyhexose)1 (NeuAc)1 + (Man)3(GlcNAc)2

GlytouCan ID: G30676OW

[M-2H] 1404.03

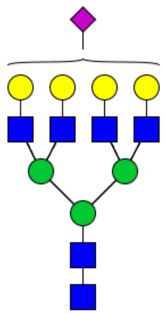

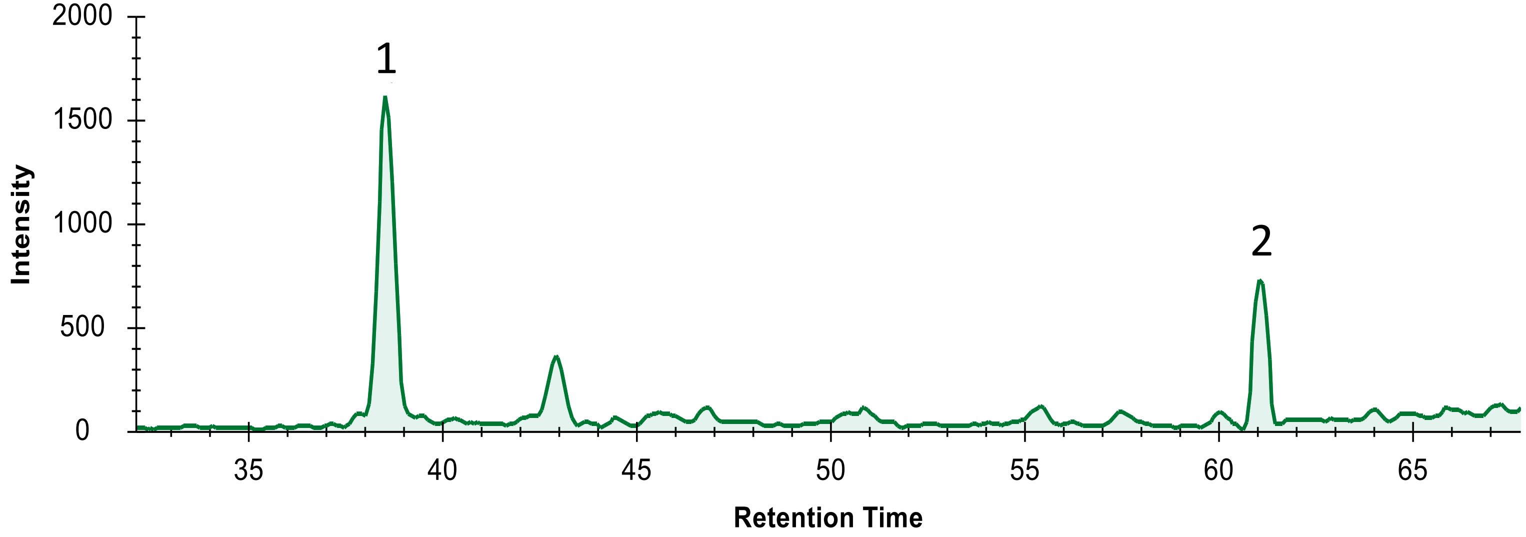

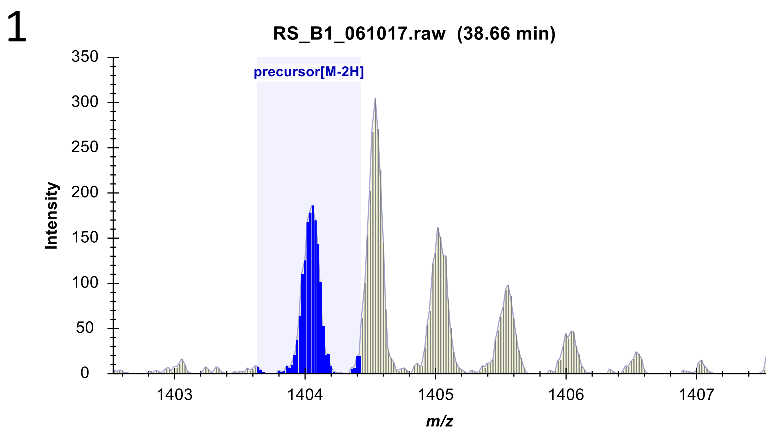

70a

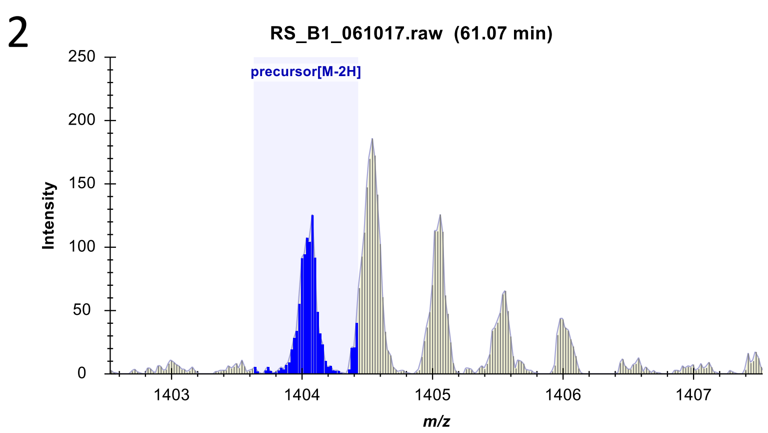

70b

GlytouCan ID: G30676OW

CID-MS/MS
(*m/z* 1404.05 $^{2-}$)

70a

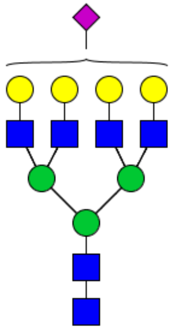

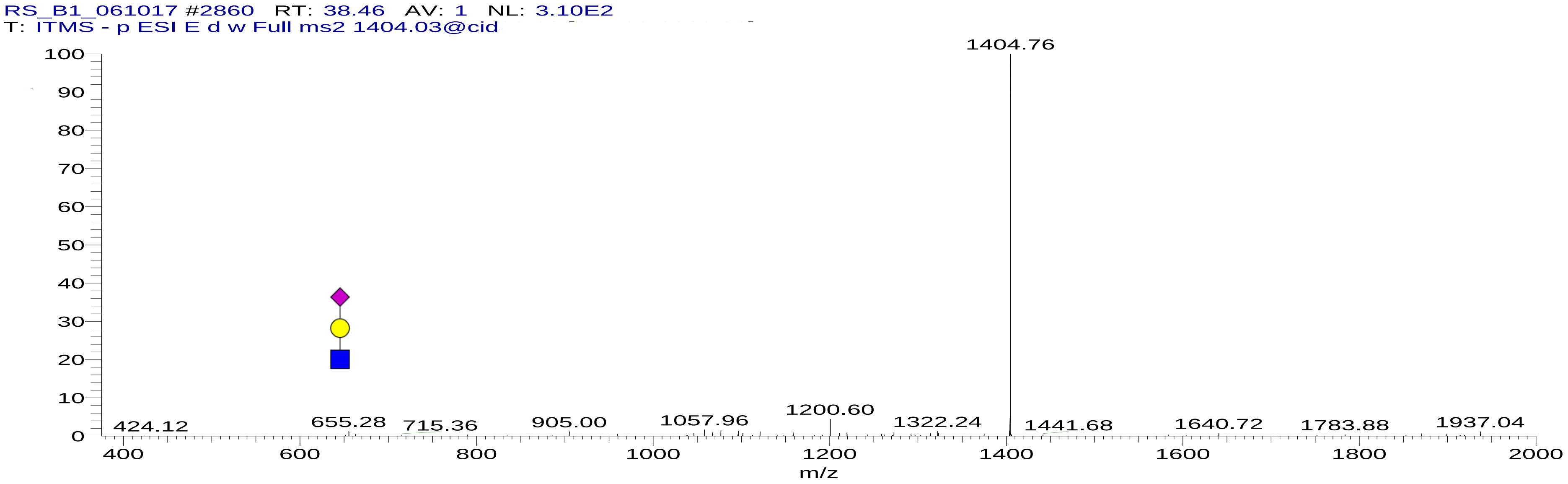

70b

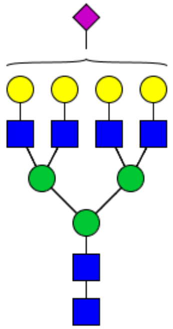

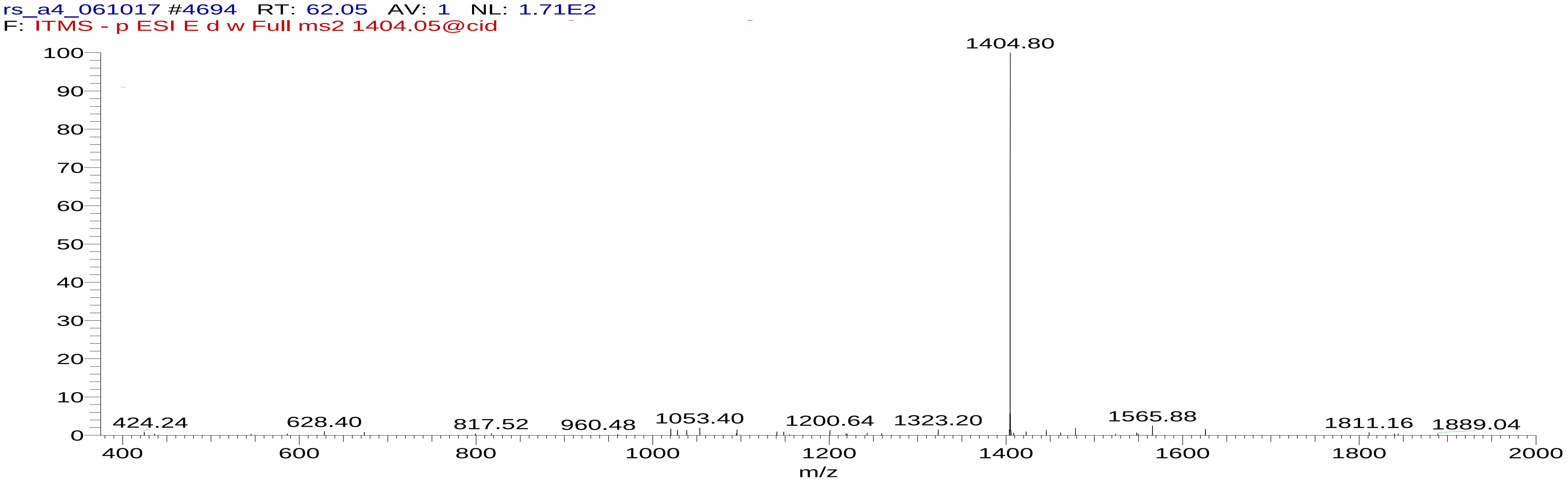

(Hex)3 (HexNAc)3 (NeuAc)3 + (Man)3(GlcNAc)2

[M-2H] 1439.55

[M-3H] 959.33

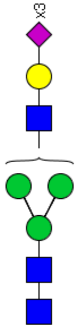

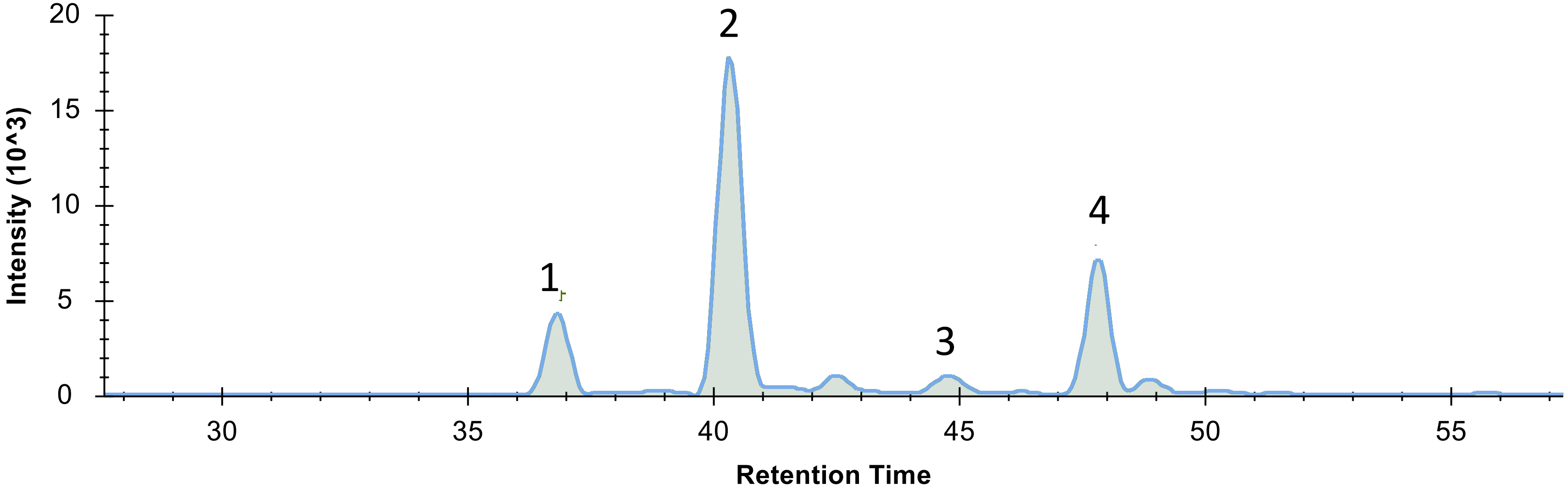

1

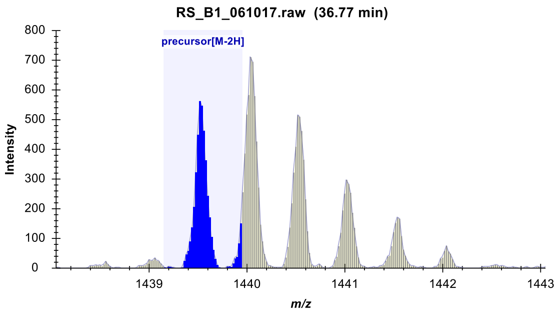

71a

2

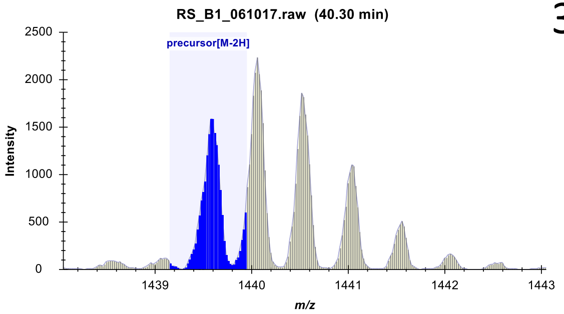

71b

3

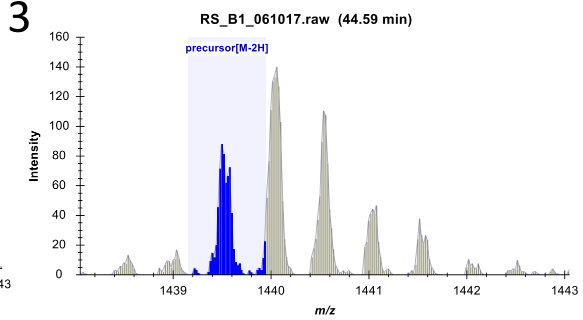

71c

4

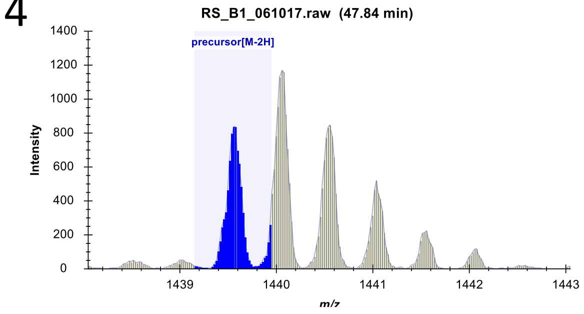

71d

CID-MS/MS

(m/z 959.66 $^{3-}$)

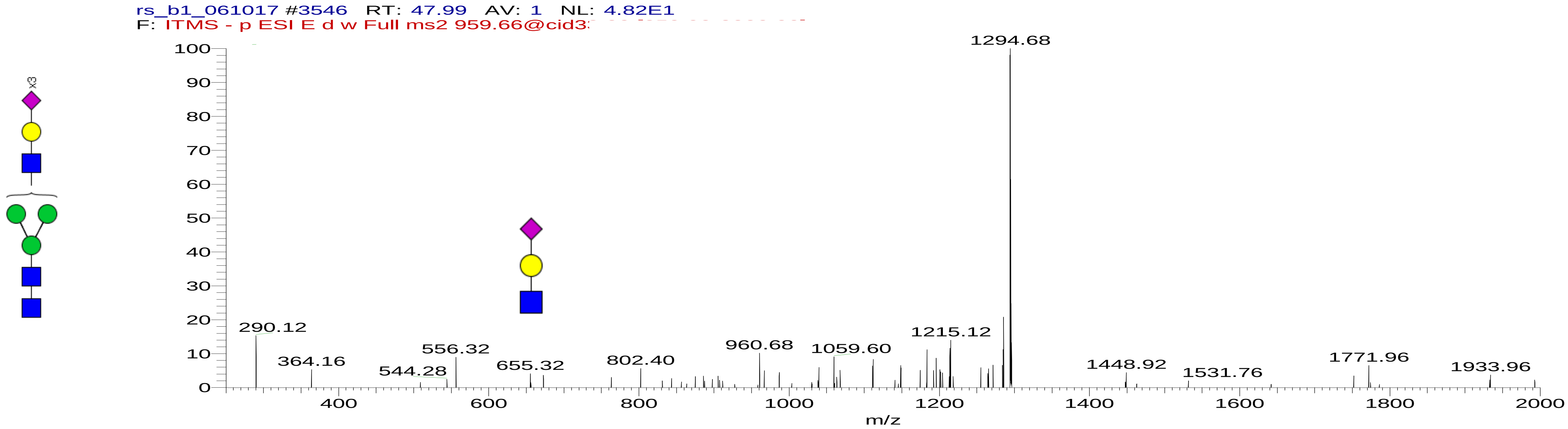

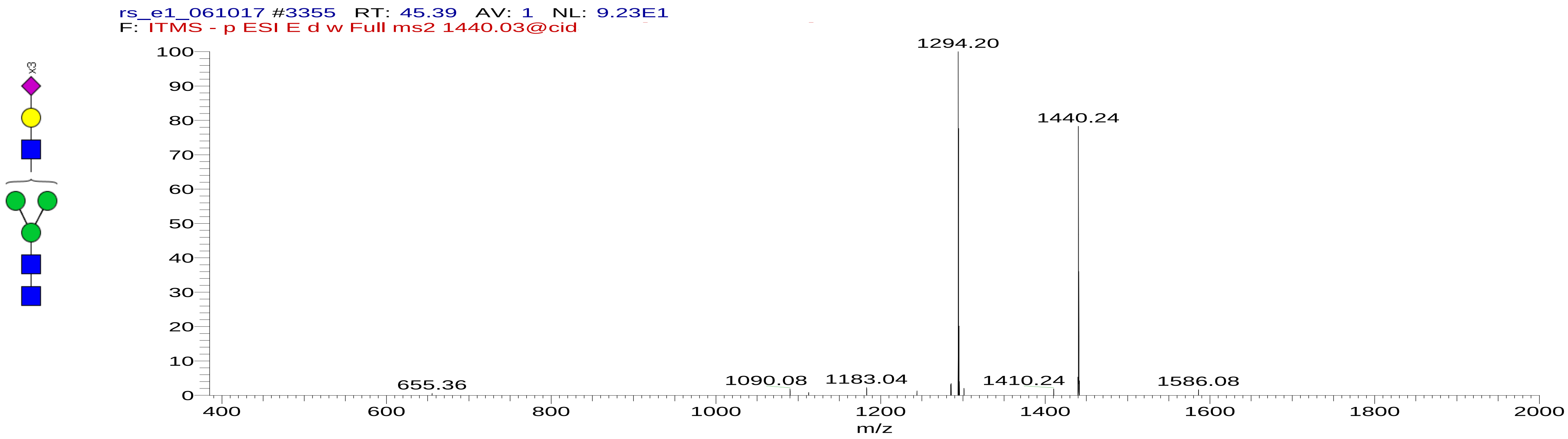

CID-MS/MS
(*m/z* 959.66 $^{3-}$)

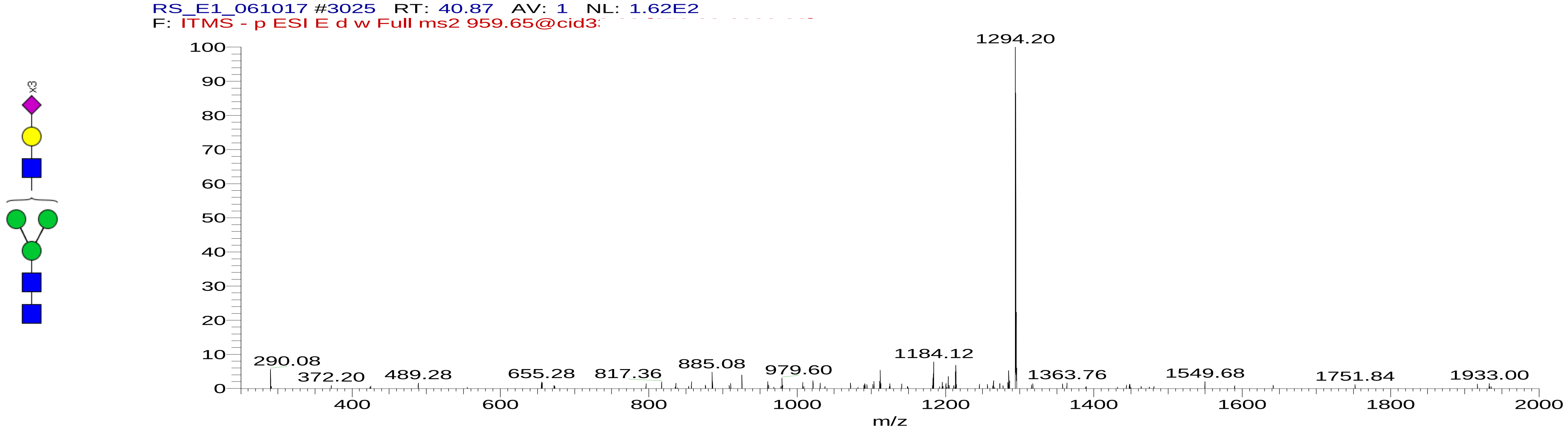

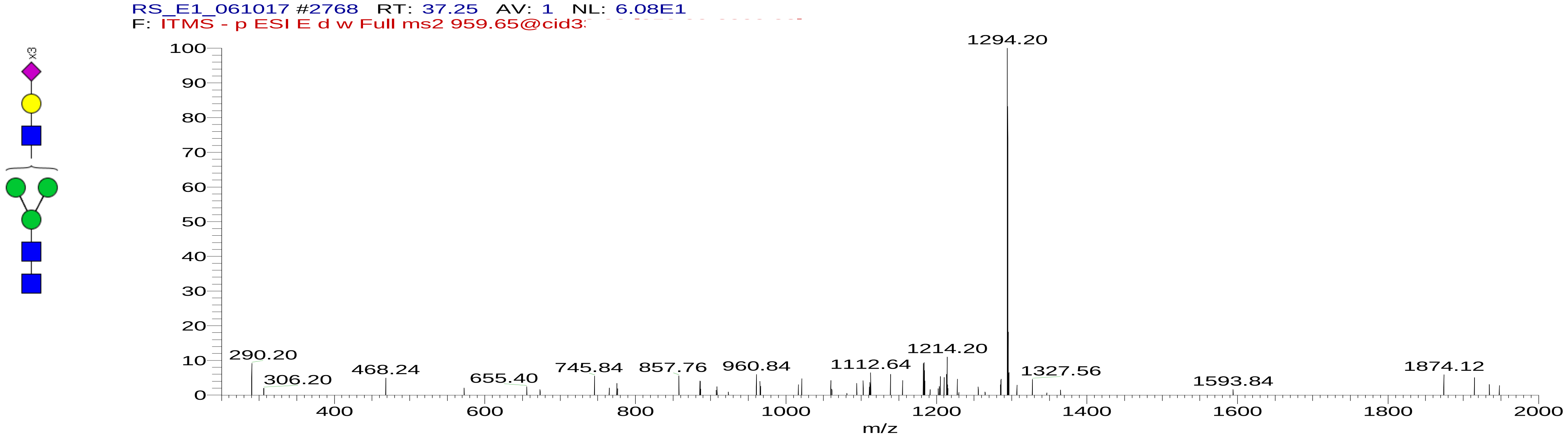

(Hex)4 (HexNAc)4 (NeuAc)2 + (Man)3(GlcNAc)2

No GlytouCan ID

[M-2H] 1476.57

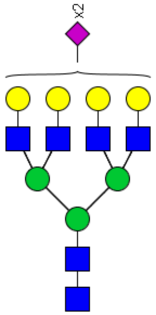

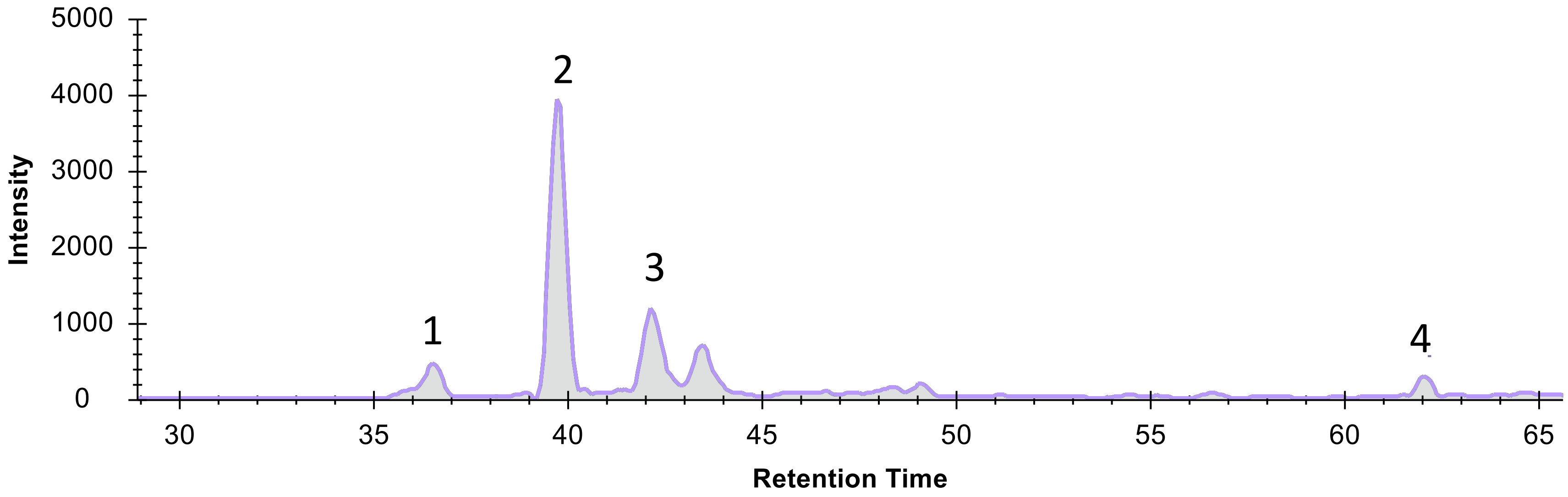

1 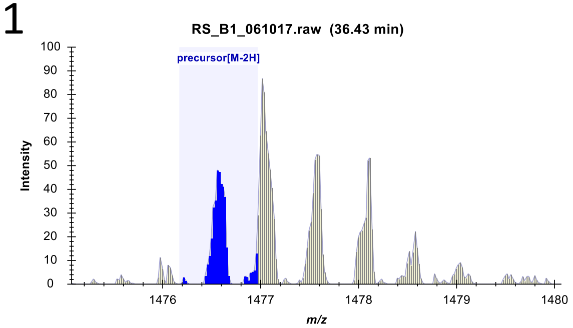

72a

2 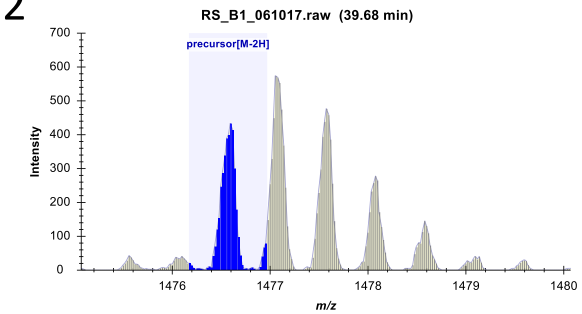

72b

3 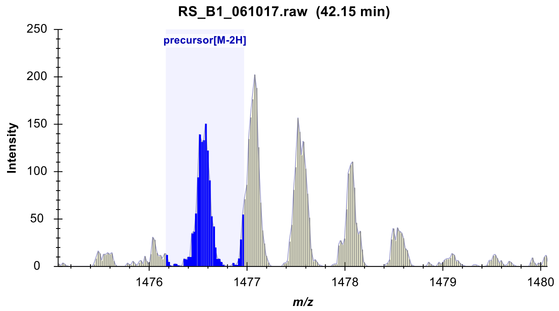

72c

4 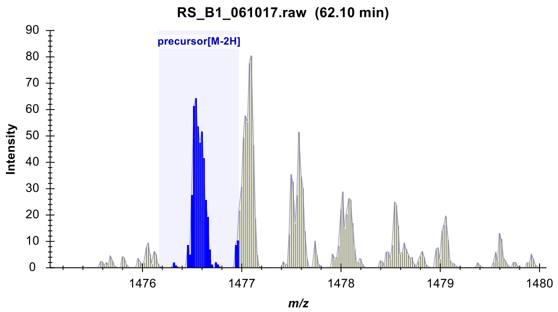

72d

CID-MS/MS
(m/z 1476.57 $^{2-}$)

72a

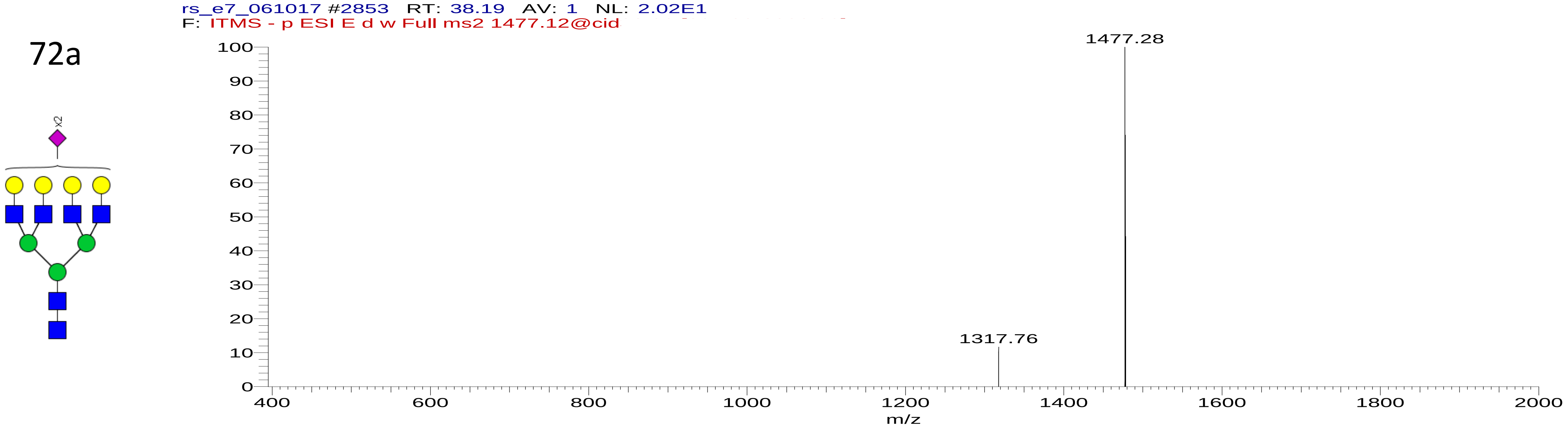

72b

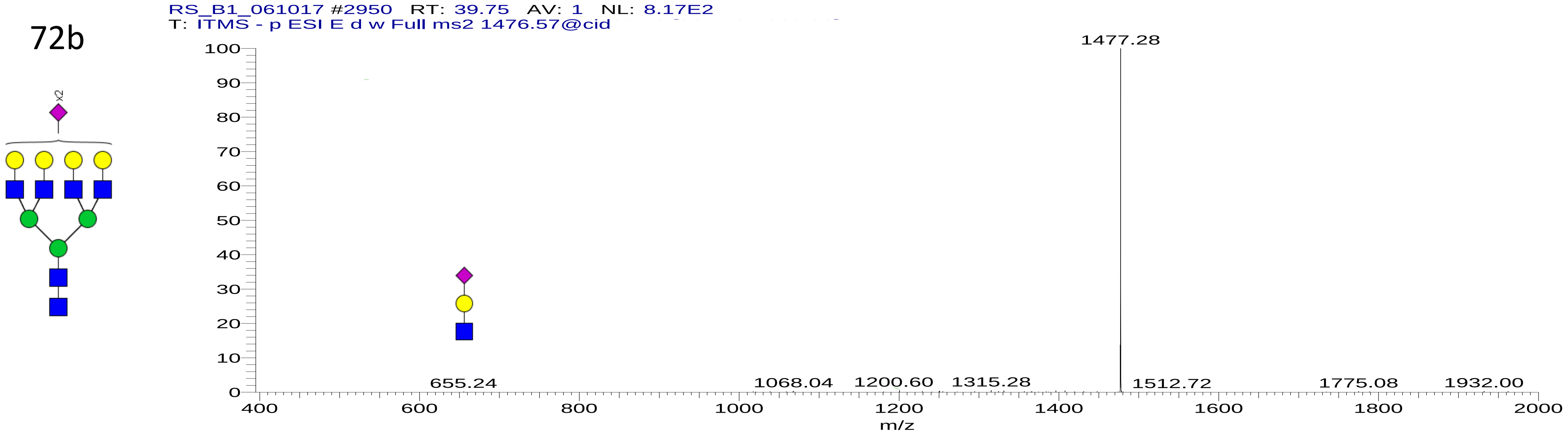

CID-MS/MS
(m/z 1476.57 $^{2-}$)

72c

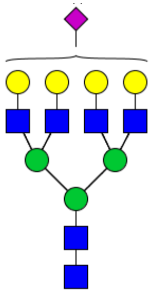

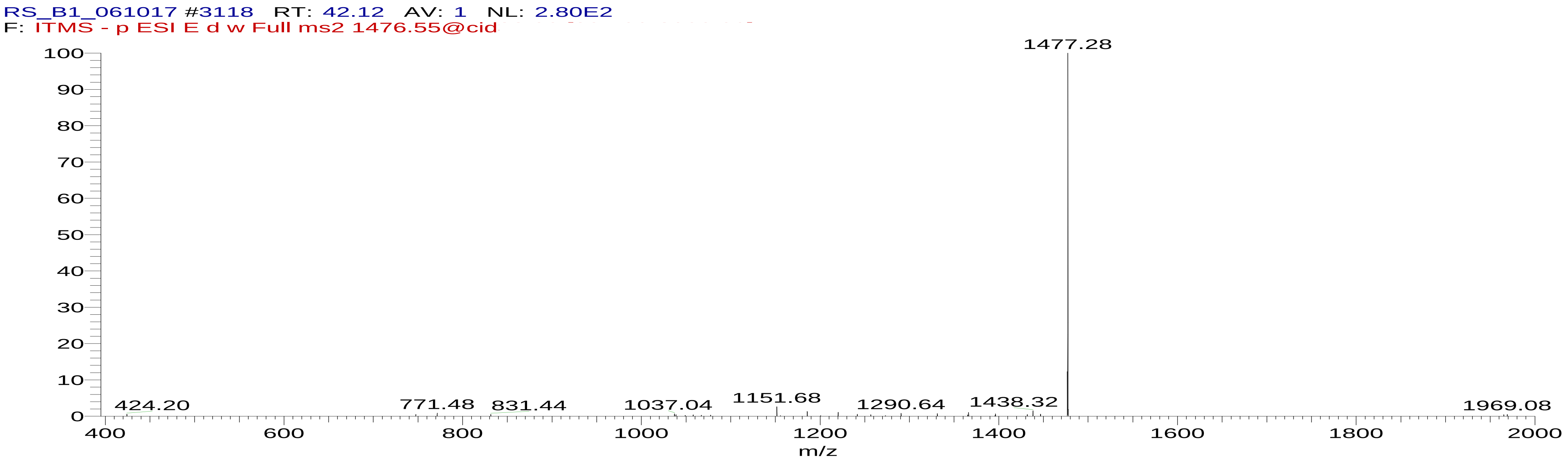

72d

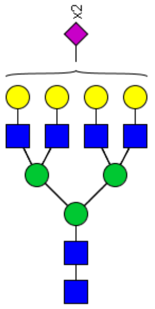

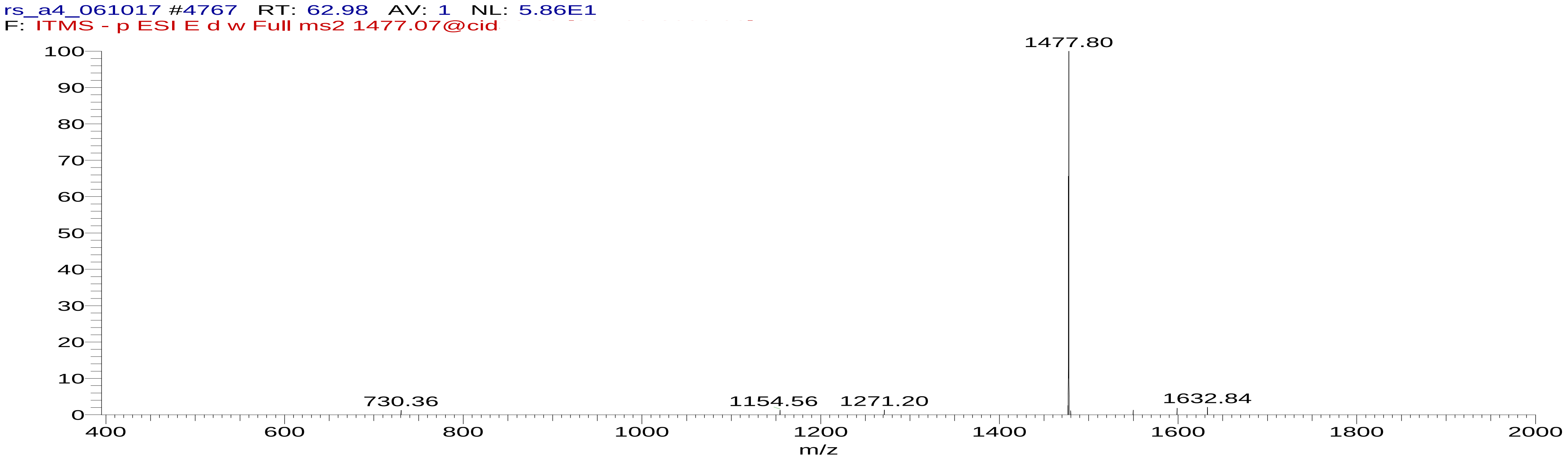

(Hex)3 (HexNAc)3 (Deoxyhexose)1 (NeuAc)3 + (Man)3(GlcNAc)2
GlytouCan ID: G72165OE
[M-2H] 1512.59

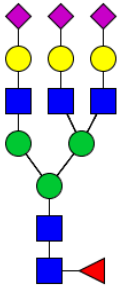

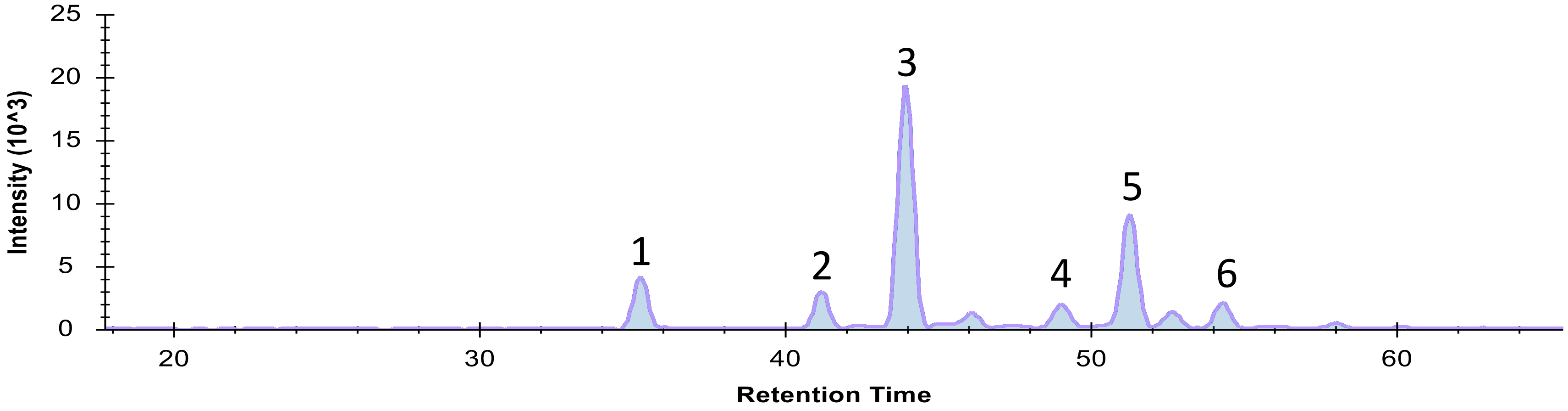

1
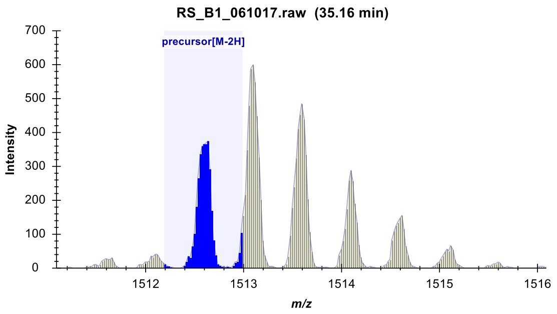

73a

2
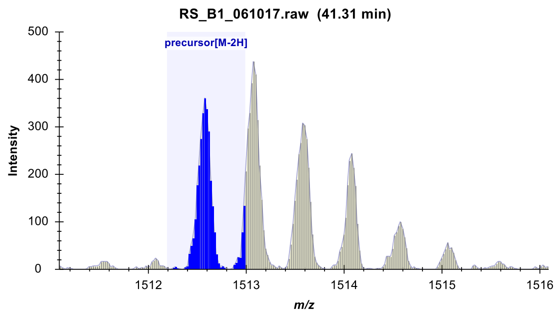

73b

3
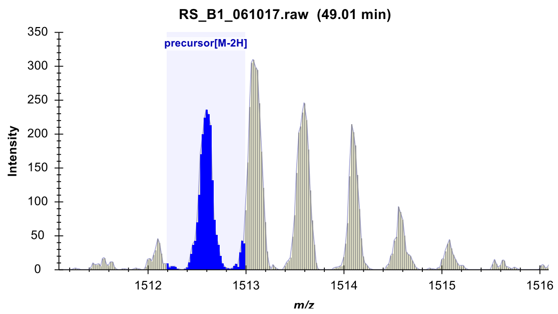

73c

4
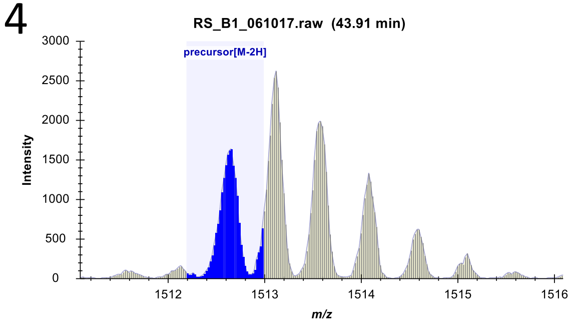

73d

5
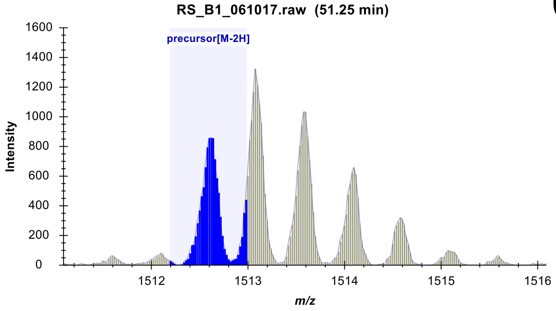

73e

6
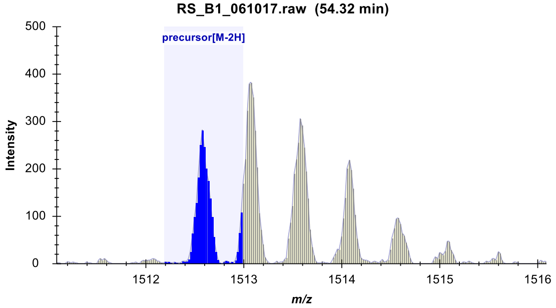

73f

GlytouCan ID: G72165OE

CID-MS/MS
(*m/z* 1008.33 $^{3-}$)

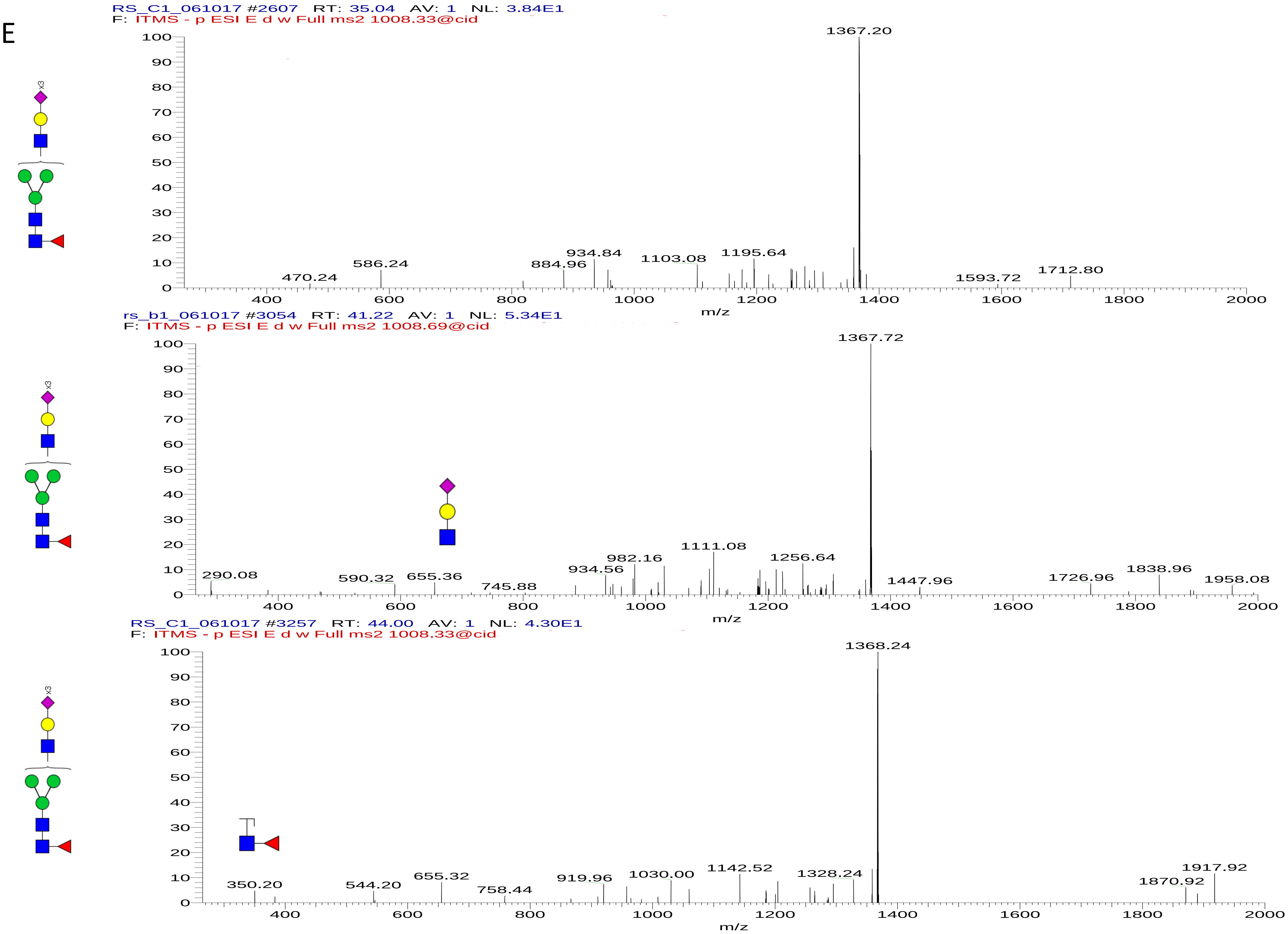

GlytouCan ID: G72165OE

CID-MS/MS
(*m/z* 1008.33 $^{3-}$)

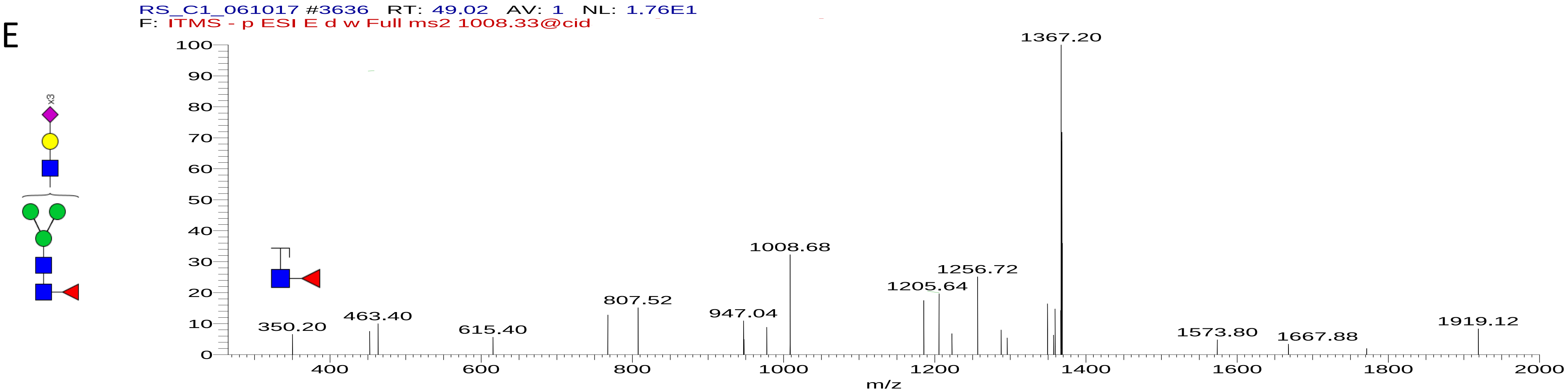

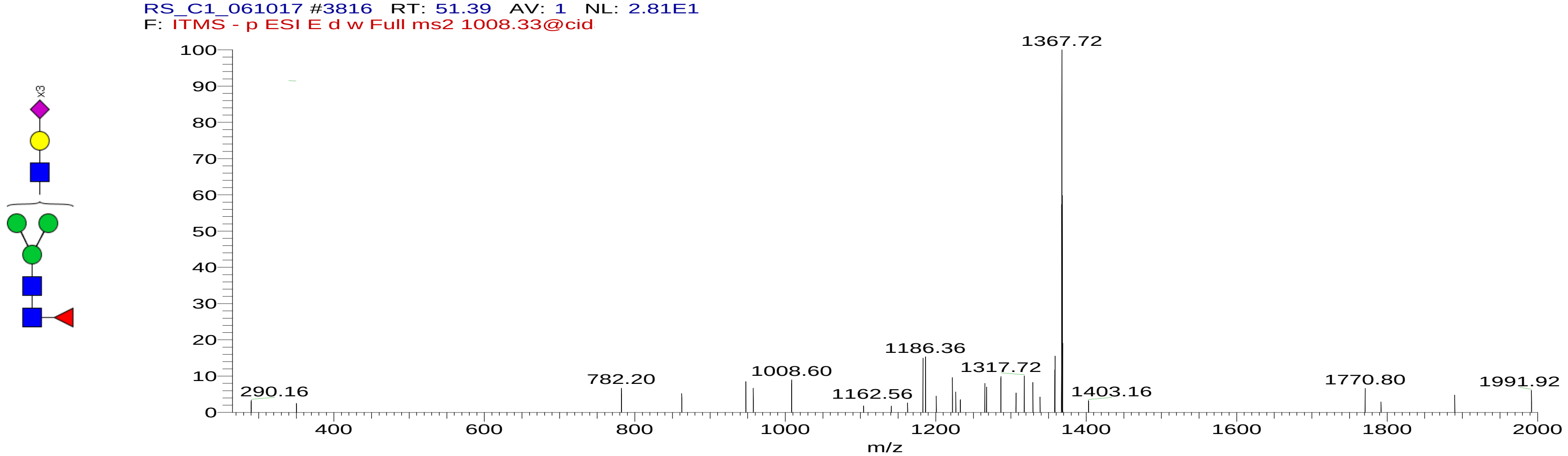

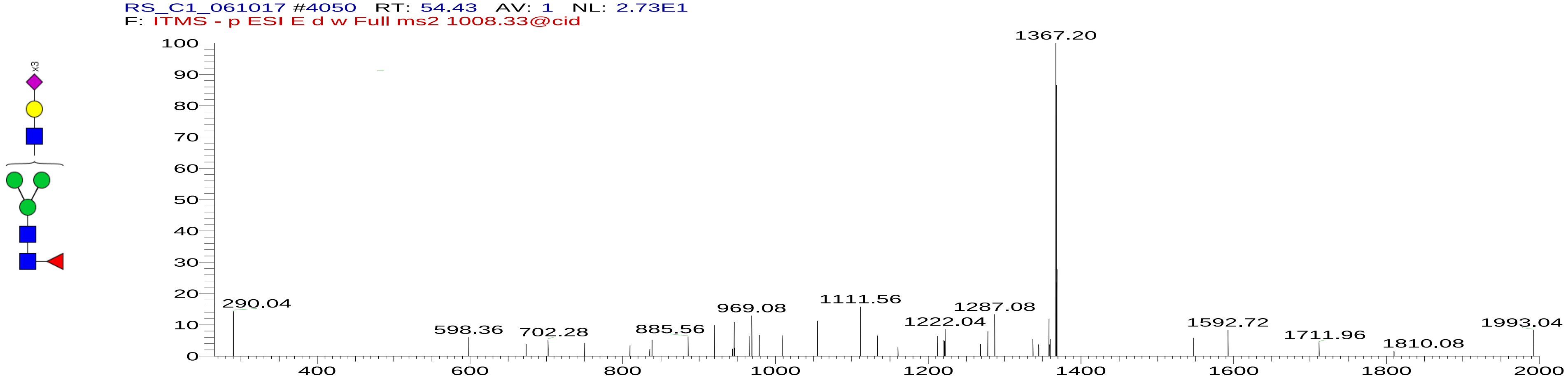

(Hex)4 (HexNAc)4 (Deoxyhexose)1 (NeuAc)2 + (Man)3(GlcNAc)2
GlytouCan ID: G86960BM

[M-2H] 1549.6
[M-3H] 1032.69

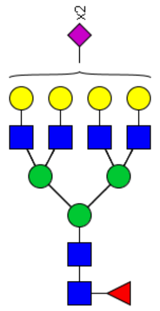

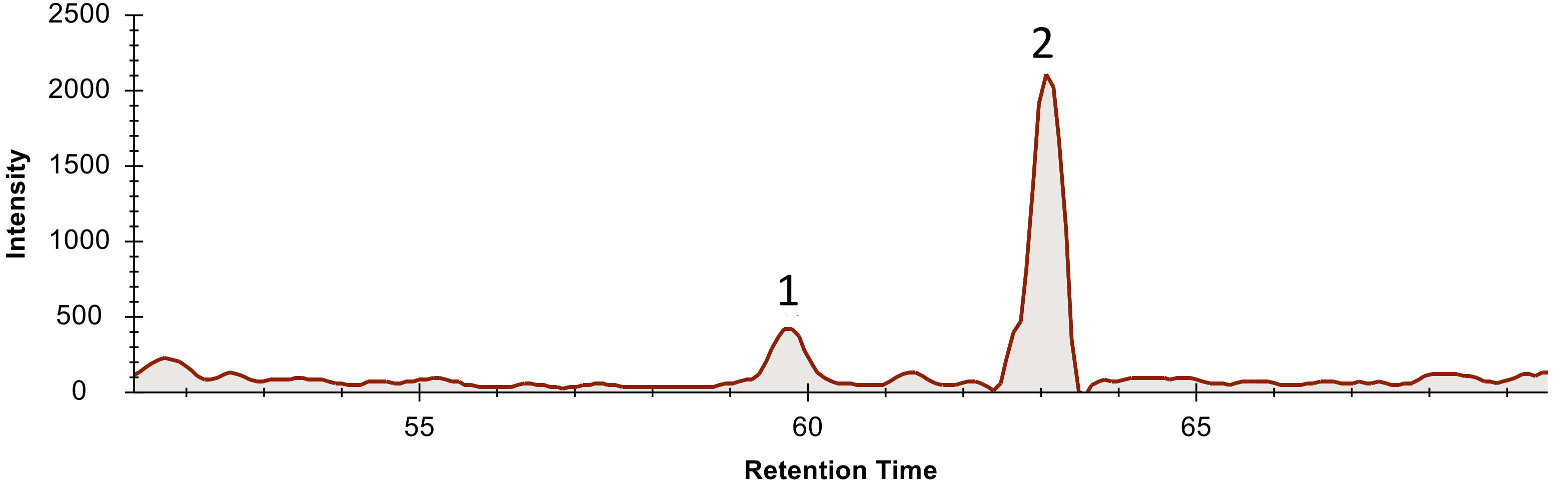

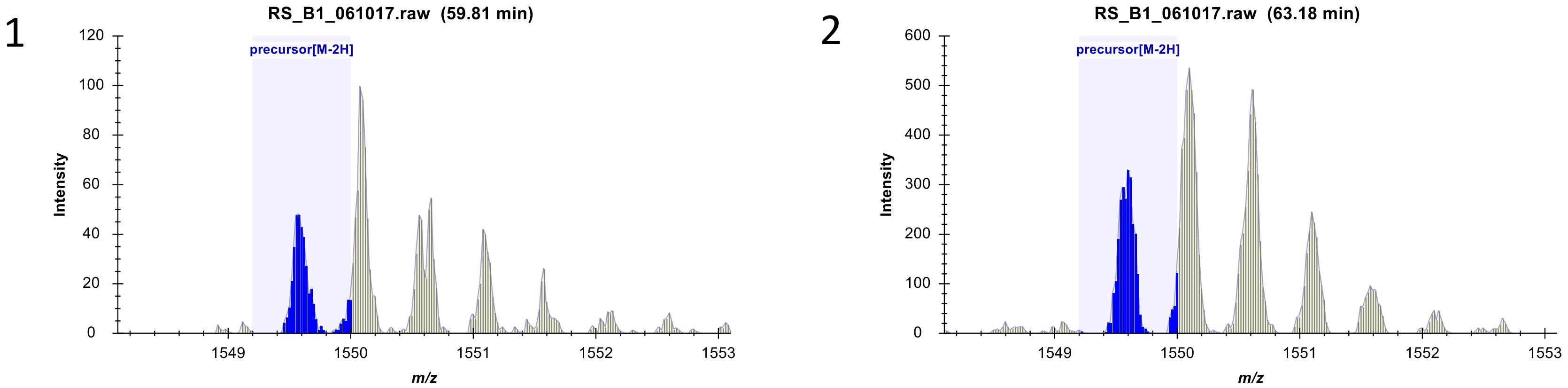

74a

74b

GlytouCan ID: G86960BM

CID-MS/MS
(m/z 1032.46 $^{3-}$)

74a

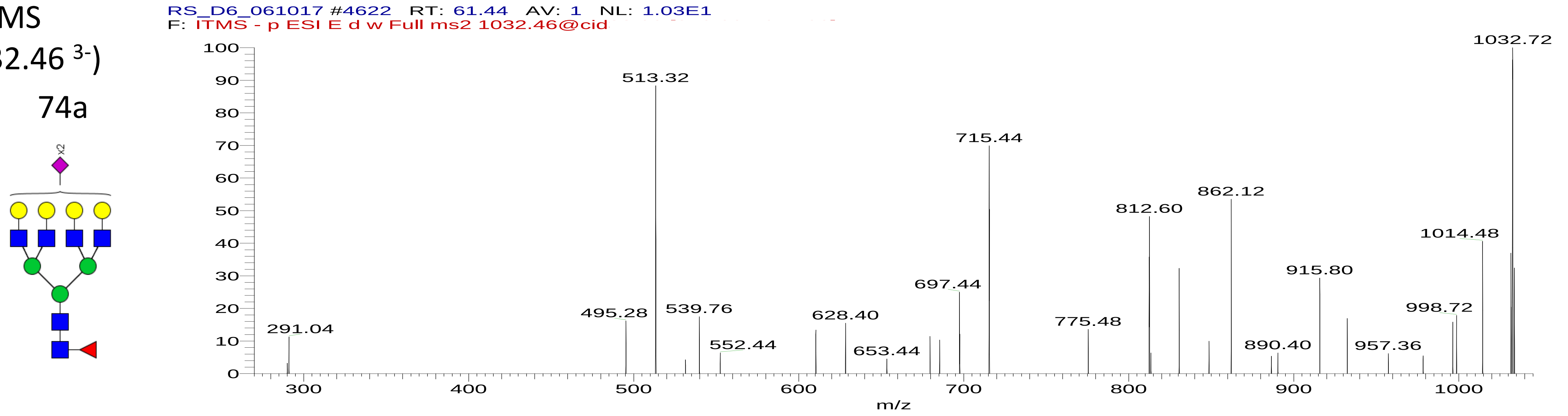

74b

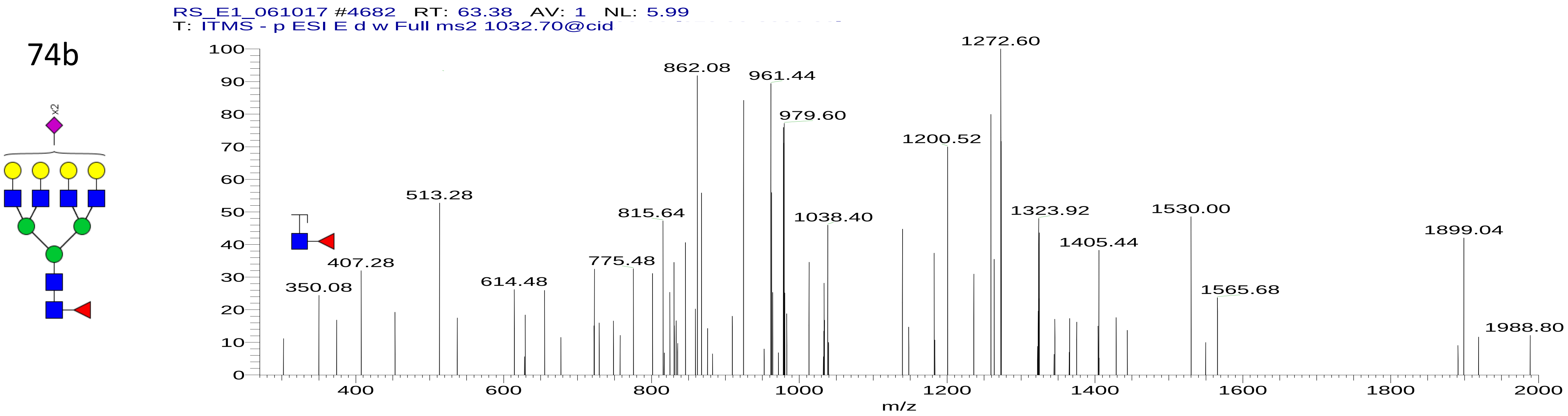

(Hex)5 (HexNAc)5 (Deoxyhexose)1 (NeuAc)1 + (Man)3(GlcNAc)2

No GlytouCan ID

[M-2H] 1586.61

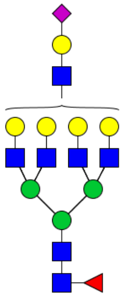

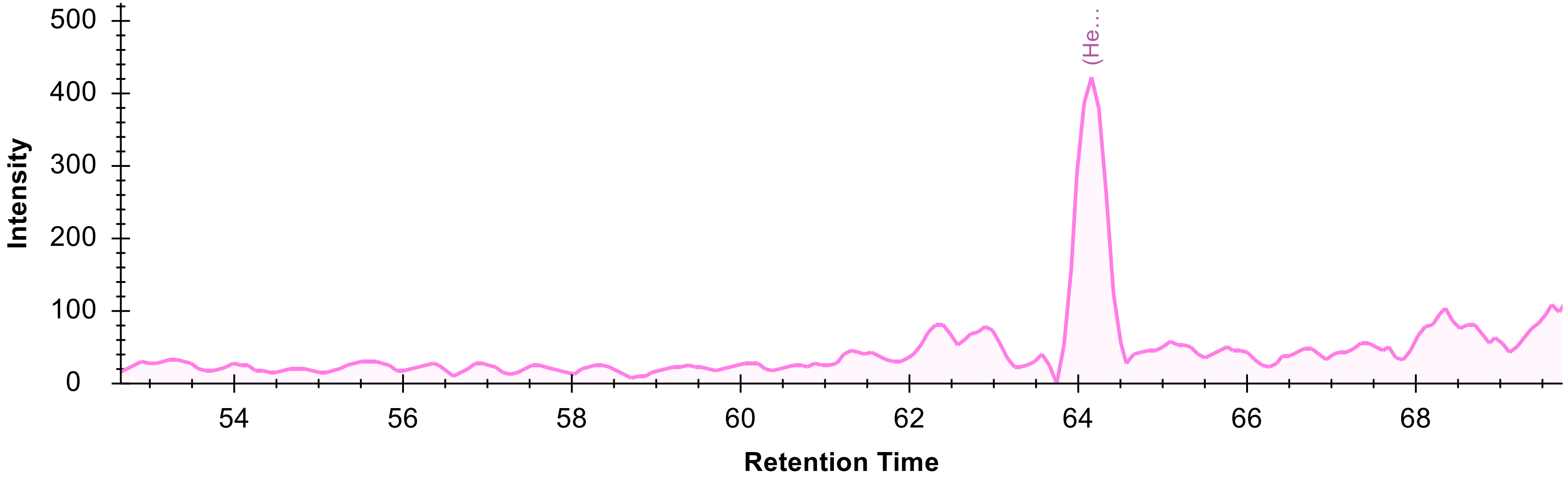

CID-MS/MS (m/z 1586.61 $^{2-}$)

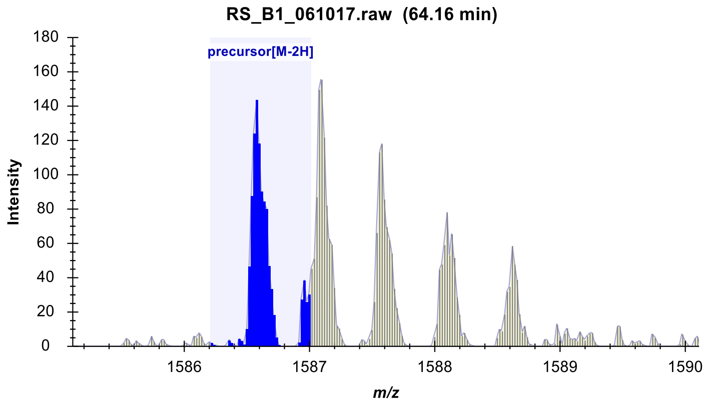

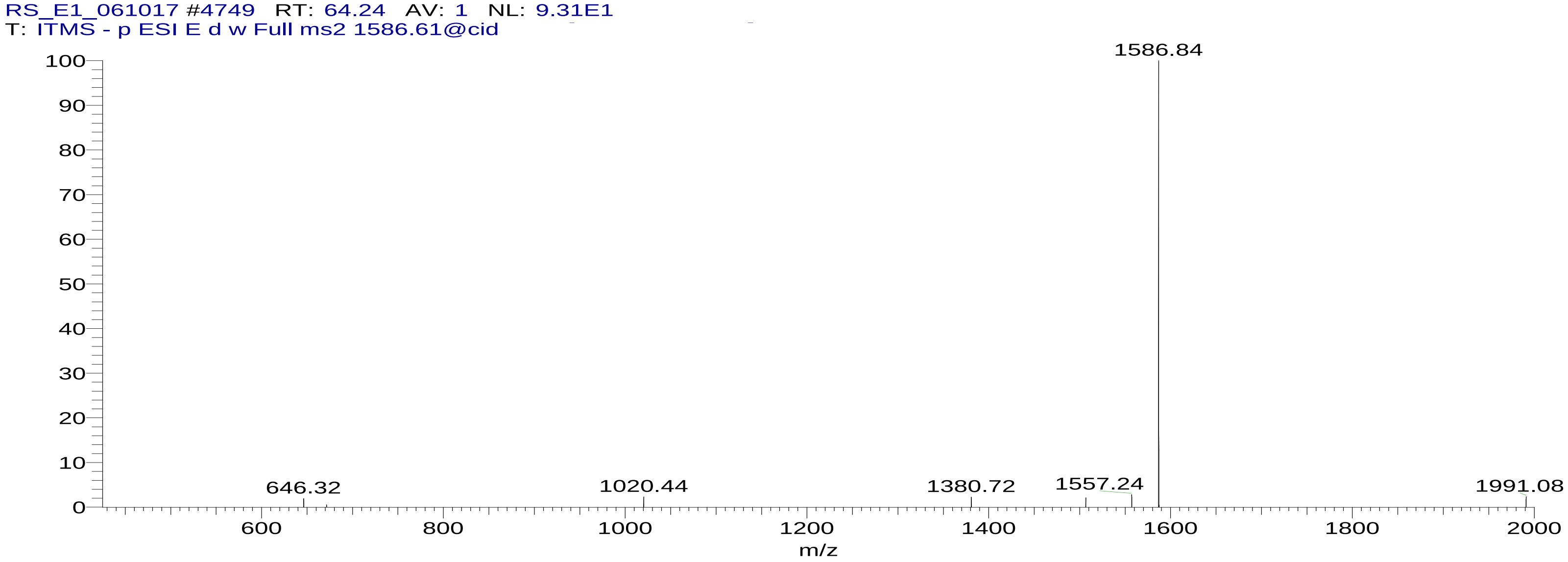

Glycan ID: 75

(Hex)4 (HexNAc)4 (NeuAc)3 + (Man)3(GlcNAc)2

[M-2H] 1622.14

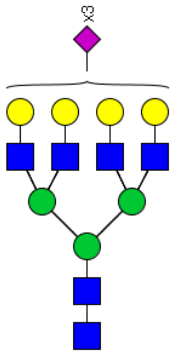

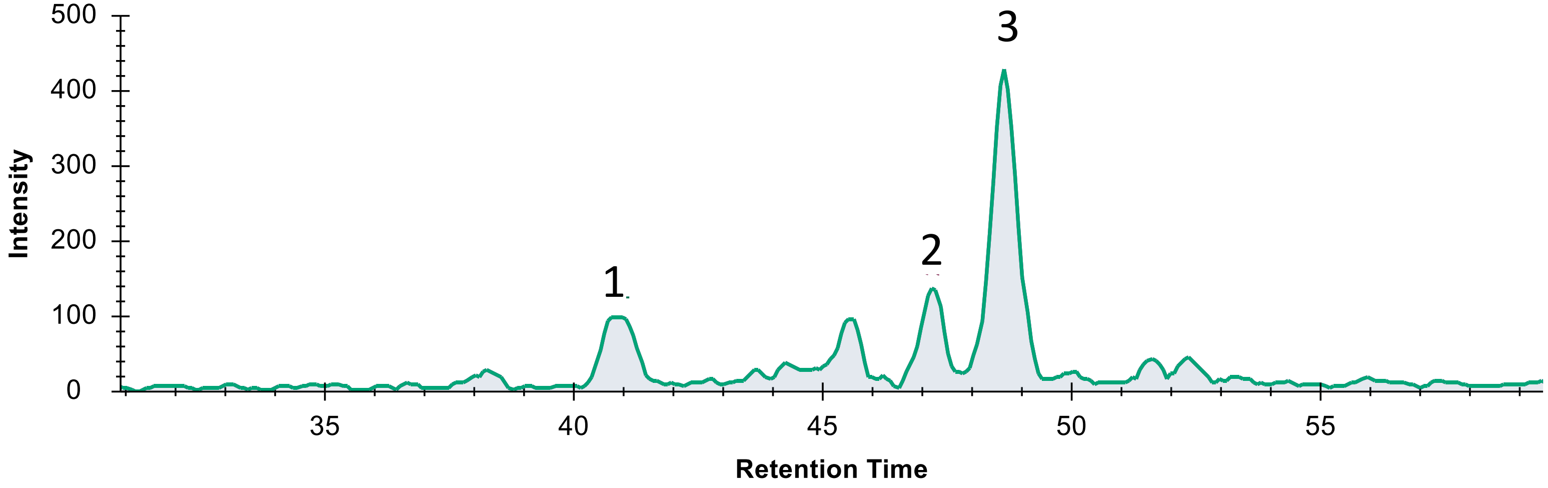

1

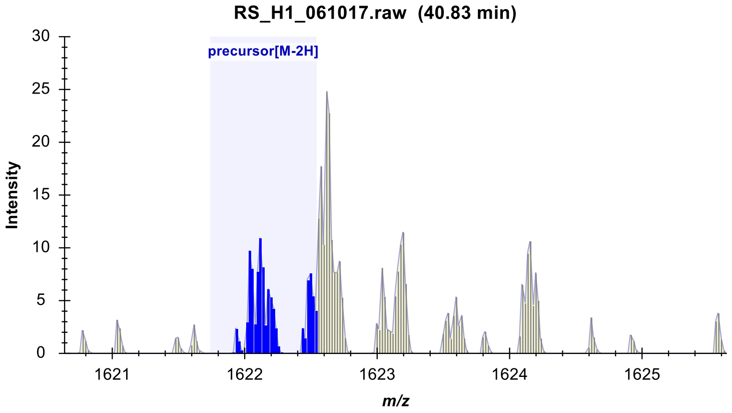

76a

2

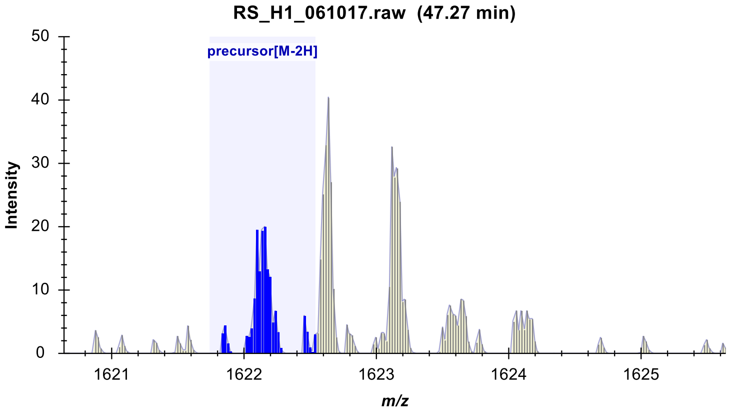

76b

3

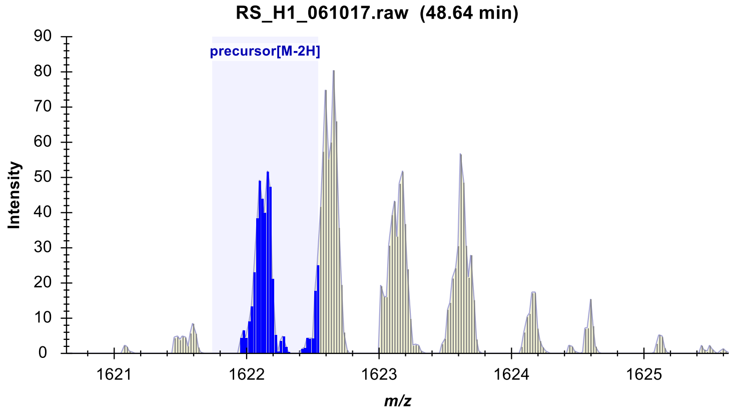

76c

CID-MS/MS

(m/z 1081.38 $^{3-}$)

76a

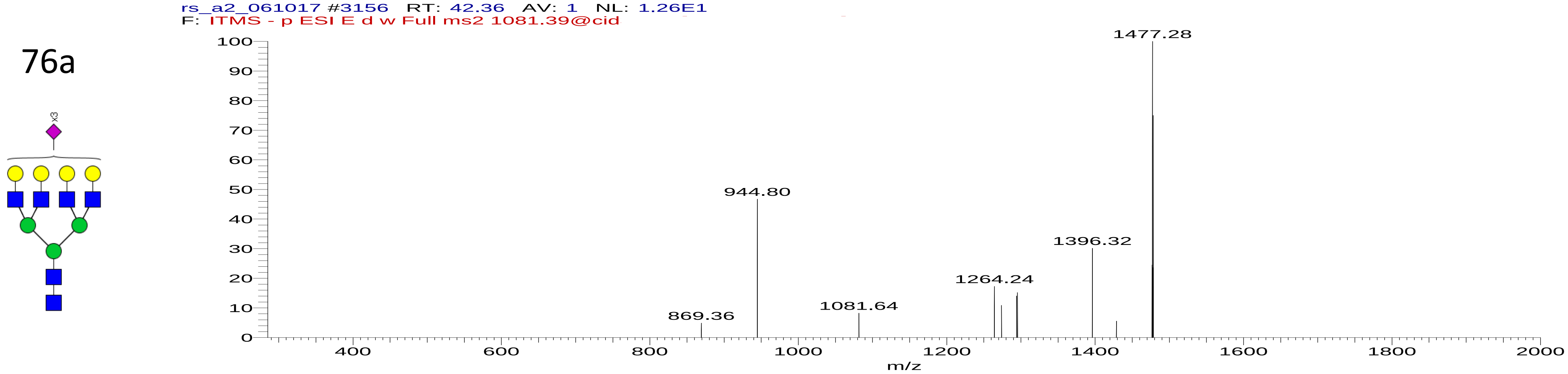

76b

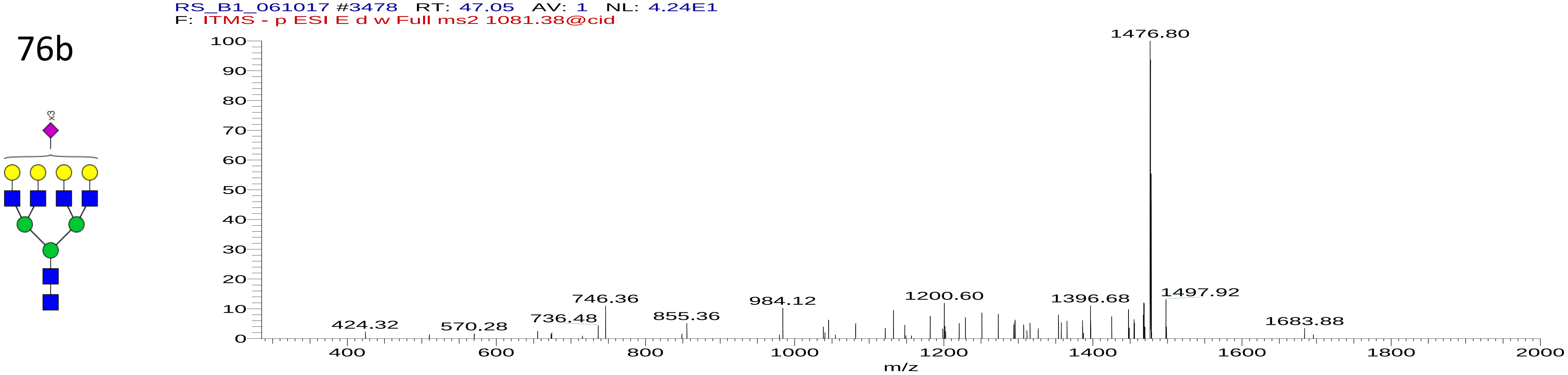

76c

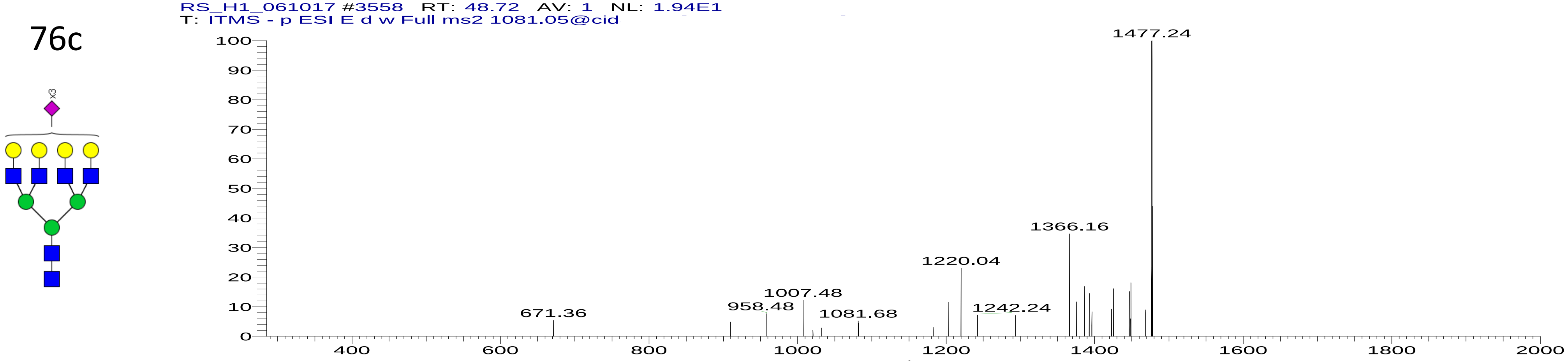

(Hex)4 (HexNAc)4 (Deoxyhexose)1 (NeuAc)3 + (Man)3(GlcNAc)2

No GlytouCan ID

[M-2H] 1695.18

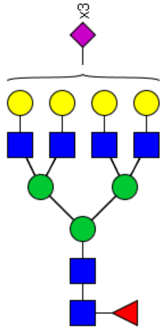

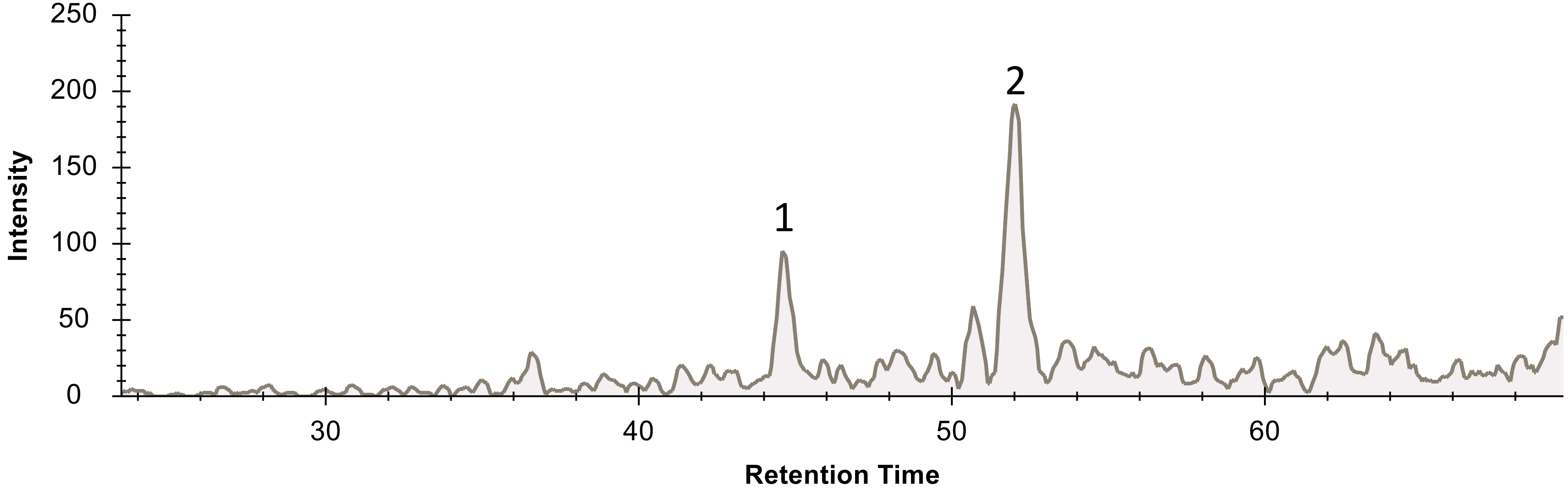

1

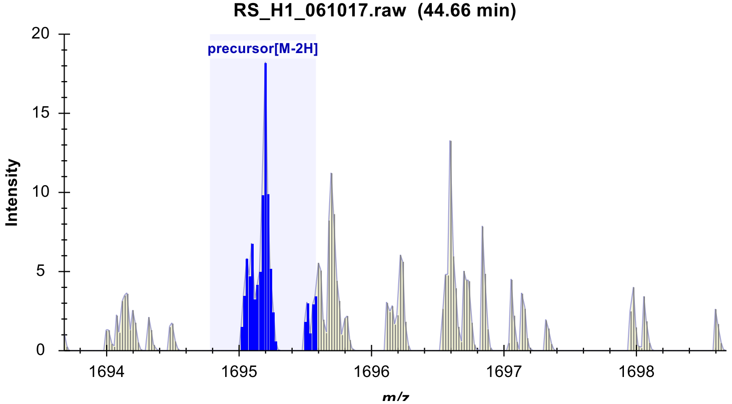

77a

2

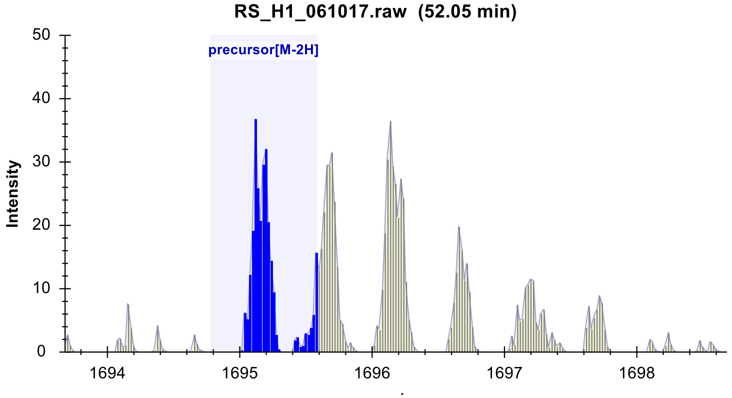

77b

CID-MS/MS

(m/z 1130 $^{3-}$)

77a

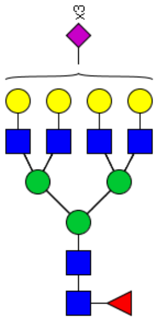

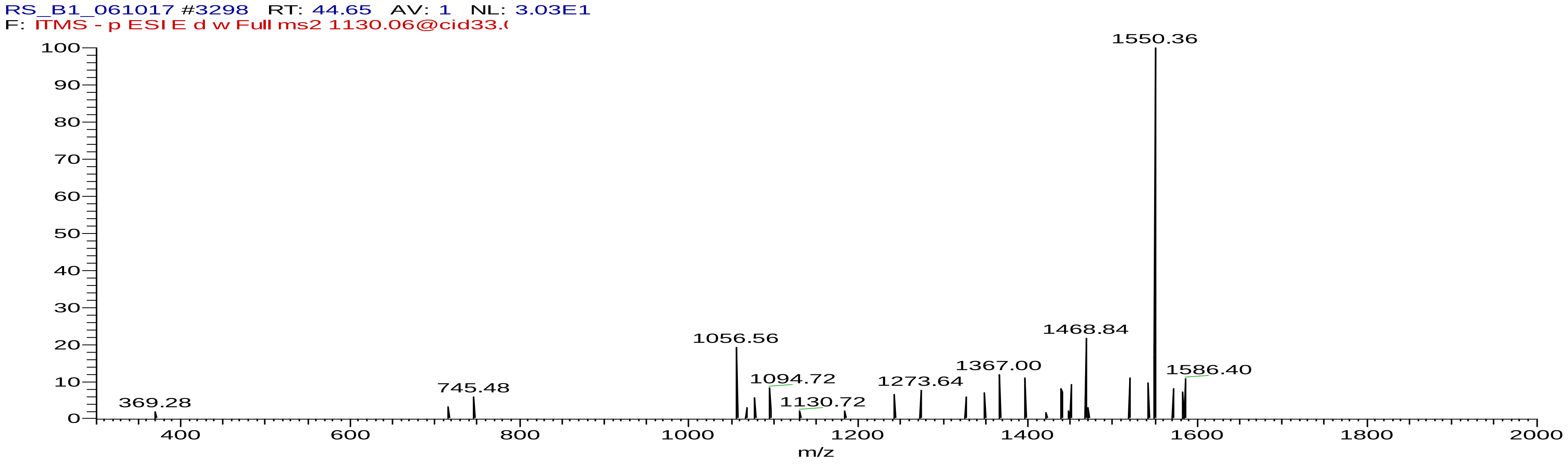

77b

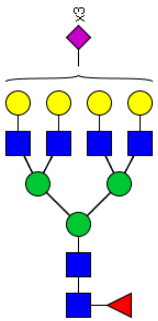

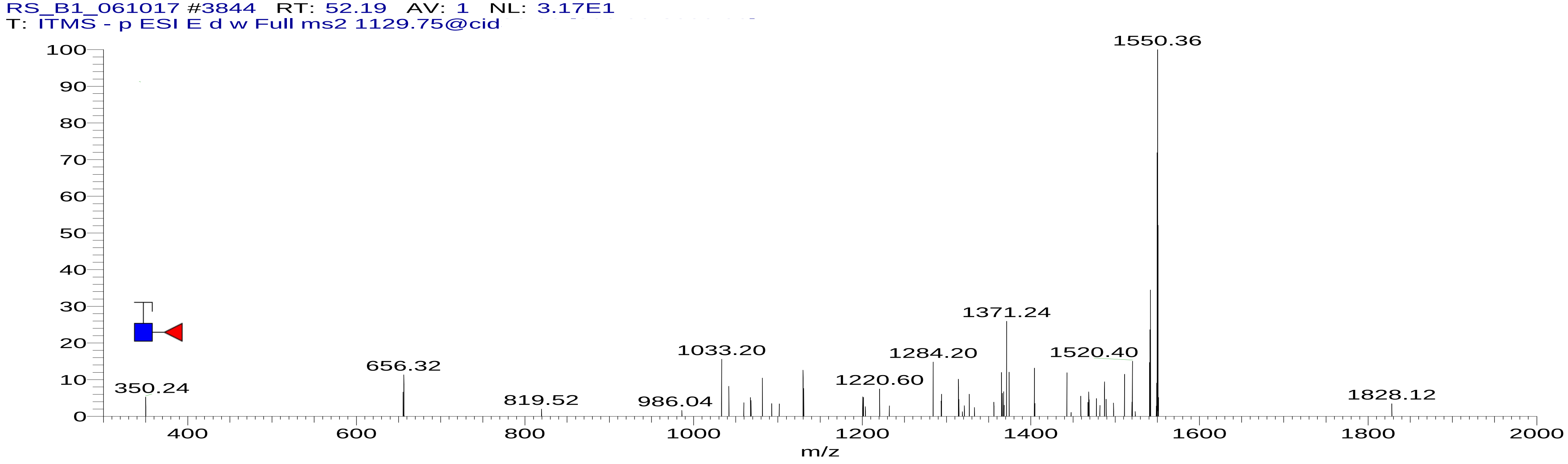

(Hex)5 (HexNAc)5 (Deoxyhexose)1 (NeuAc)2 + (Man)3(GlcNAc)2

No GlytouCan ID

[M-2H] 1732.18

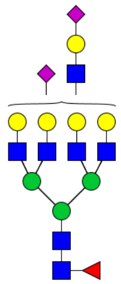

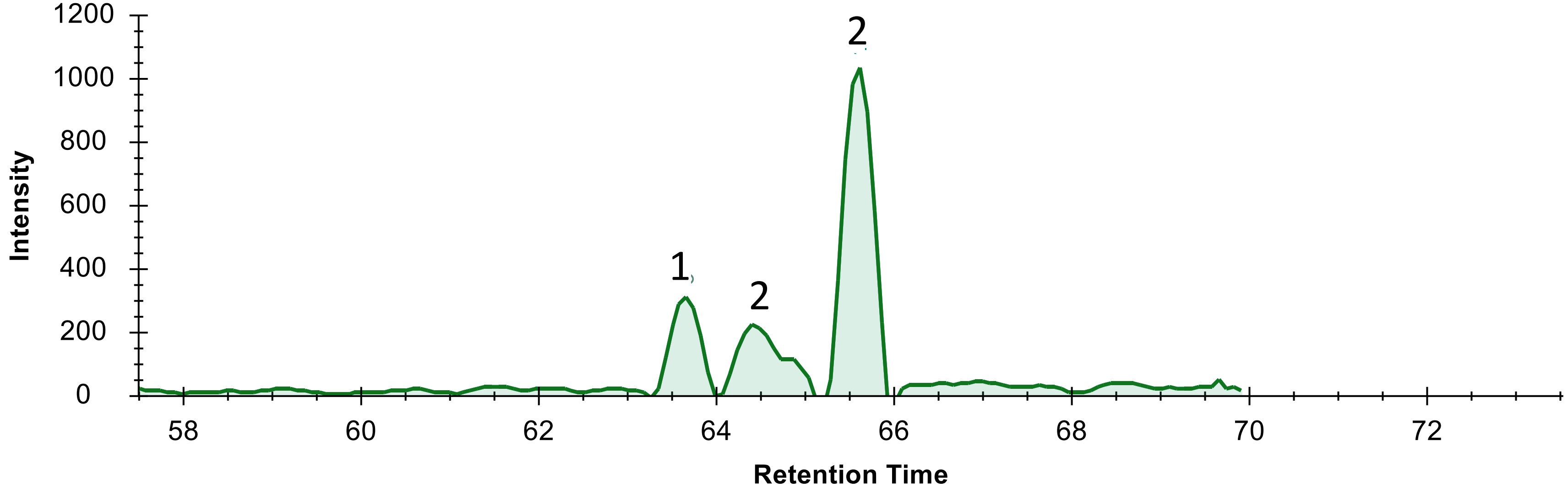

1

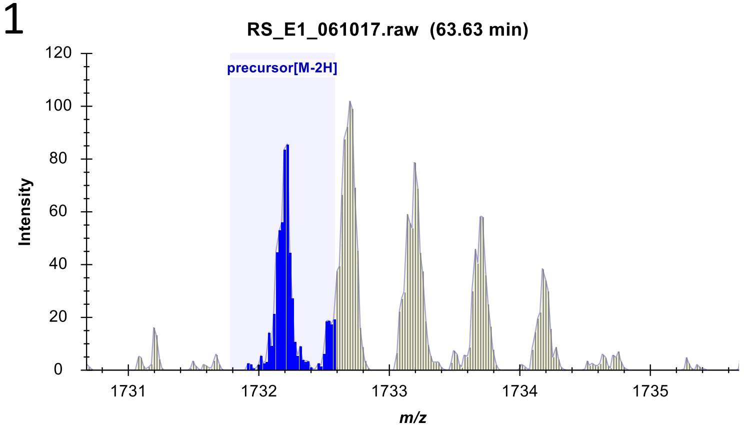

78a

2

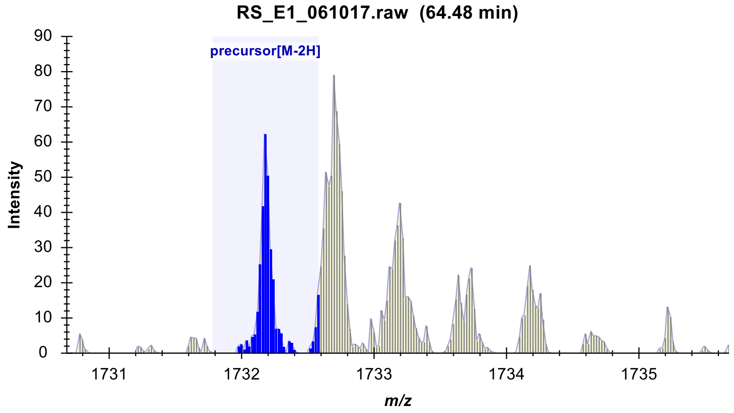

78b

3

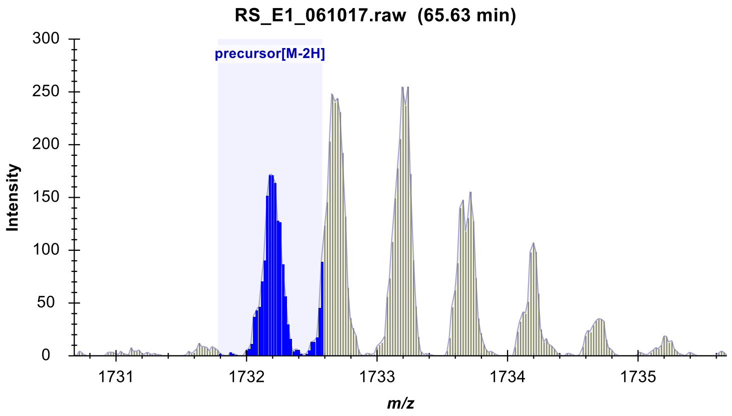

78c

CID-MS/MS
(m/z 1154.78 $^{3-}$)

78a

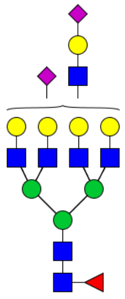

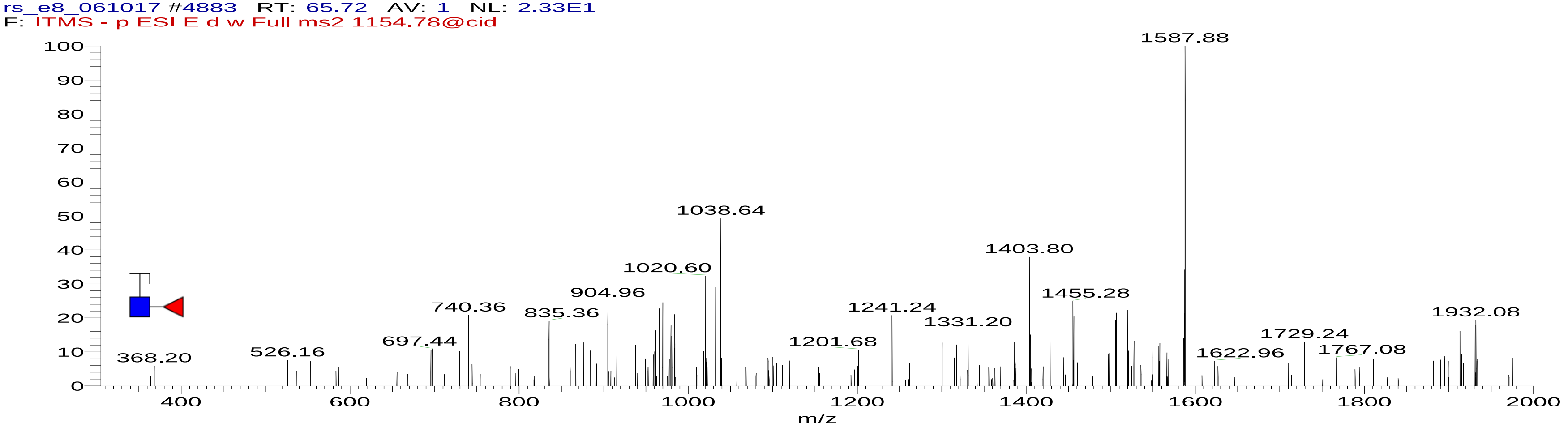

78b

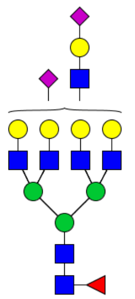

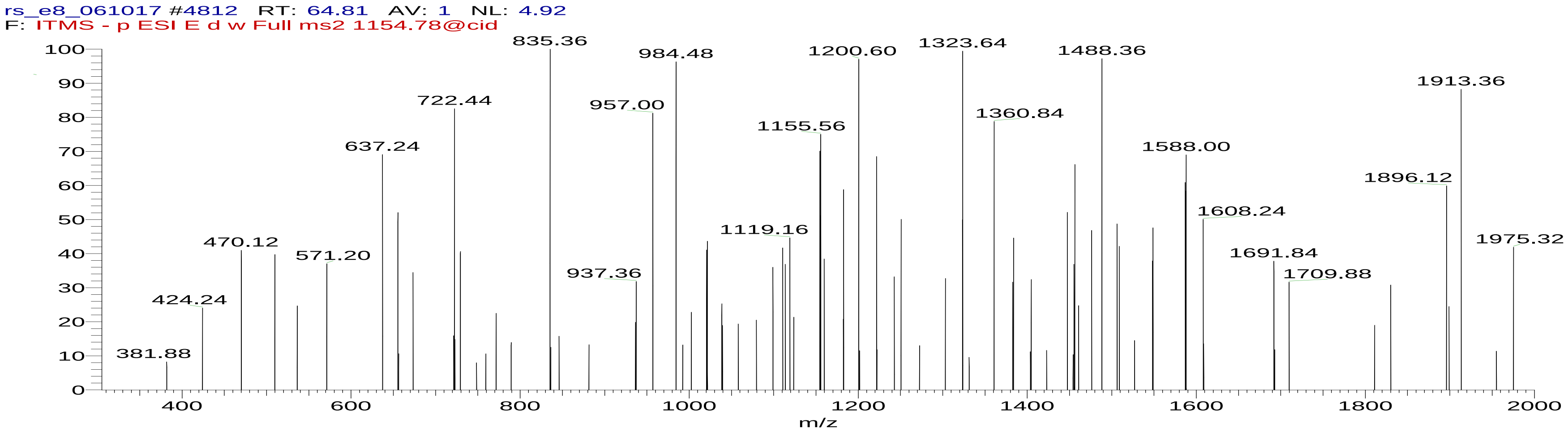

78c

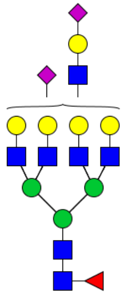

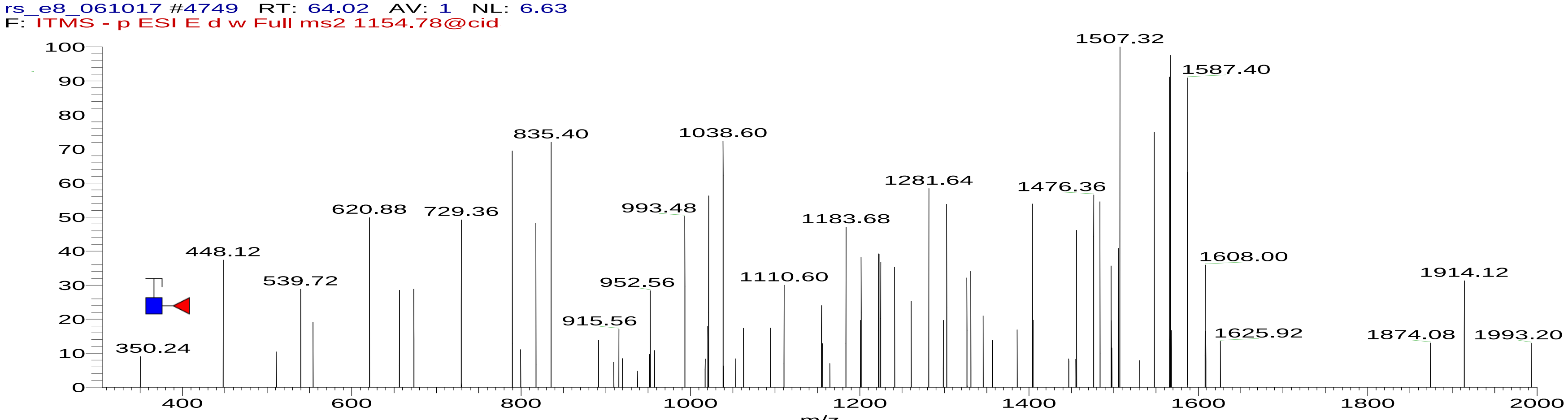

(Hex)4 (HexNAc)4 (NeuAc)4 + (Man)3(GlcNAc)2

No Glytoucan ID

[M-2H] 1767.68

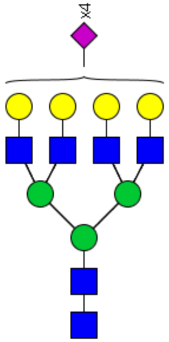

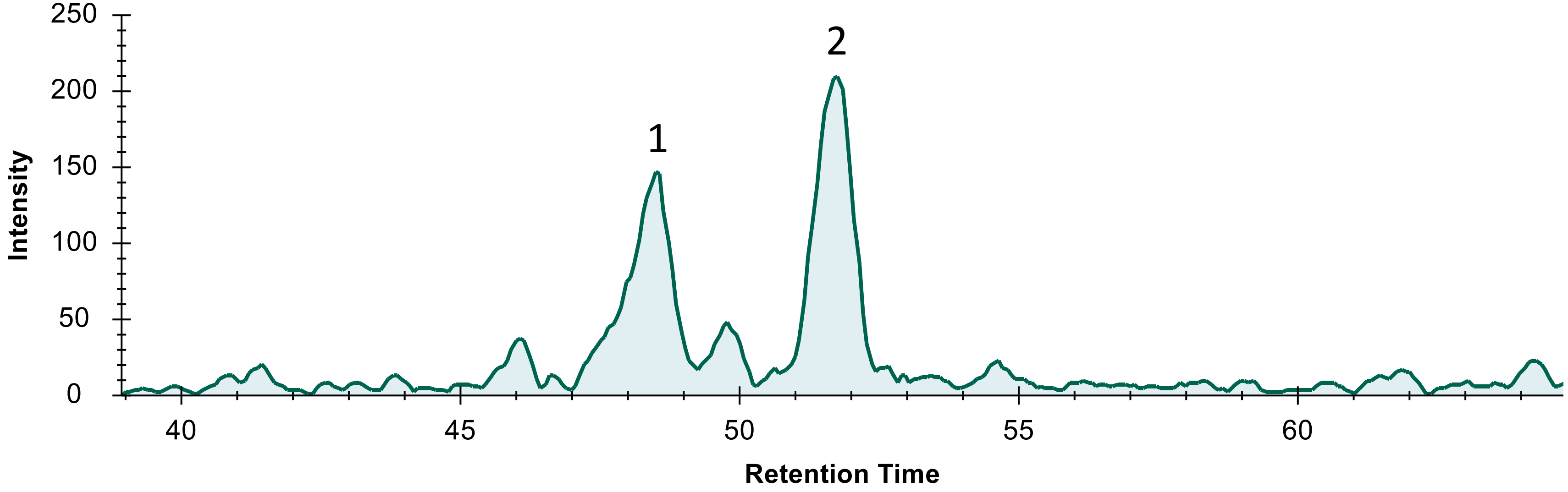

1

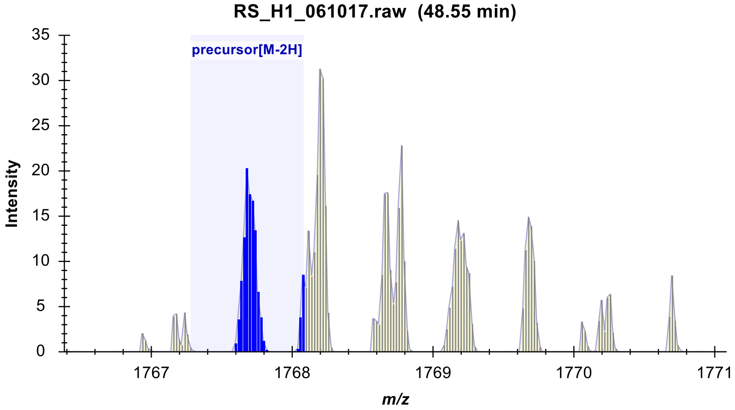

79a

2

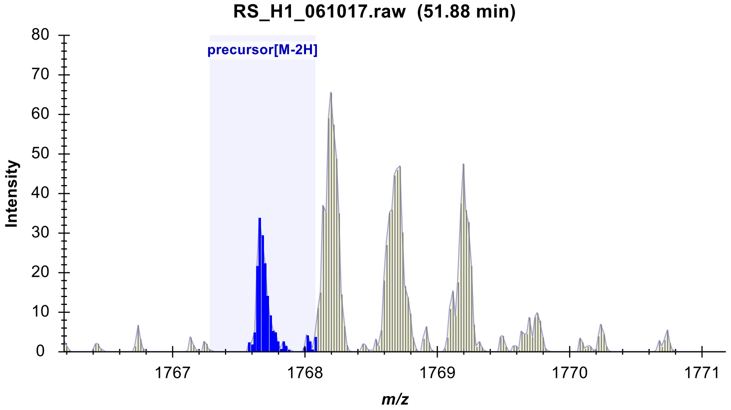

79b

CID-MS/MS
(m/z 1178 $^{3-}$)

79a

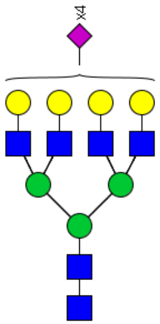

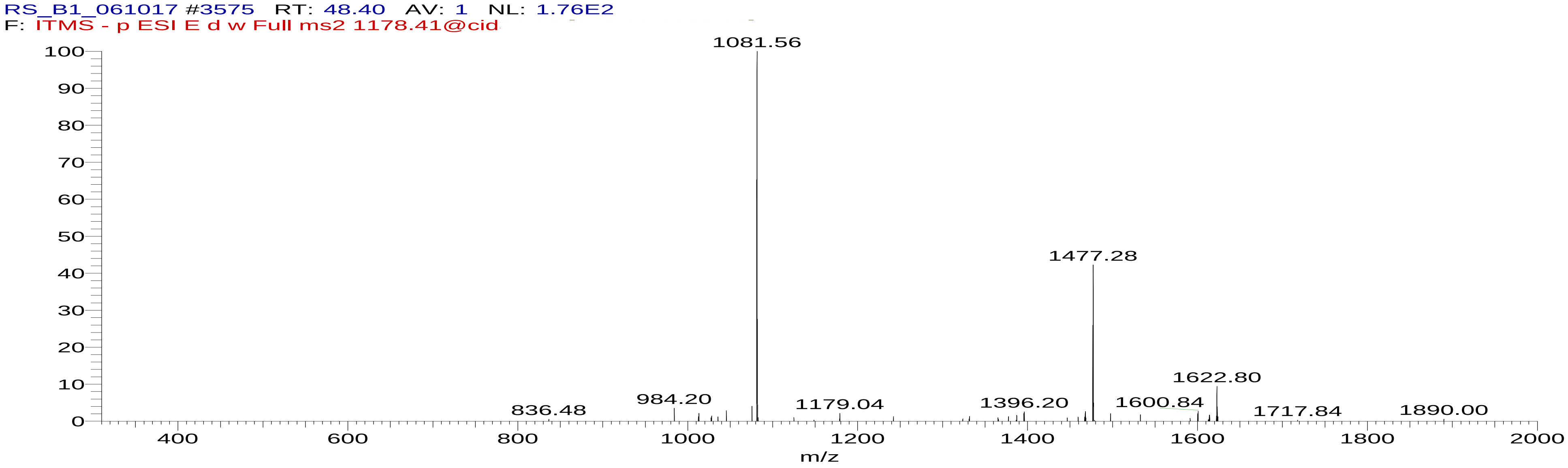

79b

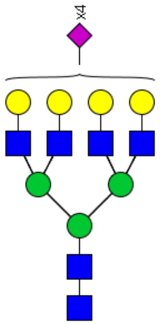

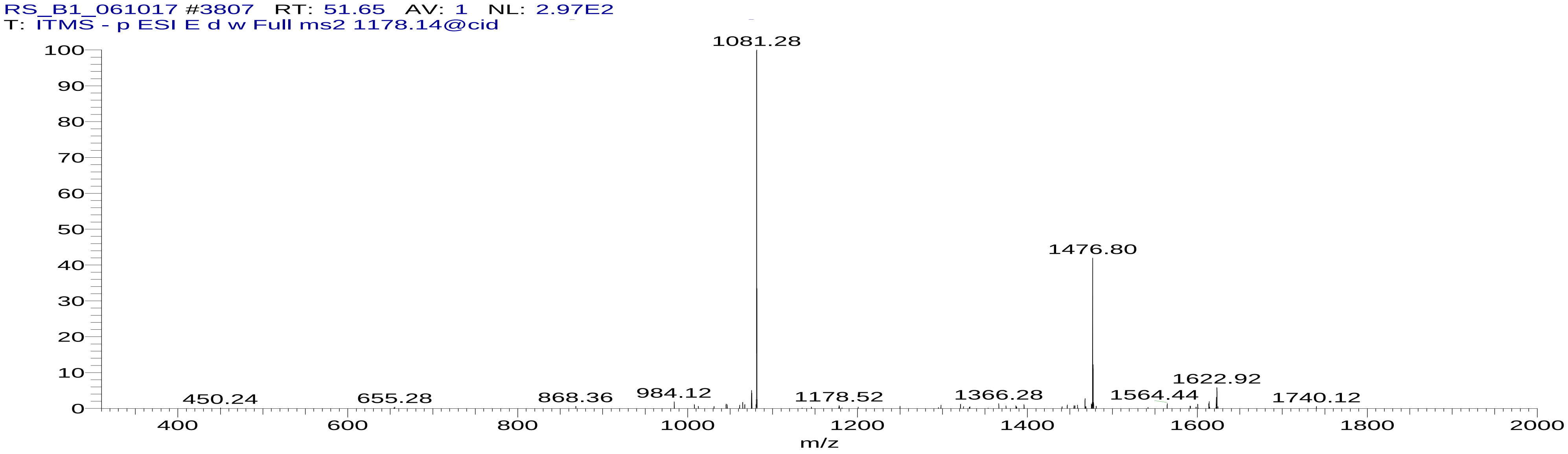

(Hex)4 (HexNAc)4 (Deoxyhexose)1 (NeuAc)4 + (Man)3(GlcNAc)2

No GlytouCan ID

[M-2H] 1840.79

[M-3H] 1227.09

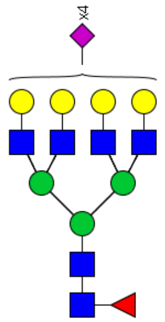

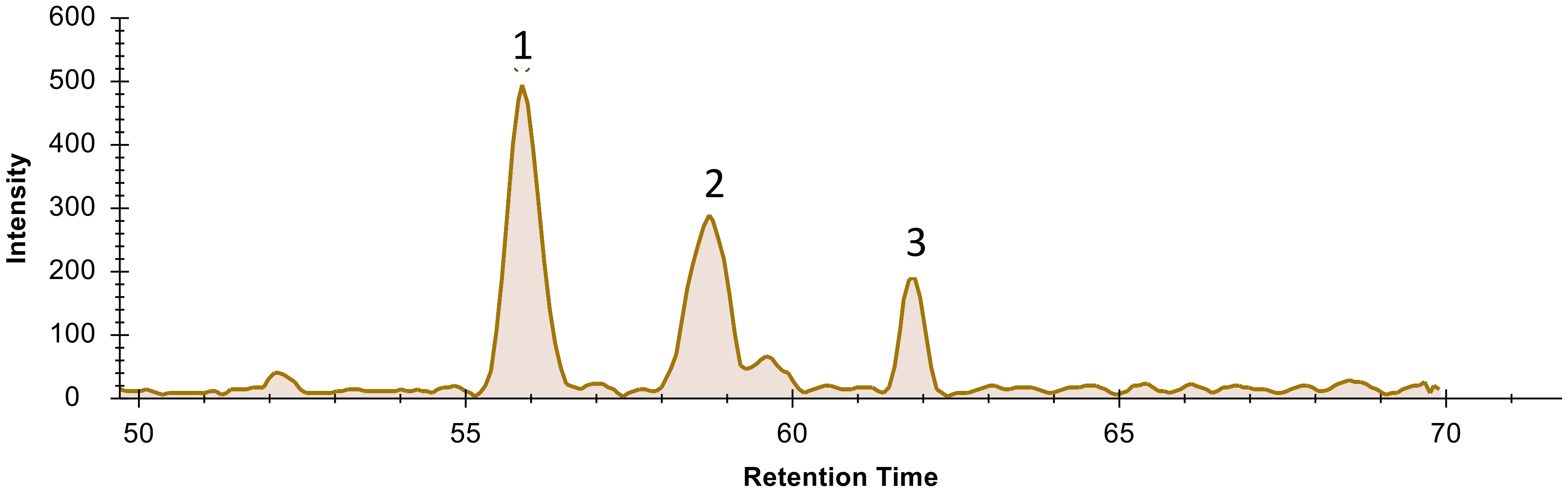

1

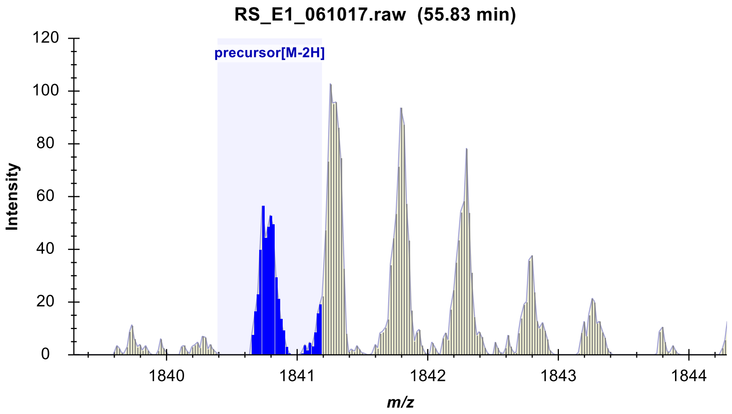

80a

2

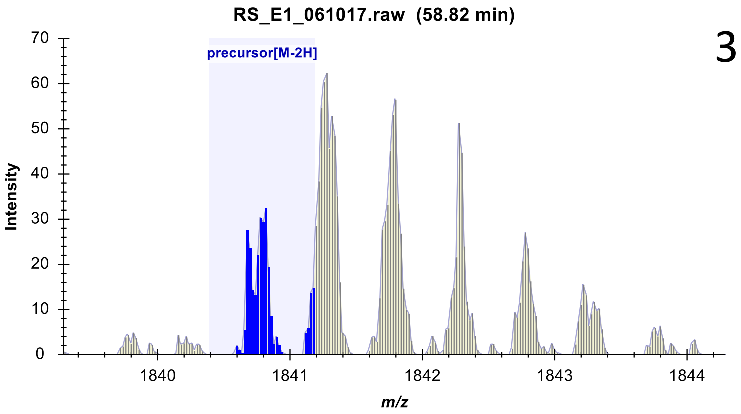

80b

3

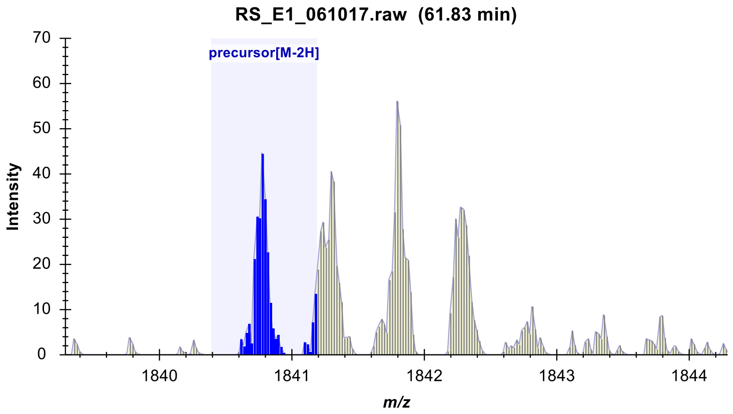

80c

CID-MS/MS
(m/z 1227.09 $^{3-}$)

80a

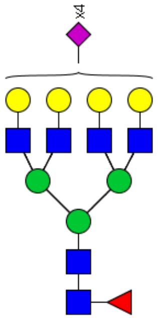

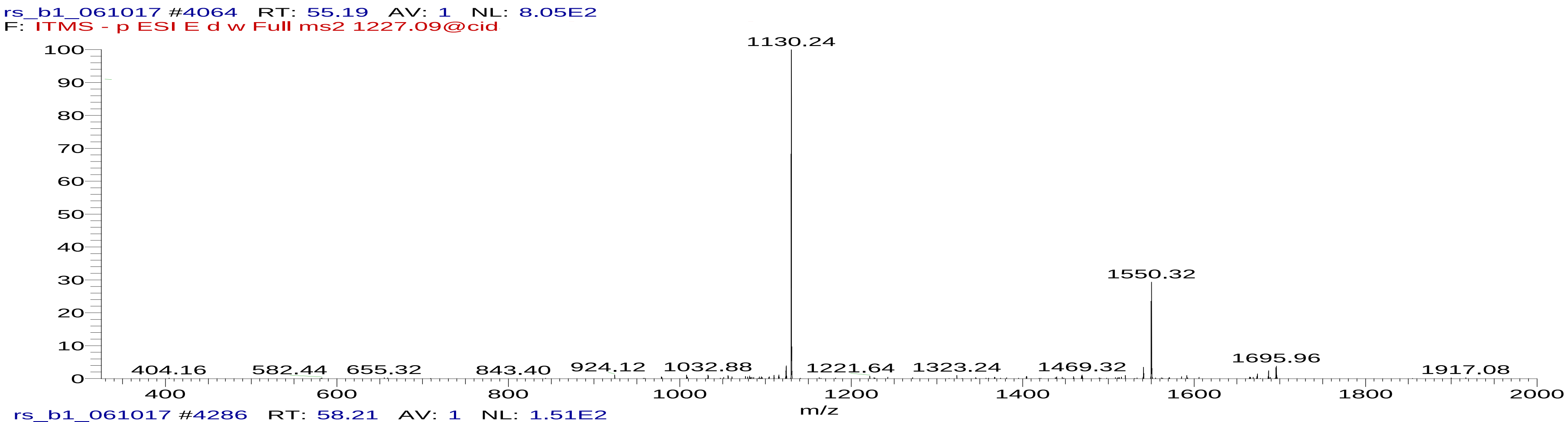

80b

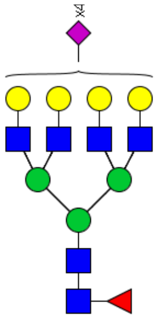

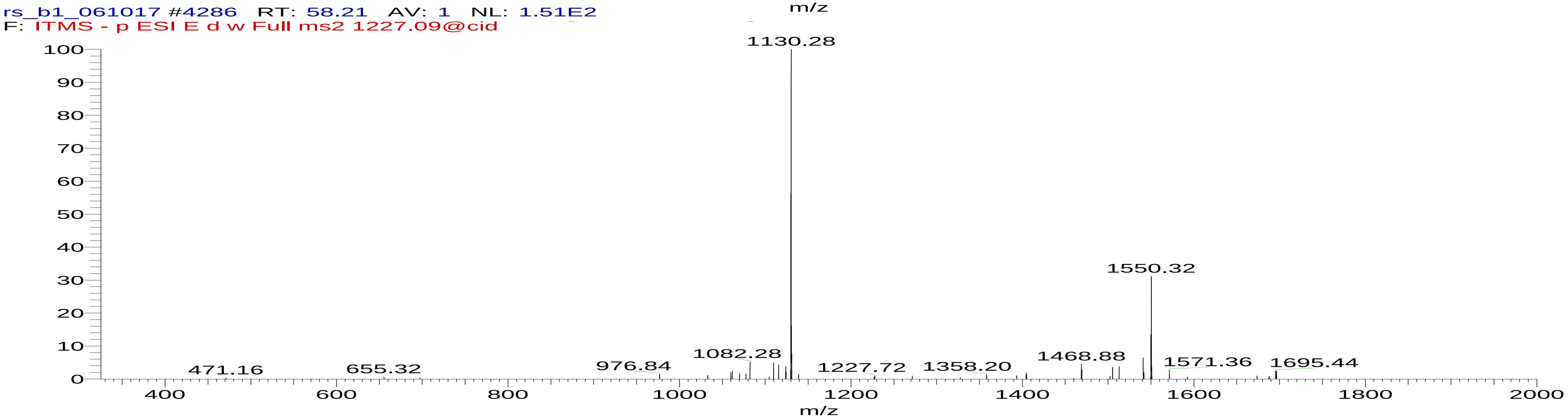

80c

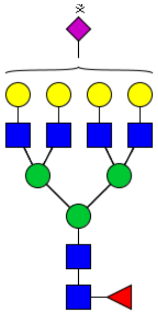

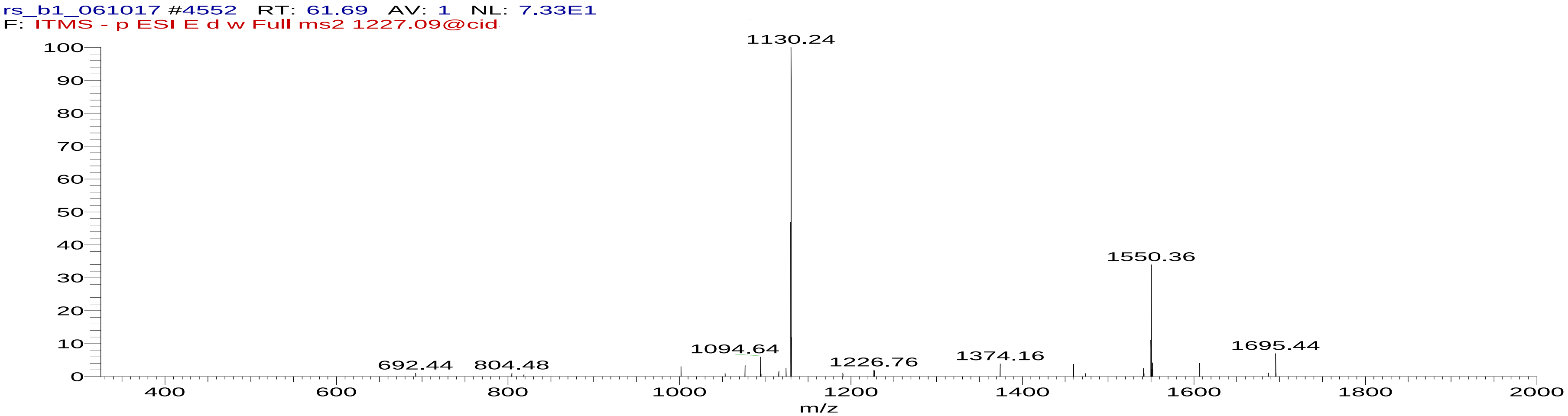

Supplementary Data S2

Spectra evidences for reduced *O*-glycans (alditols) released from PCa and BPH tissues

The *O*-glycans reported in this paper were characterised and quantified by:
a. EIC (MS) and monoisotopic integration area (blue highlights in insert) (Skyline) (top)
b. Retention time
c. CID-MS/MS (-) manual annotation (bottom)

Supporting literature:

1. Hinneburg, H., Pedersen, J. L., Bokil, N. J., Pralow, A., Schirmeister, F., Kawahara, R., Rapp, E., Saunders, B. M., and Thaysen-Andersen, M. (2020) High-resolution longitudinal N- and O-glycoprofiling of human monocyte-to- macrophage transition. *Glycobiology*
2. Everest-Dass, A. V., Abrahams, J. L., Kolarich, D., Packer, N. H., and Campbell, M. P. (2013) Structural feature ions for distinguishing N- and O-linked glycan isomers by LC-ESI-IT MS/MS. *J Am Soc Mass Spectrom* **24**, 895-906

(Hex)1 (HexNAc)1 (Deoxyhexose)1
GlytouCan ID: G05187DJ
[M-H] 530.15

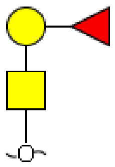

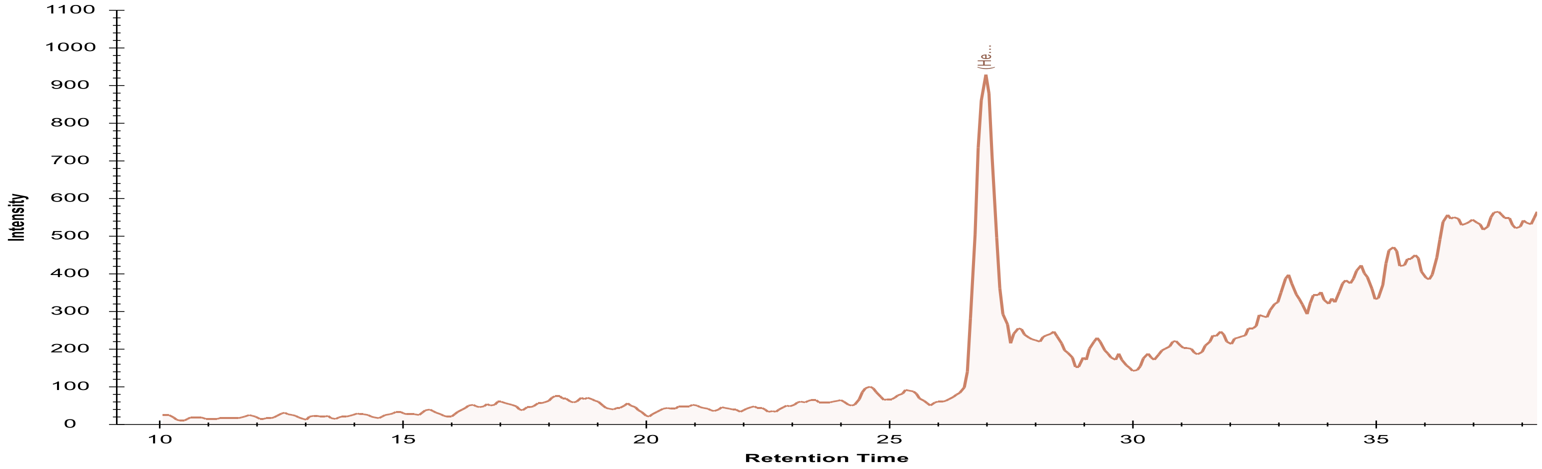

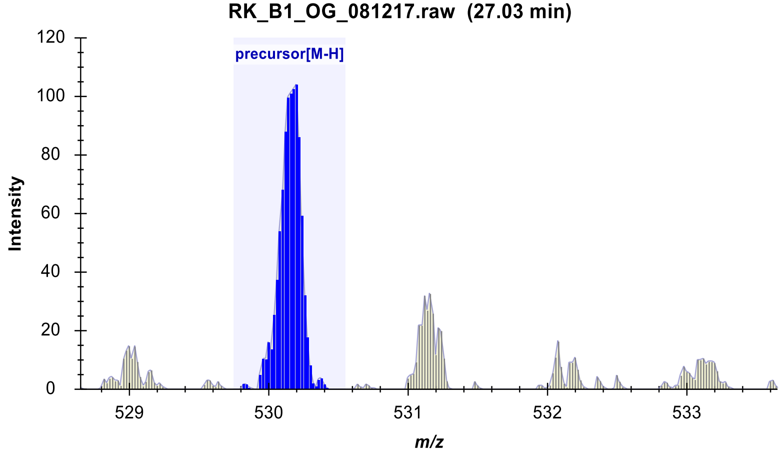

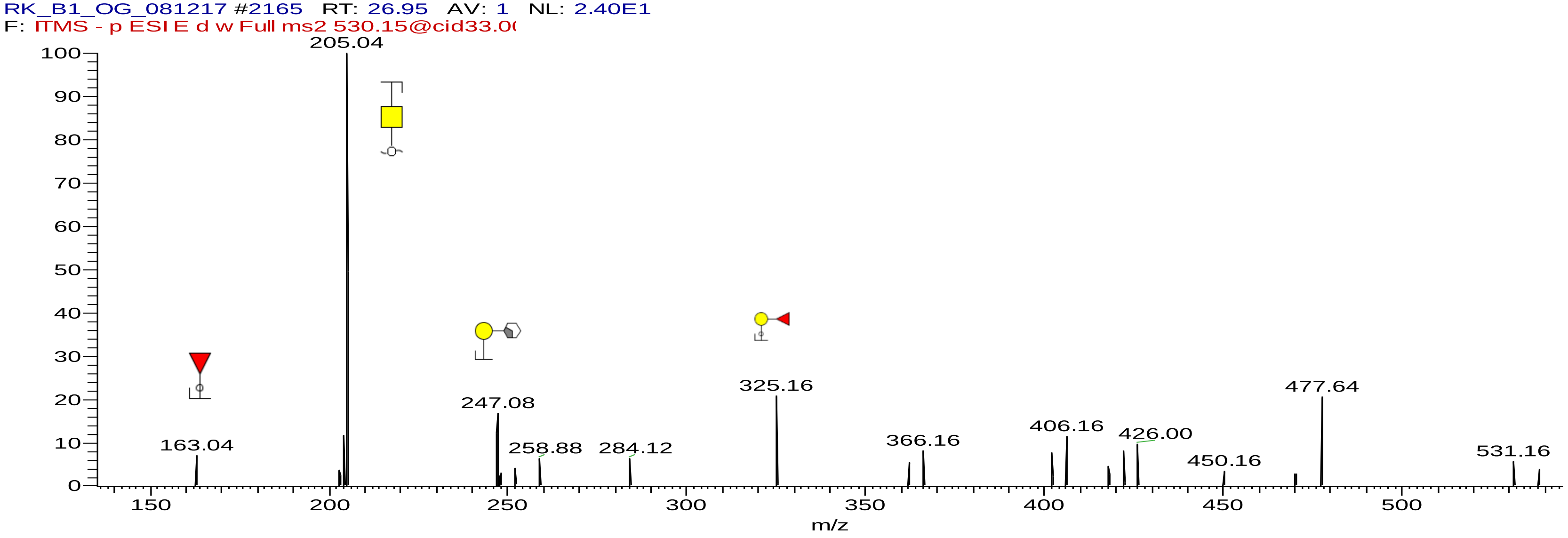

Glycan ID: 84

(Hex)1 (HexNAc)1 (NeuAc)1

GlytouCan ID: G65285QO

[M-H] 675.26

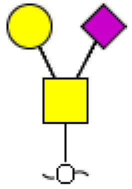

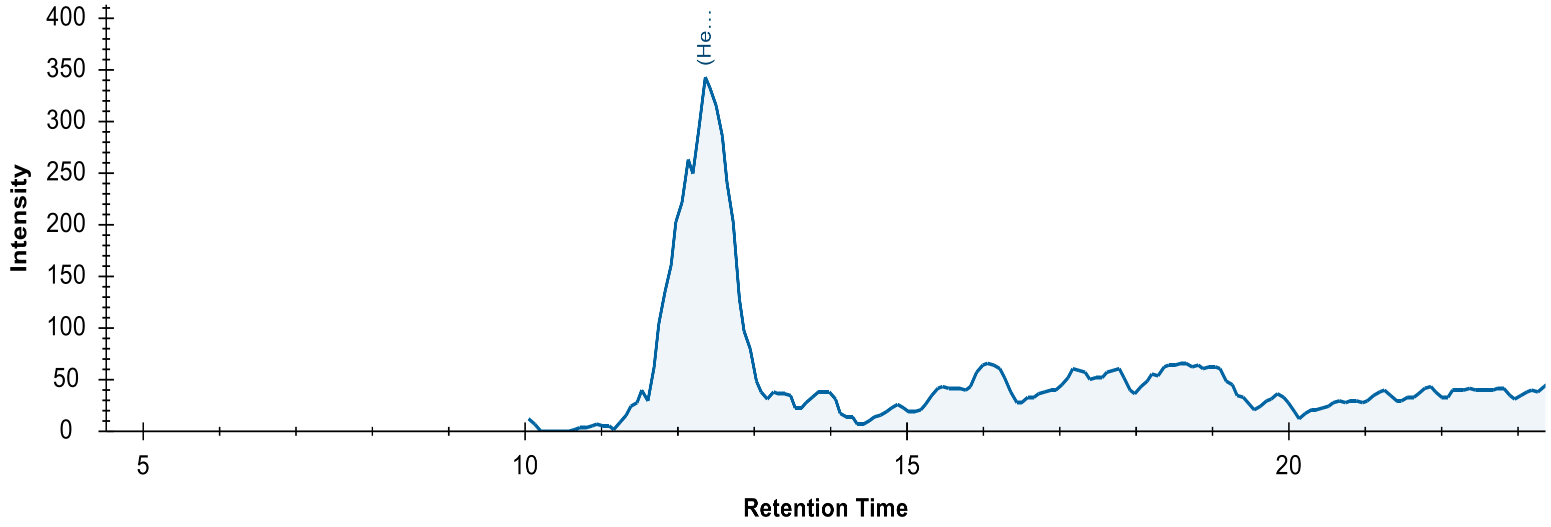

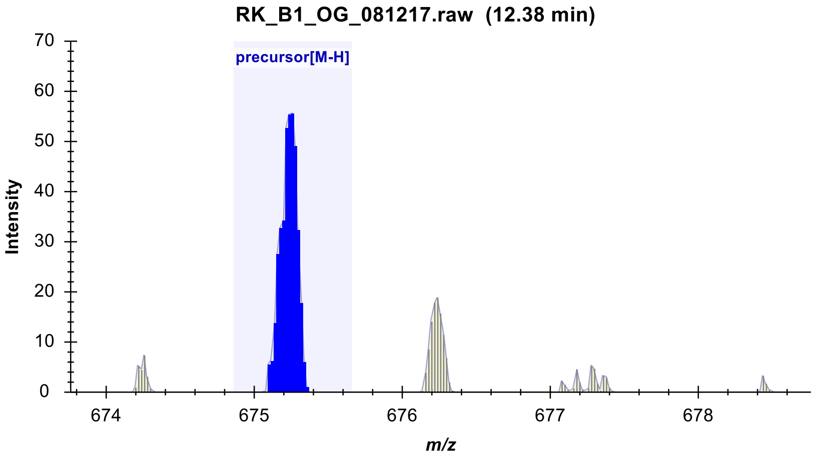

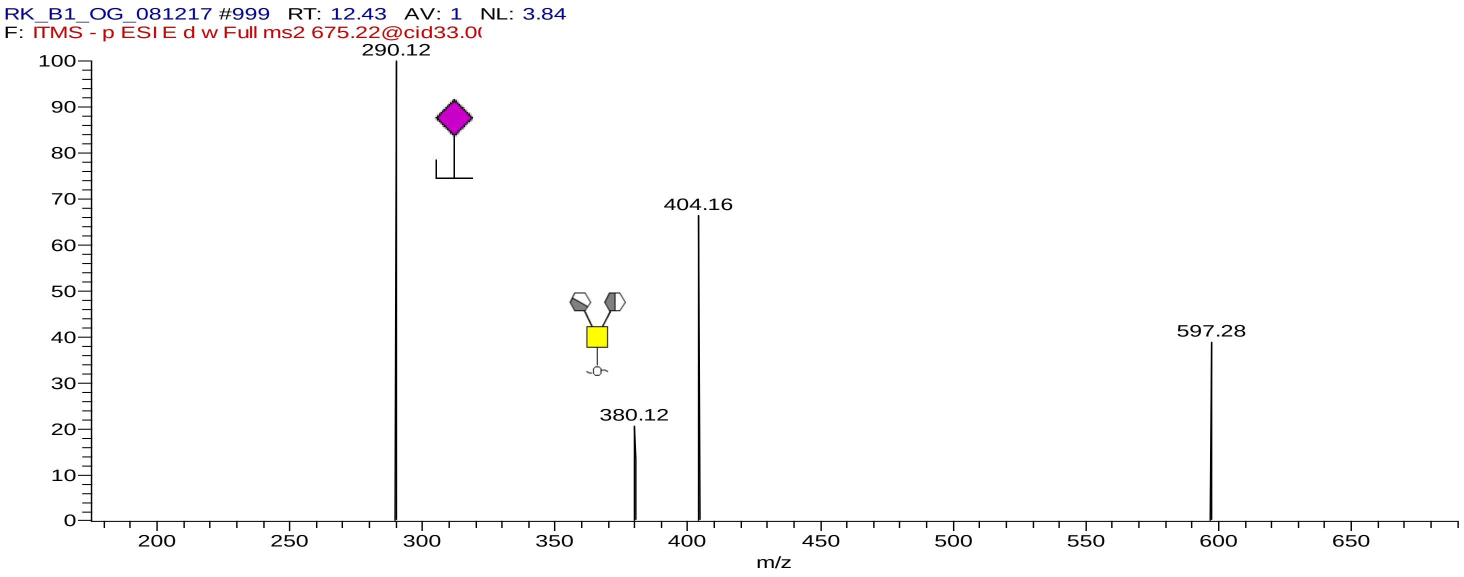

Glycan ID: 85

(Hex)1 (HexNAc)1 (NeuAc)2
GlytouCan ID: G84906ML
[M-H] 966.36

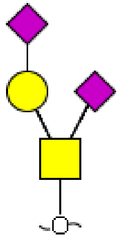

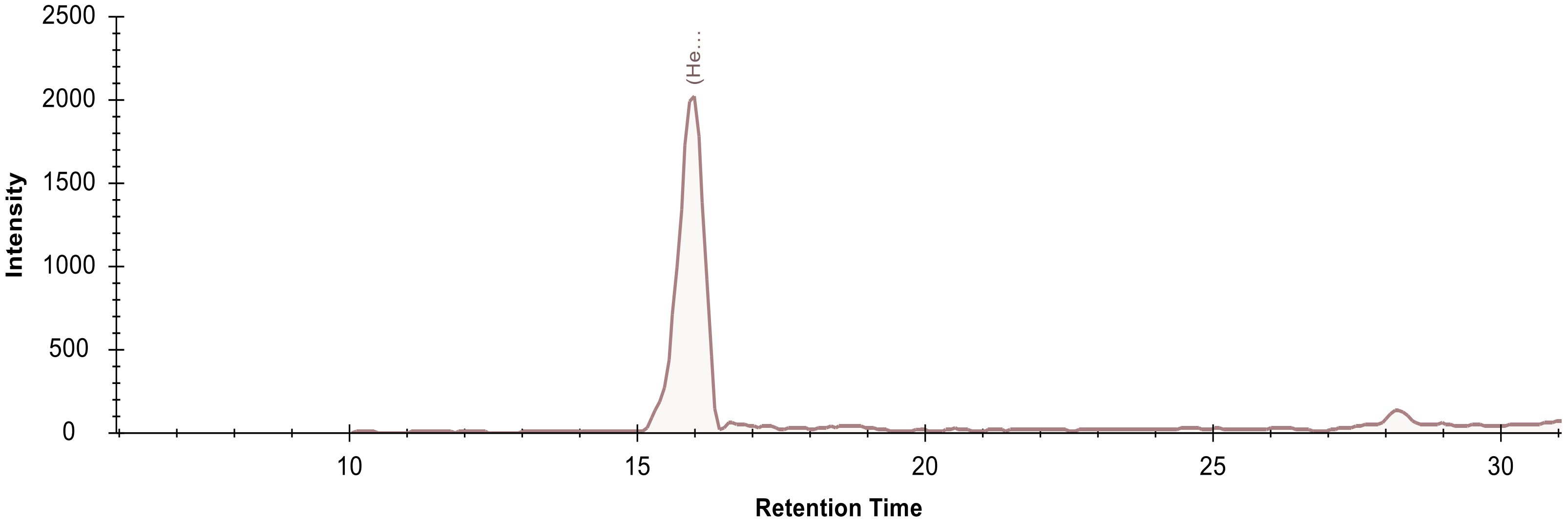

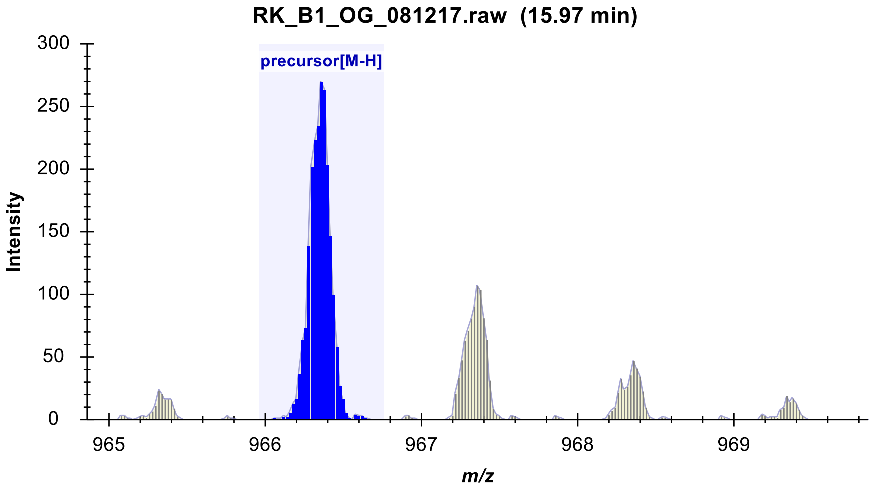

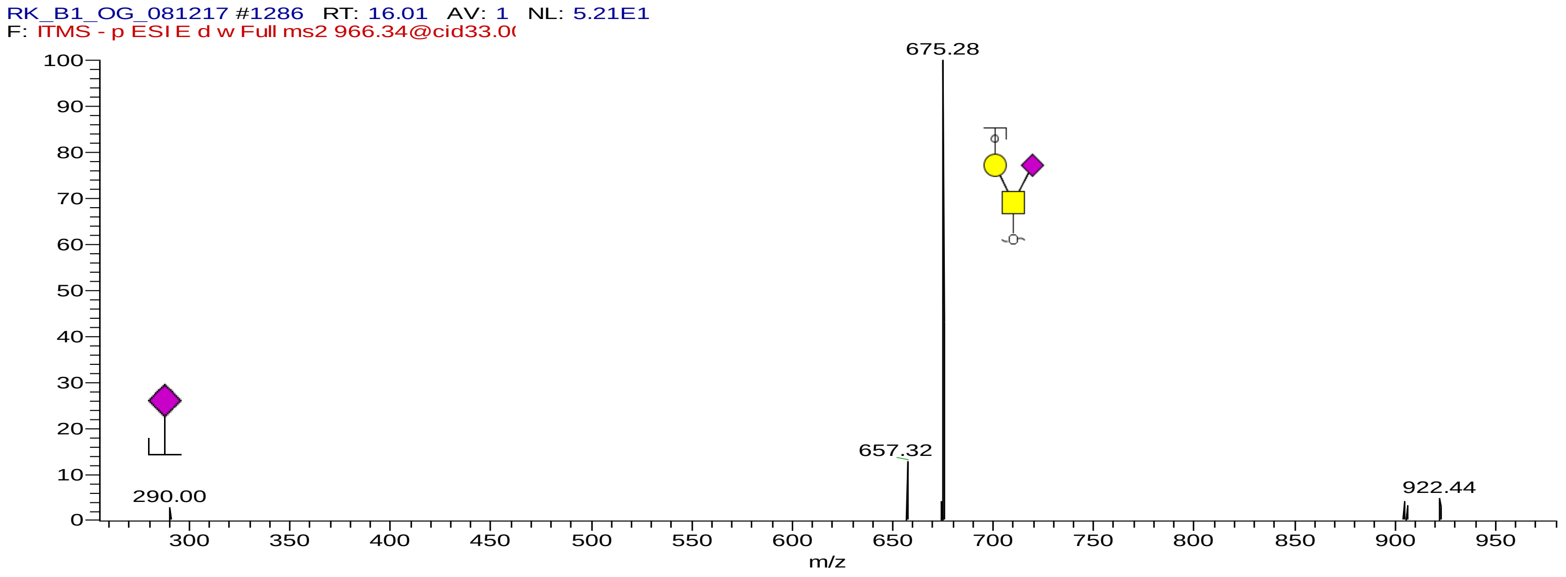

Glycan ID: 86

(Hex)1 (HexNAc)2
GlytouCan ID: G31601FB
[M-H] 587.18

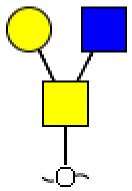

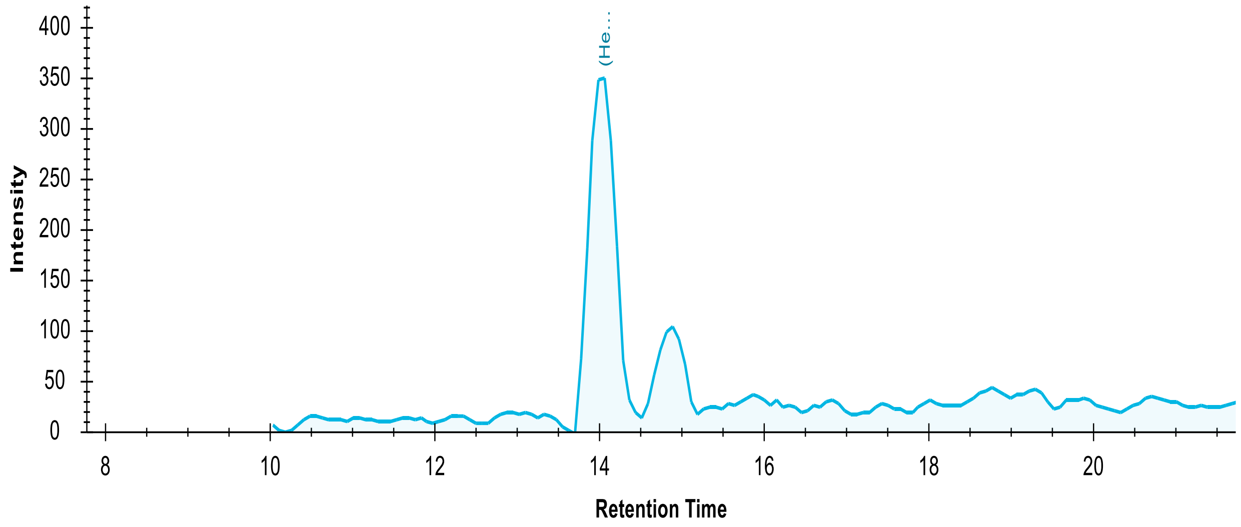

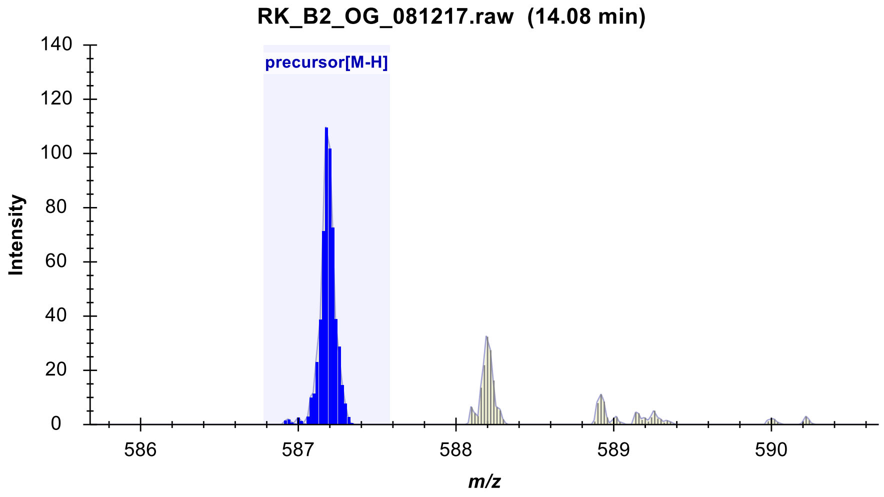

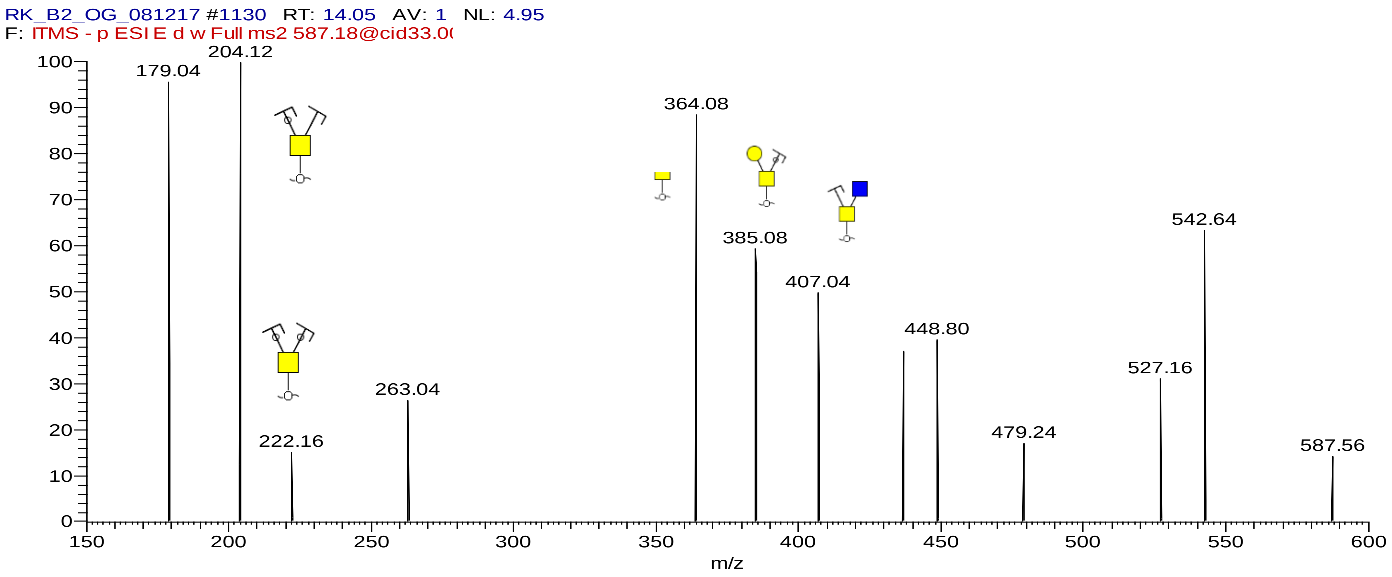

Glycan ID: 87

(Hex)1 (HexNAc)2 (NeuAc)1
GlytouCan ID: G35679QA
[M-H] 878.36

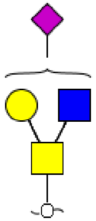

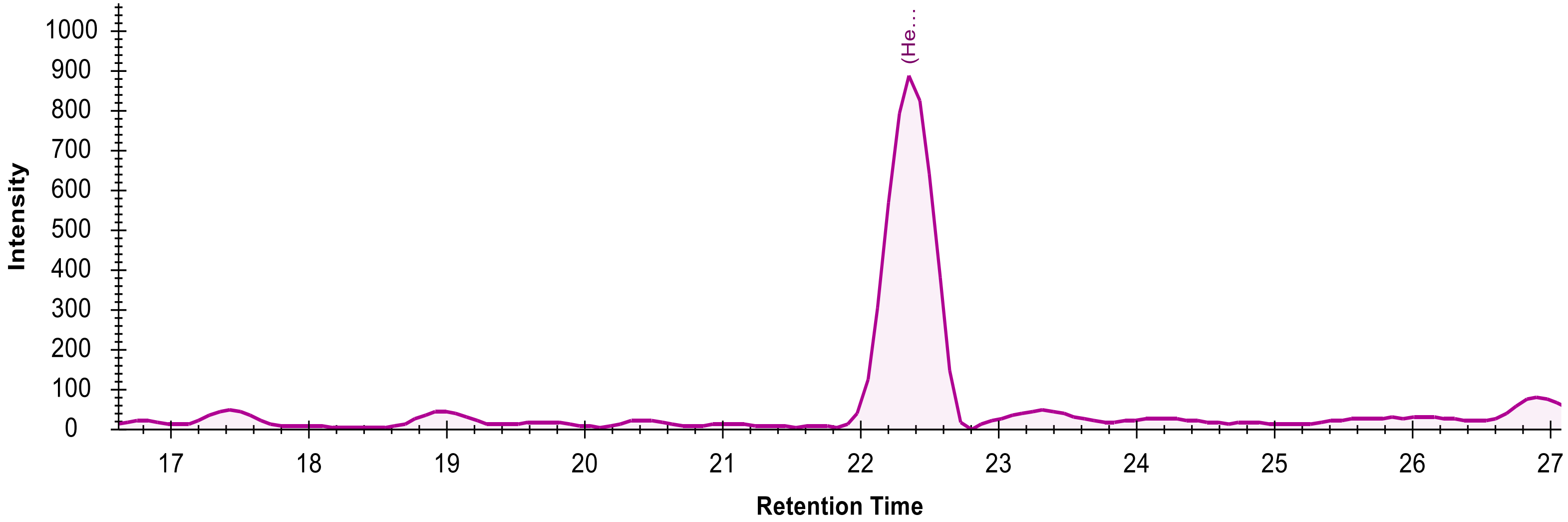

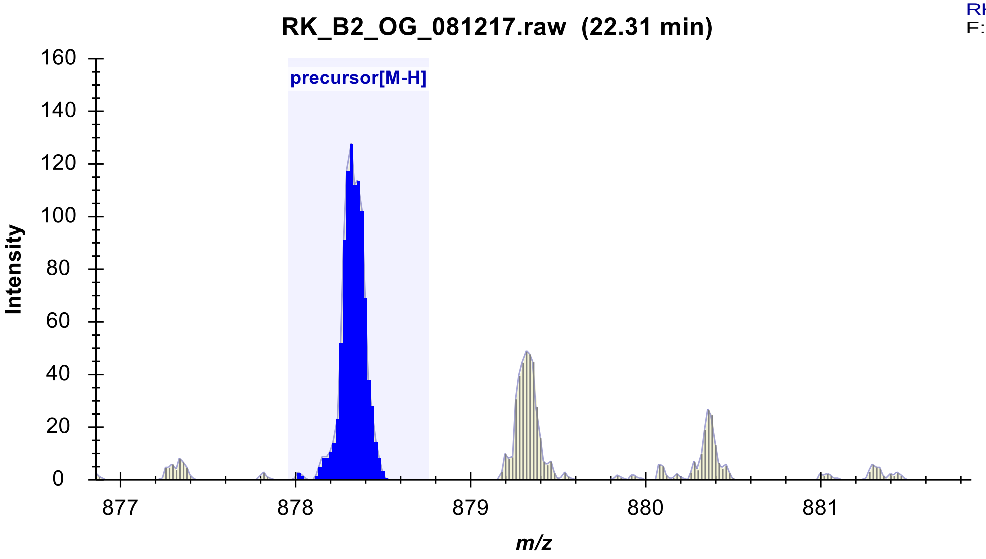

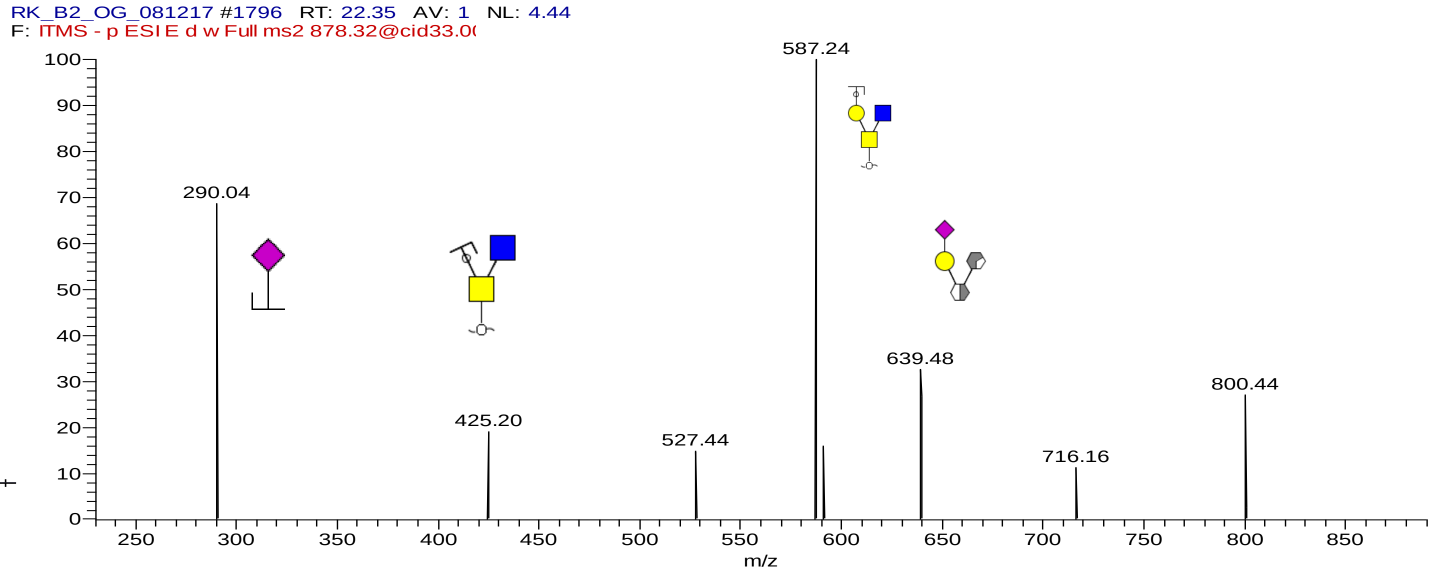

Glycan ID: 88

(Hex)2 (HexNAc)1

No GlytouCan ID

546.17

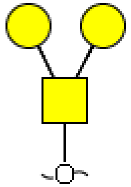

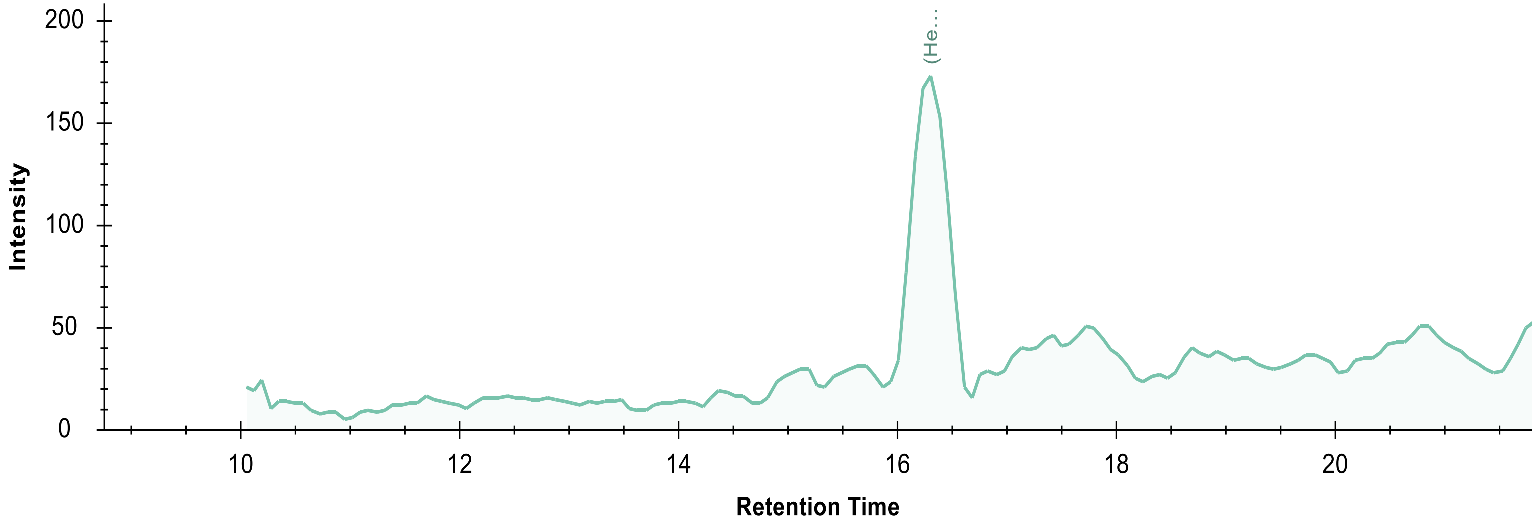

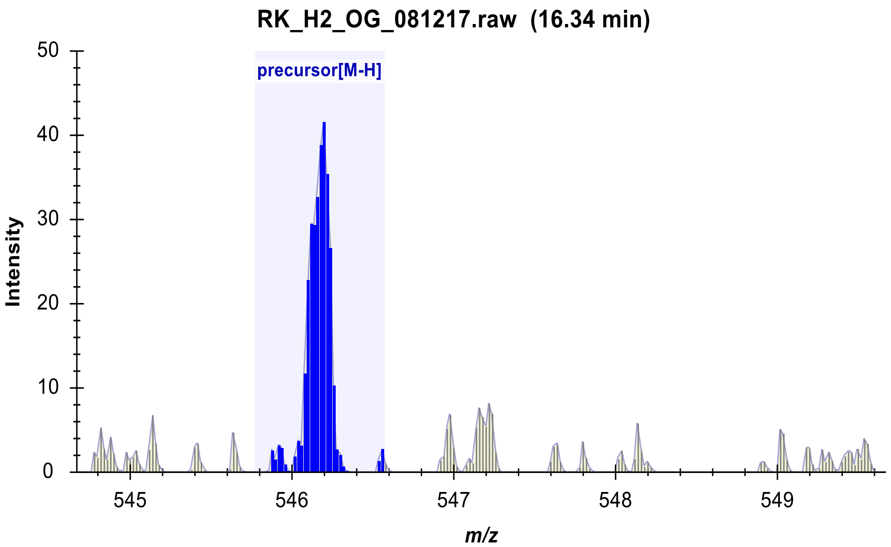

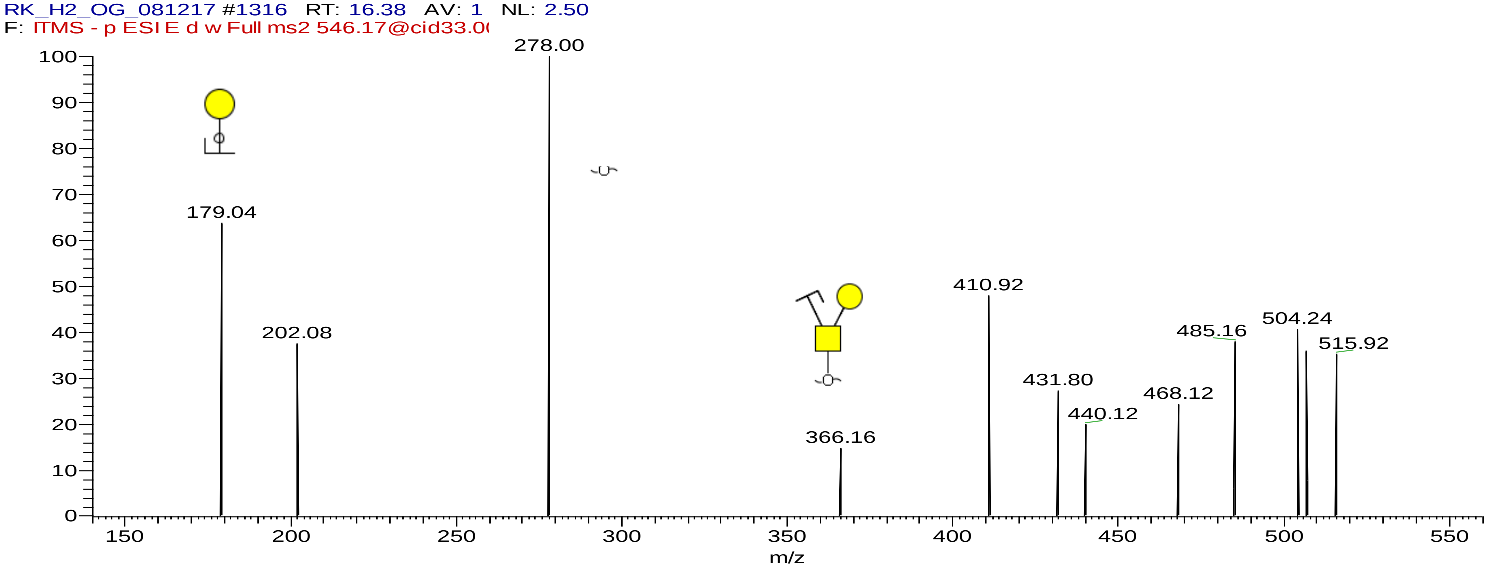

Glycan ID: 89

(Hex)2 (HexNAc)1 (NeuAc)1
No GlytouCan ID
[M-H] 837.31

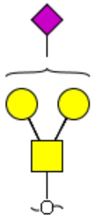

1

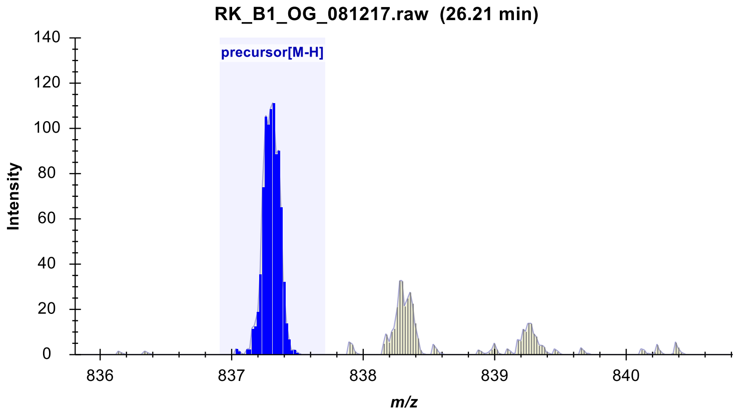

90a

2

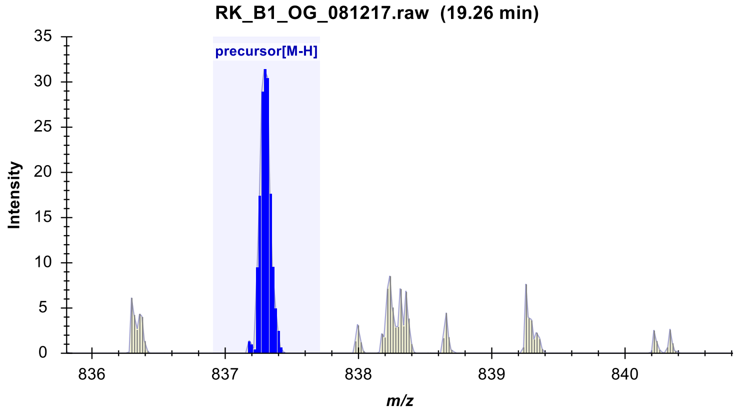

90b

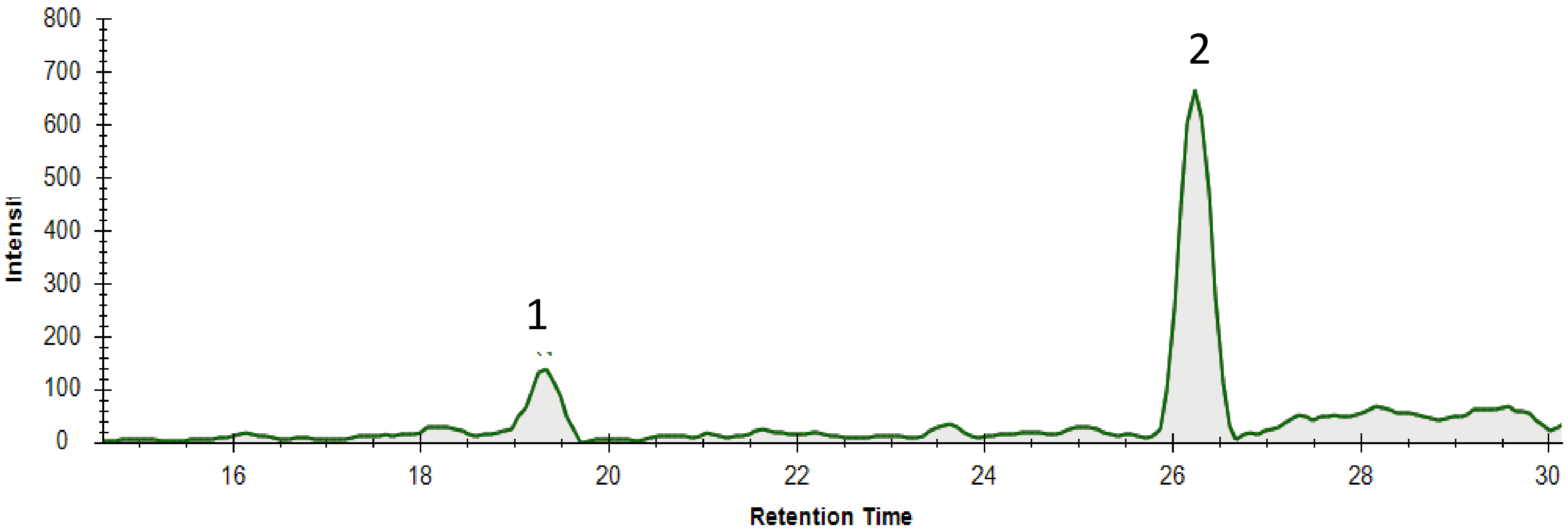

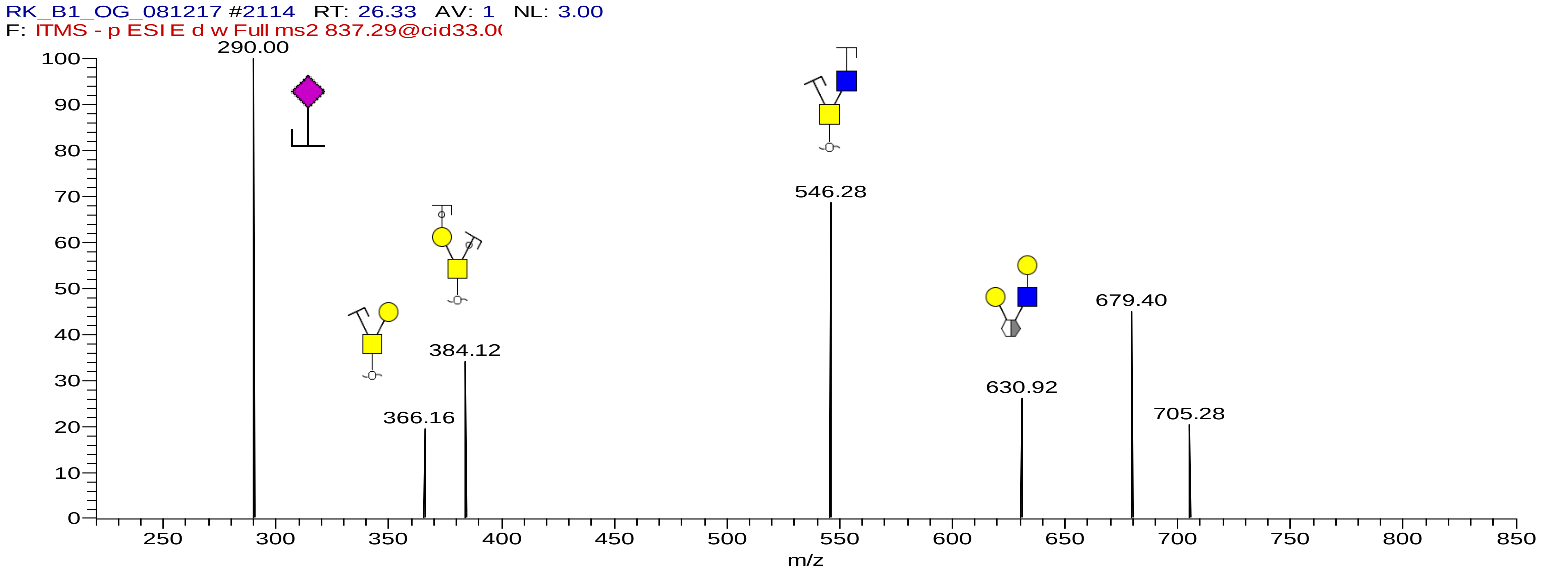

(Hex)2 (HexNAc)2

GlytouCan ID: G18132YR

[M-H] 749.28

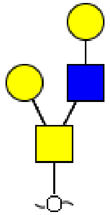

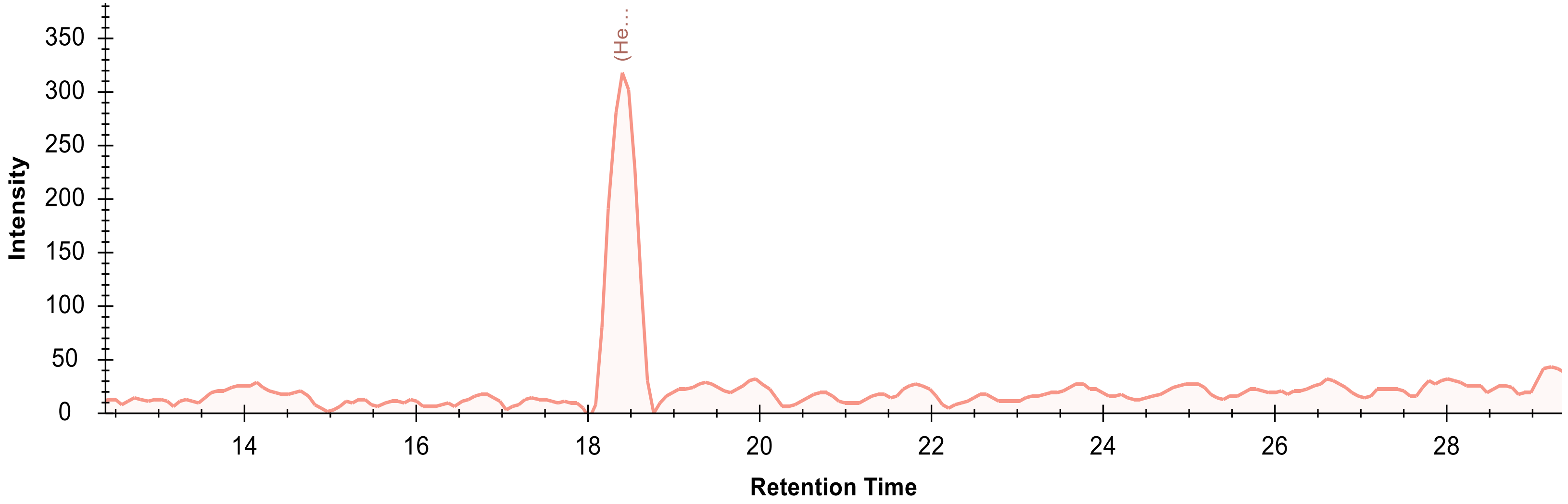

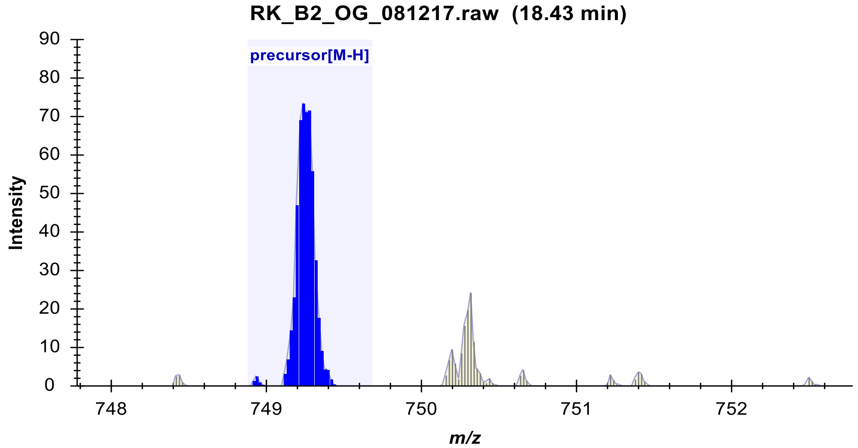

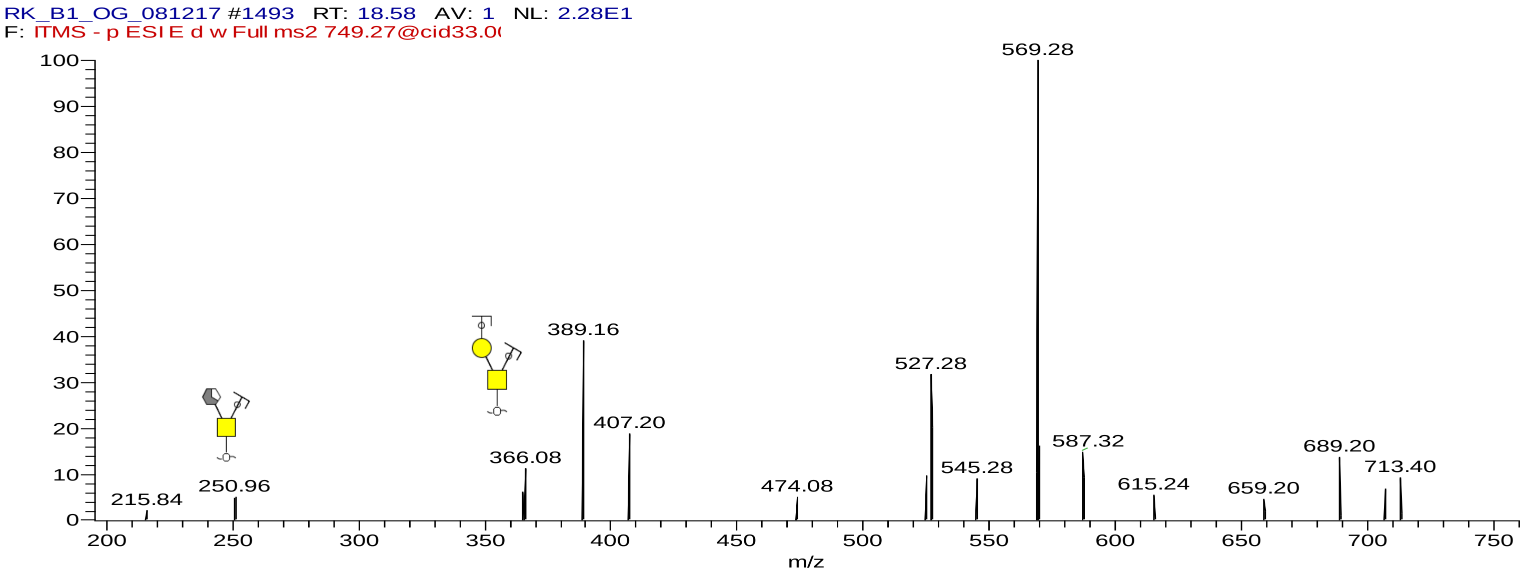

Glycan ID: 91

(Hex)2 (HexNAc)2 (Deoxyhexose)1 (NeuAc)1

GlytouCan ID: G02353KU

[M-H] 1186.5

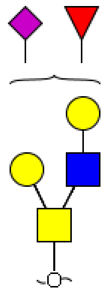

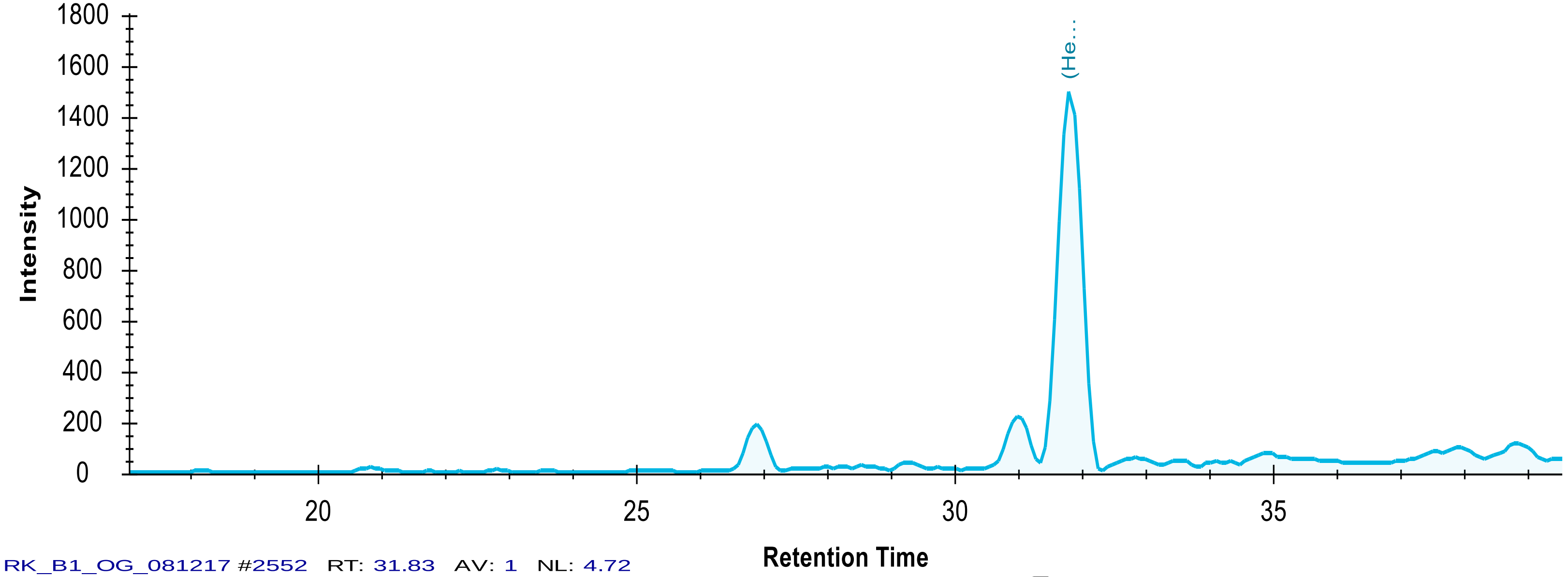

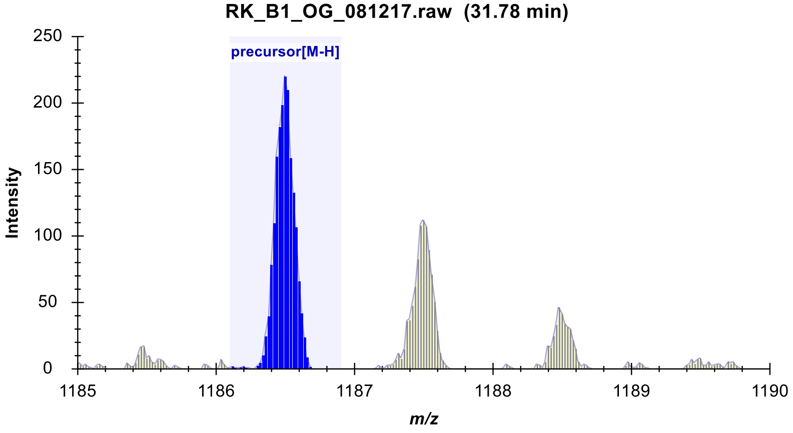

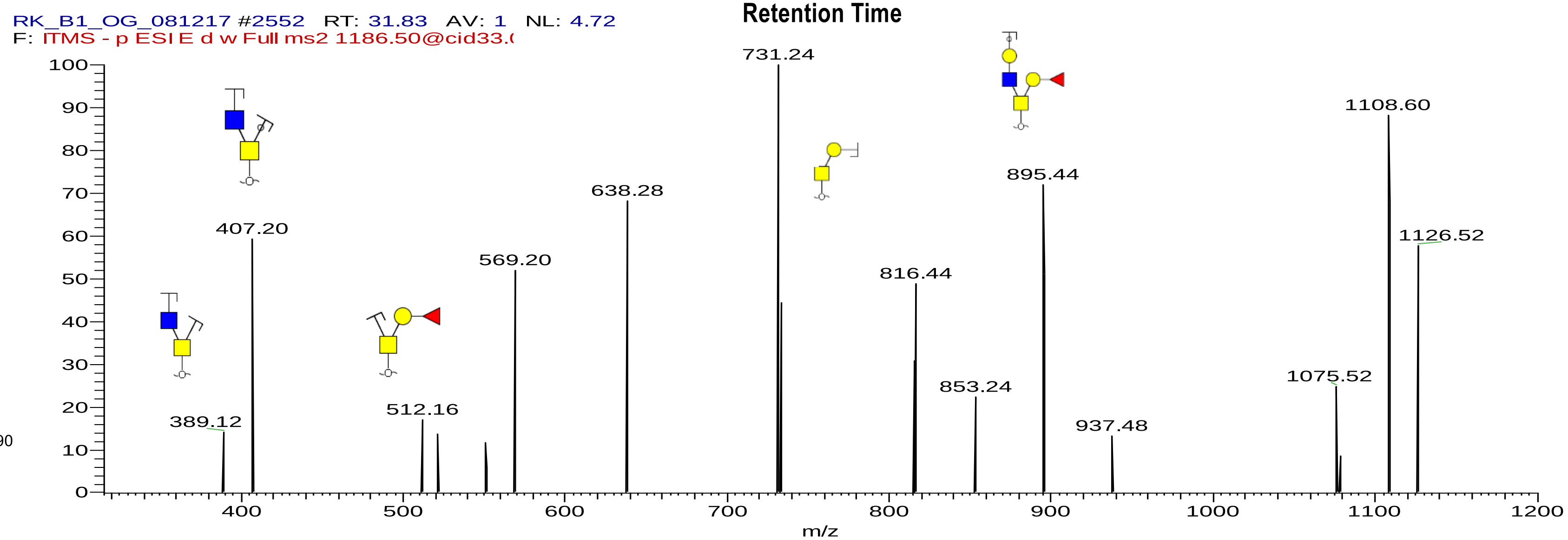

Glycan ID: 92

(Hex)2 (HexNAc)2 (Deoxyhexose)1
GlytouCan ID: G38812YS
[M-H] 895.34

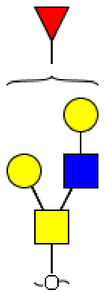

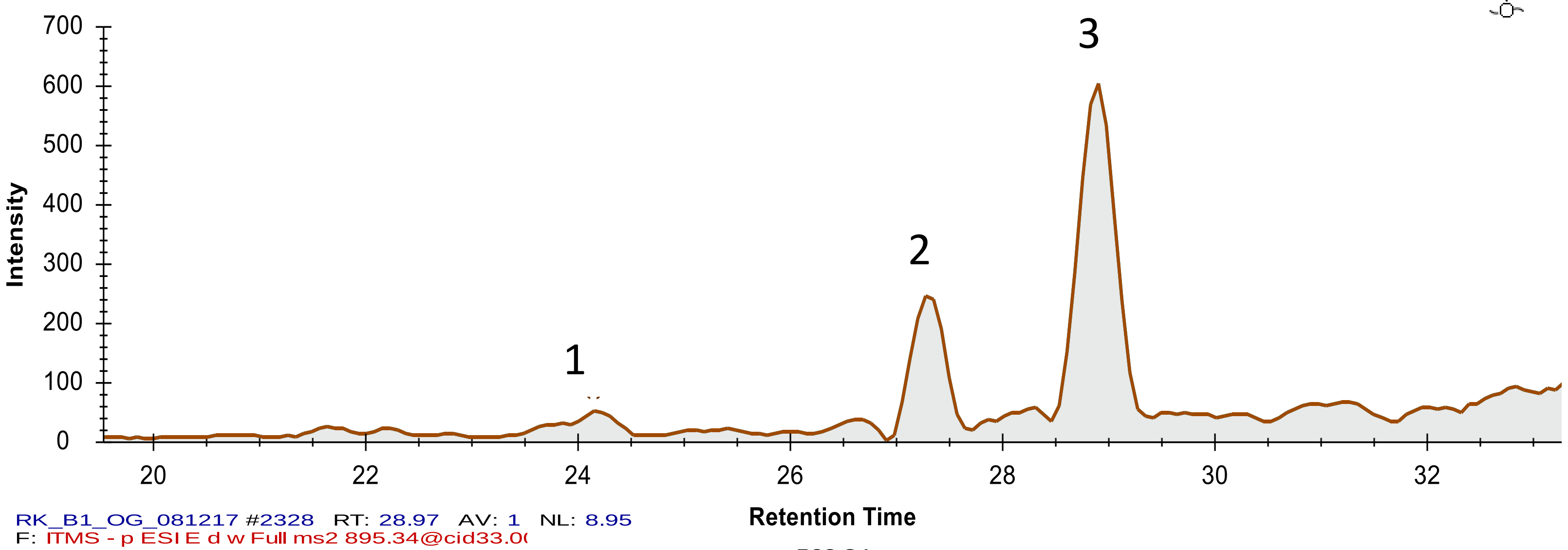

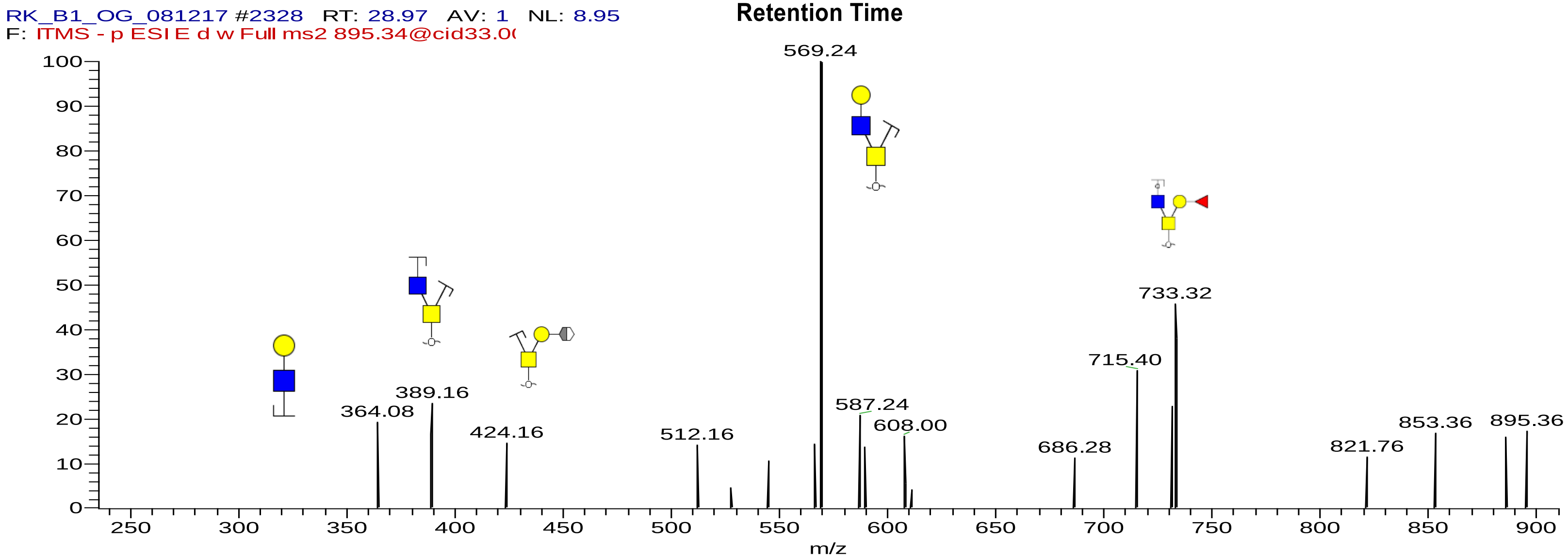

1

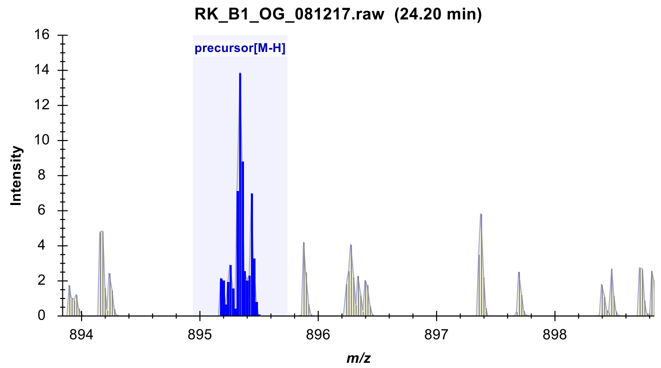

93a

2

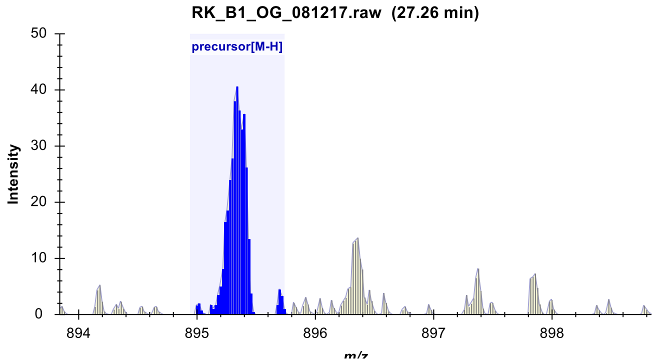

93b

3

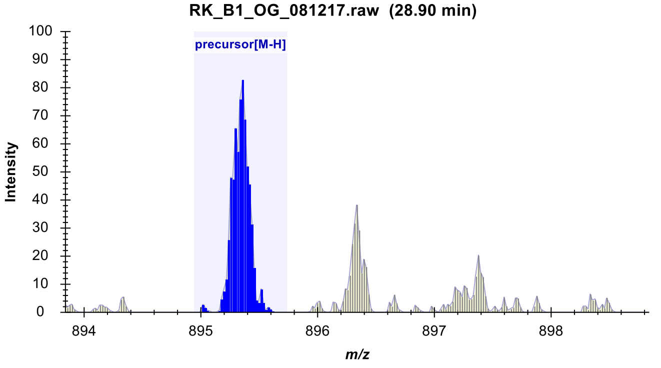

93c

(Hex)2 (HexNAc)2 (NeuAc)1
GlytouCan ID: G08140FC
[M-H] 1040.41

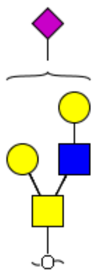

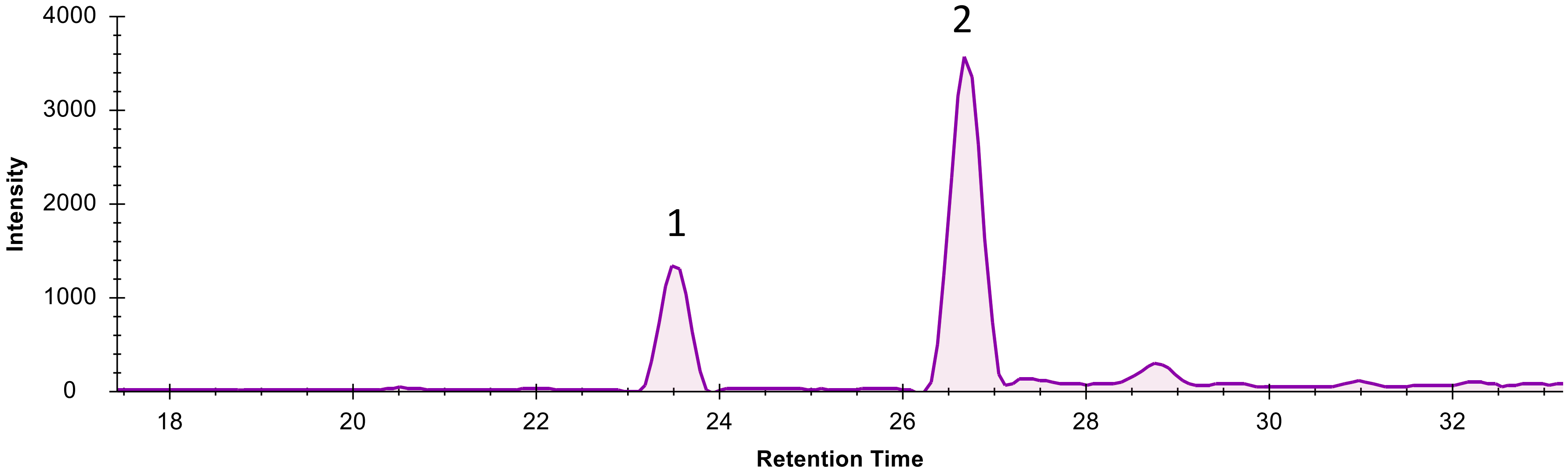

1

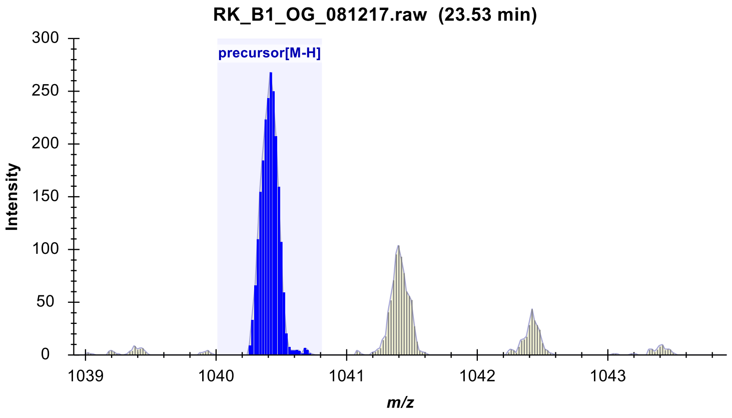

94a

2

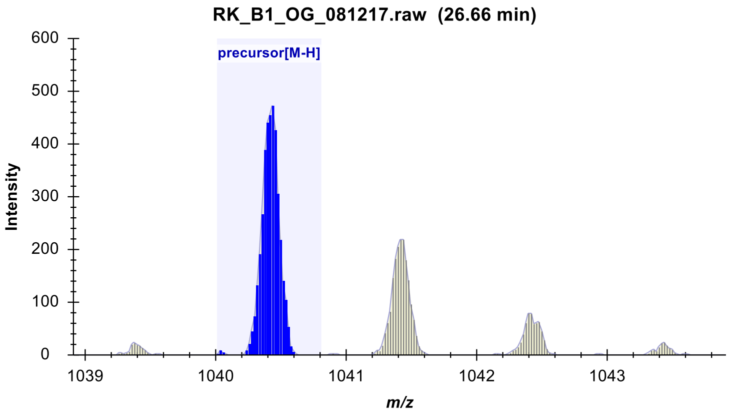

94b

GlytouCan ID: G08140FC

CID-MS/MS

(m/z 1040.41^{1-})

94a

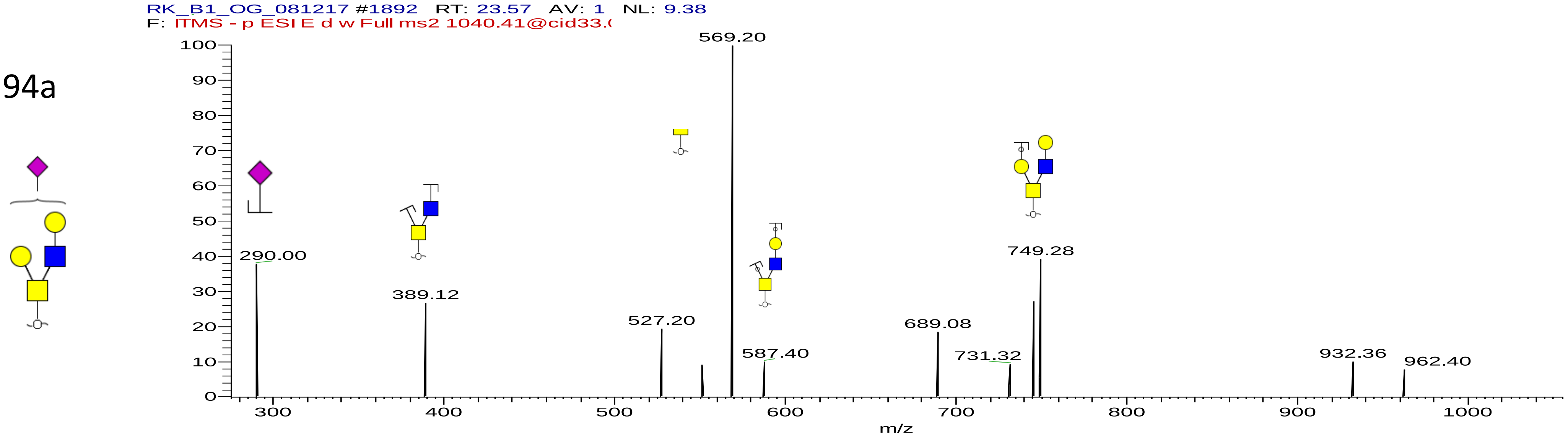

94b

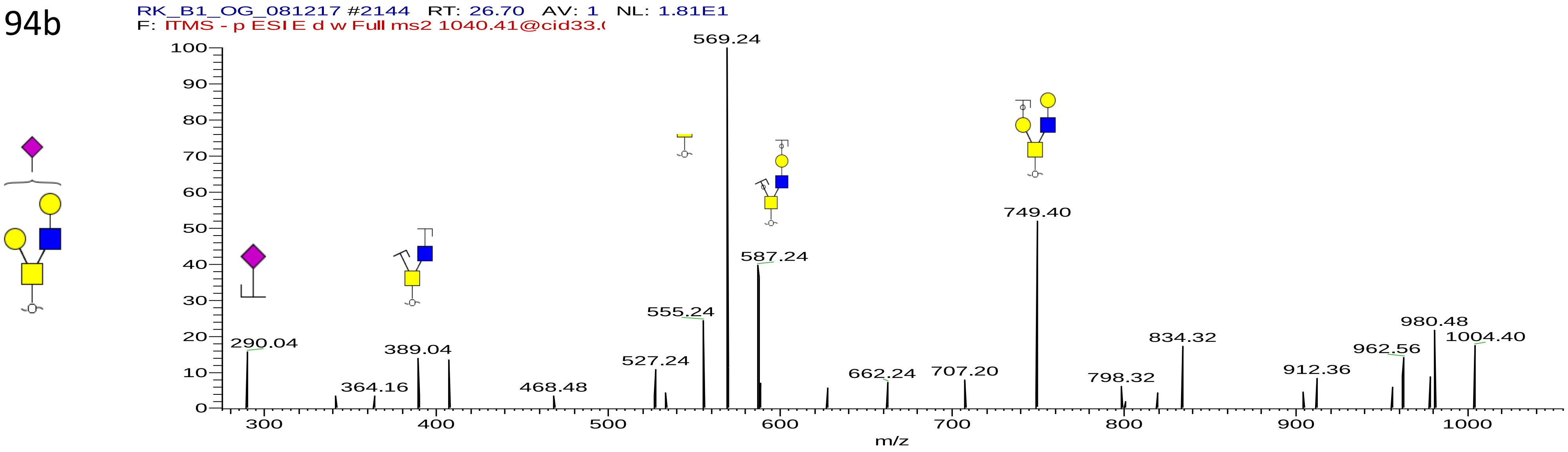

(Hex)2 (HexNAc)2 (NeuAc)2
GlytouCan ID: G52120NK
[M-H] 1331.54

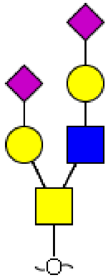

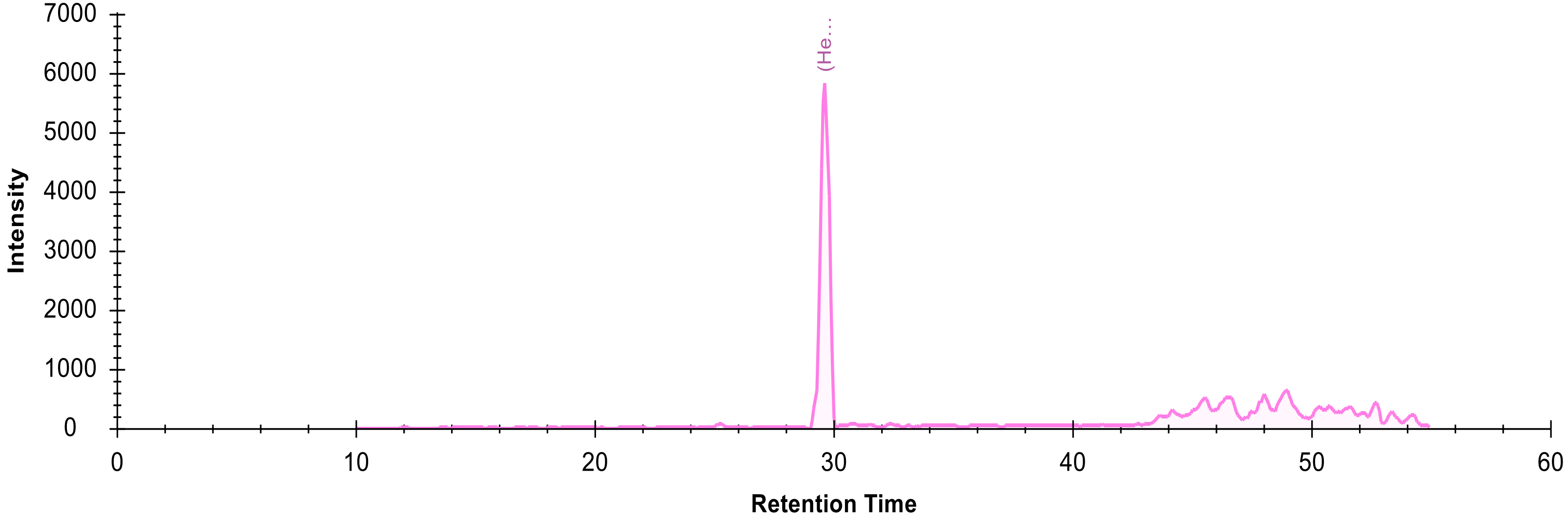

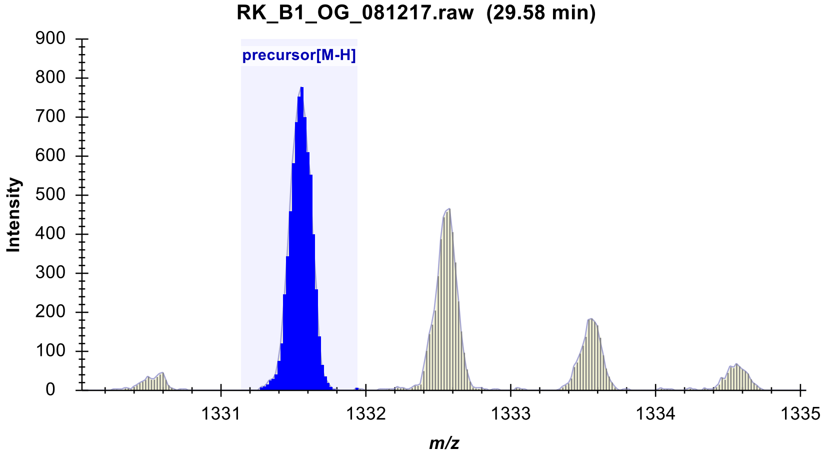

Glycan ID: 95

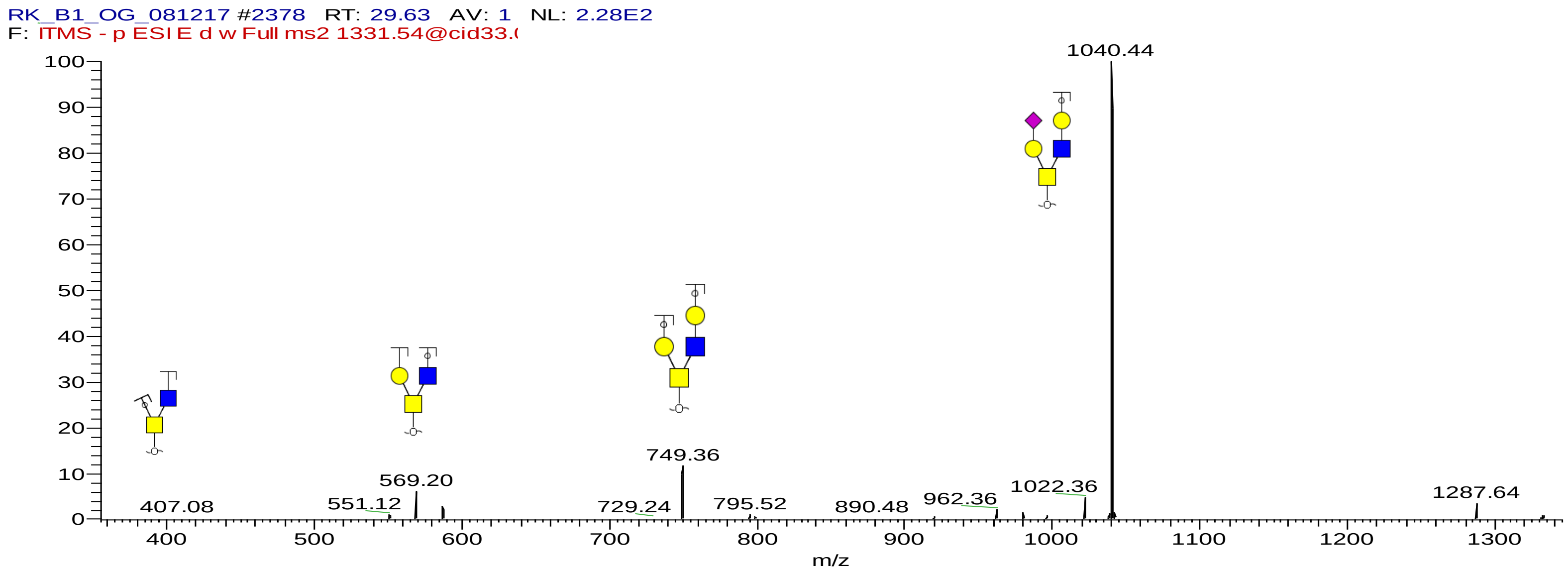

(Hex)3 (HexNAc)3
GlytouCan ID: G40107NH
[M-H] 1114.5

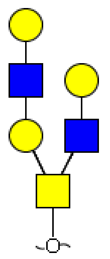

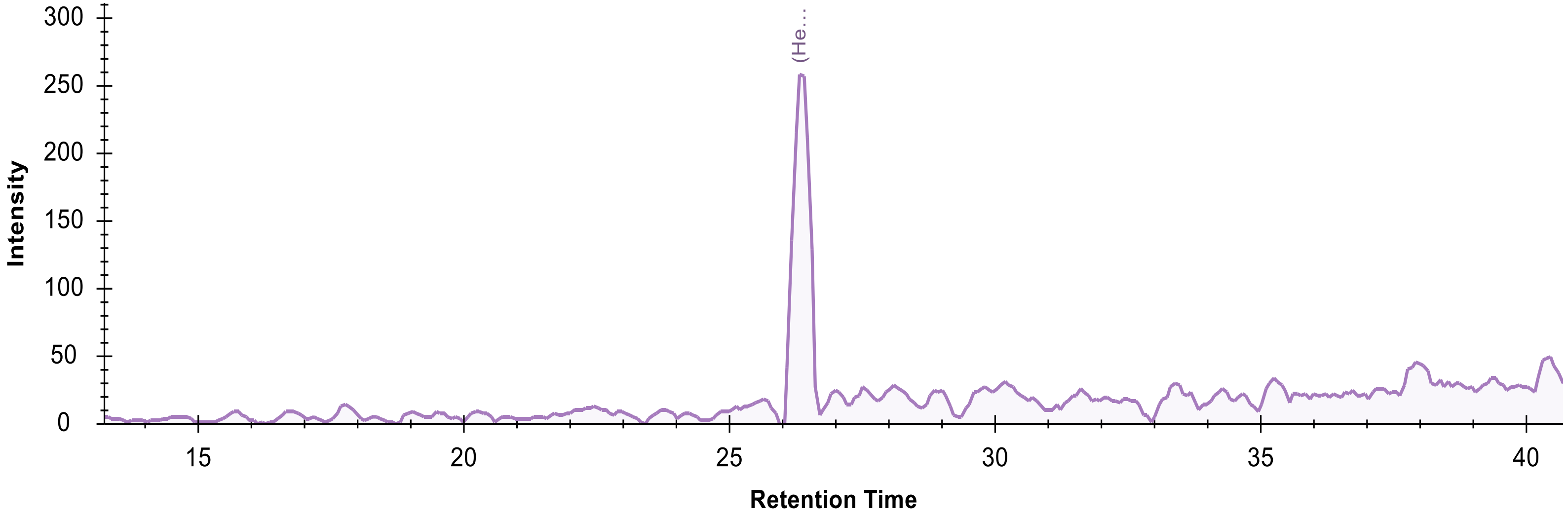

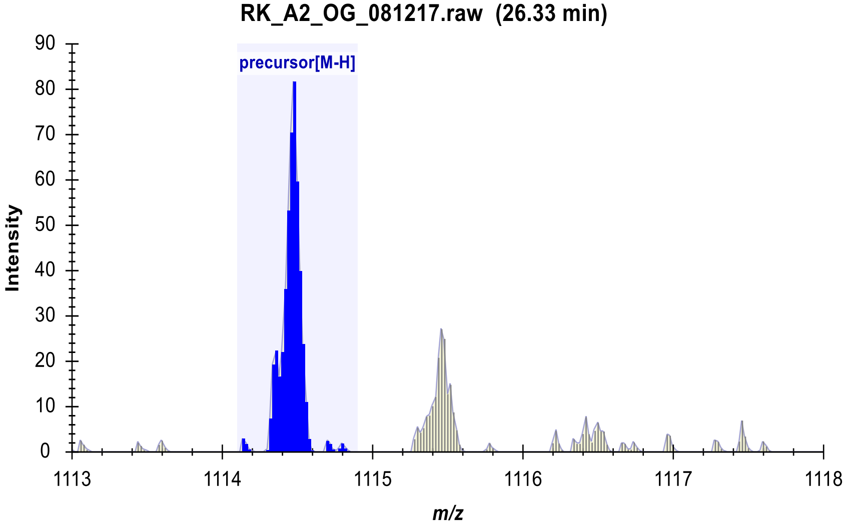

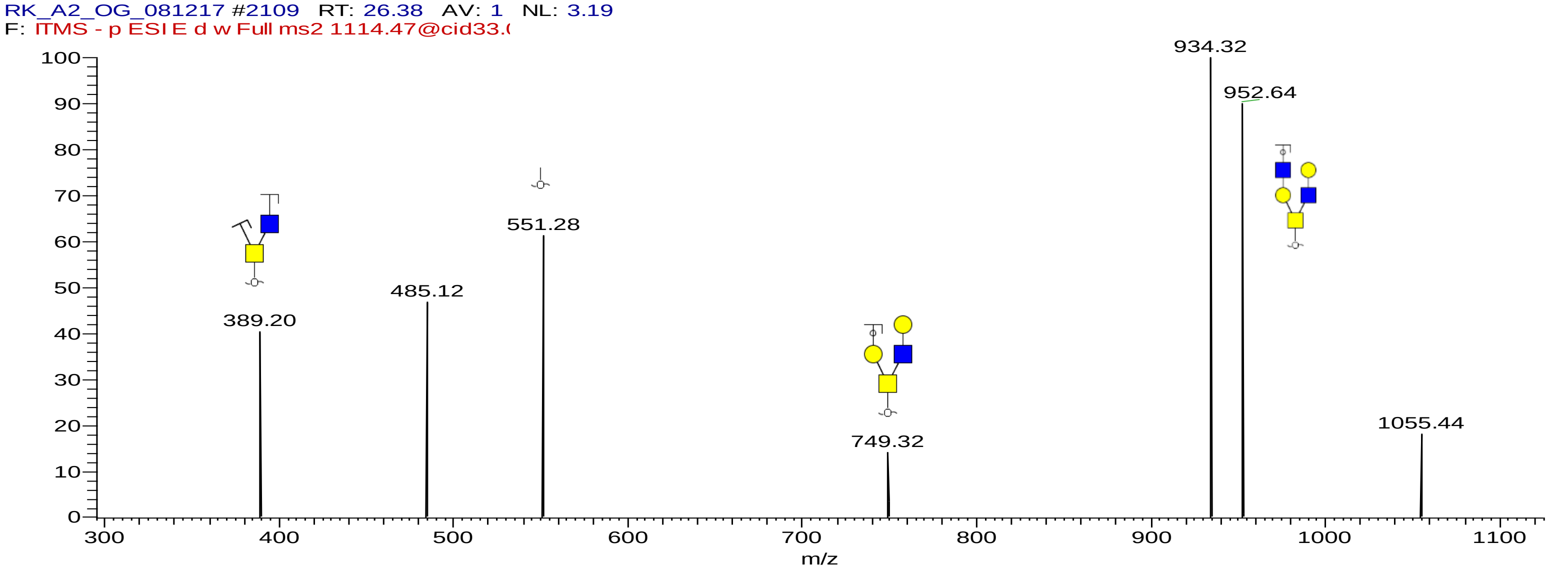

Glycan ID: 96
